# Supplementary material for: Non-autophagic Golgi-LC3 lipidation facilitates TFE3 stress response against Golgi dysfunction
Source: EMBO J. 2024 Sep 16;43(21):5085–113. doi: 10.1038/s44318-024-00233-y (PMC11535212; doi:10.1038/s44318-024-00233-y)
Supplement: Supplementary file 1 — Appendix [file 44318_2024_233_MOESM1_ESM.pdf]

Appendix

Non-autophagic Golgi-LC3 lipidation facilitates TFE3 stress response against Golgi dysfunction

Jaemin Kang[1], Cathena Meiling Li[1], Namhoon Kim[2], Jongyeon Baek[1], and Yong-Keun Jung[1,2*]

[1]*School of biological sciences, Seoul National University, Seoul 08826, Korea*

[2]*Interdisciplinary Program in Neuroscience, Seoul National University, Seoul 08826, Republic of Korea*

Table of Contents

Appendix Figure S1 .... Page 2

Appendix Figure S2 .... Page 3

Appendix Figure S3 .... Page 4

Appendix Figure S4 .... Page 5

Appendix Figure S5 .... Page 6

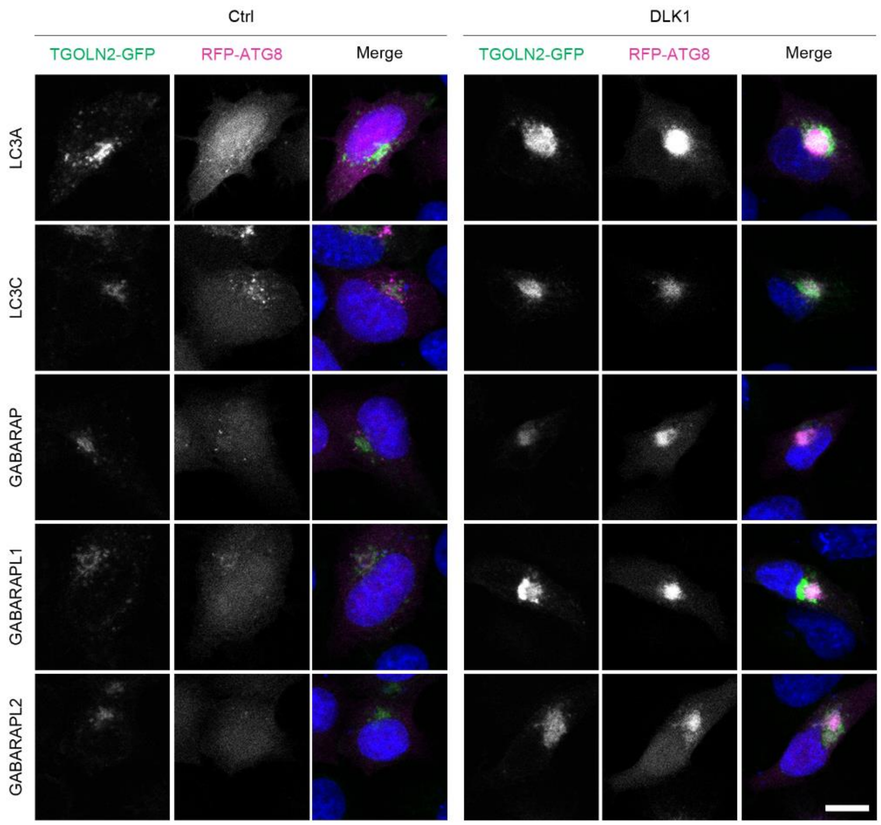

Appendix Figure S1. DLK1 overexpression accumulates ATG8 family proteins on the Golgi apparatus.

Confocal images of HeLa cells expressing DLK1-HA together with TGOLN2-GFP and one of the RFP-ATG8 family proteins. Nuclei were stained by Hoechst dye 33342. Scale bar, 10 μm.

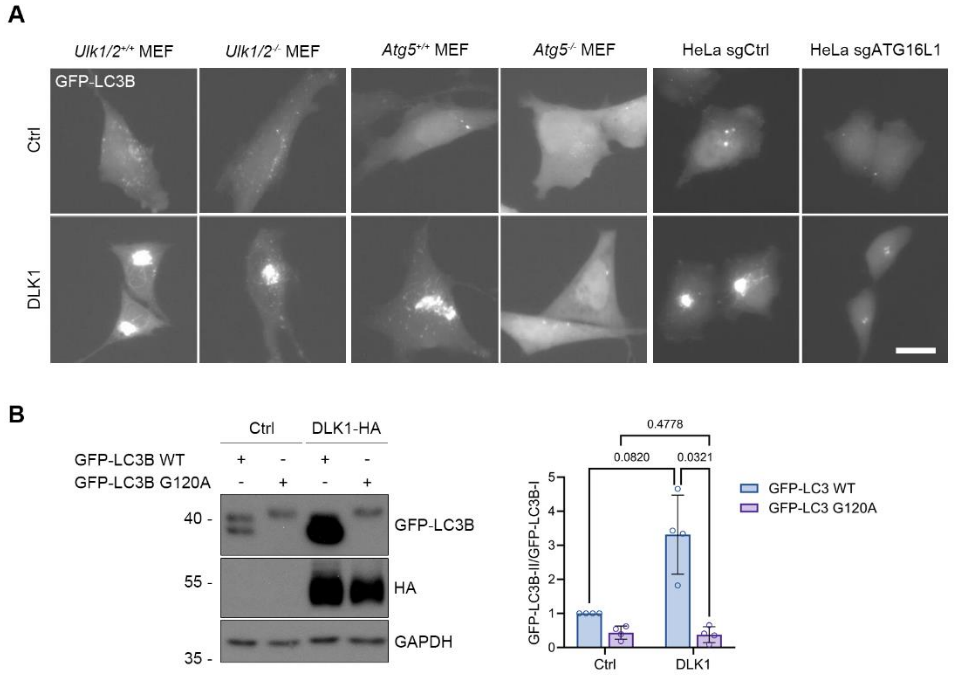

Appendix Figure S2. LC3 lipidation of Golgi by DLK1 is ATG12-ATG5-ATG16L1 complex-dependent.

(A) Fluorescence microscopy images of *Ulk1/2*$^{+/+}$, *Ulk1/2*$^{-/-}$, *Atg5*$^{+/+}$, *Atg5*$^{-/-}$ MEFs and HeLa sgCtrl, sgATG16L1 cells expressing GFP-LC3B and either pcDNA3-HA (Ctrl) or DLK1-HA. Scale bar, 20 µm.

(B) Immunoblot analysis of HeLa cells expressing DLK1-HA and either GFP-LC3B WT or GFP-LC3B G120A (left). Relative signals of GFP-LC3B-II and GFP-LC3B-I on the blots are represented as mean ± s.d. ($n = 4$, two-way ANOVA followed by Tukey's multiple comparisons test) (right).

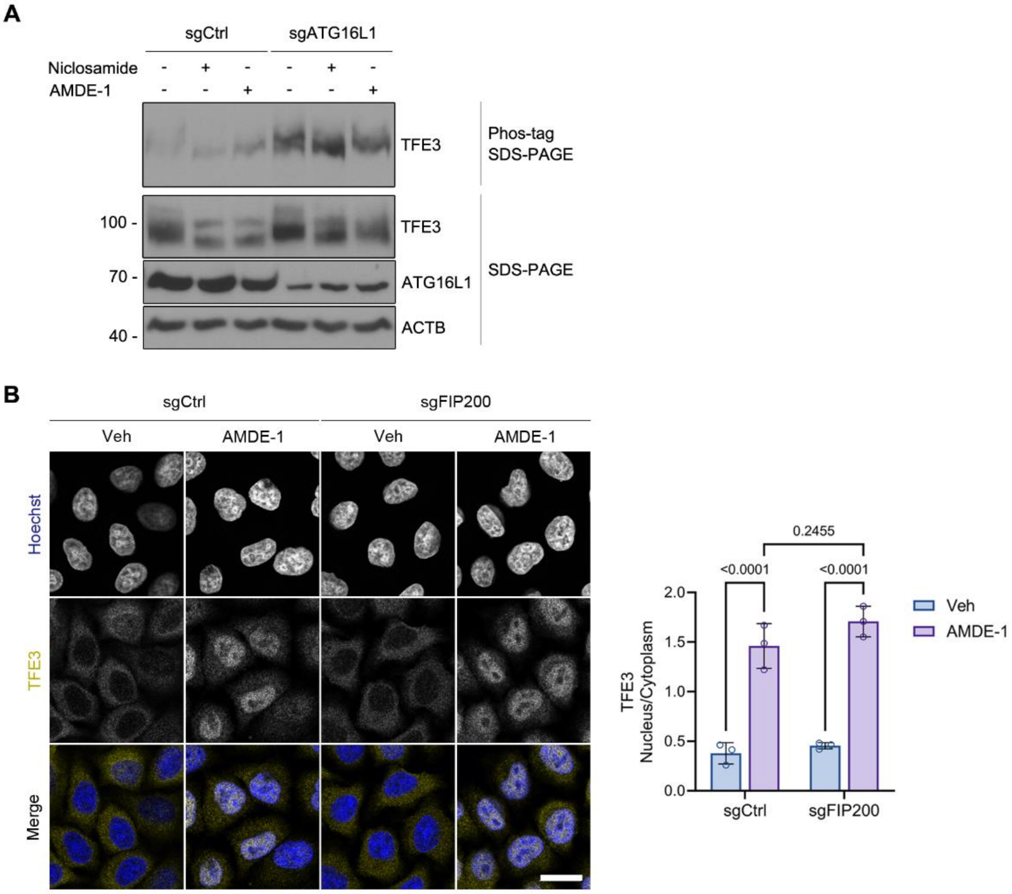

Appendix Figure S3. FIP200-independent TFE3 regulation under Golgi-stress.

(A) Phos-tag SDS-PAGE analysis of TFE3 in HeLa sgCtrl and sgATG16L1 cells after incubation with 10 µM niclosamide or 10 µM AMDE-1 for 6 h.

(B) Confocal images of HeLa sgCtrl and sgFIP200 cells incubated with 10 µM AMDE-1 for 6 h and immunostained with anti-TFE3 antibody. Nuclei were stained by Hoechst dye 33342. Scale bar, 20 µm (left). The nucleus/cytoplasm ratio of TFE3 fluorescence intensity is represented as mean ± s.d. (n = 3, 154 ~ 216 cells per experiment, two-way ANOVA followed by Tukey's multiple comparisons test) (right).

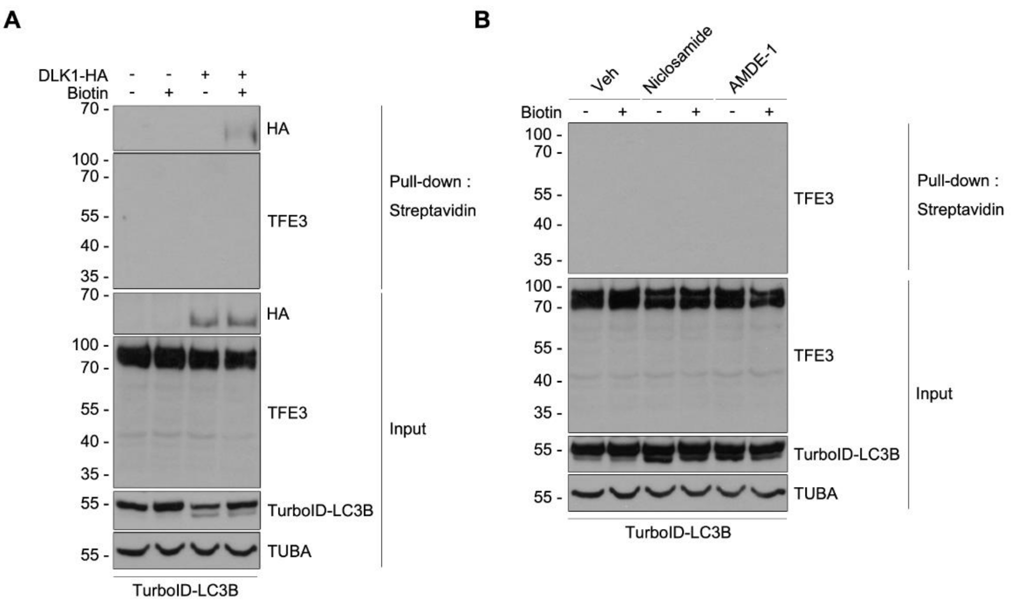

Appendix Figure S4. LC3 does not directly bind to TFE3 under Golgi stress.

(A) HeLa cells expressing DLK1-HA and TurboID-LC3B were treated with 100 µM biotin for 6 h, lysed and incubated with streptavidin-agarose beads. Enriched biotinylated proteins (Pull-down: Streptavidin) and whole cell lysates (Input) were analyzed by immunoblotting.

(B) HeLa cells expressing TurboID-LC3B were treated with 10 µM niclosamide or 10 µM AMDE-1 together with 100 µM biotin for 6 h, lysed and incubated with streptavidin-agarose beads. Enriched biotinylated proteins (Pull-down: Streptavidin) and whole cell lysates (Input) were analyzed by immunoblotting.

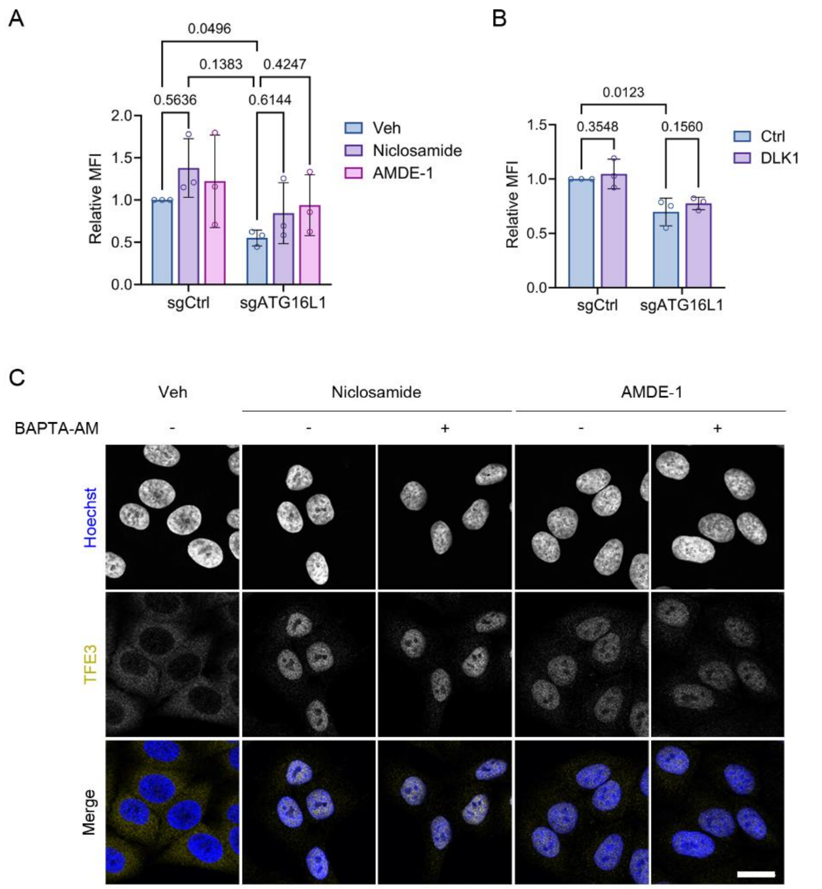

Appendix Figure S5. Calcium-independent TFE3 regulation by LC3 lipidation under Golgi stress.

(A, B) HeLa sgCtrl and sgATG16L1 cells exposed to 10 μM niclosamide or 10 μM AMDE-1 for 6 h **(A)** or HeLa sgCtrl and sgATG16L1 cells expressing pcDNA3-HA (Ctrl) or DLK1-HA **(B)** were treated with 1 μM Fluo-4 AM and subjected to flow cytometry analysis. Median values of the excitation ratios are represented as mean ± s.d. ($n = 3$, two-way ANOVA followed by Tukey's multiple comparisons test).

(C) HeLa cells were treated with 10 μM niclosamide or 10 μM AMDE-1 for 6 h in the presence or absence of 10 μM BAPTA-AM. Cells were immunostained with anti-TFE3 antibody and

observed by confocal microscopy. Nuclei were stained by Hoechst dye 33342. Scale bar, 20 μm.
