# Supplementary figures and images for: Non-autophagic Golgi-LC3 lipidation facilitates TFE3 stress response against Golgi dysfunction (part 1 of 3)
Source: EMBO J. 2024 Sep 16;43(21):5085–113. doi: 10.1038/s44318-024-00233-y (PMC11535212; doi:10.1038/s44318-024-00233-y)

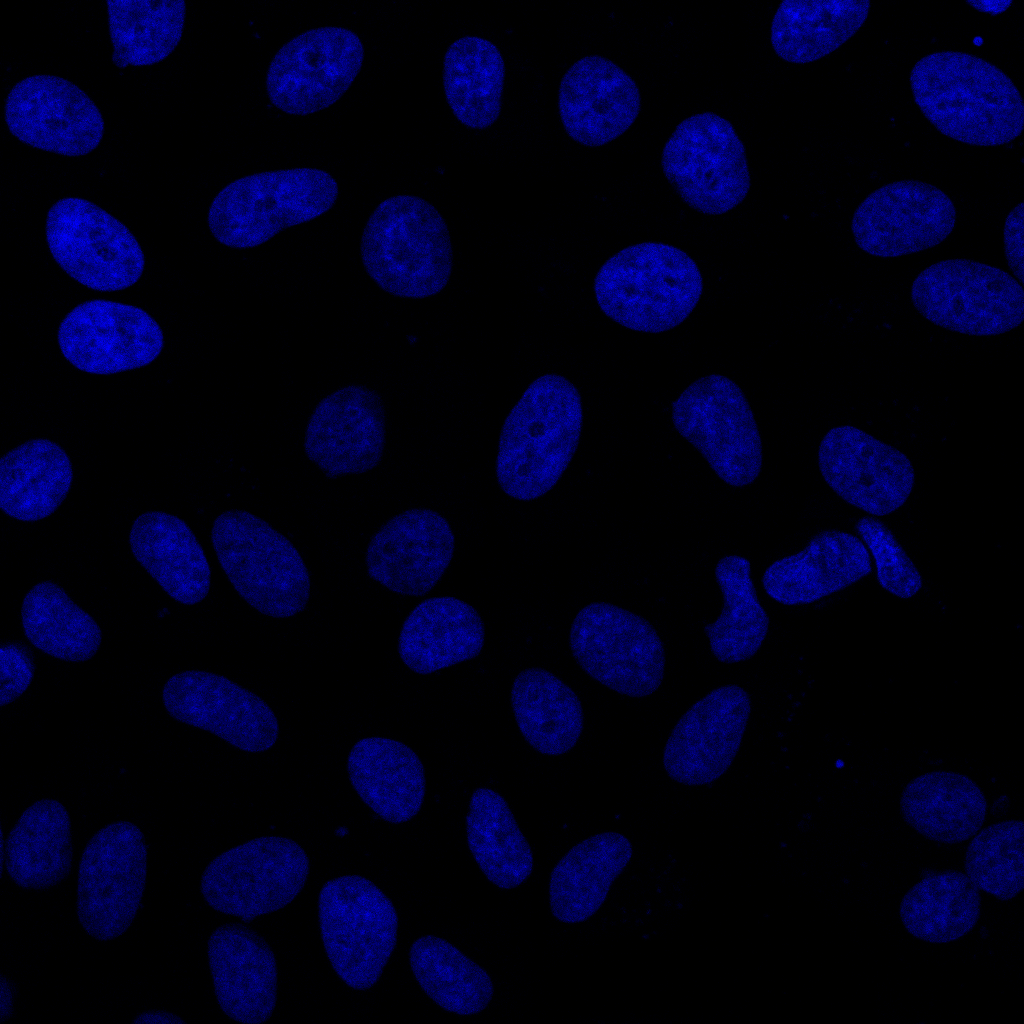

Supplement: Supplementary file 3 — Source data Fig. 1 [file 44318_2024_233_MOESM3_ESM.zip › 1A/HeLa Ctrl TGN GFP RFP LC3_Series005_ch00_SV.tif]

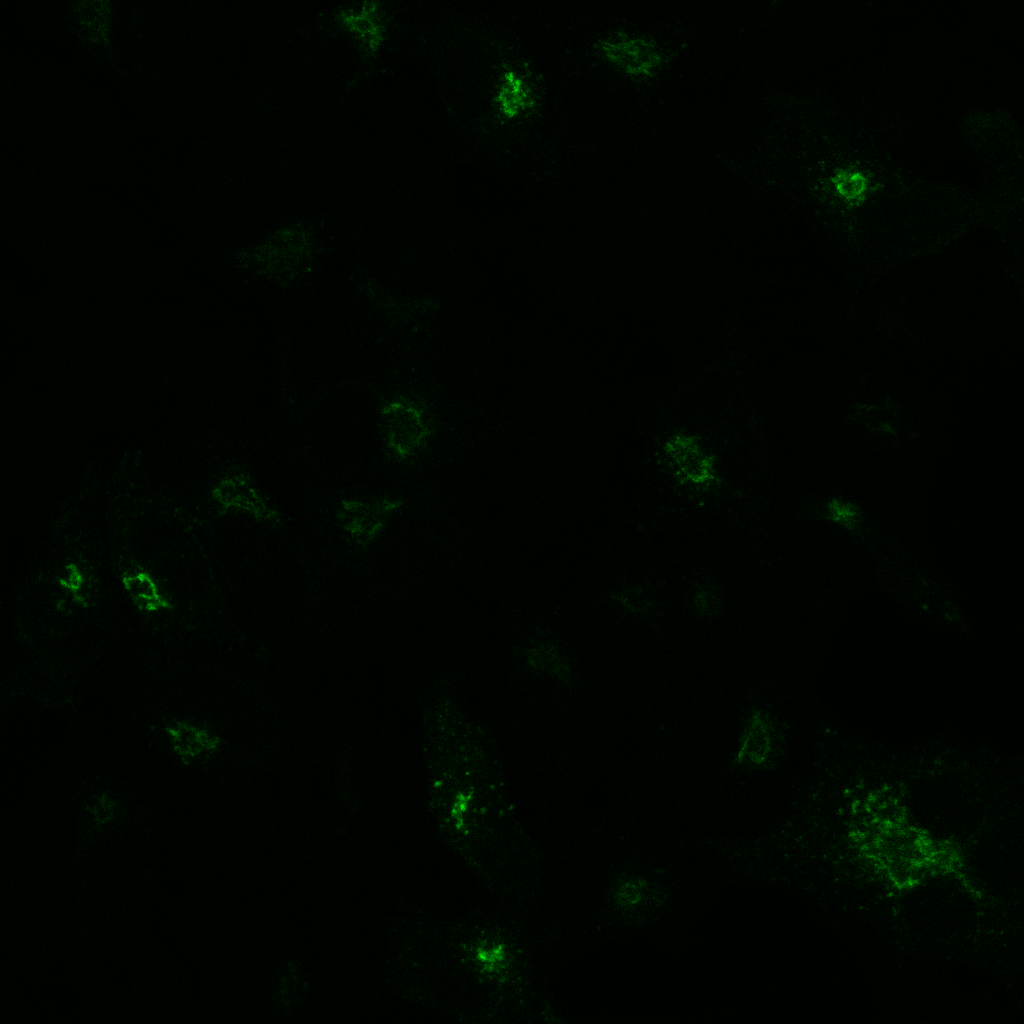

Supplement: Supplementary file 3 — Source data Fig. 1 [file 44318_2024_233_MOESM3_ESM.zip › 1A/HeLa Ctrl TGN GFP RFP LC3_Series005_ch01_SV.tif]

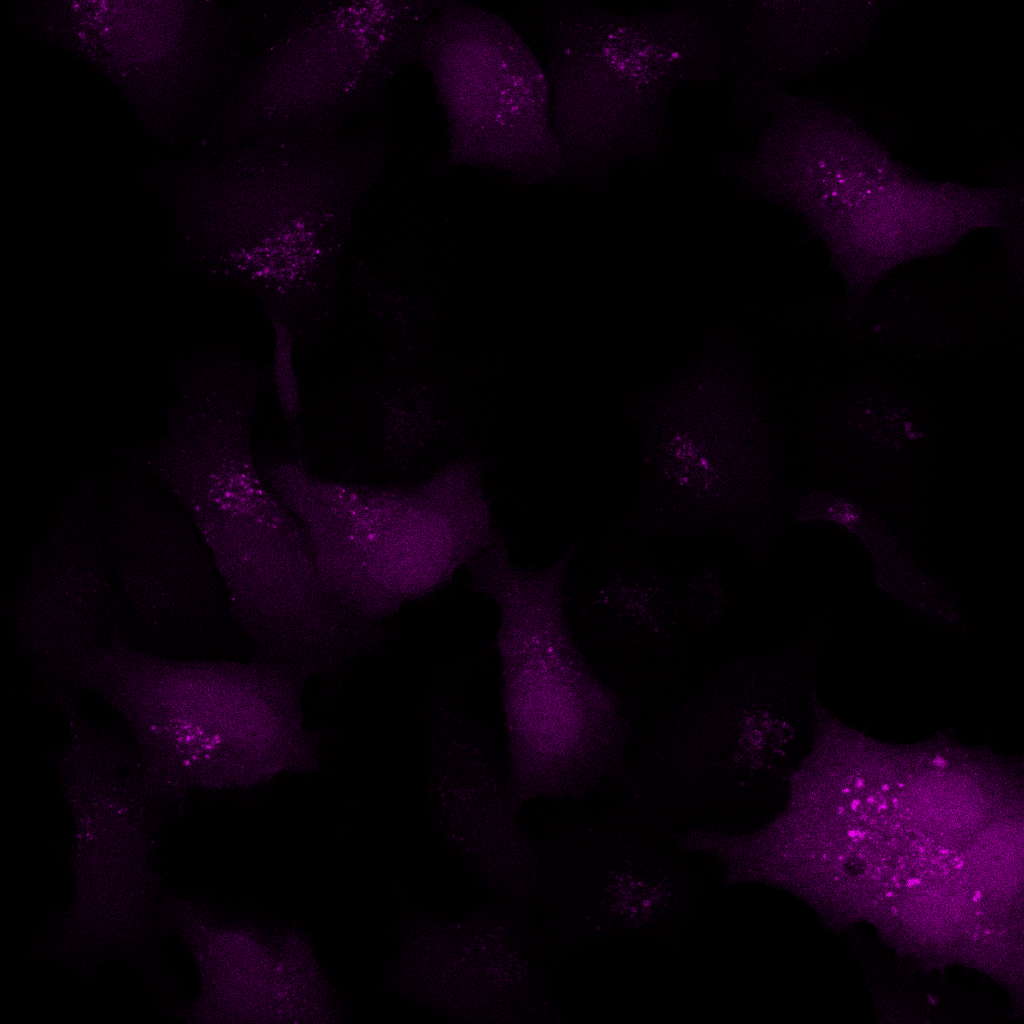

Supplement: Supplementary file 3 — Source data Fig. 1 [file 44318_2024_233_MOESM3_ESM.zip › 1A/HeLa Ctrl TGN GFP RFP LC3_Series005_ch02_SV.tif]

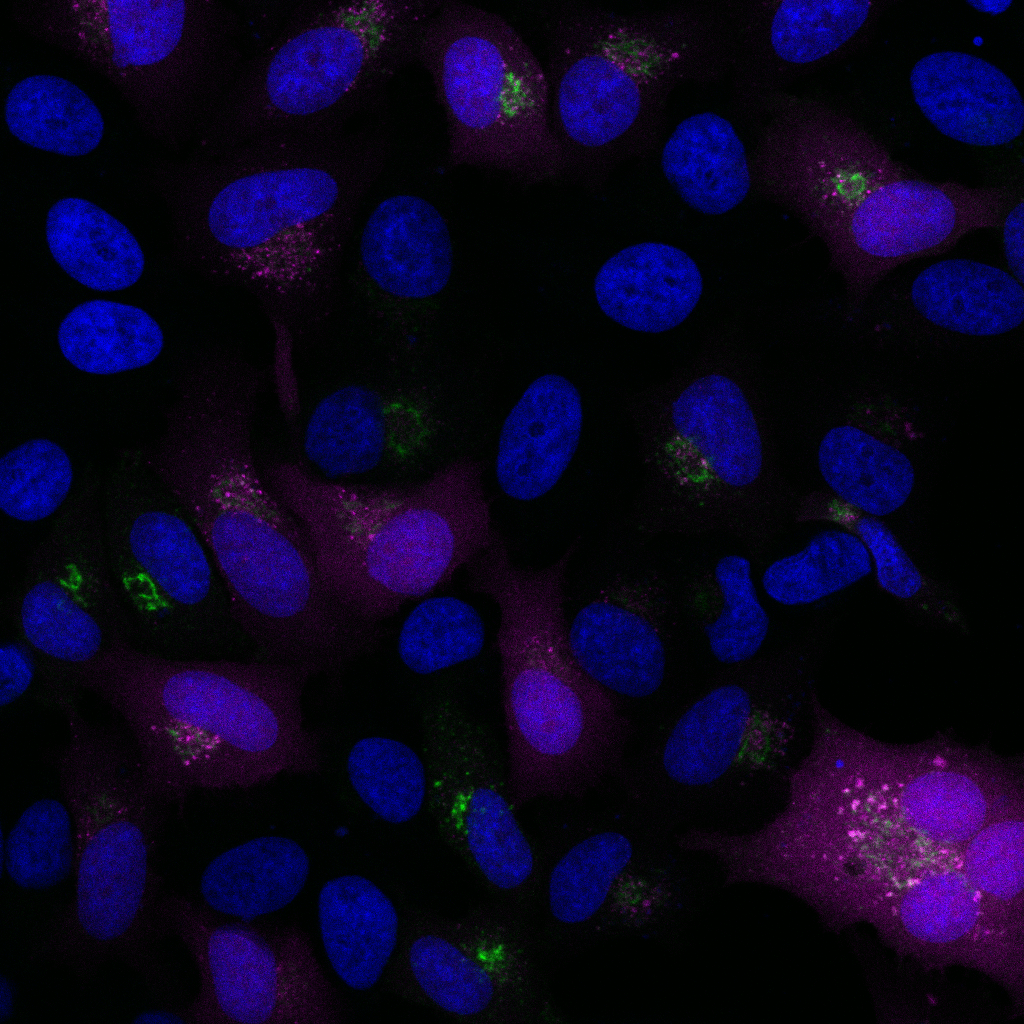

Supplement: Supplementary file 3 — Source data Fig. 1 [file 44318_2024_233_MOESM3_ESM.zip › 1A/HeLa Ctrl TGN GFP RFP LC3_Series005_overlay.tif]

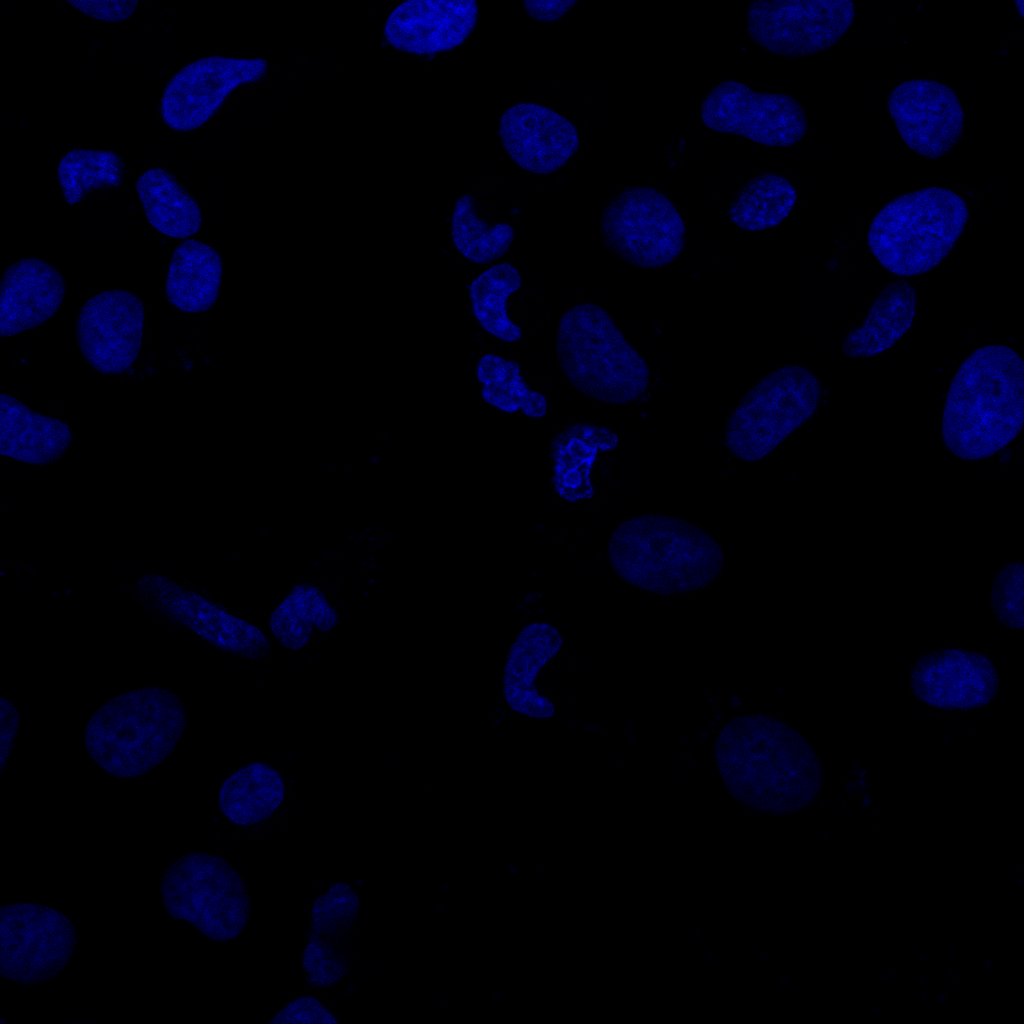

Supplement: Supplementary file 3 — Source data Fig. 1 [file 44318_2024_233_MOESM3_ESM.zip › 1A/HeLa DLK1 TGN GFP RFP LC3 _Series001_ch00_SV.tif]

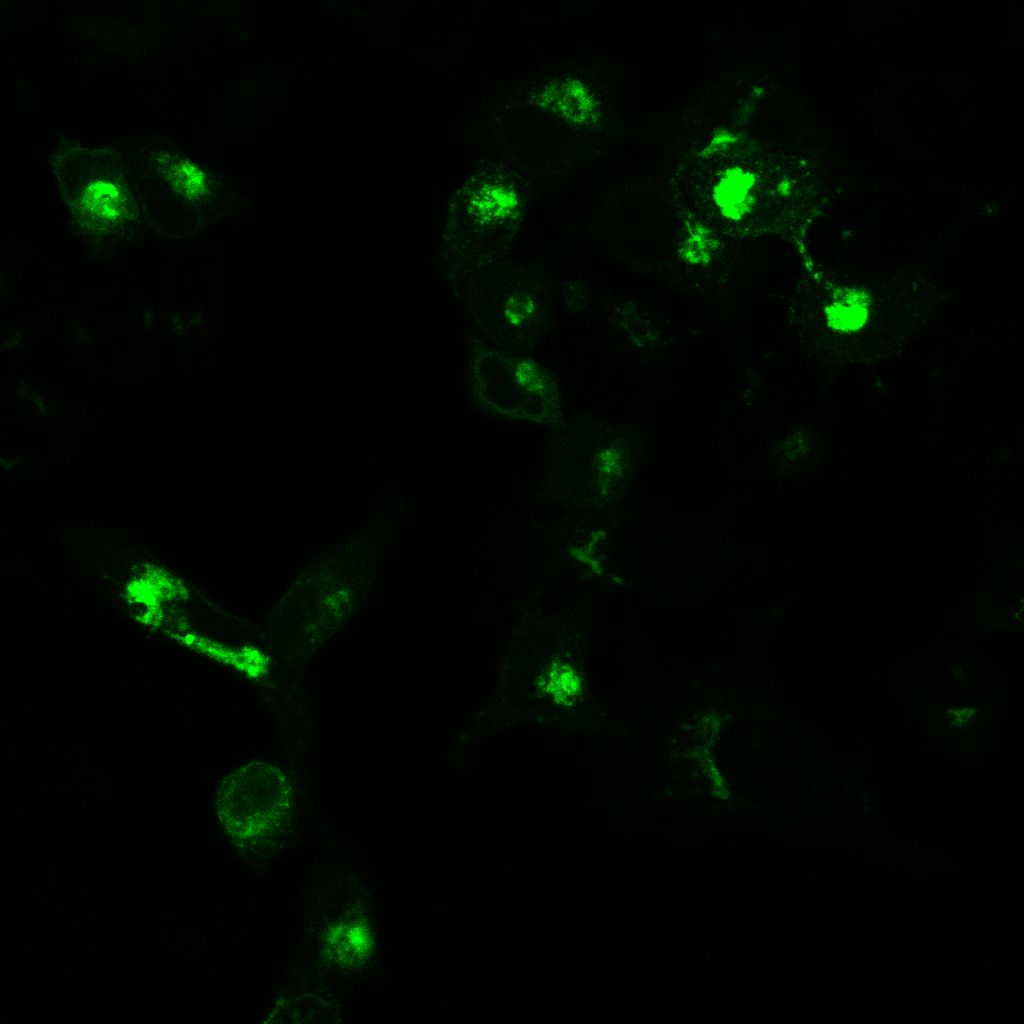

Supplement: Supplementary file 3 — Source data Fig. 1 [file 44318_2024_233_MOESM3_ESM.zip › 1A/HeLa DLK1 TGN GFP RFP LC3 _Series001_ch01_SV.tif]

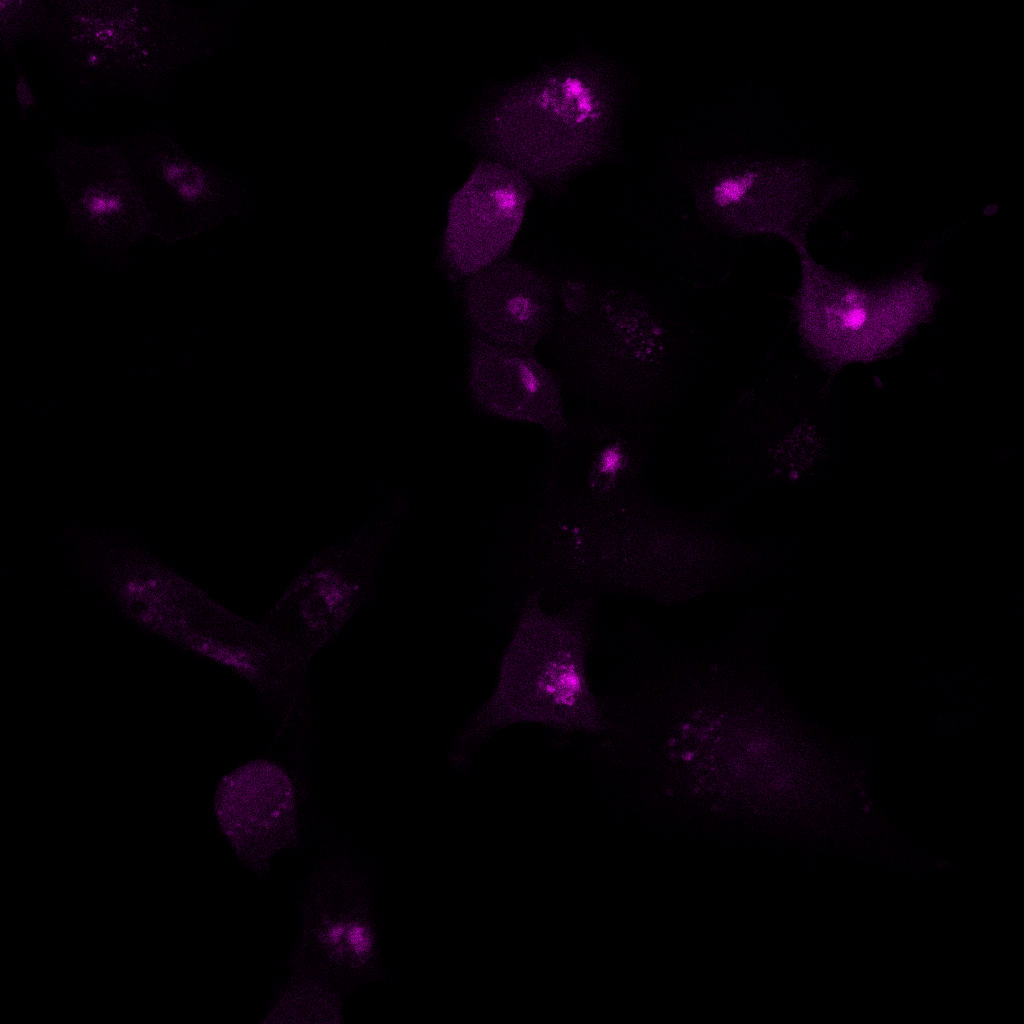

Supplement: Supplementary file 3 — Source data Fig. 1 [file 44318_2024_233_MOESM3_ESM.zip › 1A/HeLa DLK1 TGN GFP RFP LC3 _Series001_ch02_SV.tif]

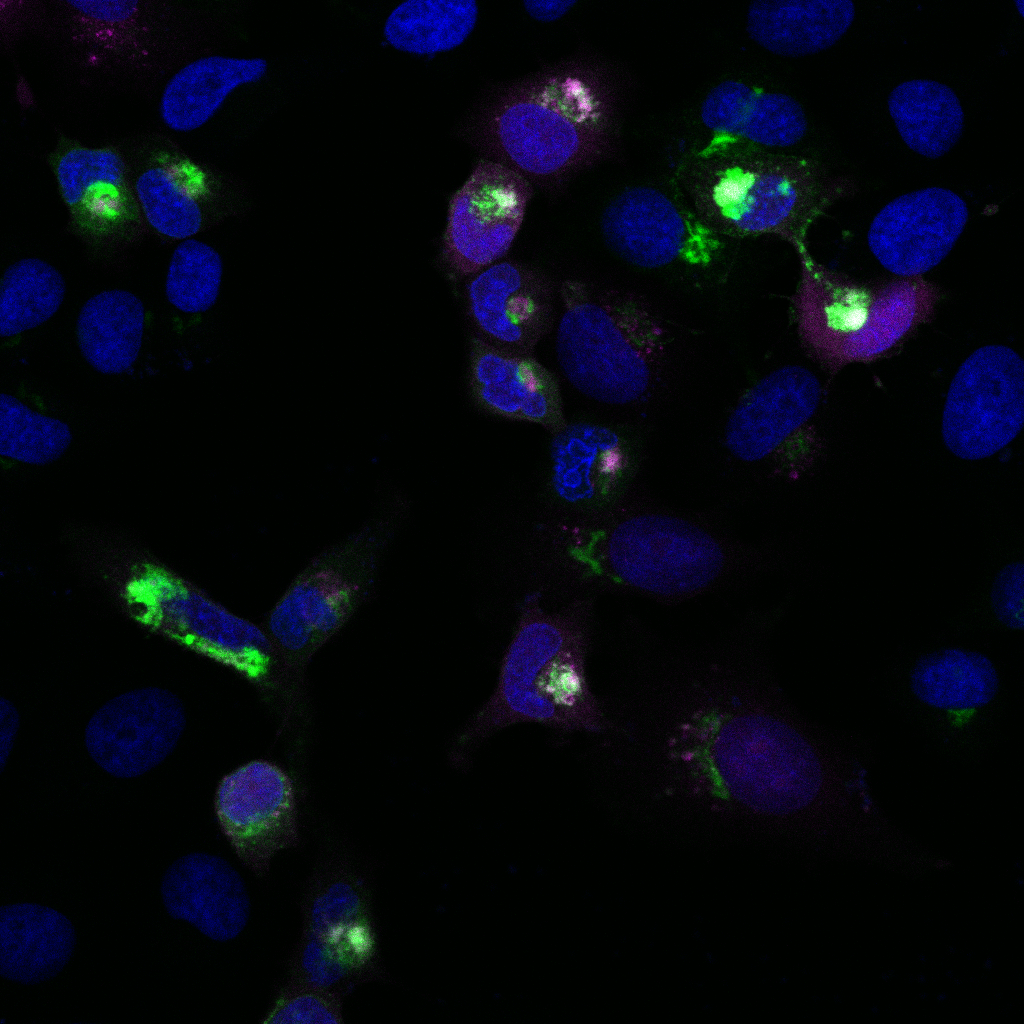

Supplement: Supplementary file 3 — Source data Fig. 1 [file 44318_2024_233_MOESM3_ESM.zip › 1A/HeLa DLK1 TGN GFP RFP LC3 _Series001_overlay.tif]

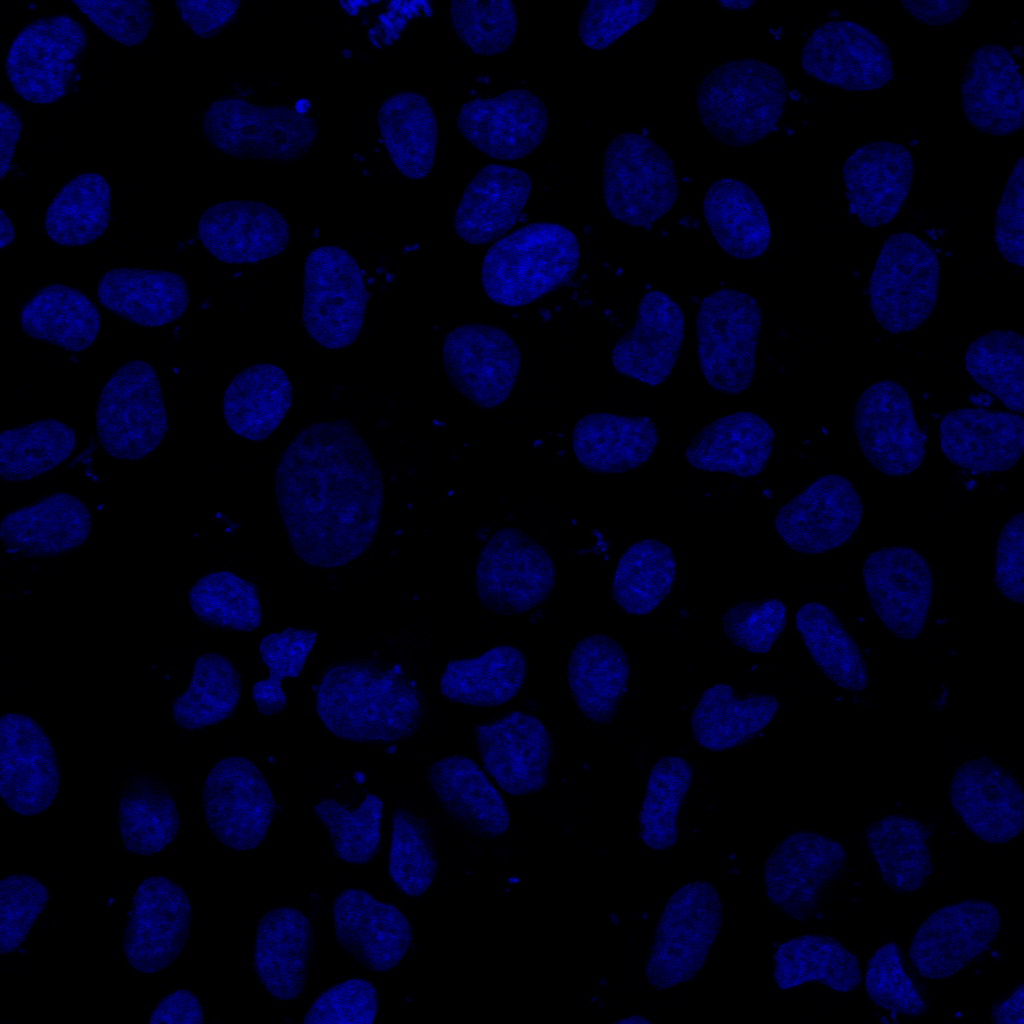

Supplement: Supplementary file 3 — Source data Fig. 1 [file 44318_2024_233_MOESM3_ESM.zip › 1B/Ctrl GFP LC3 GM130_Series003_ch00_SV.tif]

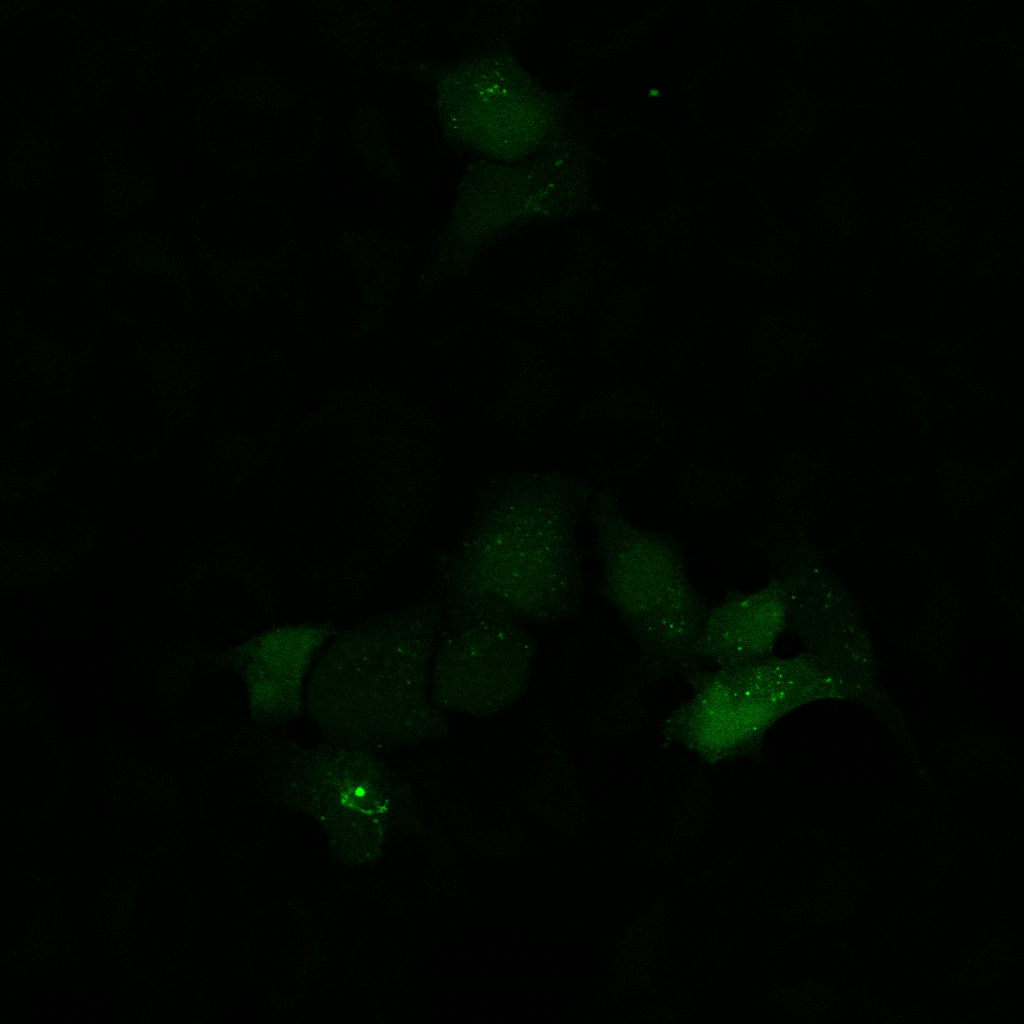

Supplement: Supplementary file 3 — Source data Fig. 1 [file 44318_2024_233_MOESM3_ESM.zip › 1B/Ctrl GFP LC3 GM130_Series003_ch01_SV.tif]

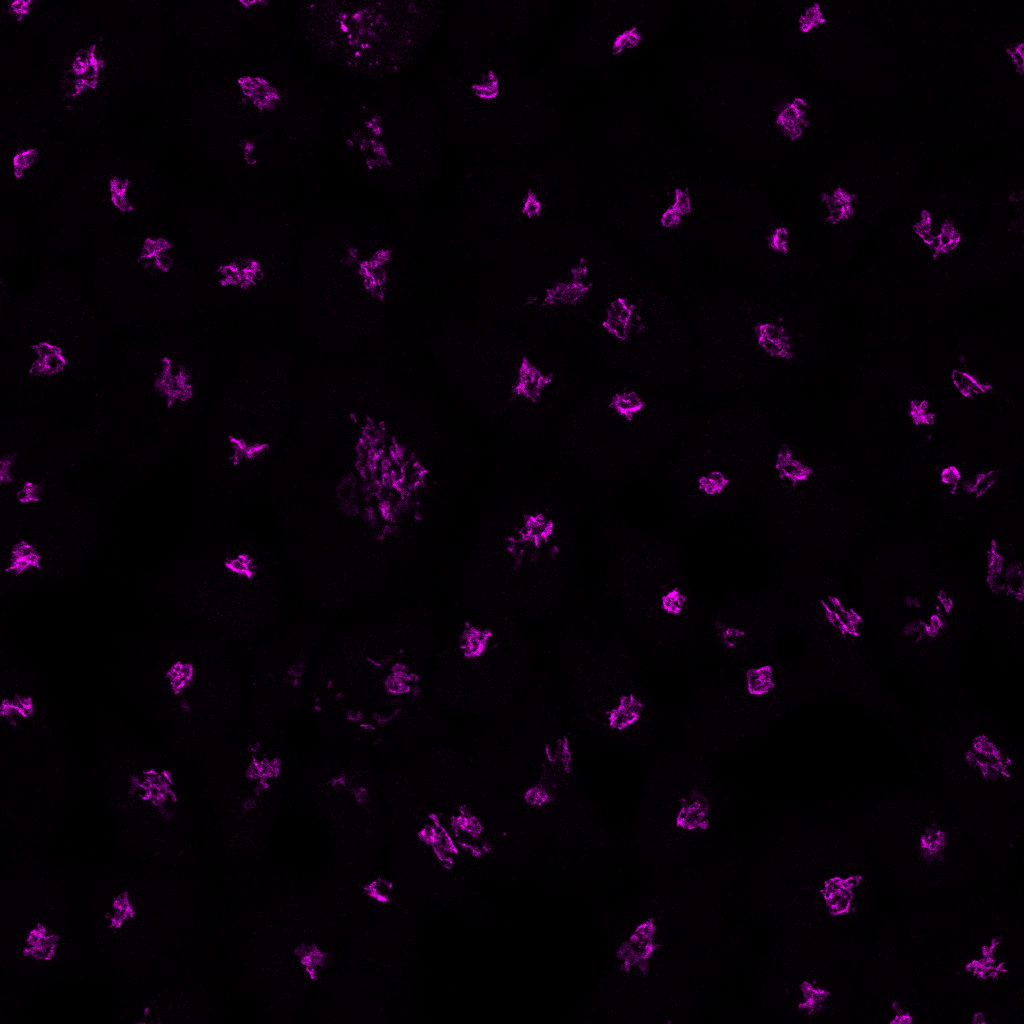

Supplement: Supplementary file 3 — Source data Fig. 1 [file 44318_2024_233_MOESM3_ESM.zip › 1B/Ctrl GFP LC3 GM130_Series003_ch02_SV.tif]

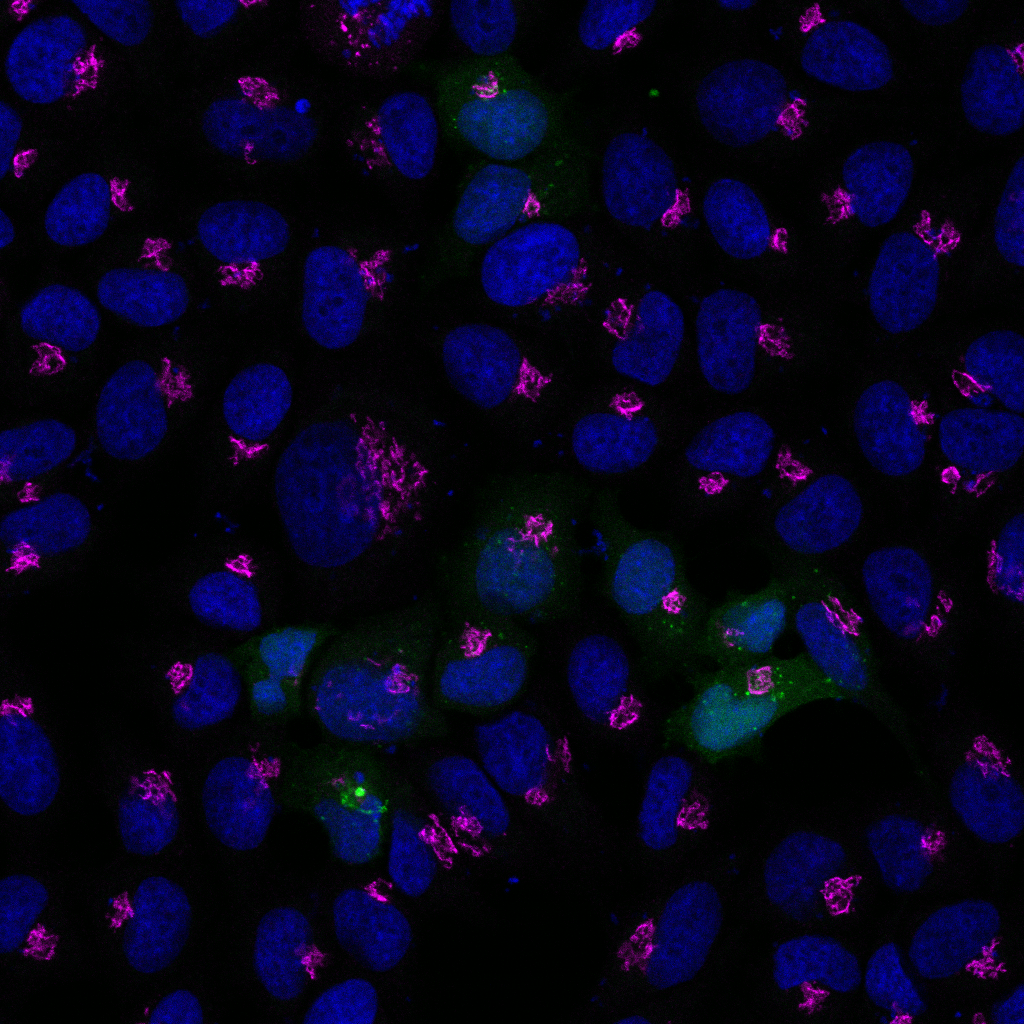

Supplement: Supplementary file 3 — Source data Fig. 1 [file 44318_2024_233_MOESM3_ESM.zip › 1B/Ctrl GFP LC3 GM130_Series003_overlay.tif]

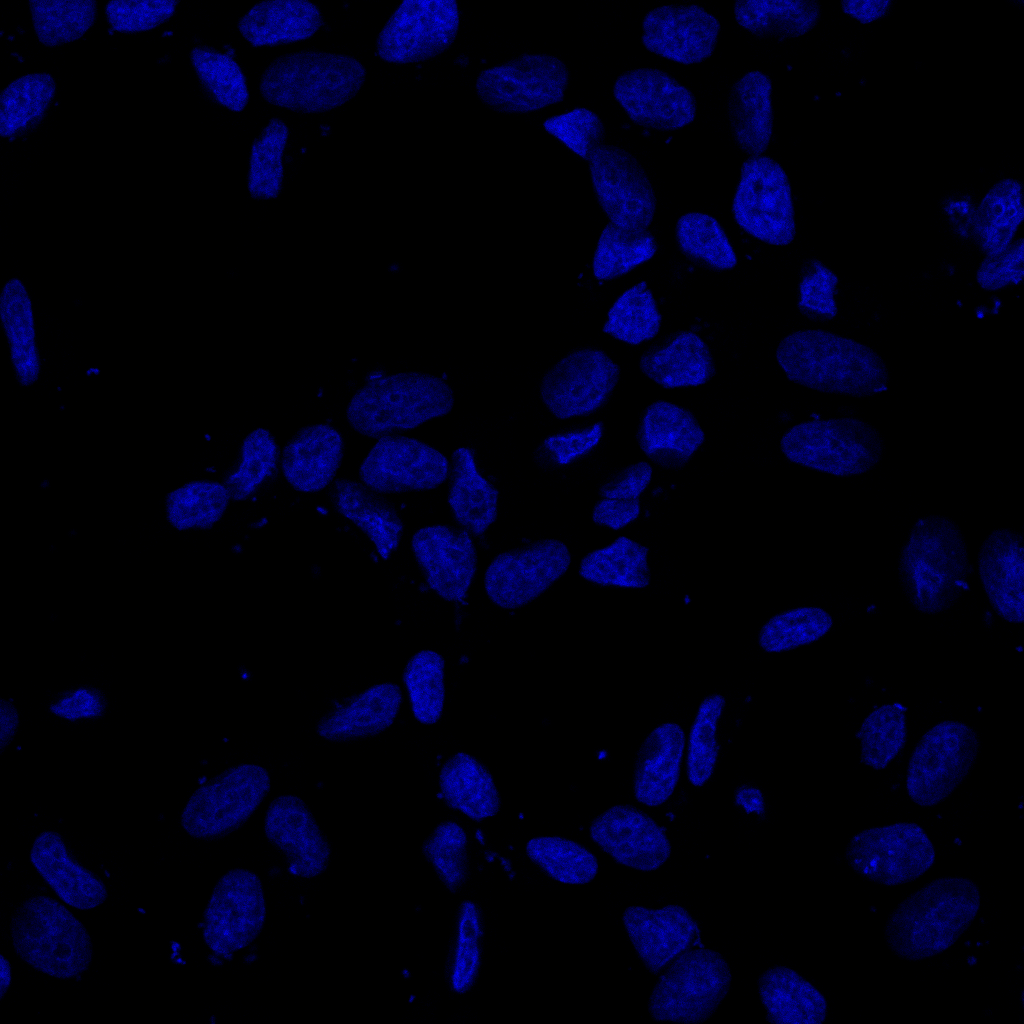

Supplement: Supplementary file 3 — Source data Fig. 1 [file 44318_2024_233_MOESM3_ESM.zip › 1B/DLK1 GFP LC3 GM130_Series009_ch00_SV.tif]

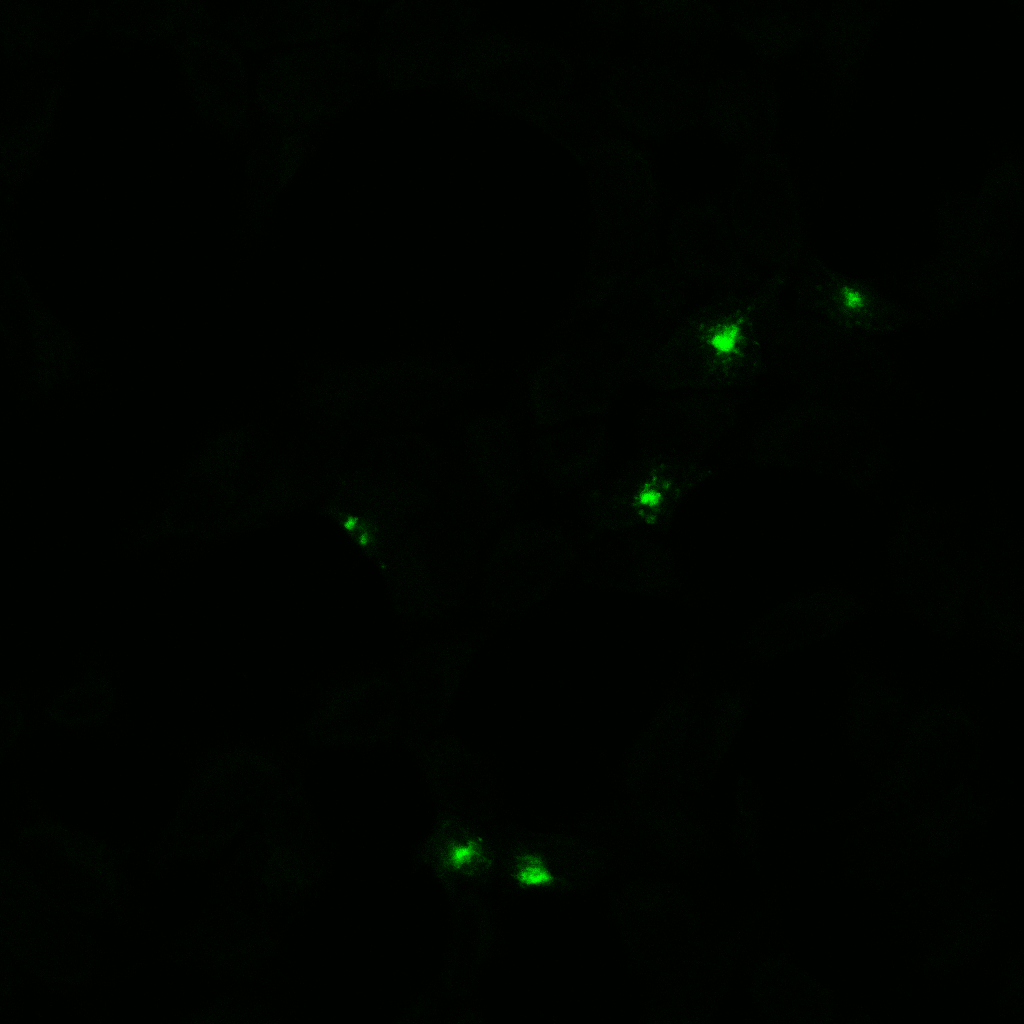

Supplement: Supplementary file 3 — Source data Fig. 1 [file 44318_2024_233_MOESM3_ESM.zip › 1B/DLK1 GFP LC3 GM130_Series009_ch01_SV.tif]

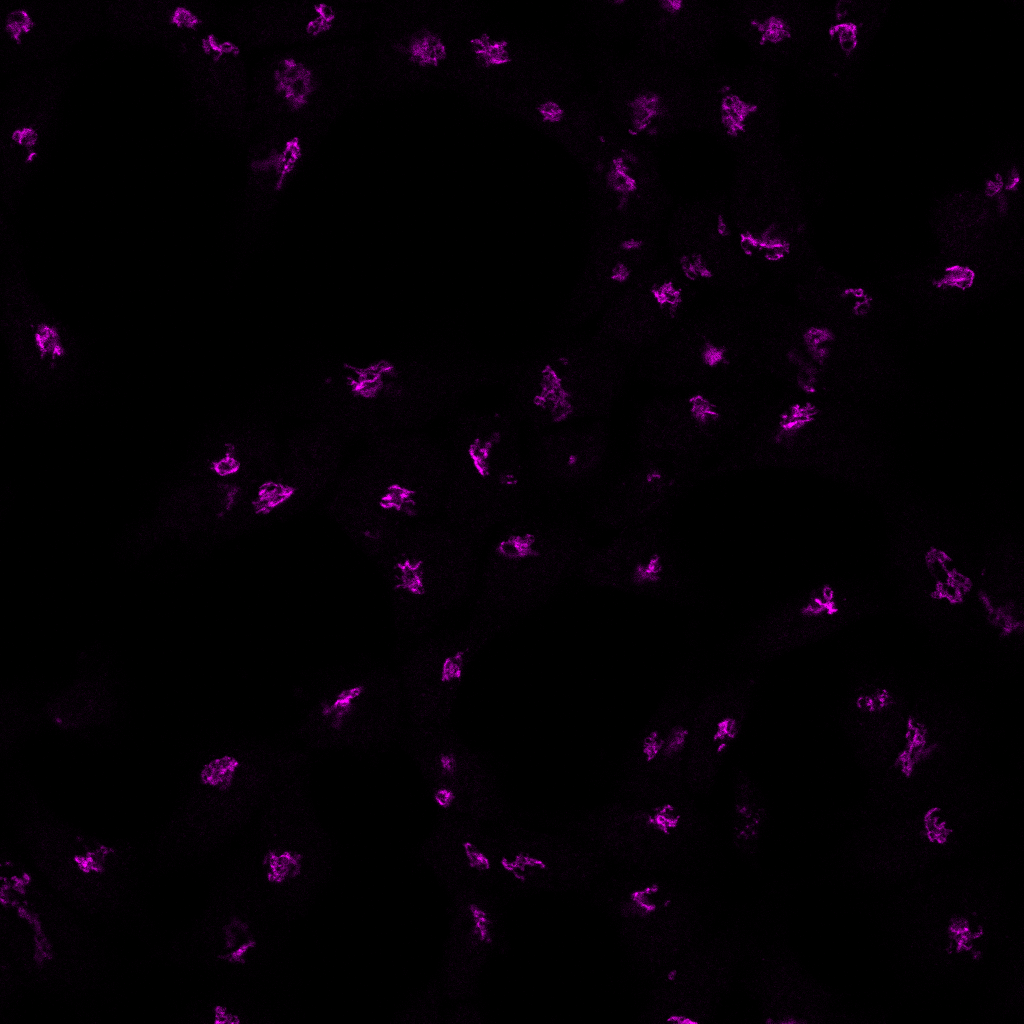

Supplement: Supplementary file 3 — Source data Fig. 1 [file 44318_2024_233_MOESM3_ESM.zip › 1B/DLK1 GFP LC3 GM130_Series009_ch02_SV.tif]

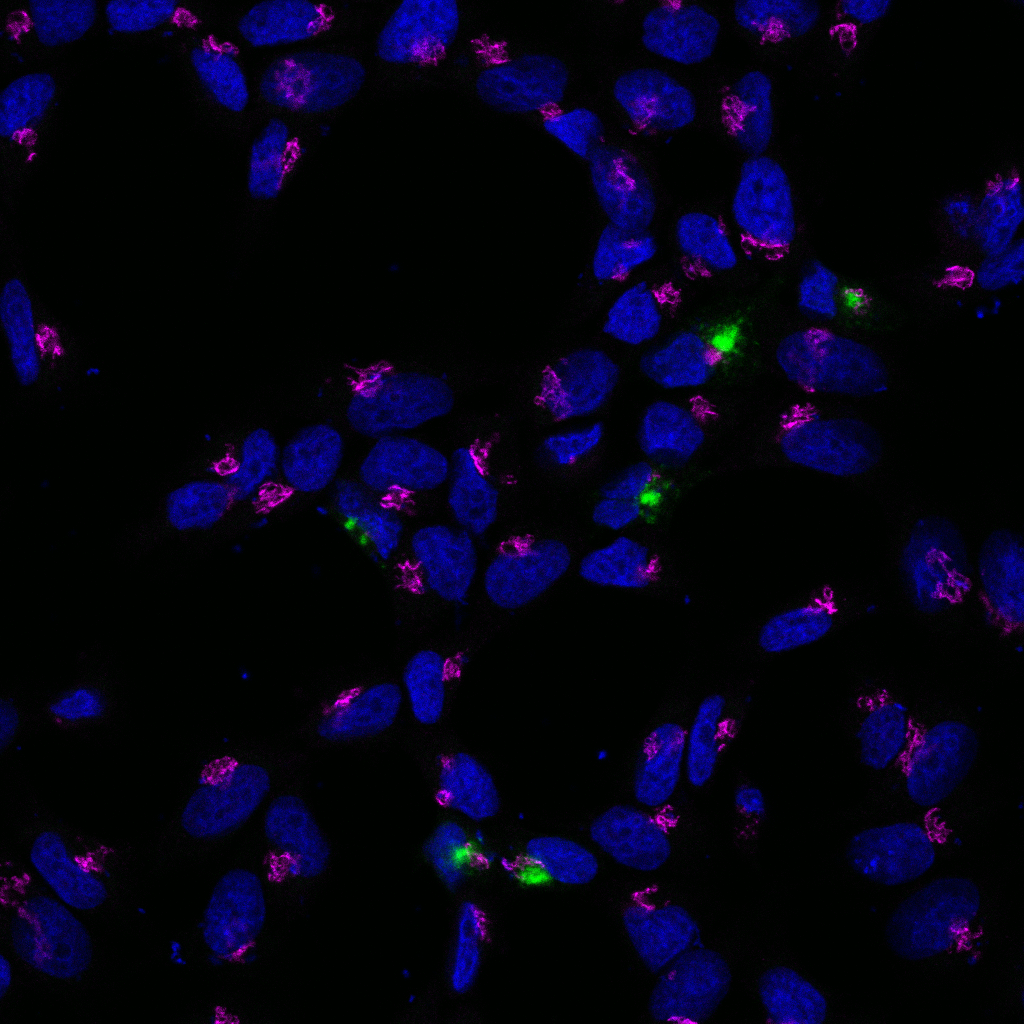

Supplement: Supplementary file 3 — Source data Fig. 1 [file 44318_2024_233_MOESM3_ESM.zip › 1B/DLK1 GFP LC3 GM130_Series009_overlay.tif]

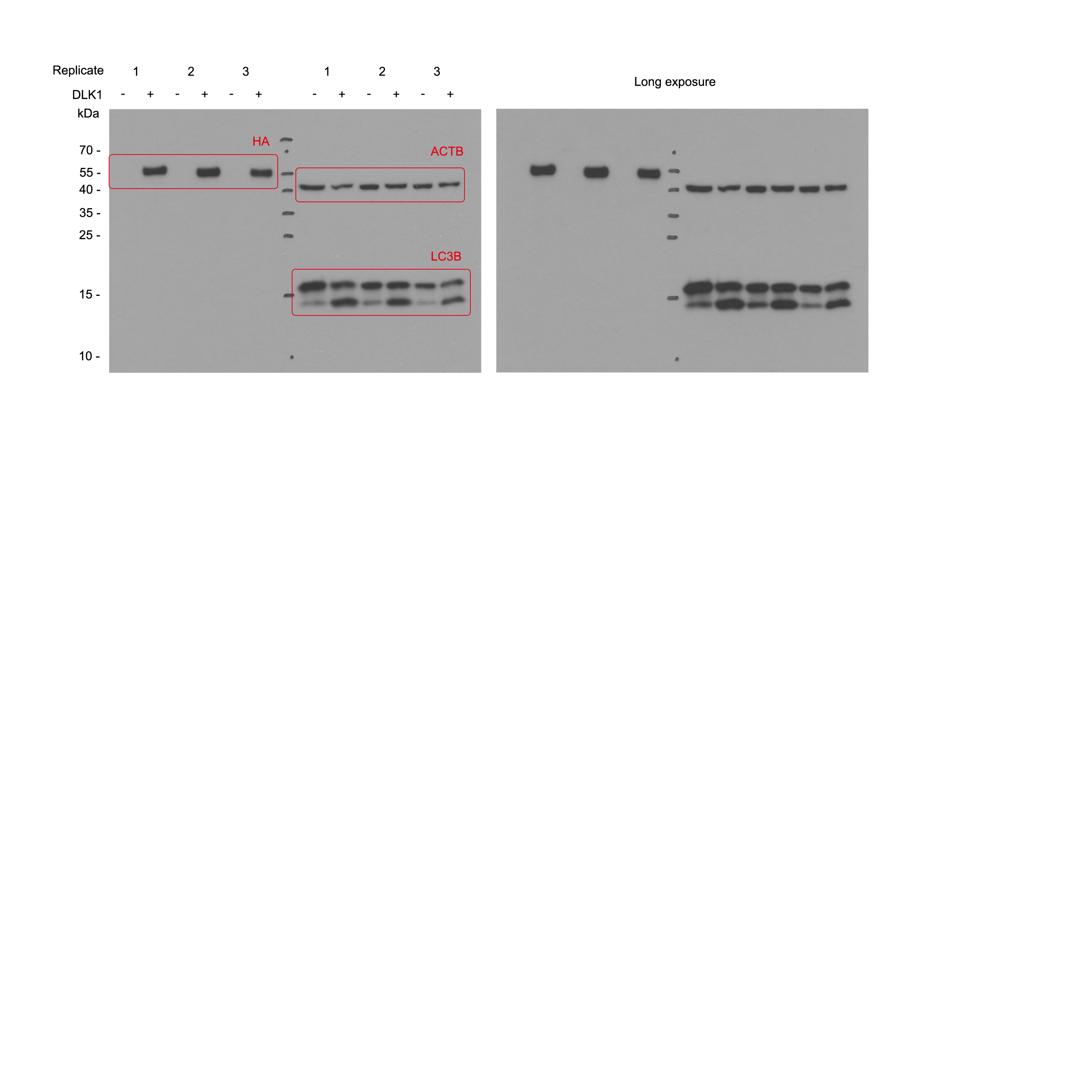

Supplement: Supplementary file 3 — Source data Fig. 1 [file 44318_2024_233_MOESM3_ESM.zip › 1E/Figure 1E.png]

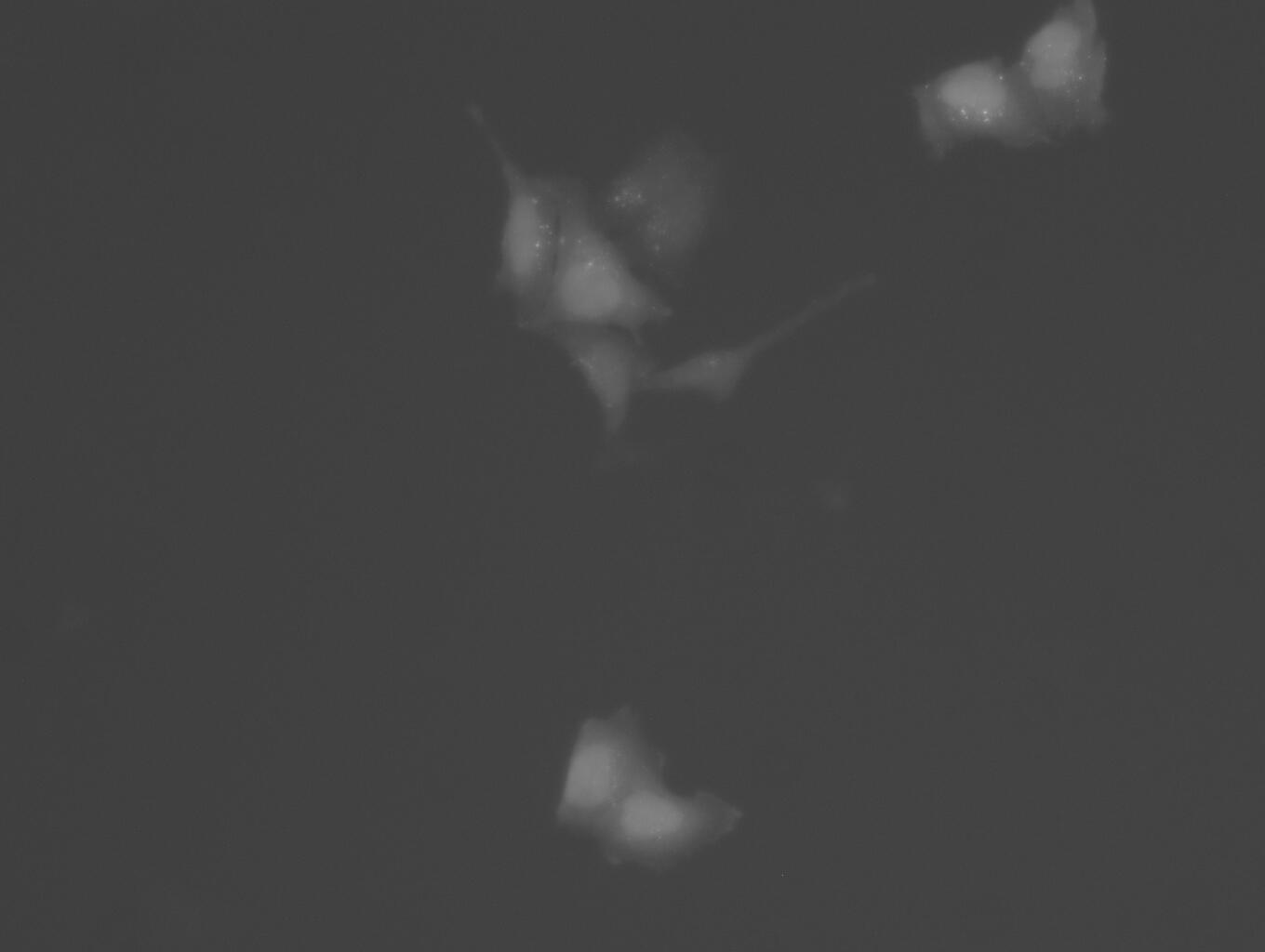

Supplement: Supplementary file 3 — Source data Fig. 1 [file 44318_2024_233_MOESM3_ESM.zip › 1G/Image/1. Ctrl-3.jpg]

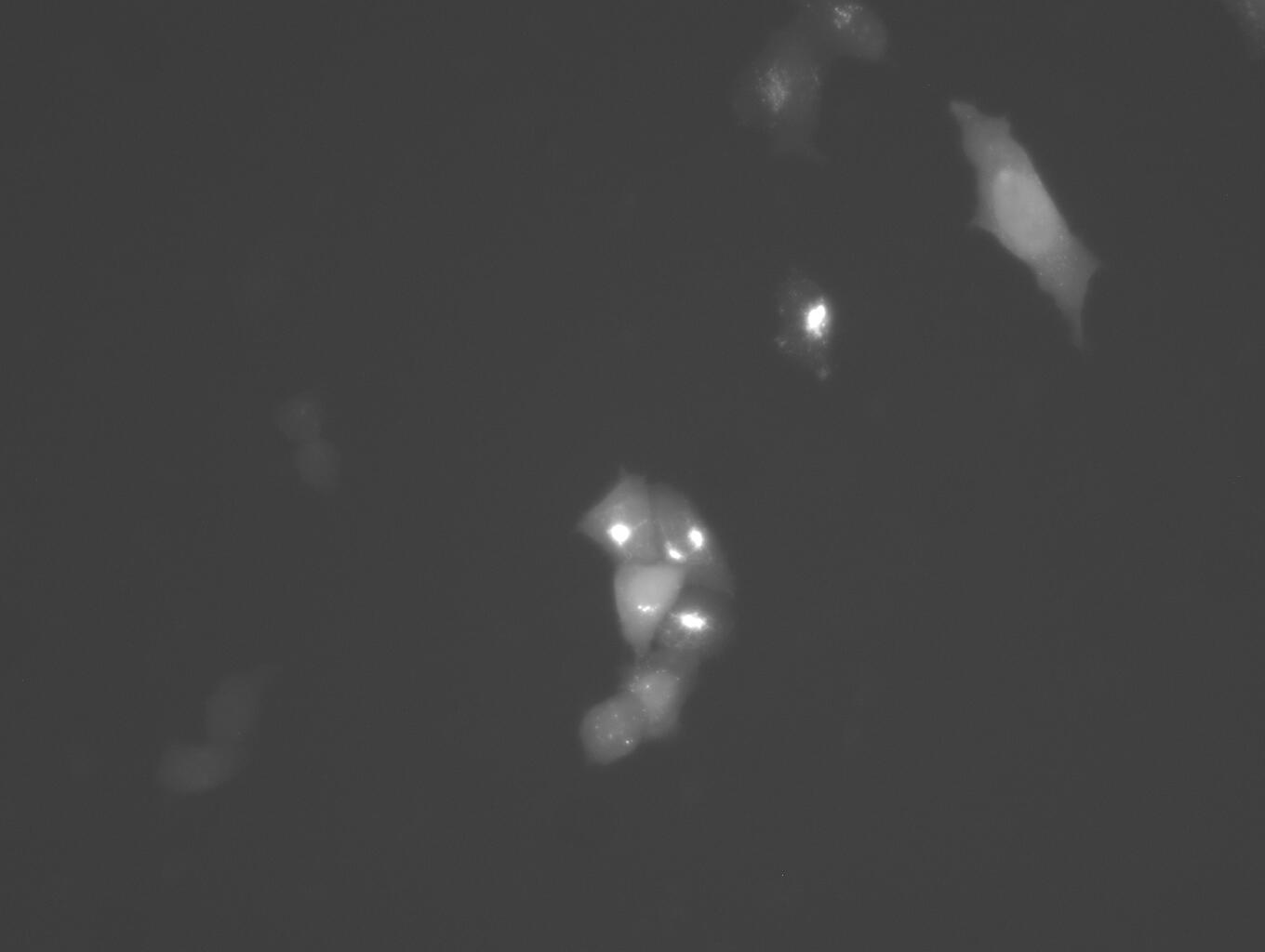

Supplement: Supplementary file 3 — Source data Fig. 1 [file 44318_2024_233_MOESM3_ESM.zip › 1G/Image/10. PR-1.jpg]

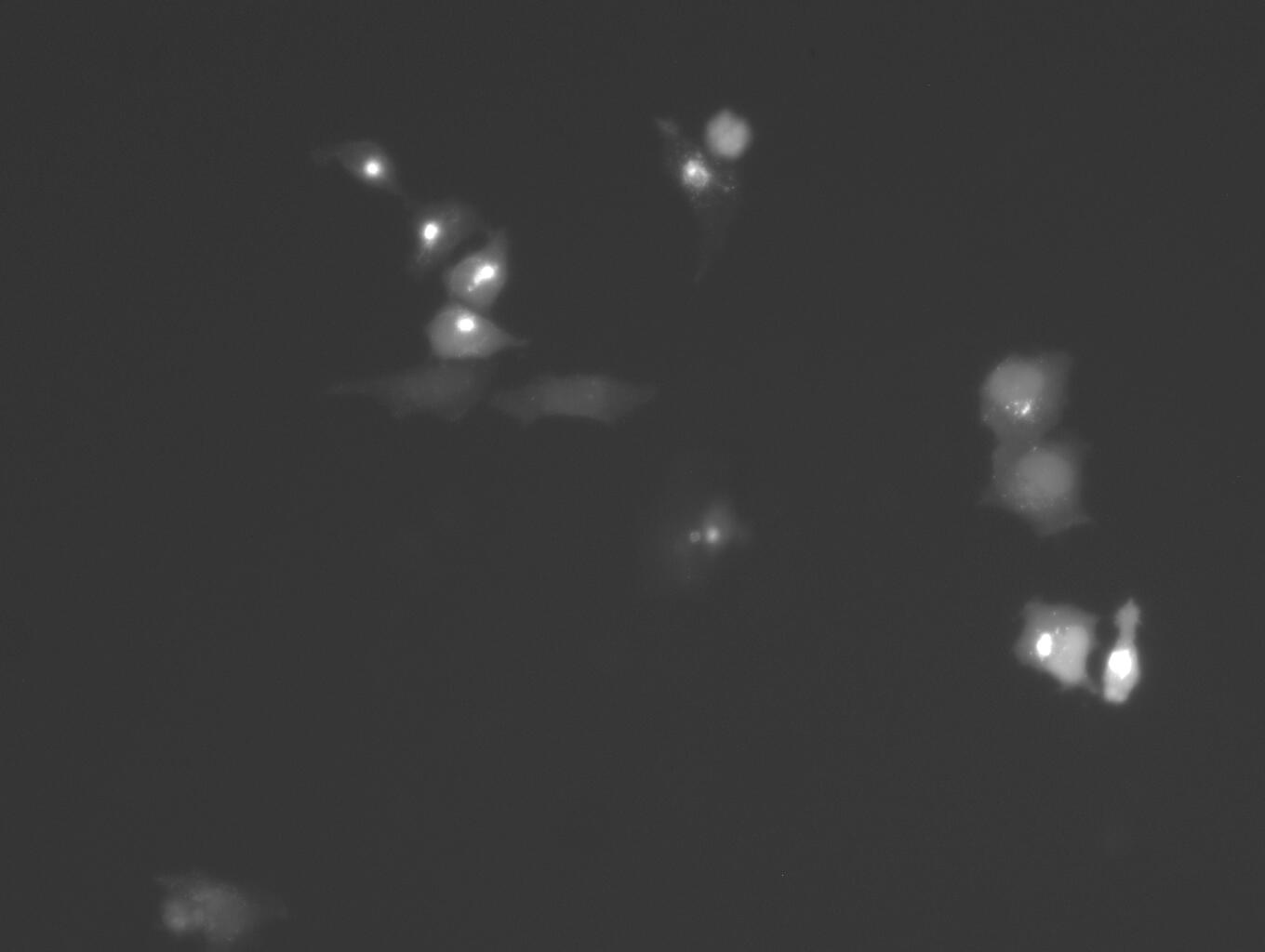

Supplement: Supplementary file 3 — Source data Fig. 1 [file 44318_2024_233_MOESM3_ESM.zip › 1G/Image/2. WT-5.jpg]

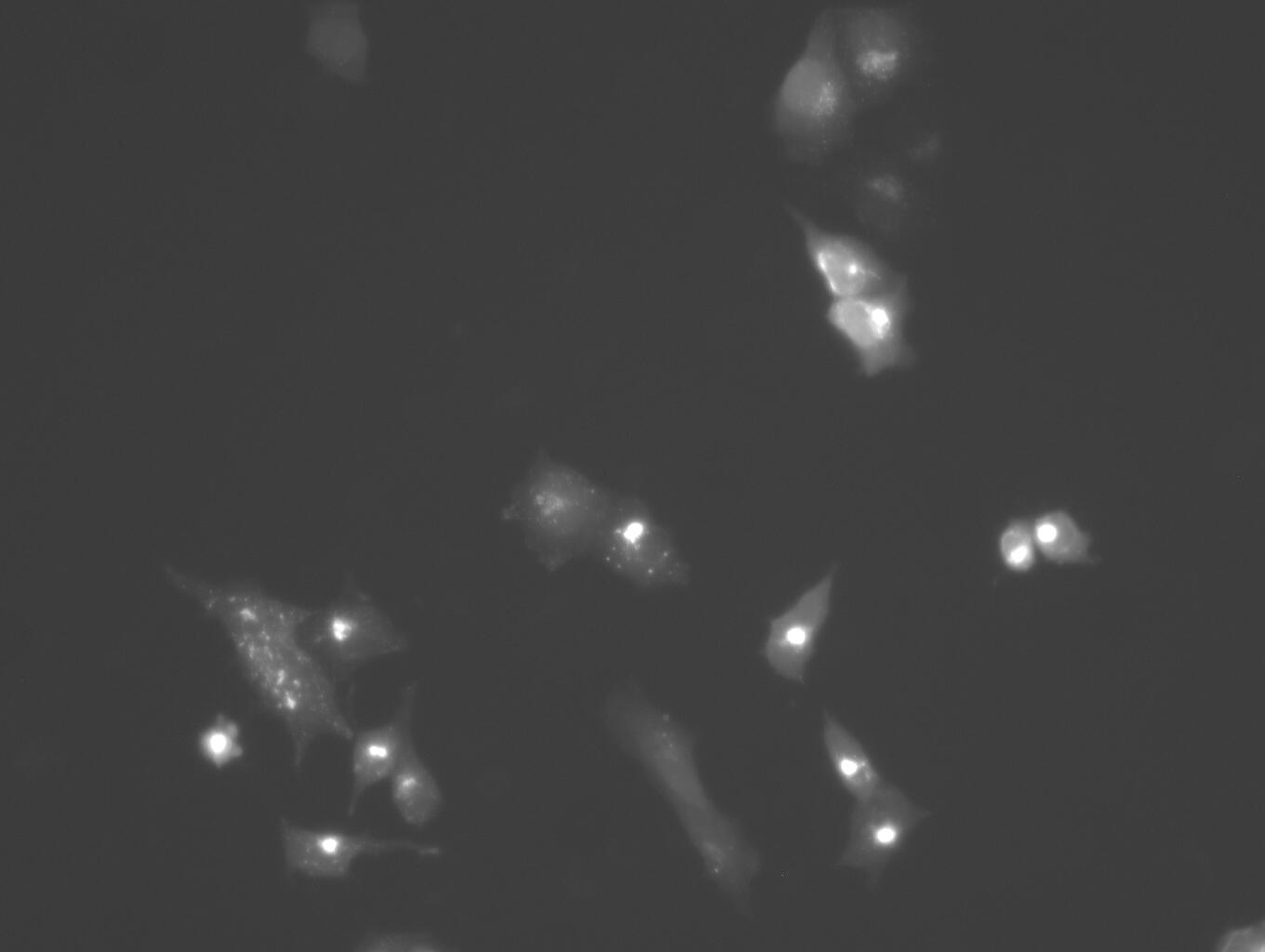

Supplement: Supplementary file 3 — Source data Fig. 1 [file 44318_2024_233_MOESM3_ESM.zip › 1G/Image/3. EGF1-5.jpg]

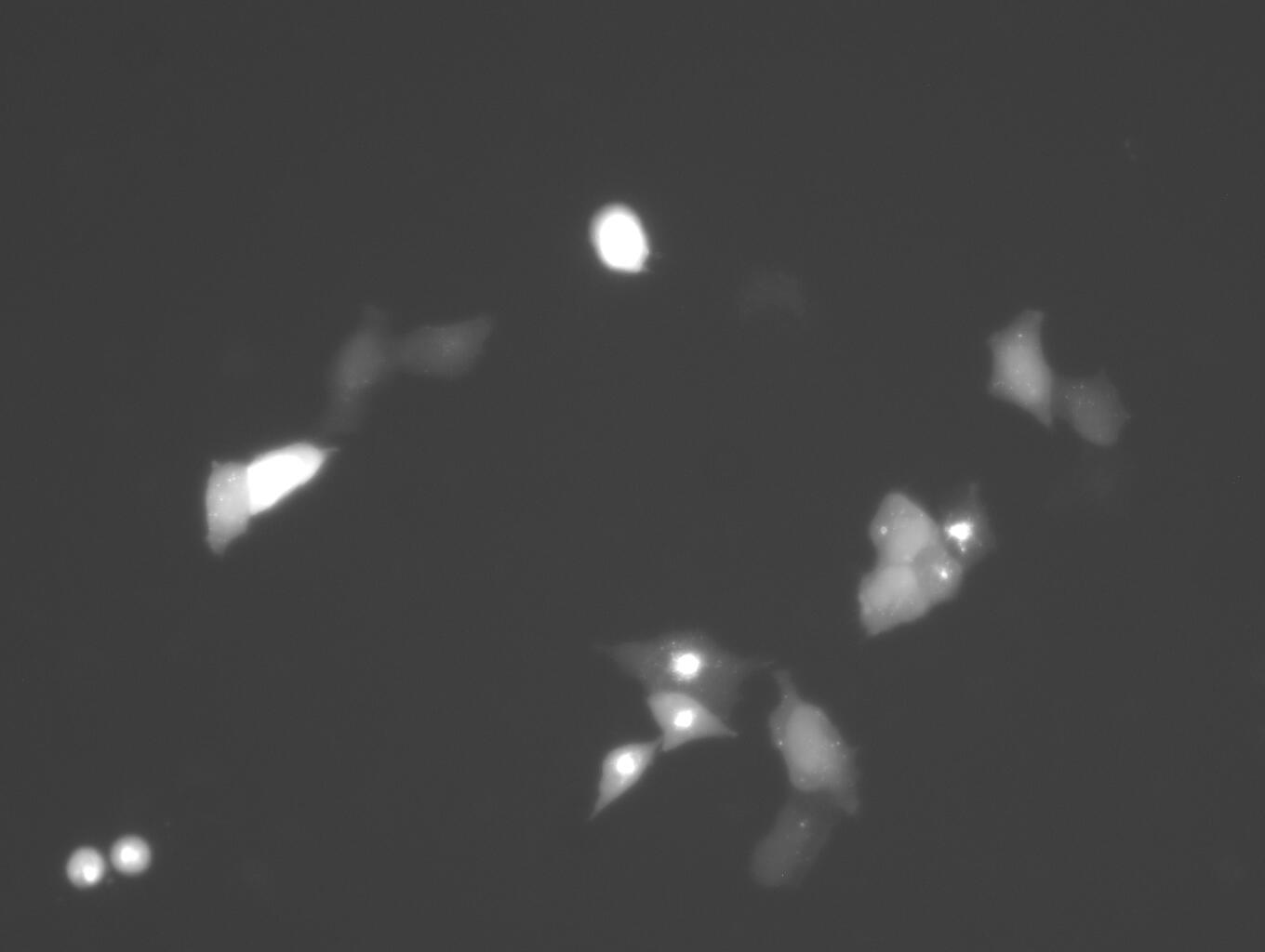

Supplement: Supplementary file 3 — Source data Fig. 1 [file 44318_2024_233_MOESM3_ESM.zip › 1G/Image/4. EGF2-5.jpg]

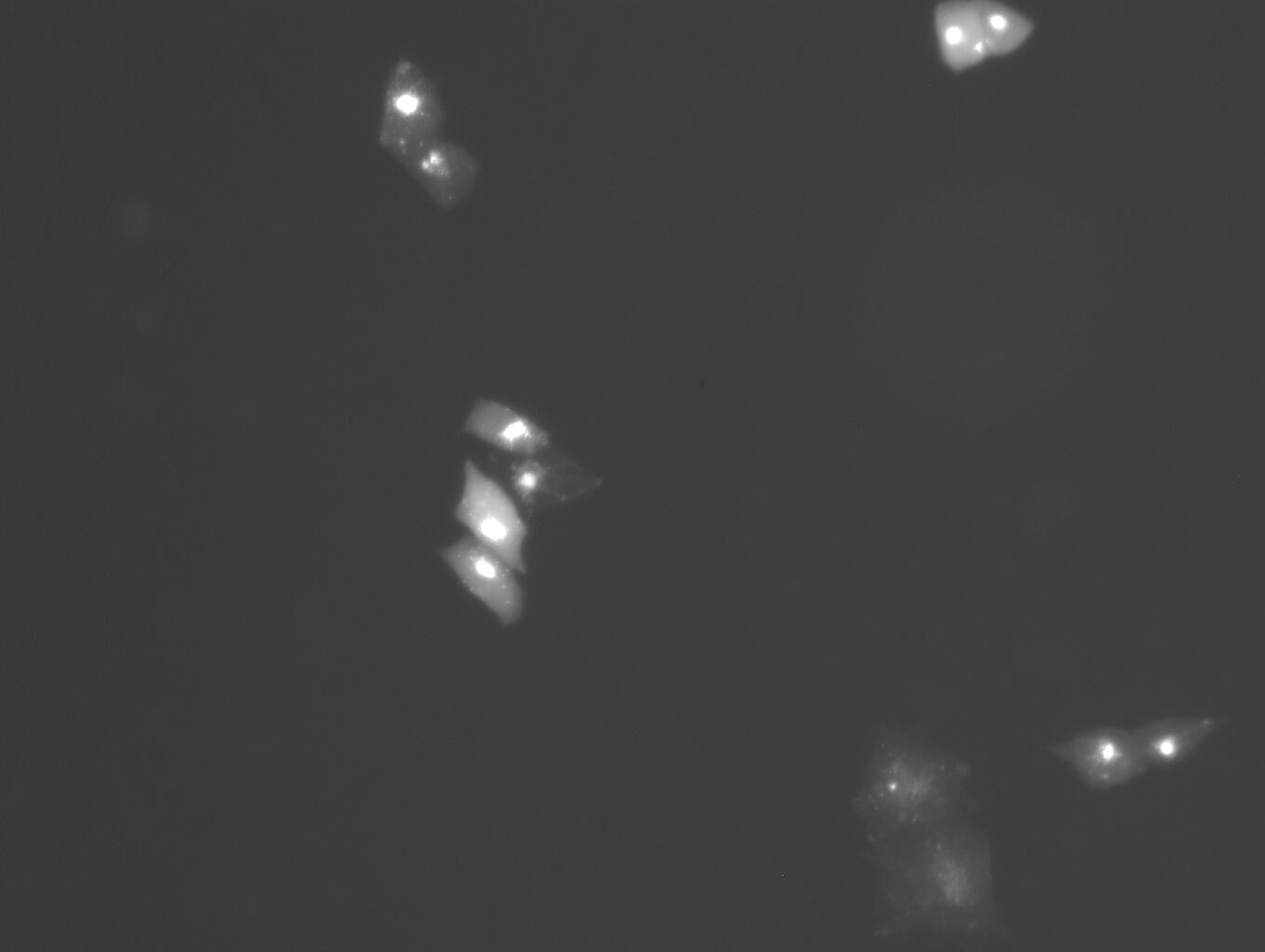

Supplement: Supplementary file 3 — Source data Fig. 1 [file 44318_2024_233_MOESM3_ESM.zip › 1G/Image/5. EGF3-1.jpg]

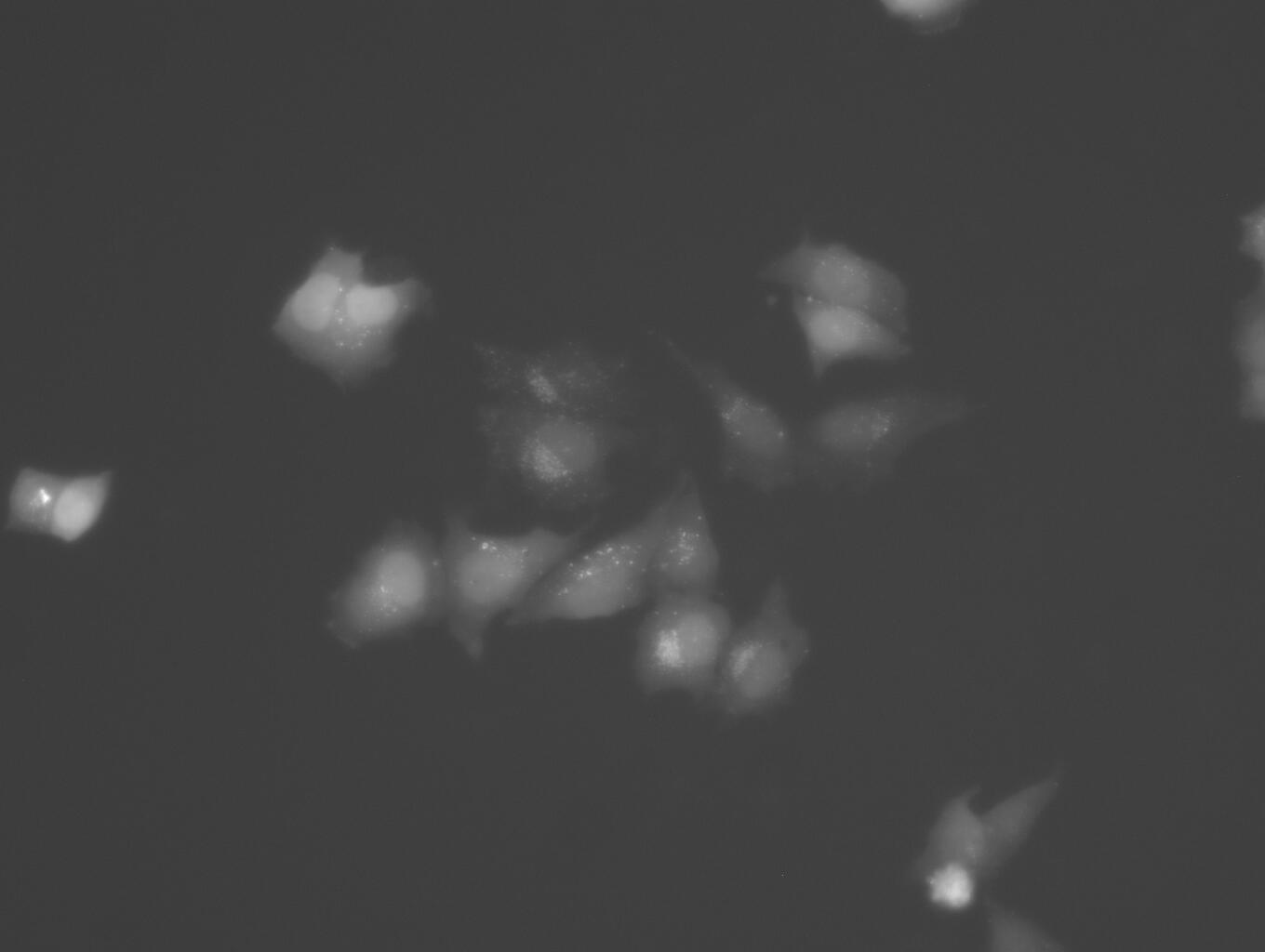

Supplement: Supplementary file 3 — Source data Fig. 1 [file 44318_2024_233_MOESM3_ESM.zip › 1G/Image/6. EGF4-1.jpg]

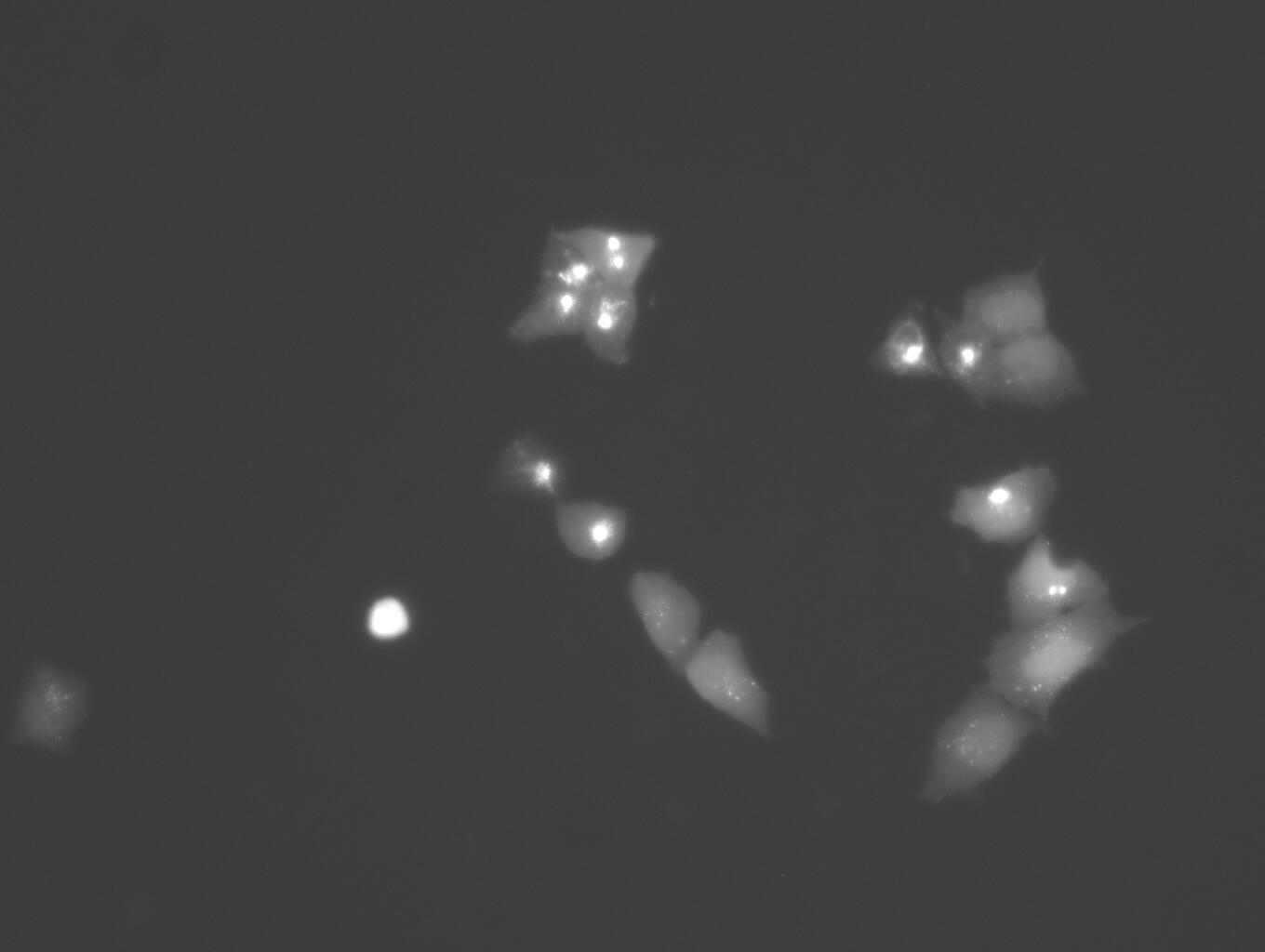

Supplement: Supplementary file 3 — Source data Fig. 1 [file 44318_2024_233_MOESM3_ESM.zip › 1G/Image/7. EGF5-3.jpg]

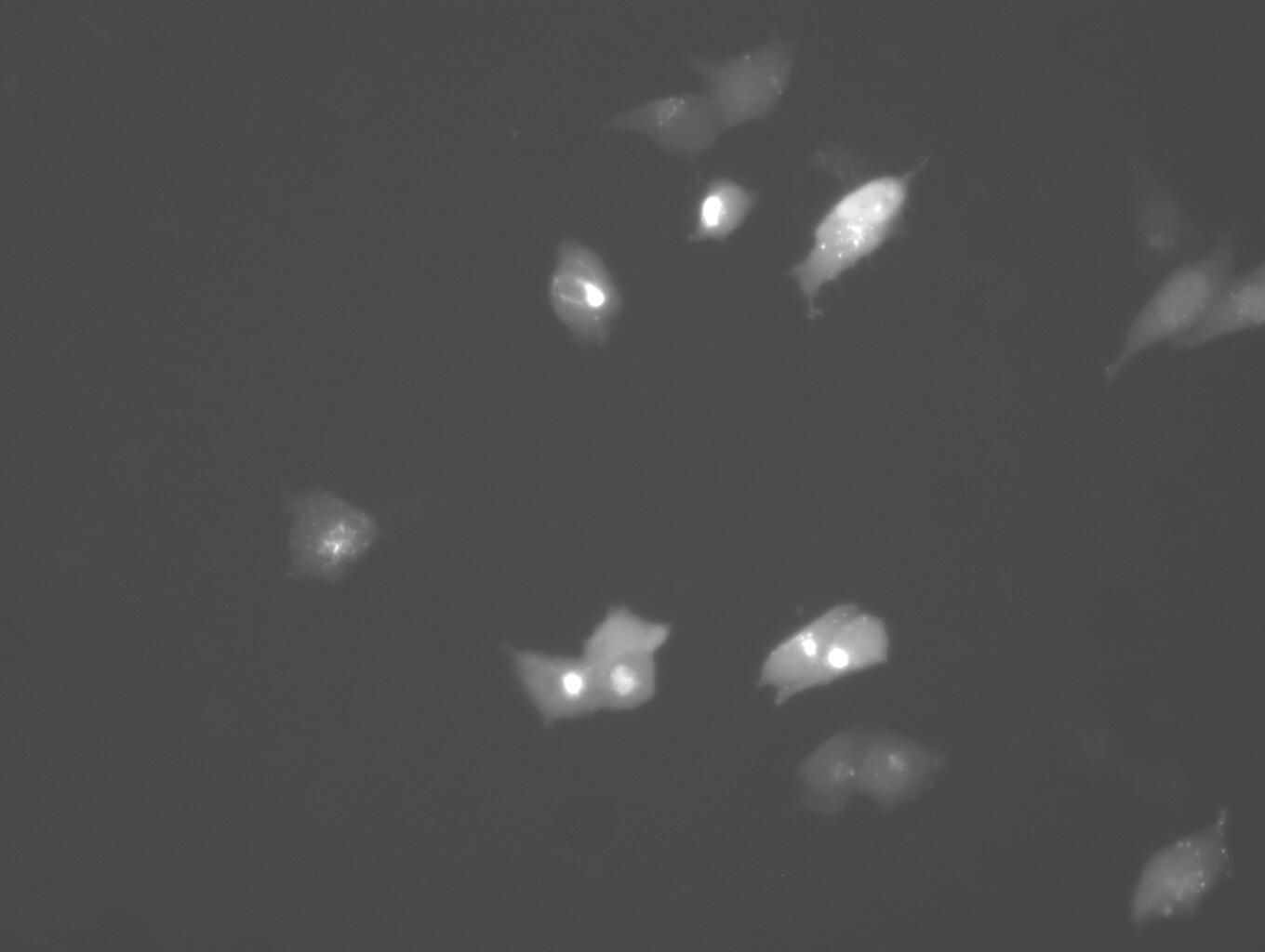

Supplement: Supplementary file 3 — Source data Fig. 1 [file 44318_2024_233_MOESM3_ESM.zip › 1G/Image/8. EGF6-2.jpg]

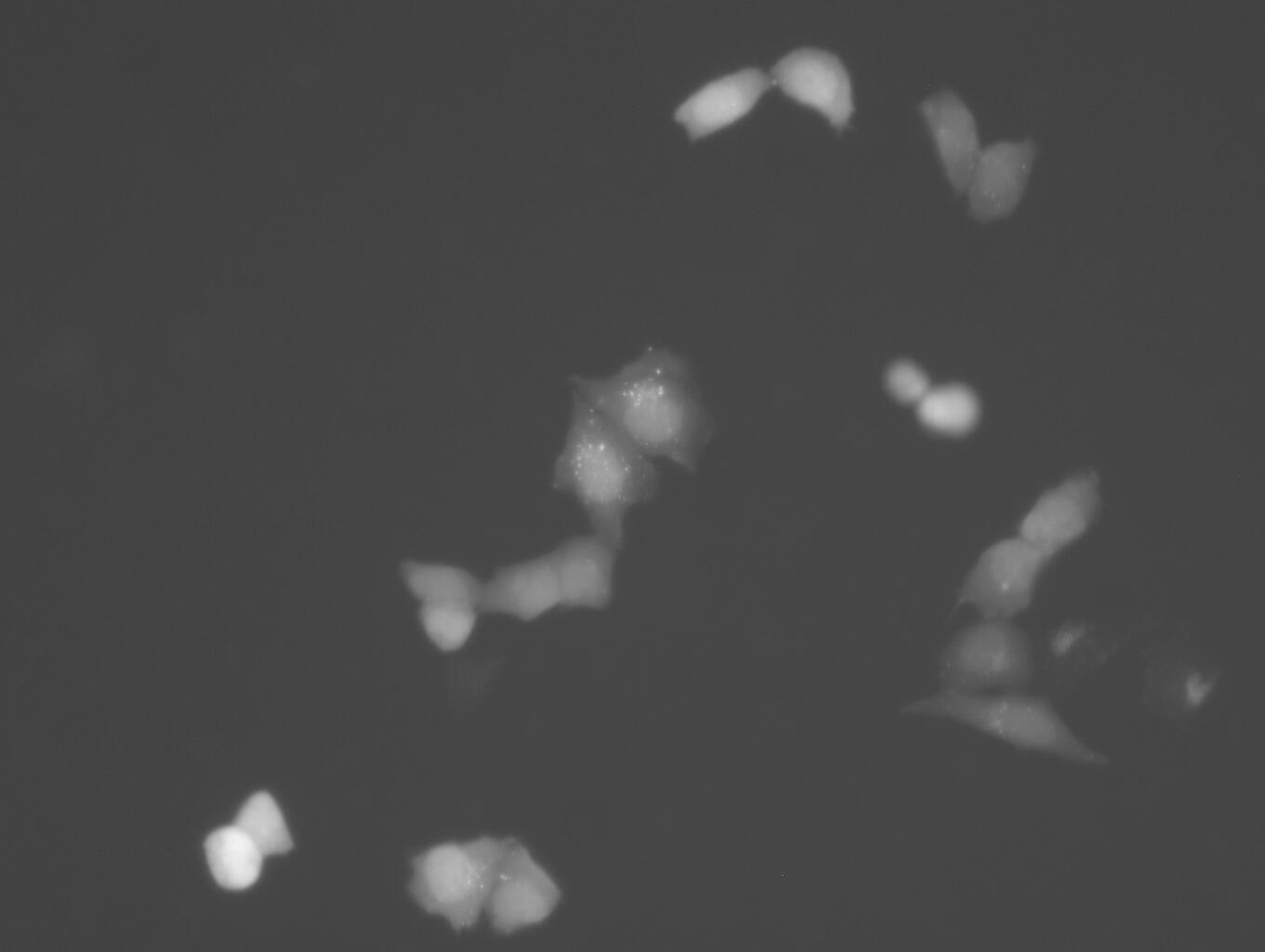

Supplement: Supplementary file 3 — Source data Fig. 1 [file 44318_2024_233_MOESM3_ESM.zip › 1G/Image/9. Isoform2-5.jpg]

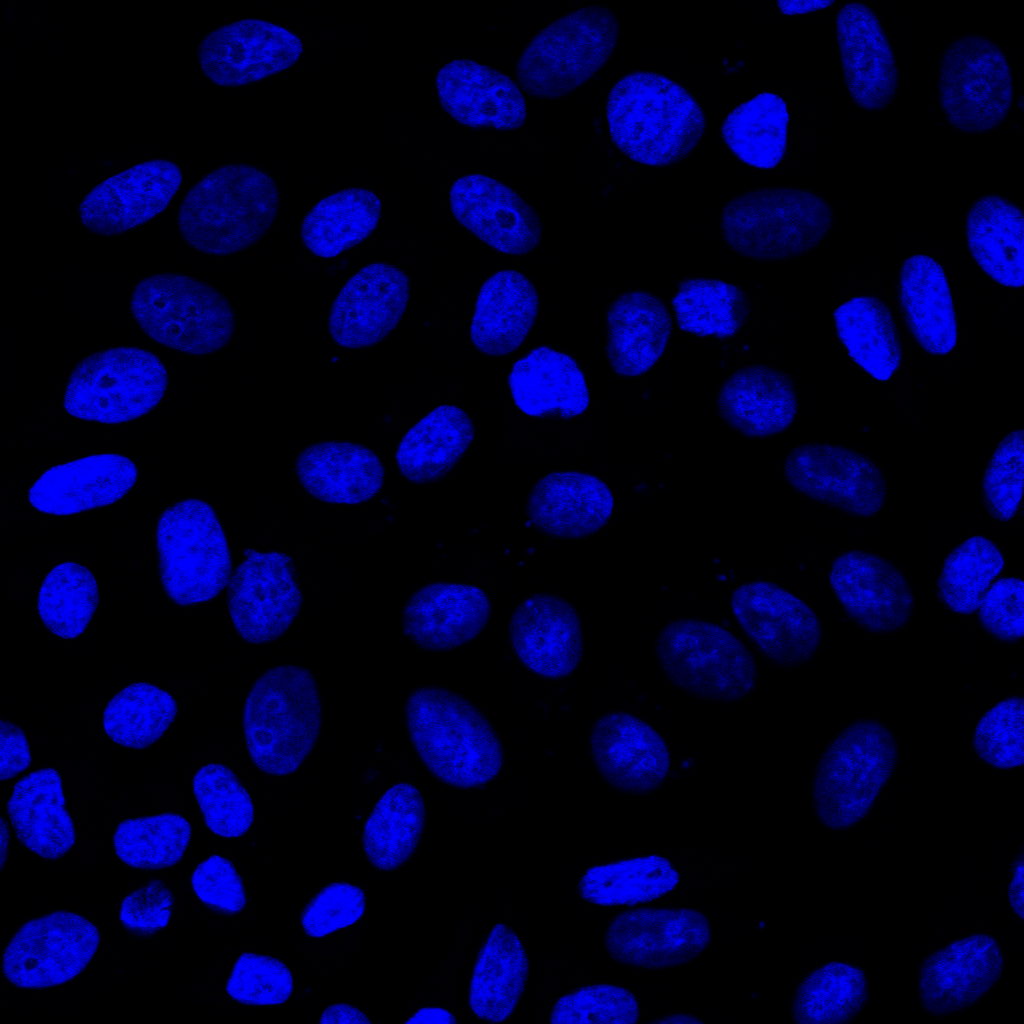

Supplement: Supplementary file 3 — Source data Fig. 1 [file 44318_2024_233_MOESM3_ESM.zip › 1H/HeLa Ctrl TGOLN2 GFP DLK1 594_Series003_ch00_SV.tif]

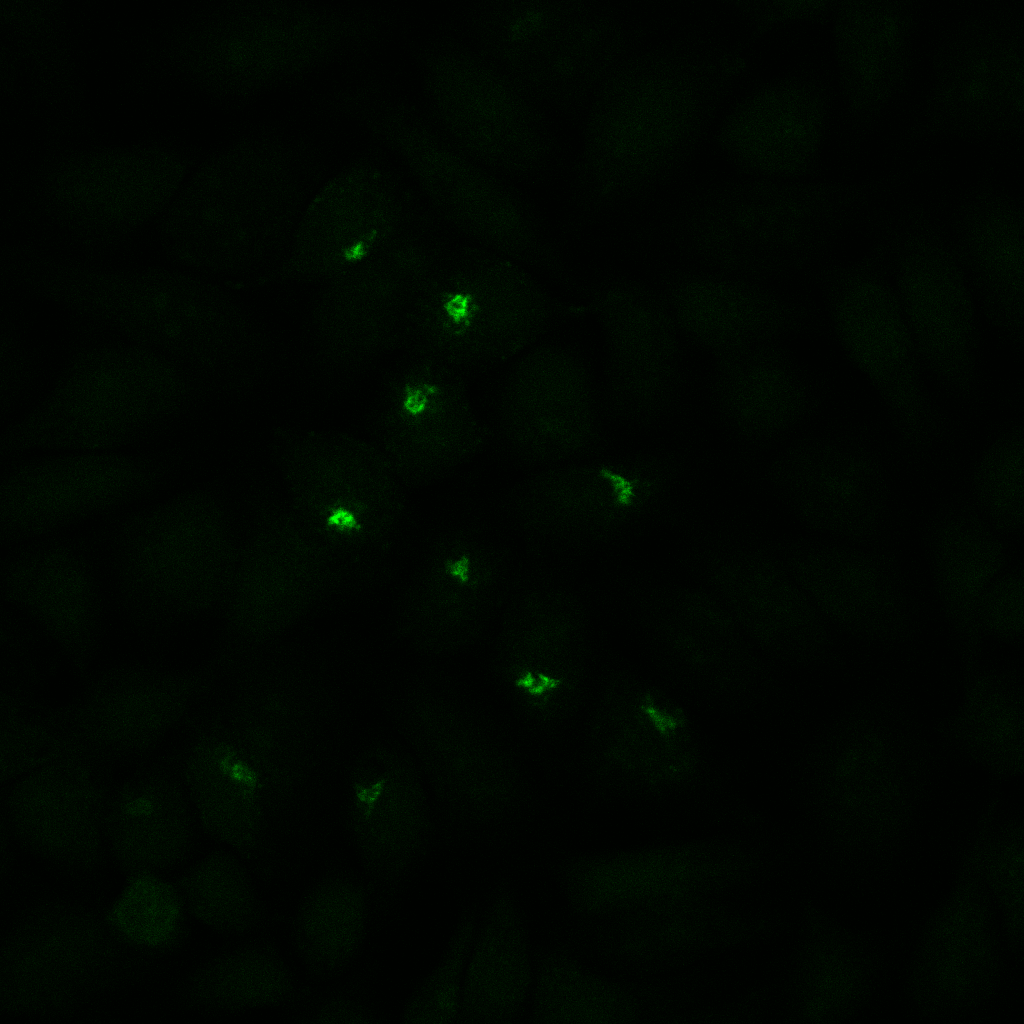

Supplement: Supplementary file 3 — Source data Fig. 1 [file 44318_2024_233_MOESM3_ESM.zip › 1H/HeLa Ctrl TGOLN2 GFP DLK1 594_Series003_ch01_SV.tif]

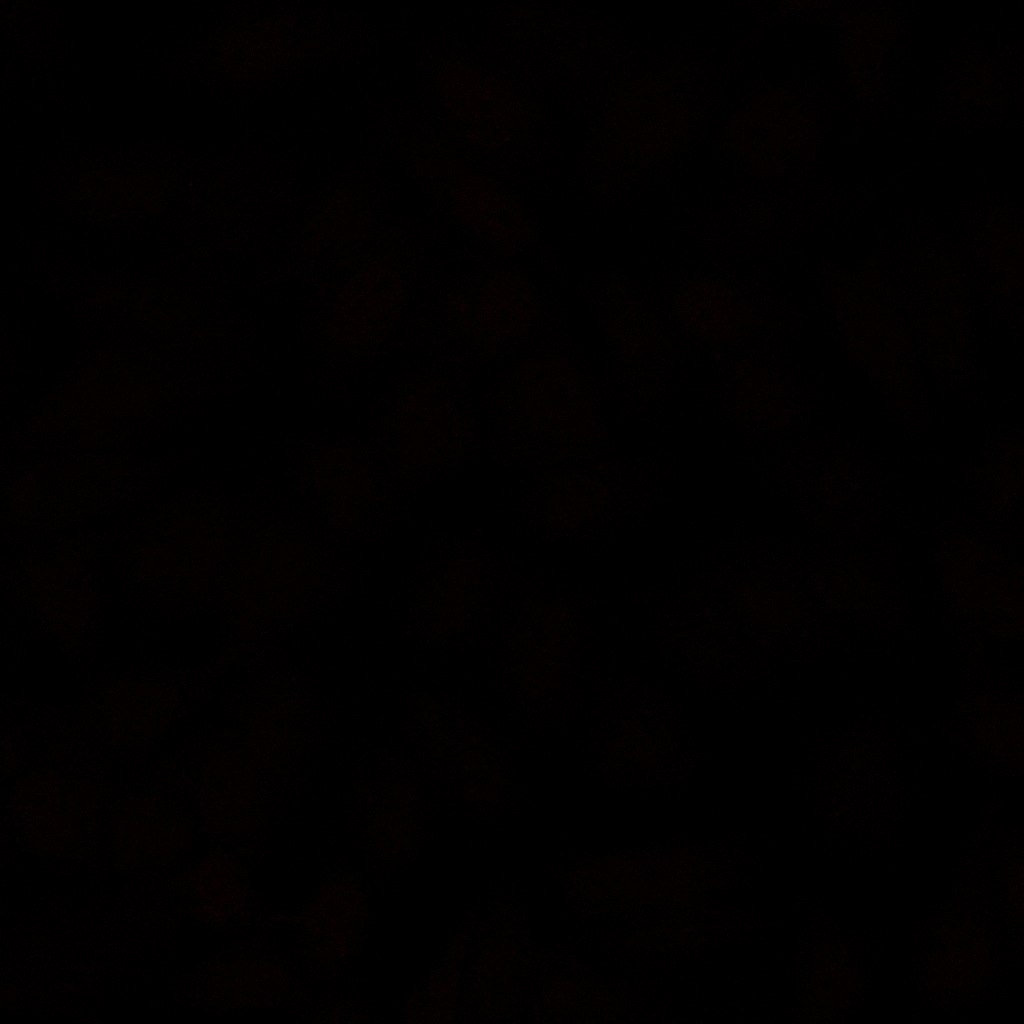

Supplement: Supplementary file 3 — Source data Fig. 1 [file 44318_2024_233_MOESM3_ESM.zip › 1H/HeLa Ctrl TGOLN2 GFP DLK1 594_Series003_ch02_SV.tif]

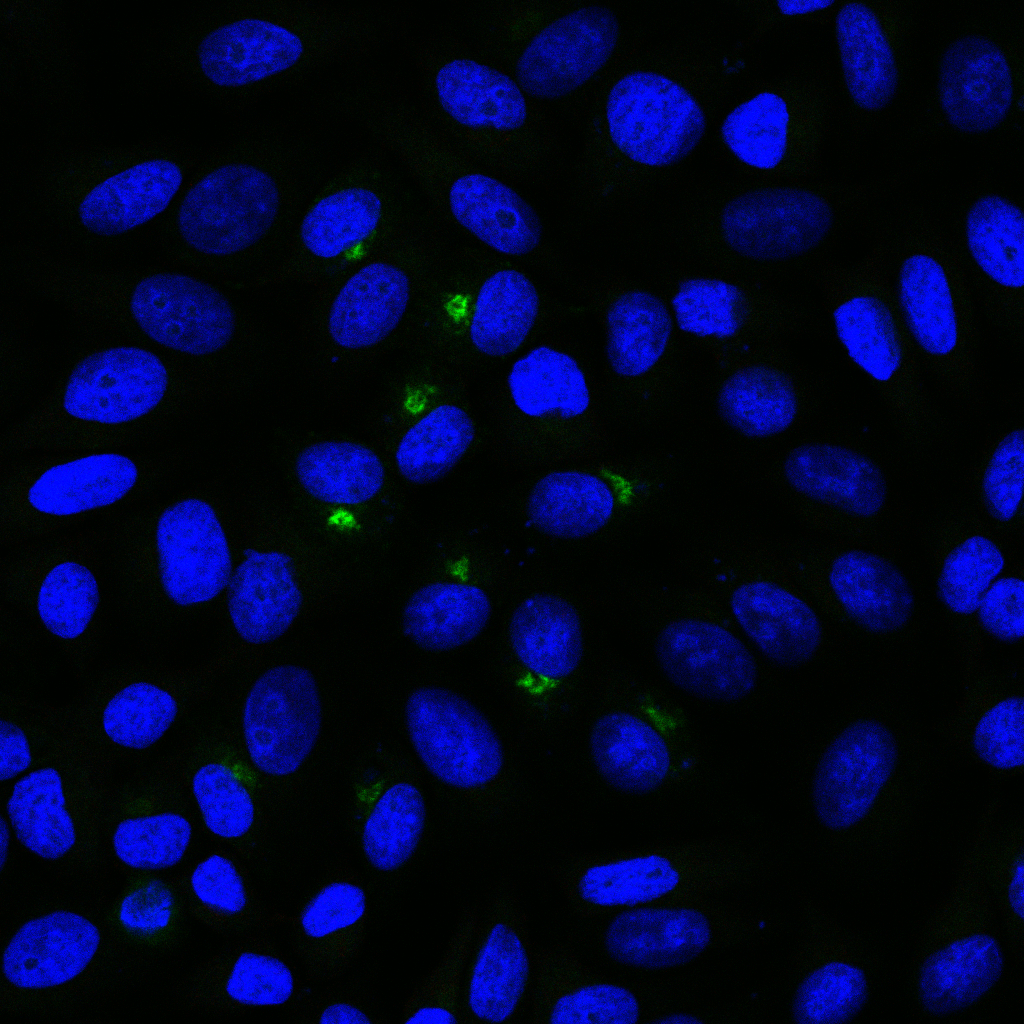

Supplement: Supplementary file 3 — Source data Fig. 1 [file 44318_2024_233_MOESM3_ESM.zip › 1H/HeLa Ctrl TGOLN2 GFP DLK1 594_Series003_overlay.tif]

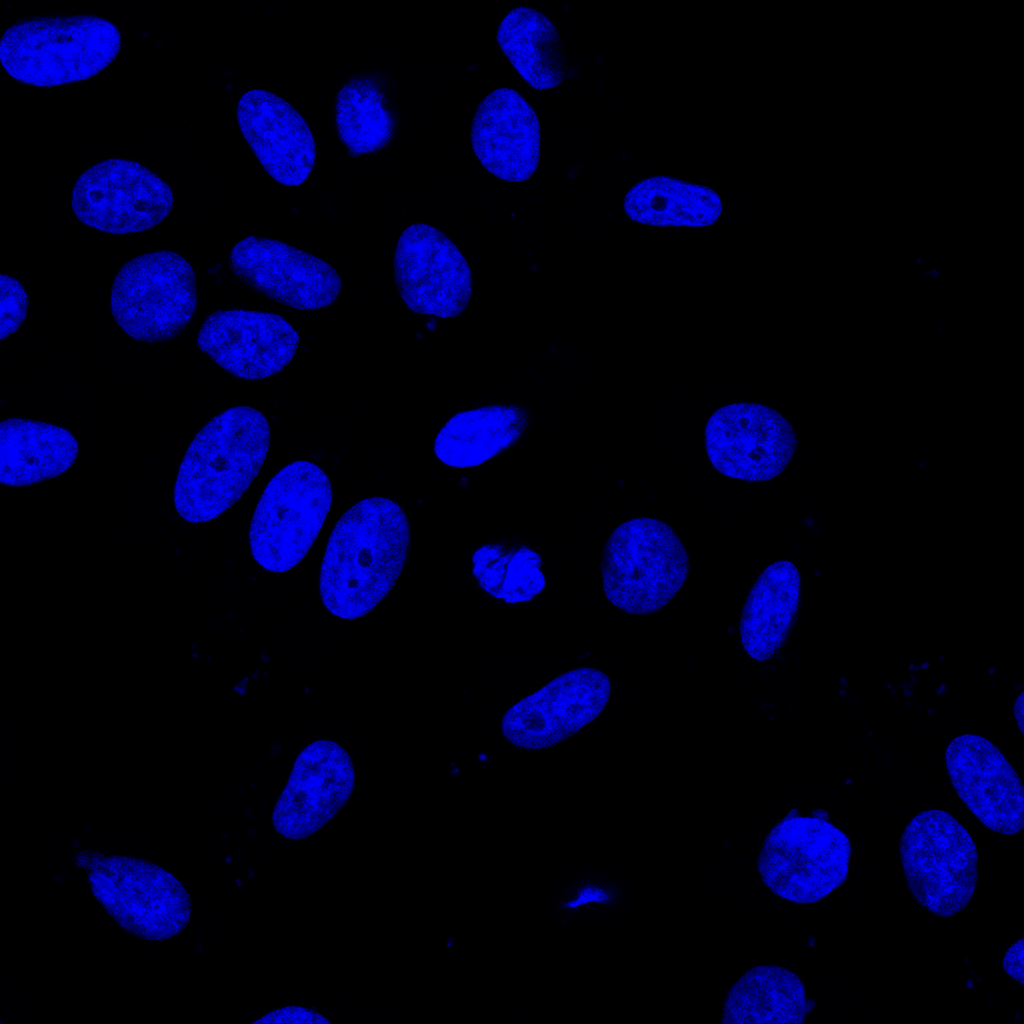

Supplement: Supplementary file 3 — Source data Fig. 1 [file 44318_2024_233_MOESM3_ESM.zip › 1H/HeLa DLK1 EGF4 TGOLN2 GFP DLK1 594_Series004_ch00_SV.tif]

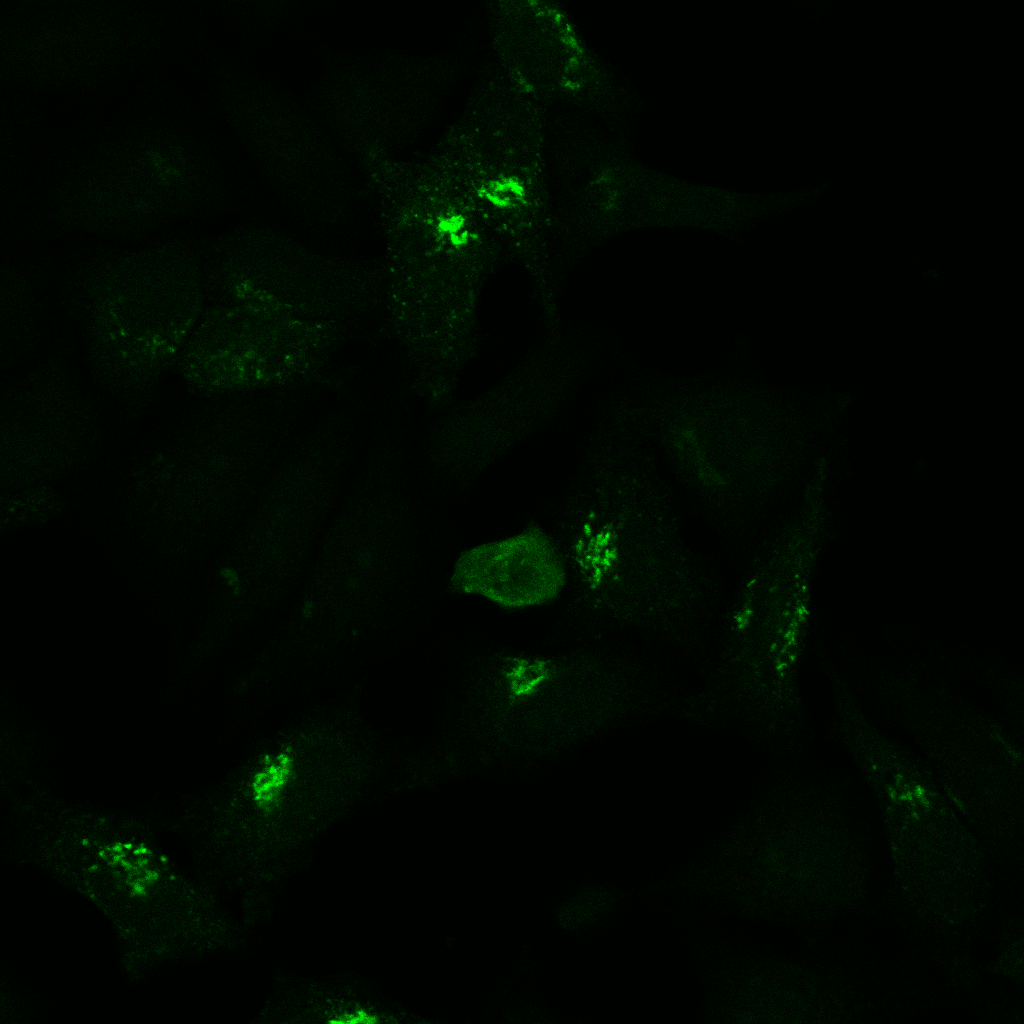

Supplement: Supplementary file 3 — Source data Fig. 1 [file 44318_2024_233_MOESM3_ESM.zip › 1H/HeLa DLK1 EGF4 TGOLN2 GFP DLK1 594_Series004_ch01_SV.tif]

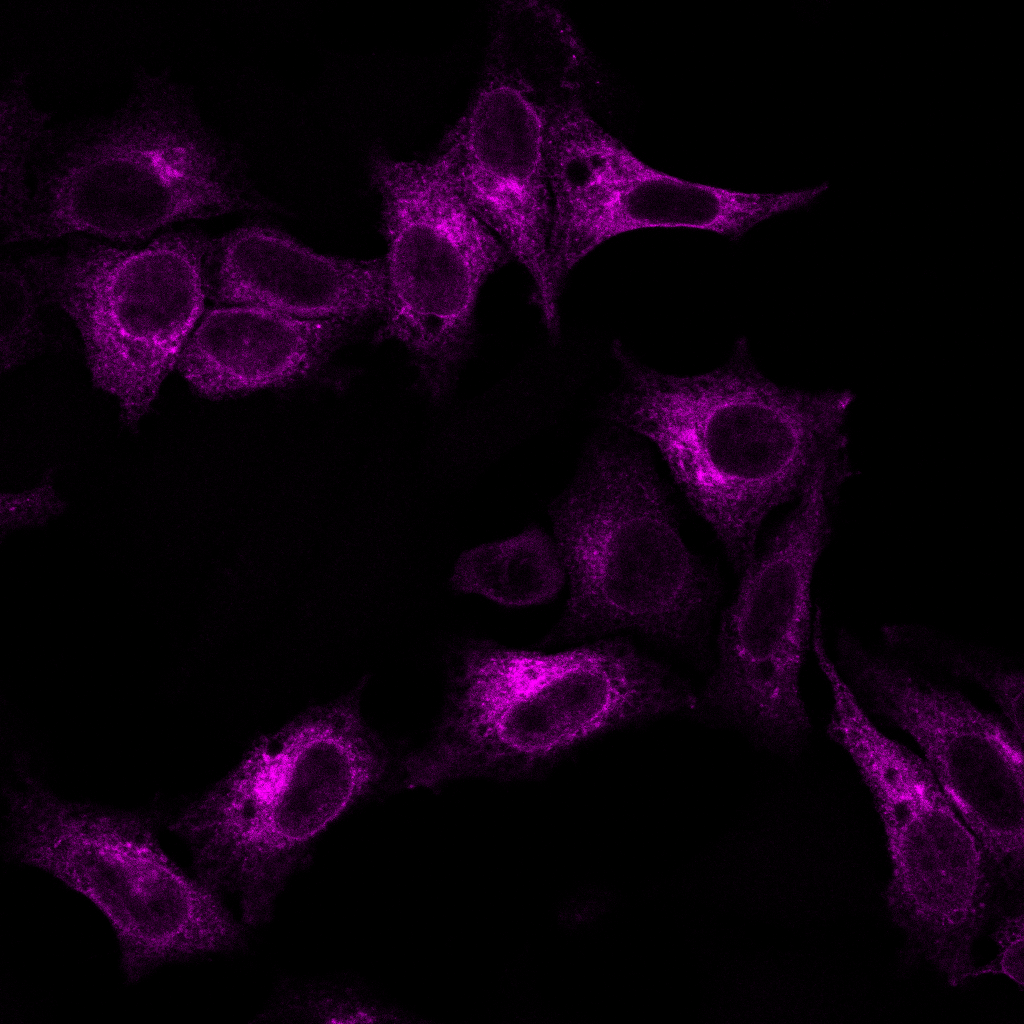

Supplement: Supplementary file 3 — Source data Fig. 1 [file 44318_2024_233_MOESM3_ESM.zip › 1H/HeLa DLK1 EGF4 TGOLN2 GFP DLK1 594_Series004_ch02_SV.tif]

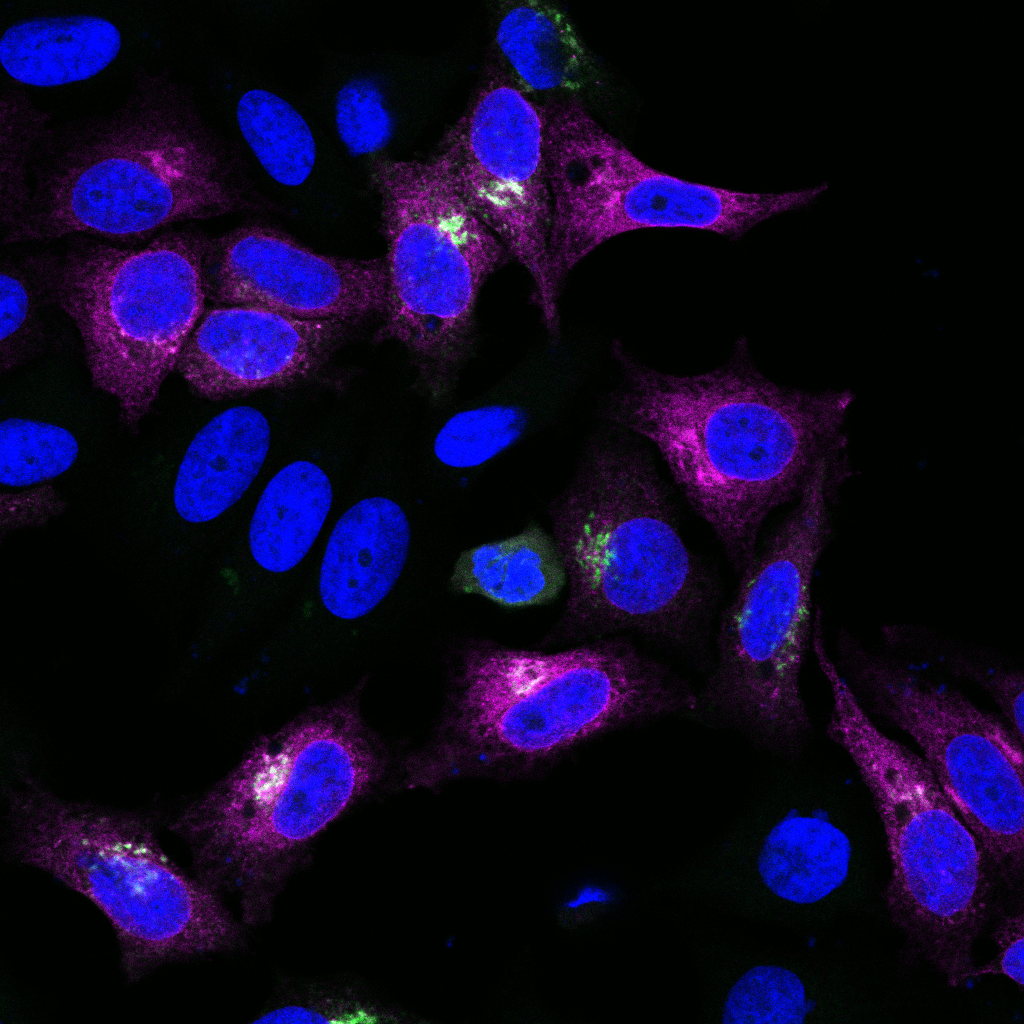

Supplement: Supplementary file 3 — Source data Fig. 1 [file 44318_2024_233_MOESM3_ESM.zip › 1H/HeLa DLK1 EGF4 TGOLN2 GFP DLK1 594_Series004_overlay.tif]

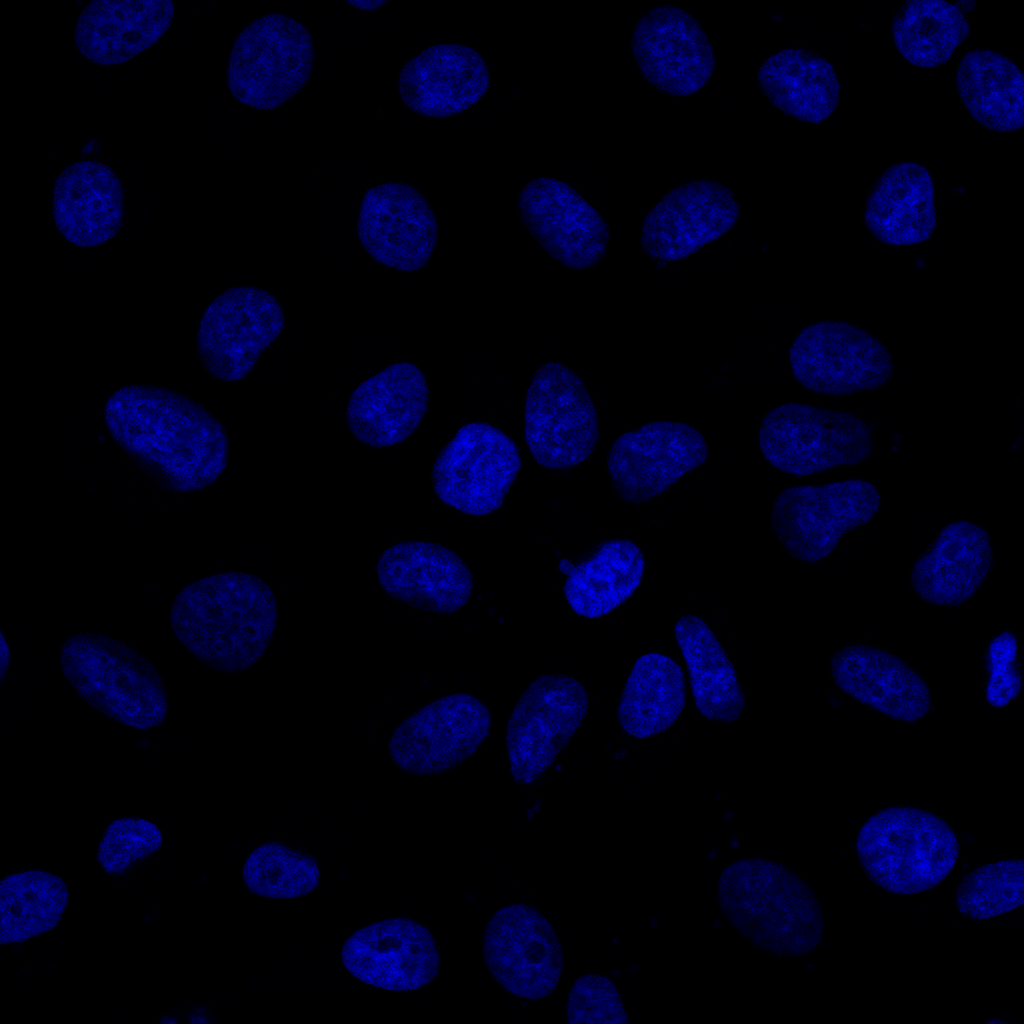

Supplement: Supplementary file 3 — Source data Fig. 1 [file 44318_2024_233_MOESM3_ESM.zip › 1H/HeLa DLK1 Isoform2 TGOLN2 GFP DLK1 594_Series001_ch00_SV.tif]

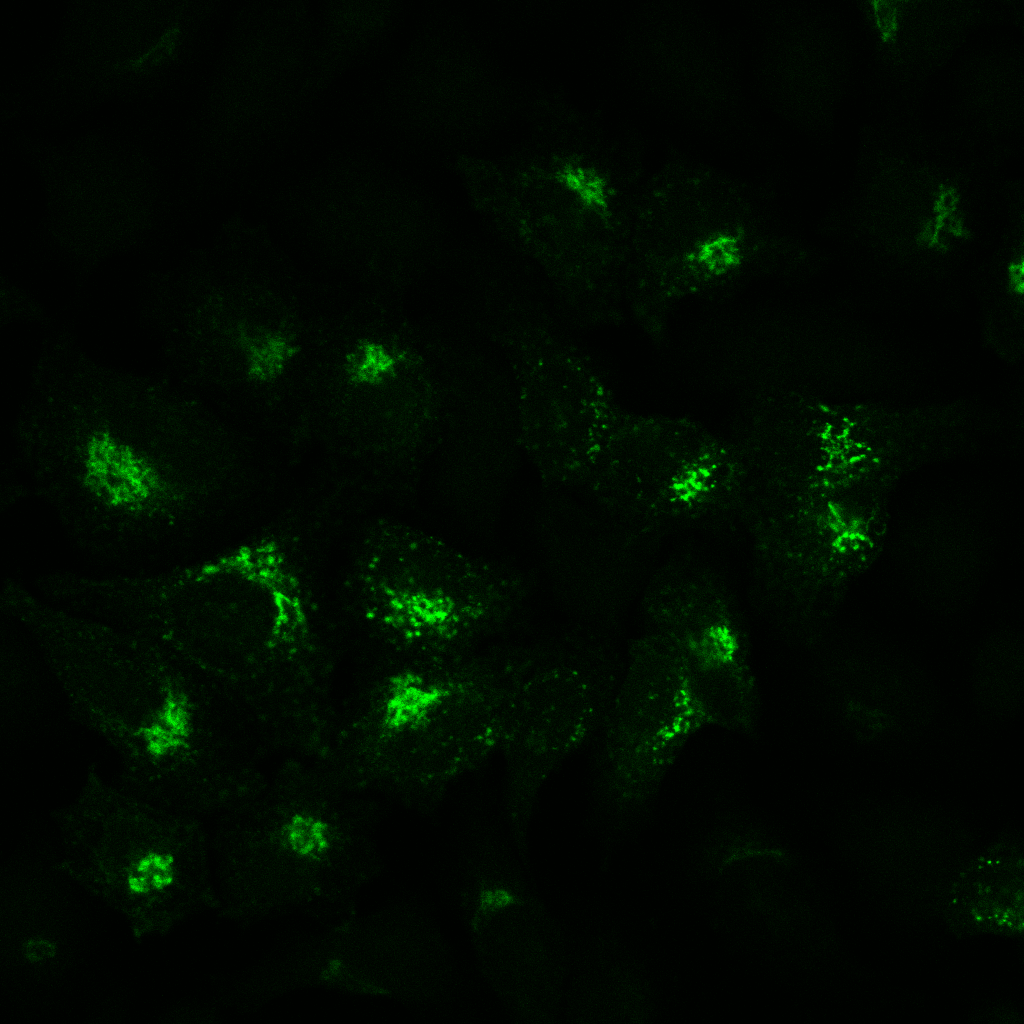

Supplement: Supplementary file 3 — Source data Fig. 1 [file 44318_2024_233_MOESM3_ESM.zip › 1H/HeLa DLK1 Isoform2 TGOLN2 GFP DLK1 594_Series001_ch01_SV.tif]

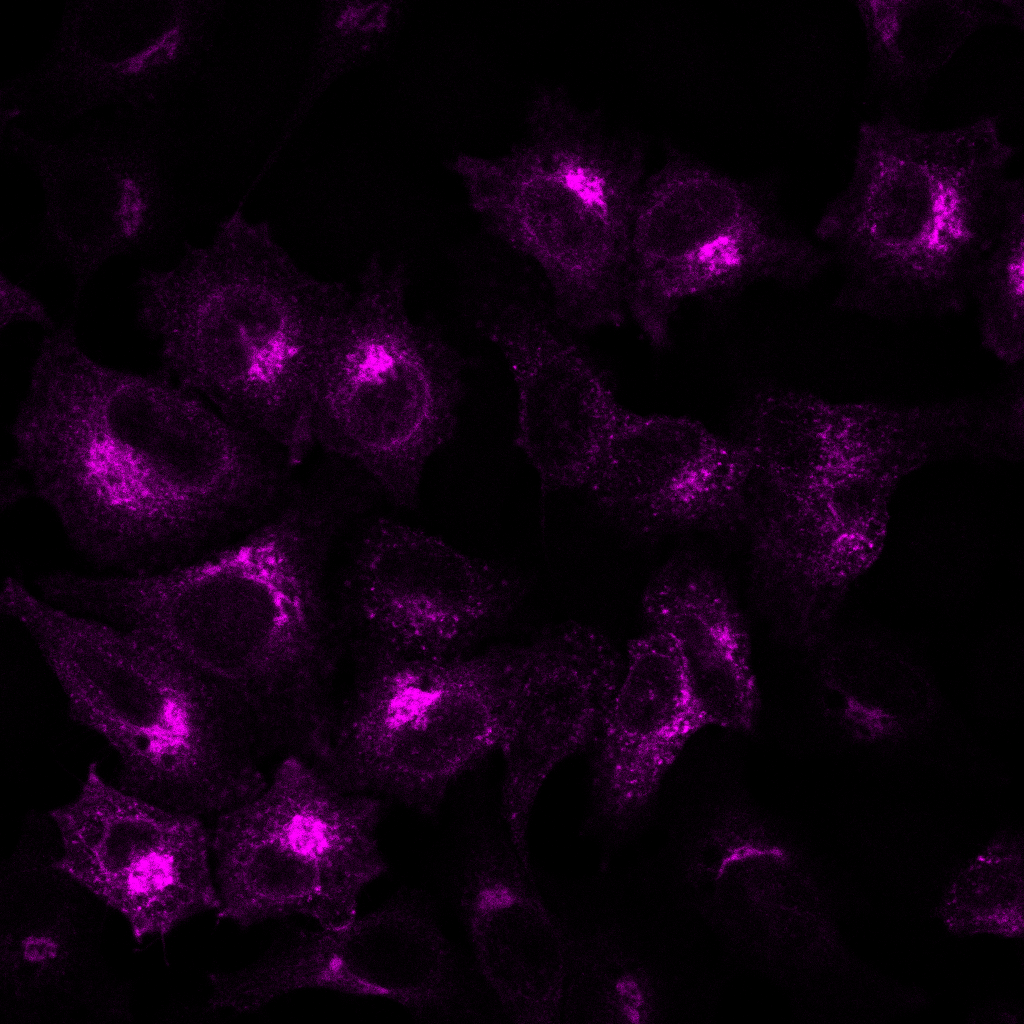

Supplement: Supplementary file 3 — Source data Fig. 1 [file 44318_2024_233_MOESM3_ESM.zip › 1H/HeLa DLK1 Isoform2 TGOLN2 GFP DLK1 594_Series001_ch02_SV.tif]

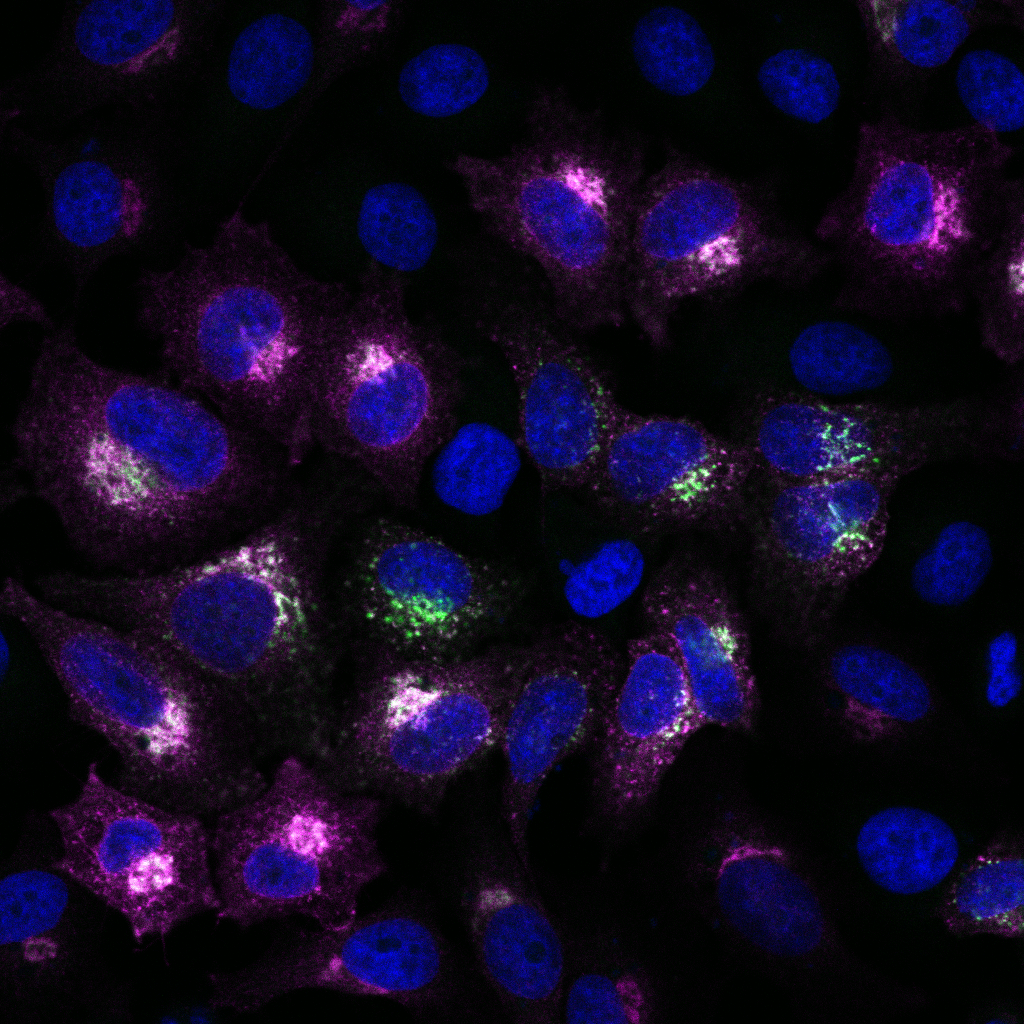

Supplement: Supplementary file 3 — Source data Fig. 1 [file 44318_2024_233_MOESM3_ESM.zip › 1H/HeLa DLK1 Isoform2 TGOLN2 GFP DLK1 594_Series001_overlay.tif]

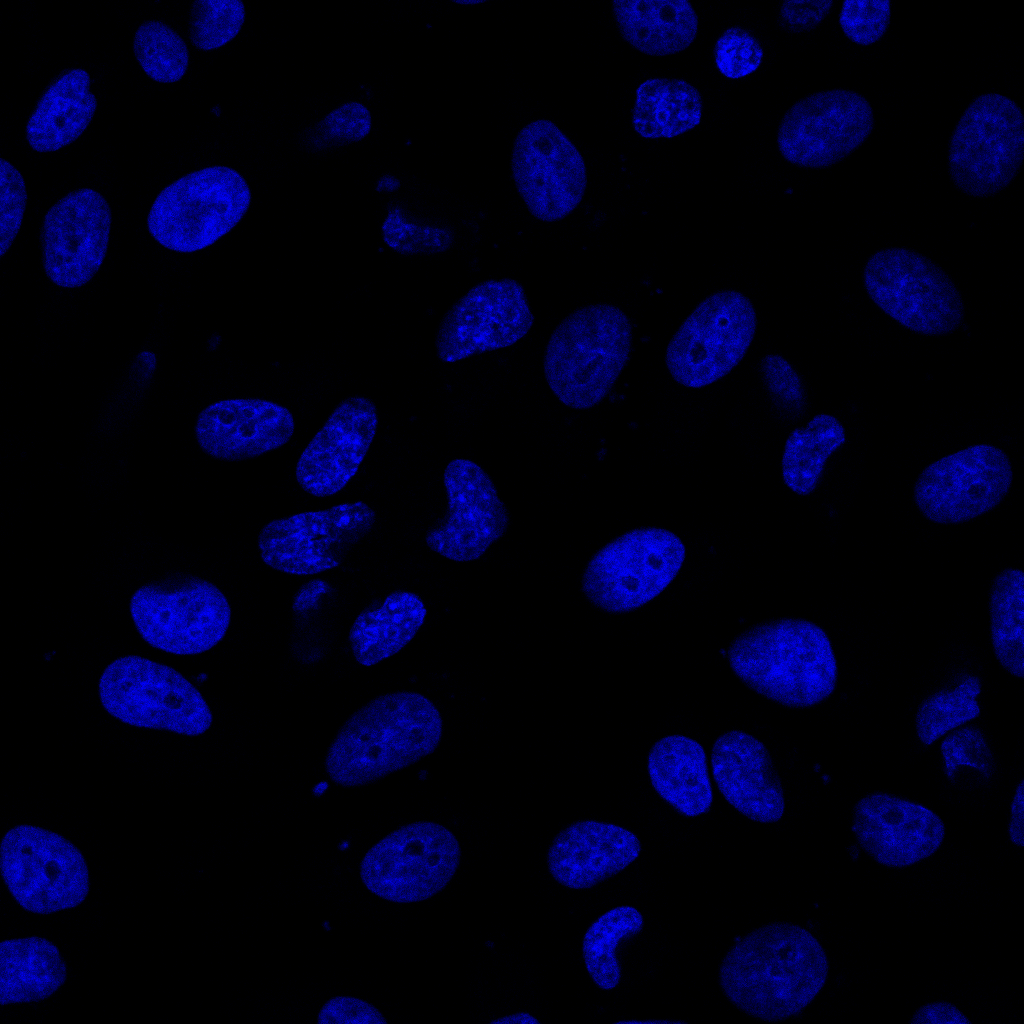

Supplement: Supplementary file 3 — Source data Fig. 1 [file 44318_2024_233_MOESM3_ESM.zip › 1H/HeLa DLK1 WT TGOLN2 GFP DLK1 594_Series002_ch00_SV.tif]

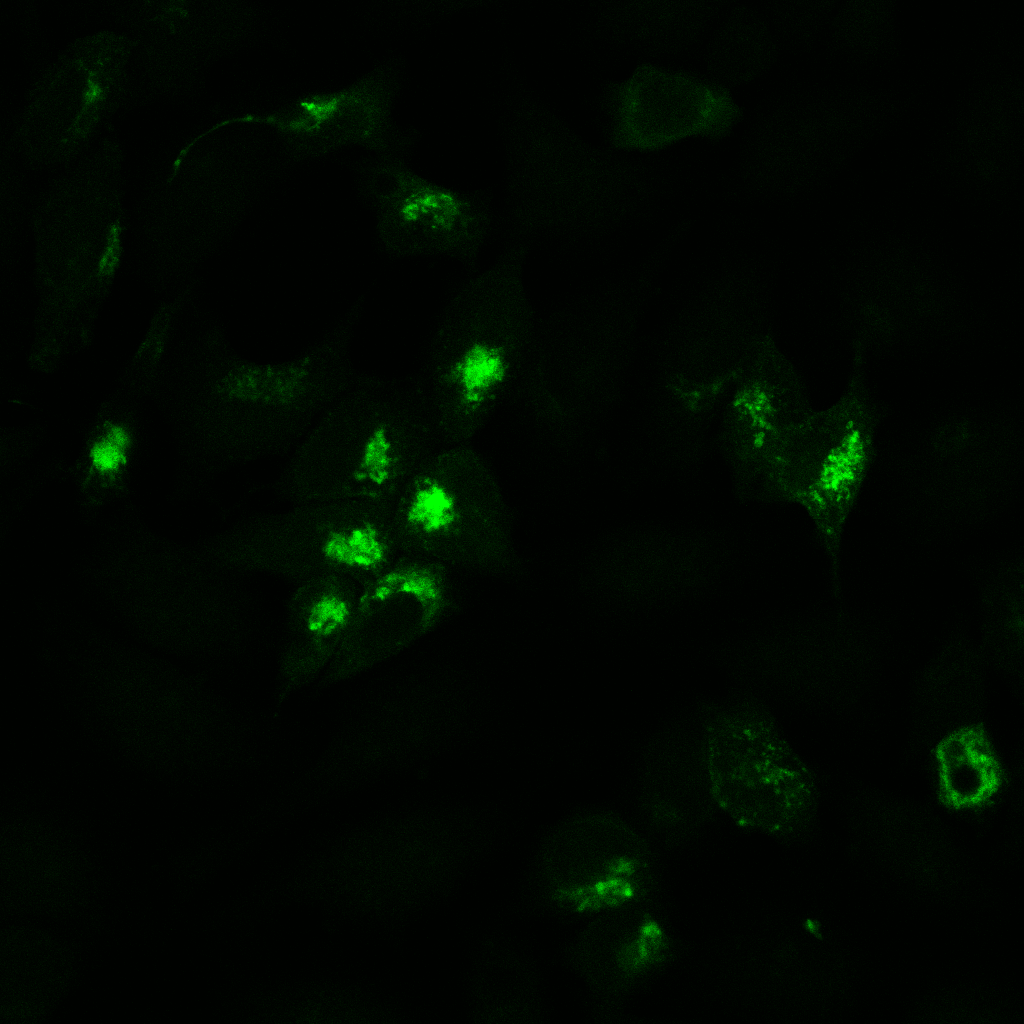

Supplement: Supplementary file 3 — Source data Fig. 1 [file 44318_2024_233_MOESM3_ESM.zip › 1H/HeLa DLK1 WT TGOLN2 GFP DLK1 594_Series002_ch01_SV.tif]

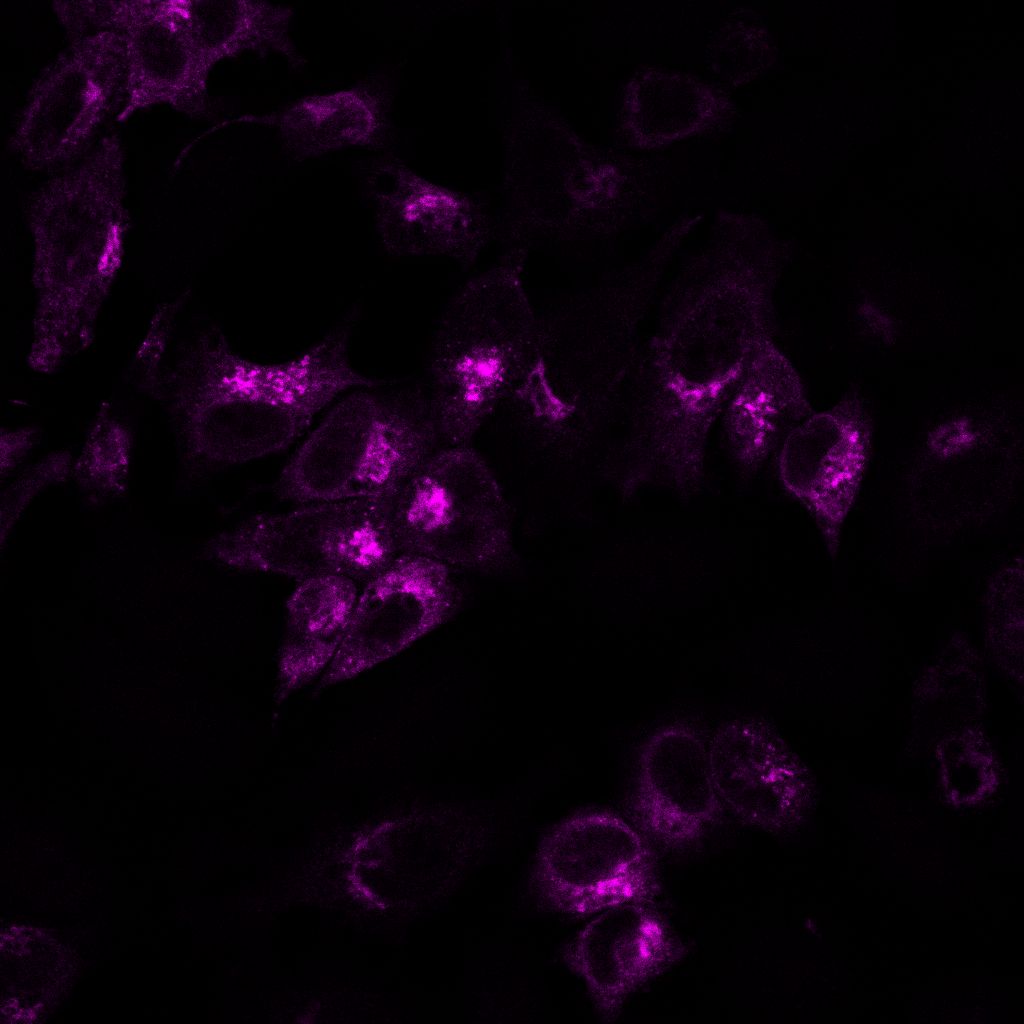

Supplement: Supplementary file 3 — Source data Fig. 1 [file 44318_2024_233_MOESM3_ESM.zip › 1H/HeLa DLK1 WT TGOLN2 GFP DLK1 594_Series002_ch02_SV.tif]

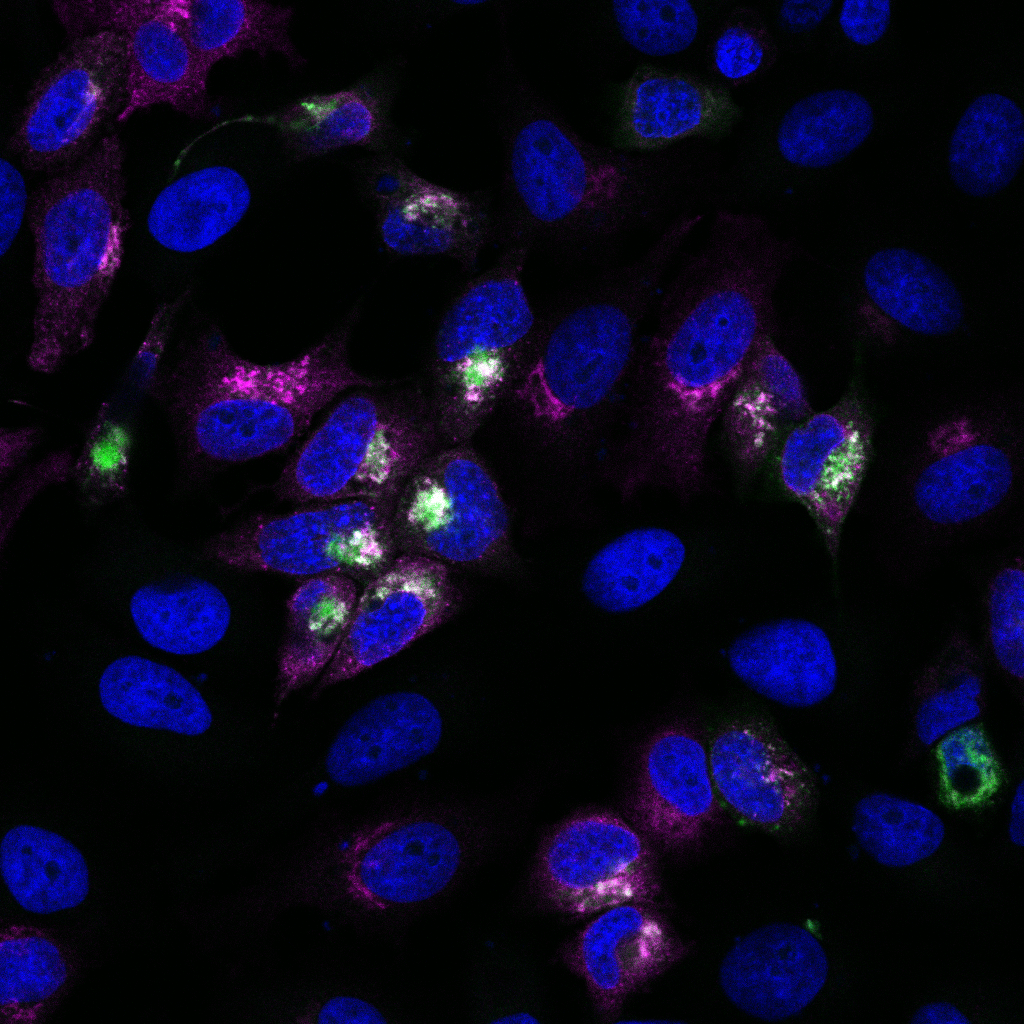

Supplement: Supplementary file 3 — Source data Fig. 1 [file 44318_2024_233_MOESM3_ESM.zip › 1H/HeLa DLK1 WT TGOLN2 GFP DLK1 594_Series002_overlay.tif]

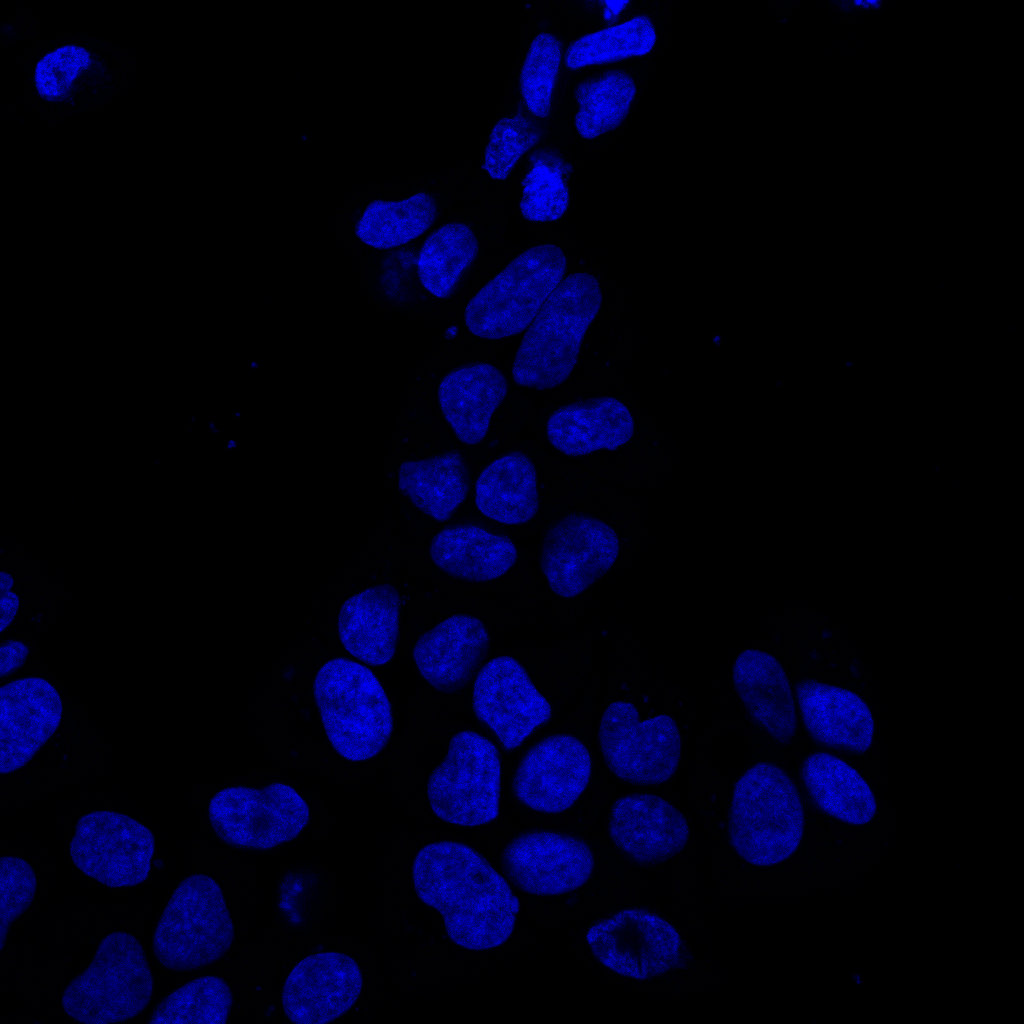

Supplement: Supplementary file 4 — Source data Fig. 2 [file 44318_2024_233_MOESM4_ESM.zip › 2A/HeLa sgATG16L1 Ctrl_Series001_ch00_SV.tif]

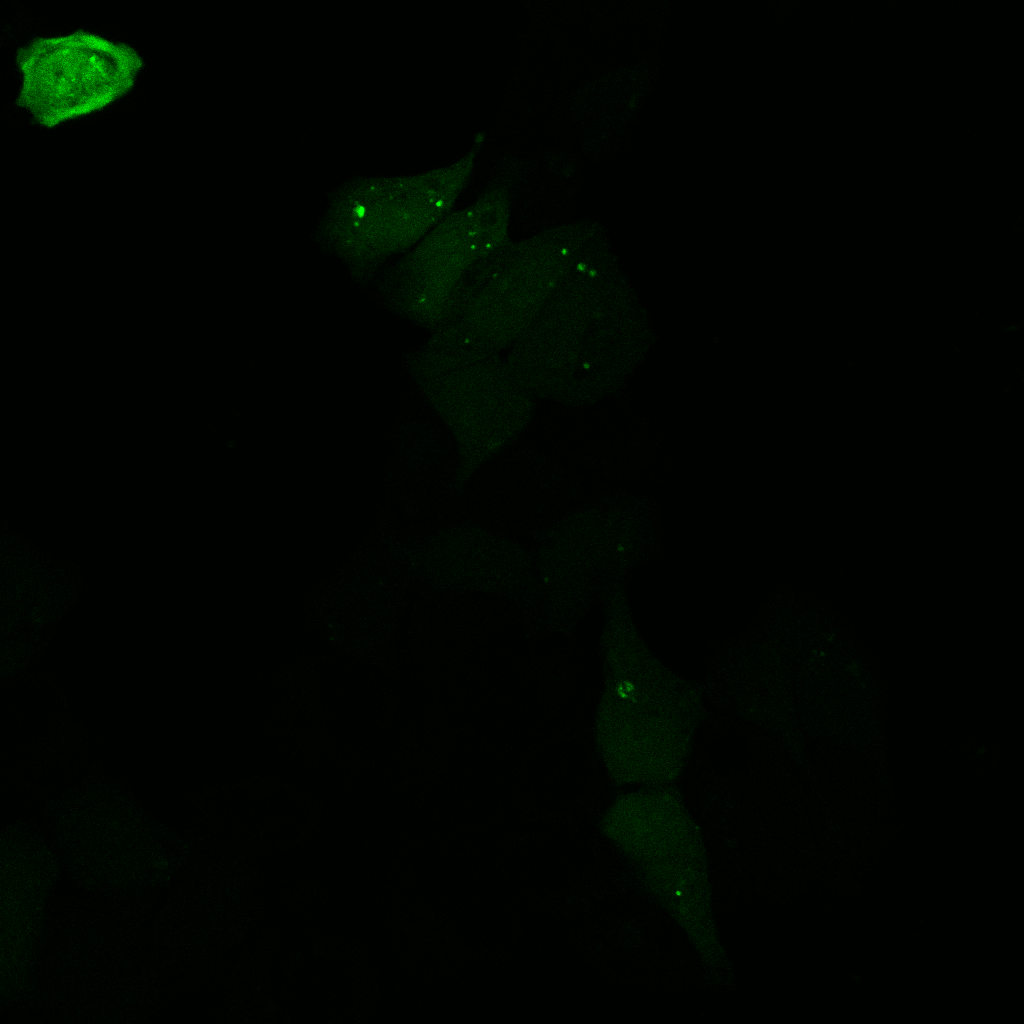

Supplement: Supplementary file 4 — Source data Fig. 2 [file 44318_2024_233_MOESM4_ESM.zip › 2A/HeLa sgATG16L1 Ctrl_Series001_ch01_SV.tif]

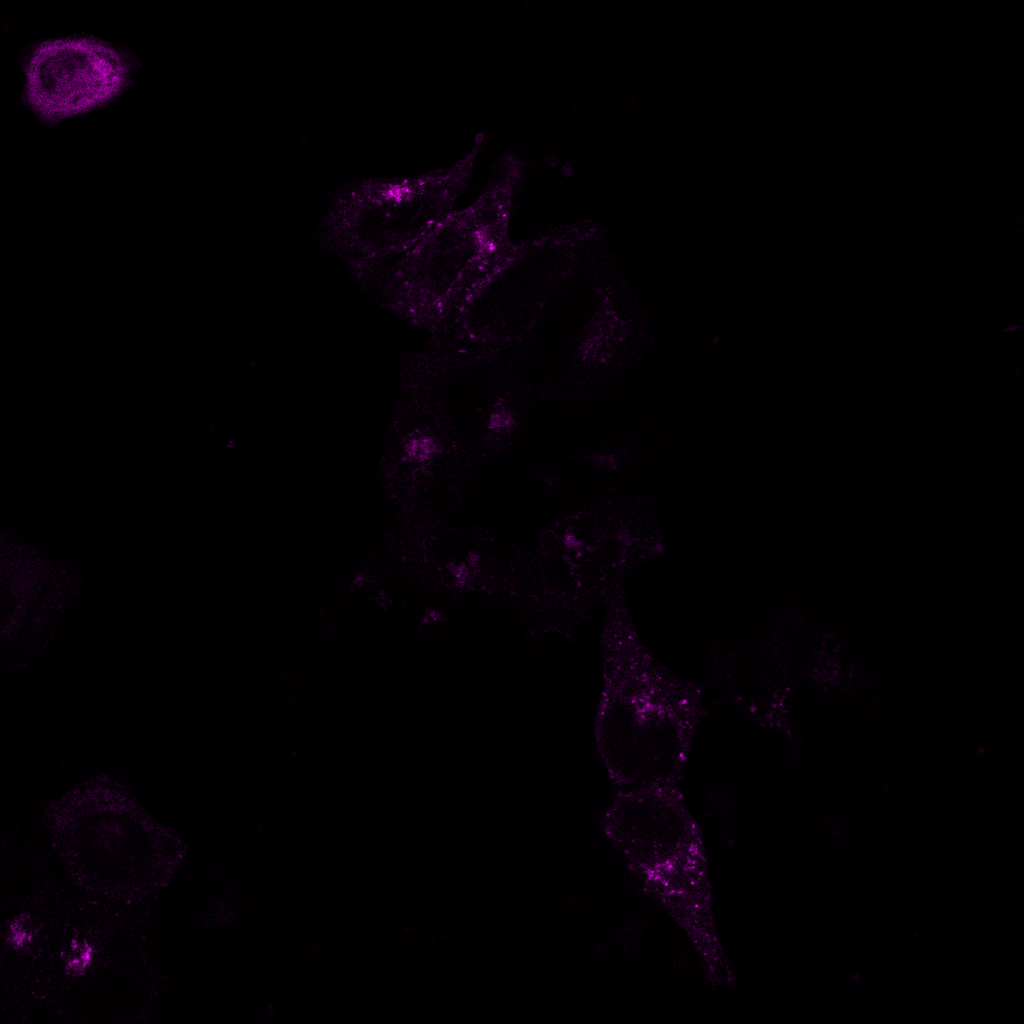

Supplement: Supplementary file 4 — Source data Fig. 2 [file 44318_2024_233_MOESM4_ESM.zip › 2A/HeLa sgATG16L1 Ctrl_Series001_ch02_SV.tif]

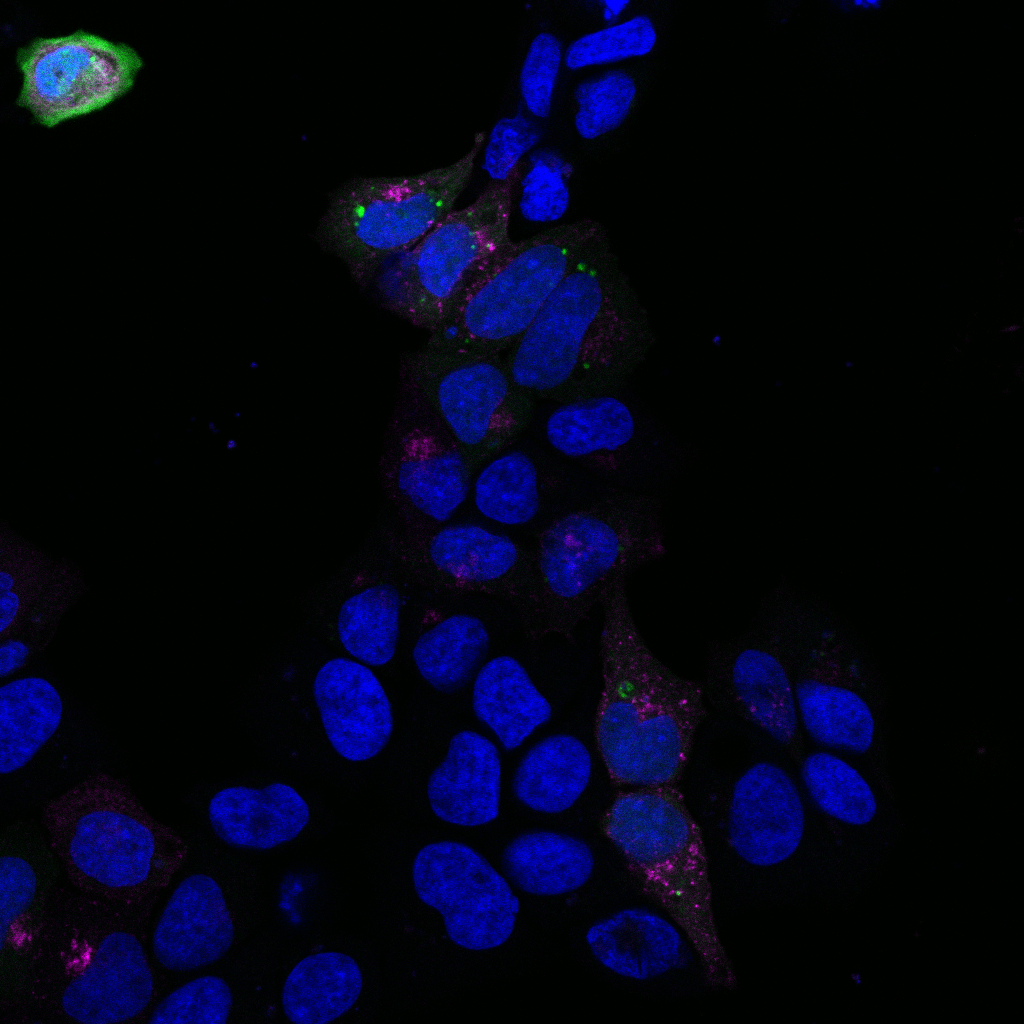

Supplement: Supplementary file 4 — Source data Fig. 2 [file 44318_2024_233_MOESM4_ESM.zip › 2A/HeLa sgATG16L1 Ctrl_Series001_overlay.tif]

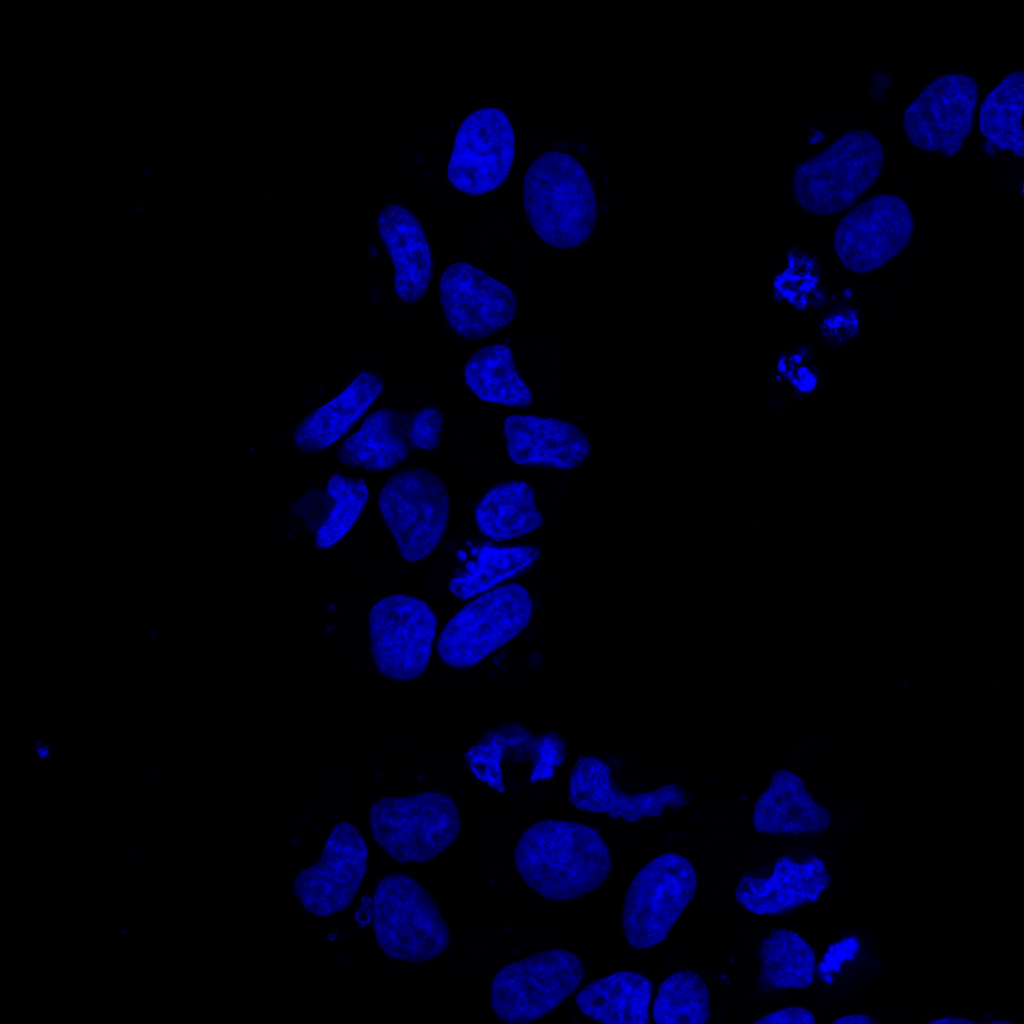

Supplement: Supplementary file 4 — Source data Fig. 2 [file 44318_2024_233_MOESM4_ESM.zip › 2A/HeLa sgATG16L1 DLK1_Series002_ch00_SV.tif]

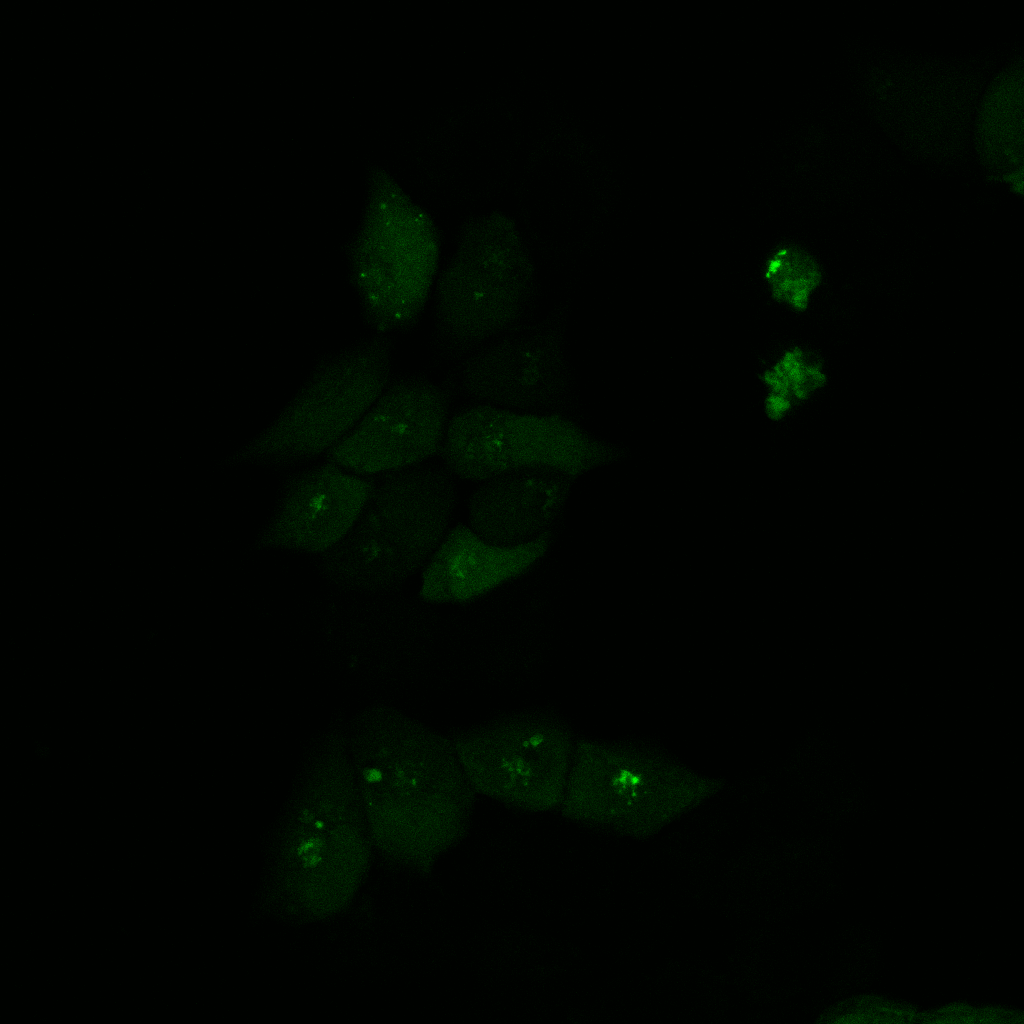

Supplement: Supplementary file 4 — Source data Fig. 2 [file 44318_2024_233_MOESM4_ESM.zip › 2A/HeLa sgATG16L1 DLK1_Series002_ch01_SV.tif]

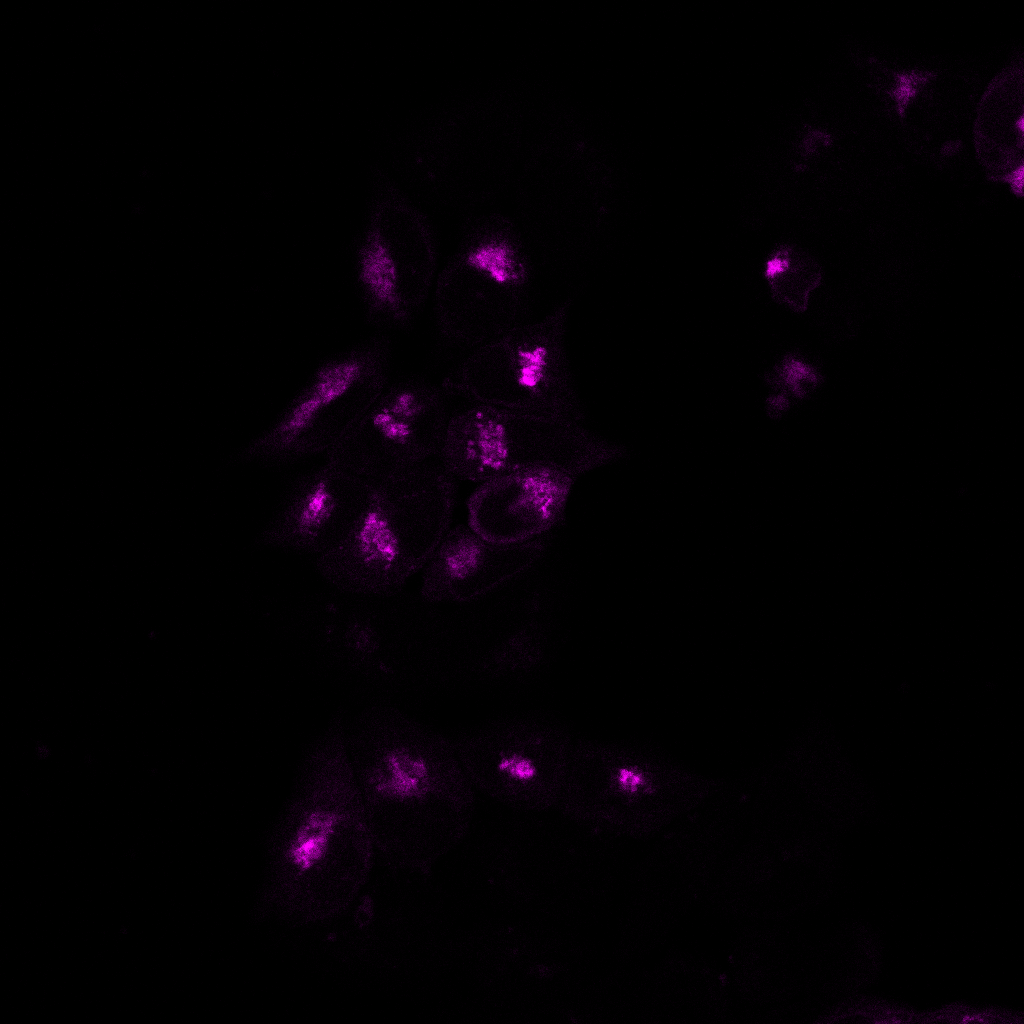

Supplement: Supplementary file 4 — Source data Fig. 2 [file 44318_2024_233_MOESM4_ESM.zip › 2A/HeLa sgATG16L1 DLK1_Series002_ch02_SV.tif]

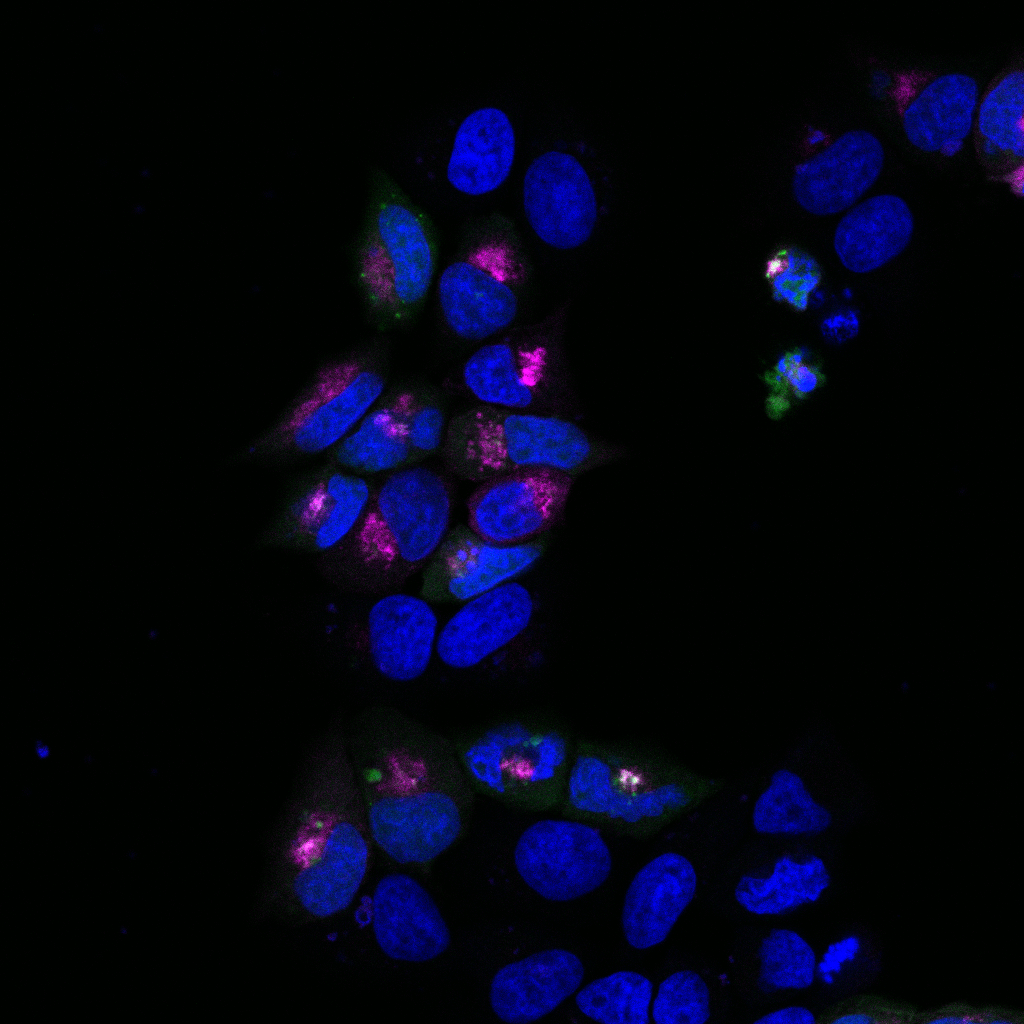

Supplement: Supplementary file 4 — Source data Fig. 2 [file 44318_2024_233_MOESM4_ESM.zip › 2A/HeLa sgATG16L1 DLK1_Series002_overlay.tif]

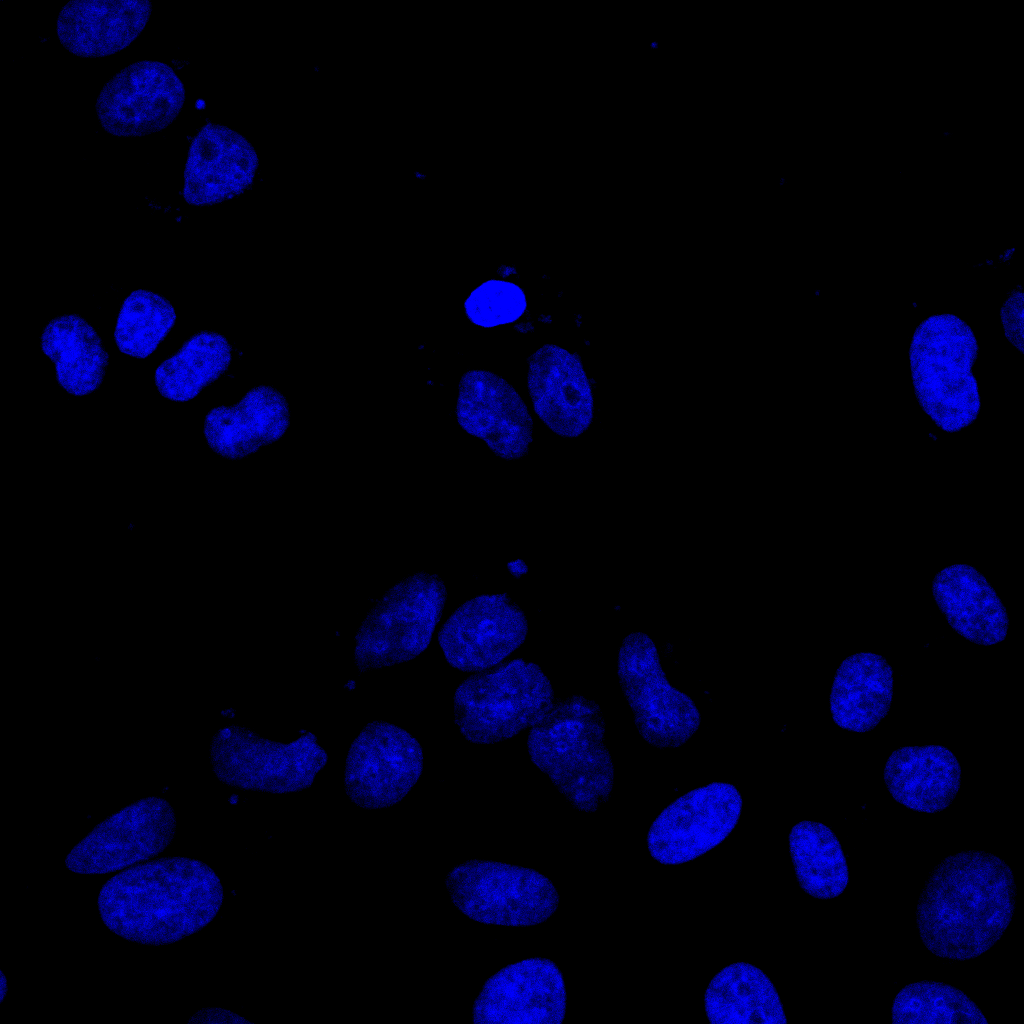

Supplement: Supplementary file 4 — Source data Fig. 2 [file 44318_2024_233_MOESM4_ESM.zip › 2A/HeLa sgCtrl Ctrl_Series001_ch00_SV.tif]

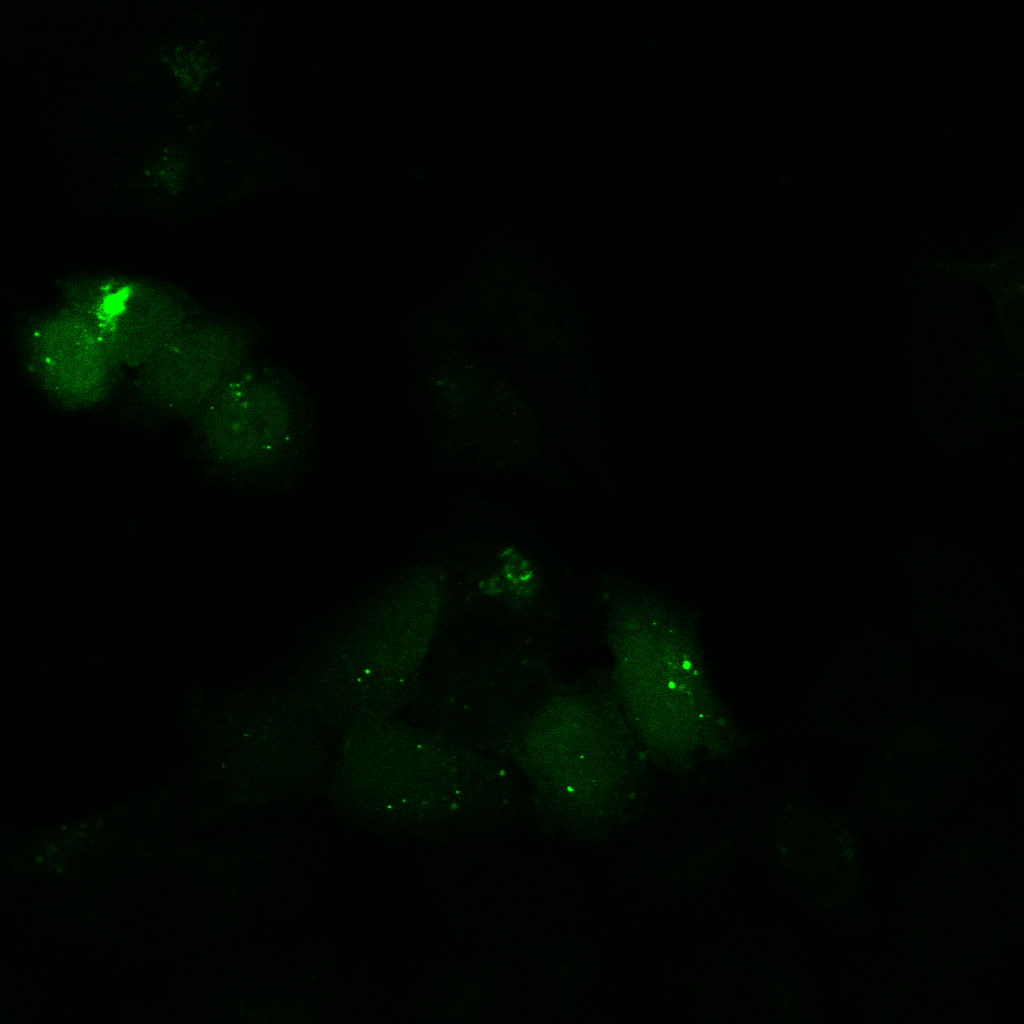

Supplement: Supplementary file 4 — Source data Fig. 2 [file 44318_2024_233_MOESM4_ESM.zip › 2A/HeLa sgCtrl Ctrl_Series001_ch01_SV.tif]

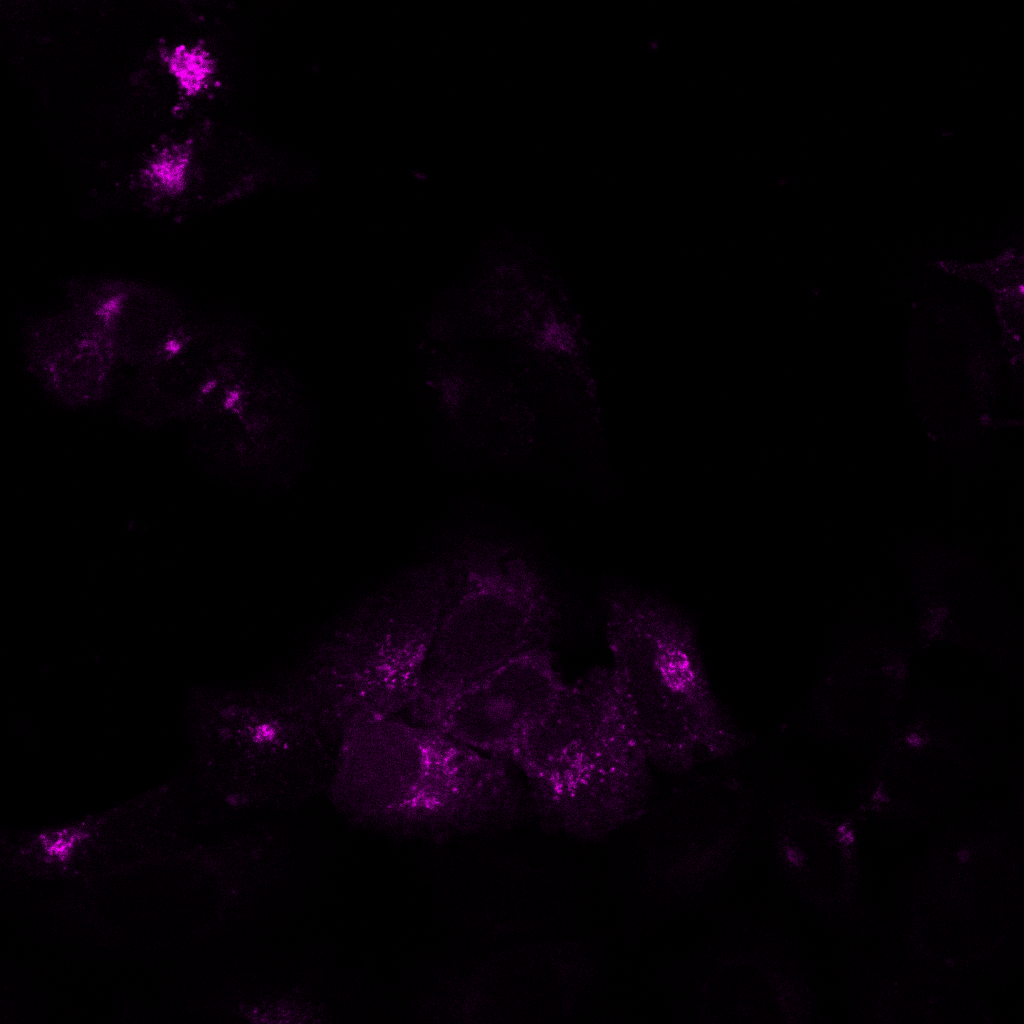

Supplement: Supplementary file 4 — Source data Fig. 2 [file 44318_2024_233_MOESM4_ESM.zip › 2A/HeLa sgCtrl Ctrl_Series001_ch02_SV.tif]

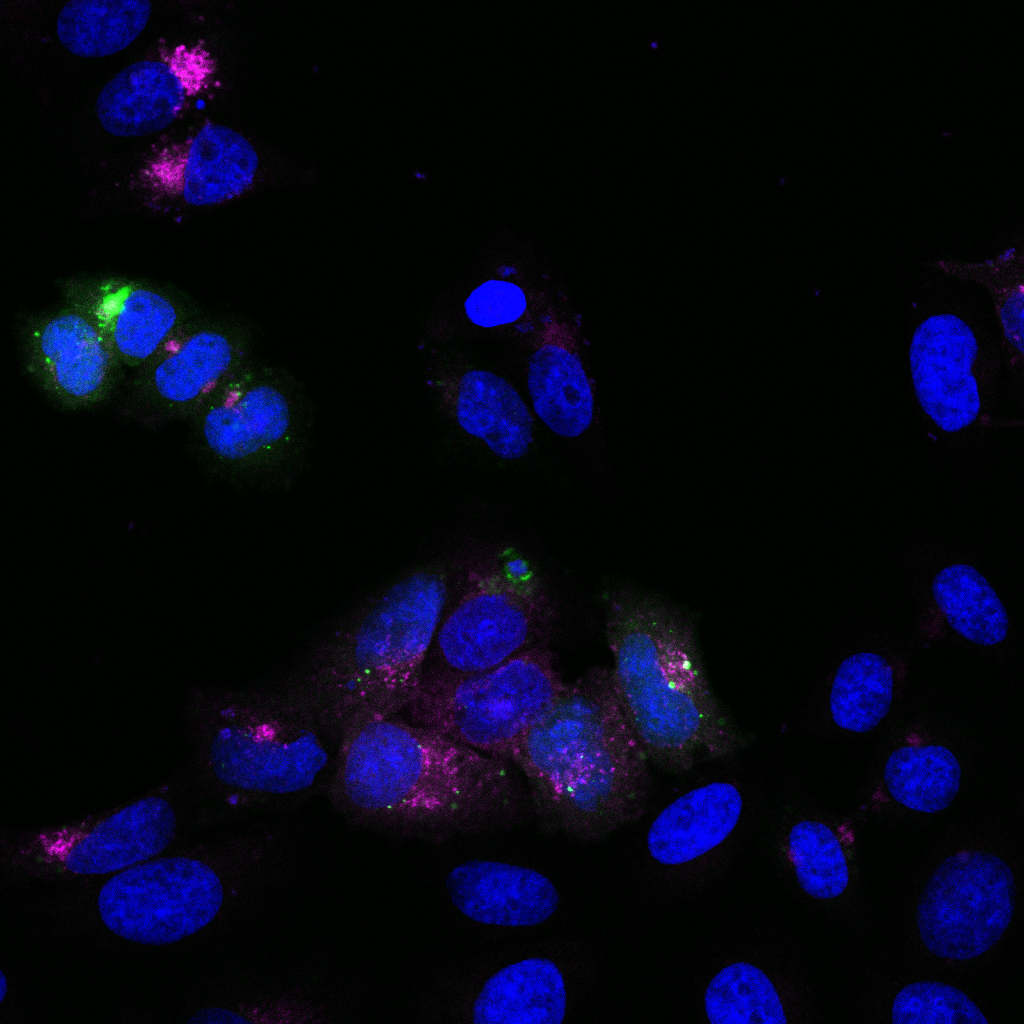

Supplement: Supplementary file 4 — Source data Fig. 2 [file 44318_2024_233_MOESM4_ESM.zip › 2A/HeLa sgCtrl Ctrl_Series001_overlay.tif]

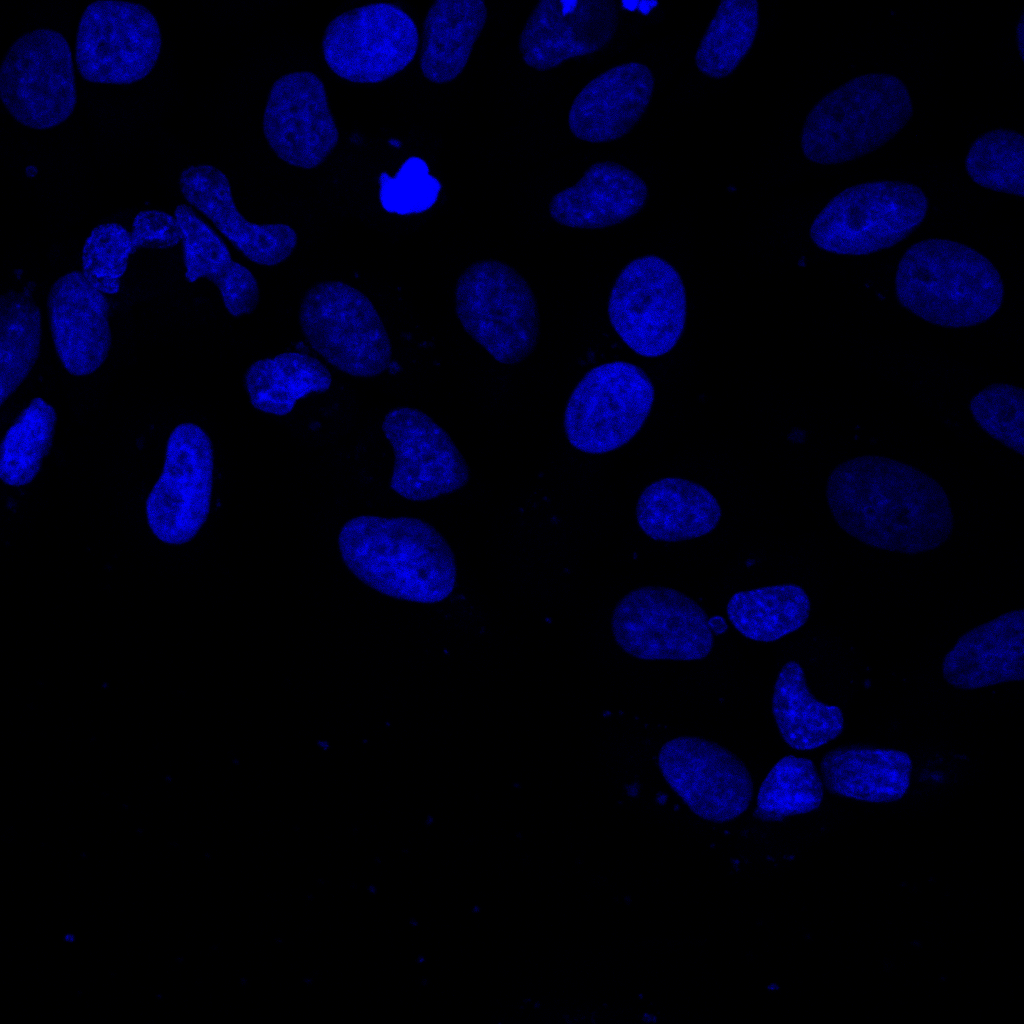

Supplement: Supplementary file 4 — Source data Fig. 2 [file 44318_2024_233_MOESM4_ESM.zip › 2A/HeLa sgCtrl DLK1_Series001_ch00_SV.tif]

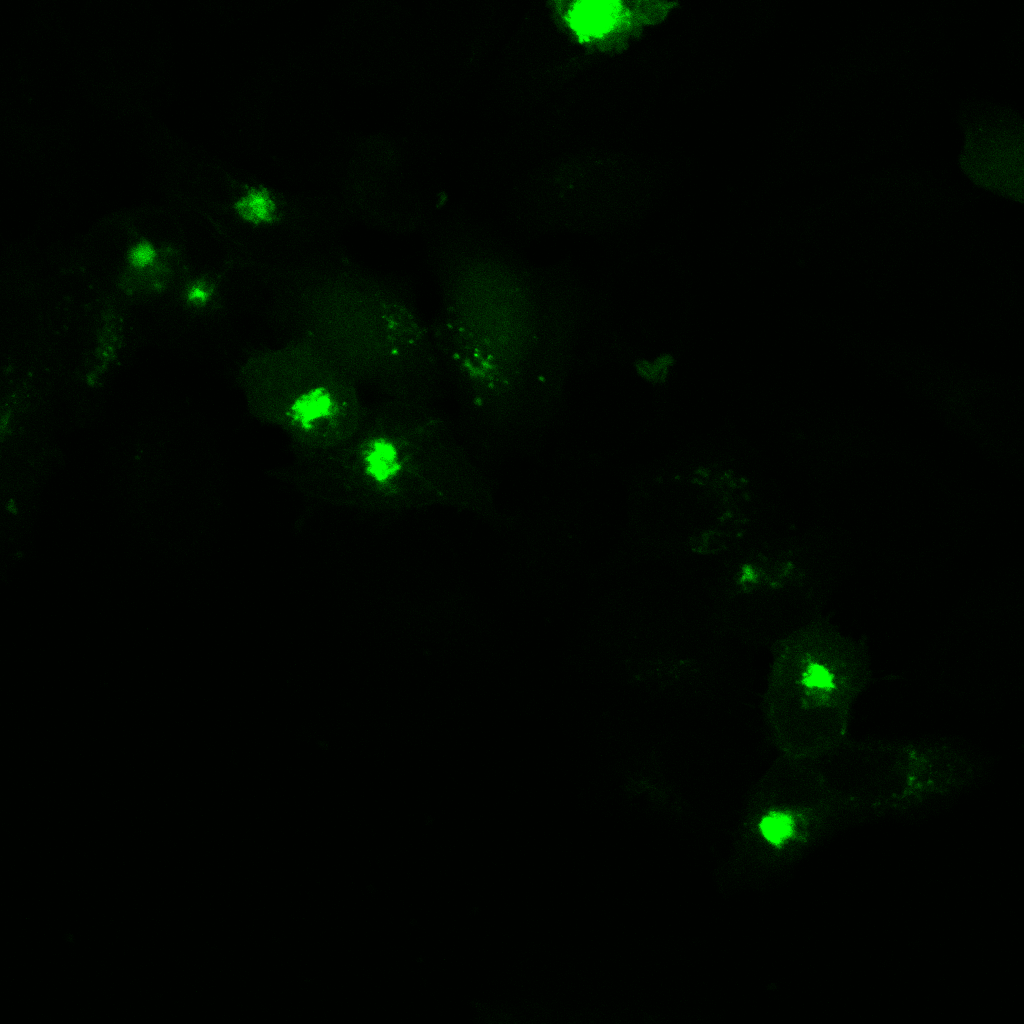

Supplement: Supplementary file 4 — Source data Fig. 2 [file 44318_2024_233_MOESM4_ESM.zip › 2A/HeLa sgCtrl DLK1_Series001_ch01_SV.tif]

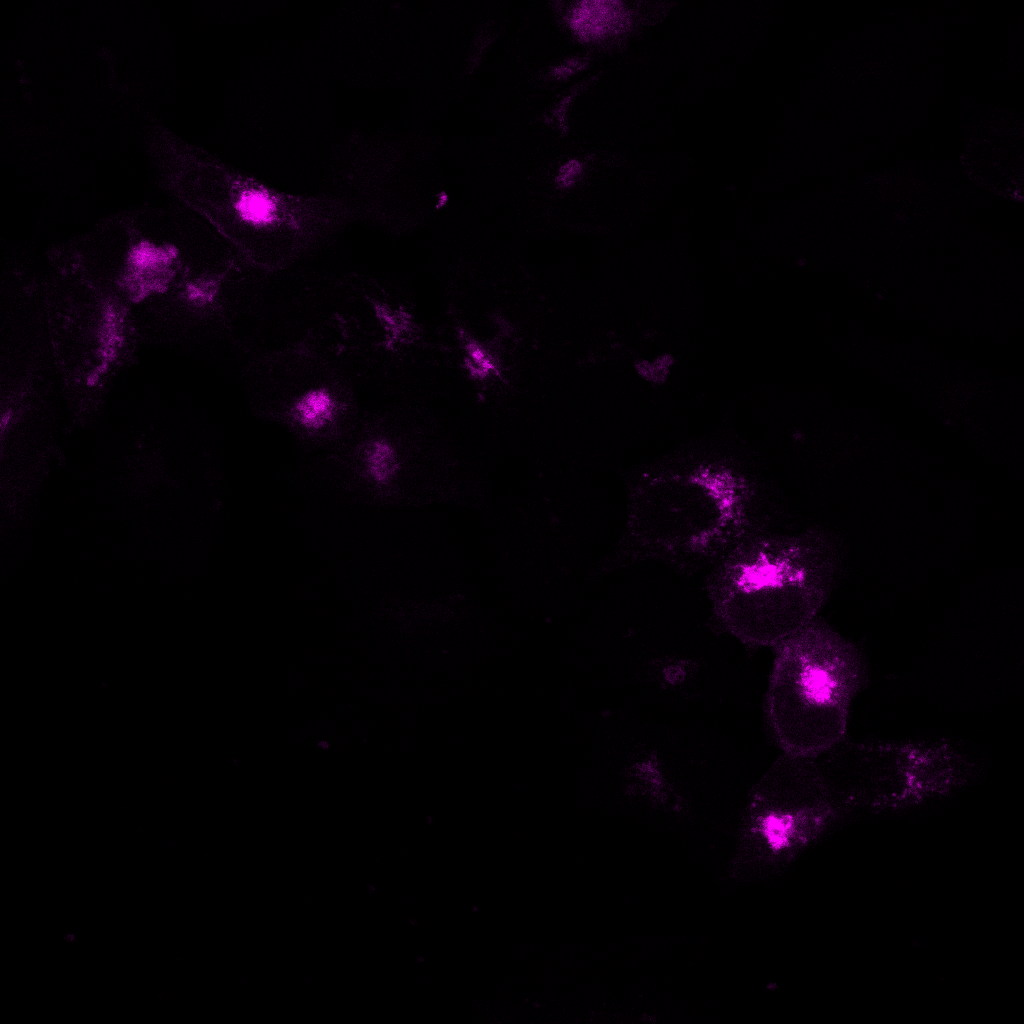

Supplement: Supplementary file 4 — Source data Fig. 2 [file 44318_2024_233_MOESM4_ESM.zip › 2A/HeLa sgCtrl DLK1_Series001_ch02_SV.tif]

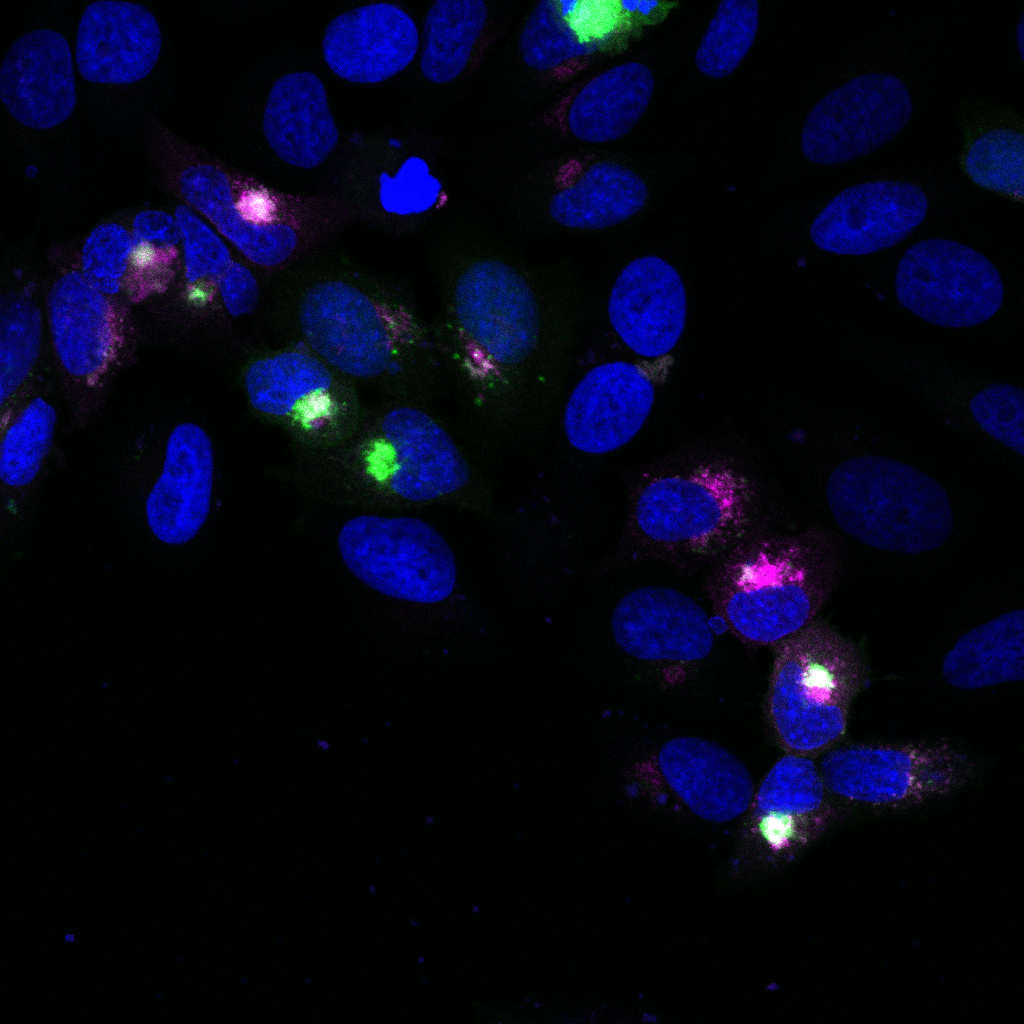

Supplement: Supplementary file 4 — Source data Fig. 2 [file 44318_2024_233_MOESM4_ESM.zip › 2A/HeLa sgCtrl DLK1_Series001_overlay.tif]

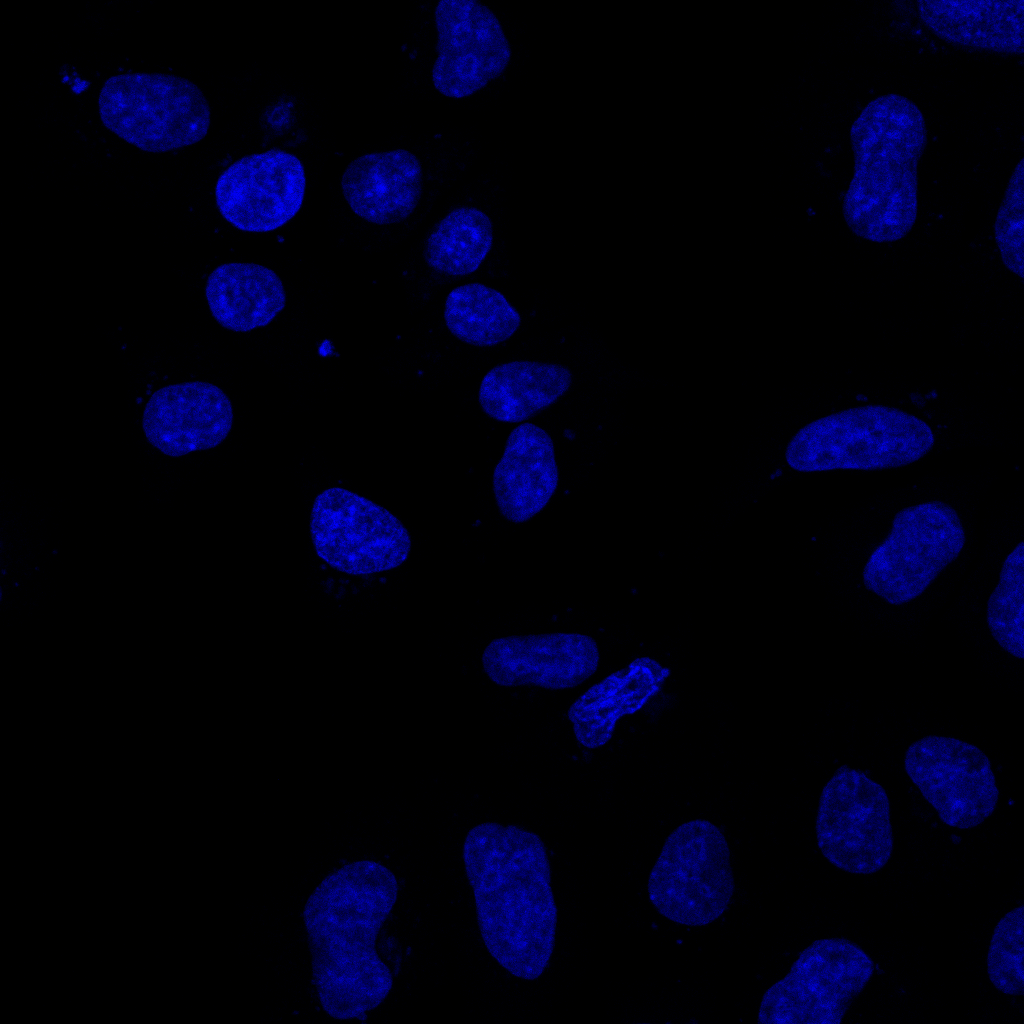

Supplement: Supplementary file 4 — Source data Fig. 2 [file 44318_2024_233_MOESM4_ESM.zip › 2A/HeLa sgFIP200 Ctrl_Series001_ch00_SV.tif]

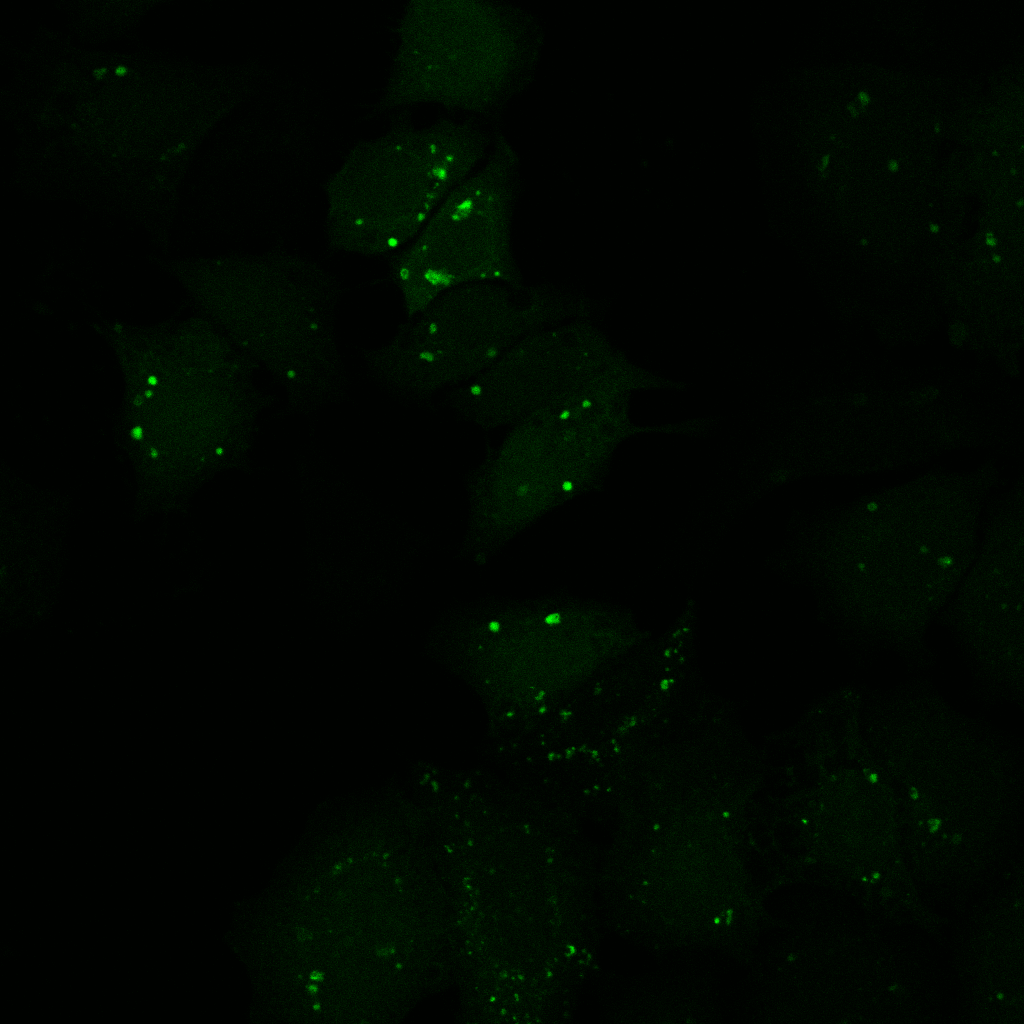

Supplement: Supplementary file 4 — Source data Fig. 2 [file 44318_2024_233_MOESM4_ESM.zip › 2A/HeLa sgFIP200 Ctrl_Series001_ch01_SV.tif]

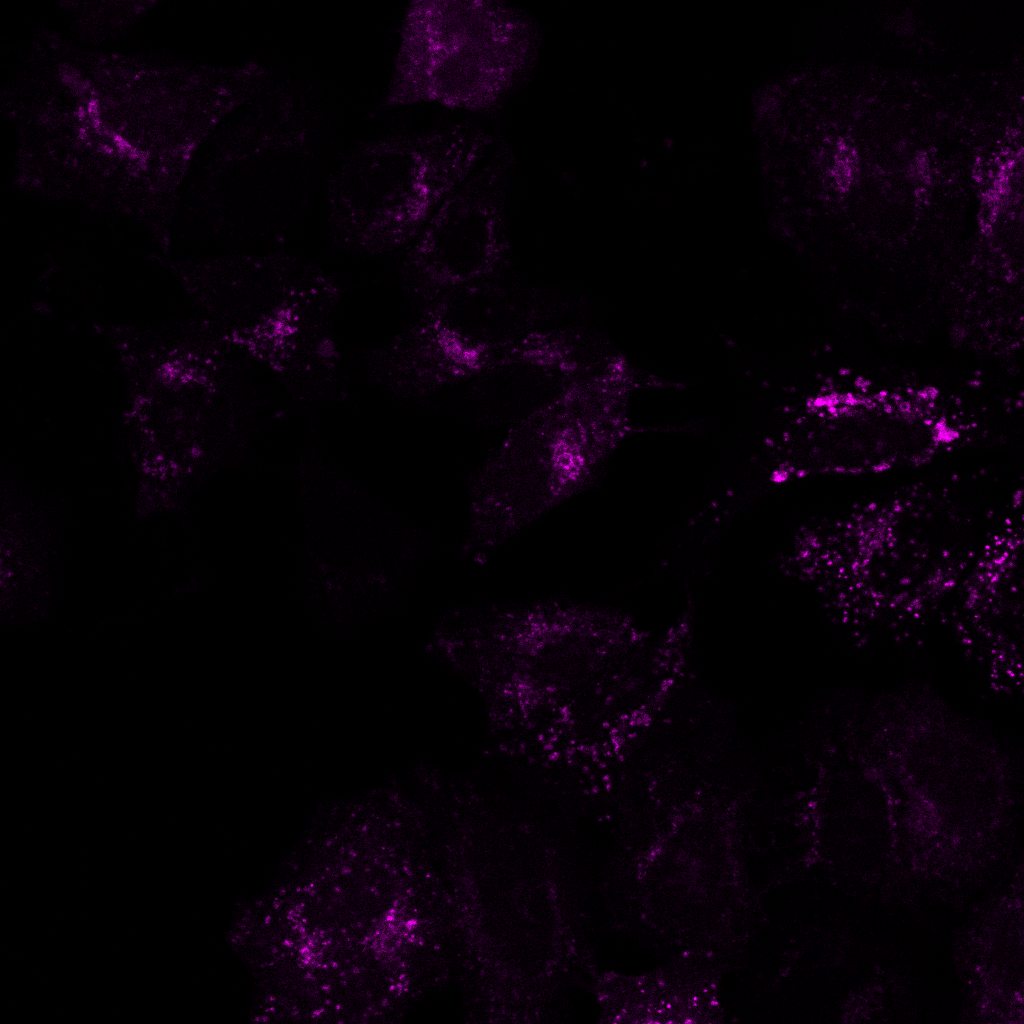

Supplement: Supplementary file 4 — Source data Fig. 2 [file 44318_2024_233_MOESM4_ESM.zip › 2A/HeLa sgFIP200 Ctrl_Series001_ch02_SV.tif]

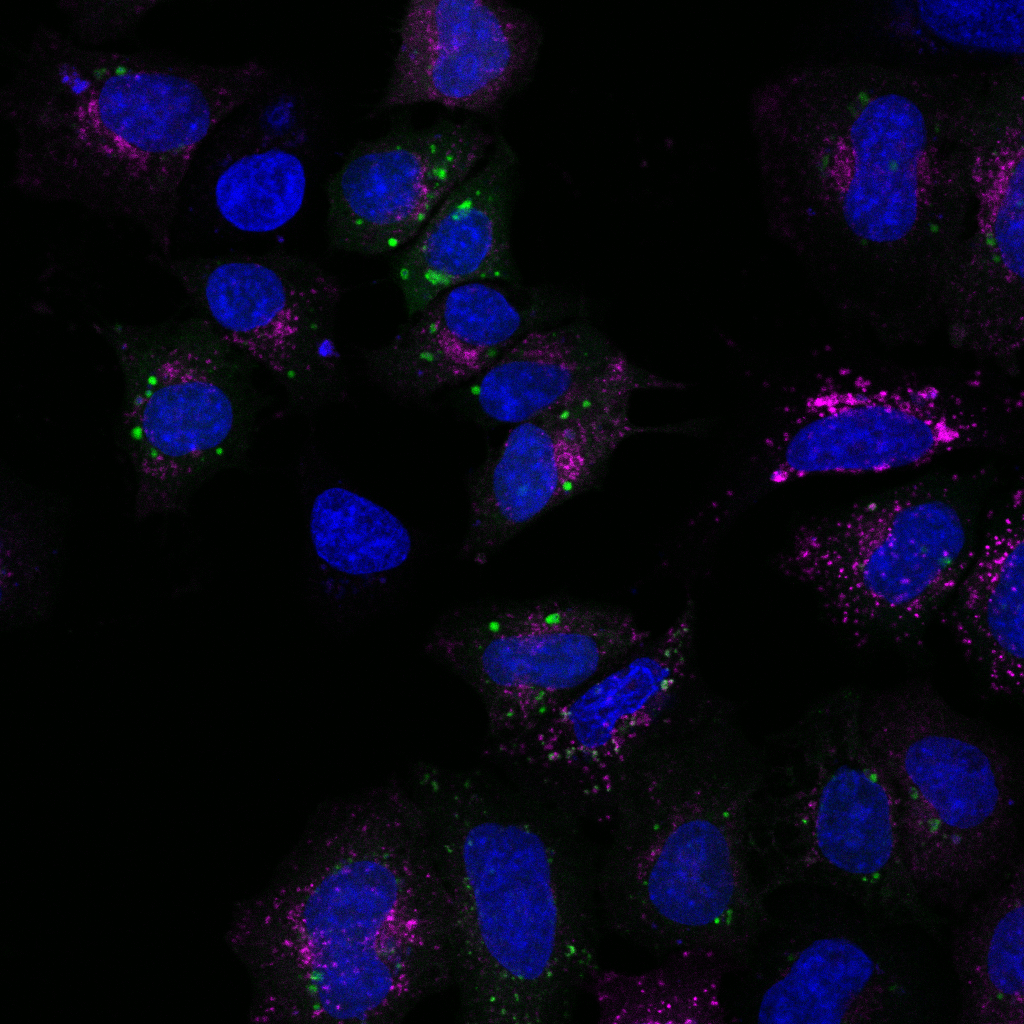

Supplement: Supplementary file 4 — Source data Fig. 2 [file 44318_2024_233_MOESM4_ESM.zip › 2A/HeLa sgFIP200 Ctrl_Series001_overlay.tif]

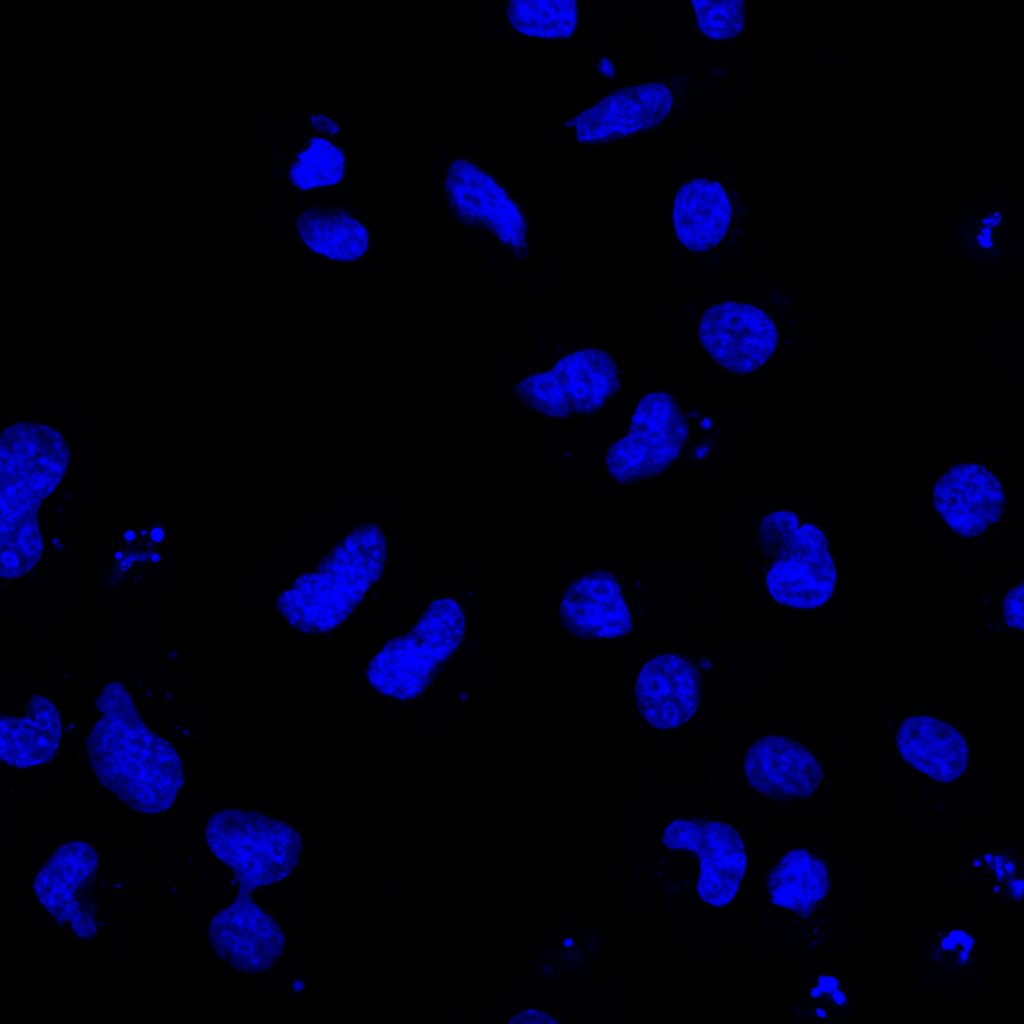

Supplement: Supplementary file 4 — Source data Fig. 2 [file 44318_2024_233_MOESM4_ESM.zip › 2A/HeLa sgFIP200 DLK1_Series001_ch00_SV.tif]

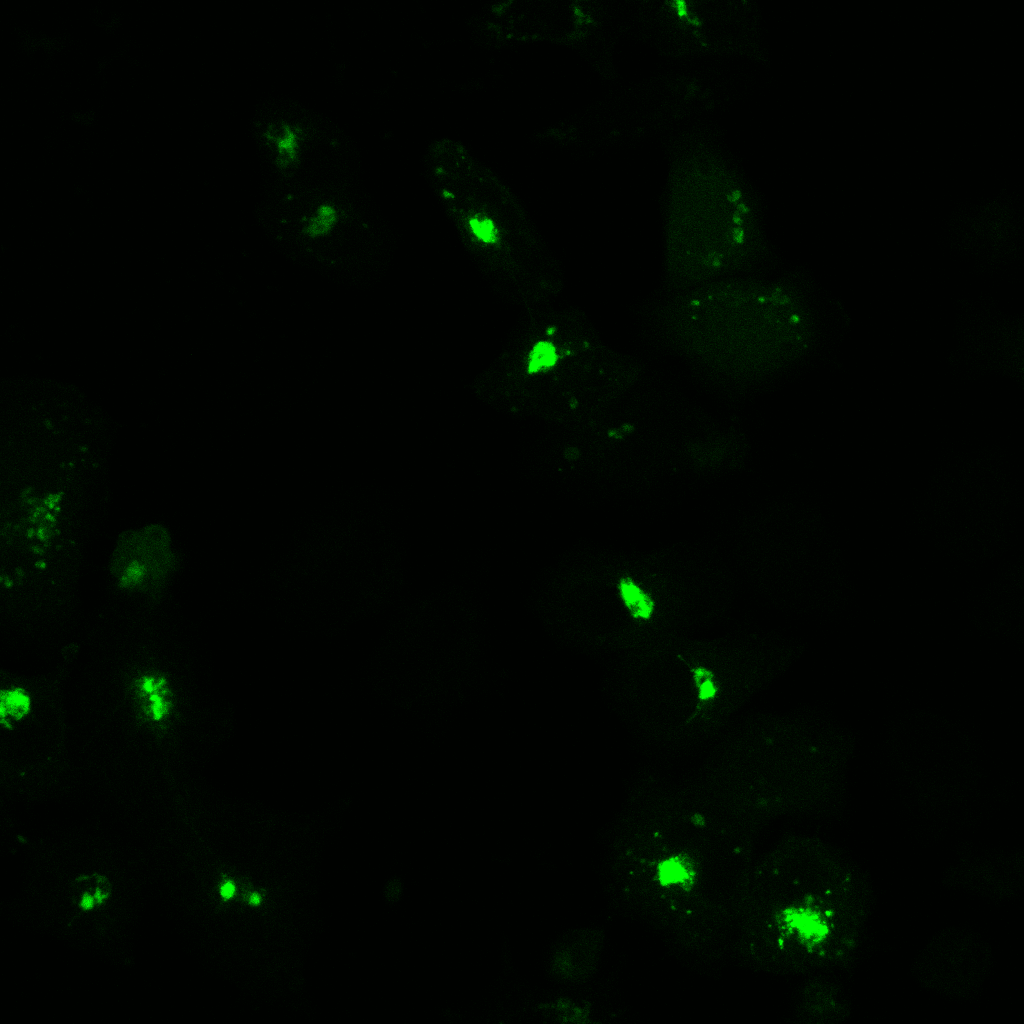

Supplement: Supplementary file 4 — Source data Fig. 2 [file 44318_2024_233_MOESM4_ESM.zip › 2A/HeLa sgFIP200 DLK1_Series001_ch01_SV.tif]

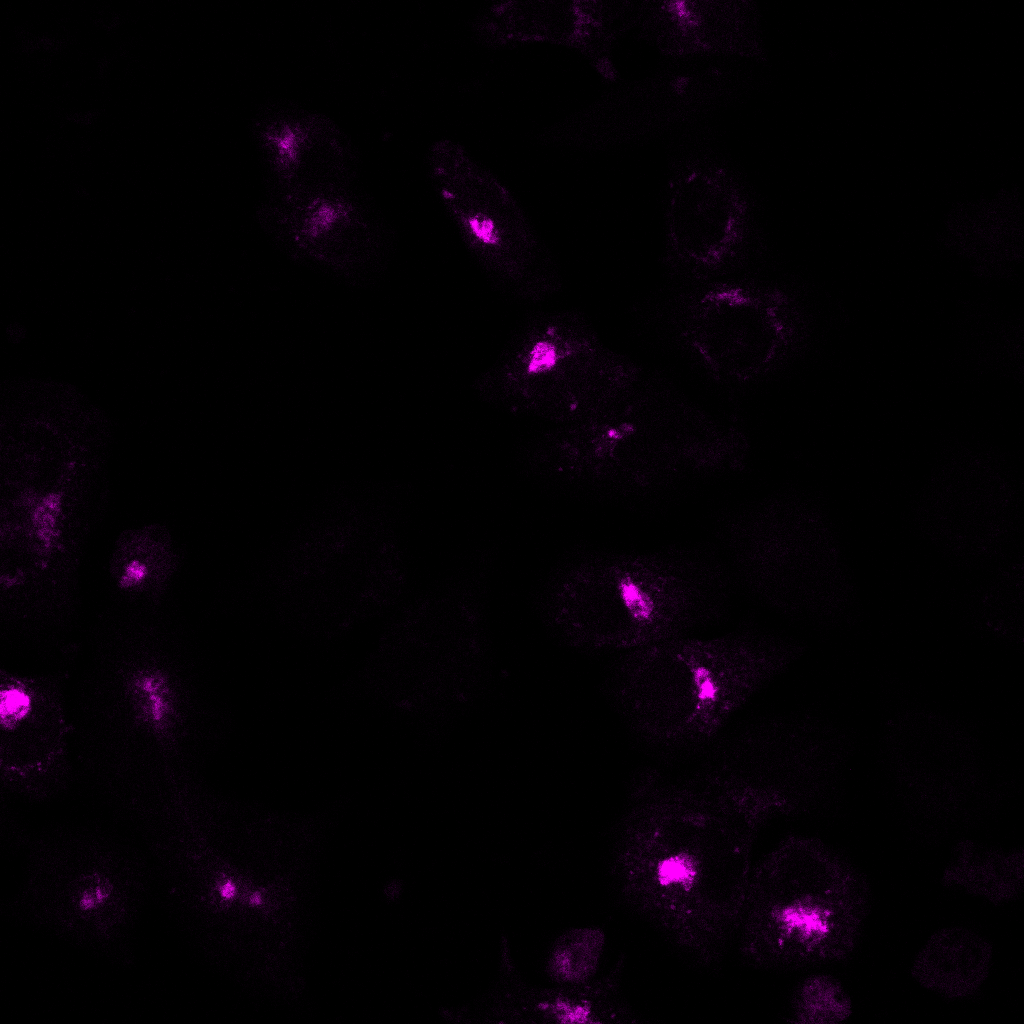

Supplement: Supplementary file 4 — Source data Fig. 2 [file 44318_2024_233_MOESM4_ESM.zip › 2A/HeLa sgFIP200 DLK1_Series001_ch02_SV.tif]

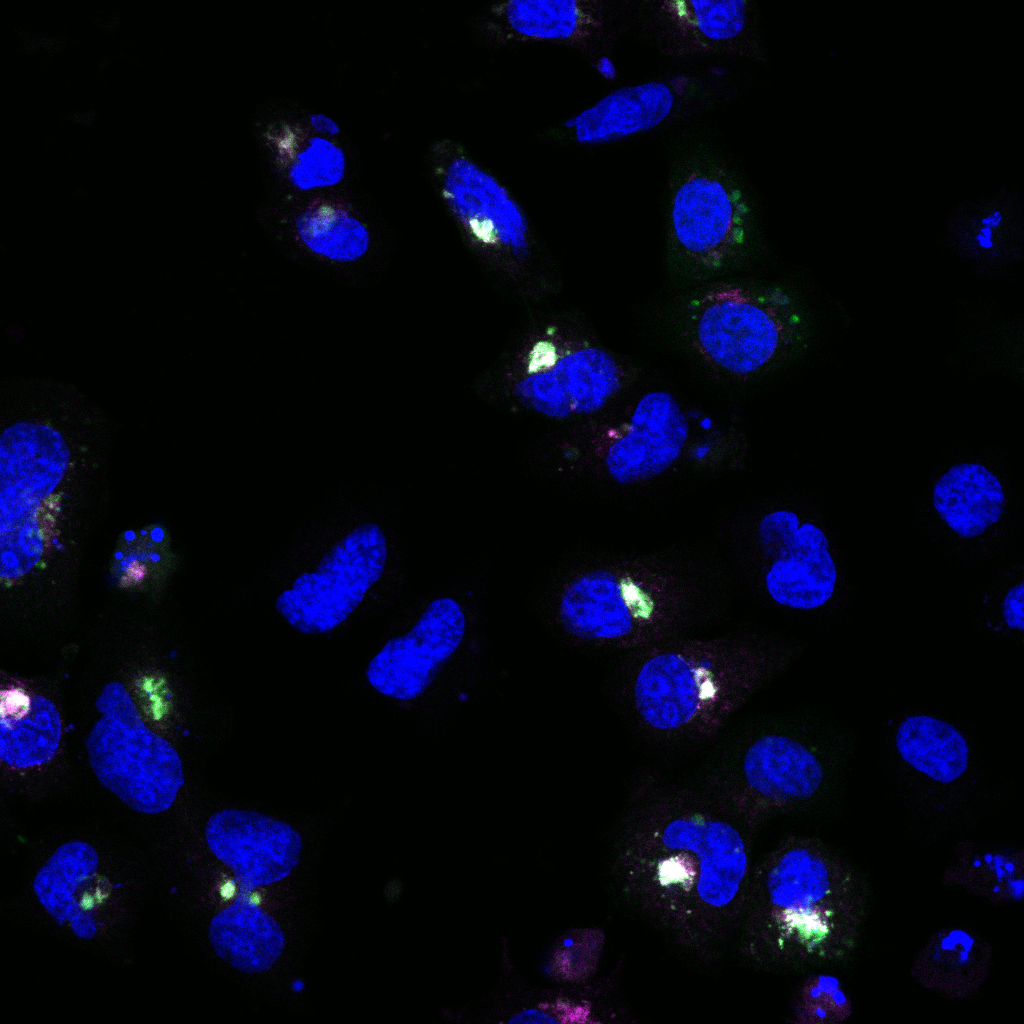

Supplement: Supplementary file 4 — Source data Fig. 2 [file 44318_2024_233_MOESM4_ESM.zip › 2A/HeLa sgFIP200 DLK1_Series001_overlay.tif]

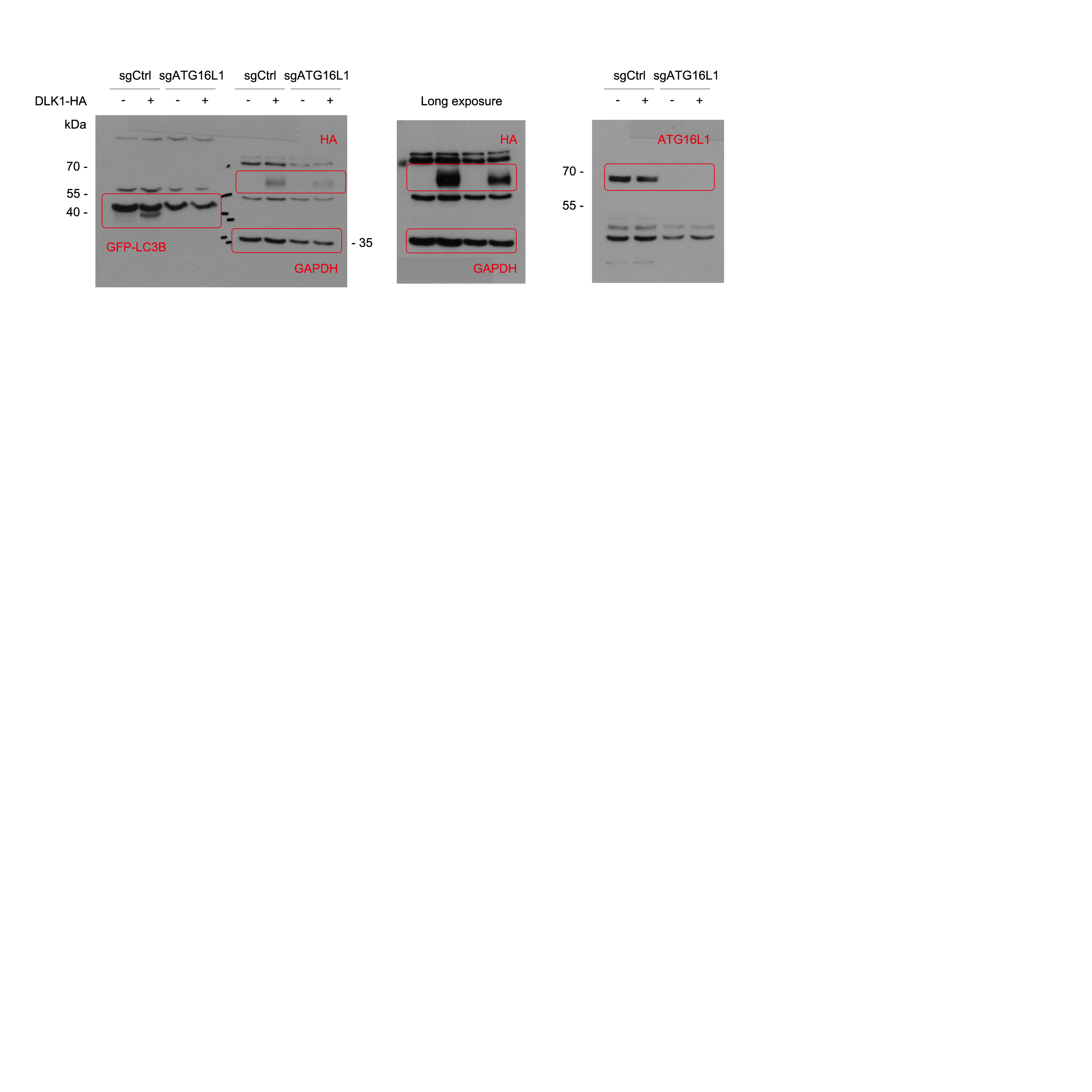

Supplement: Supplementary file 4 — Source data Fig. 2 [file 44318_2024_233_MOESM4_ESM.zip › 2B/Figure 2B.png]

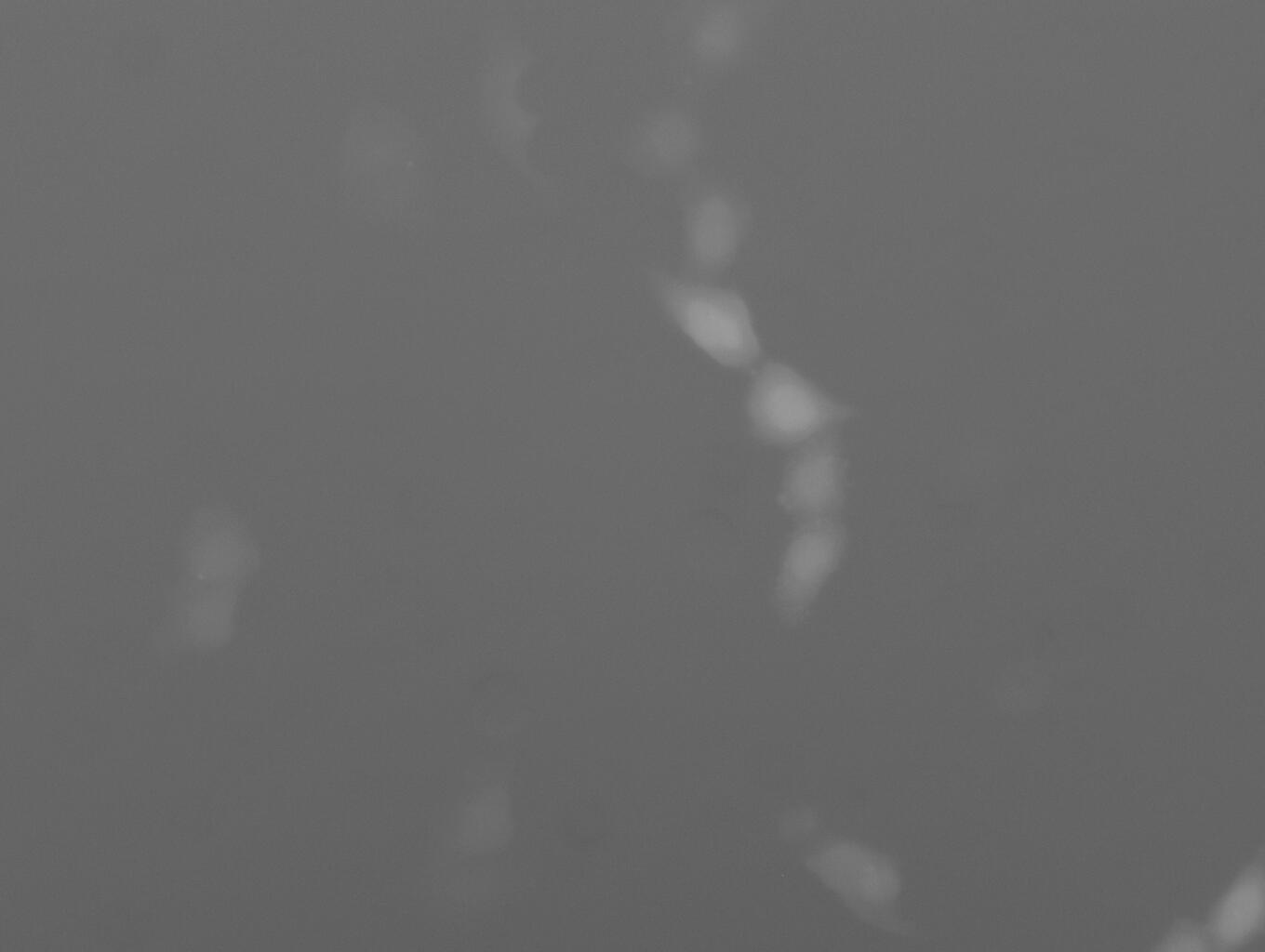

Supplement: Supplementary file 4 — Source data Fig. 2 [file 44318_2024_233_MOESM4_ESM.zip › 2D/Image/Ctrl GFP-LC3B G120A.jpg]

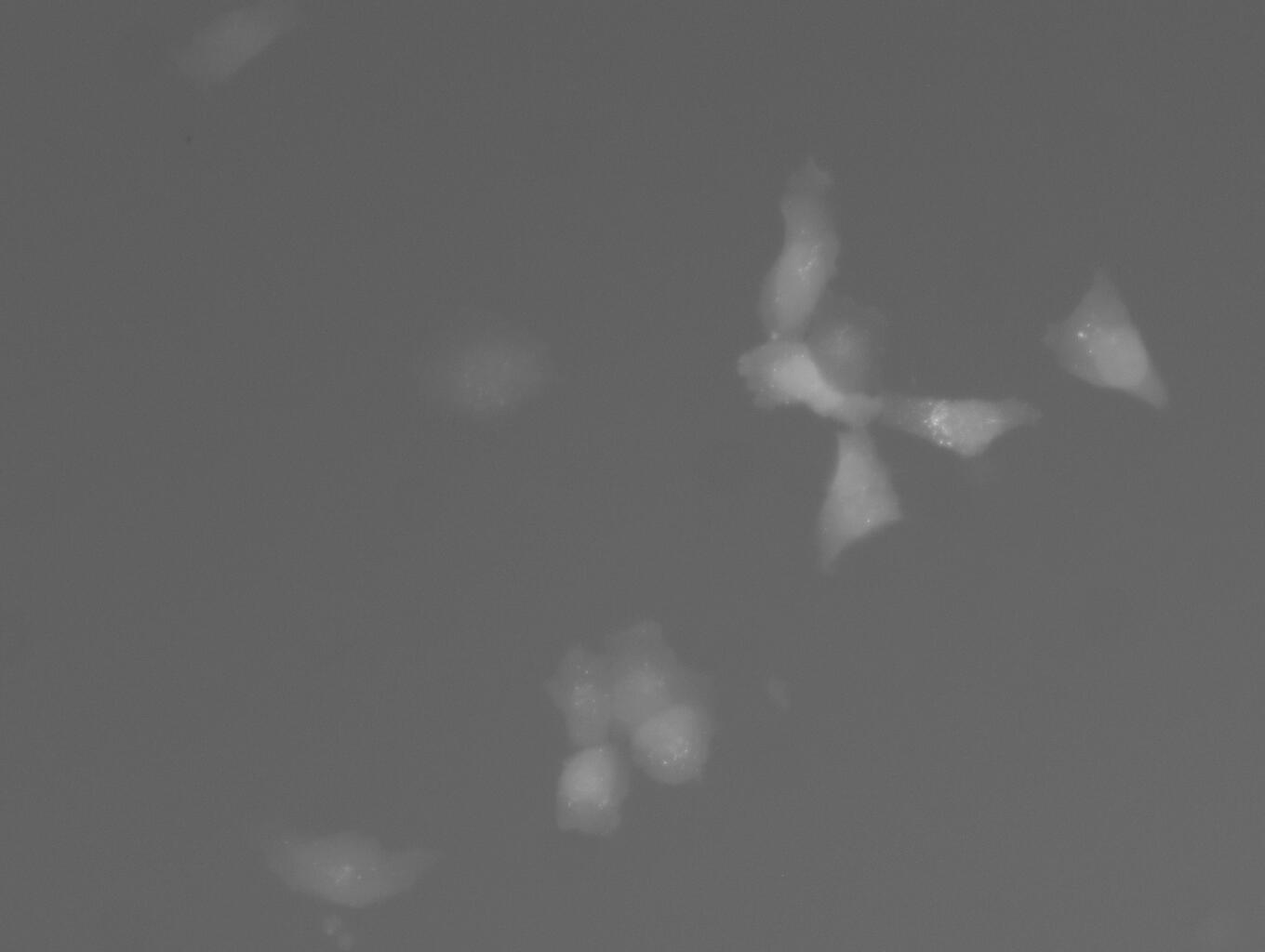

Supplement: Supplementary file 4 — Source data Fig. 2 [file 44318_2024_233_MOESM4_ESM.zip › 2D/Image/Ctrl GFP-LC3B WT.jpg]

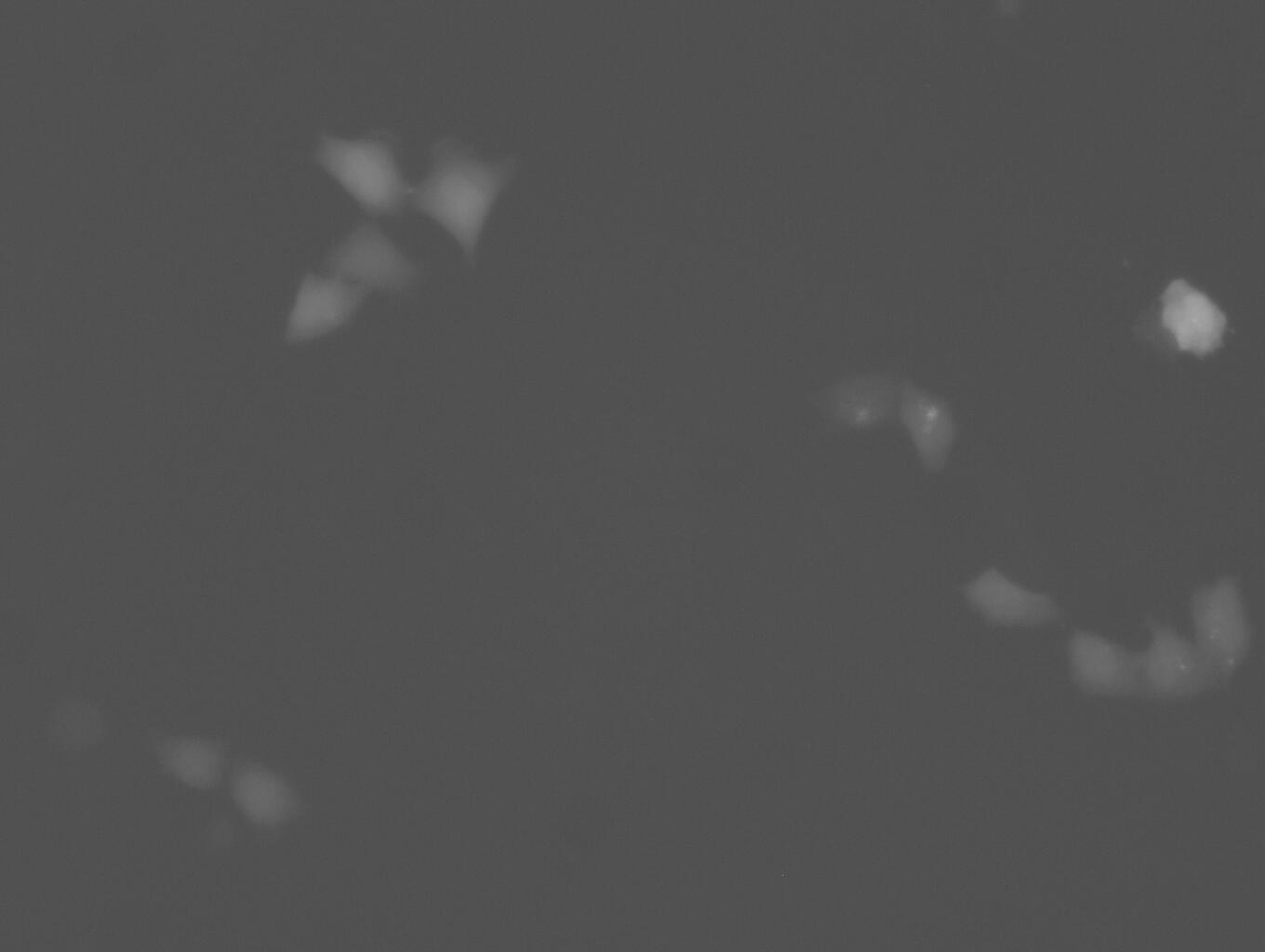

Supplement: Supplementary file 4 — Source data Fig. 2 [file 44318_2024_233_MOESM4_ESM.zip › 2D/Image/DLK1 GFP-LC3B G120A.jpg]

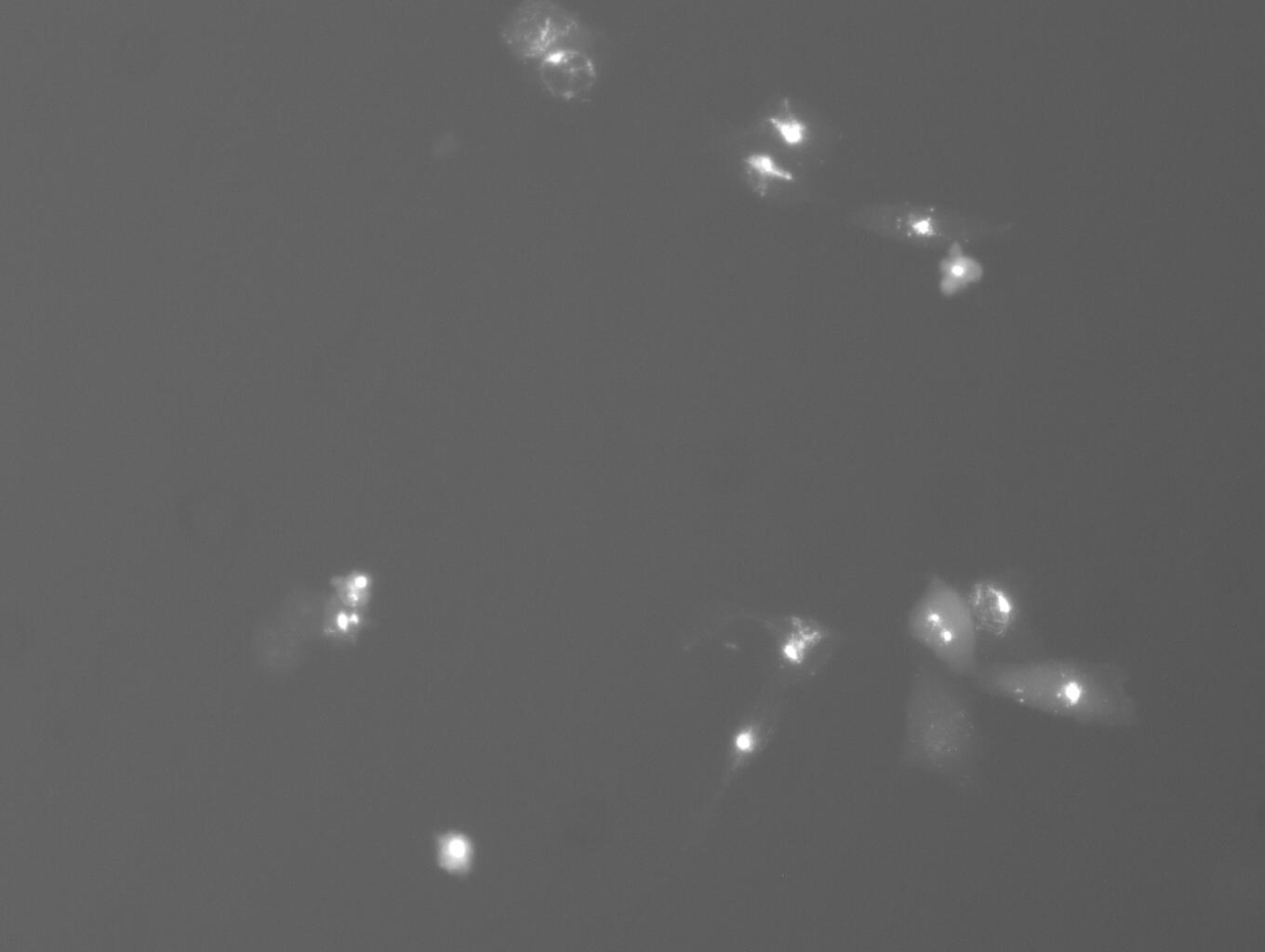

Supplement: Supplementary file 4 — Source data Fig. 2 [file 44318_2024_233_MOESM4_ESM.zip › 2D/Image/DLK1 GFP-LC3B WT.jpg]

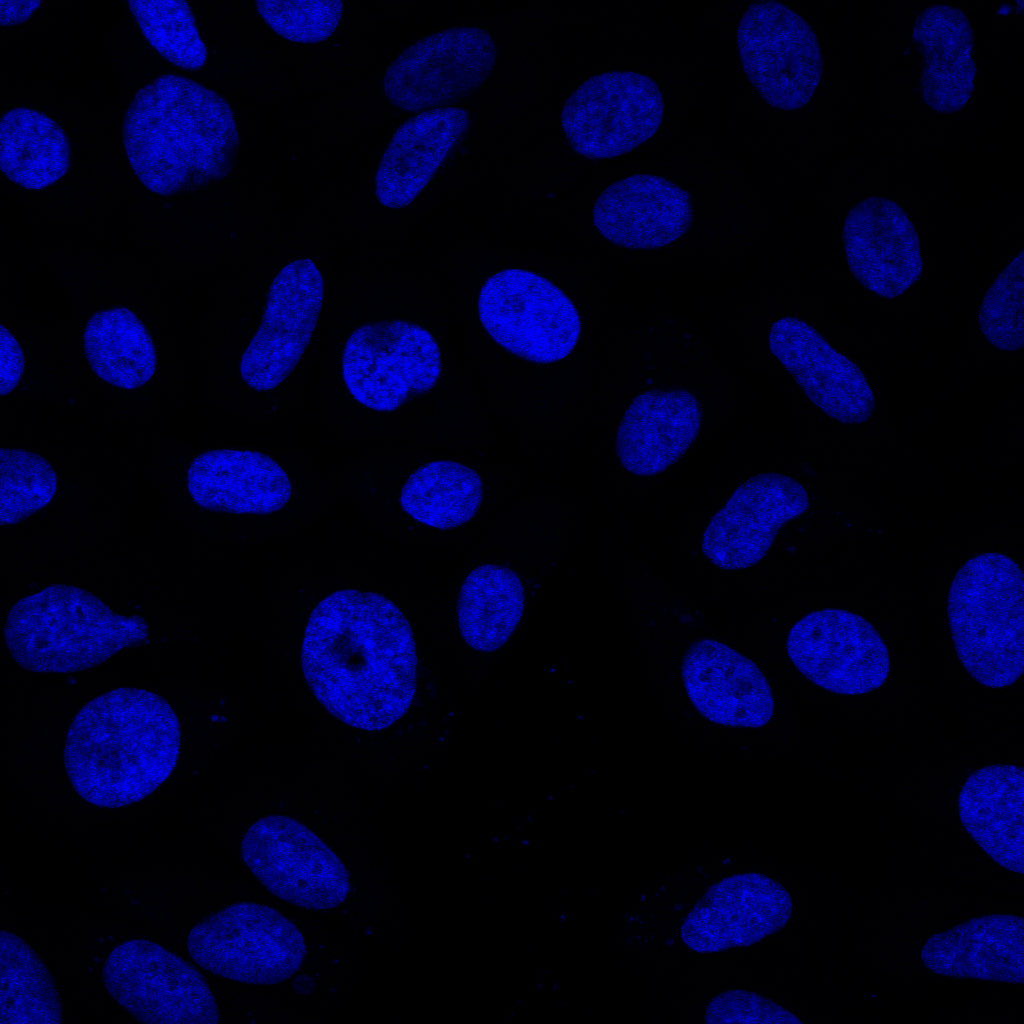

Supplement: Supplementary file 5 — Source data Fig. 3 [file 44318_2024_233_MOESM5_ESM.zip › 3A/Image/HeLa AMDE-1 GFP LC3 TGOLN2 RFP_Series002_ch00_SV.tif]

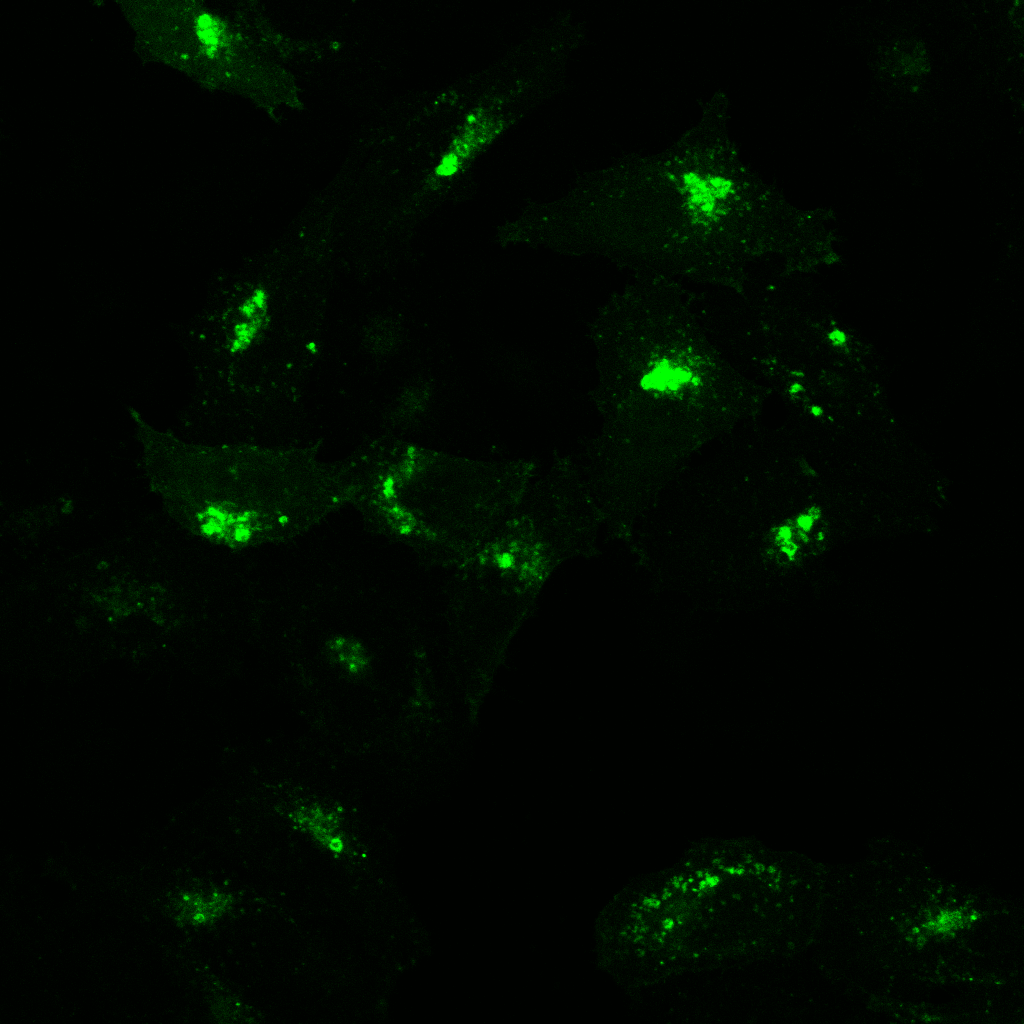

Supplement: Supplementary file 5 — Source data Fig. 3 [file 44318_2024_233_MOESM5_ESM.zip › 3A/Image/HeLa AMDE-1 GFP LC3 TGOLN2 RFP_Series002_ch01_SV.tif]

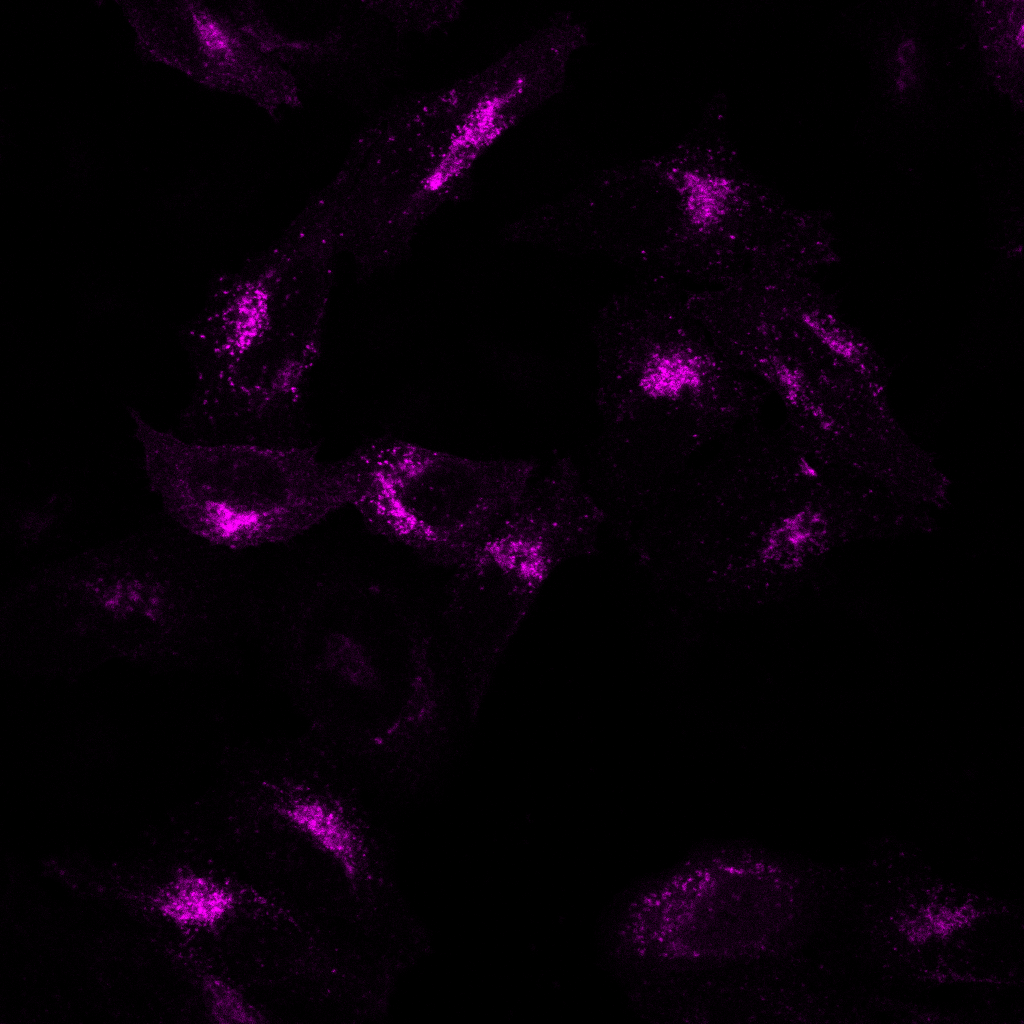

Supplement: Supplementary file 5 — Source data Fig. 3 [file 44318_2024_233_MOESM5_ESM.zip › 3A/Image/HeLa AMDE-1 GFP LC3 TGOLN2 RFP_Series002_ch02_SV.tif]

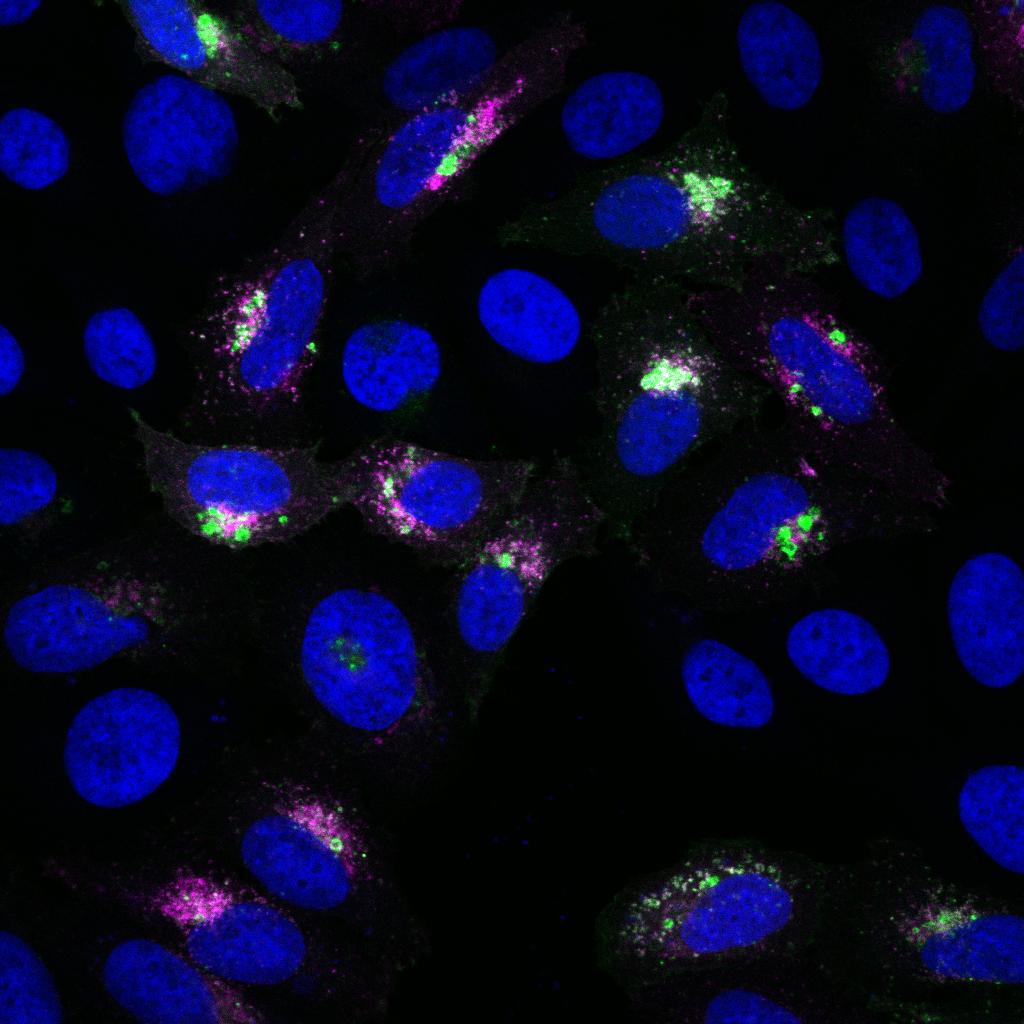

Supplement: Supplementary file 5 — Source data Fig. 3 [file 44318_2024_233_MOESM5_ESM.zip › 3A/Image/HeLa AMDE-1 GFP LC3 TGOLN2 RFP_Series002_overlay.tif]

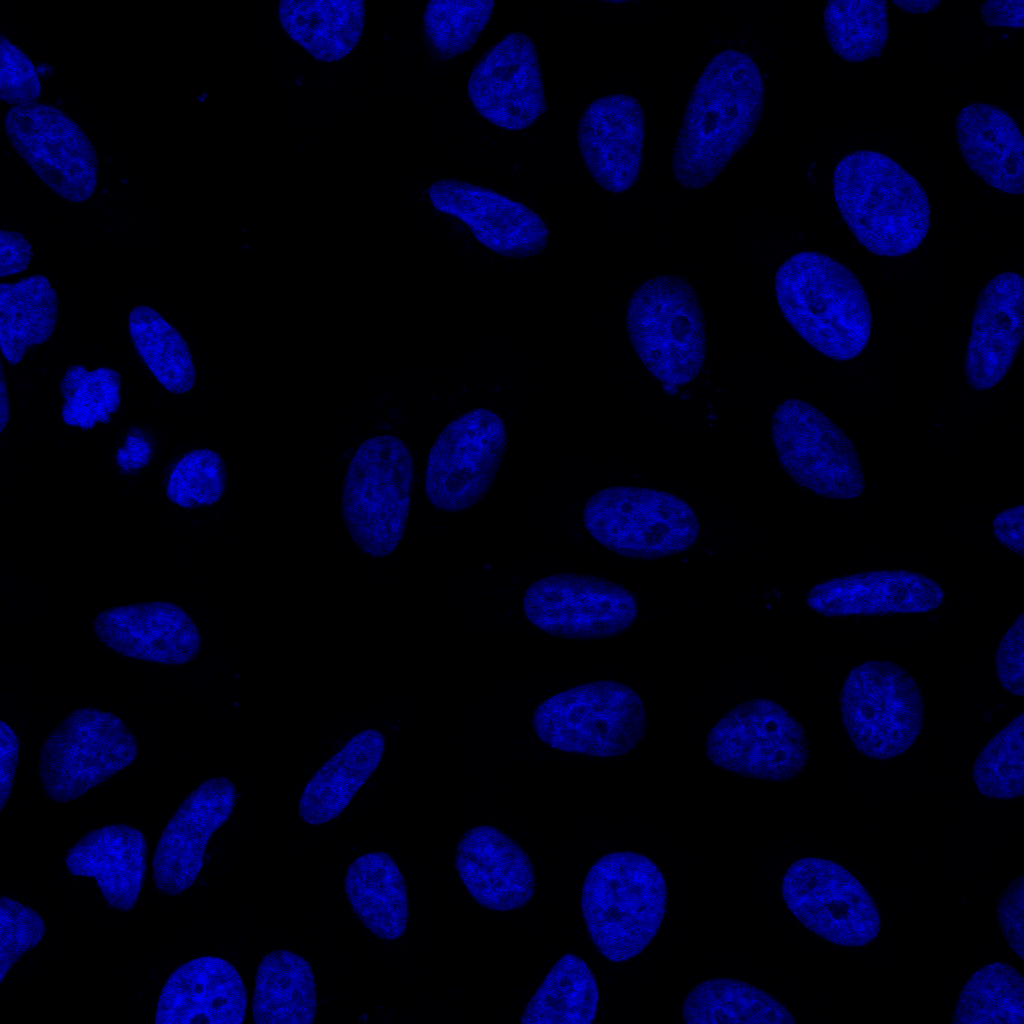

Supplement: Supplementary file 5 — Source data Fig. 3 [file 44318_2024_233_MOESM5_ESM.zip › 3A/Image/HeLa brefeldin a GFP LC3 TGOLN2 RFP_Series001_ch00_SV.tif]

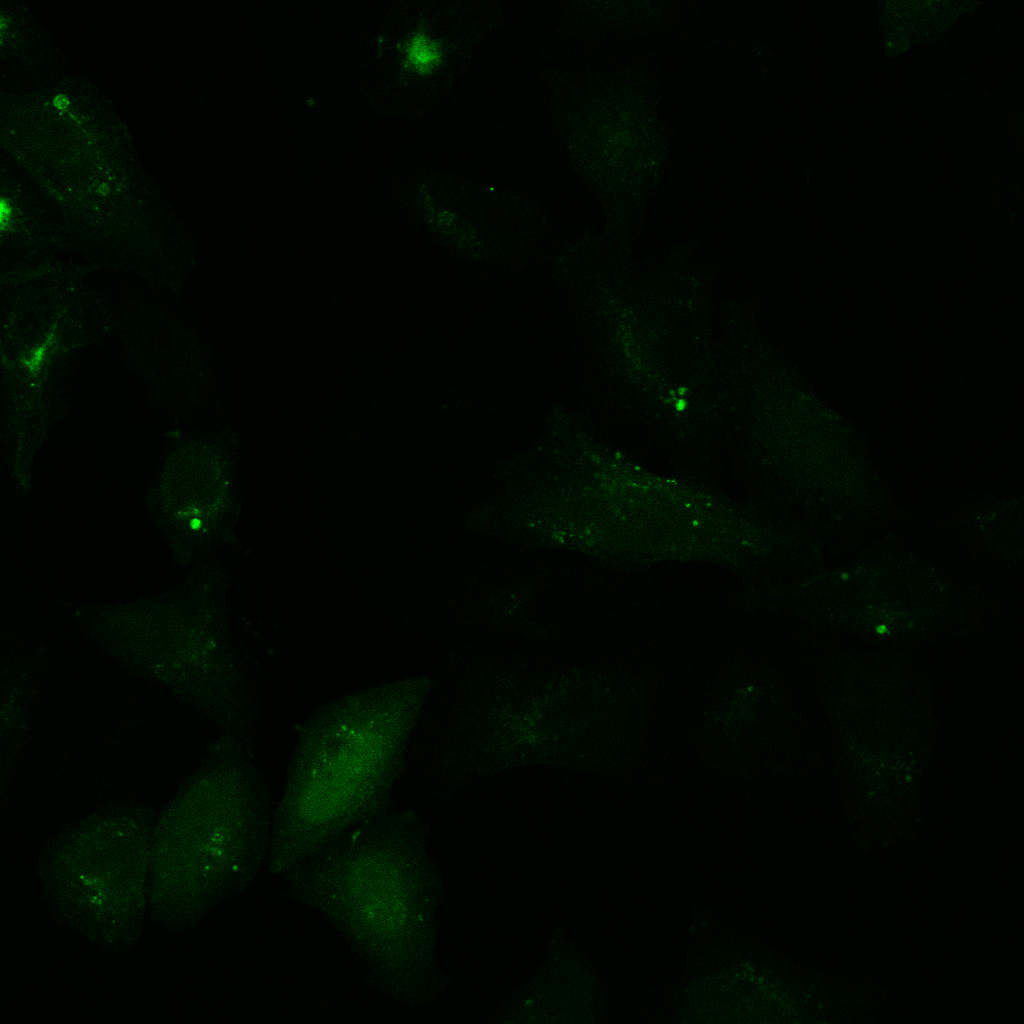

Supplement: Supplementary file 5 — Source data Fig. 3 [file 44318_2024_233_MOESM5_ESM.zip › 3A/Image/HeLa brefeldin a GFP LC3 TGOLN2 RFP_Series001_ch01_SV.tif]

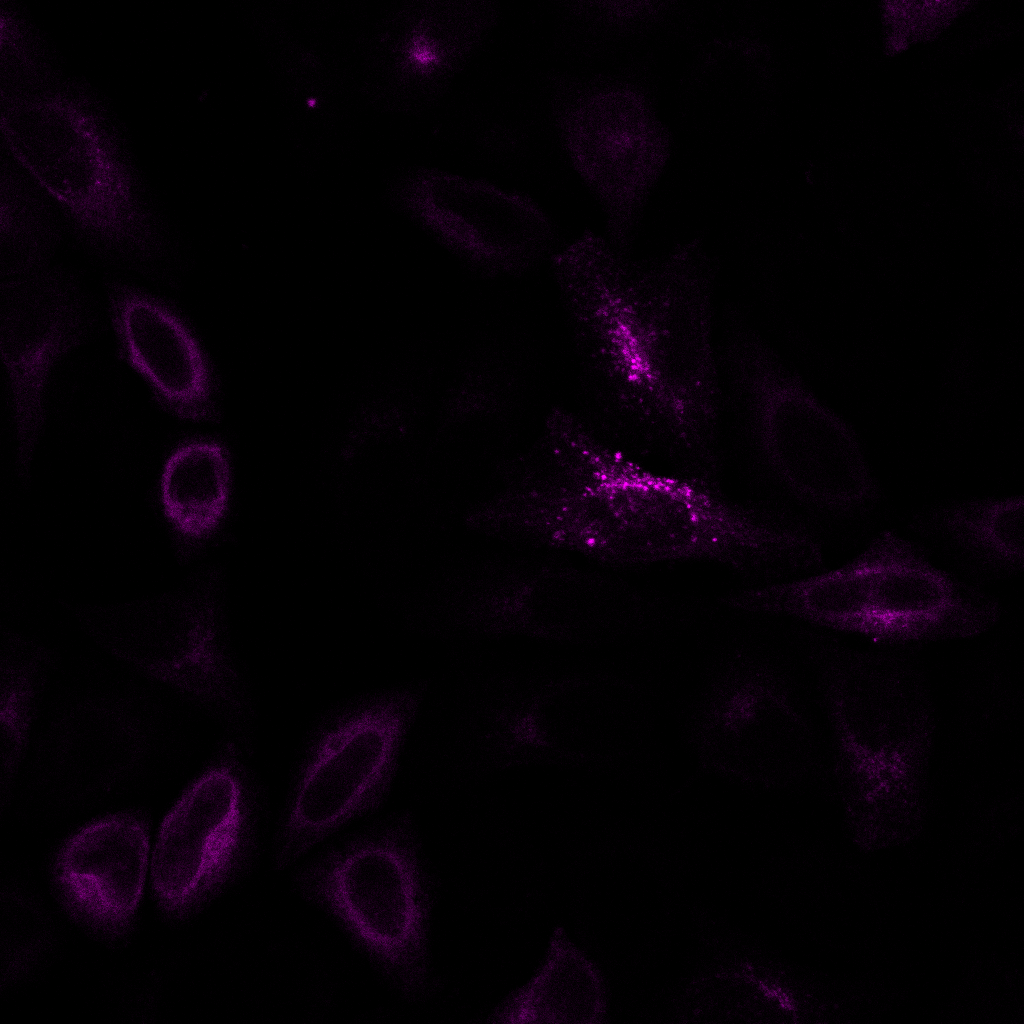

Supplement: Supplementary file 5 — Source data Fig. 3 [file 44318_2024_233_MOESM5_ESM.zip › 3A/Image/HeLa brefeldin a GFP LC3 TGOLN2 RFP_Series001_ch02_SV.tif]

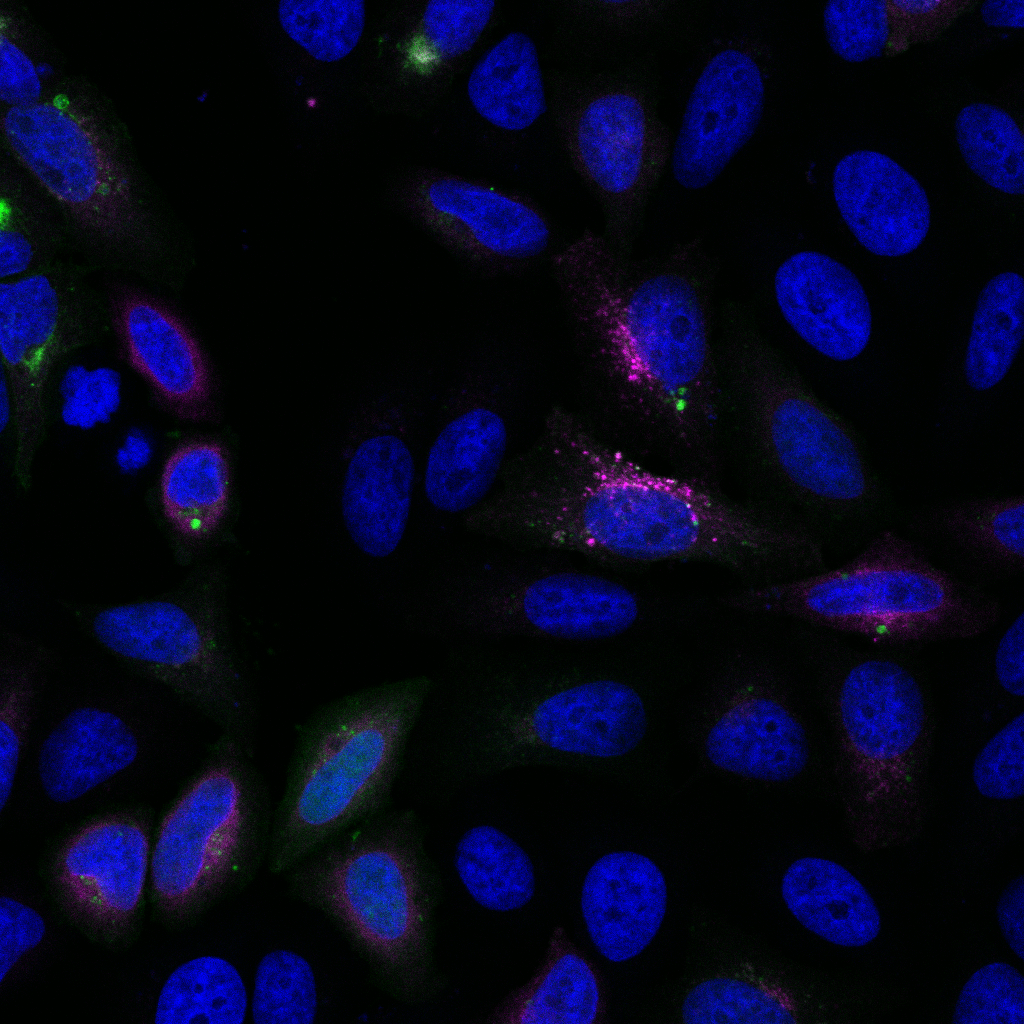

Supplement: Supplementary file 5 — Source data Fig. 3 [file 44318_2024_233_MOESM5_ESM.zip › 3A/Image/HeLa brefeldin a GFP LC3 TGOLN2 RFP_Series001_overlay.tif]

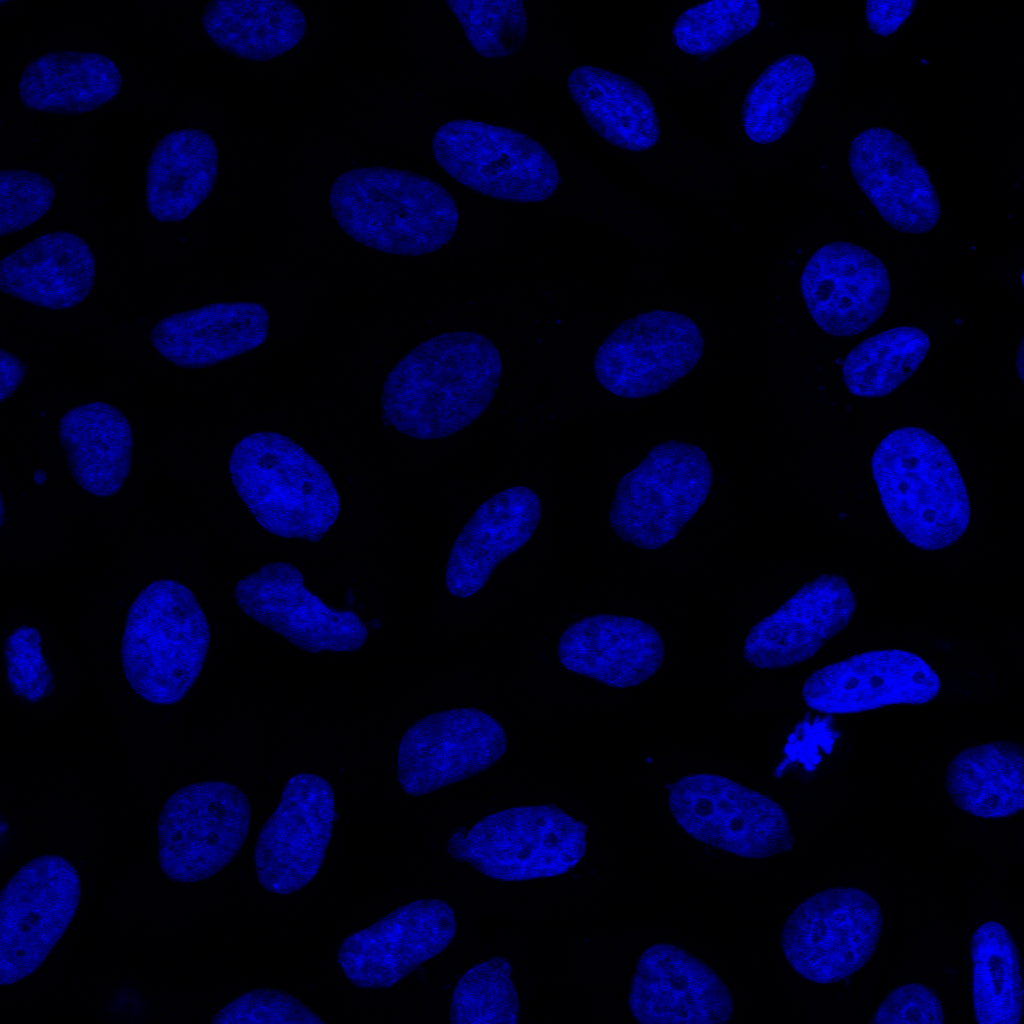

Supplement: Supplementary file 5 — Source data Fig. 3 [file 44318_2024_233_MOESM5_ESM.zip › 3A/Image/HeLa monensin GFP LC3 TGOLN2 RFP_Series002_ch00_SV.tif]

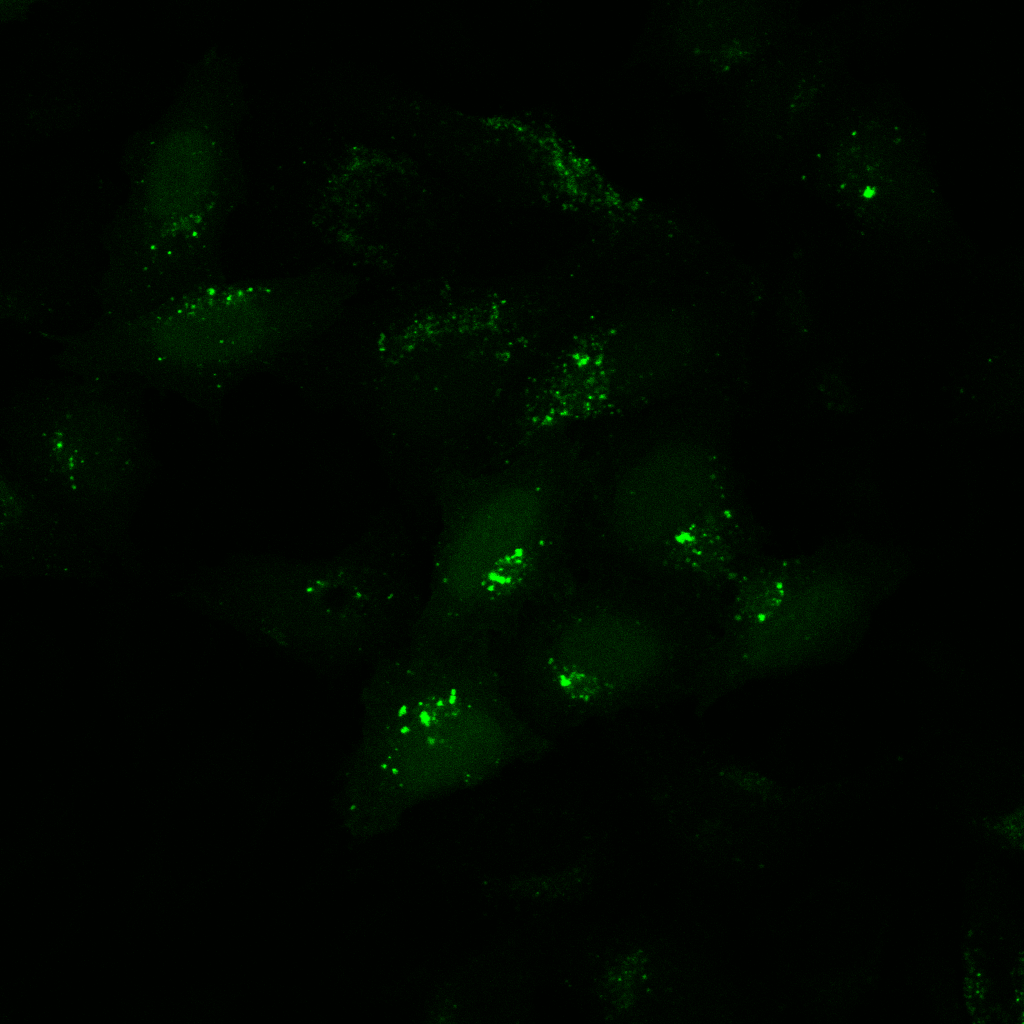

Supplement: Supplementary file 5 — Source data Fig. 3 [file 44318_2024_233_MOESM5_ESM.zip › 3A/Image/HeLa monensin GFP LC3 TGOLN2 RFP_Series002_ch01_SV.tif]

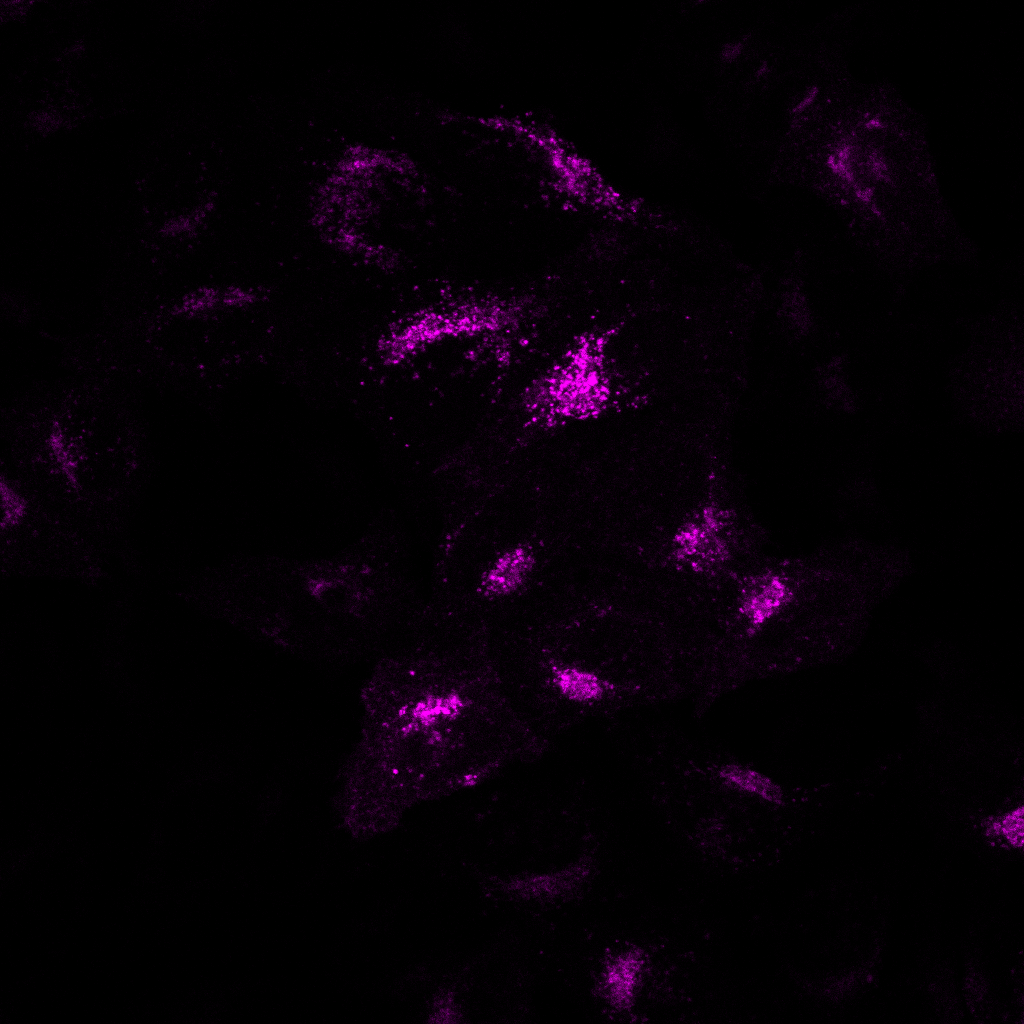

Supplement: Supplementary file 5 — Source data Fig. 3 [file 44318_2024_233_MOESM5_ESM.zip › 3A/Image/HeLa monensin GFP LC3 TGOLN2 RFP_Series002_ch02_SV.tif]

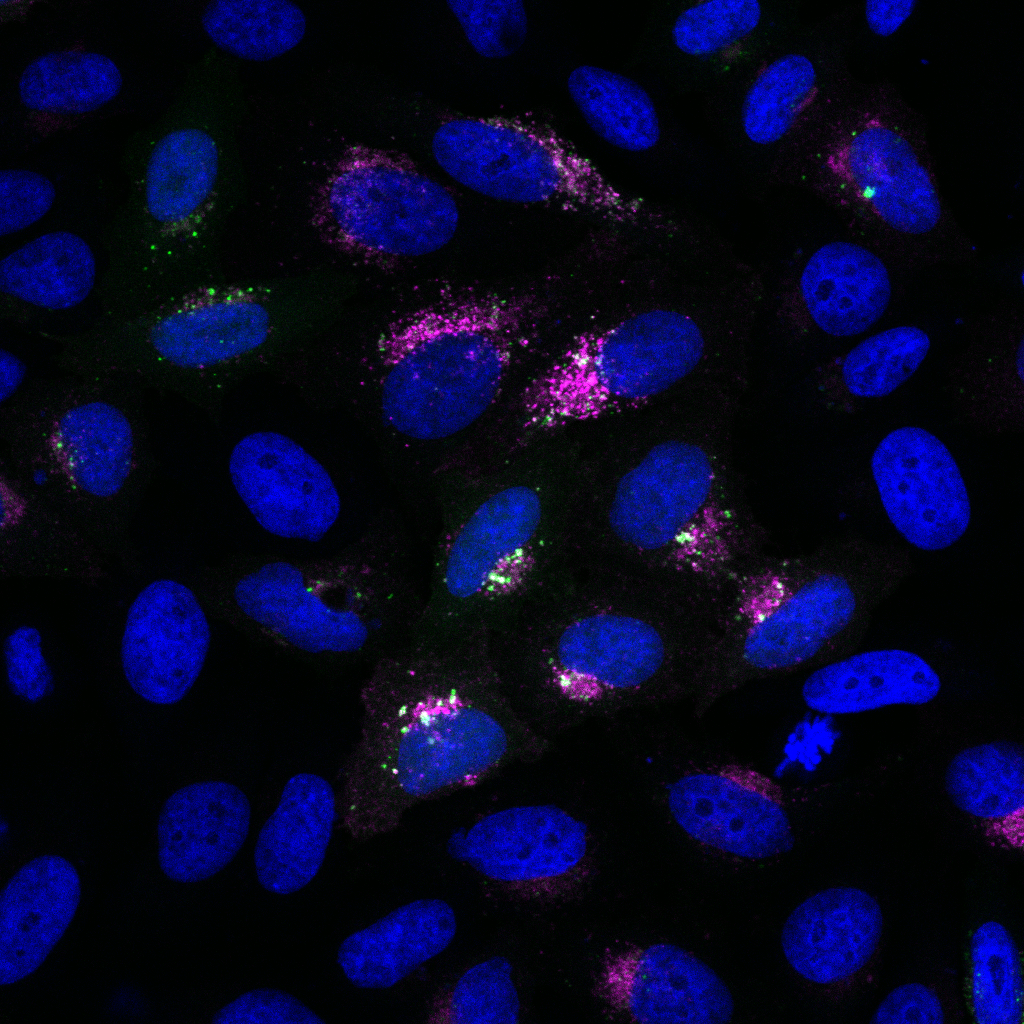

Supplement: Supplementary file 5 — Source data Fig. 3 [file 44318_2024_233_MOESM5_ESM.zip › 3A/Image/HeLa monensin GFP LC3 TGOLN2 RFP_Series002_overlay.tif]

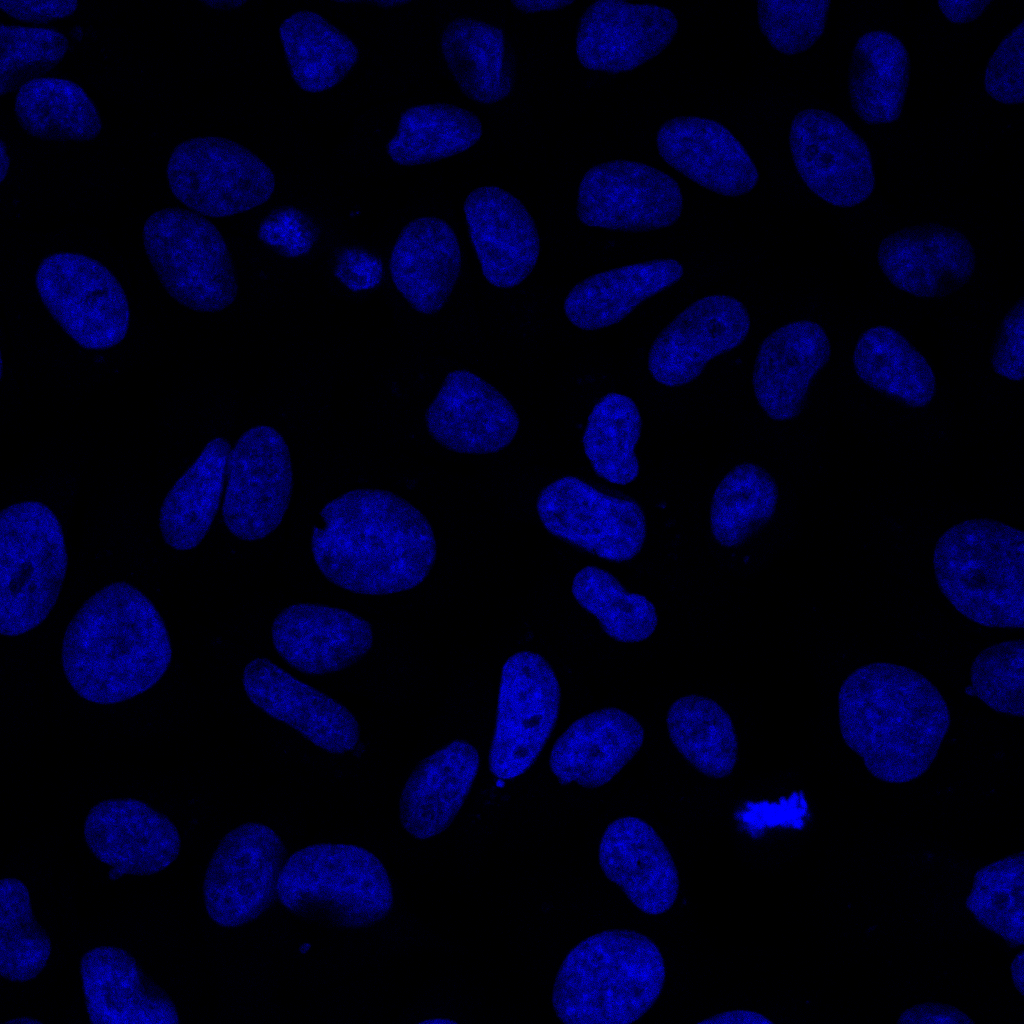

Supplement: Supplementary file 5 — Source data Fig. 3 [file 44318_2024_233_MOESM5_ESM.zip › 3A/Image/HeLa niclosamide GFP LC3 TGOLN2 RFP_Series001_ch00_SV.tif]

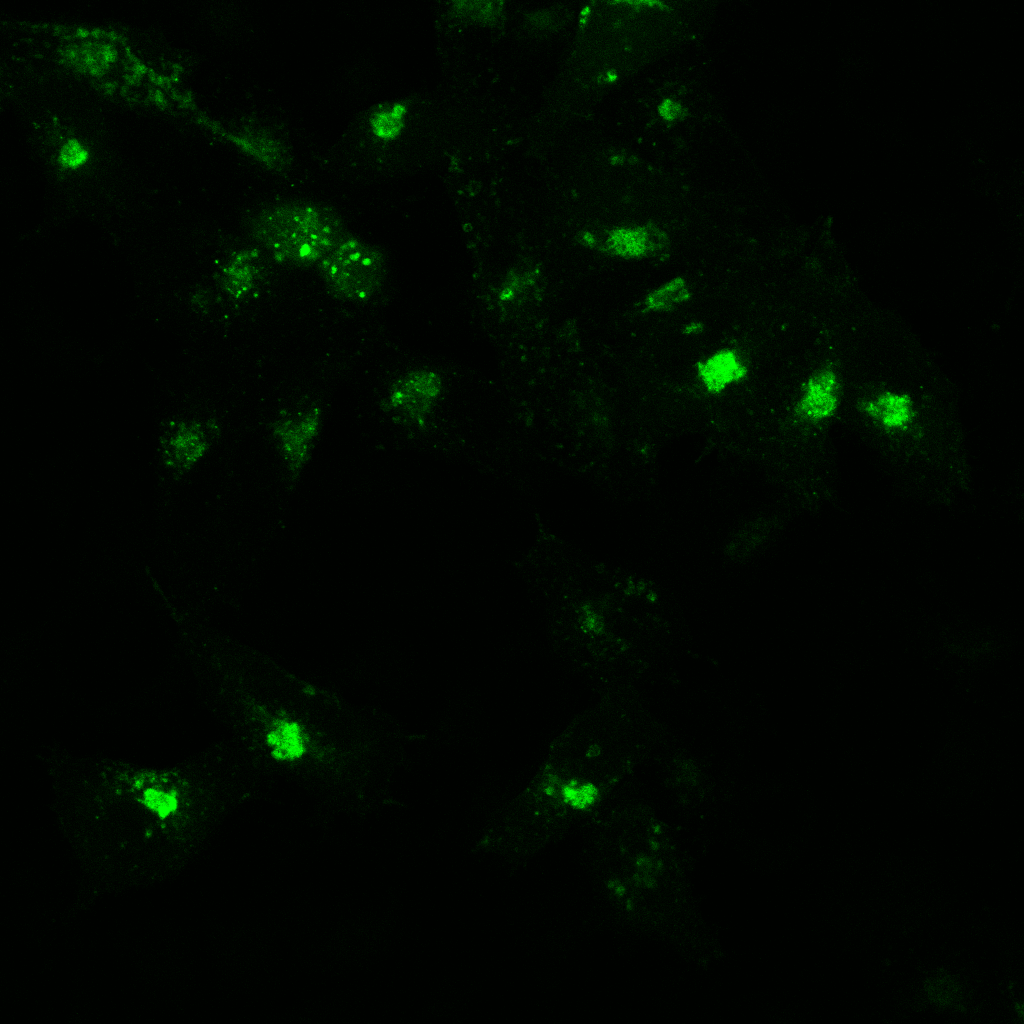

Supplement: Supplementary file 5 — Source data Fig. 3 [file 44318_2024_233_MOESM5_ESM.zip › 3A/Image/HeLa niclosamide GFP LC3 TGOLN2 RFP_Series001_ch01_SV.tif]

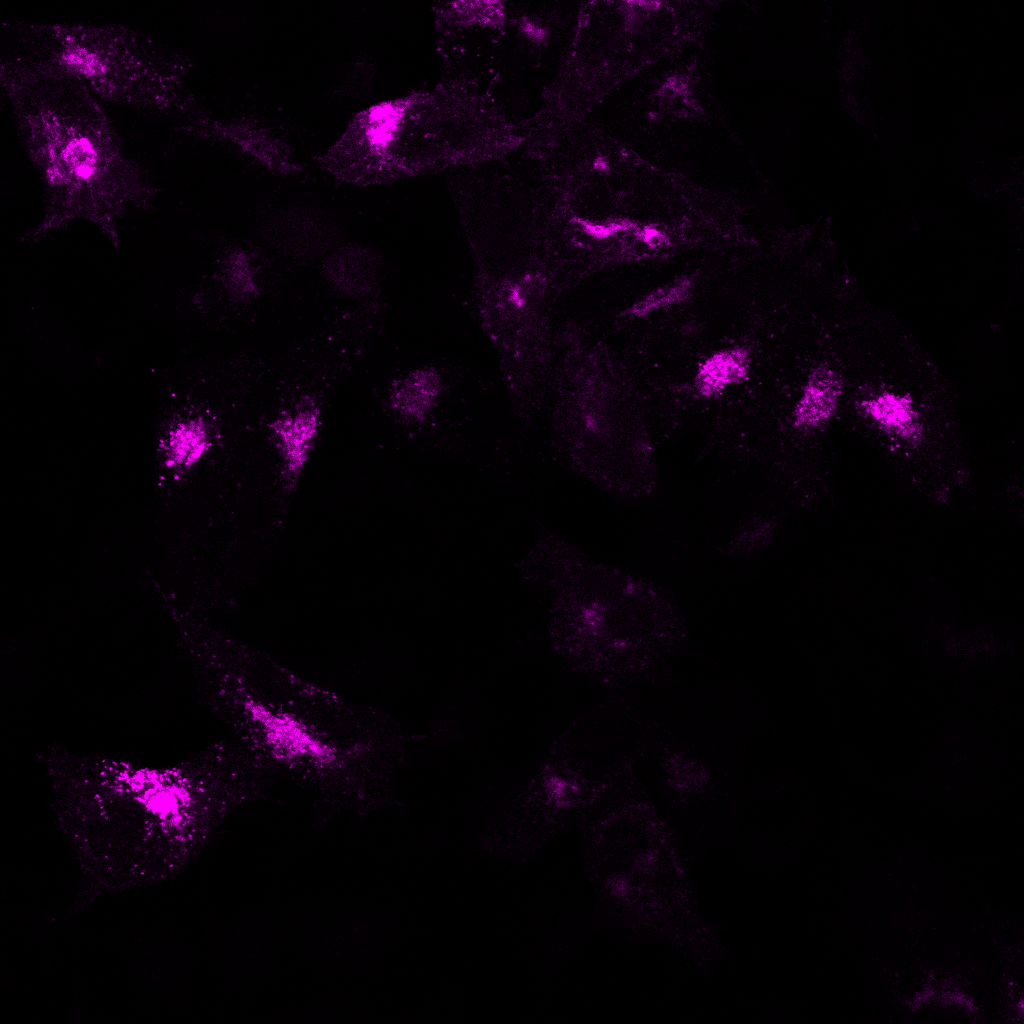

Supplement: Supplementary file 5 — Source data Fig. 3 [file 44318_2024_233_MOESM5_ESM.zip › 3A/Image/HeLa niclosamide GFP LC3 TGOLN2 RFP_Series001_ch02_SV.tif]

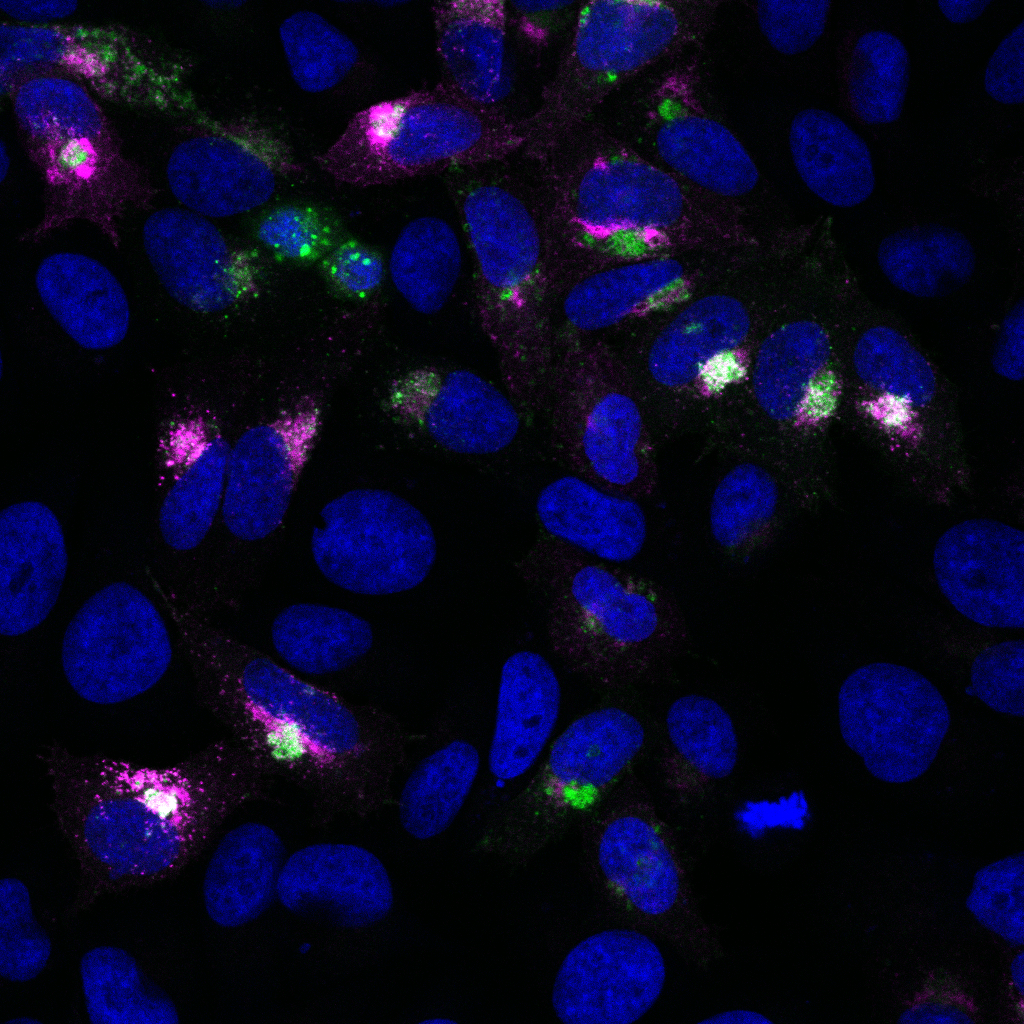

Supplement: Supplementary file 5 — Source data Fig. 3 [file 44318_2024_233_MOESM5_ESM.zip › 3A/Image/HeLa niclosamide GFP LC3 TGOLN2 RFP_Series001_overlay.tif]

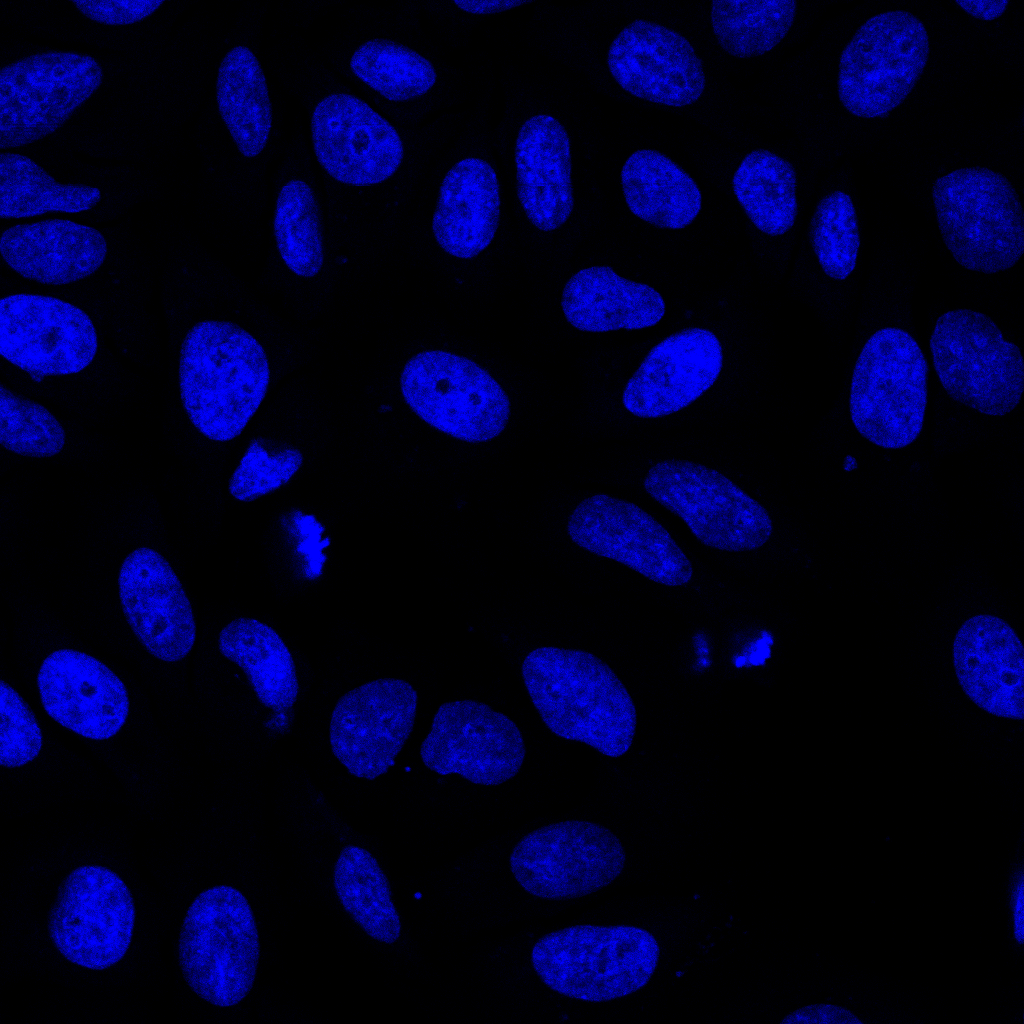

Supplement: Supplementary file 5 — Source data Fig. 3 [file 44318_2024_233_MOESM5_ESM.zip › 3A/Image/HeLa veh GFP LC3 TGOLN2 RFP_Series006_ch00_SV.tif]

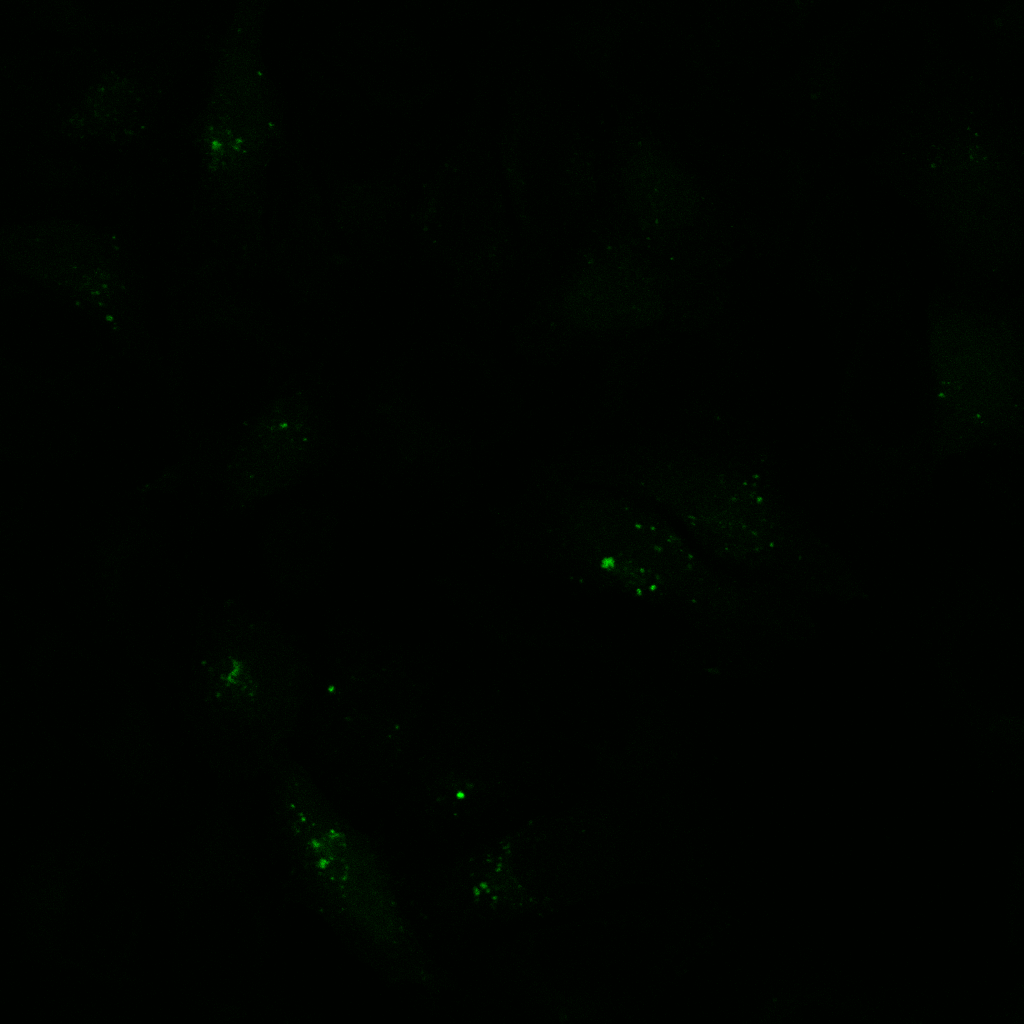

Supplement: Supplementary file 5 — Source data Fig. 3 [file 44318_2024_233_MOESM5_ESM.zip › 3A/Image/HeLa veh GFP LC3 TGOLN2 RFP_Series006_ch01_SV.tif]

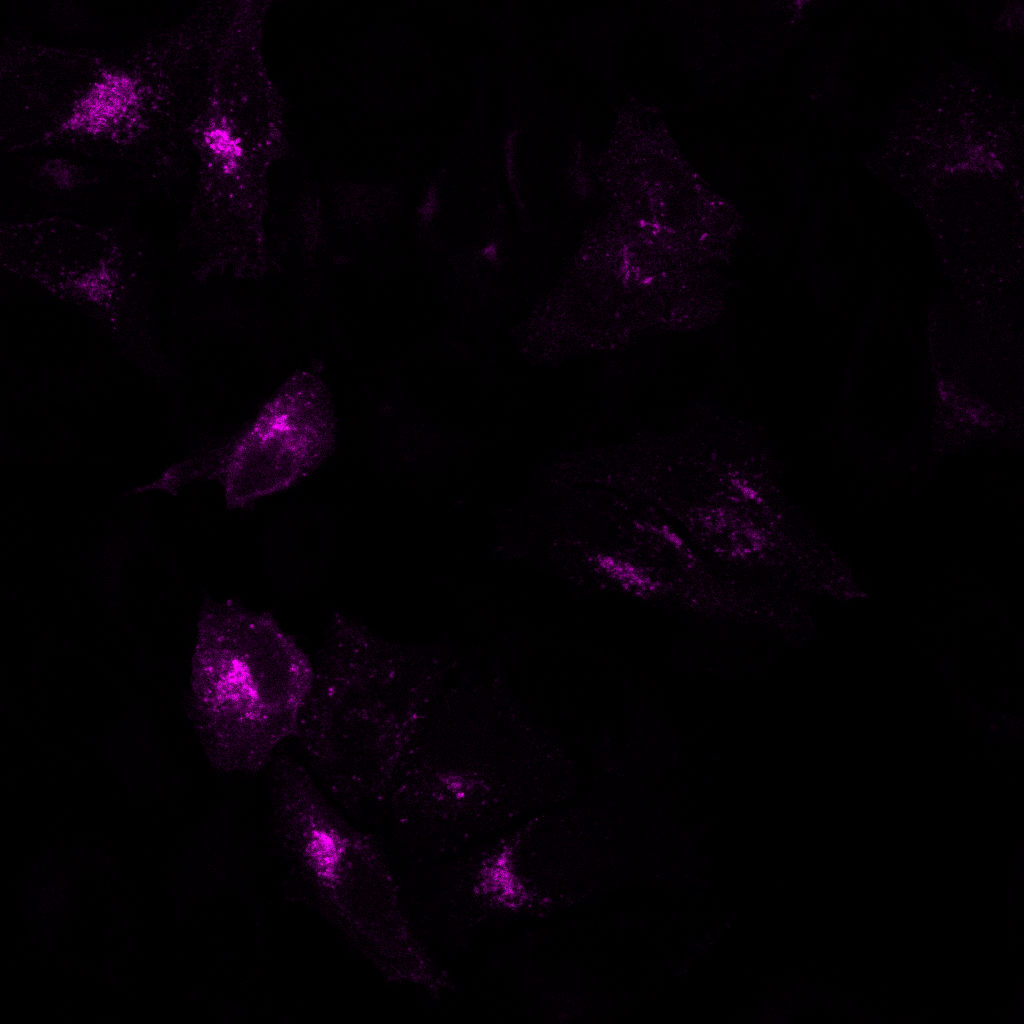

Supplement: Supplementary file 5 — Source data Fig. 3 [file 44318_2024_233_MOESM5_ESM.zip › 3A/Image/HeLa veh GFP LC3 TGOLN2 RFP_Series006_ch02_SV.tif]

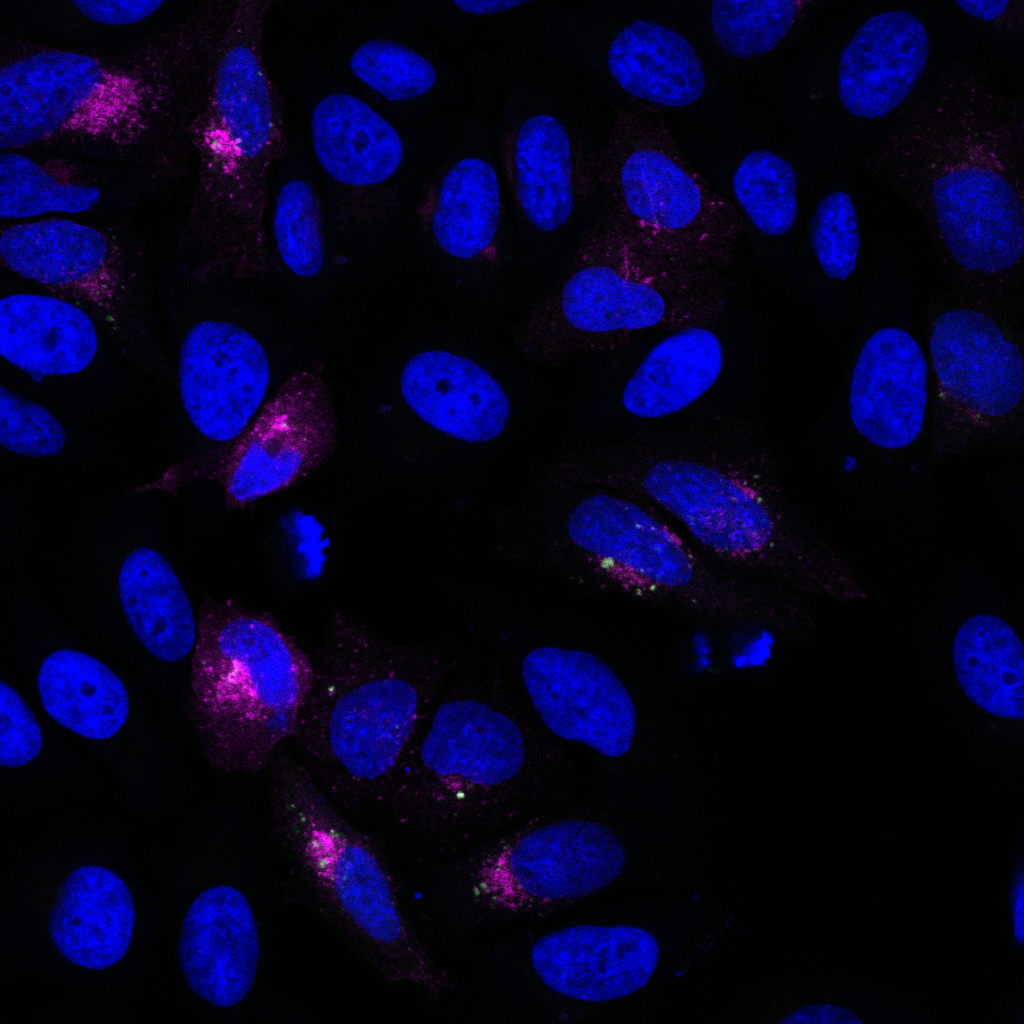

Supplement: Supplementary file 5 — Source data Fig. 3 [file 44318_2024_233_MOESM5_ESM.zip › 3A/Image/HeLa veh GFP LC3 TGOLN2 RFP_Series006_overlay.tif]

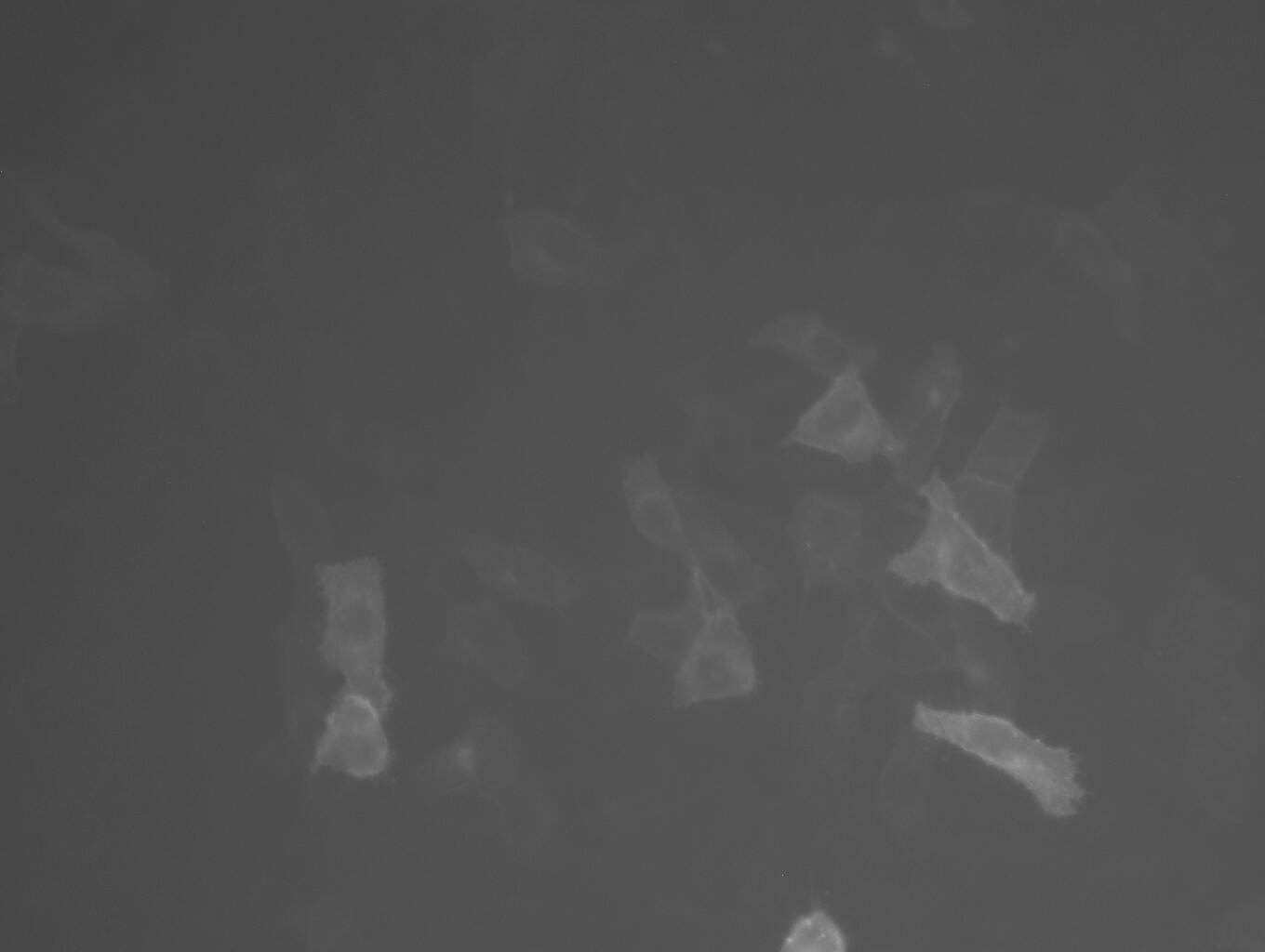

Supplement: Supplementary file 5 — Source data Fig. 3 [file 44318_2024_233_MOESM5_ESM.zip › 3B/Ctrl Biotin (+) GFP.jpg]

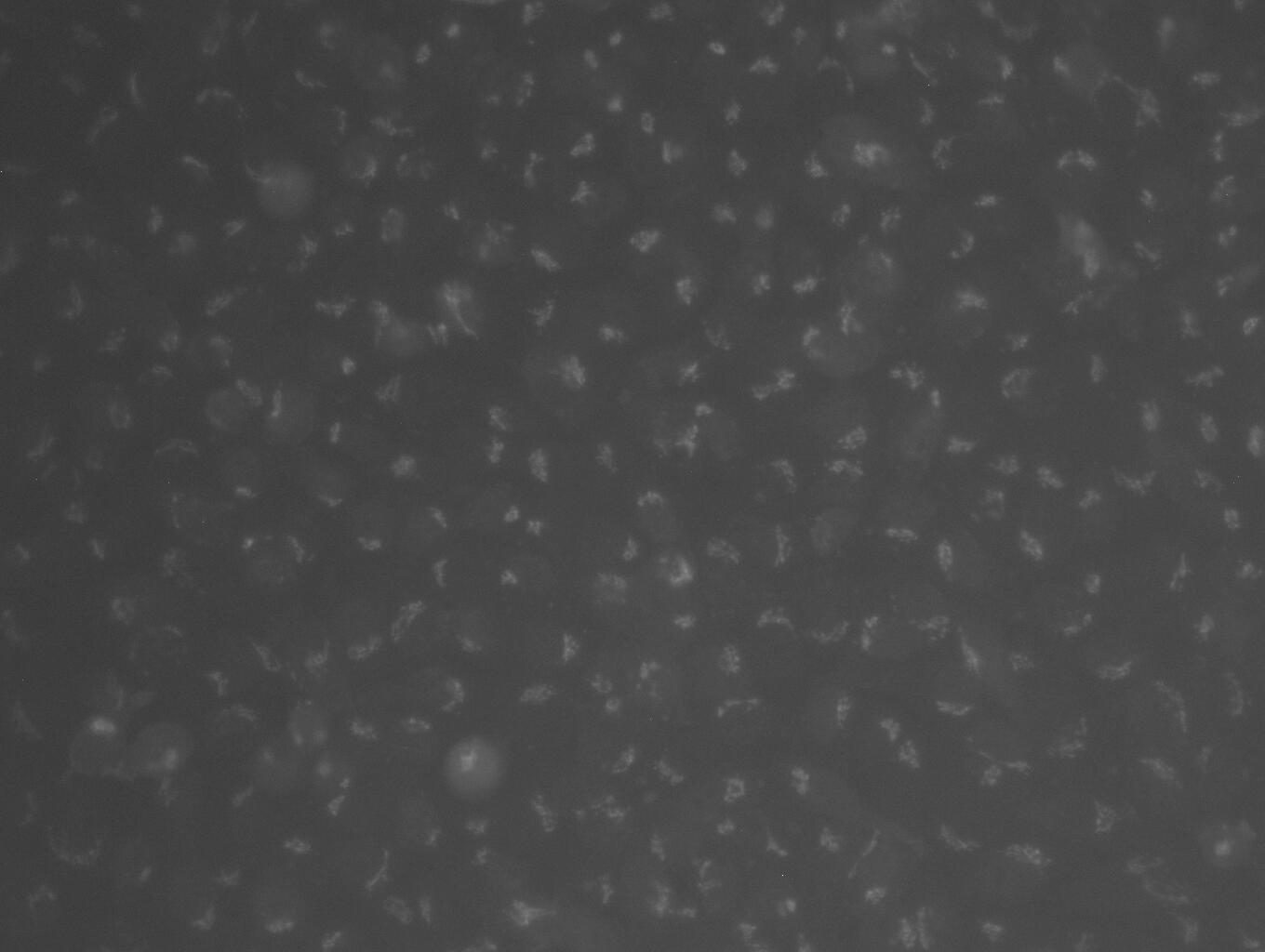

Supplement: Supplementary file 5 — Source data Fig. 3 [file 44318_2024_233_MOESM5_ESM.zip › 3B/Ctrl Biotin (+) Golgin-97.jpg]

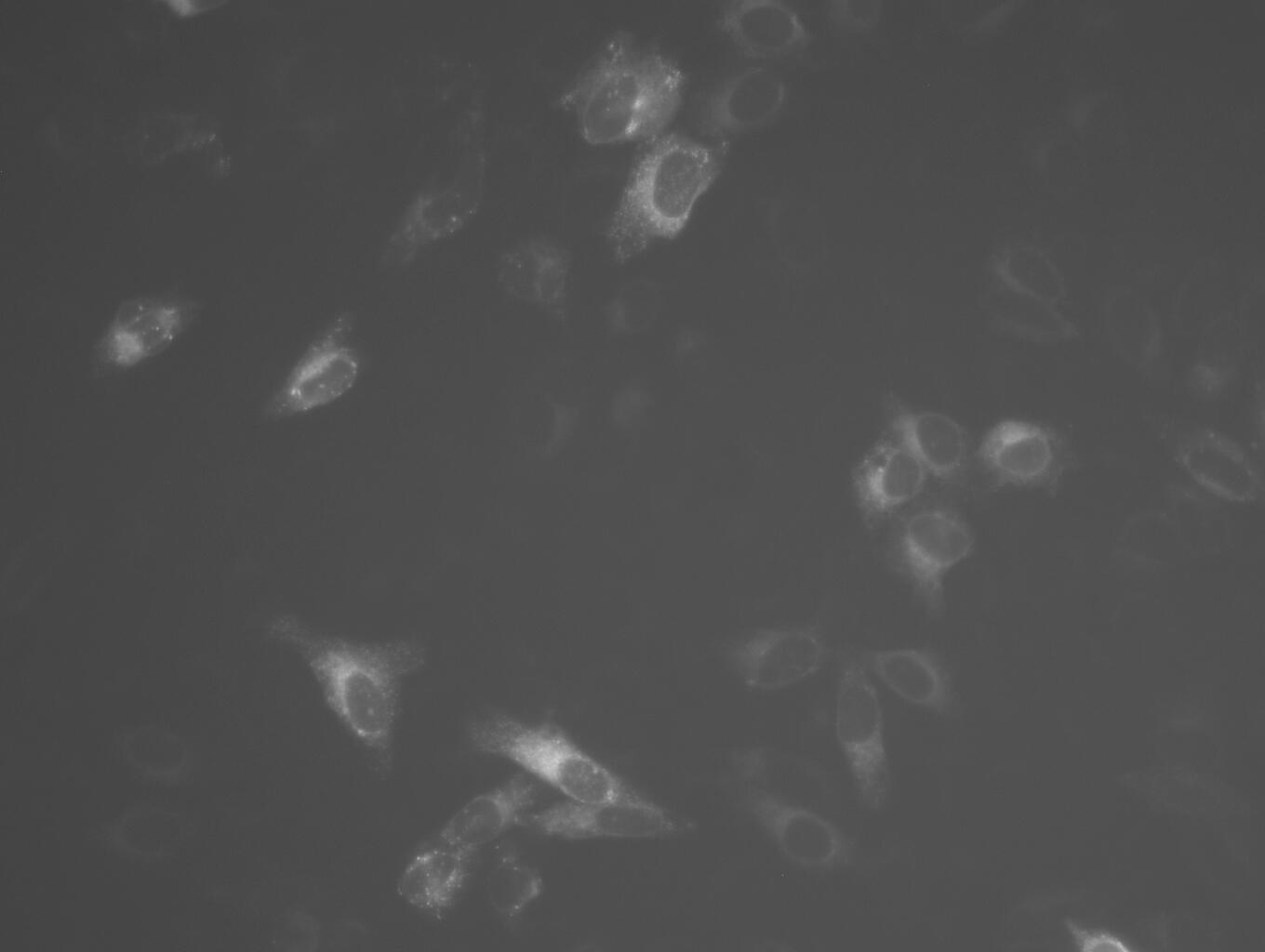

Supplement: Supplementary file 5 — Source data Fig. 3 [file 44318_2024_233_MOESM5_ESM.zip › 3B/Ctrl Biotin (-) GFP.jpg]

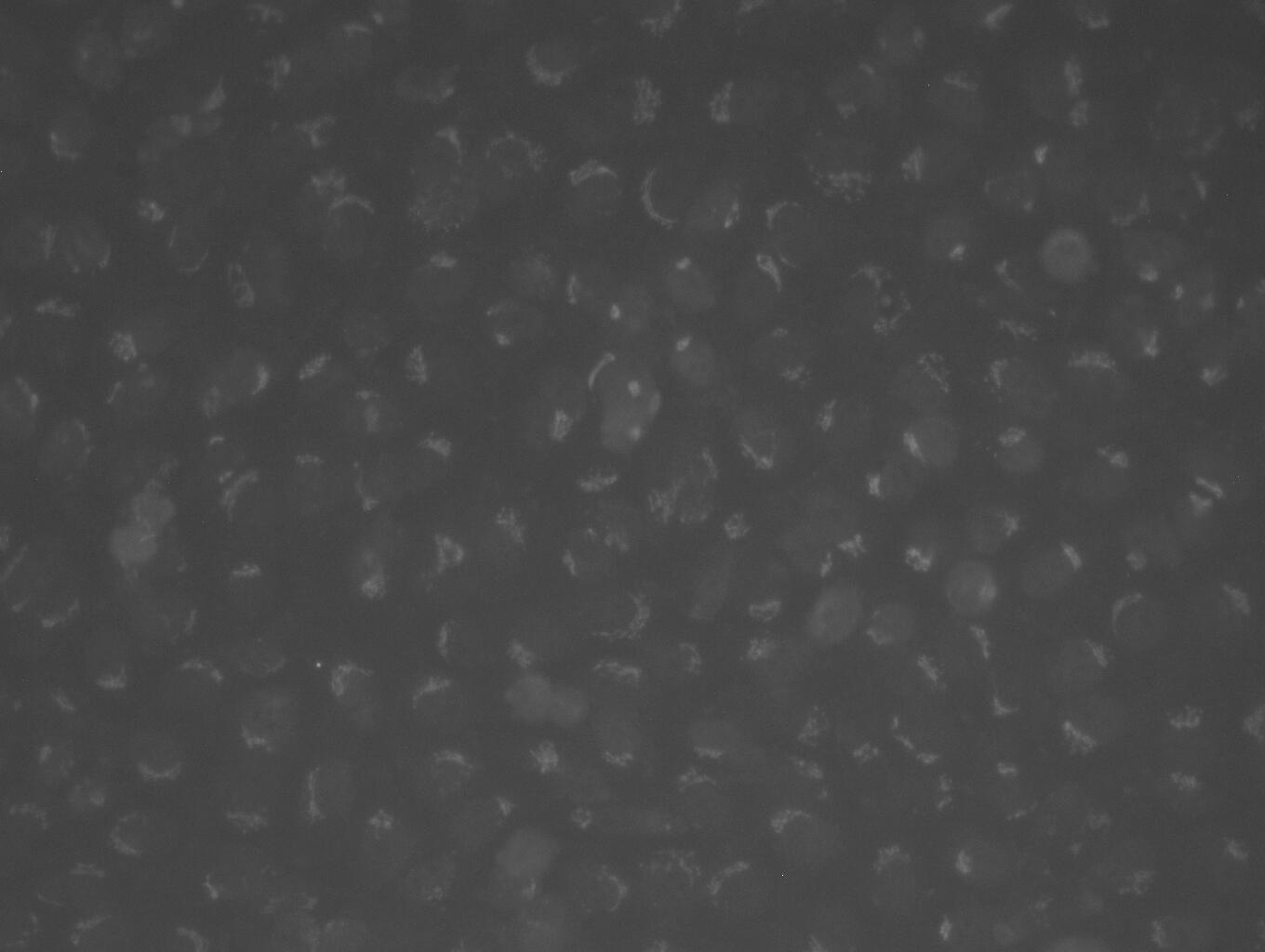

Supplement: Supplementary file 5 — Source data Fig. 3 [file 44318_2024_233_MOESM5_ESM.zip › 3B/Ctrl Biotin (-) Golgin-97.jpg]

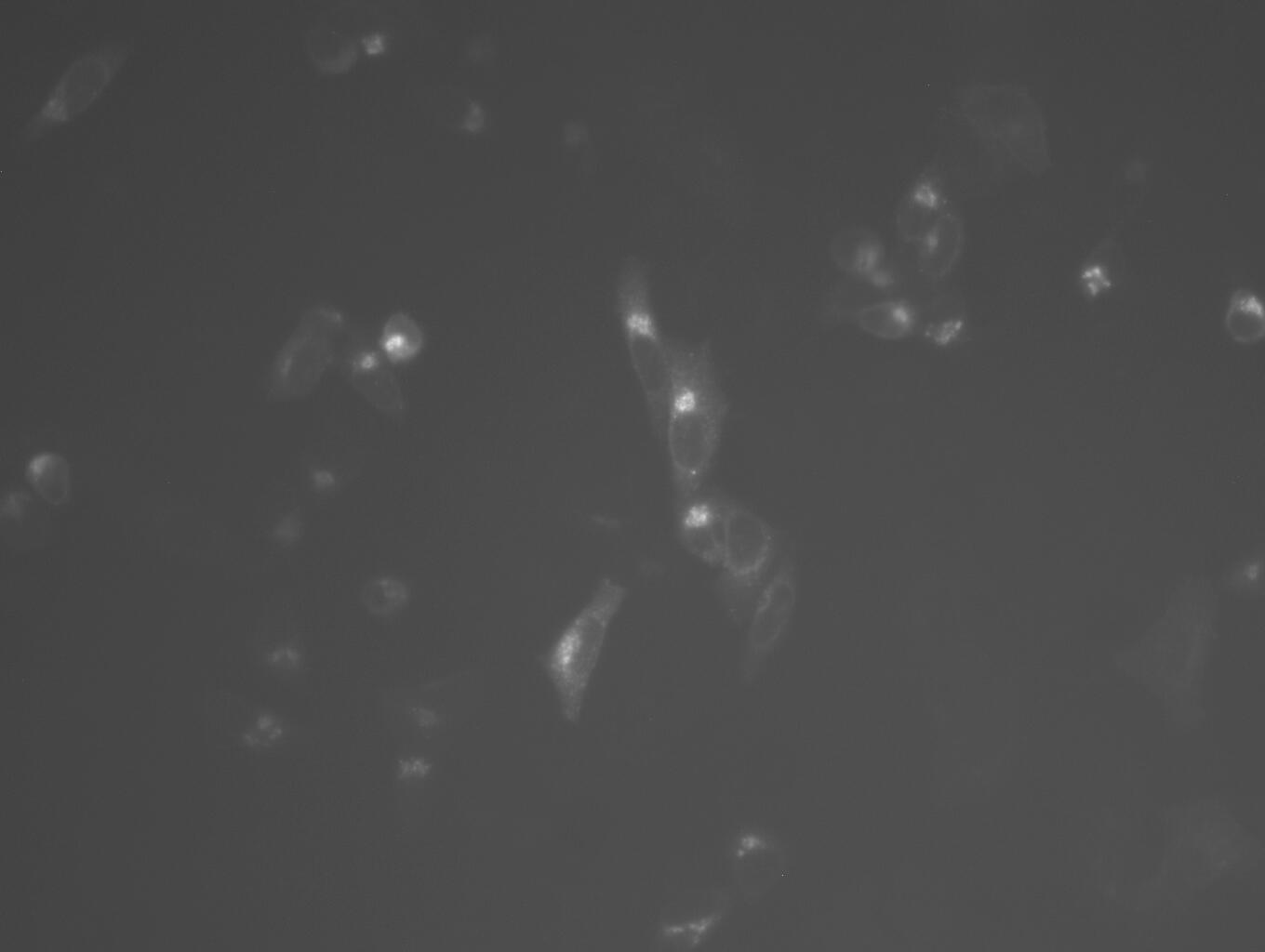

Supplement: Supplementary file 5 — Source data Fig. 3 [file 44318_2024_233_MOESM5_ESM.zip › 3B/DLK1 Biotin (+) GFP.jpg]

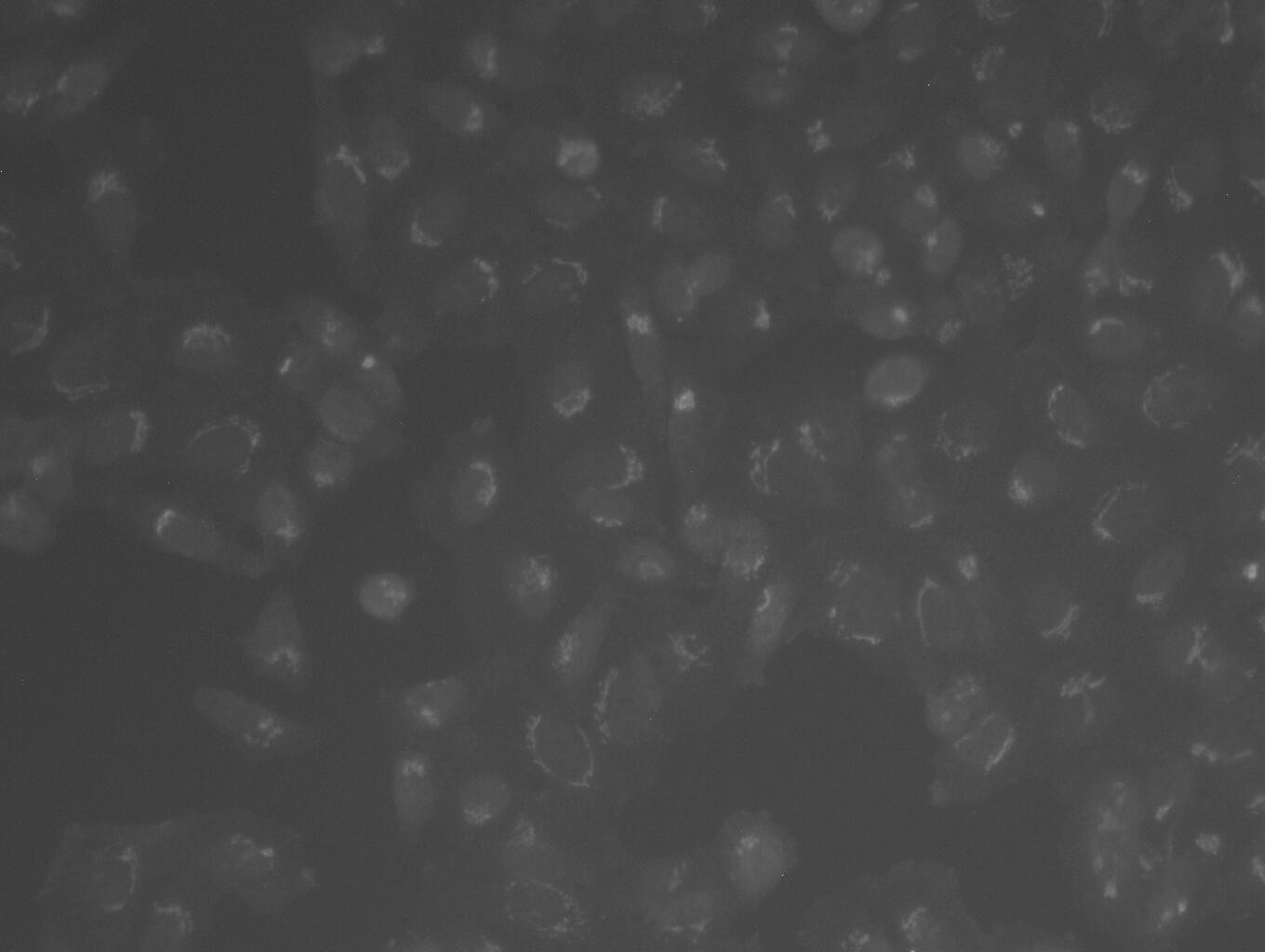

Supplement: Supplementary file 5 — Source data Fig. 3 [file 44318_2024_233_MOESM5_ESM.zip › 3B/DLK1 Biotin (+) Golgin-97.jpg]

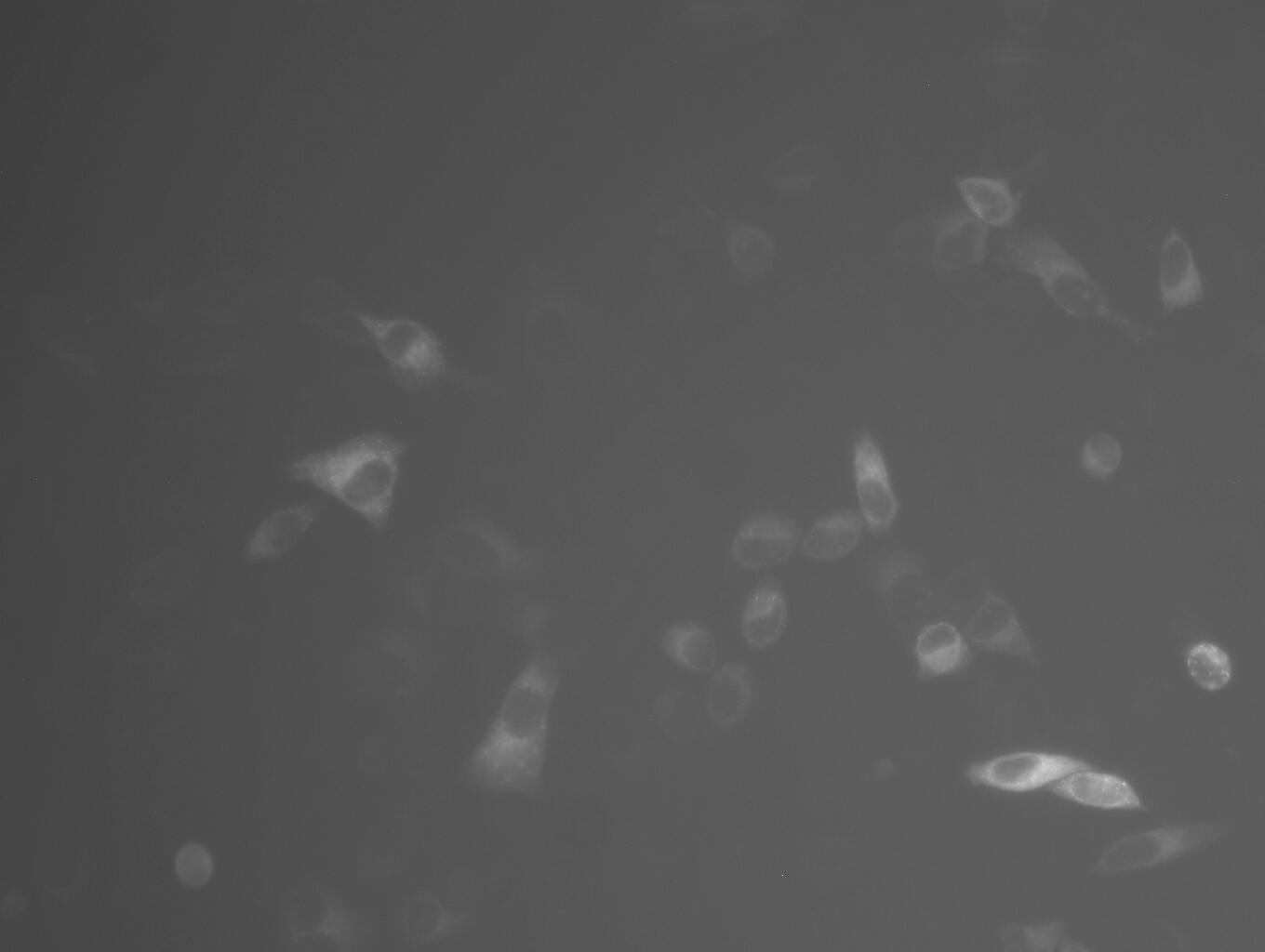

Supplement: Supplementary file 5 — Source data Fig. 3 [file 44318_2024_233_MOESM5_ESM.zip › 3B/DLK1 Biotin (-) GFP.jpg]

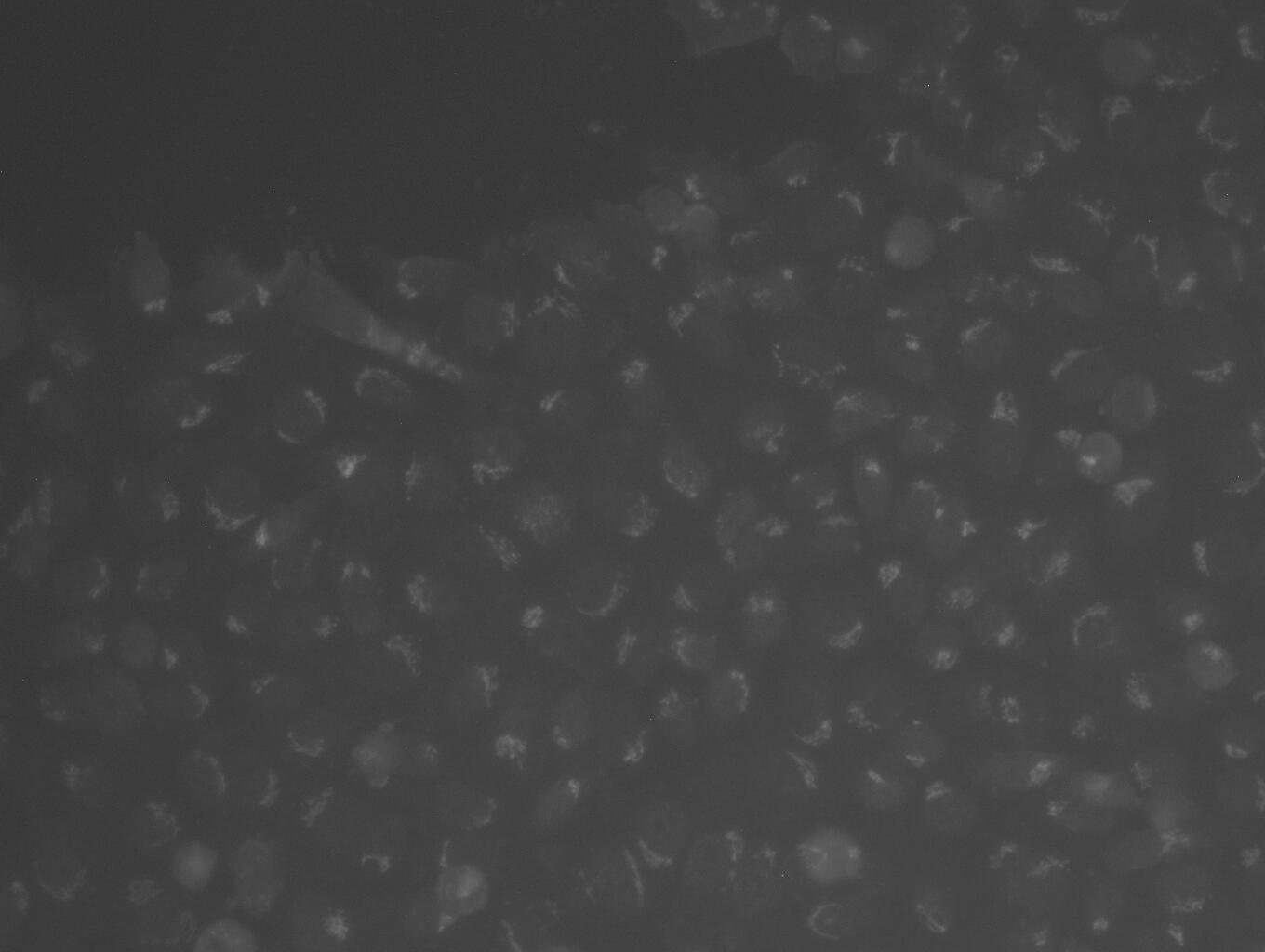

Supplement: Supplementary file 5 — Source data Fig. 3 [file 44318_2024_233_MOESM5_ESM.zip › 3B/DLK1 Biotin (-) Golgin-97.jpg]
