# Supplementary figures and images for: Non-autophagic Golgi-LC3 lipidation facilitates TFE3 stress response against Golgi dysfunction (part 2 of 3)
Source: EMBO J. 2024 Sep 16;43(21):5085–113. doi: 10.1038/s44318-024-00233-y (PMC11535212; doi:10.1038/s44318-024-00233-y)

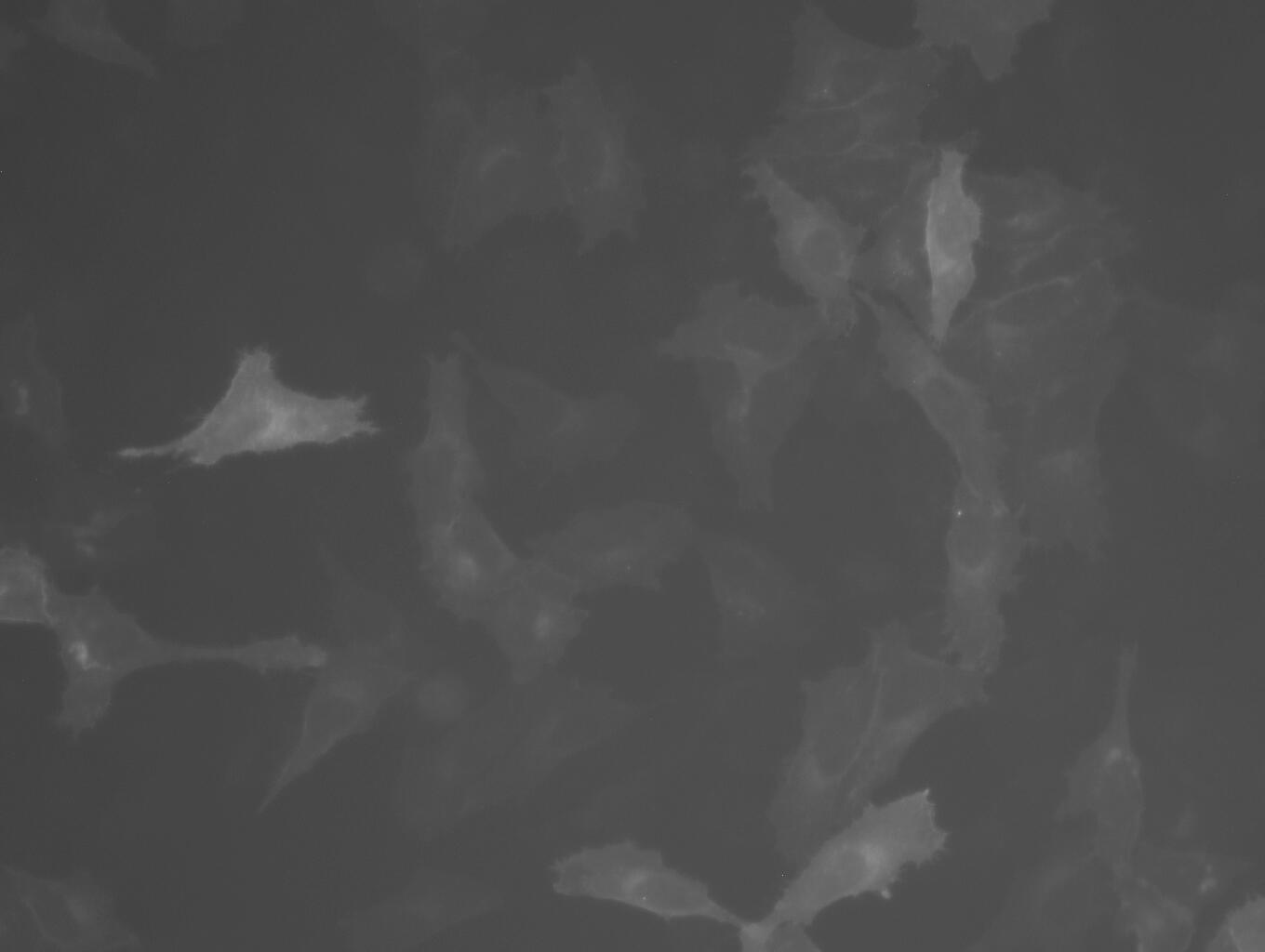

Supplement: Supplementary file 5 — Source data Fig. 3 [file 44318_2024_233_MOESM5_ESM.zip › 3C/1. Veh biotin (+) GFP.jpg]

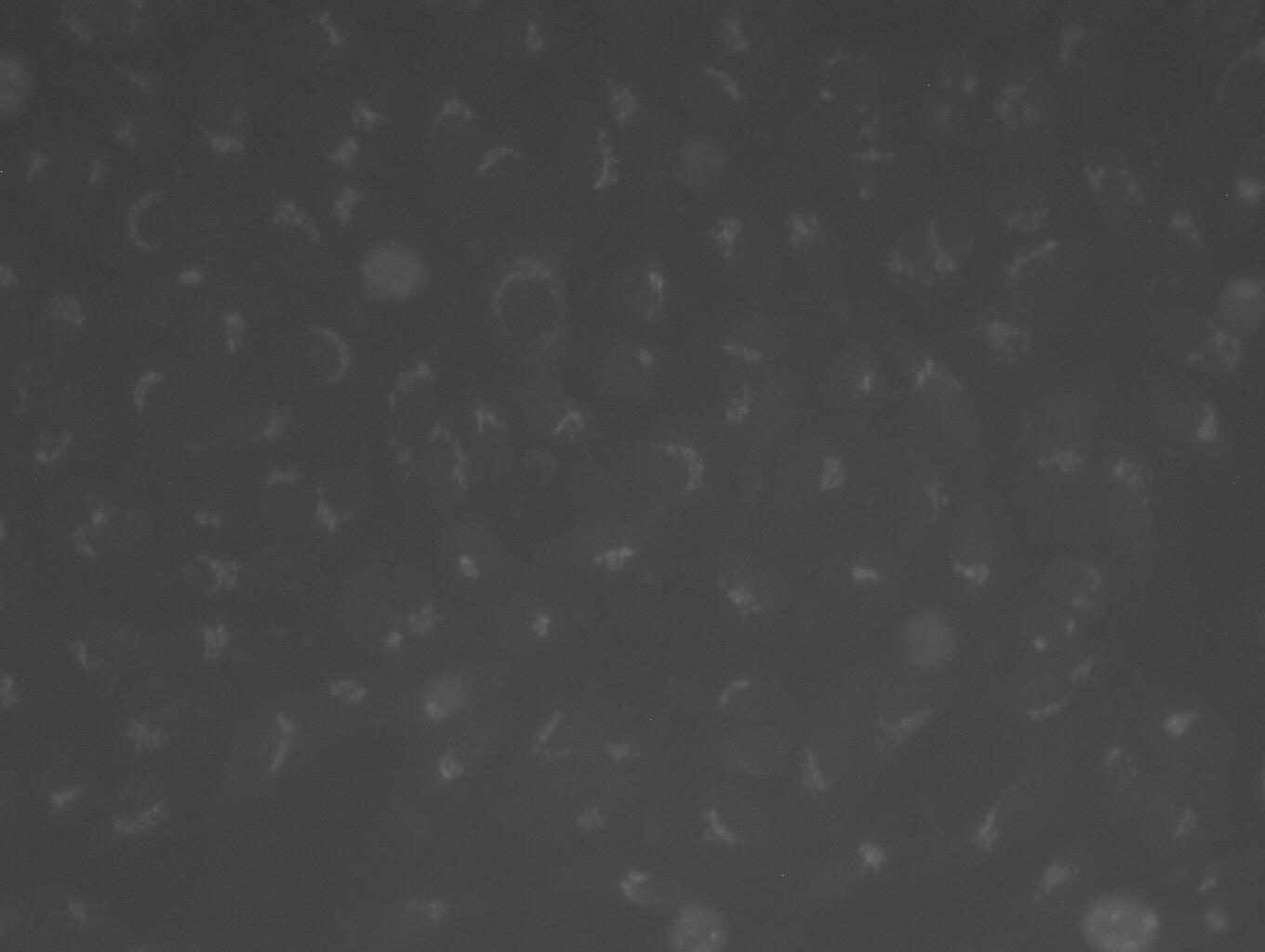

Supplement: Supplementary file 5 — Source data Fig. 3 [file 44318_2024_233_MOESM5_ESM.zip › 3C/1. Veh biotin (+) Golgin-97.jpg]

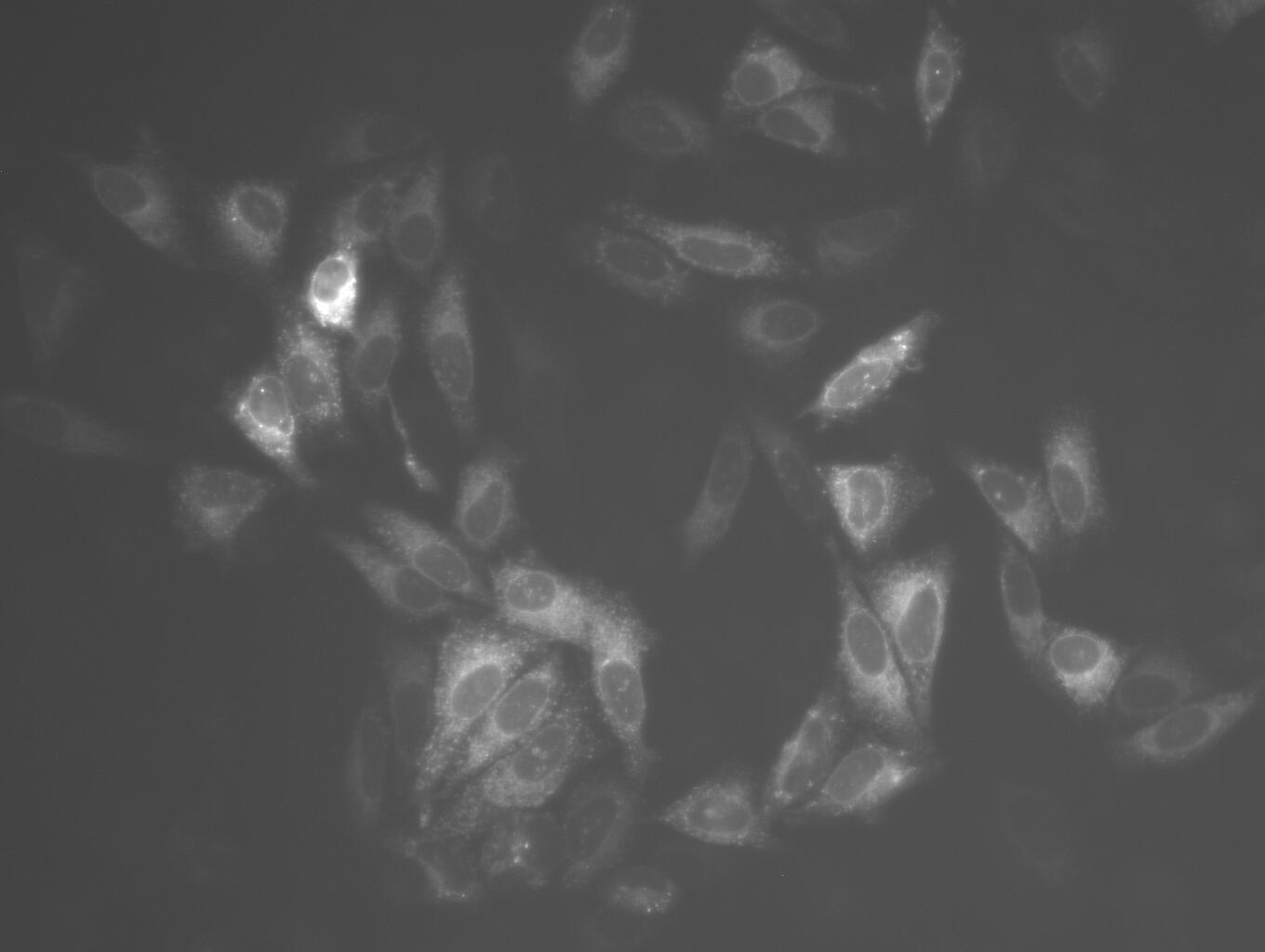

Supplement: Supplementary file 5 — Source data Fig. 3 [file 44318_2024_233_MOESM5_ESM.zip › 3C/1. Veh biotin (-) GFP.jpg]

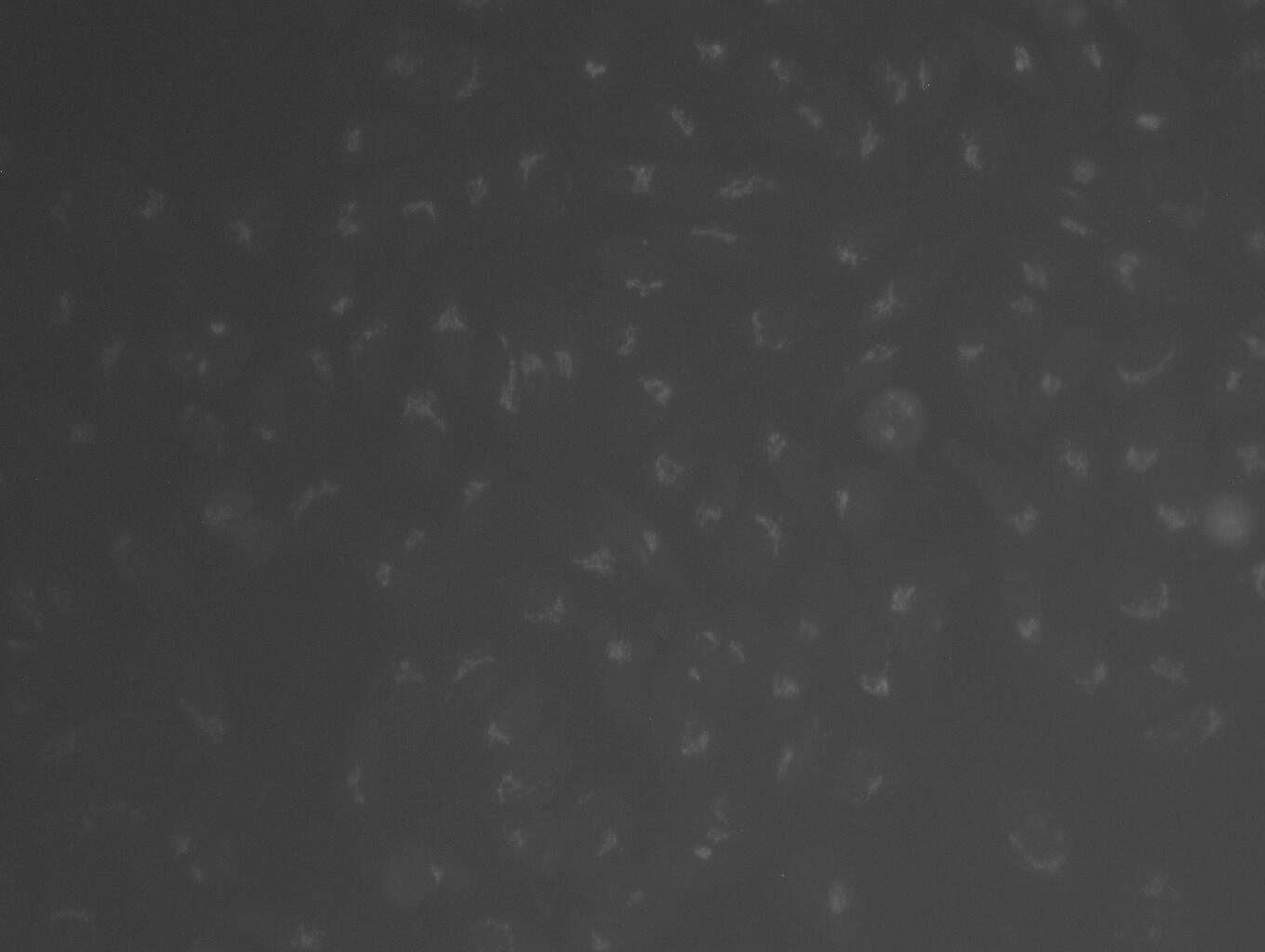

Supplement: Supplementary file 5 — Source data Fig. 3 [file 44318_2024_233_MOESM5_ESM.zip › 3C/1. Veh biotin (-) Golgin-97.jpg]

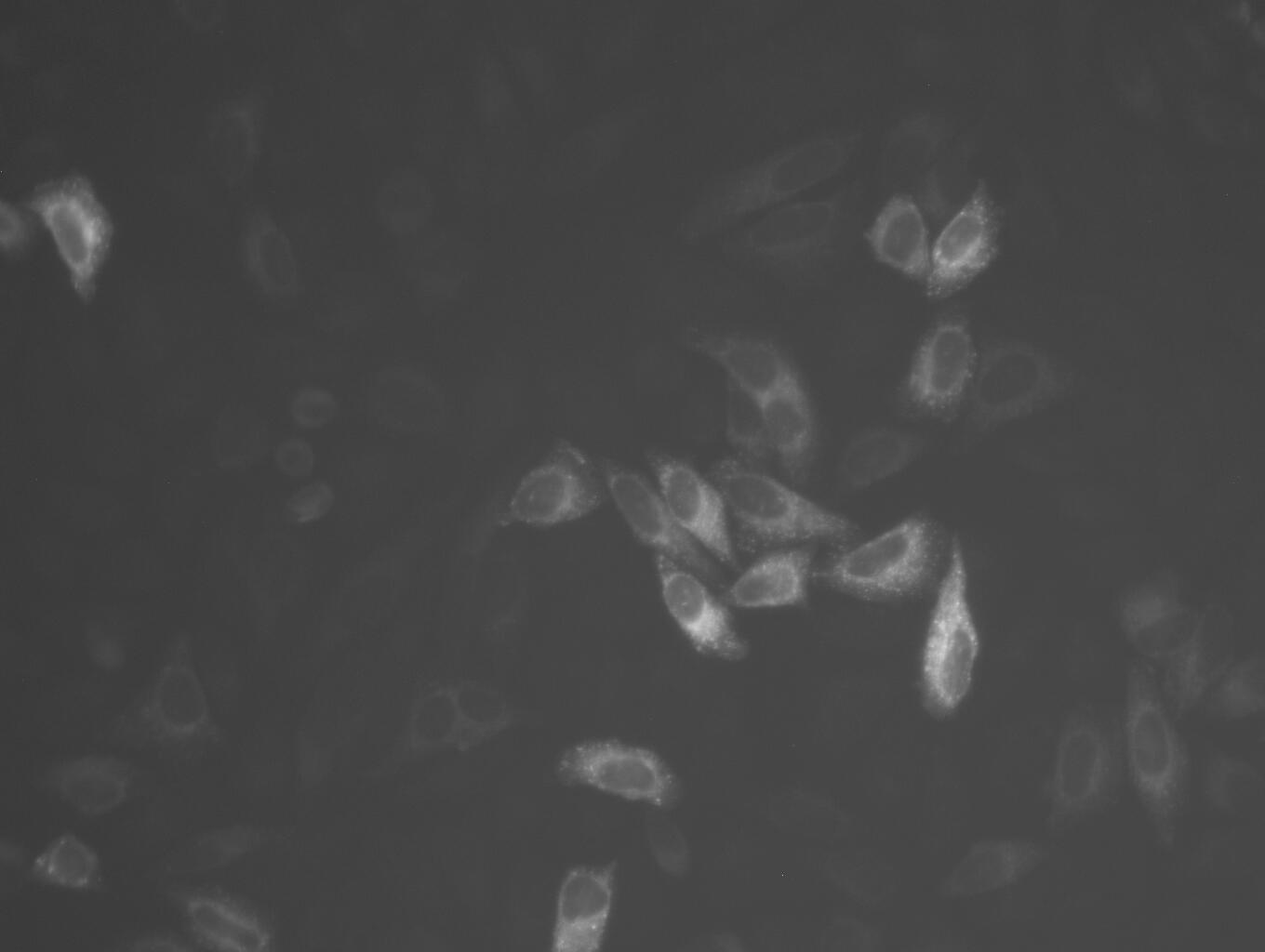

Supplement: Supplementary file 5 — Source data Fig. 3 [file 44318_2024_233_MOESM5_ESM.zip › 3C/2. brefeldin a biotin (+) GFP.jpg]

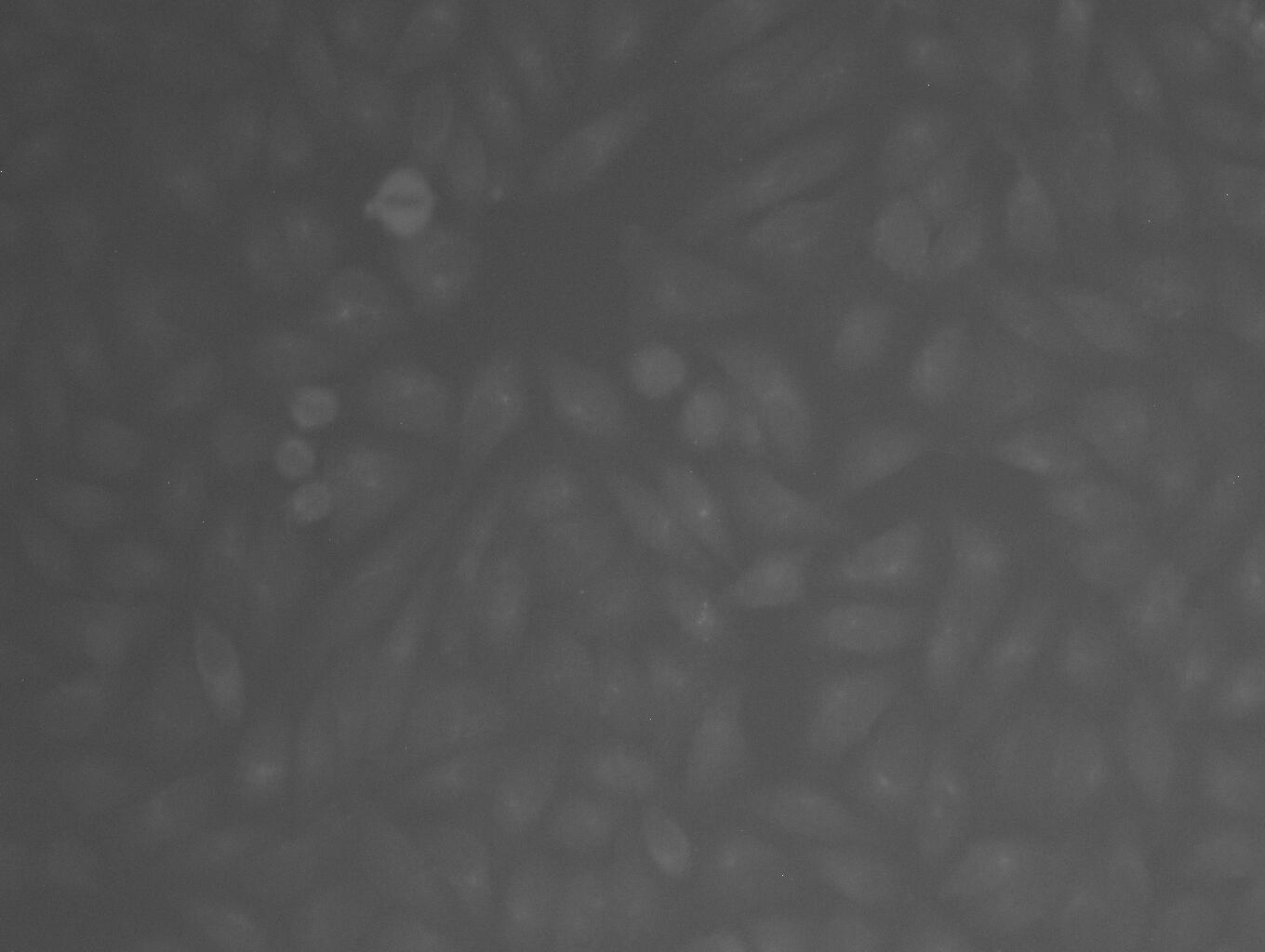

Supplement: Supplementary file 5 — Source data Fig. 3 [file 44318_2024_233_MOESM5_ESM.zip › 3C/2. brefeldin a biotin (+) Golgin-97.jpg]

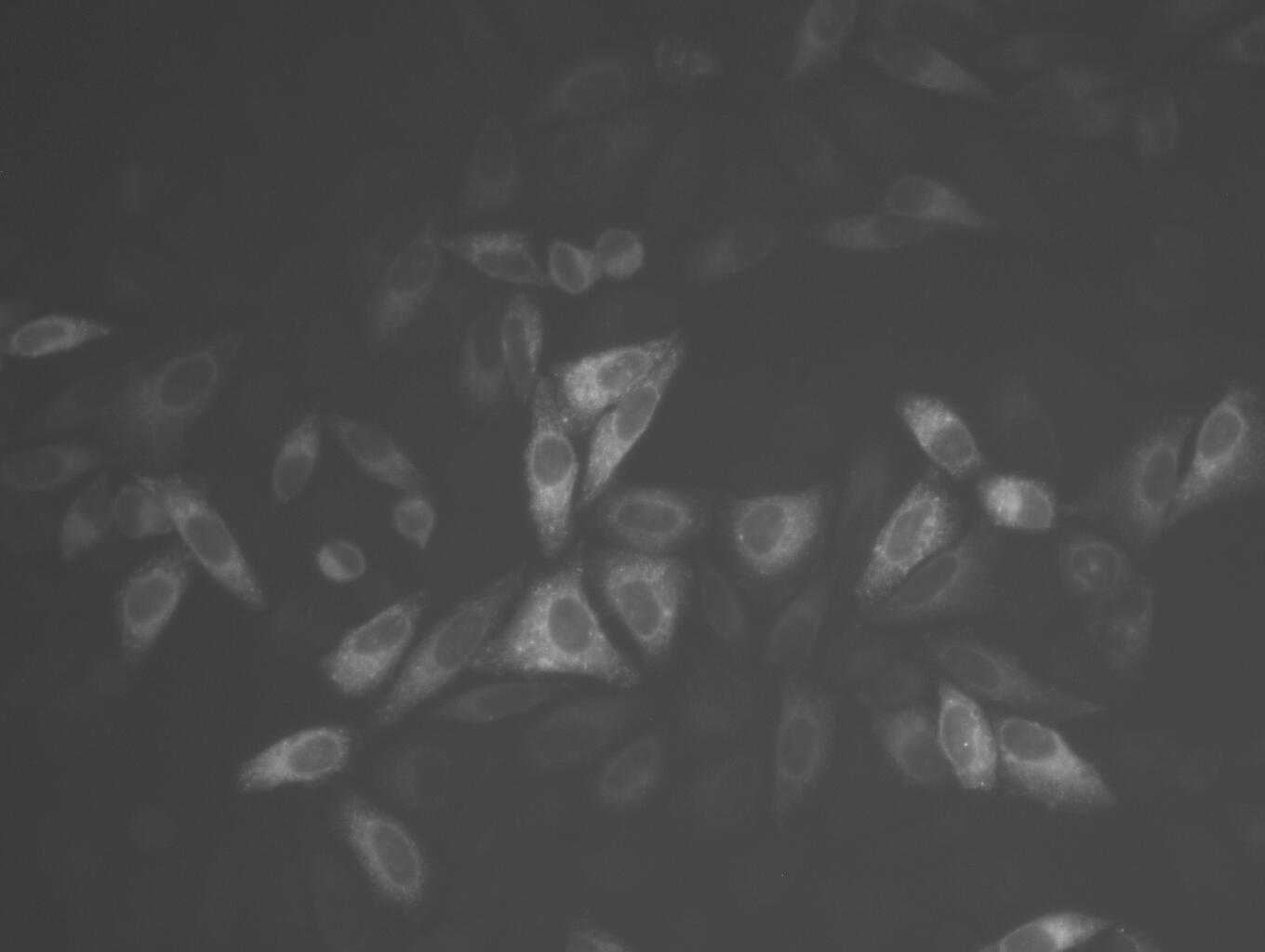

Supplement: Supplementary file 5 — Source data Fig. 3 [file 44318_2024_233_MOESM5_ESM.zip › 3C/2. brefeldin a biotin (-) GFP.jpg]

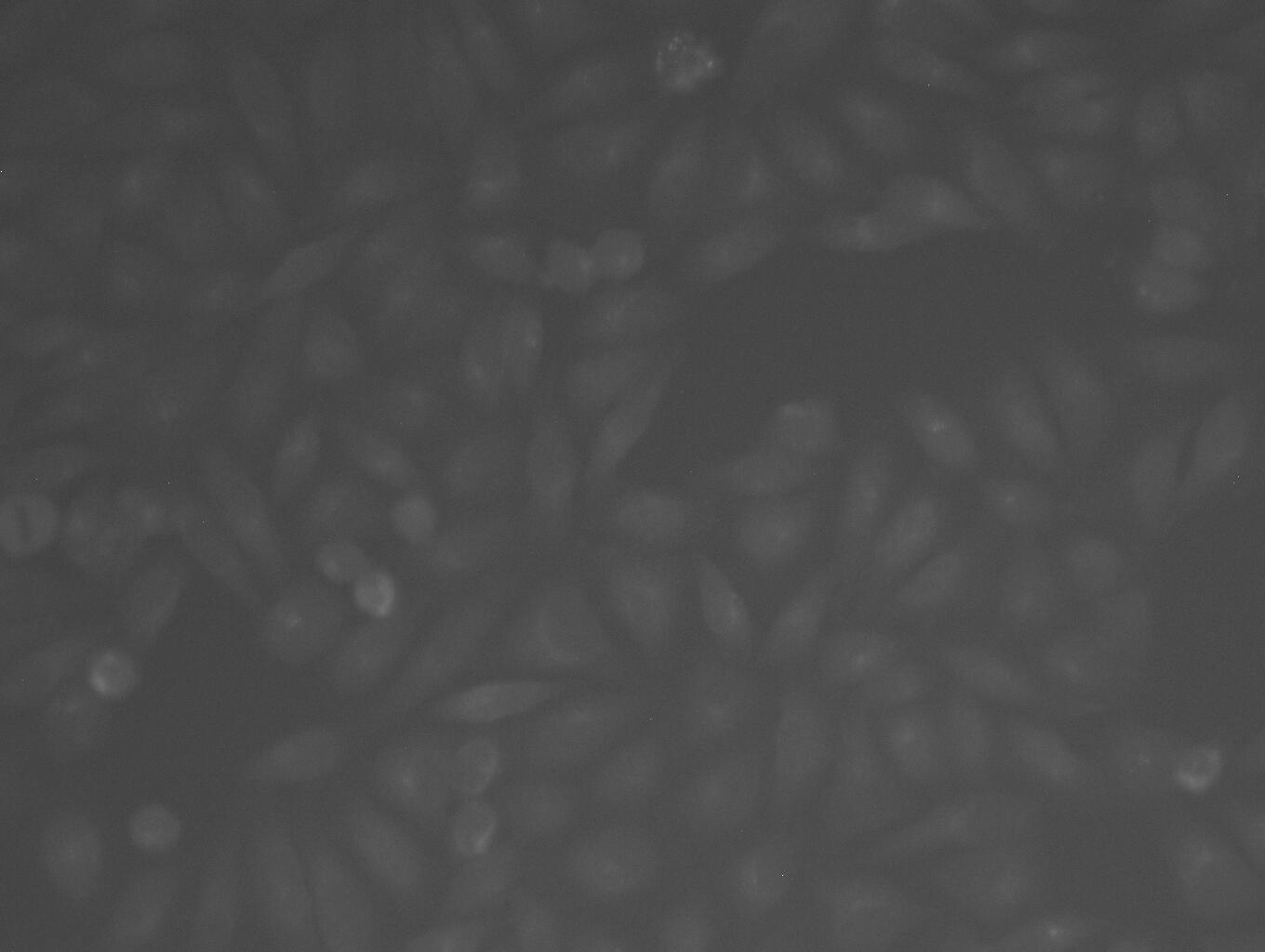

Supplement: Supplementary file 5 — Source data Fig. 3 [file 44318_2024_233_MOESM5_ESM.zip › 3C/2. brefeldin a biotin (-) Golgin-97.jpg]

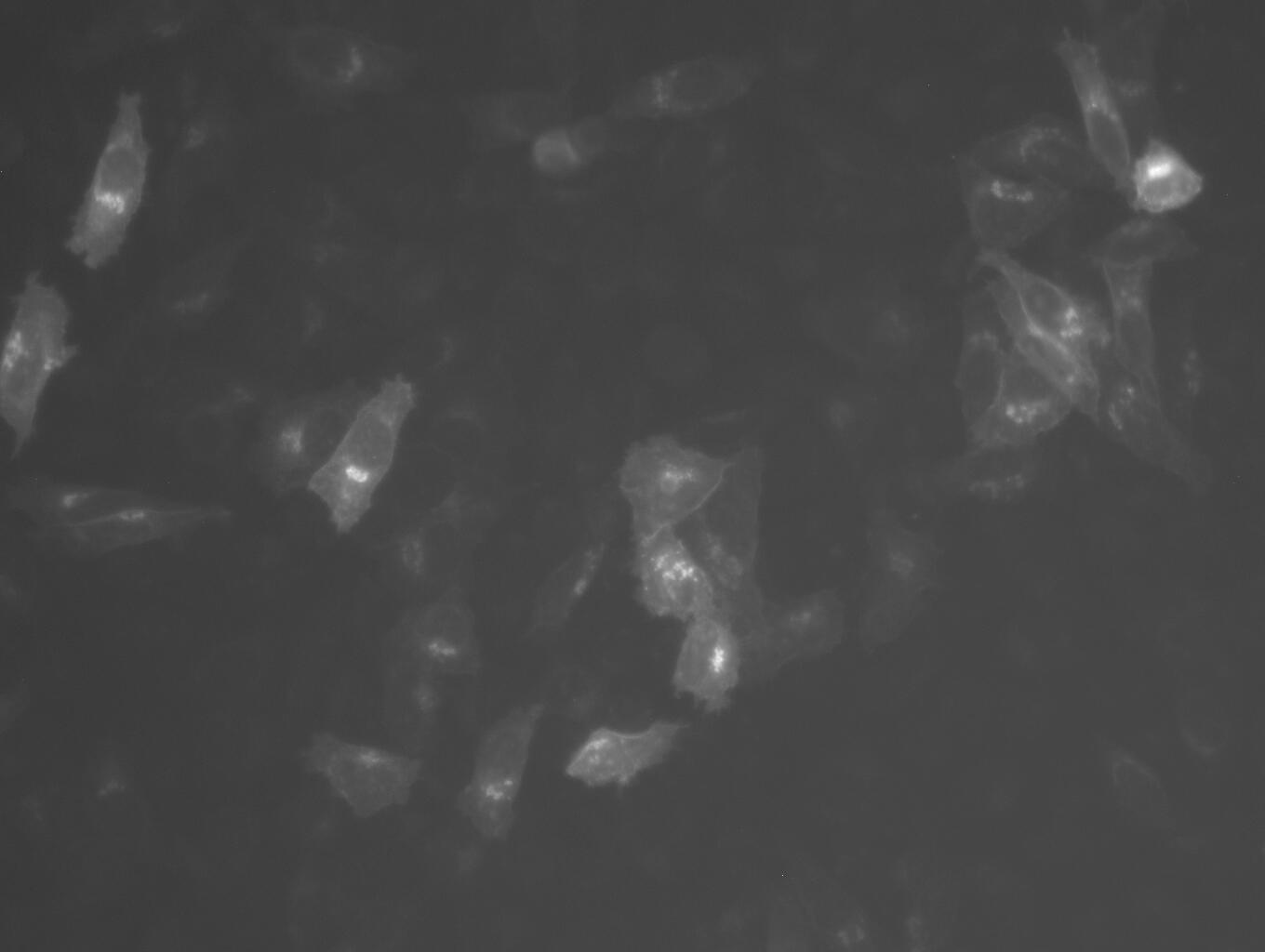

Supplement: Supplementary file 5 — Source data Fig. 3 [file 44318_2024_233_MOESM5_ESM.zip › 3C/3. monensin biotin (+) GFP.jpg]

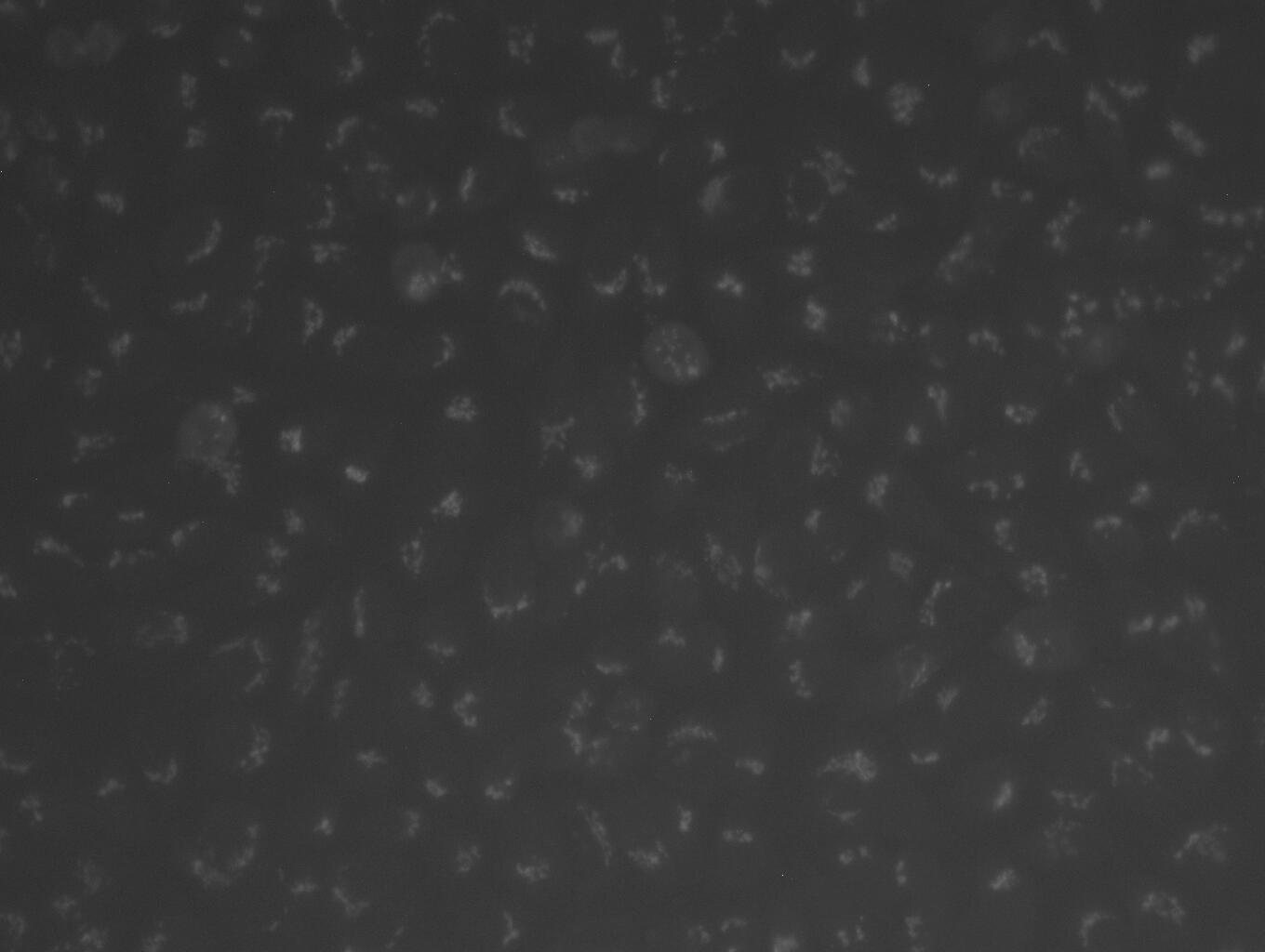

Supplement: Supplementary file 5 — Source data Fig. 3 [file 44318_2024_233_MOESM5_ESM.zip › 3C/3. monensin biotin (+) Golgin-97.jpg]

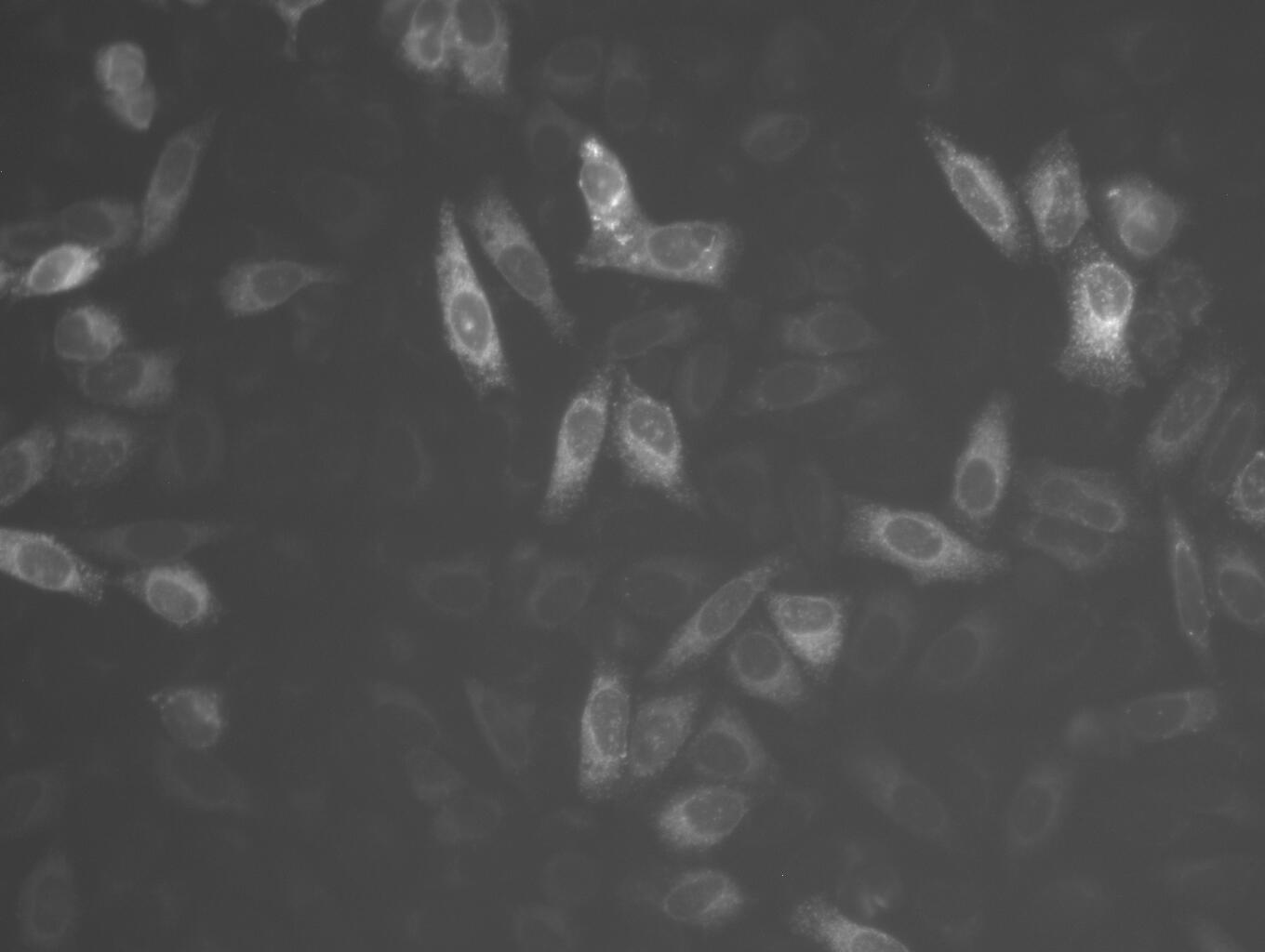

Supplement: Supplementary file 5 — Source data Fig. 3 [file 44318_2024_233_MOESM5_ESM.zip › 3C/3. monensin biotin (-) GFP.jpg]

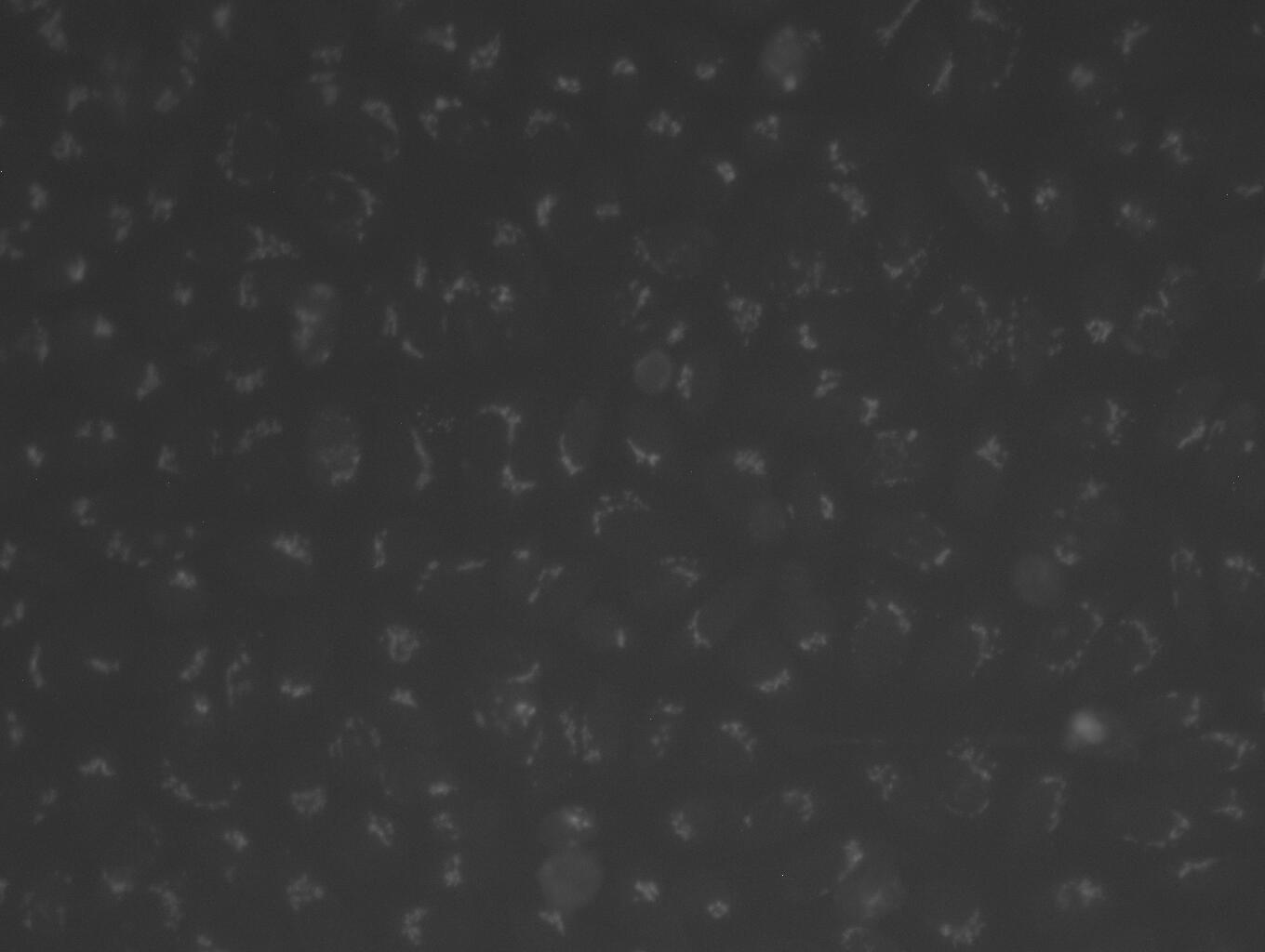

Supplement: Supplementary file 5 — Source data Fig. 3 [file 44318_2024_233_MOESM5_ESM.zip › 3C/3. monensin biotin (-) Golgin-97.jpg]

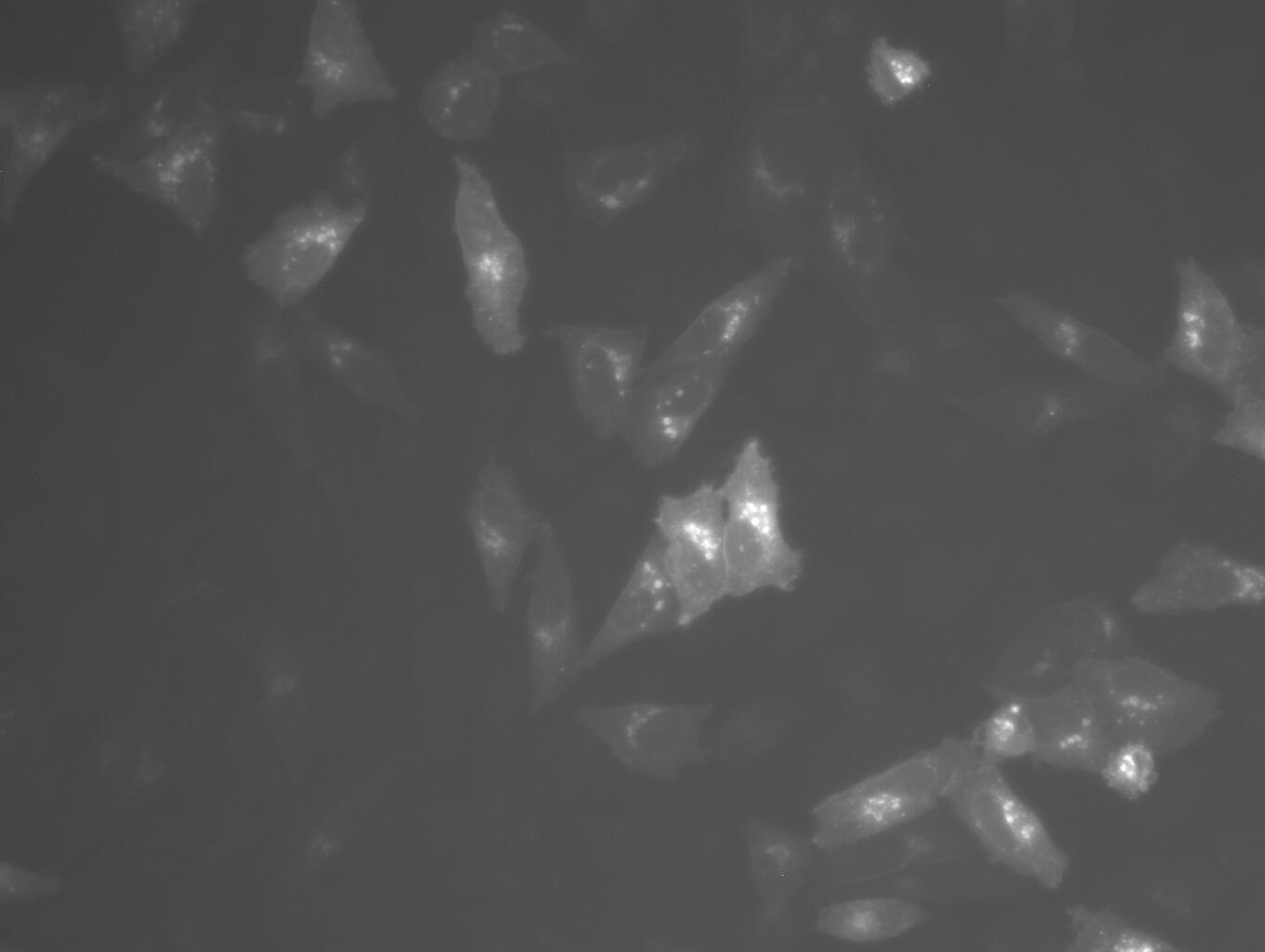

Supplement: Supplementary file 5 — Source data Fig. 3 [file 44318_2024_233_MOESM5_ESM.zip › 3C/4. niclosamide biotin (+) GFP.jpg]

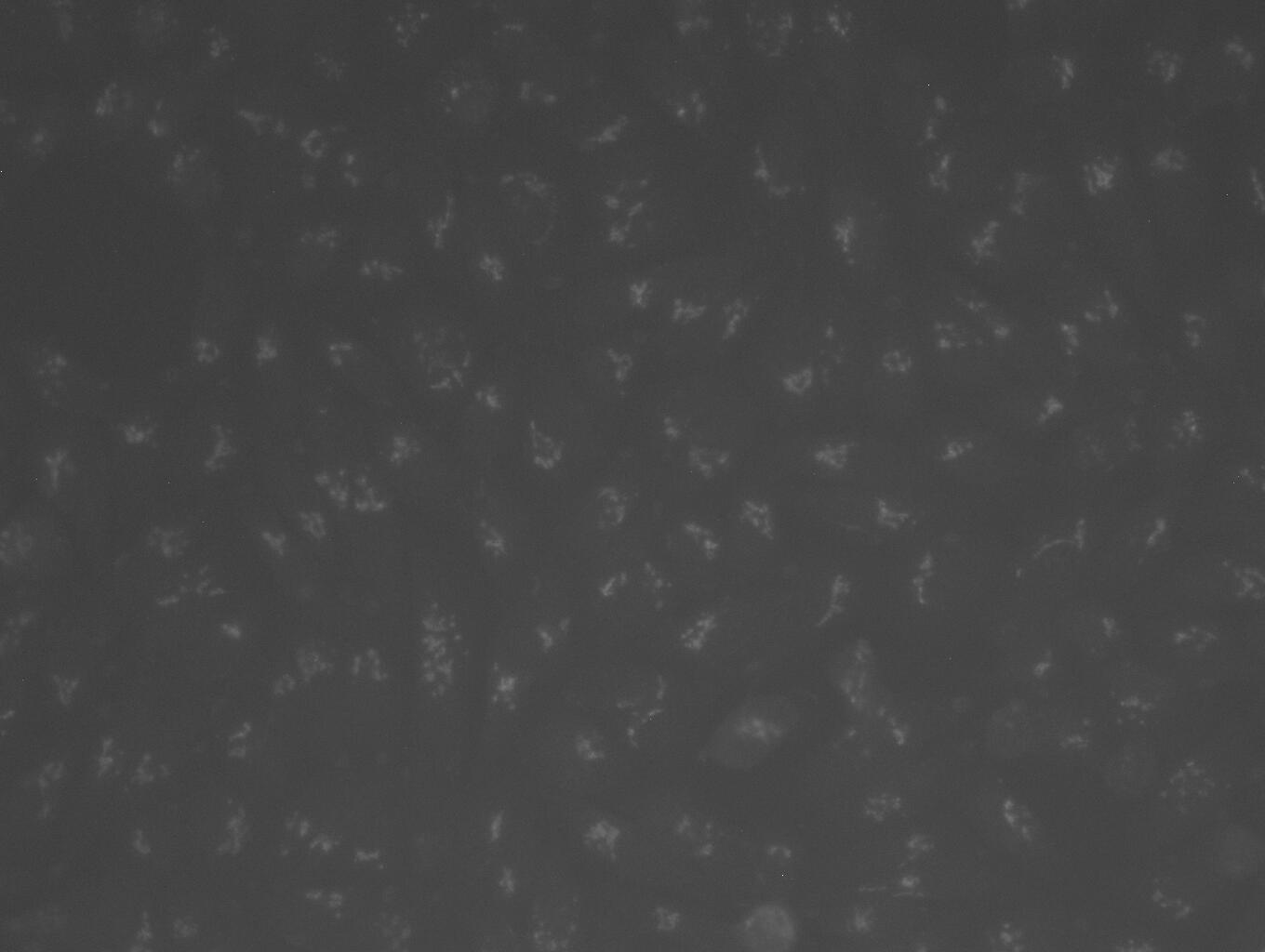

Supplement: Supplementary file 5 — Source data Fig. 3 [file 44318_2024_233_MOESM5_ESM.zip › 3C/4. niclosamide biotin (+) Golgin-97.jpg]

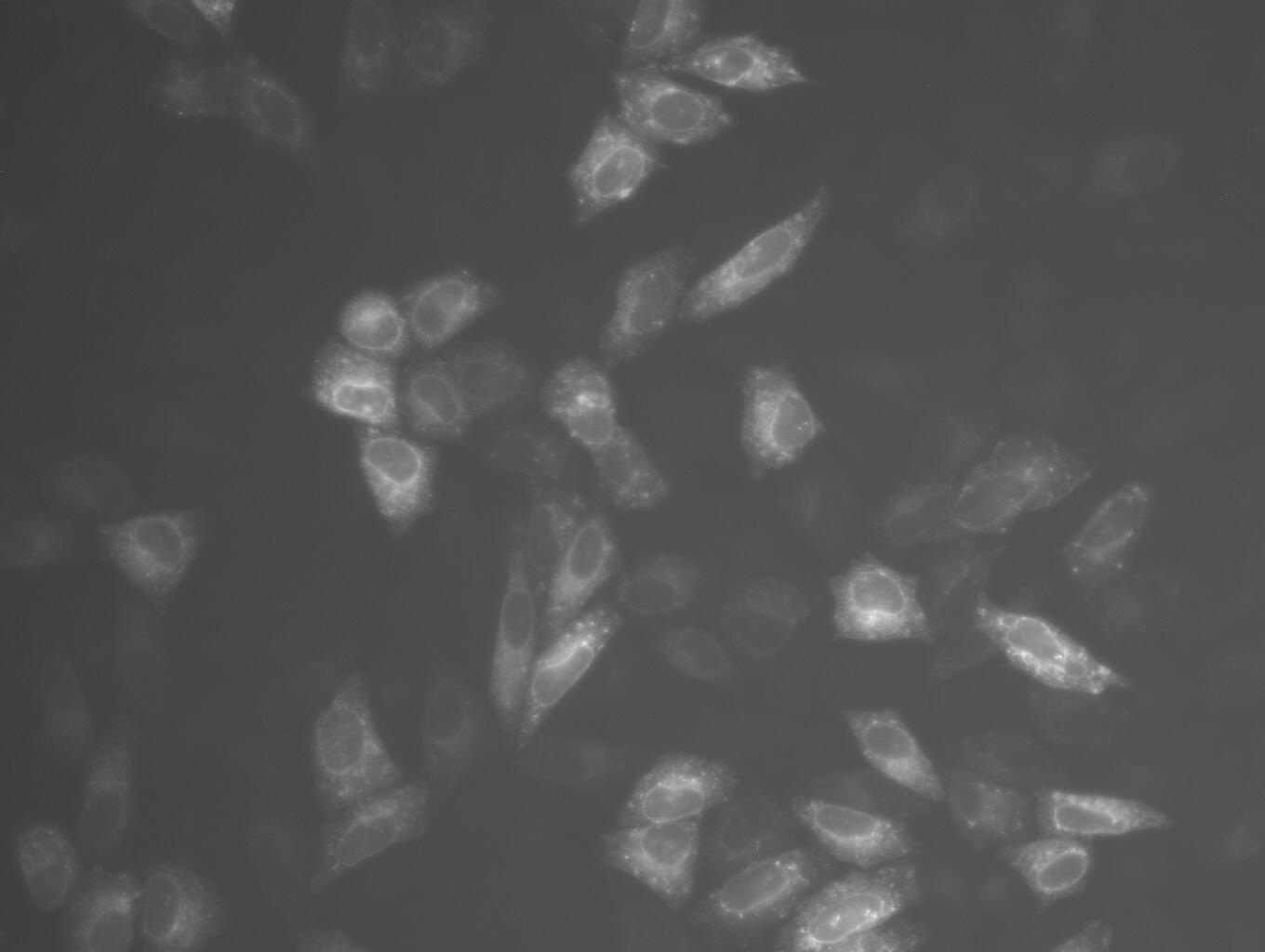

Supplement: Supplementary file 5 — Source data Fig. 3 [file 44318_2024_233_MOESM5_ESM.zip › 3C/4. niclosamide biotin (-) GFP.jpg]

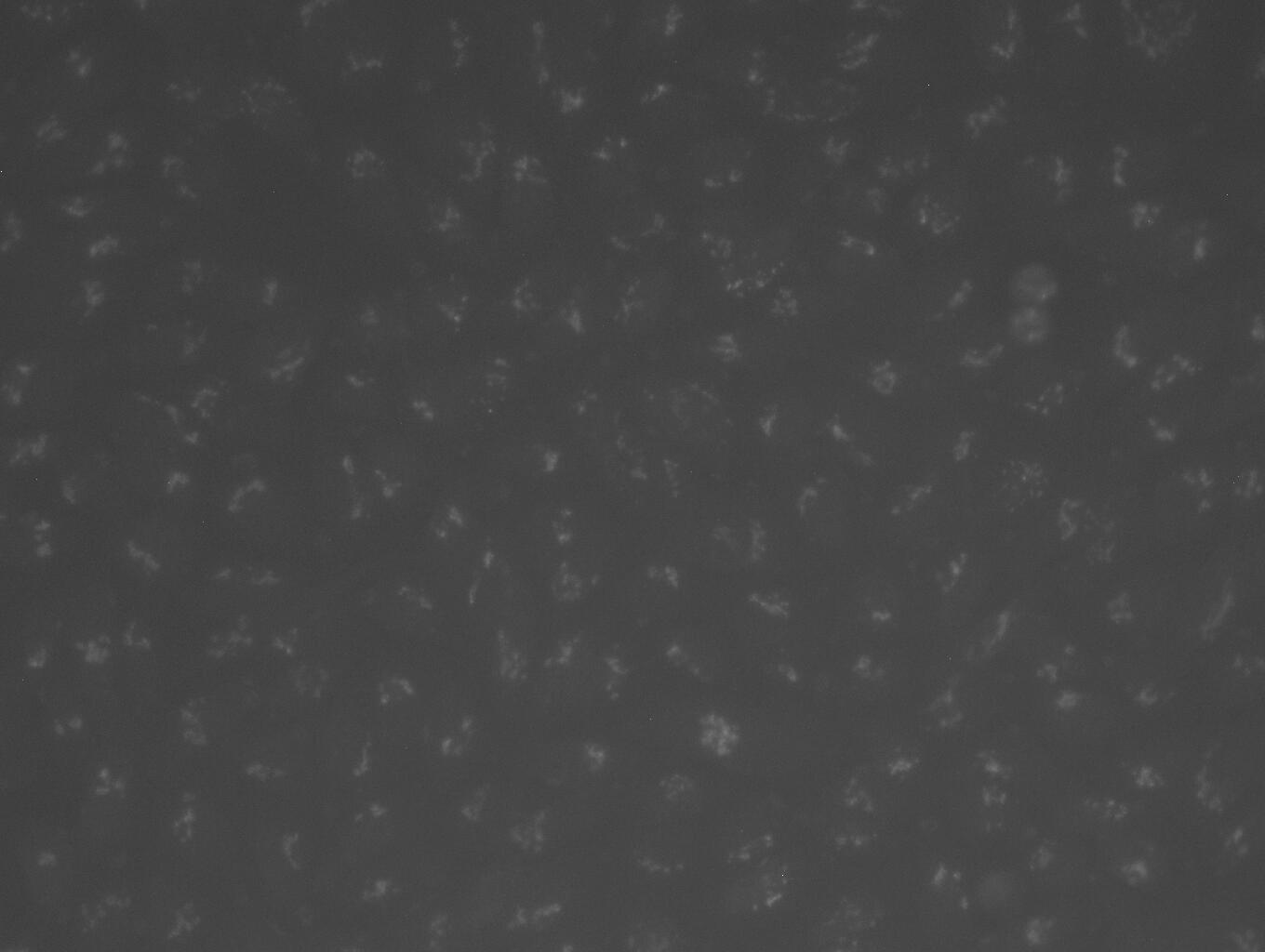

Supplement: Supplementary file 5 — Source data Fig. 3 [file 44318_2024_233_MOESM5_ESM.zip › 3C/4. niclosamide biotin (-) Golgi-97.jpg]

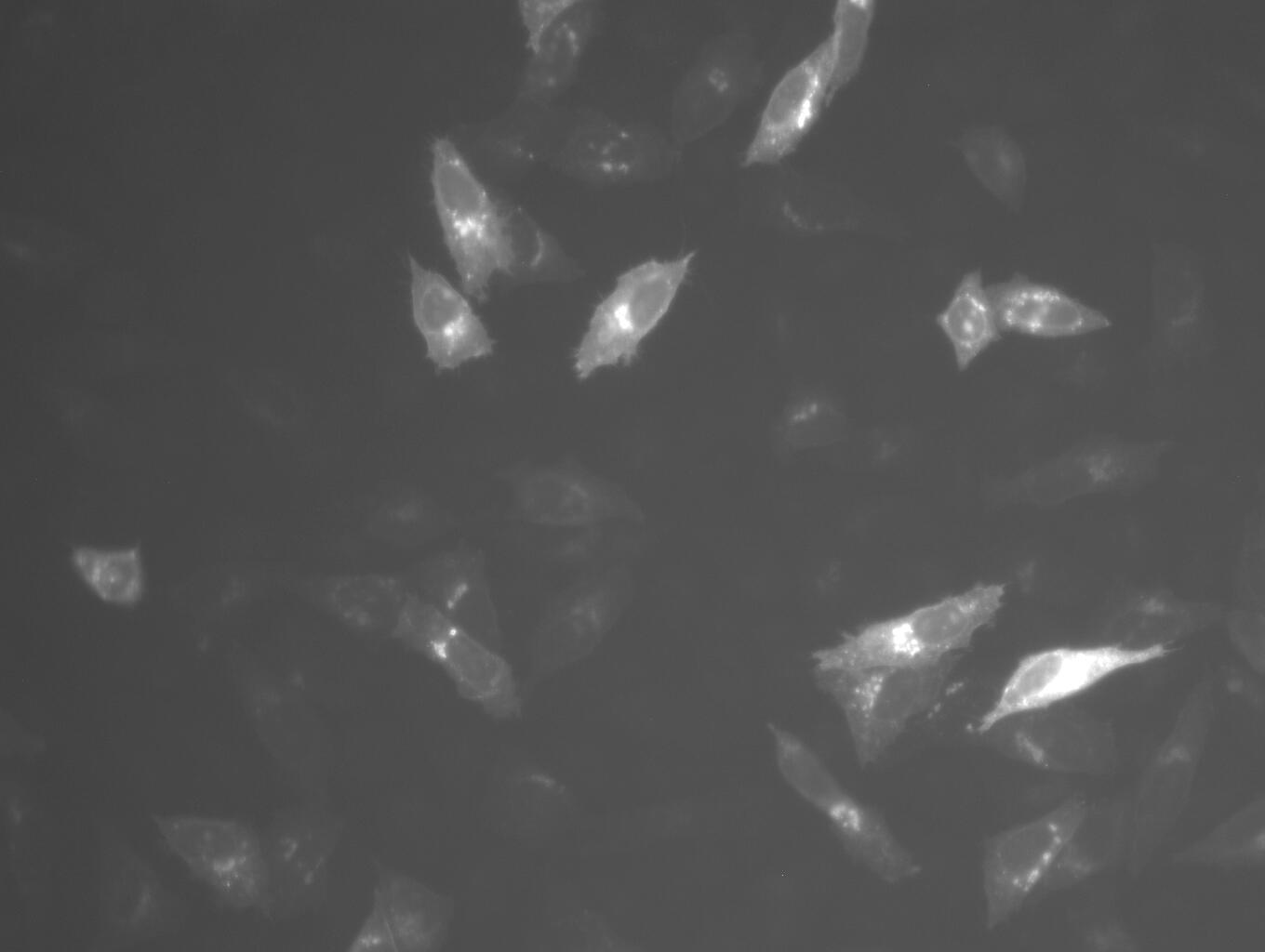

Supplement: Supplementary file 5 — Source data Fig. 3 [file 44318_2024_233_MOESM5_ESM.zip › 3C/5. AMDE-1 biotin (+) GFP.jpg]

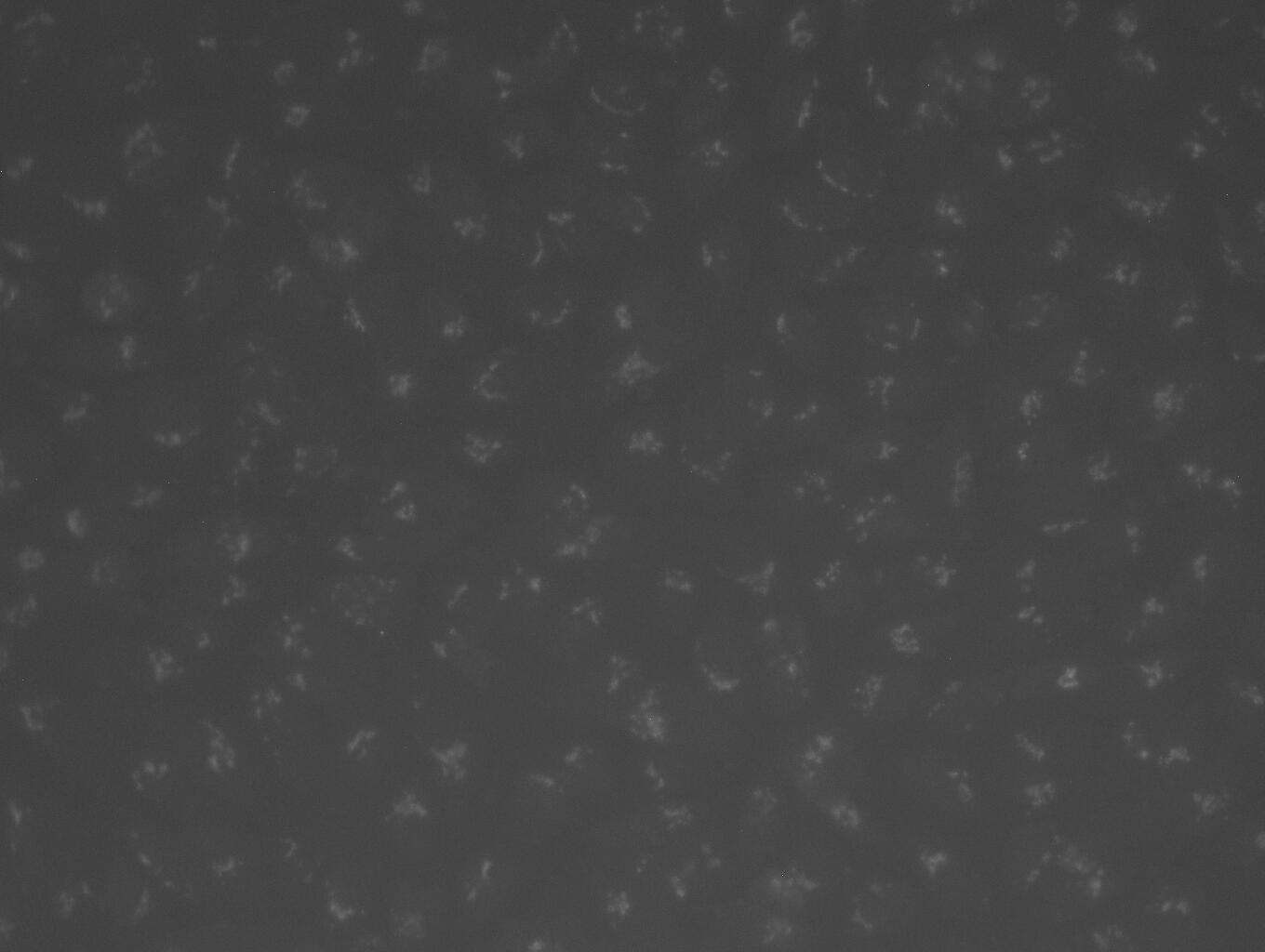

Supplement: Supplementary file 5 — Source data Fig. 3 [file 44318_2024_233_MOESM5_ESM.zip › 3C/5. AMDE-1 biotin (+) Golgin-97.jpg]

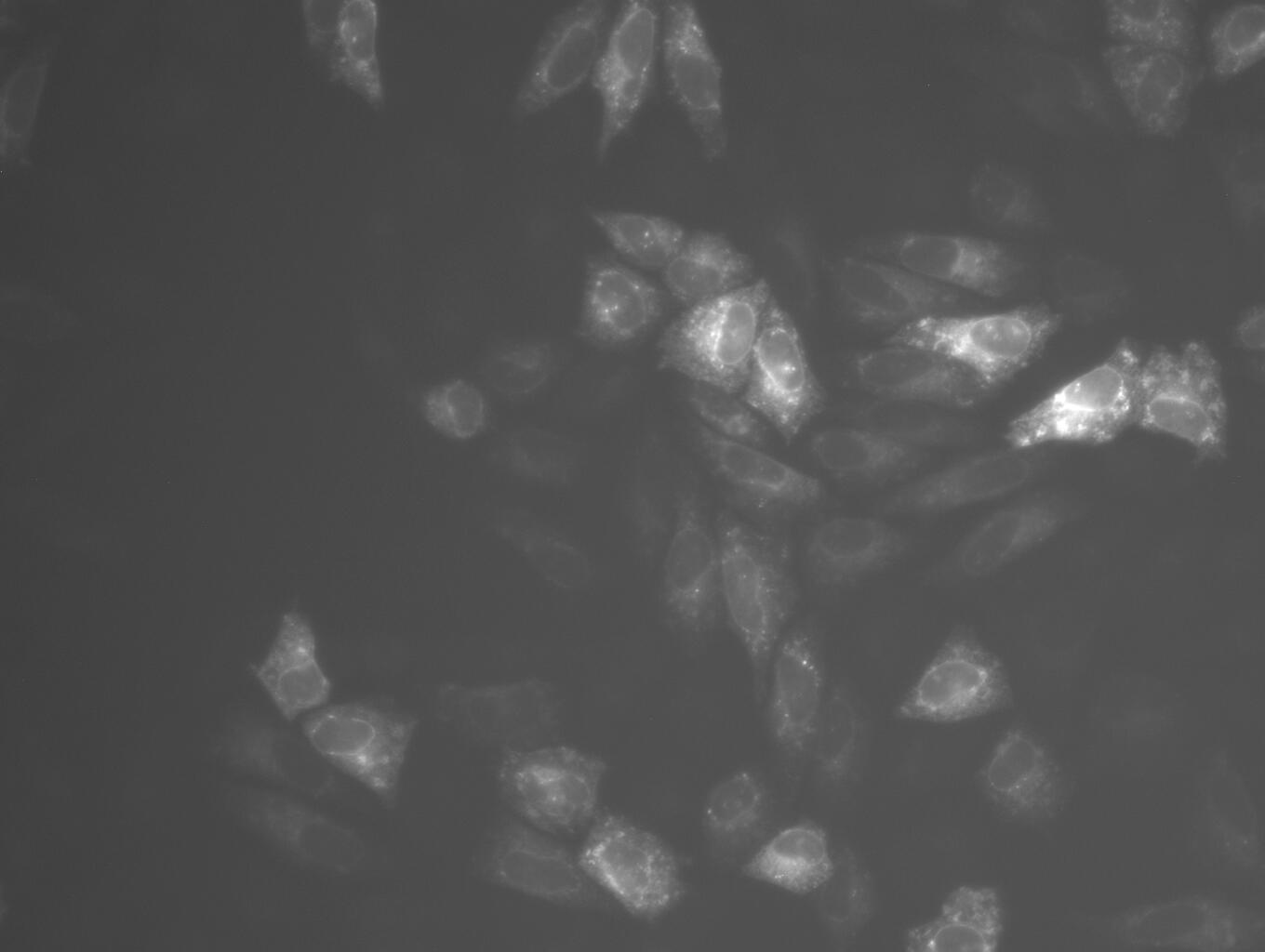

Supplement: Supplementary file 5 — Source data Fig. 3 [file 44318_2024_233_MOESM5_ESM.zip › 3C/5. AMDE-1 biotin (-) GFP.jpg]

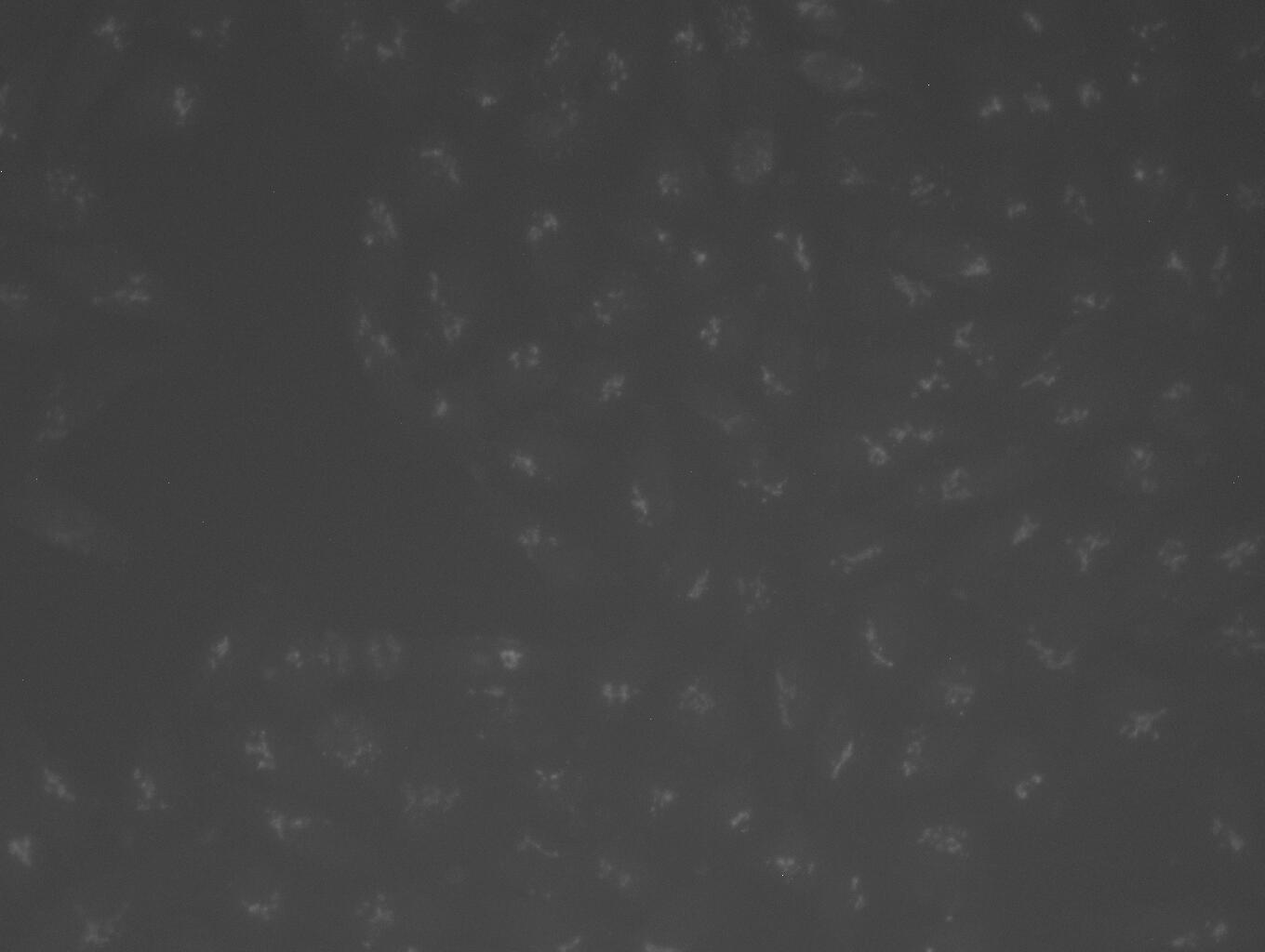

Supplement: Supplementary file 5 — Source data Fig. 3 [file 44318_2024_233_MOESM5_ESM.zip › 3C/5. AMDE-1 biotin (-) Golgin-97.jpg]

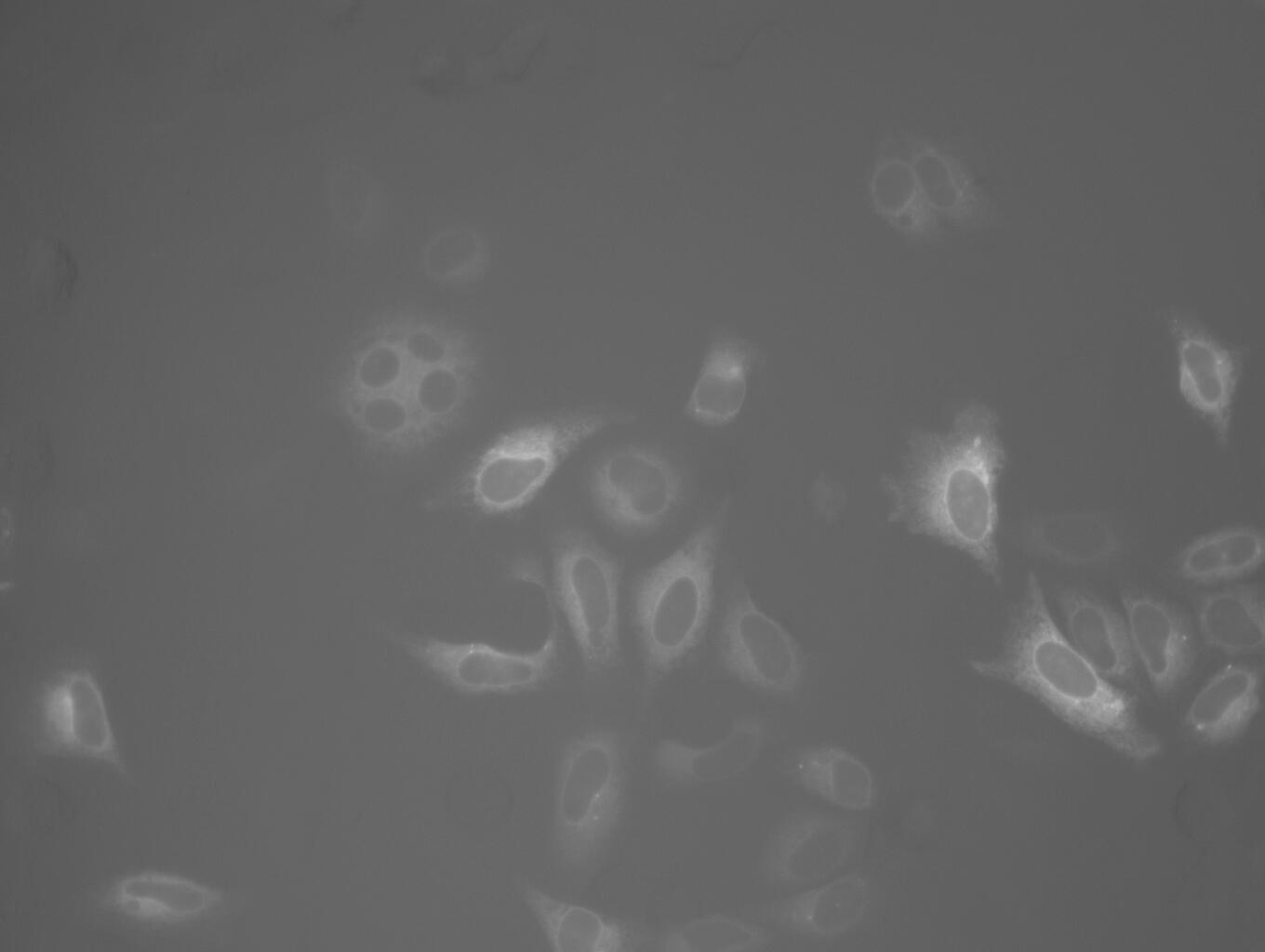

Supplement: Supplementary file 5 — Source data Fig. 3 [file 44318_2024_233_MOESM5_ESM.zip › 3F/Image/1. sgCtrl veh biotin (-).jpg]

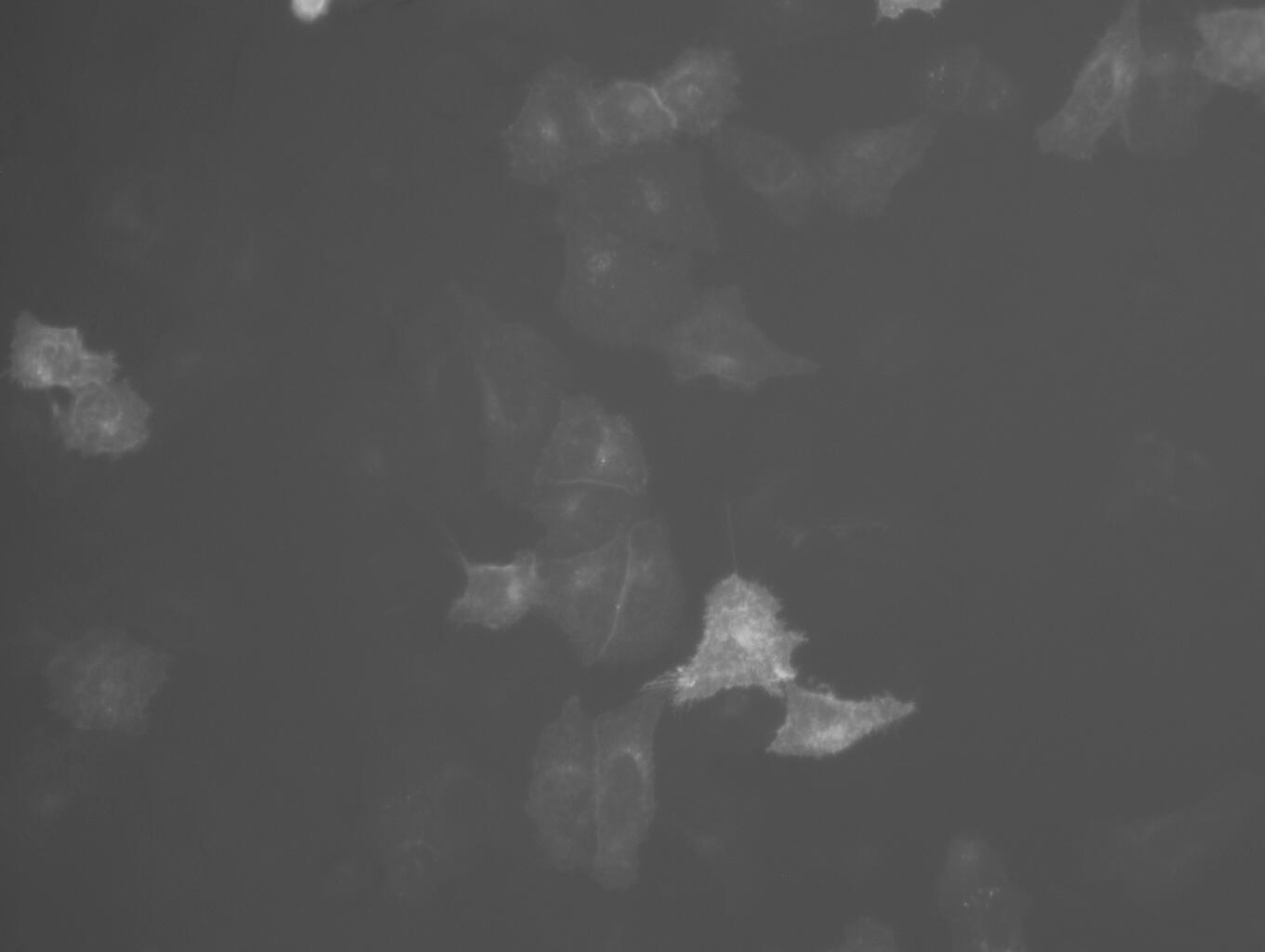

Supplement: Supplementary file 5 — Source data Fig. 3 [file 44318_2024_233_MOESM5_ESM.zip › 3F/Image/2. sgCtrl veh biotin (+).jpg]

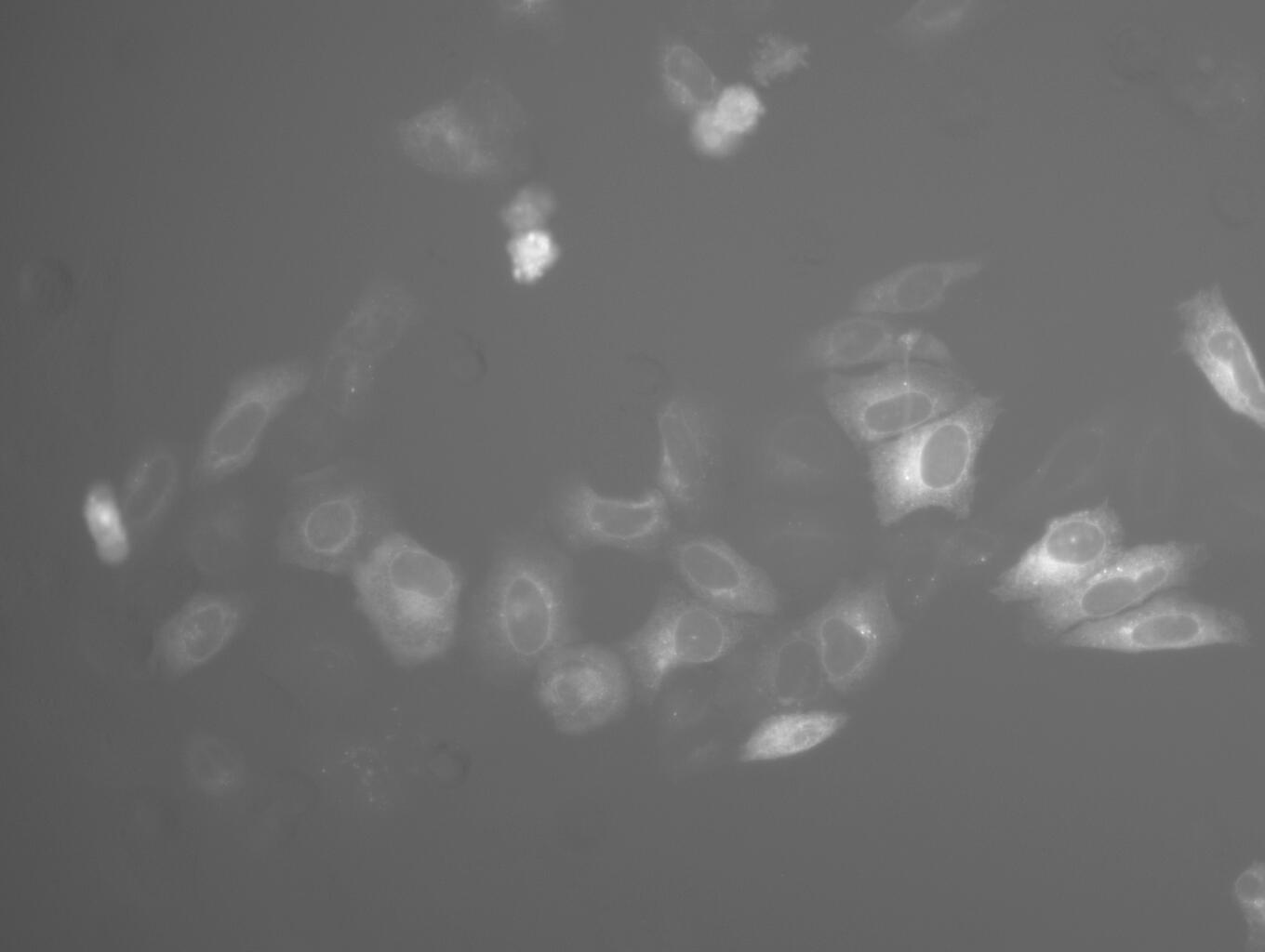

Supplement: Supplementary file 5 — Source data Fig. 3 [file 44318_2024_233_MOESM5_ESM.zip › 3F/Image/3. sgCtrl niclosamide biotin (-).jpg]

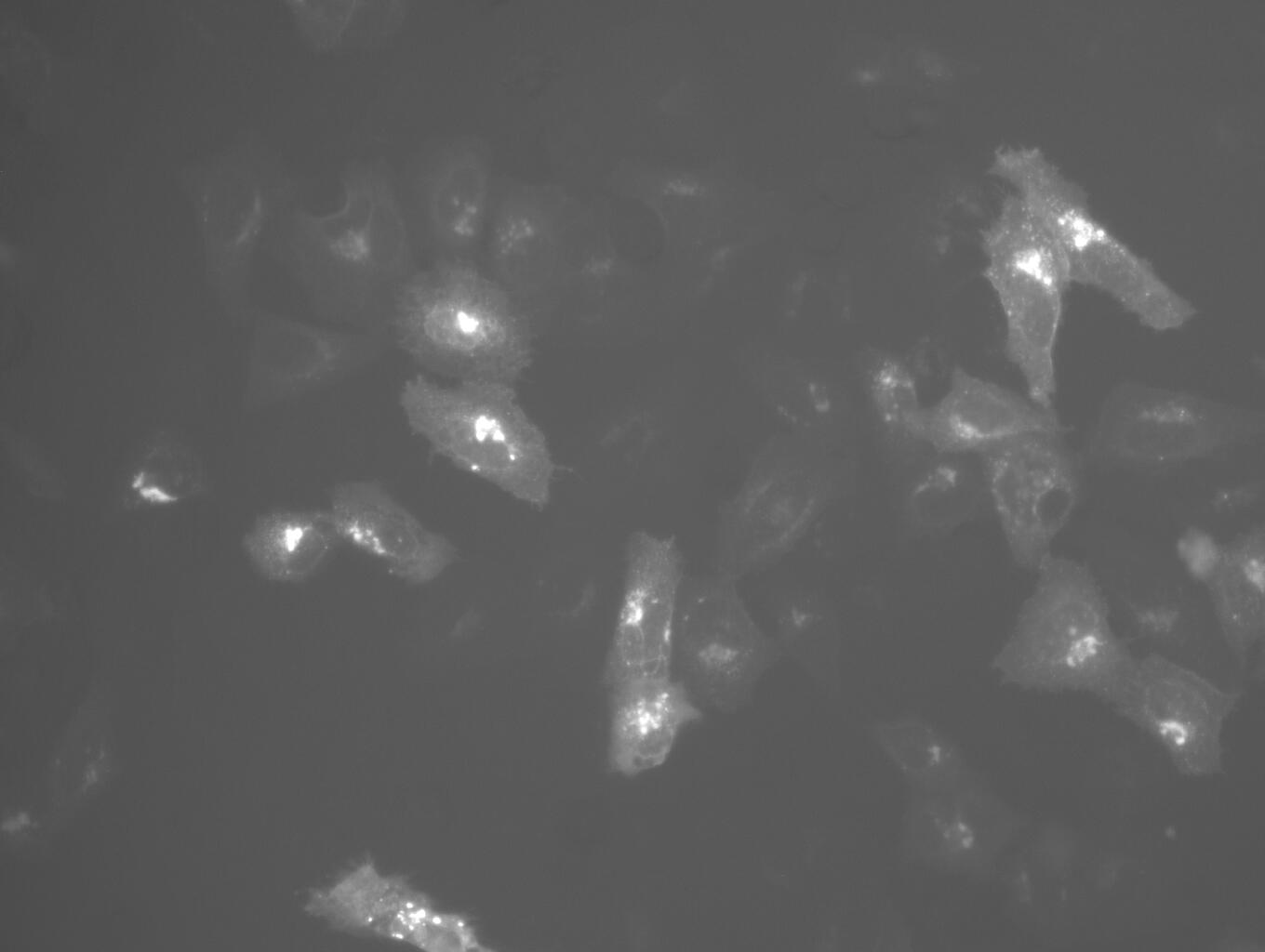

Supplement: Supplementary file 5 — Source data Fig. 3 [file 44318_2024_233_MOESM5_ESM.zip › 3F/Image/4. sgCtrl niclosamide biotin (+).jpg]

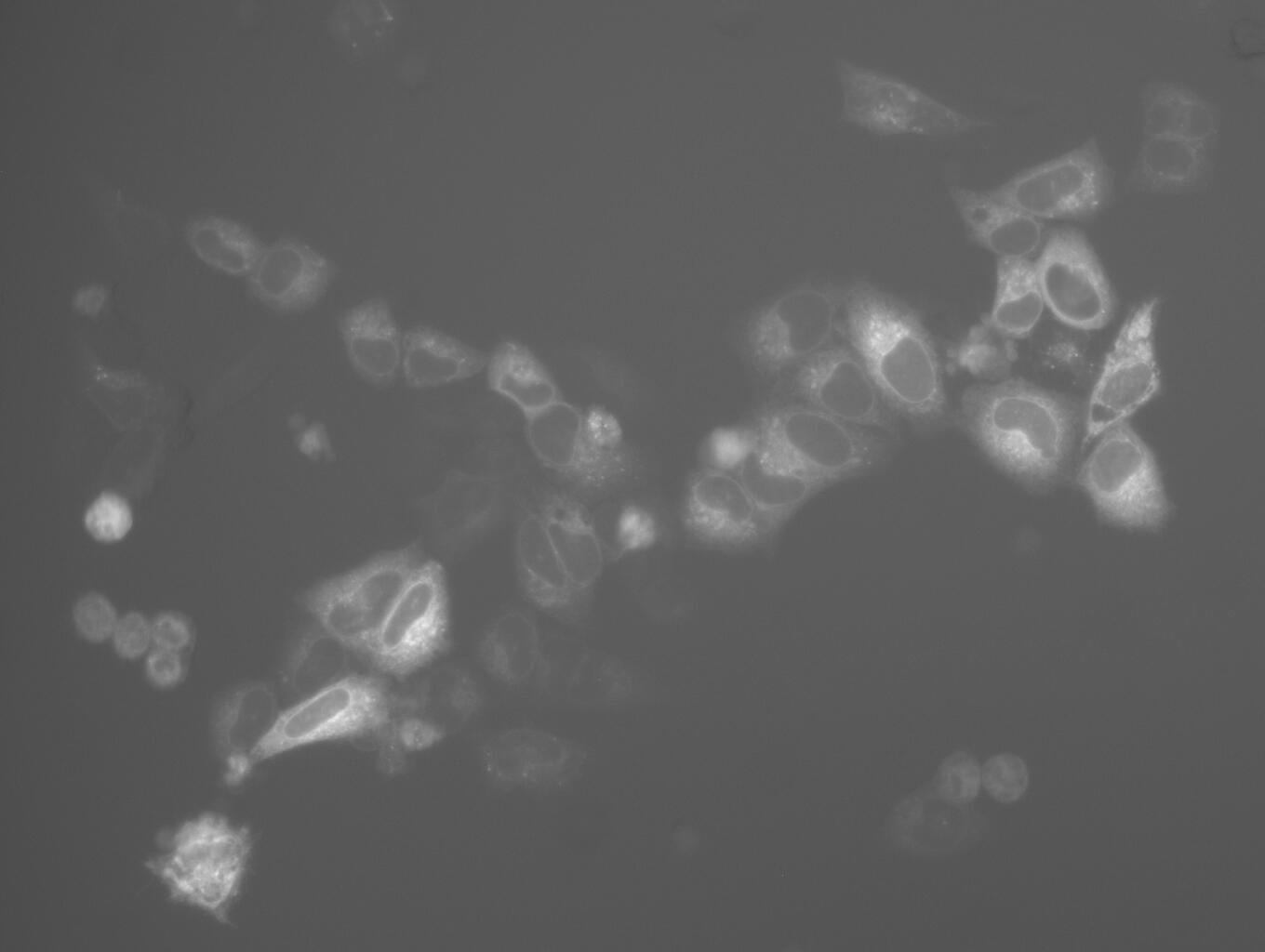

Supplement: Supplementary file 5 — Source data Fig. 3 [file 44318_2024_233_MOESM5_ESM.zip › 3F/Image/5. sgATG16L1 veh biotin (-).jpg]

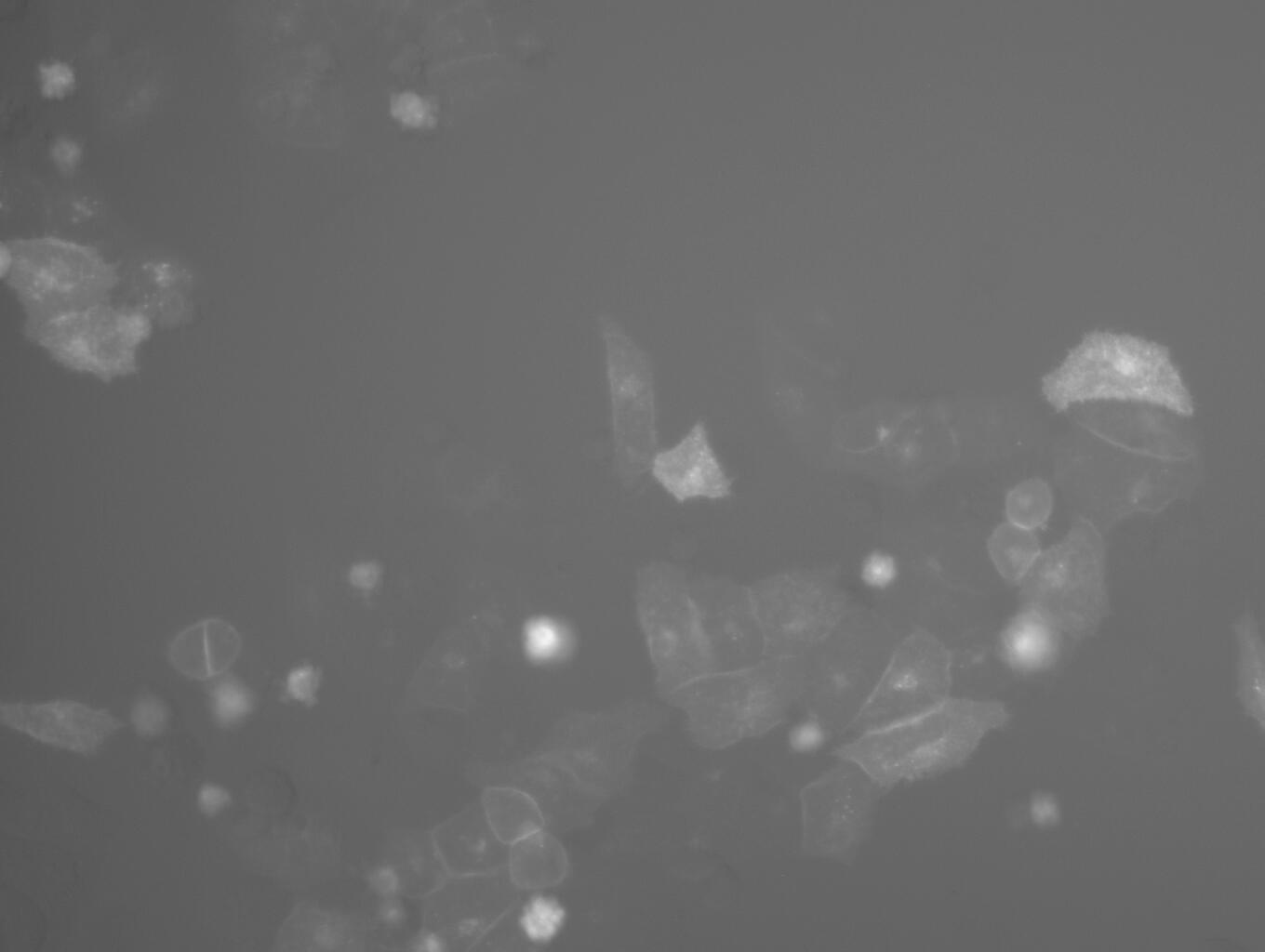

Supplement: Supplementary file 5 — Source data Fig. 3 [file 44318_2024_233_MOESM5_ESM.zip › 3F/Image/6. sgATG16L1 veh biotin (+).jpg]

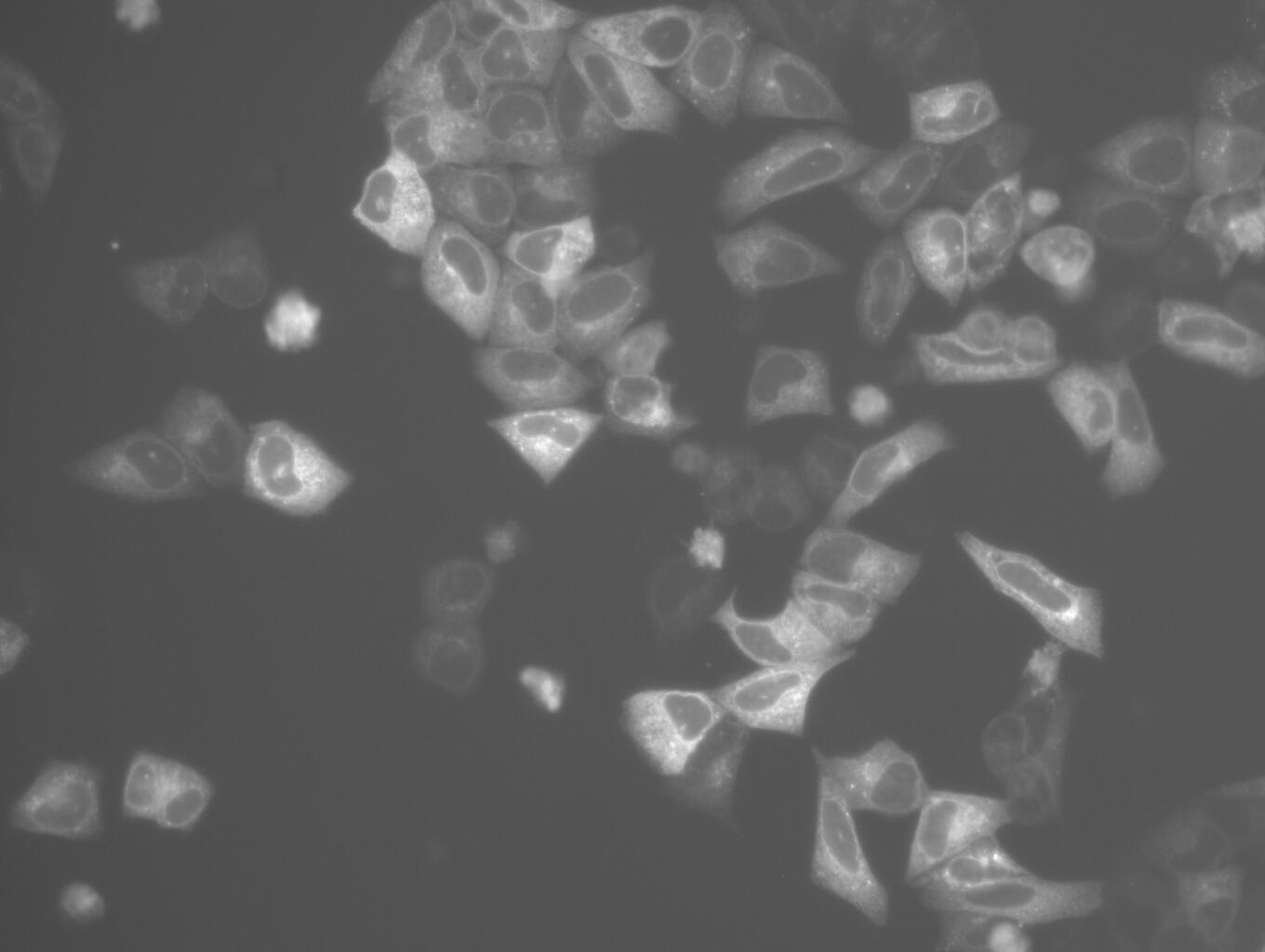

Supplement: Supplementary file 5 — Source data Fig. 3 [file 44318_2024_233_MOESM5_ESM.zip › 3F/Image/7. sgATG16L1 niclosamide biotin (-).jpg]

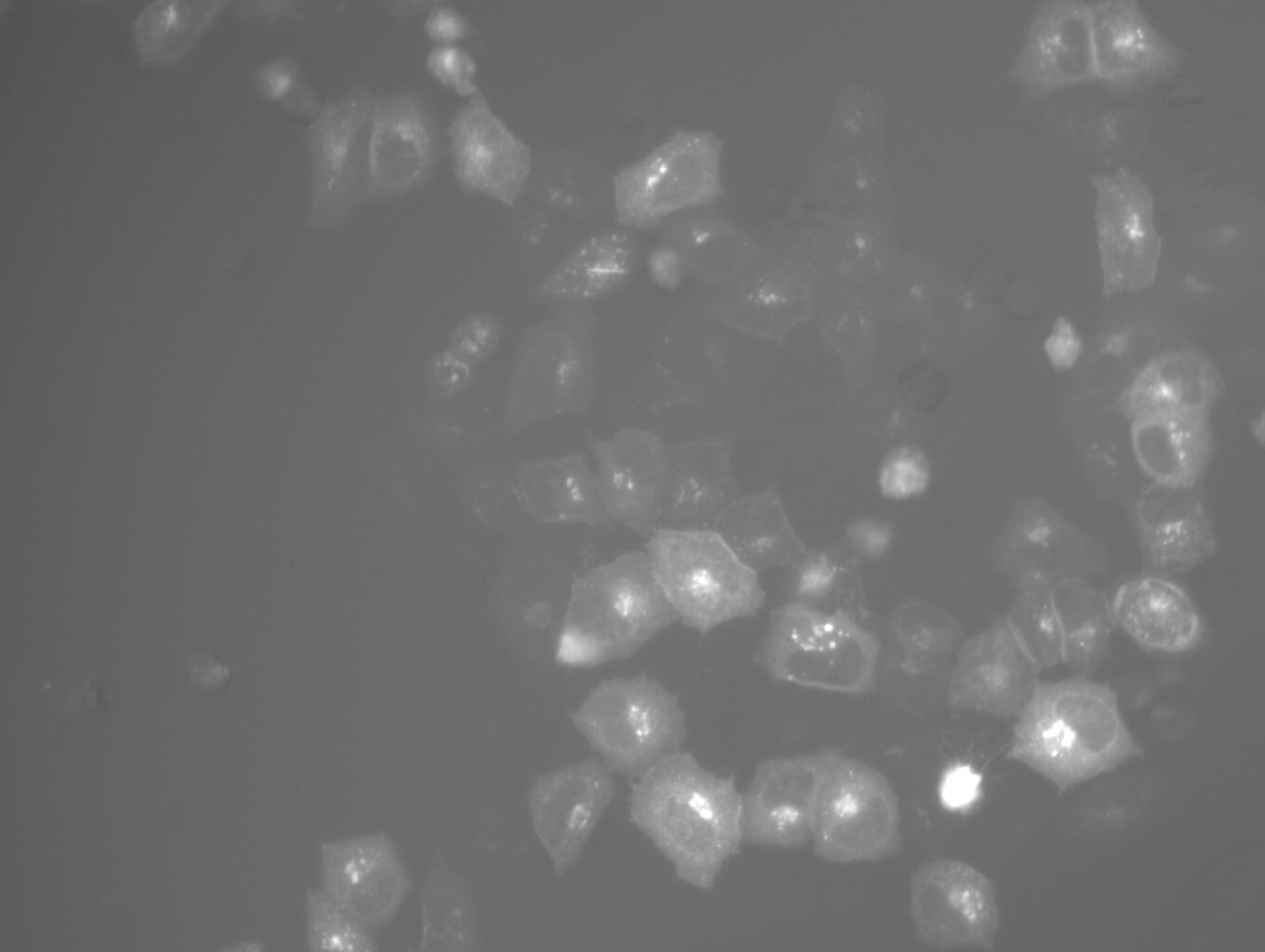

Supplement: Supplementary file 5 — Source data Fig. 3 [file 44318_2024_233_MOESM5_ESM.zip › 3F/Image/8. sgATG16L1 niclosamide biotin (+).jpg]

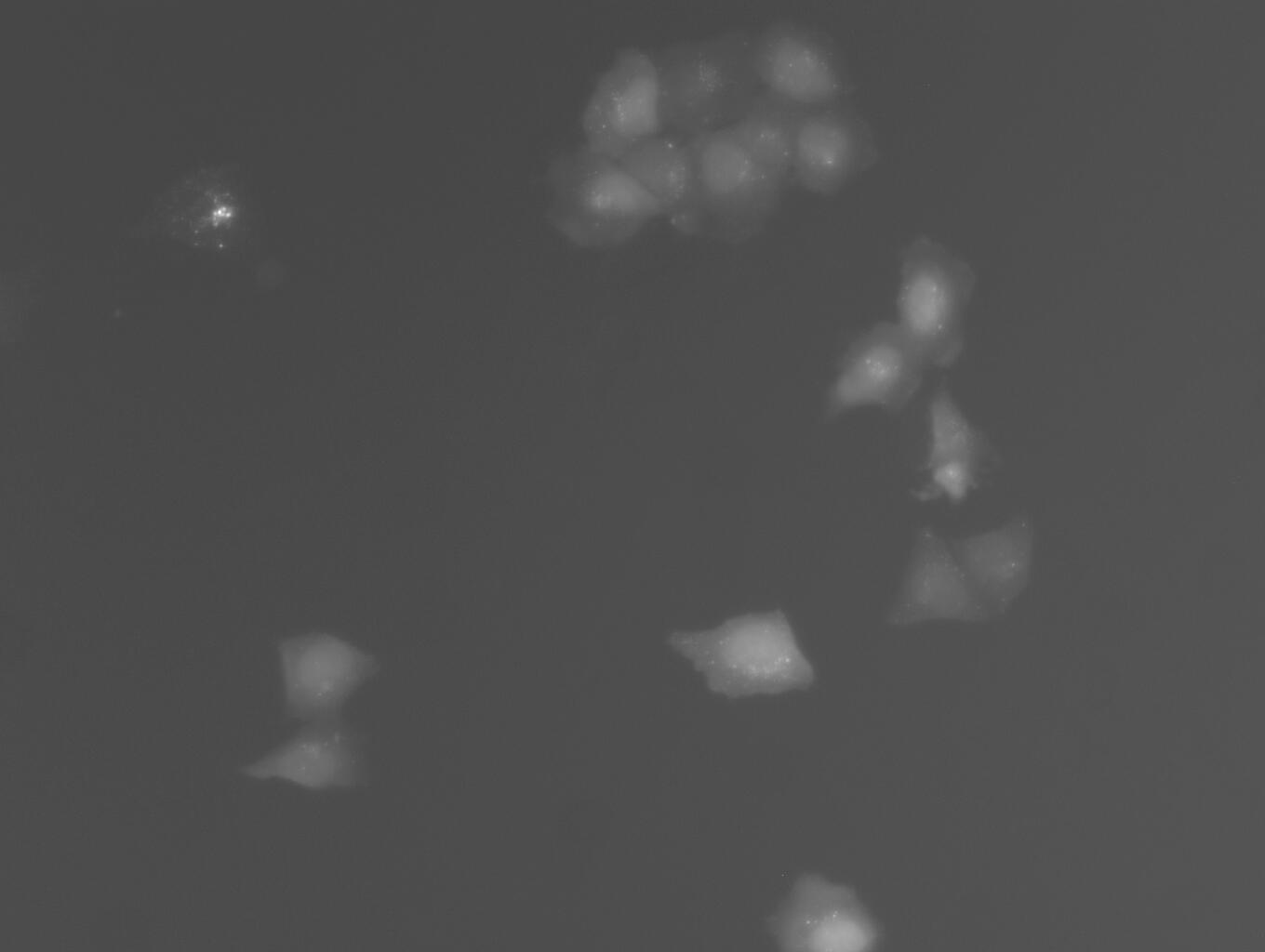

Supplement: Supplementary file 6 — Source data Fig. 4 [file 44318_2024_233_MOESM6_ESM.zip › 4A/1. Ctrl Veh.jpg]

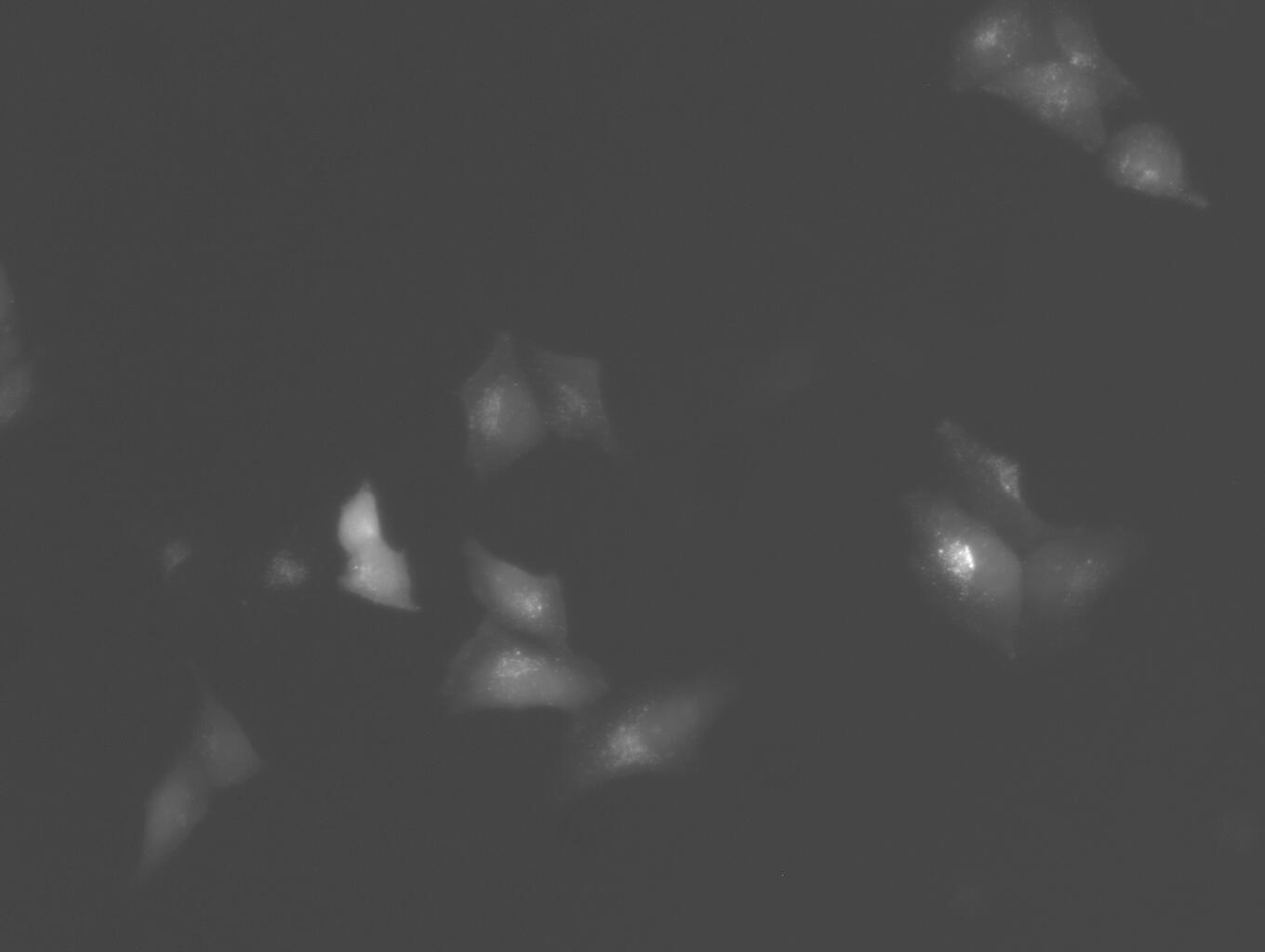

Supplement: Supplementary file 6 — Source data Fig. 4 [file 44318_2024_233_MOESM6_ESM.zip › 4A/2. Ctrl Baf.A1.jpg]

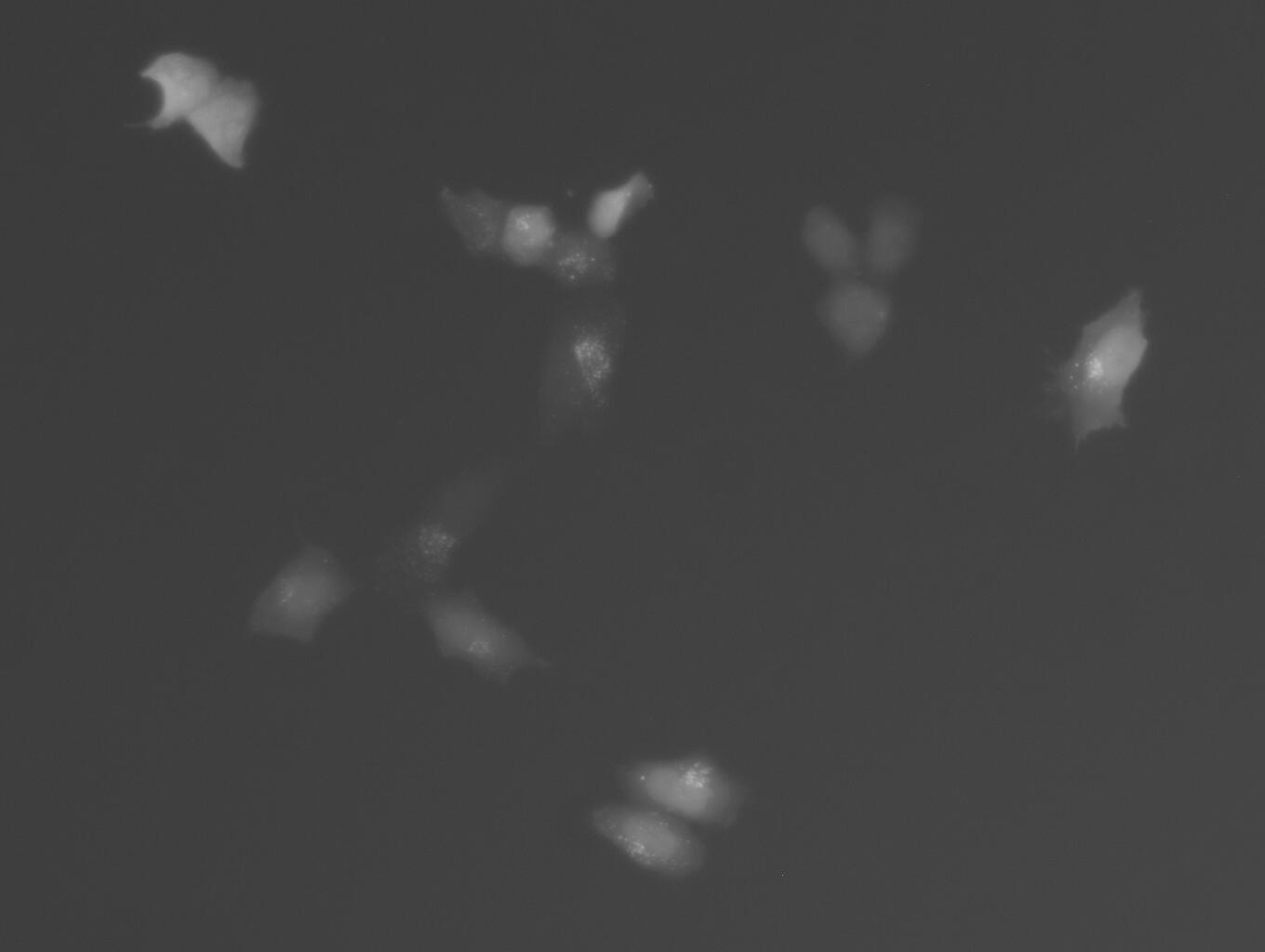

Supplement: Supplementary file 6 — Source data Fig. 4 [file 44318_2024_233_MOESM6_ESM.zip › 4A/3. Ctrl ConA.jpg]

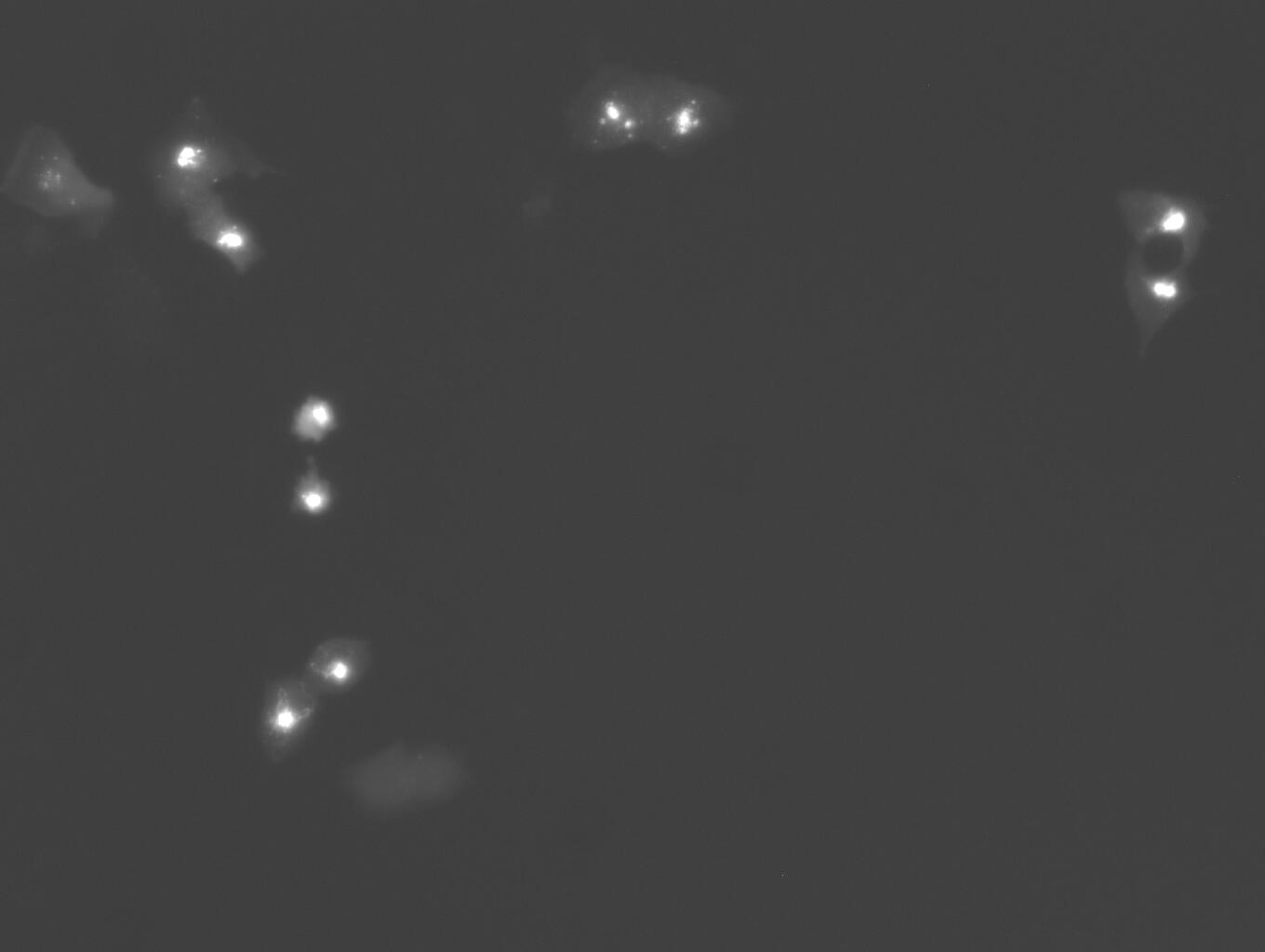

Supplement: Supplementary file 6 — Source data Fig. 4 [file 44318_2024_233_MOESM6_ESM.zip › 4A/4. DLK1 Veh.jpg]

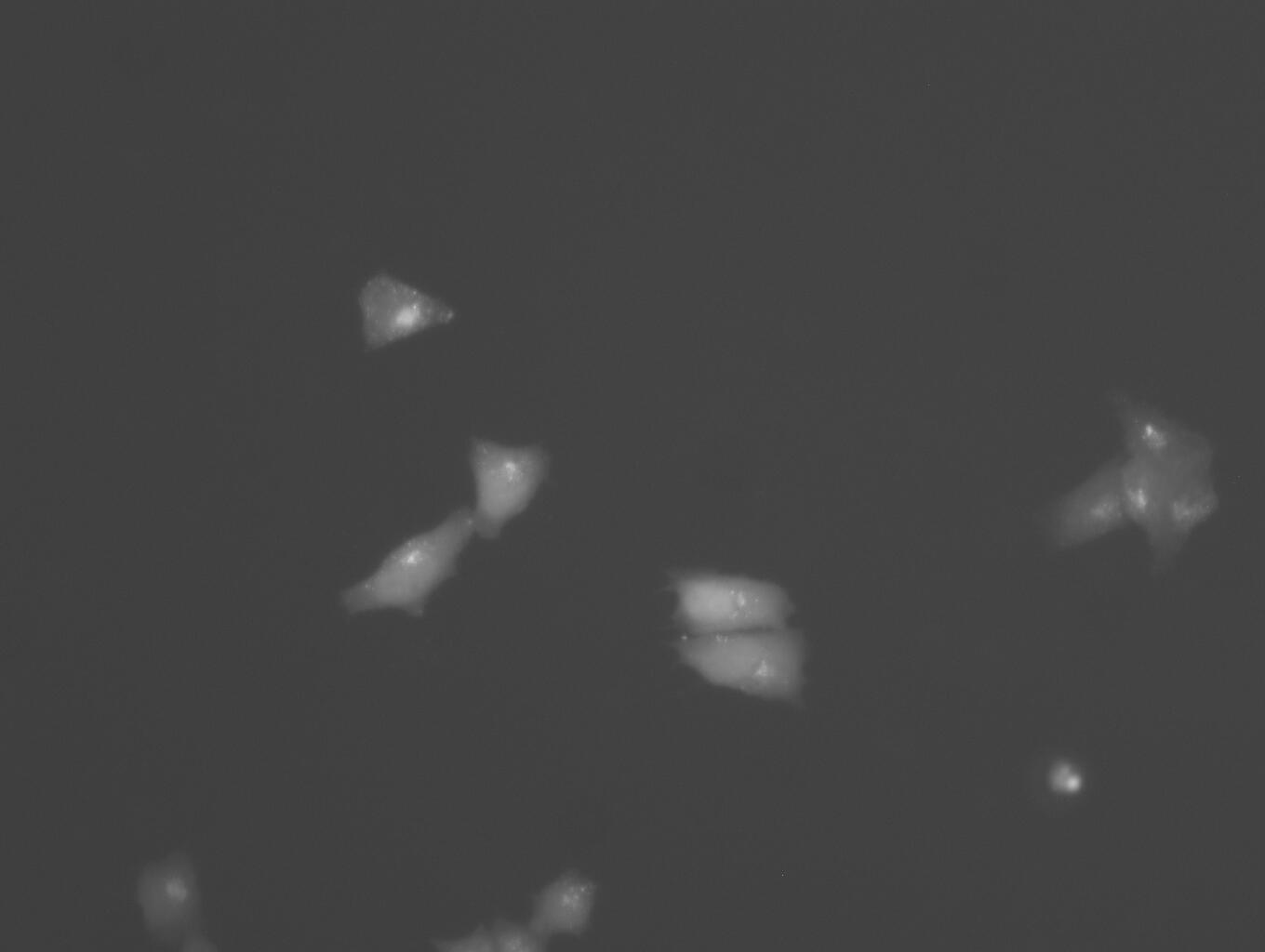

Supplement: Supplementary file 6 — Source data Fig. 4 [file 44318_2024_233_MOESM6_ESM.zip › 4A/5. DLK1 Baf.A1.jpg]

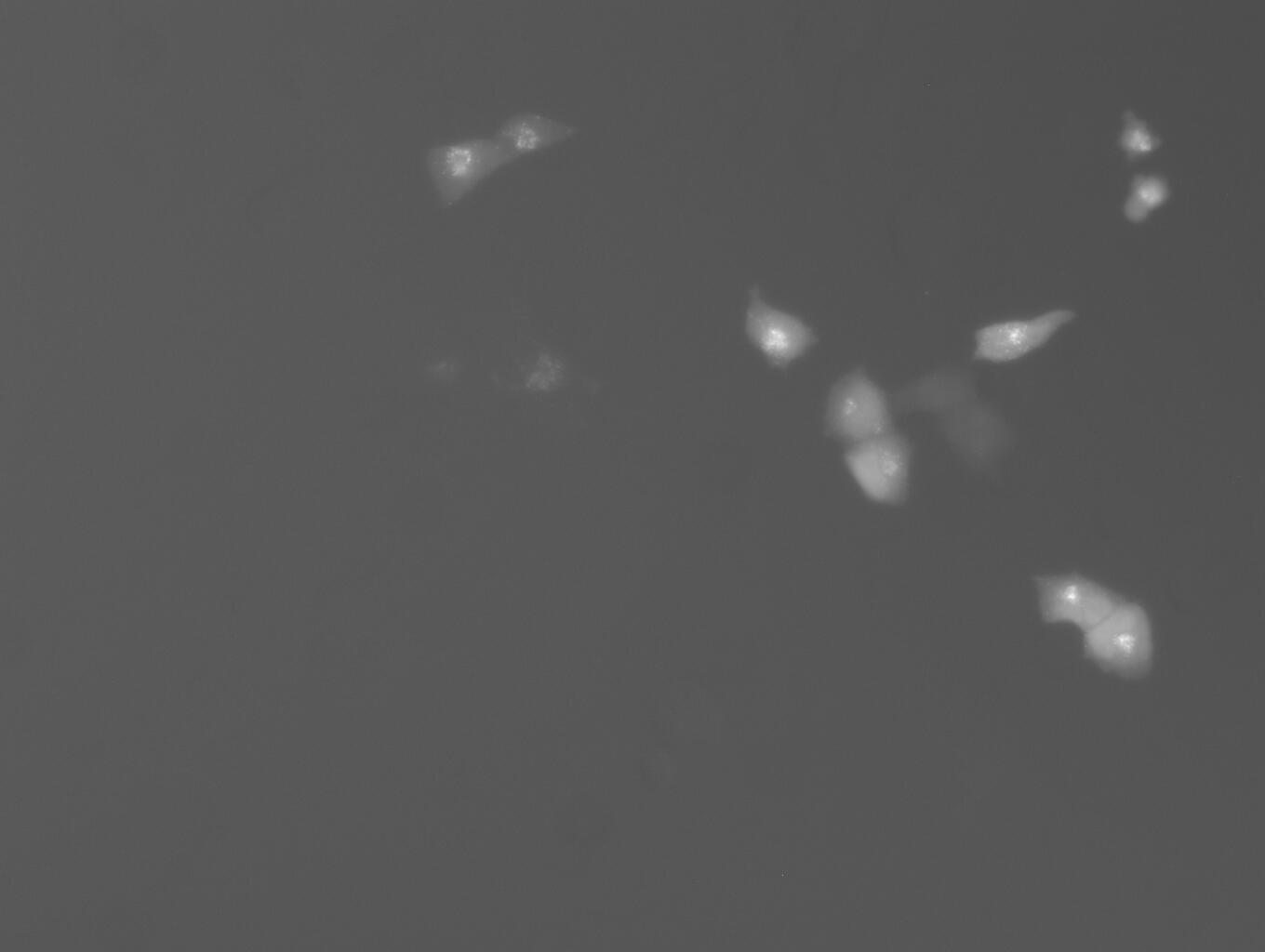

Supplement: Supplementary file 6 — Source data Fig. 4 [file 44318_2024_233_MOESM6_ESM.zip › 4A/6. DLK1 ConA.jpg]

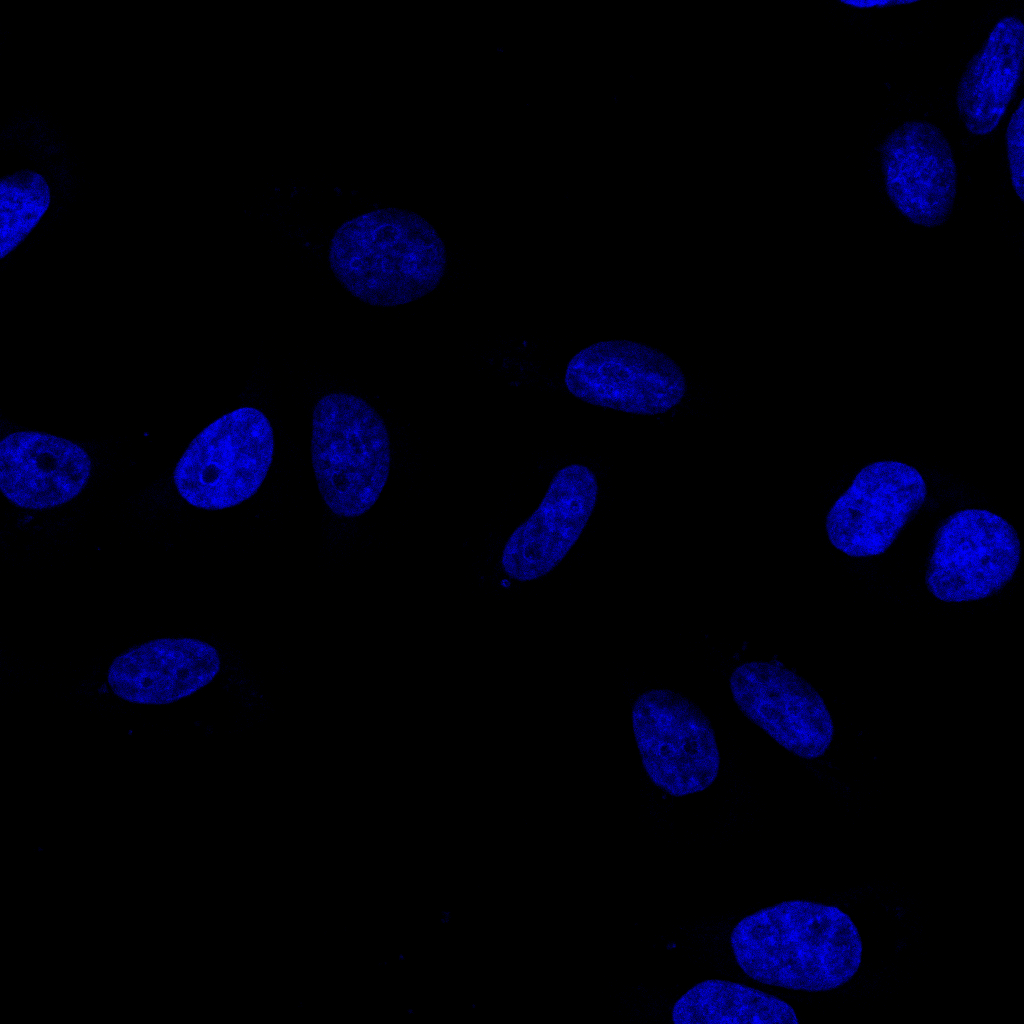

Supplement: Supplementary file 6 — Source data Fig. 4 [file 44318_2024_233_MOESM6_ESM.zip › 4B/Image/HeLa Ctrl Baf.A1 TGOLN2 GFP RFP ATG16L1_Series002_ch00_SV.tif]

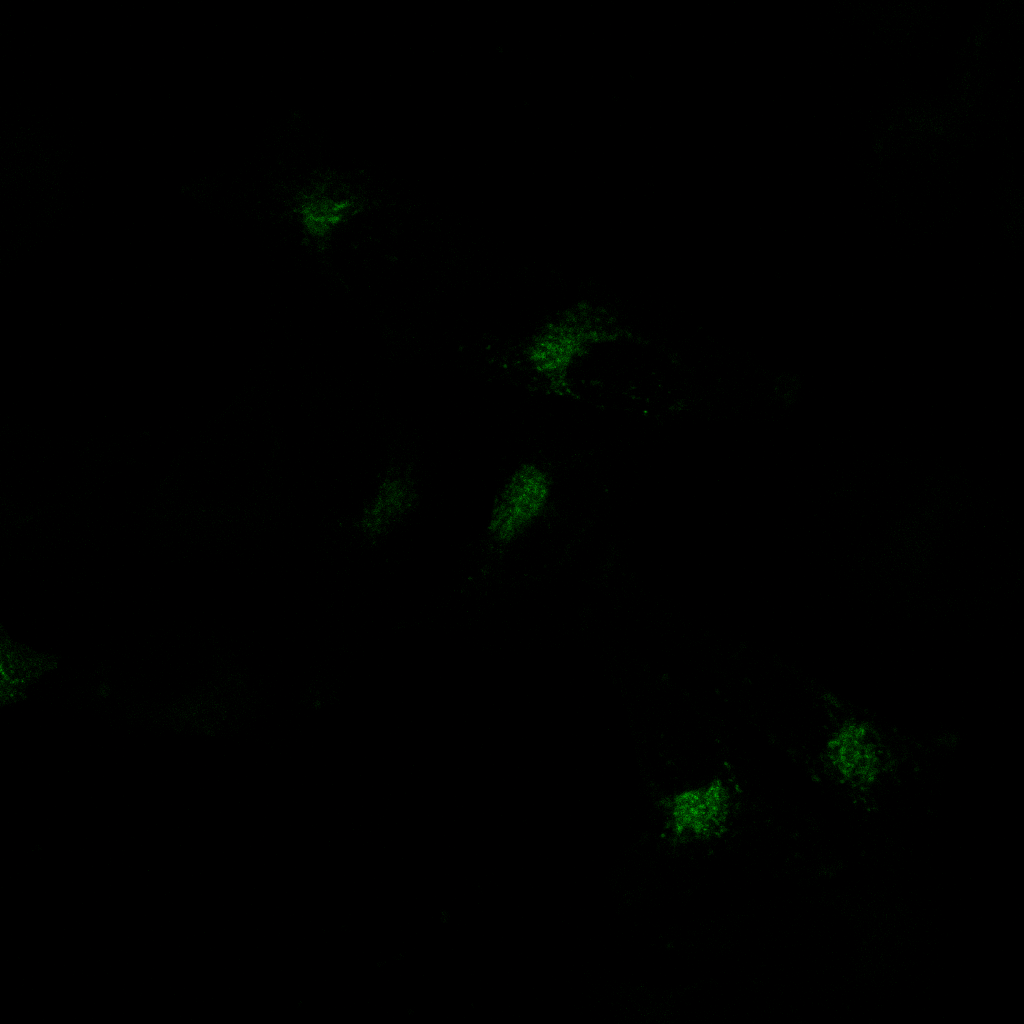

Supplement: Supplementary file 6 — Source data Fig. 4 [file 44318_2024_233_MOESM6_ESM.zip › 4B/Image/HeLa Ctrl Baf.A1 TGOLN2 GFP RFP ATG16L1_Series002_ch01_SV.tif]

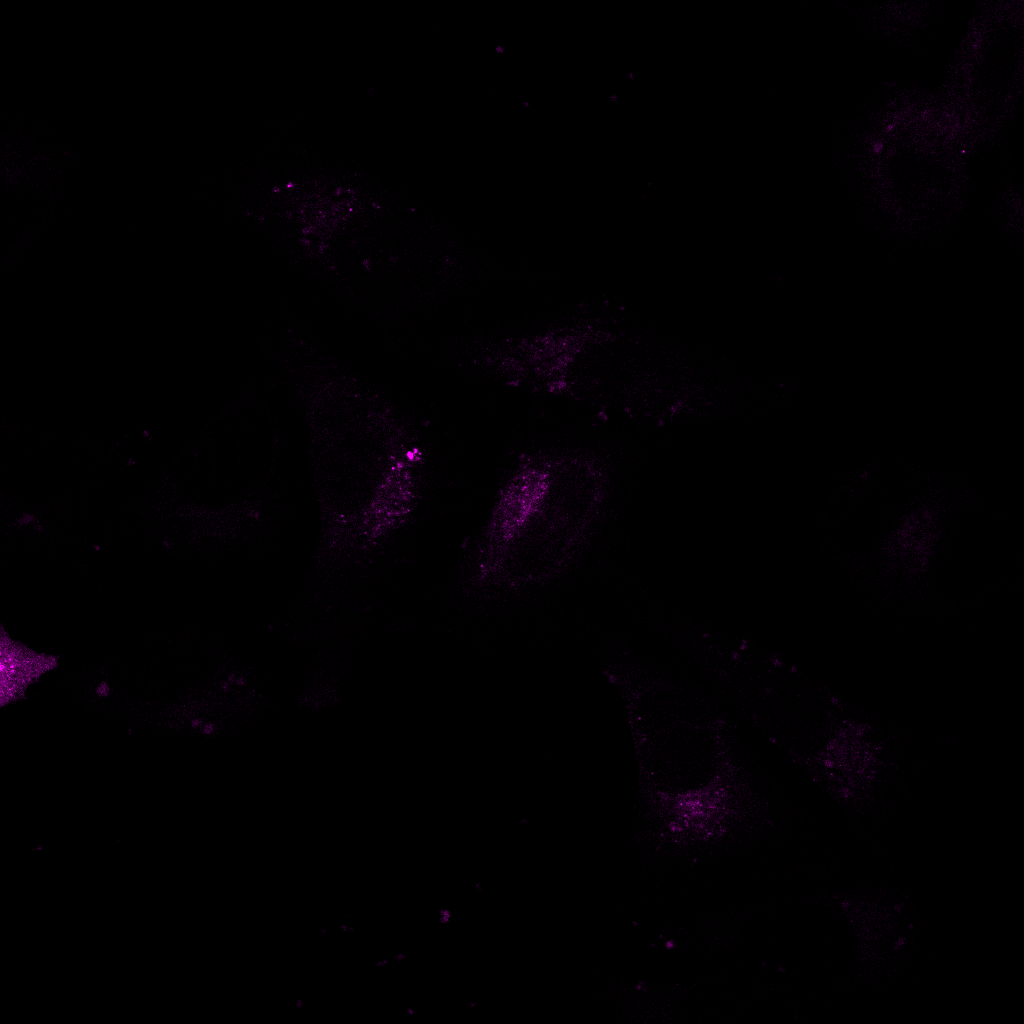

Supplement: Supplementary file 6 — Source data Fig. 4 [file 44318_2024_233_MOESM6_ESM.zip › 4B/Image/HeLa Ctrl Baf.A1 TGOLN2 GFP RFP ATG16L1_Series002_ch02_SV.tif]

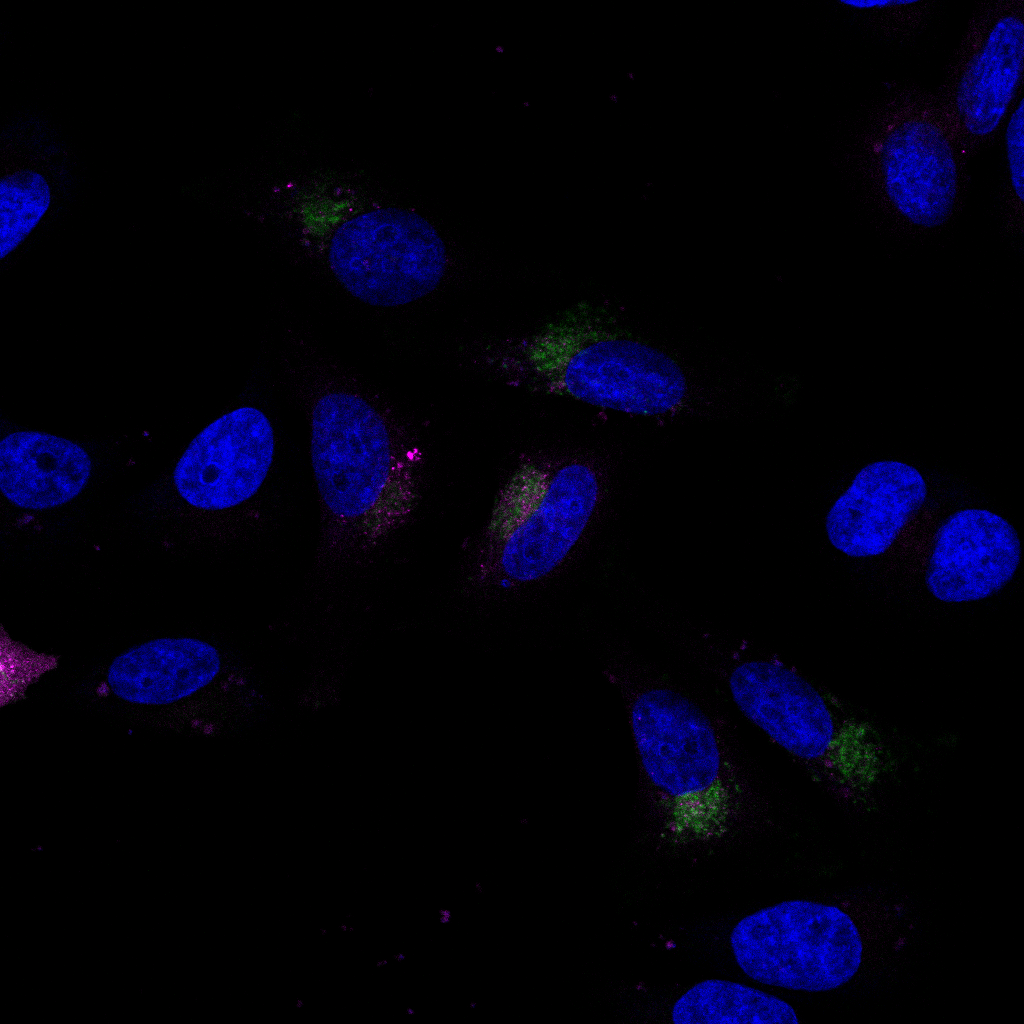

Supplement: Supplementary file 6 — Source data Fig. 4 [file 44318_2024_233_MOESM6_ESM.zip › 4B/Image/HeLa Ctrl Baf.A1 TGOLN2 GFP RFP ATG16L1_Series002_overlay.tif]

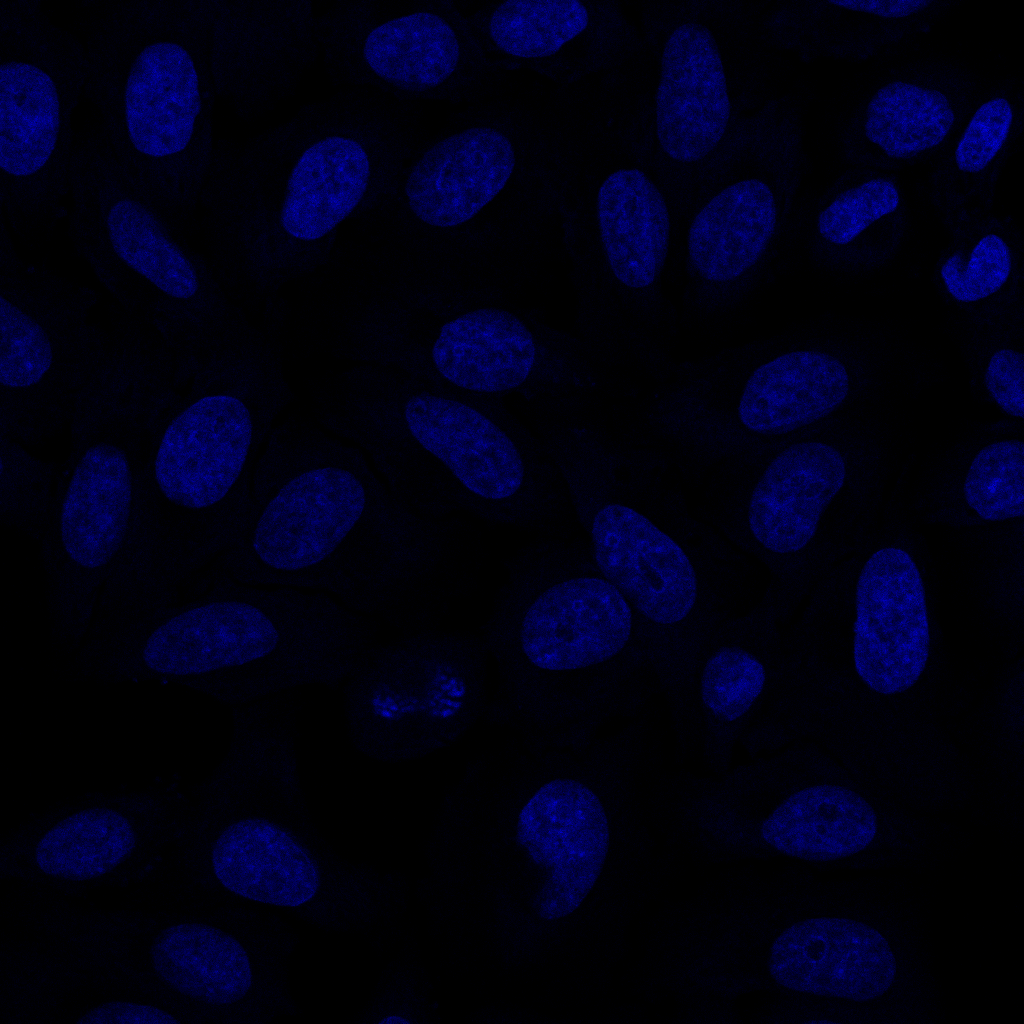

Supplement: Supplementary file 6 — Source data Fig. 4 [file 44318_2024_233_MOESM6_ESM.zip › 4B/Image/HeLa Ctrl Veh TGOLN2 GFP RFP ATG16L1_Series003_ch00_SV.tif]

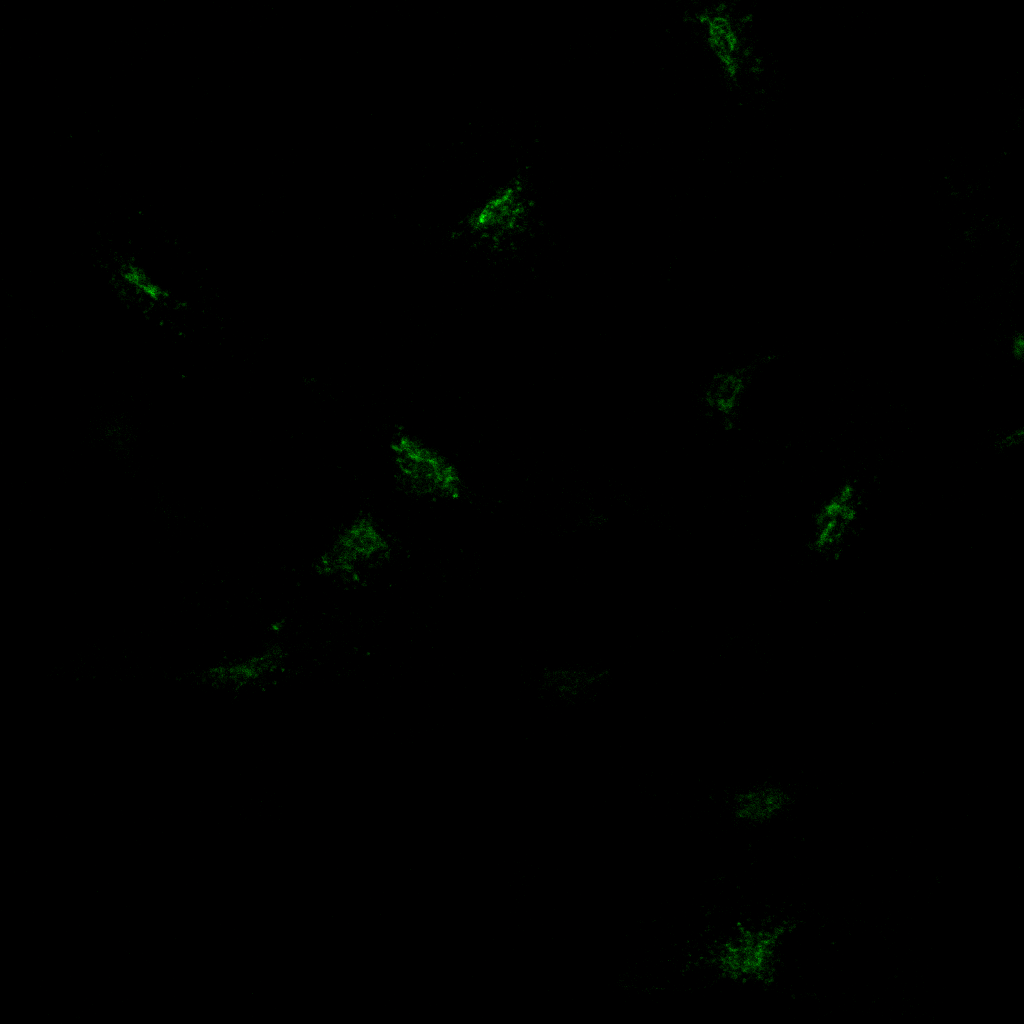

Supplement: Supplementary file 6 — Source data Fig. 4 [file 44318_2024_233_MOESM6_ESM.zip › 4B/Image/HeLa Ctrl Veh TGOLN2 GFP RFP ATG16L1_Series003_ch01_SV.tif]

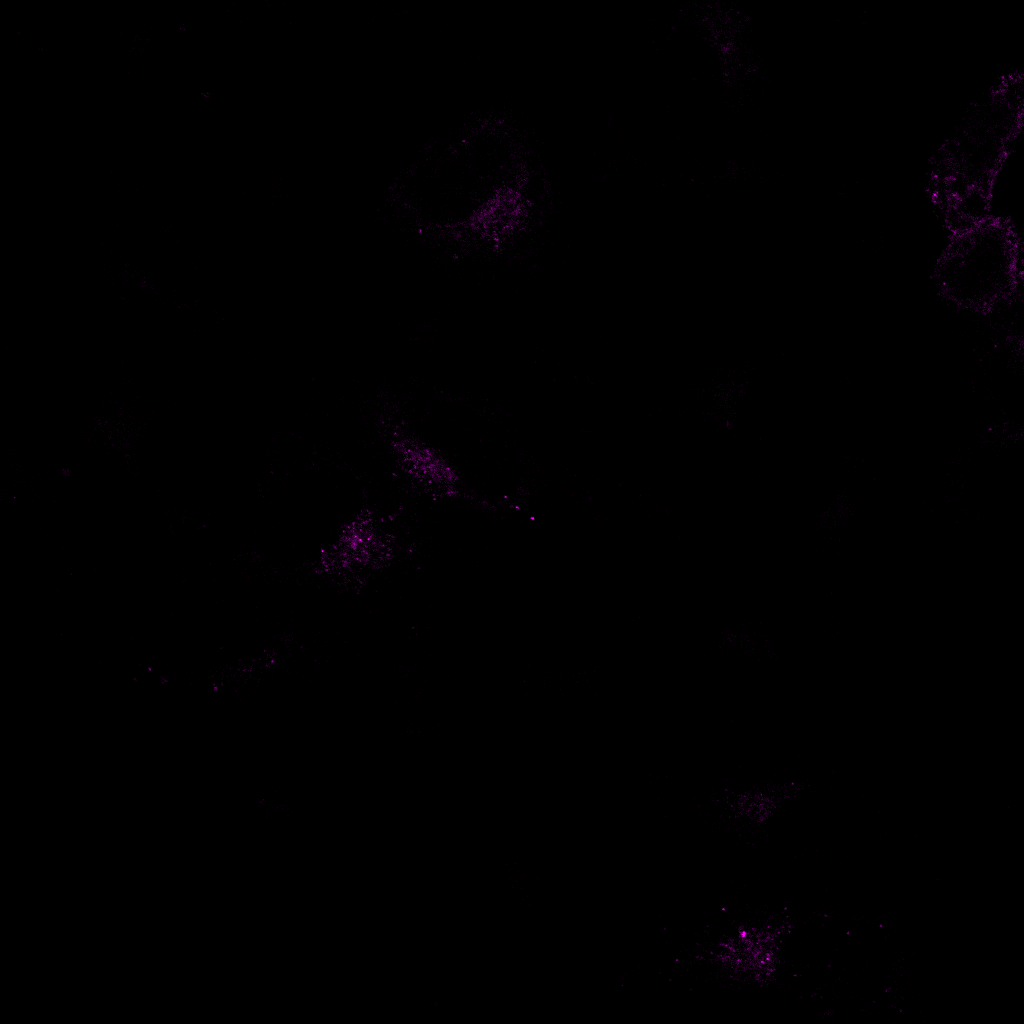

Supplement: Supplementary file 6 — Source data Fig. 4 [file 44318_2024_233_MOESM6_ESM.zip › 4B/Image/HeLa Ctrl Veh TGOLN2 GFP RFP ATG16L1_Series003_ch02_SV.tif]

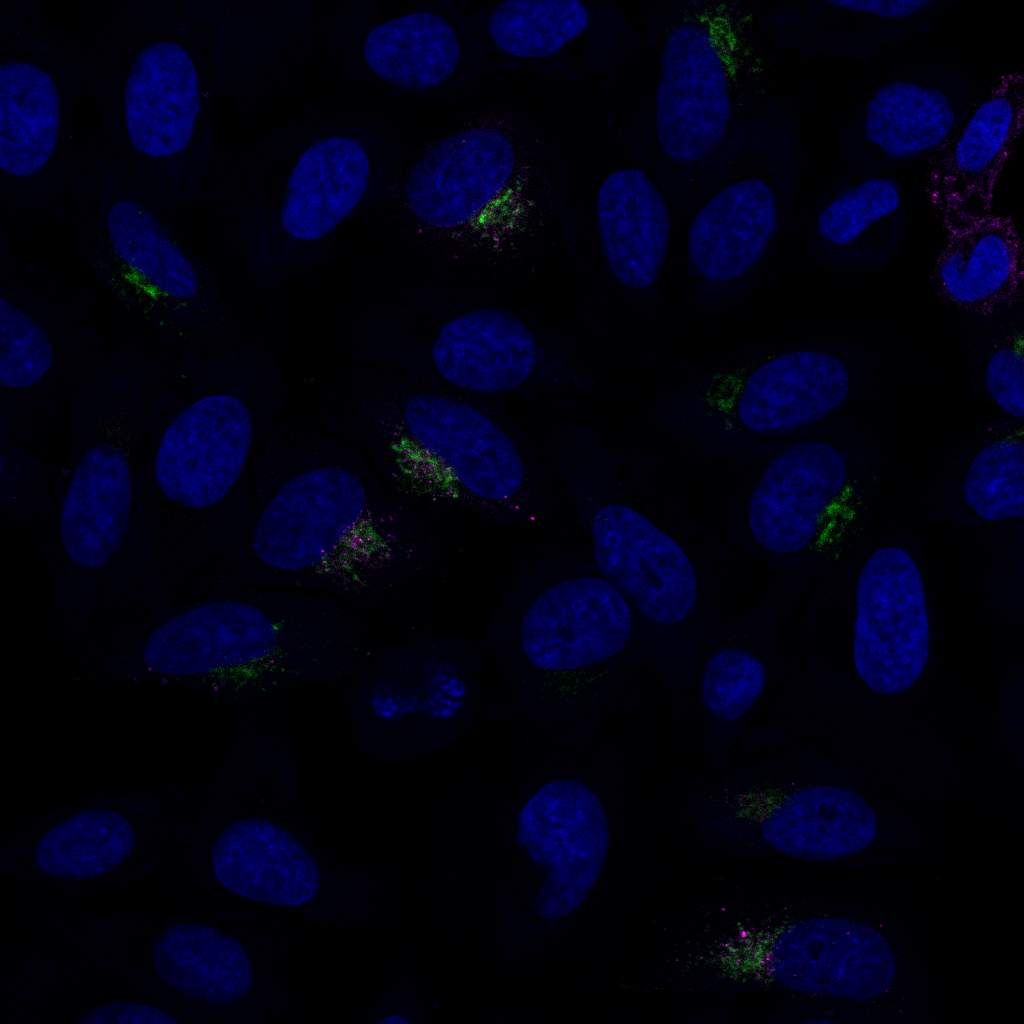

Supplement: Supplementary file 6 — Source data Fig. 4 [file 44318_2024_233_MOESM6_ESM.zip › 4B/Image/HeLa Ctrl Veh TGOLN2 GFP RFP ATG16L1_Series003_overlay.tif]

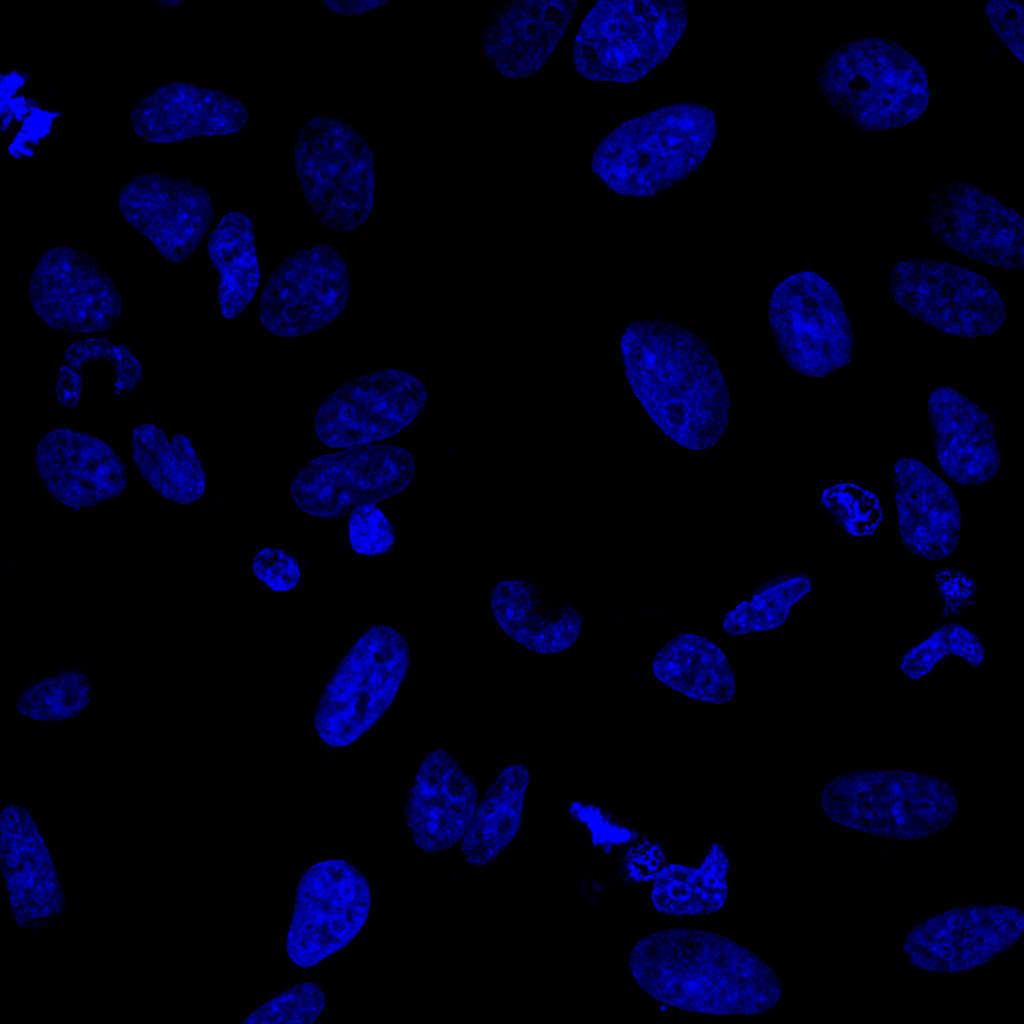

Supplement: Supplementary file 6 — Source data Fig. 4 [file 44318_2024_233_MOESM6_ESM.zip › 4B/Image/HeLa DLK1 Baf.A1 TGOLN2 GFP RFP ATG16L1_Series010_ch00_SV.tif]

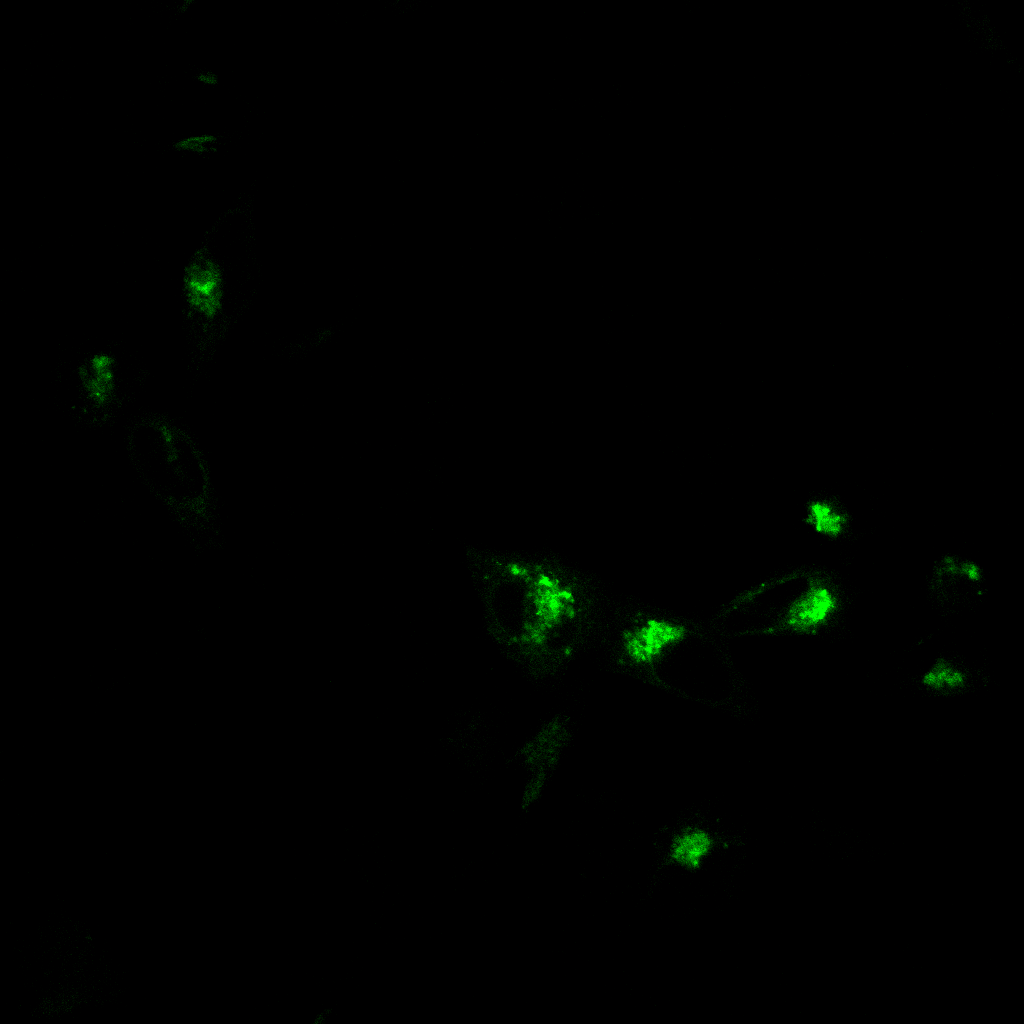

Supplement: Supplementary file 6 — Source data Fig. 4 [file 44318_2024_233_MOESM6_ESM.zip › 4B/Image/HeLa DLK1 Baf.A1 TGOLN2 GFP RFP ATG16L1_Series010_ch01_SV.tif]

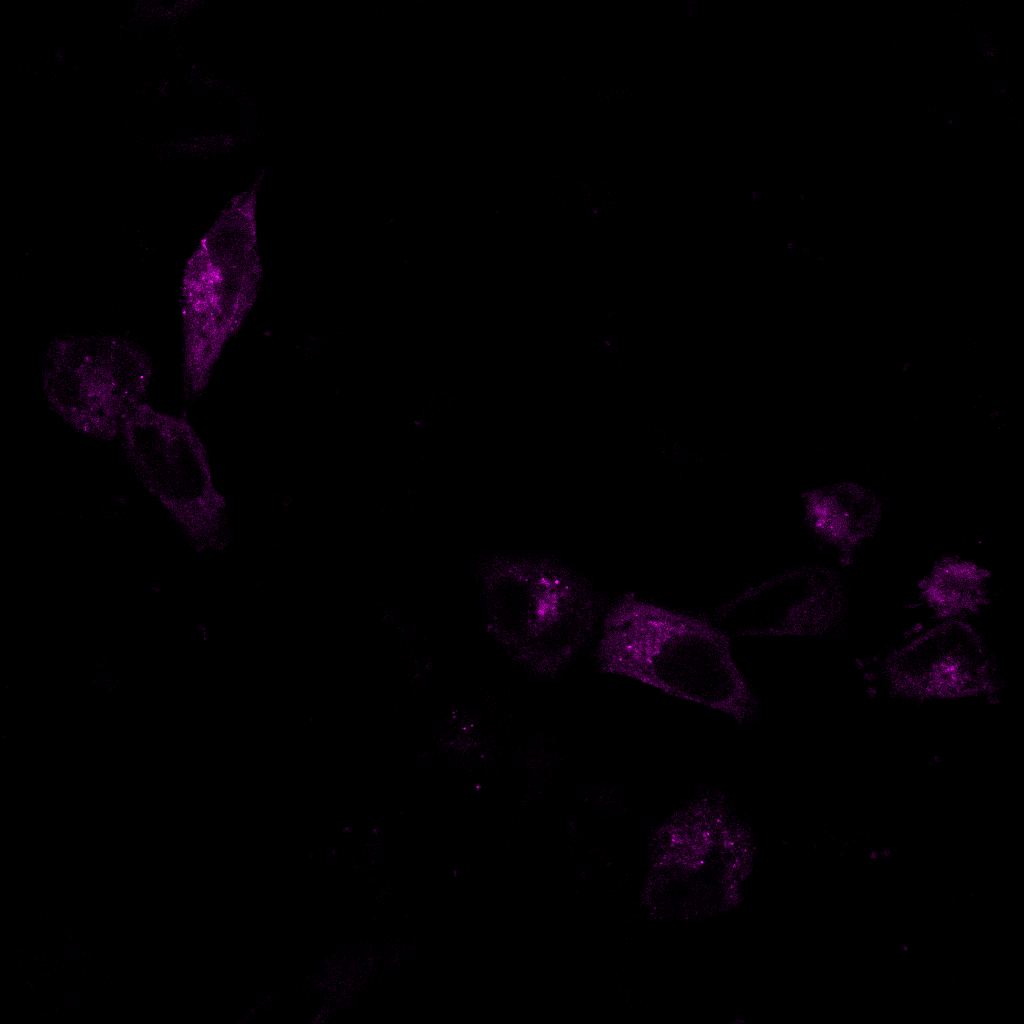

Supplement: Supplementary file 6 — Source data Fig. 4 [file 44318_2024_233_MOESM6_ESM.zip › 4B/Image/HeLa DLK1 Baf.A1 TGOLN2 GFP RFP ATG16L1_Series010_ch02_SV.tif]

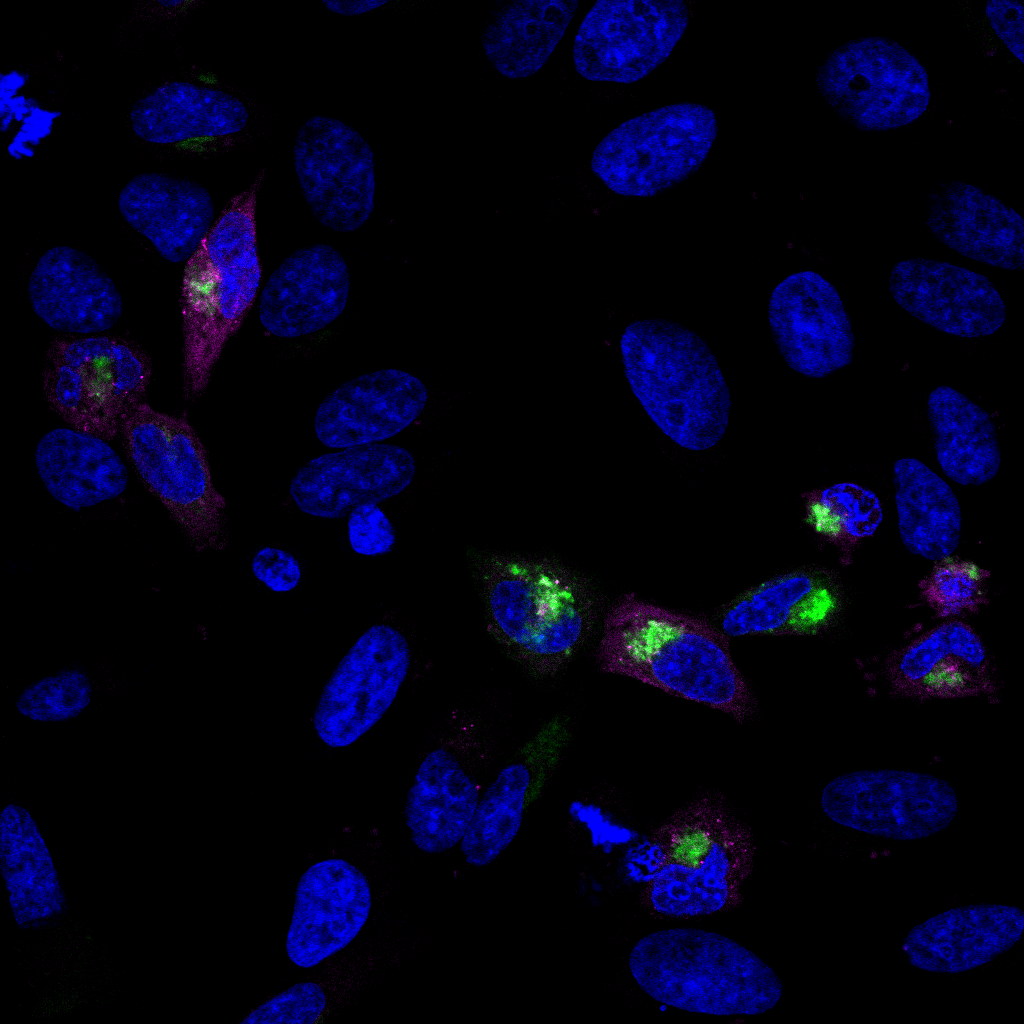

Supplement: Supplementary file 6 — Source data Fig. 4 [file 44318_2024_233_MOESM6_ESM.zip › 4B/Image/HeLa DLK1 Baf.A1 TGOLN2 GFP RFP ATG16L1_Series010_overlay.tif]

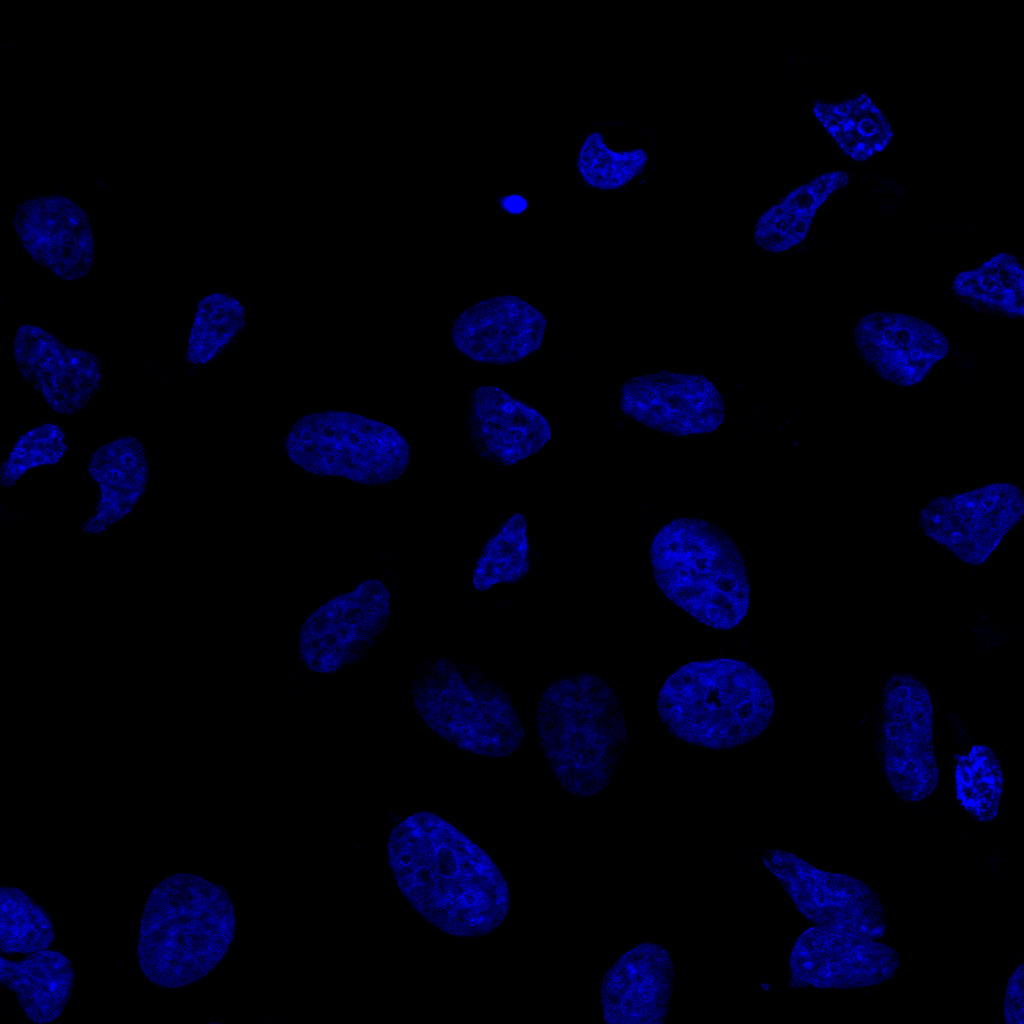

Supplement: Supplementary file 6 — Source data Fig. 4 [file 44318_2024_233_MOESM6_ESM.zip › 4B/Image/HeLa DLK1 Veh TGOLN2 GFP RFP ATG16L1_Series009_ch00_SV.tif]

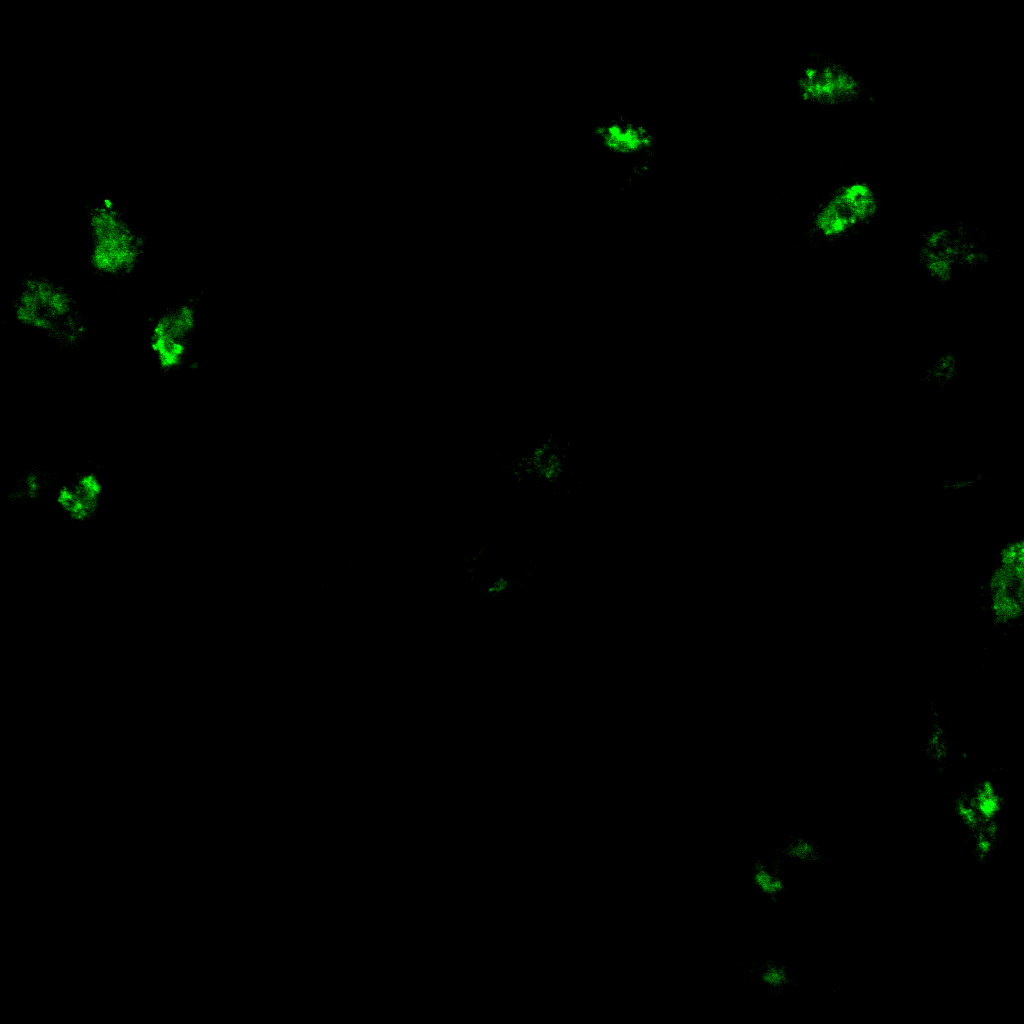

Supplement: Supplementary file 6 — Source data Fig. 4 [file 44318_2024_233_MOESM6_ESM.zip › 4B/Image/HeLa DLK1 Veh TGOLN2 GFP RFP ATG16L1_Series009_ch01_SV.tif]

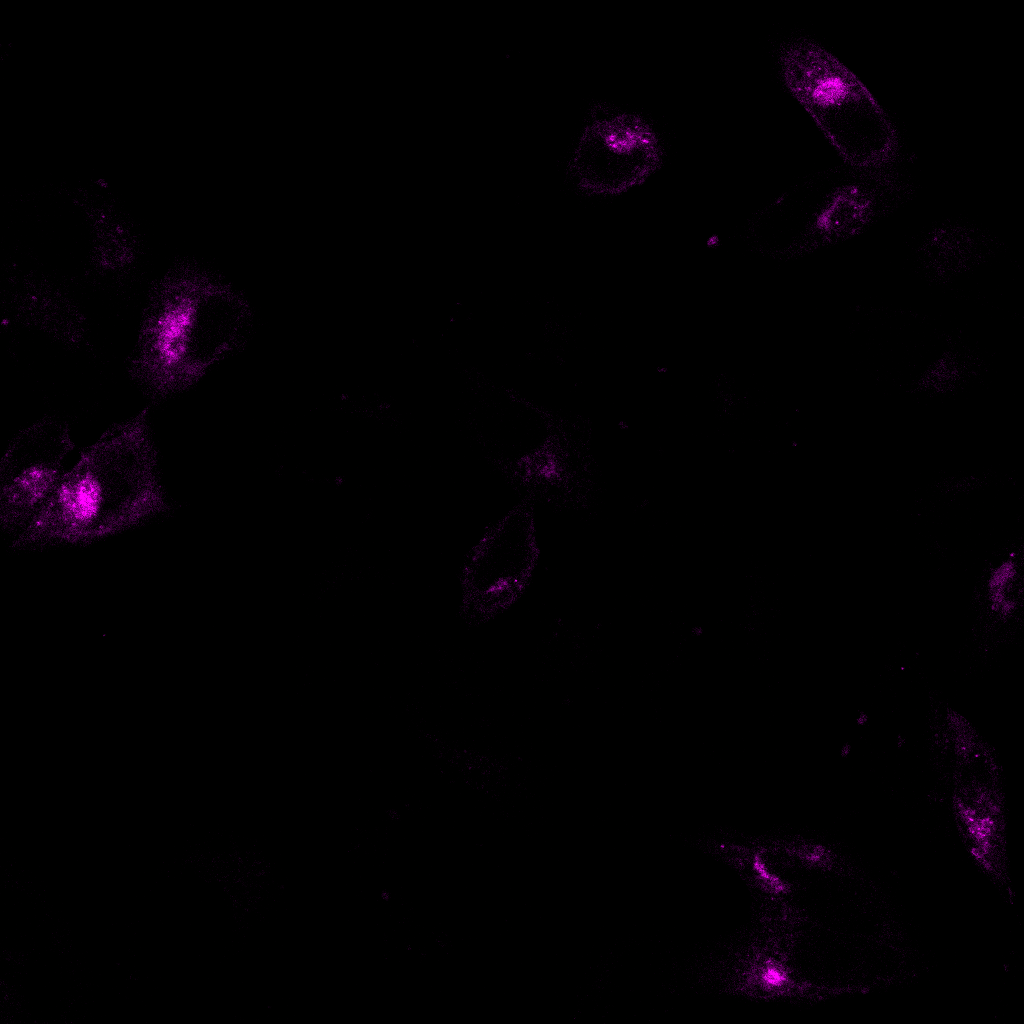

Supplement: Supplementary file 6 — Source data Fig. 4 [file 44318_2024_233_MOESM6_ESM.zip › 4B/Image/HeLa DLK1 Veh TGOLN2 GFP RFP ATG16L1_Series009_ch02_SV.tif]

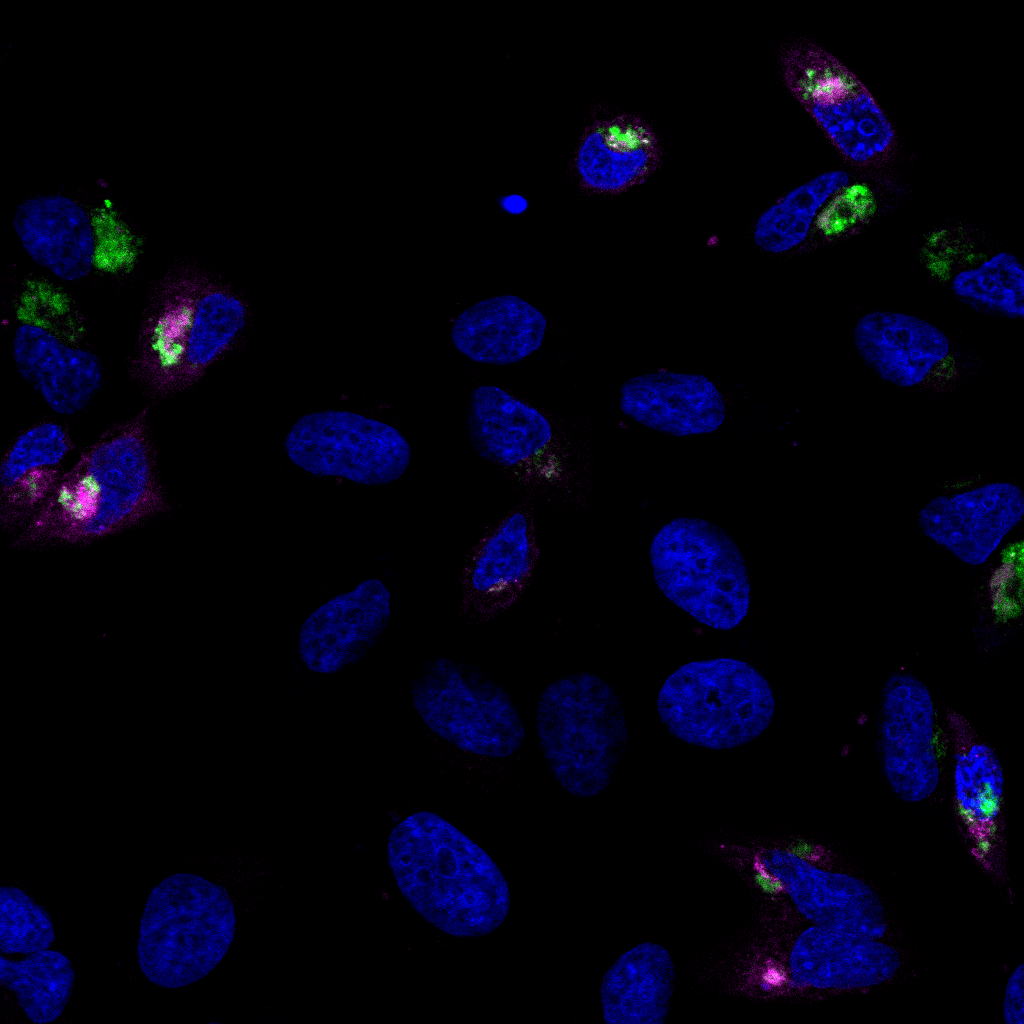

Supplement: Supplementary file 6 — Source data Fig. 4 [file 44318_2024_233_MOESM6_ESM.zip › 4B/Image/HeLa DLK1 Veh TGOLN2 GFP RFP ATG16L1_Series009_overlay.tif]

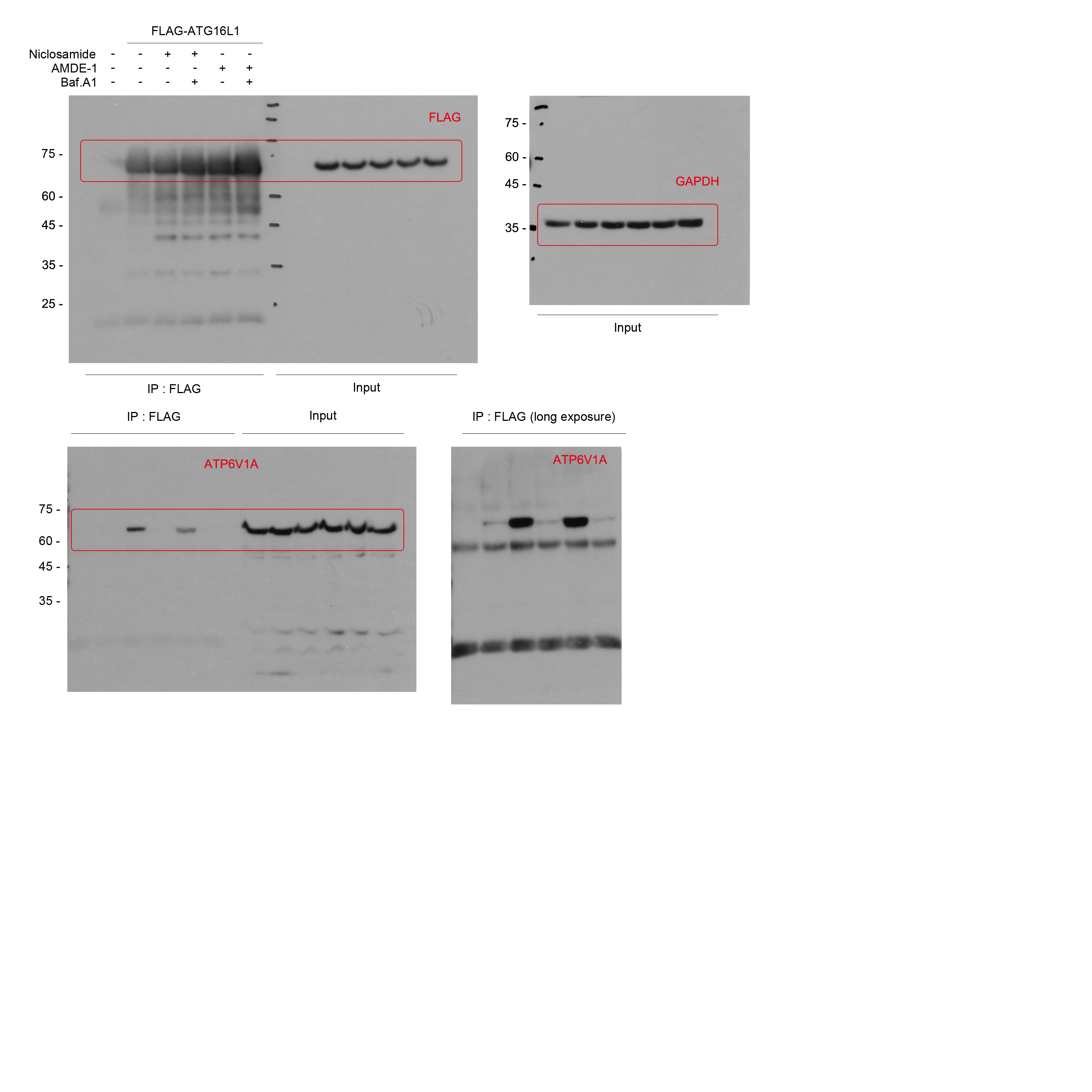

Supplement: Supplementary file 6 — Source data Fig. 4 [file 44318_2024_233_MOESM6_ESM.zip › 4C/Figure 4C.png]

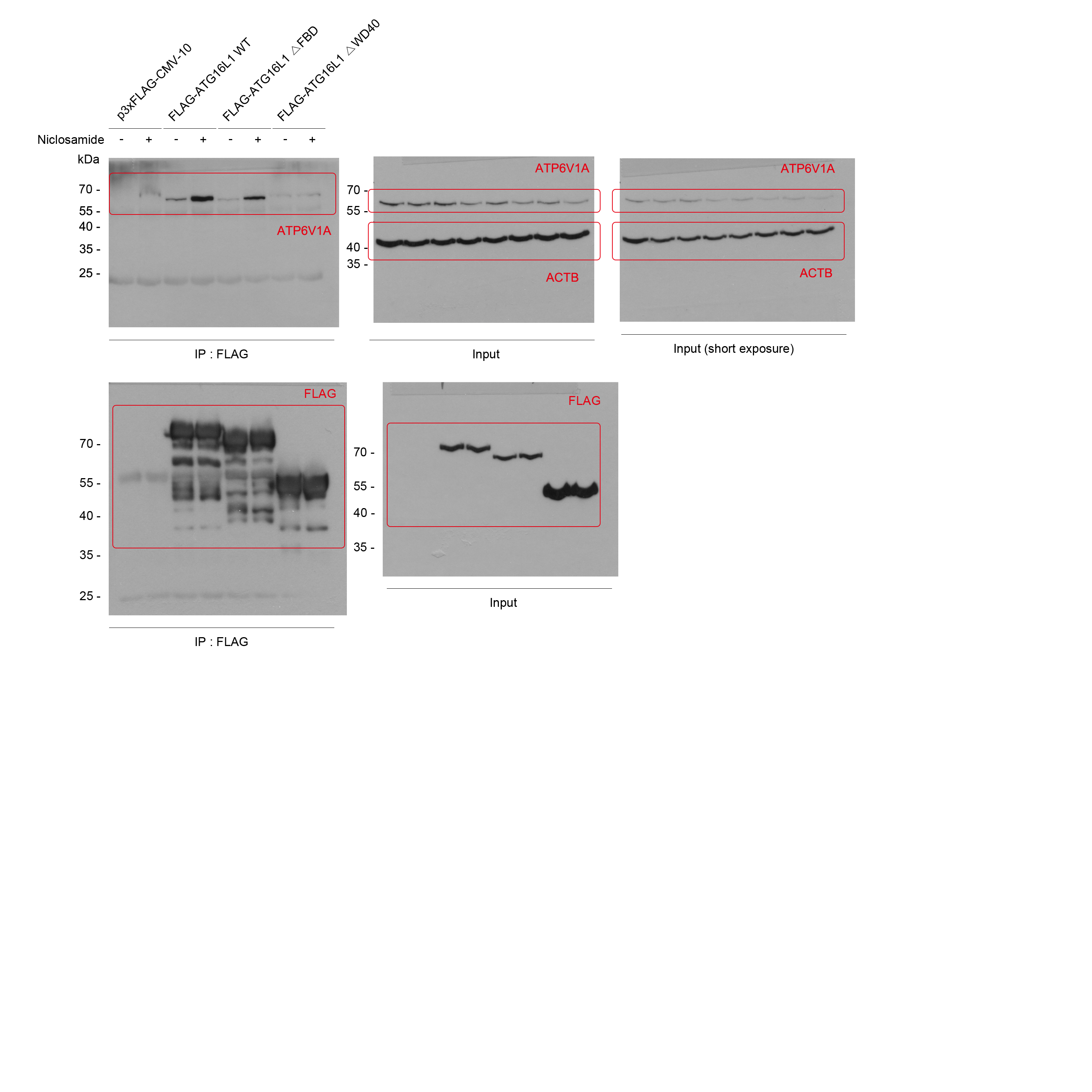

Supplement: Supplementary file 6 — Source data Fig. 4 [file 44318_2024_233_MOESM6_ESM.zip › 4D/Figure 4D.png]

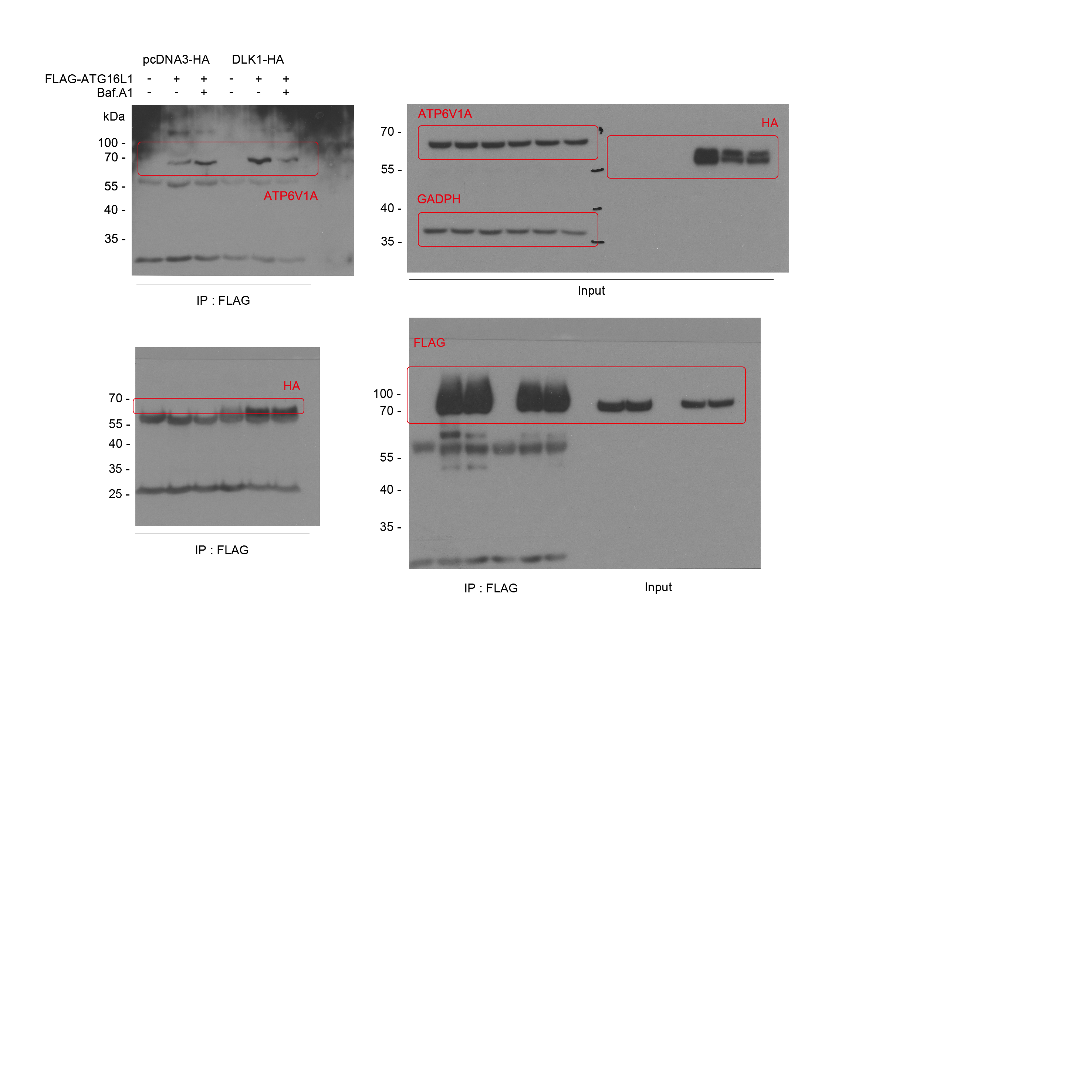

Supplement: Supplementary file 6 — Source data Fig. 4 [file 44318_2024_233_MOESM6_ESM.zip › 4E/Figure 4E.png]

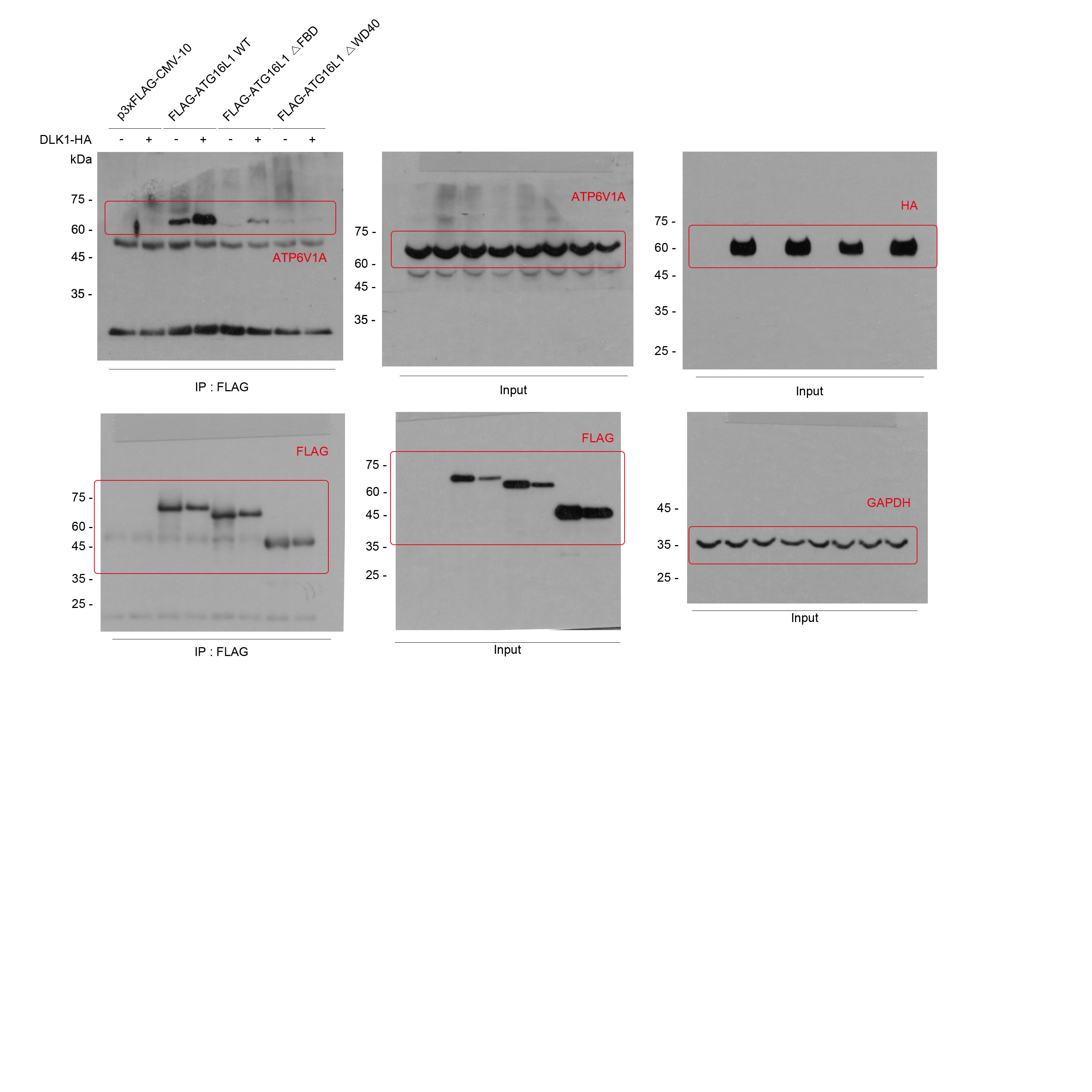

Supplement: Supplementary file 6 — Source data Fig. 4 [file 44318_2024_233_MOESM6_ESM.zip › 4F/Figure 4F.png]

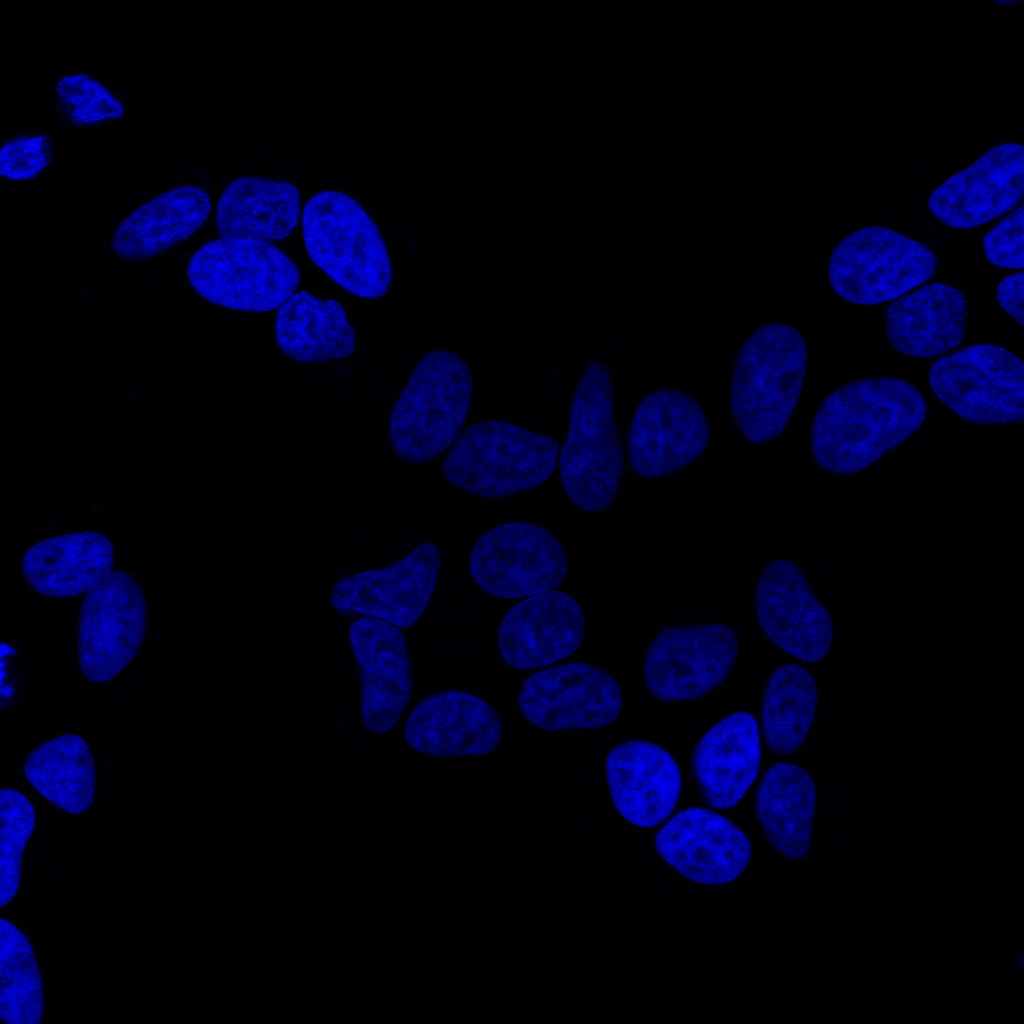

Supplement: Supplementary file 6 — Source data Fig. 4 [file 44318_2024_233_MOESM6_ESM.zip › 4G/Image/Ctrl WT RFP ATG16L1 TGOLN2 GFP_Series008_ch00_SV.tif]

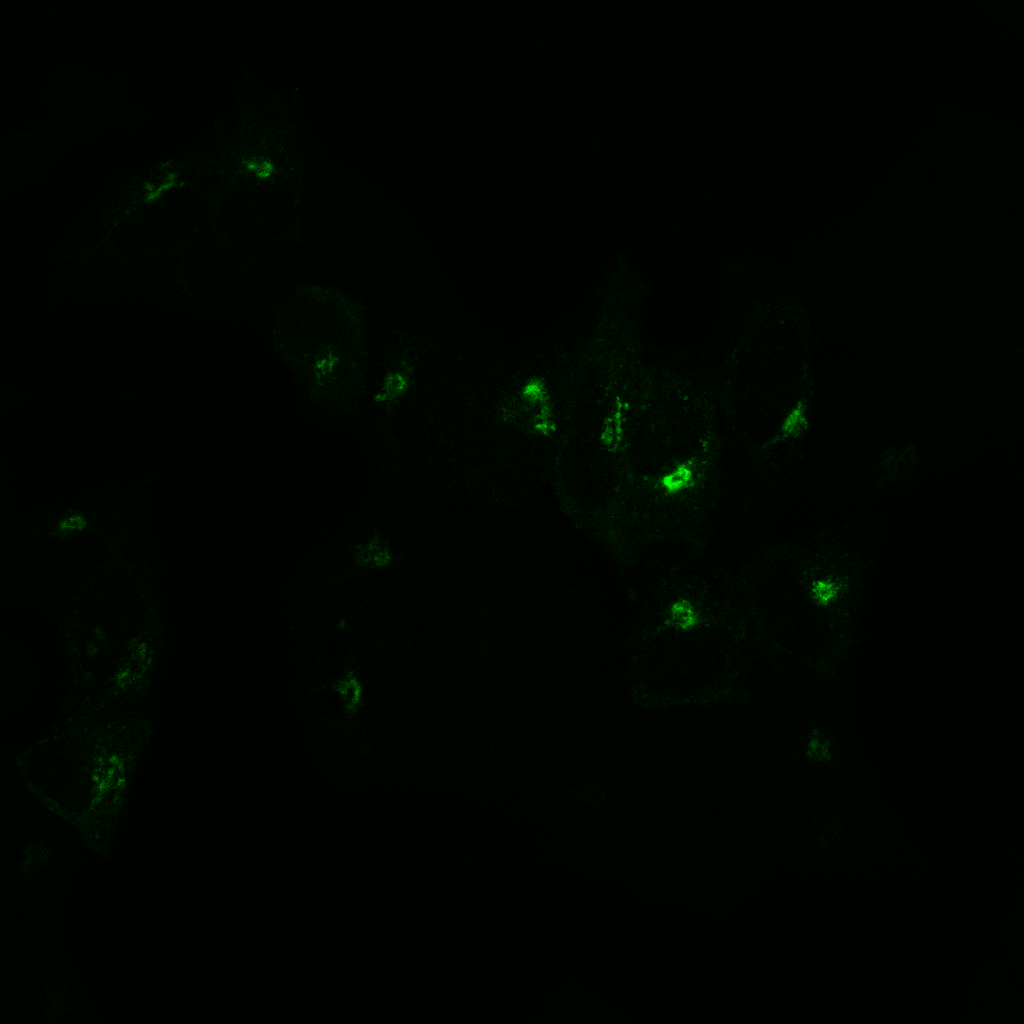

Supplement: Supplementary file 6 — Source data Fig. 4 [file 44318_2024_233_MOESM6_ESM.zip › 4G/Image/Ctrl WT RFP ATG16L1 TGOLN2 GFP_Series008_ch01_SV.tif]

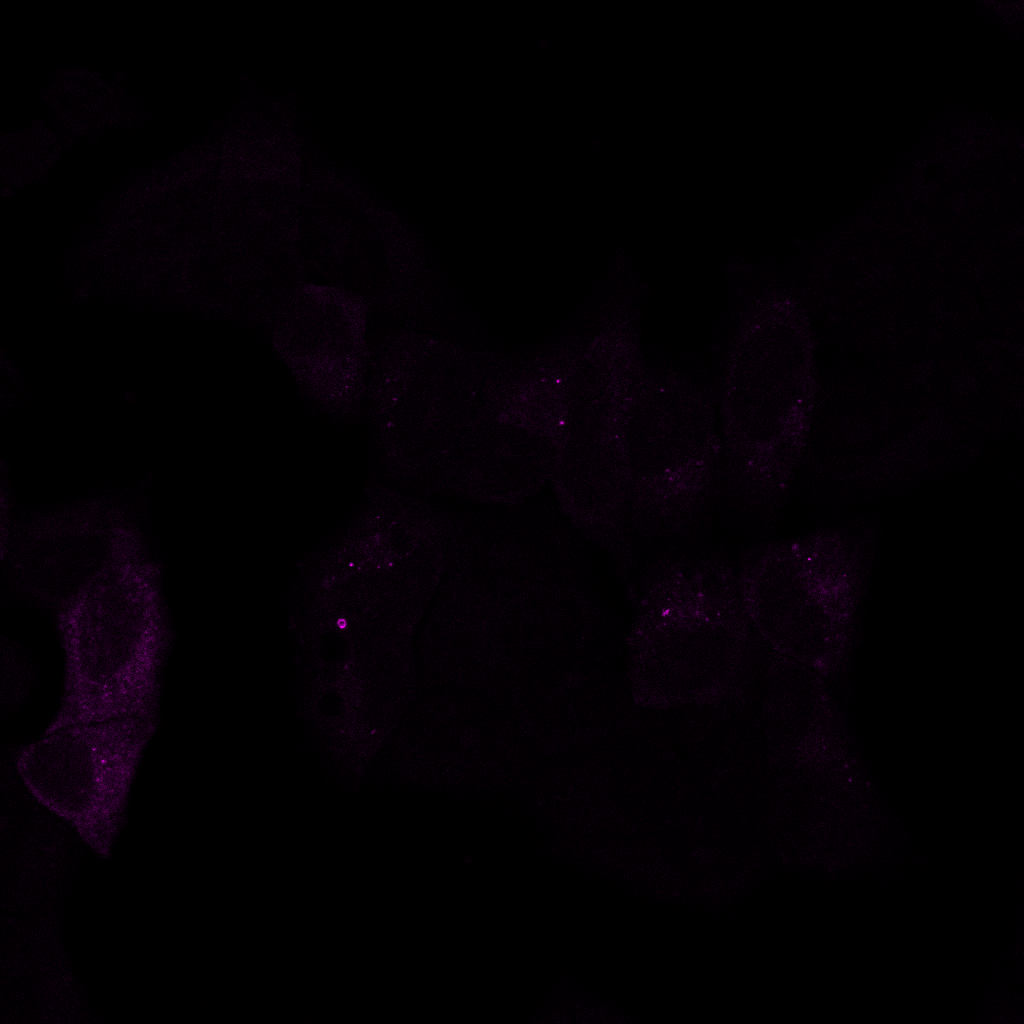

Supplement: Supplementary file 6 — Source data Fig. 4 [file 44318_2024_233_MOESM6_ESM.zip › 4G/Image/Ctrl WT RFP ATG16L1 TGOLN2 GFP_Series008_ch02_SV.tif]

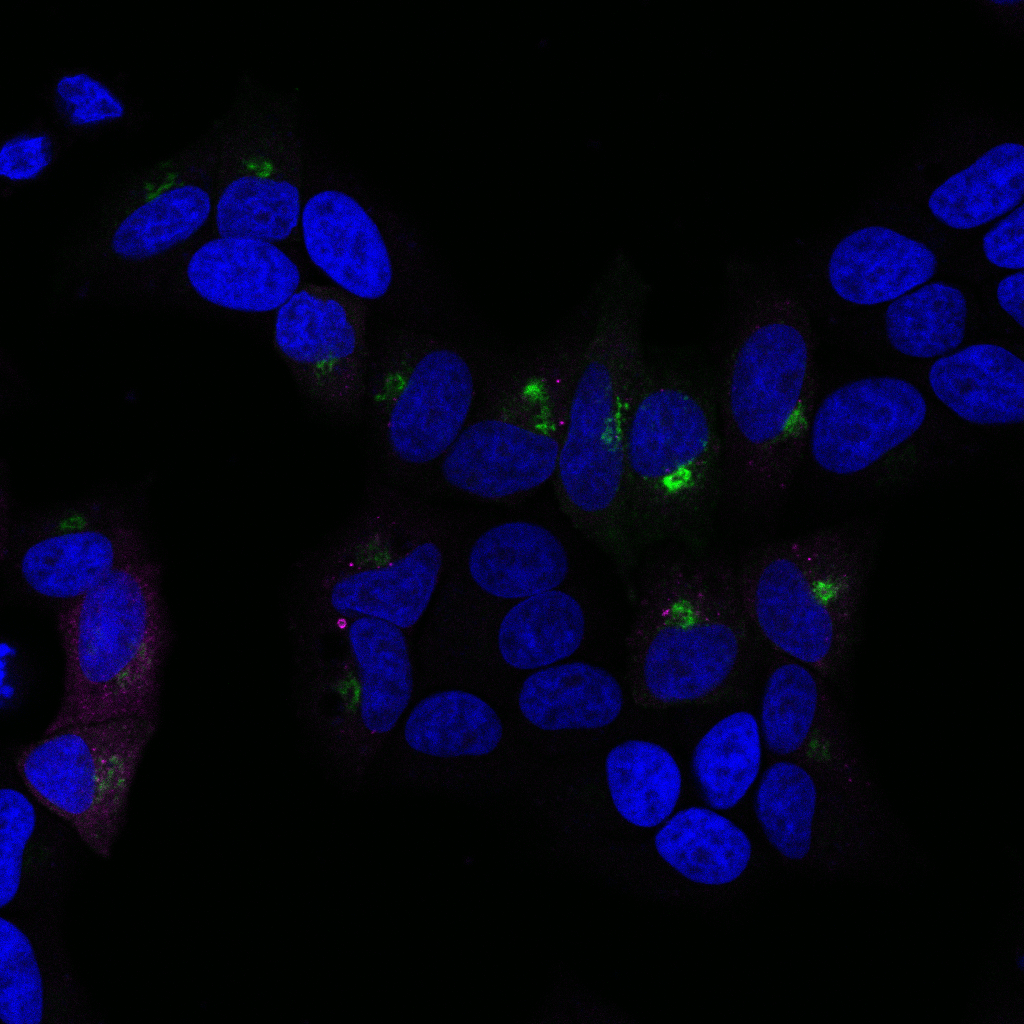

Supplement: Supplementary file 6 — Source data Fig. 4 [file 44318_2024_233_MOESM6_ESM.zip › 4G/Image/Ctrl WT RFP ATG16L1 TGOLN2 GFP_Series008_overlay.tif]

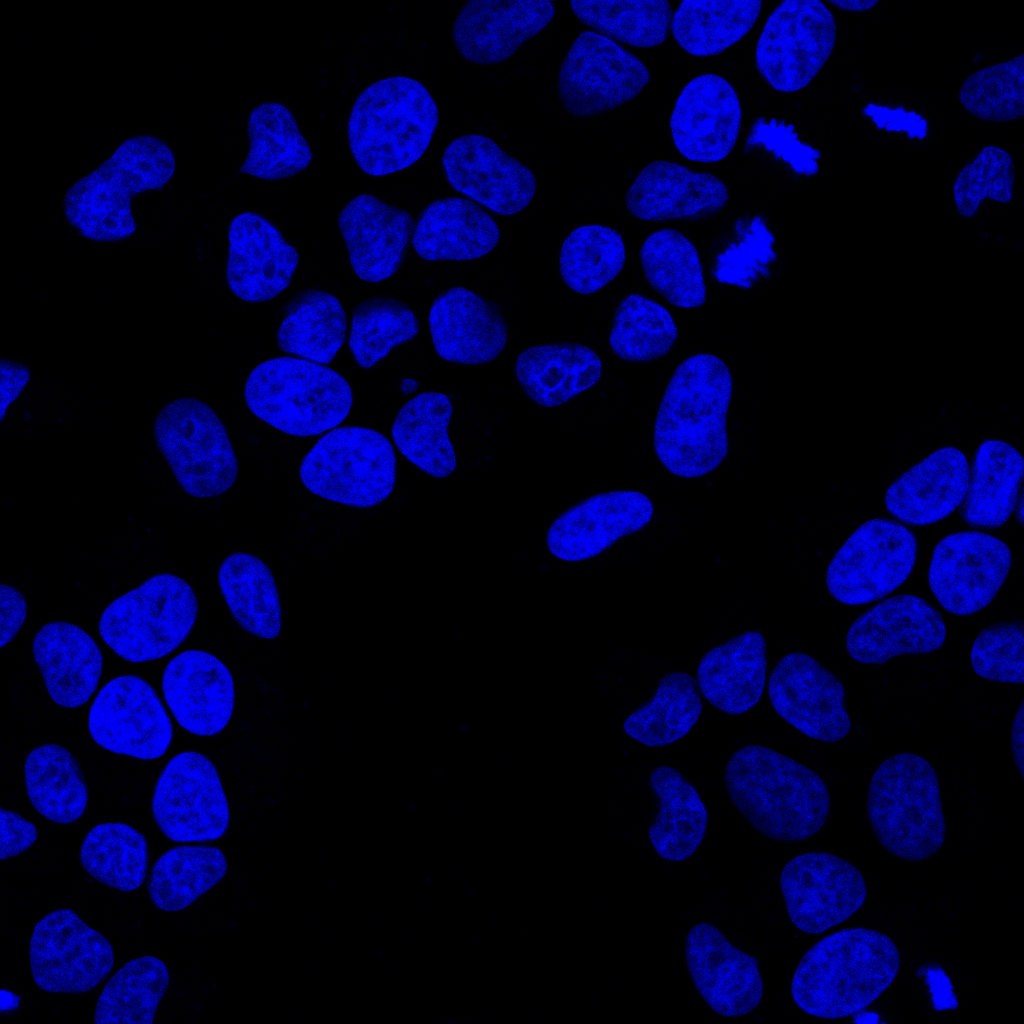

Supplement: Supplementary file 6 — Source data Fig. 4 [file 44318_2024_233_MOESM6_ESM.zip › 4G/Image/DLK1 ATG5-BD RFP ATG16L1 TGOLN2 GFP_Series012_ch00_SV.tif]

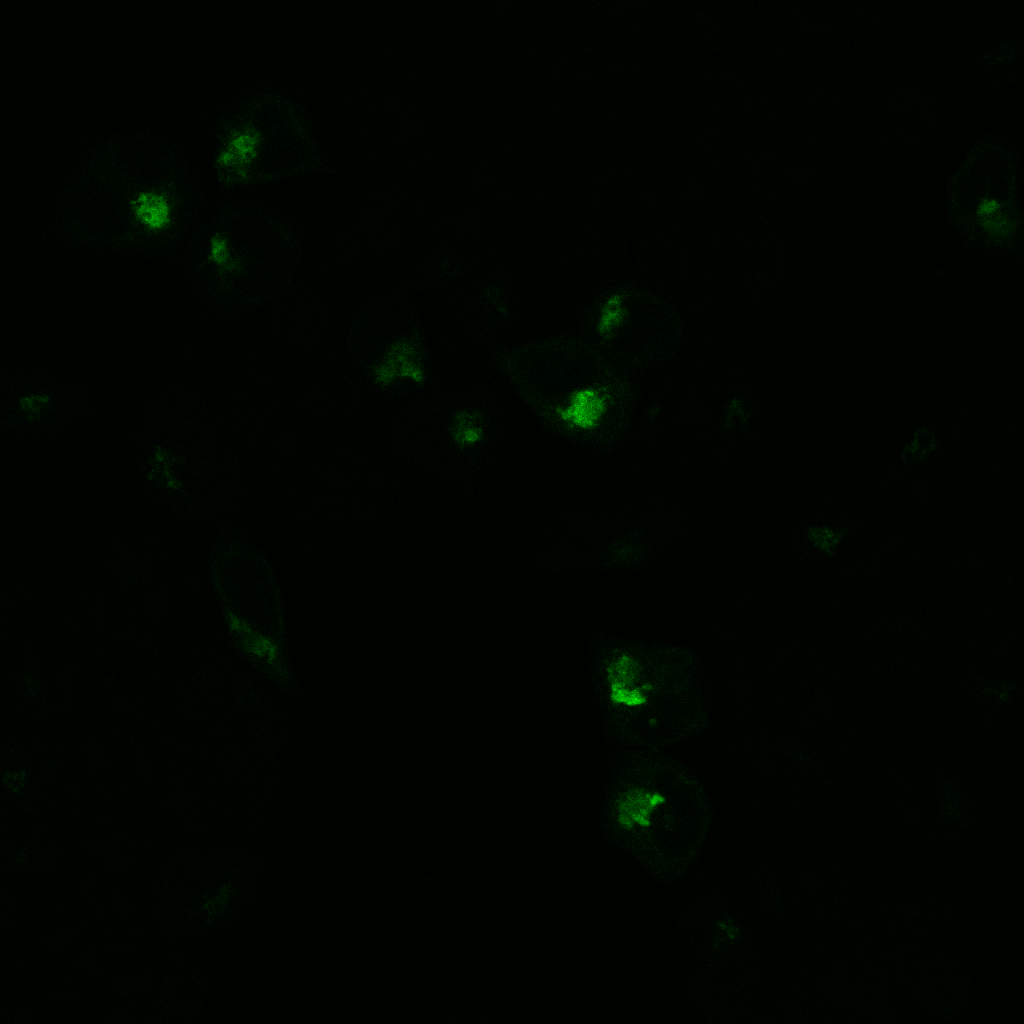

Supplement: Supplementary file 6 — Source data Fig. 4 [file 44318_2024_233_MOESM6_ESM.zip › 4G/Image/DLK1 ATG5-BD RFP ATG16L1 TGOLN2 GFP_Series012_ch01_SV.tif]

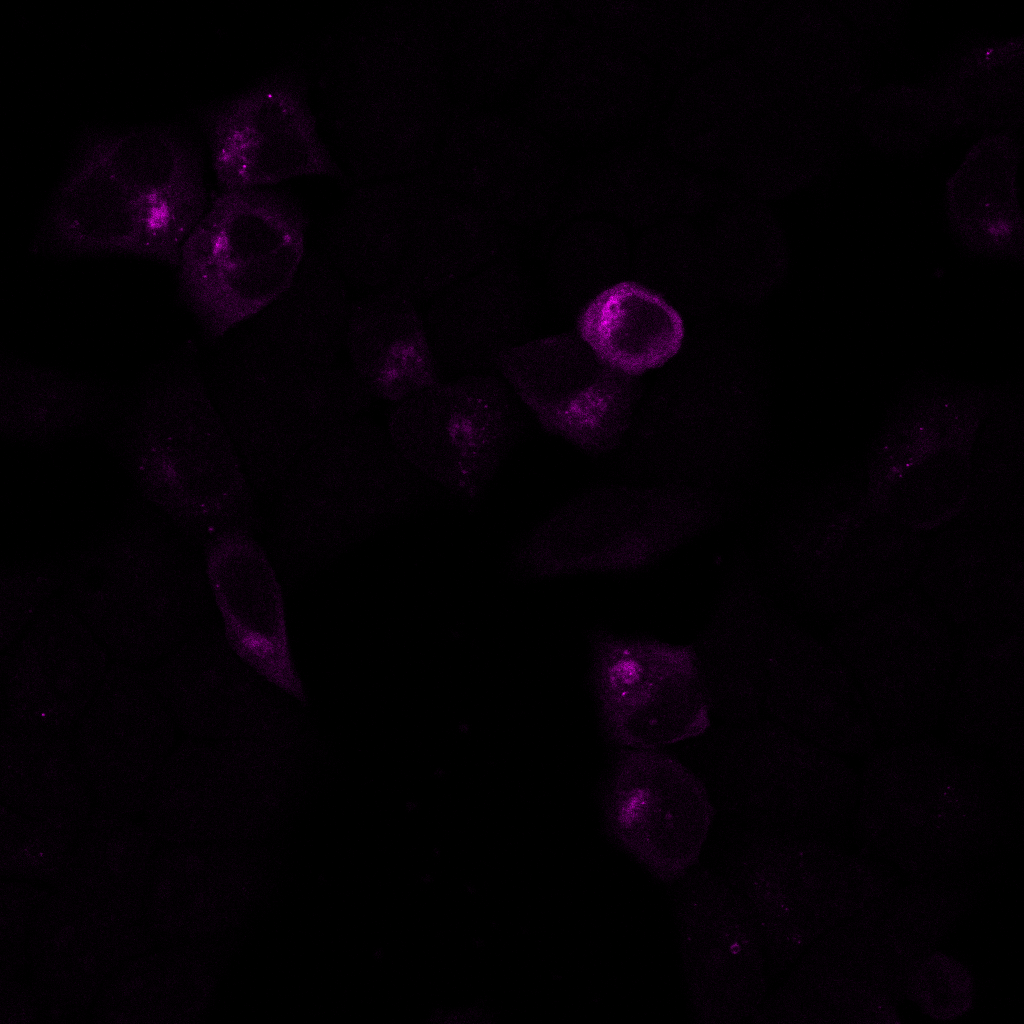

Supplement: Supplementary file 6 — Source data Fig. 4 [file 44318_2024_233_MOESM6_ESM.zip › 4G/Image/DLK1 ATG5-BD RFP ATG16L1 TGOLN2 GFP_Series012_ch02_SV.tif]

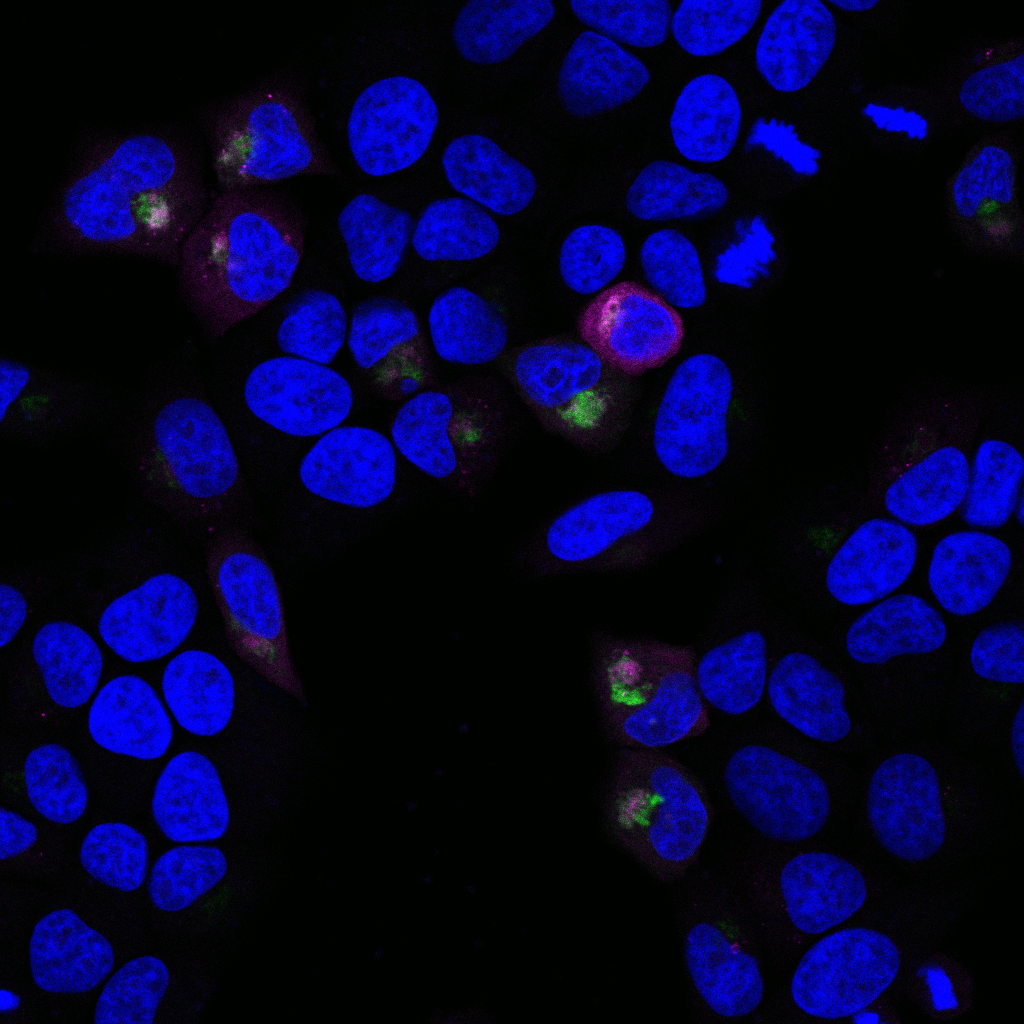

Supplement: Supplementary file 6 — Source data Fig. 4 [file 44318_2024_233_MOESM6_ESM.zip › 4G/Image/DLK1 ATG5-BD RFP ATG16L1 TGOLN2 GFP_Series012_overlay.tif]

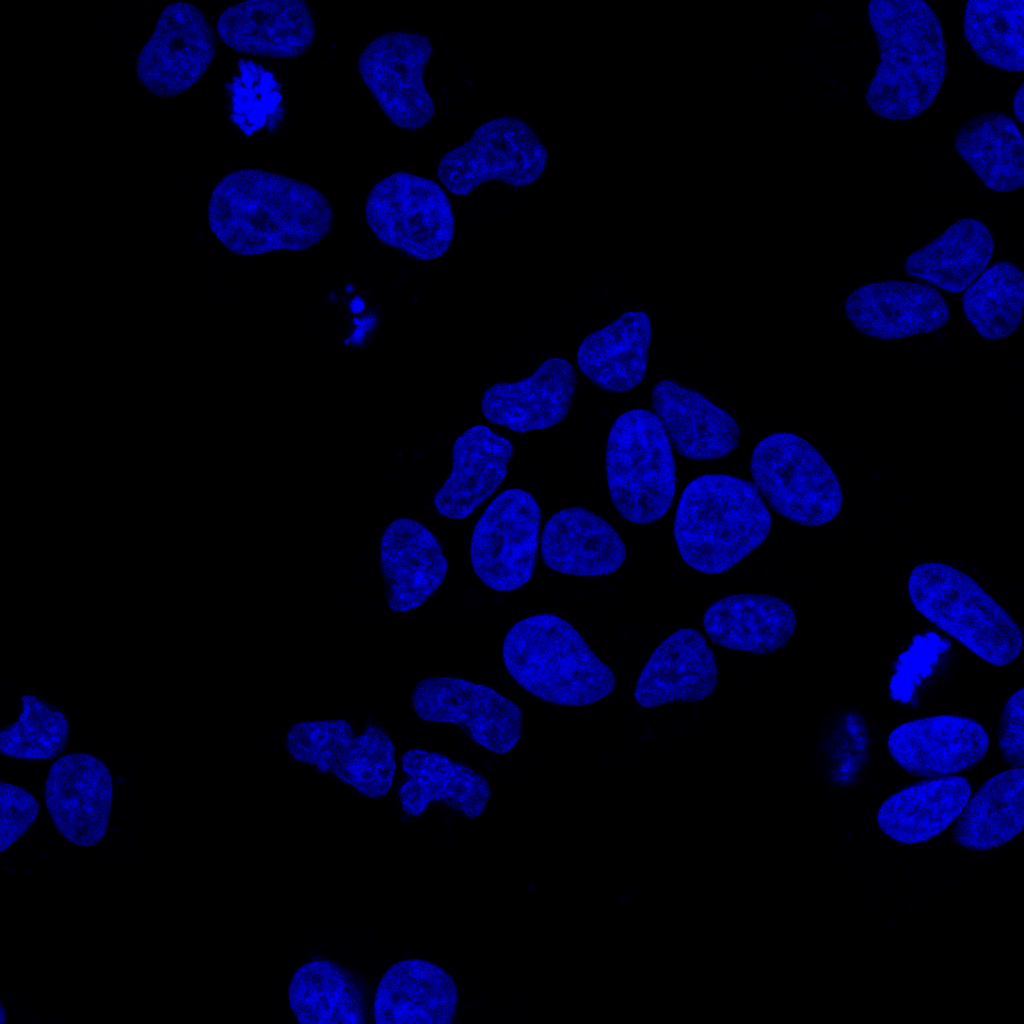

Supplement: Supplementary file 6 — Source data Fig. 4 [file 44318_2024_233_MOESM6_ESM.zip › 4G/Image/DLK1 CCD RFP ATG16L1 TGOLN2 GFP_Series010_ch00_SV.tif]

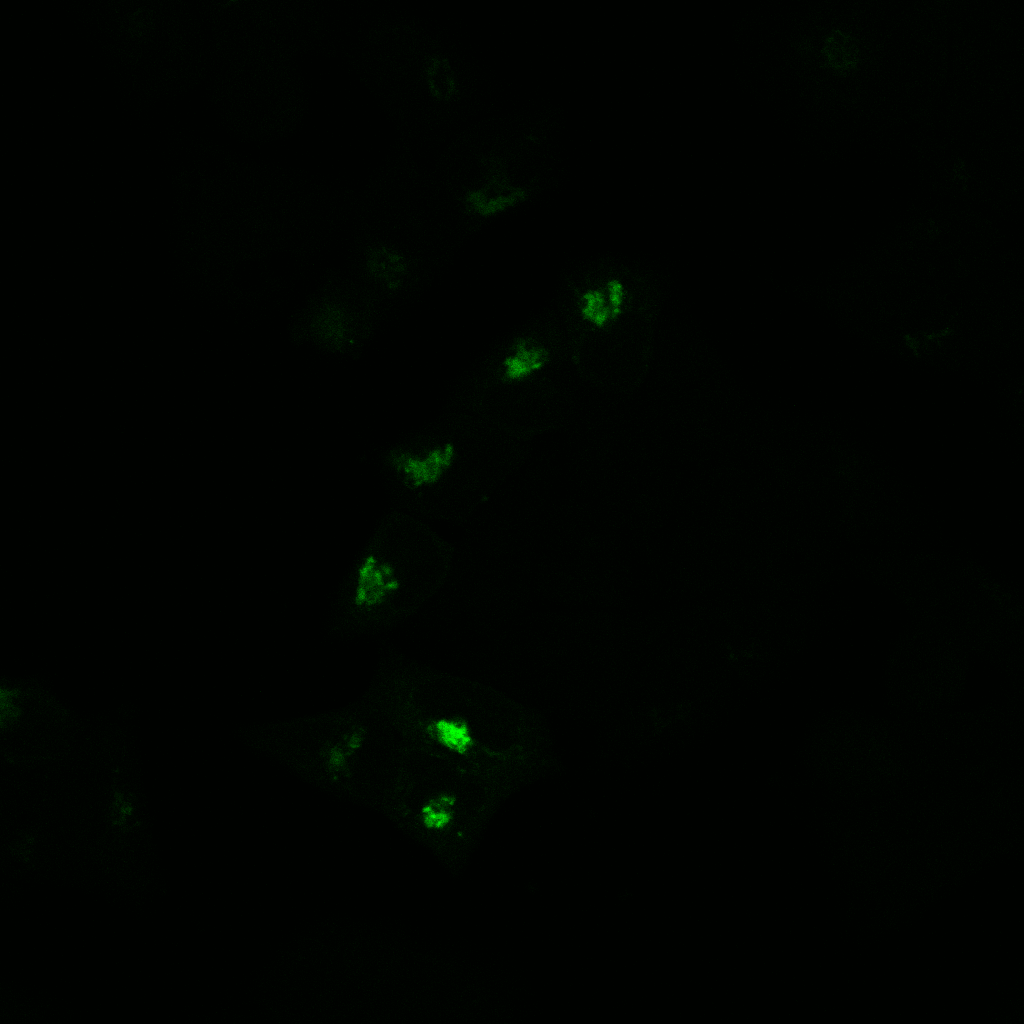

Supplement: Supplementary file 6 — Source data Fig. 4 [file 44318_2024_233_MOESM6_ESM.zip › 4G/Image/DLK1 CCD RFP ATG16L1 TGOLN2 GFP_Series010_ch01_SV.tif]

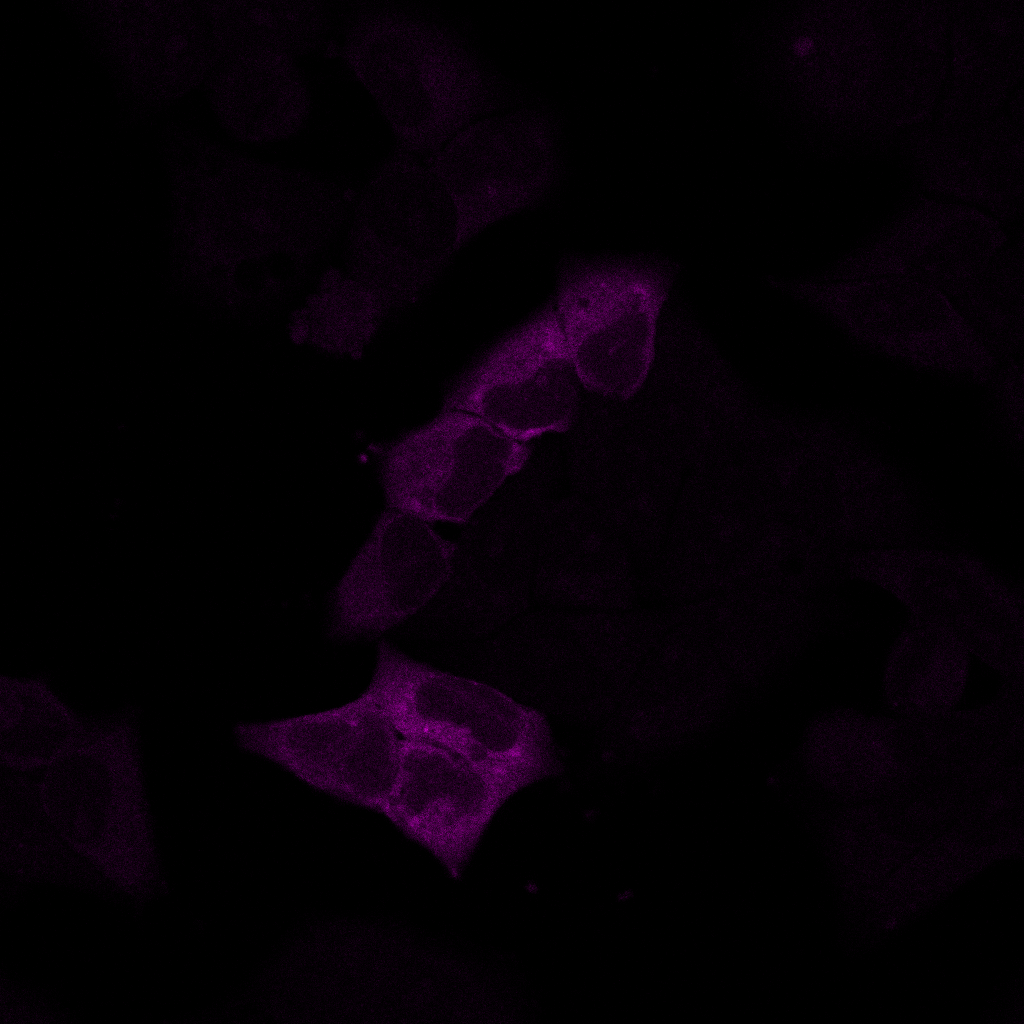

Supplement: Supplementary file 6 — Source data Fig. 4 [file 44318_2024_233_MOESM6_ESM.zip › 4G/Image/DLK1 CCD RFP ATG16L1 TGOLN2 GFP_Series010_ch02_SV.tif]

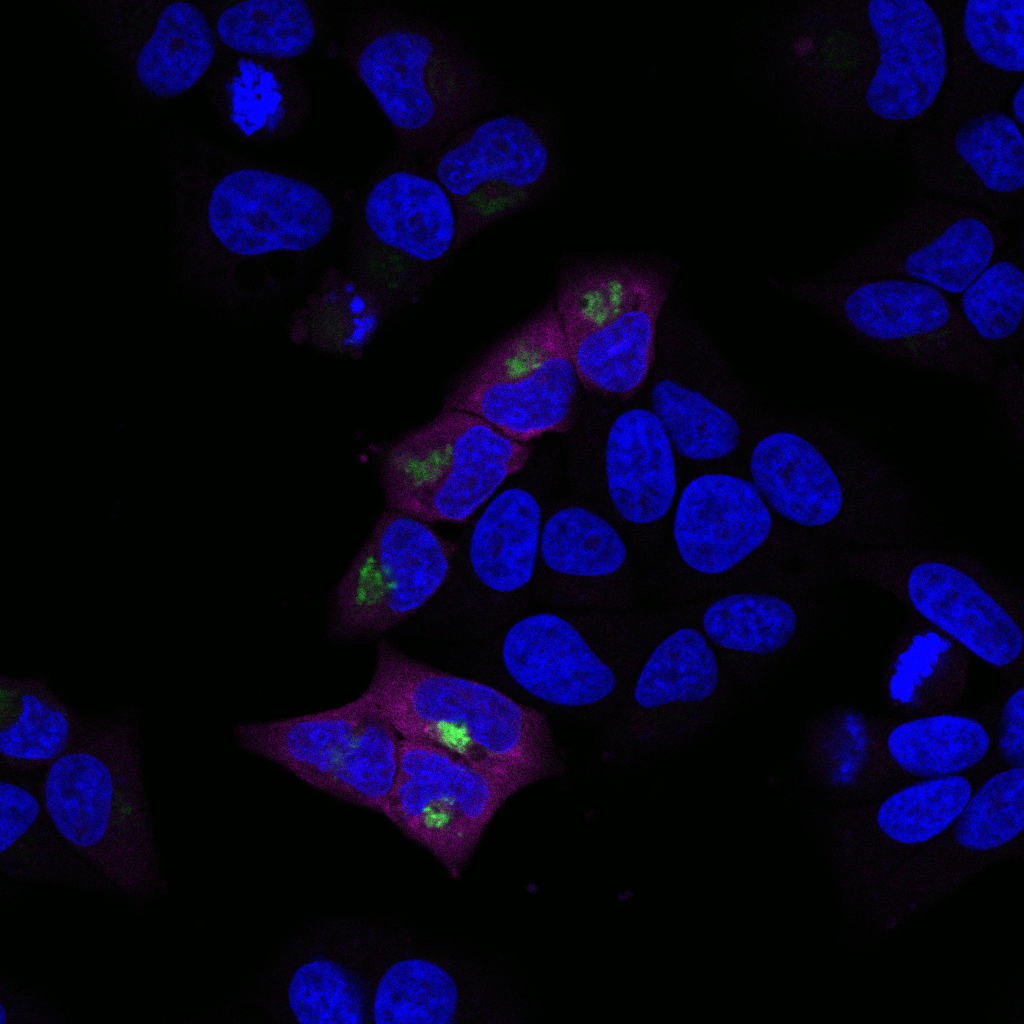

Supplement: Supplementary file 6 — Source data Fig. 4 [file 44318_2024_233_MOESM6_ESM.zip › 4G/Image/DLK1 CCD RFP ATG16L1 TGOLN2 GFP_Series010_overlay.tif]

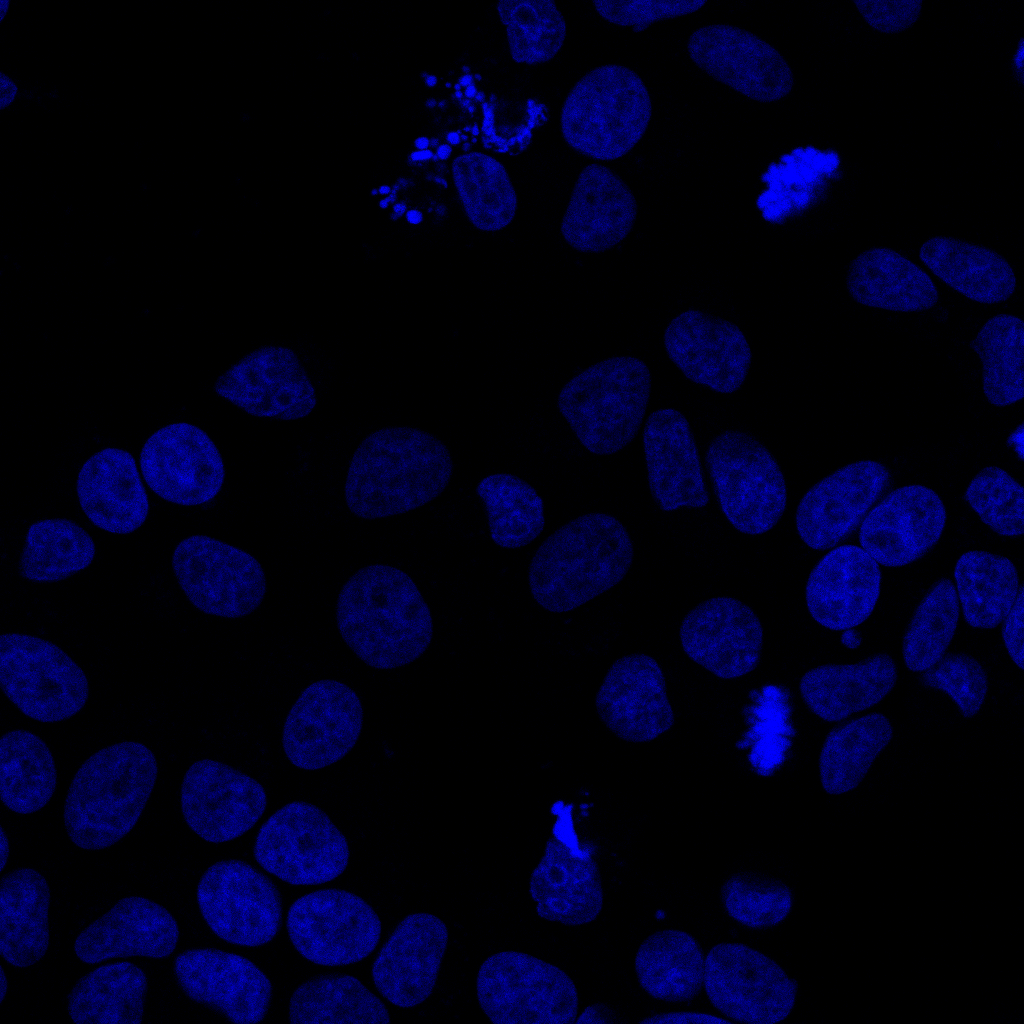

Supplement: Supplementary file 6 — Source data Fig. 4 [file 44318_2024_233_MOESM6_ESM.zip › 4G/Image/DLK1 FBD RFP ATG16L1 TGOLN2 GFP_Series011_ch00_SV.tif]

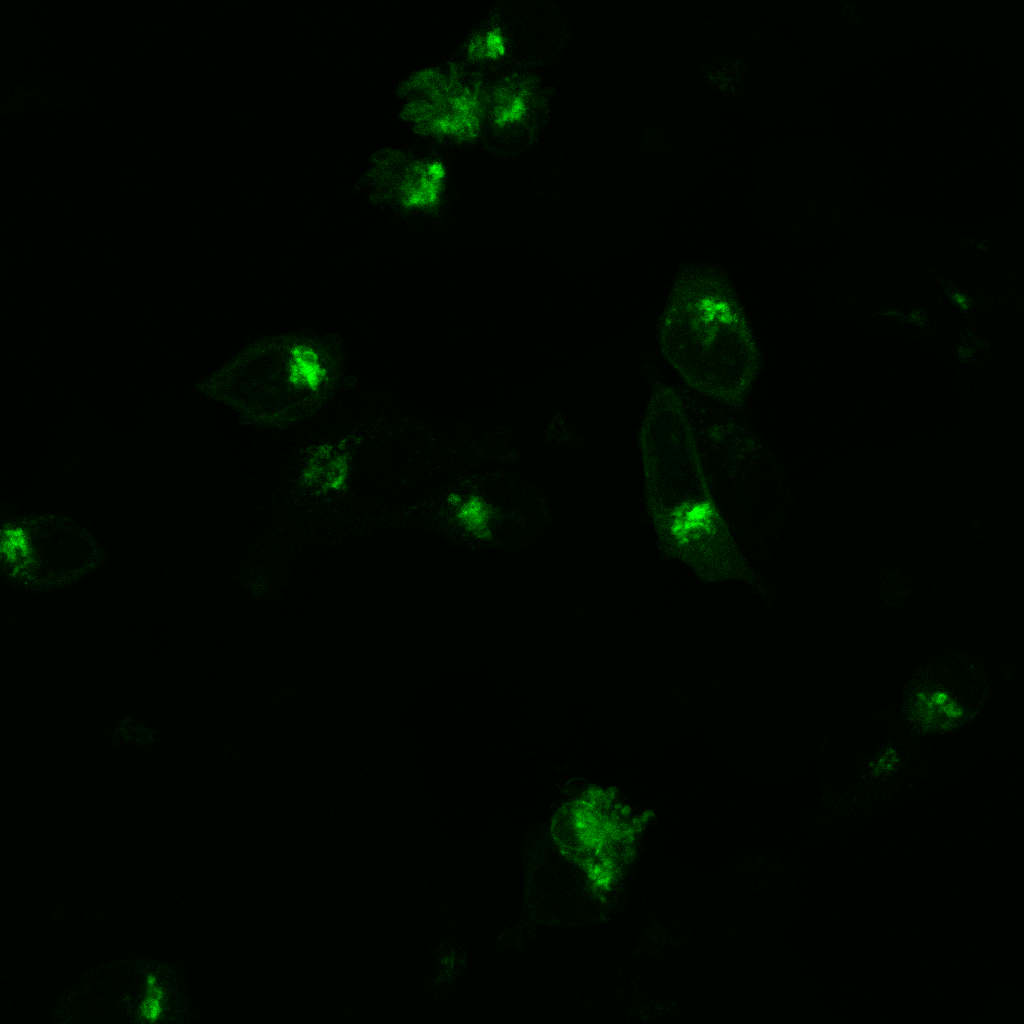

Supplement: Supplementary file 6 — Source data Fig. 4 [file 44318_2024_233_MOESM6_ESM.zip › 4G/Image/DLK1 FBD RFP ATG16L1 TGOLN2 GFP_Series011_ch01_SV.tif]

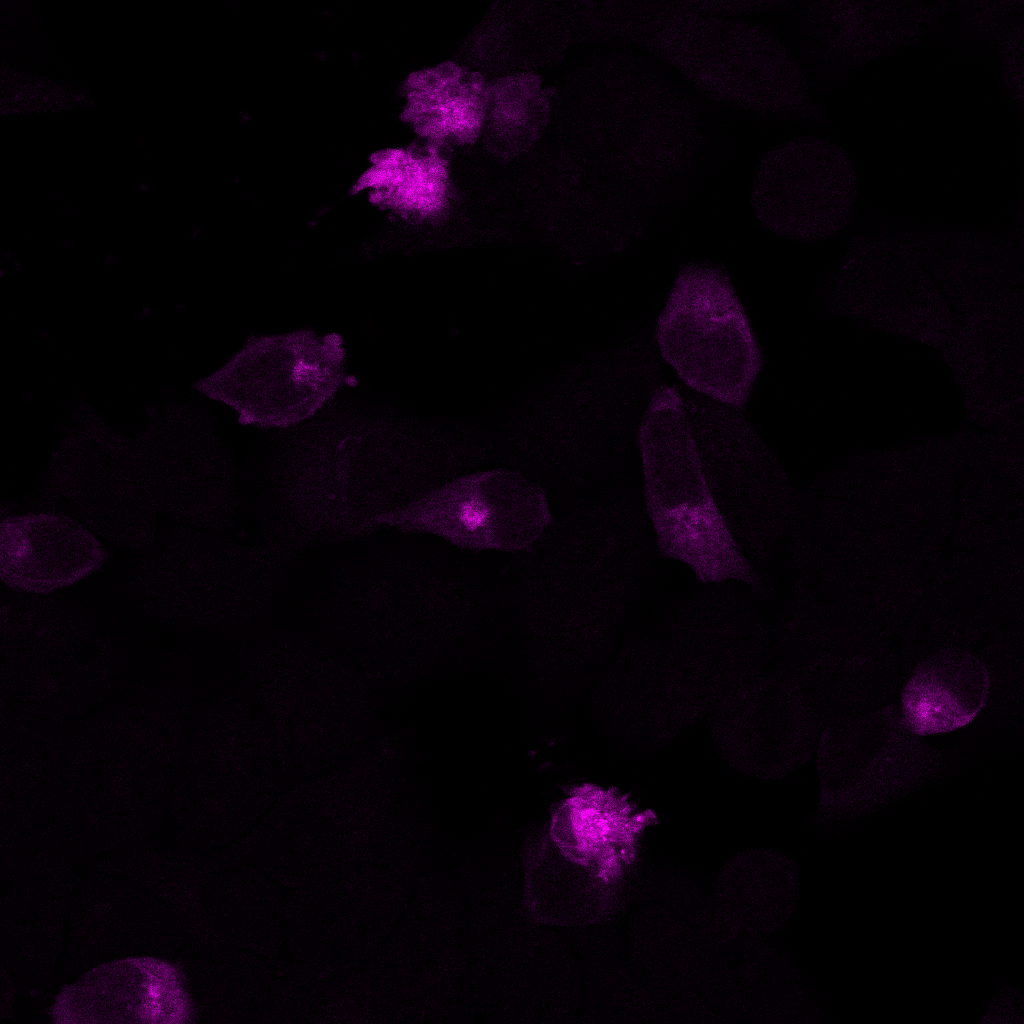

Supplement: Supplementary file 6 — Source data Fig. 4 [file 44318_2024_233_MOESM6_ESM.zip › 4G/Image/DLK1 FBD RFP ATG16L1 TGOLN2 GFP_Series011_ch02_SV.tif]

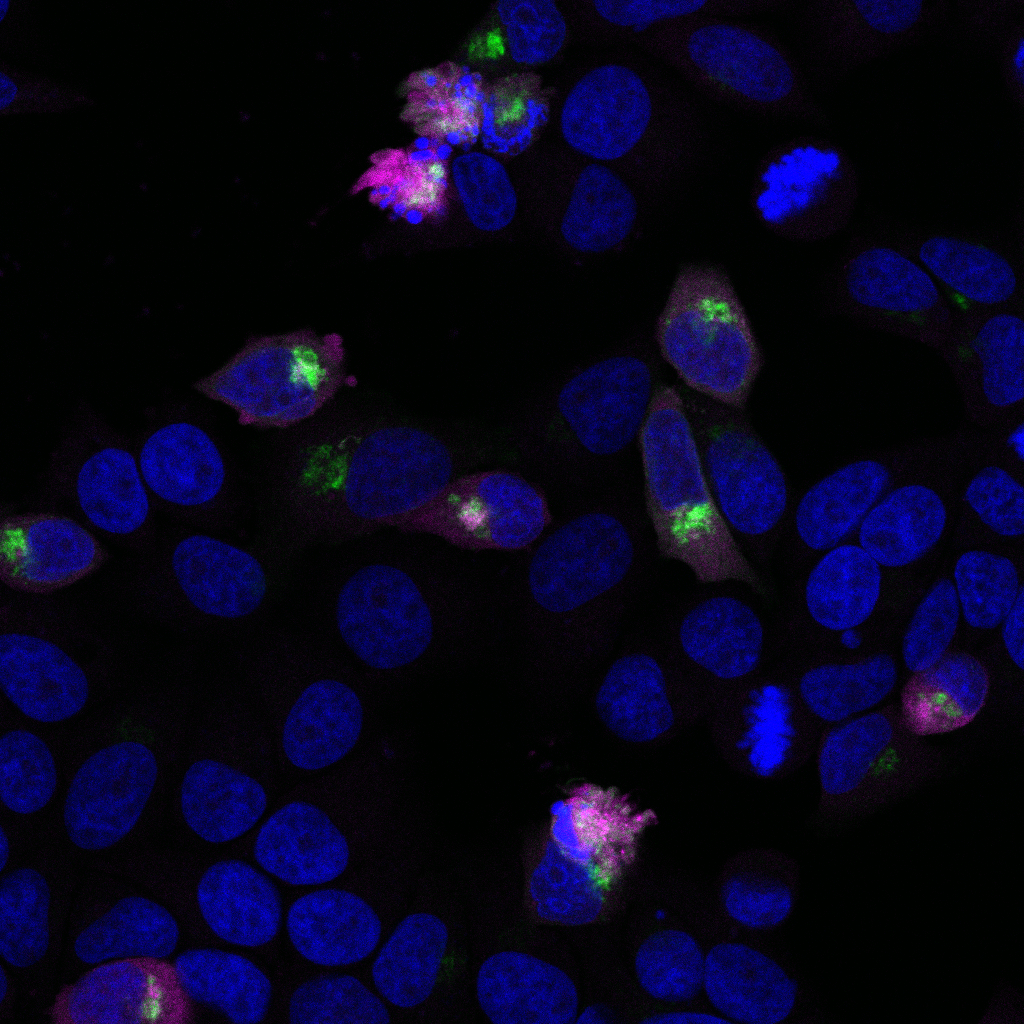

Supplement: Supplementary file 6 — Source data Fig. 4 [file 44318_2024_233_MOESM6_ESM.zip › 4G/Image/DLK1 FBD RFP ATG16L1 TGOLN2 GFP_Series011_overlay.tif]

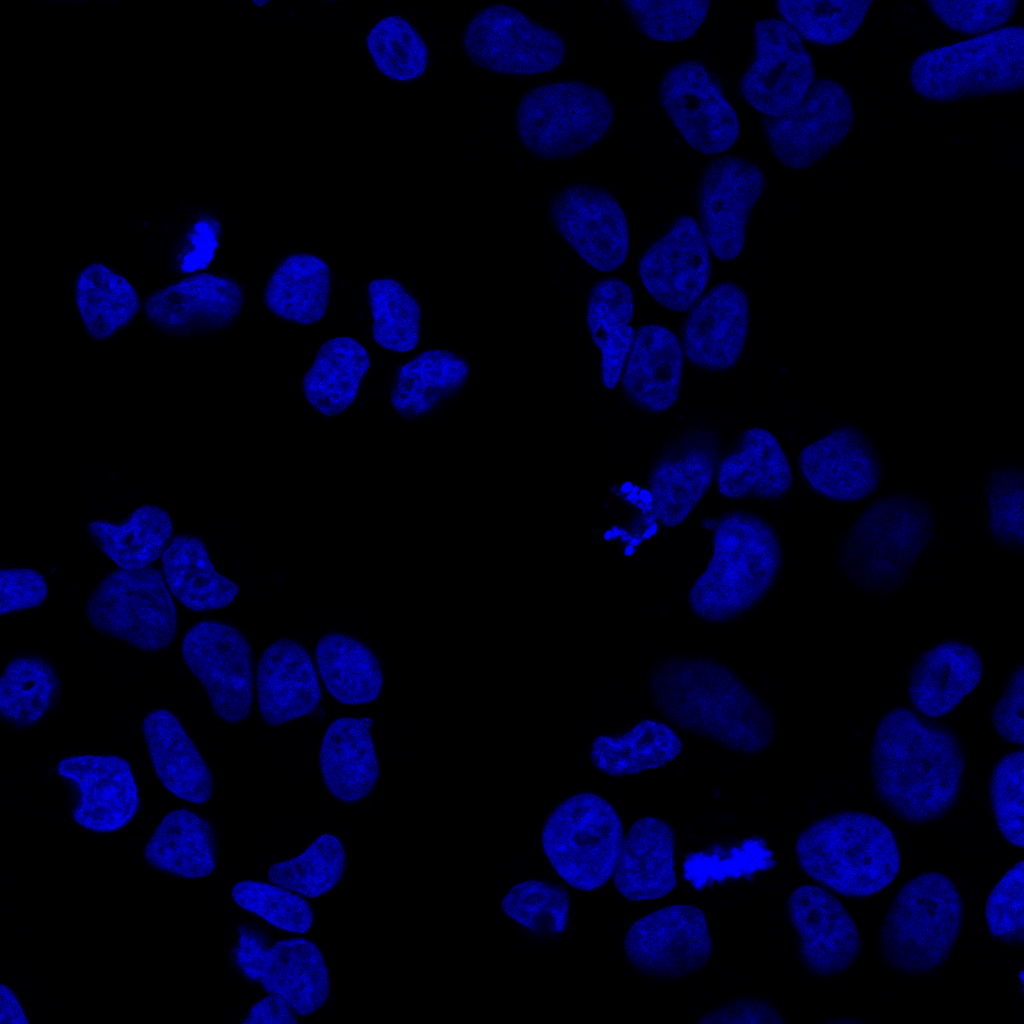

Supplement: Supplementary file 6 — Source data Fig. 4 [file 44318_2024_233_MOESM6_ESM.zip › 4G/Image/DLK1 WD40 RFP ATG16L1 TGOLN2 GFP_Series008_ch00_SV.tif]

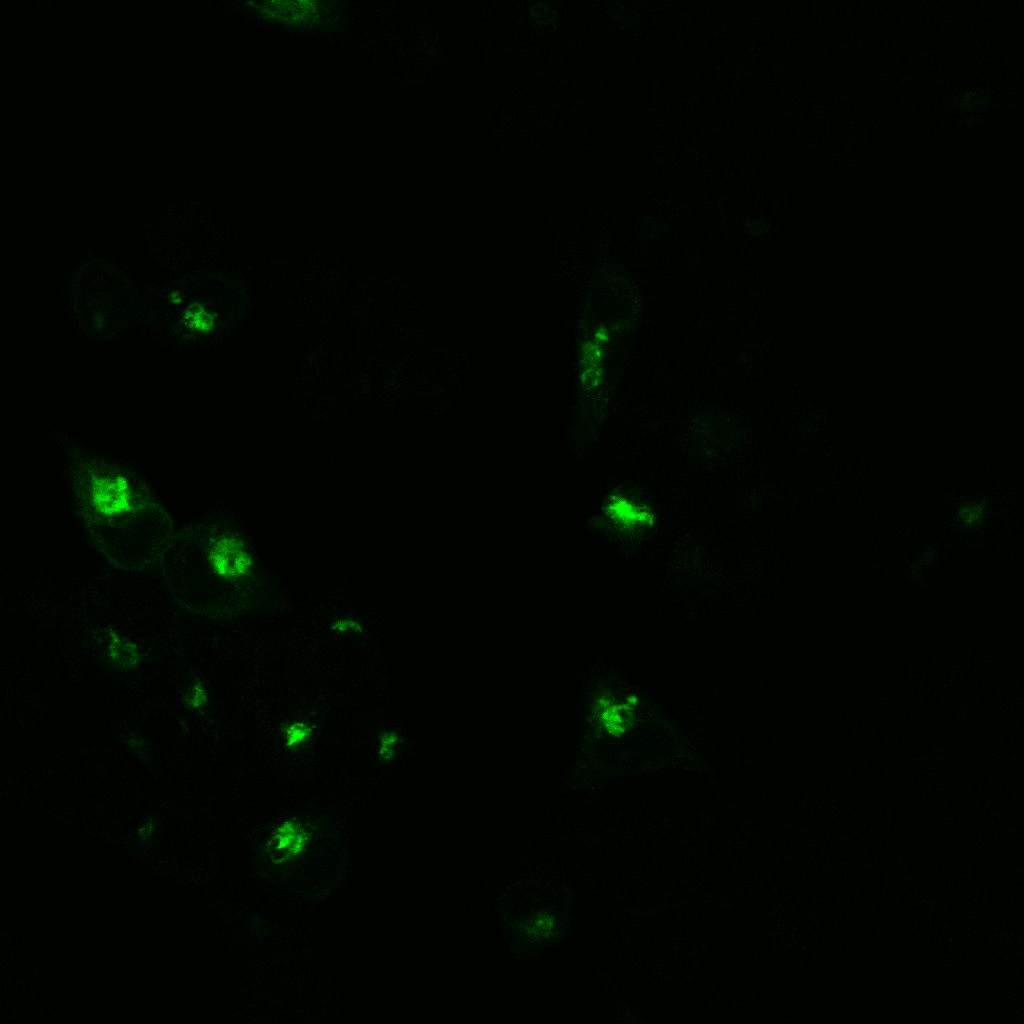

Supplement: Supplementary file 6 — Source data Fig. 4 [file 44318_2024_233_MOESM6_ESM.zip › 4G/Image/DLK1 WD40 RFP ATG16L1 TGOLN2 GFP_Series008_ch01_SV.tif]

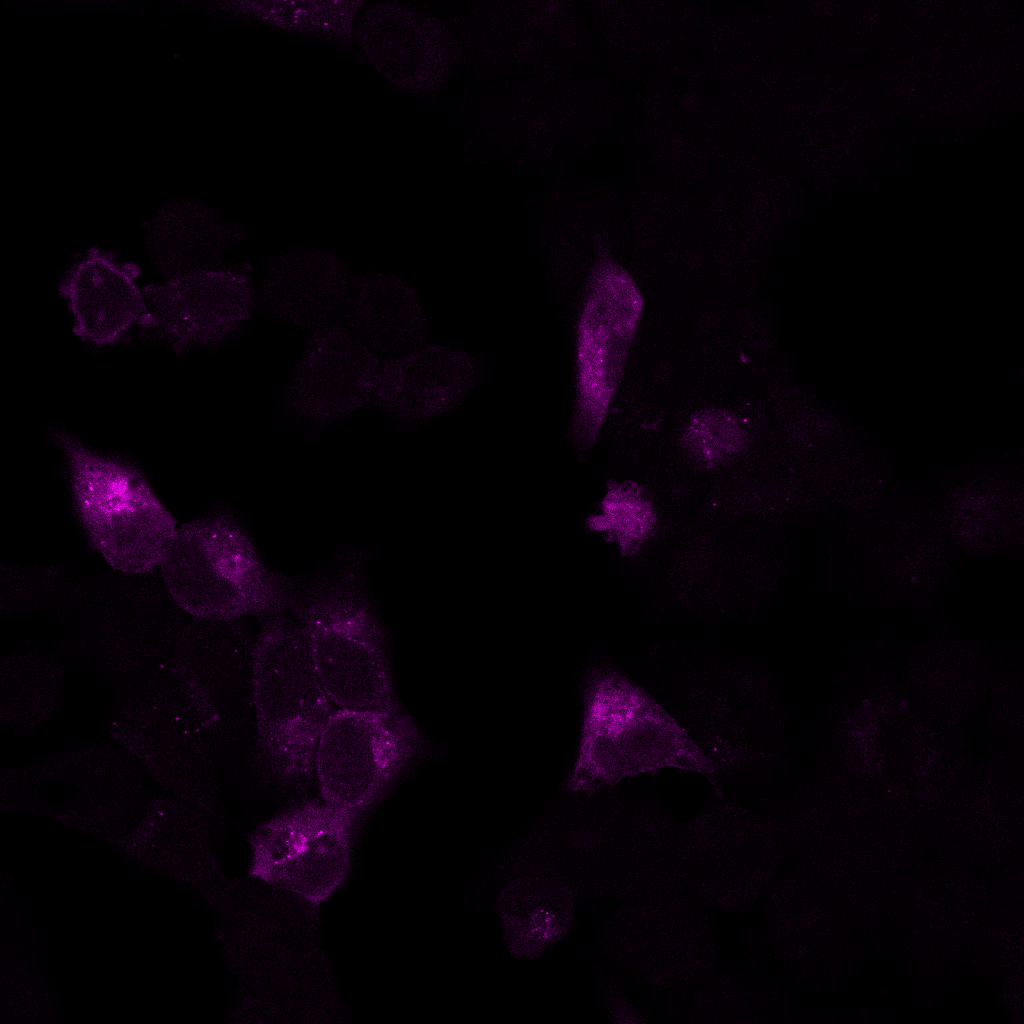

Supplement: Supplementary file 6 — Source data Fig. 4 [file 44318_2024_233_MOESM6_ESM.zip › 4G/Image/DLK1 WD40 RFP ATG16L1 TGOLN2 GFP_Series008_ch02_SV.tif]

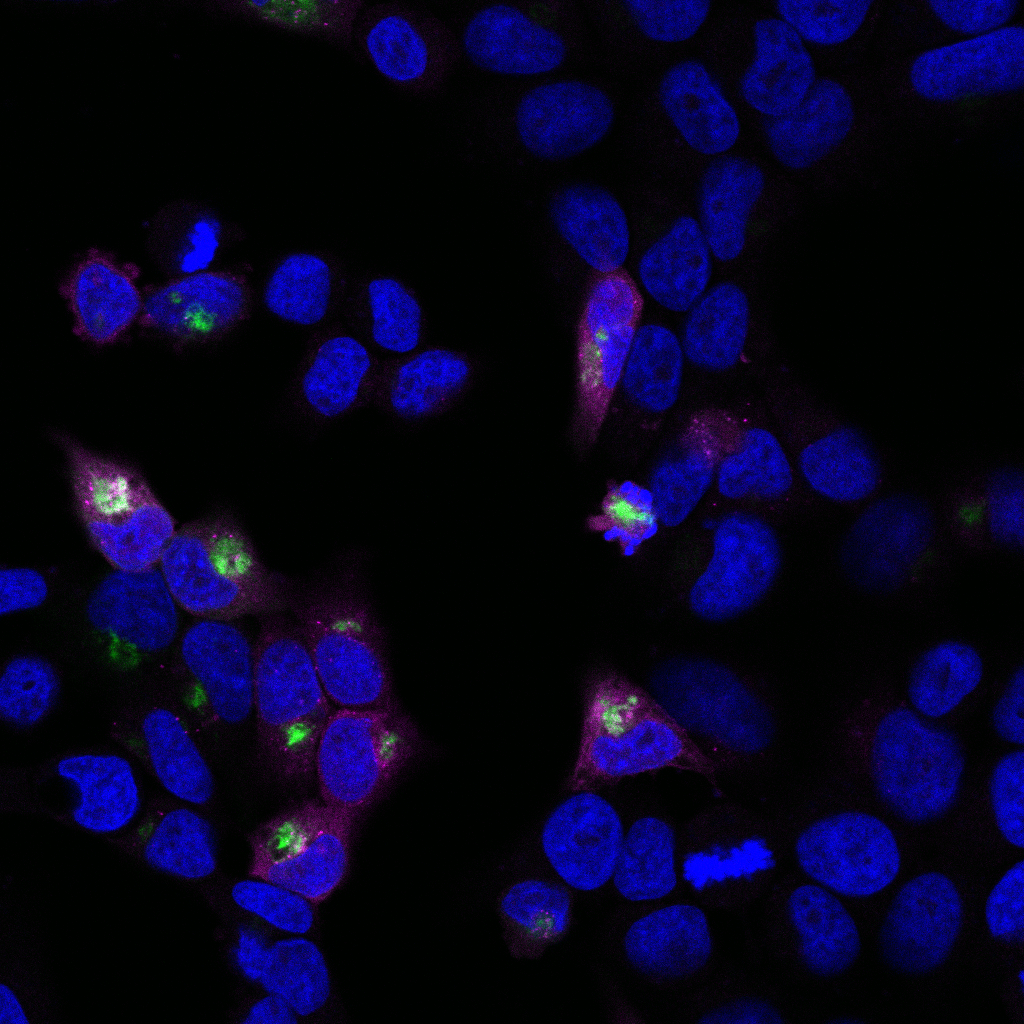

Supplement: Supplementary file 6 — Source data Fig. 4 [file 44318_2024_233_MOESM6_ESM.zip › 4G/Image/DLK1 WD40 RFP ATG16L1 TGOLN2 GFP_Series008_overlay.tif]

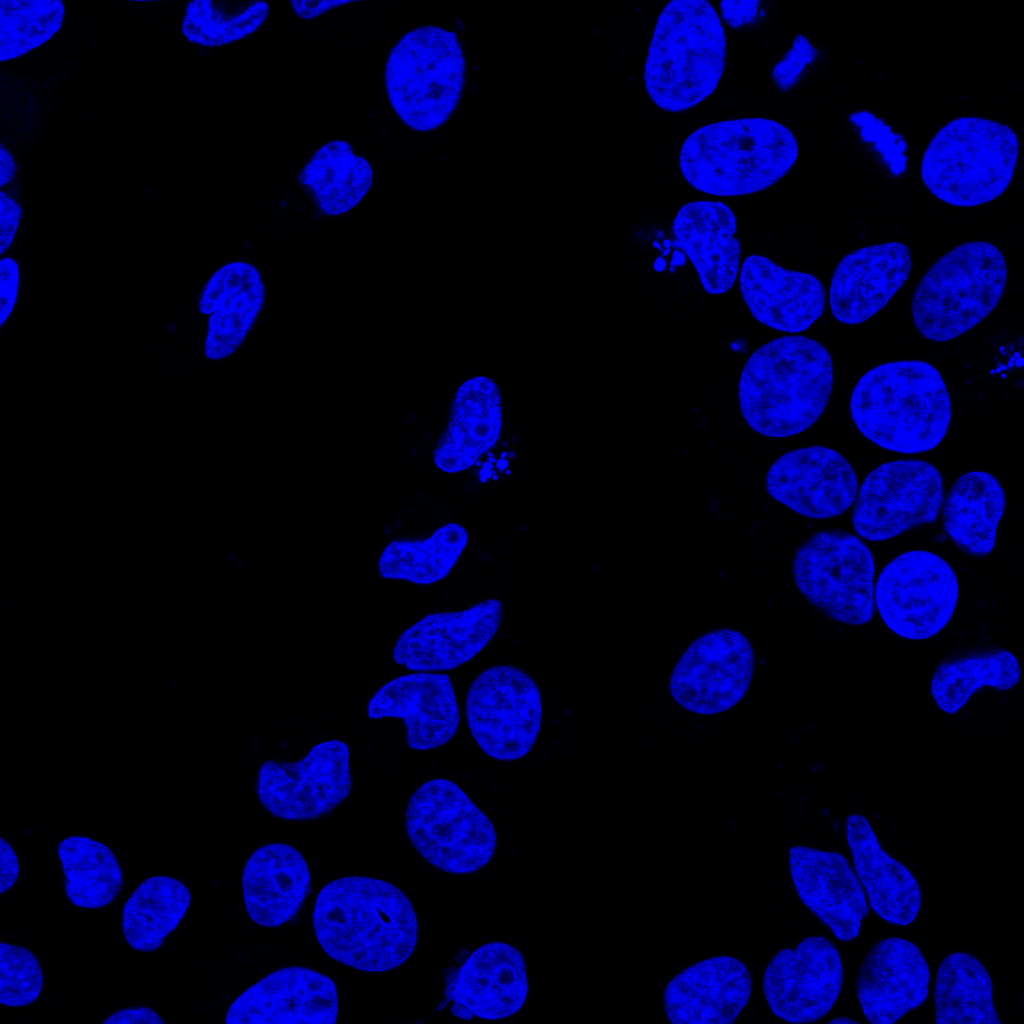

Supplement: Supplementary file 6 — Source data Fig. 4 [file 44318_2024_233_MOESM6_ESM.zip › 4G/Image/DLK1 WT RFP ATG16L1 TGOLN2 GFP_Series006_ch00_SV.tif]

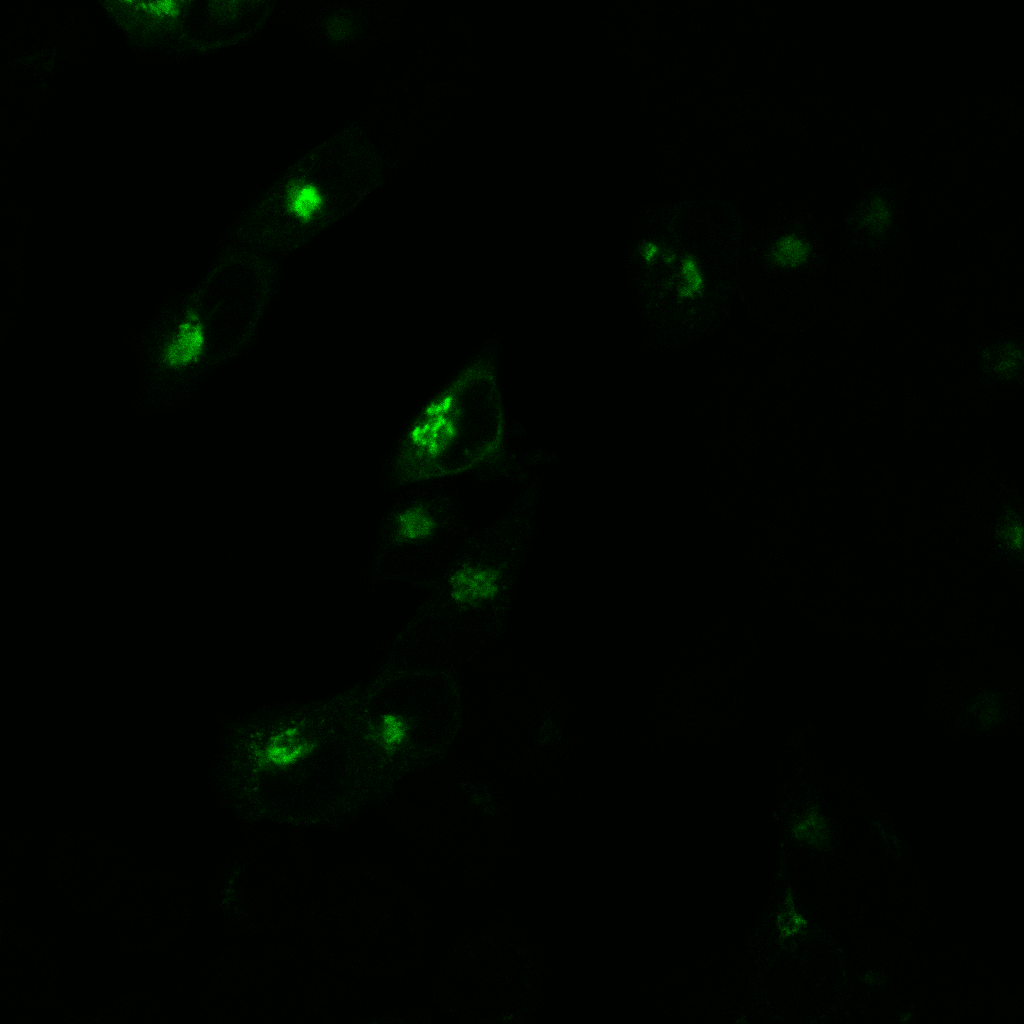

Supplement: Supplementary file 6 — Source data Fig. 4 [file 44318_2024_233_MOESM6_ESM.zip › 4G/Image/DLK1 WT RFP ATG16L1 TGOLN2 GFP_Series006_ch01_SV.tif]

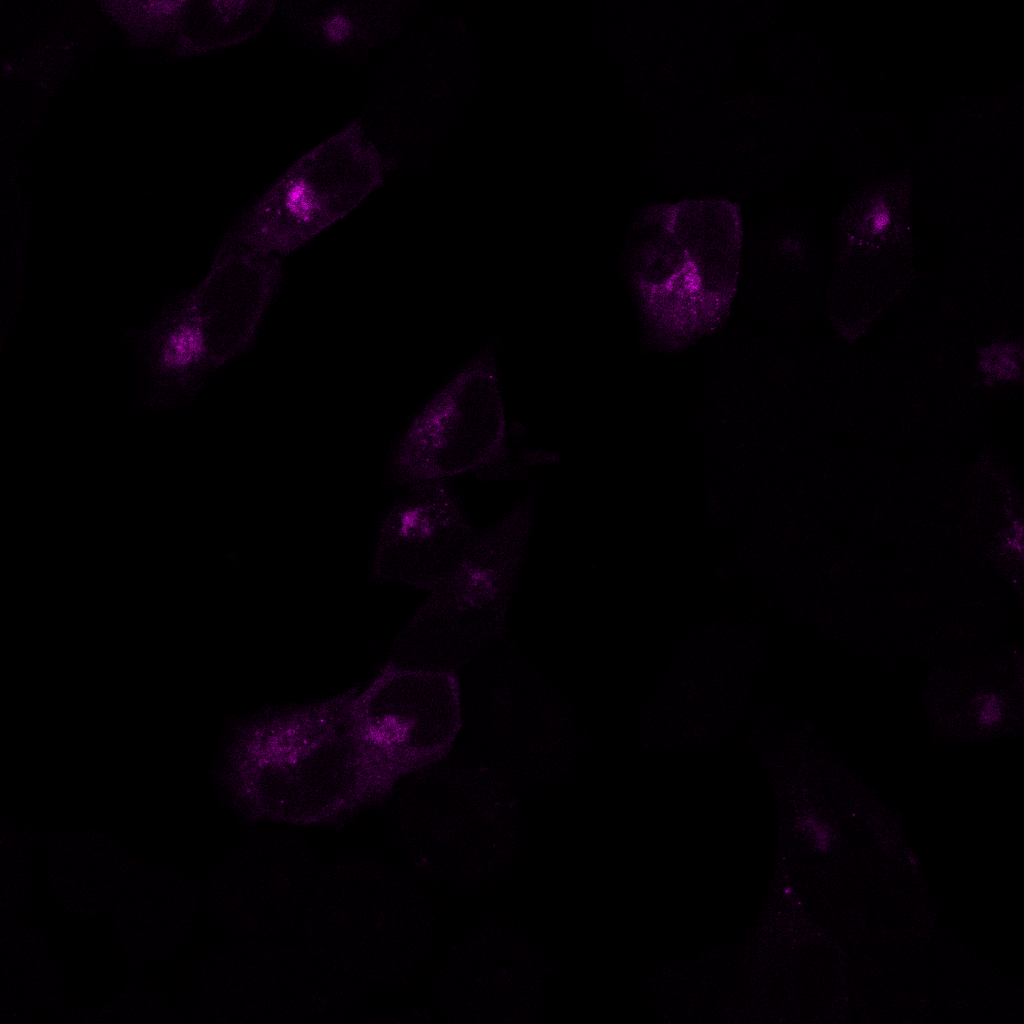

Supplement: Supplementary file 6 — Source data Fig. 4 [file 44318_2024_233_MOESM6_ESM.zip › 4G/Image/DLK1 WT RFP ATG16L1 TGOLN2 GFP_Series006_ch02_SV.tif]

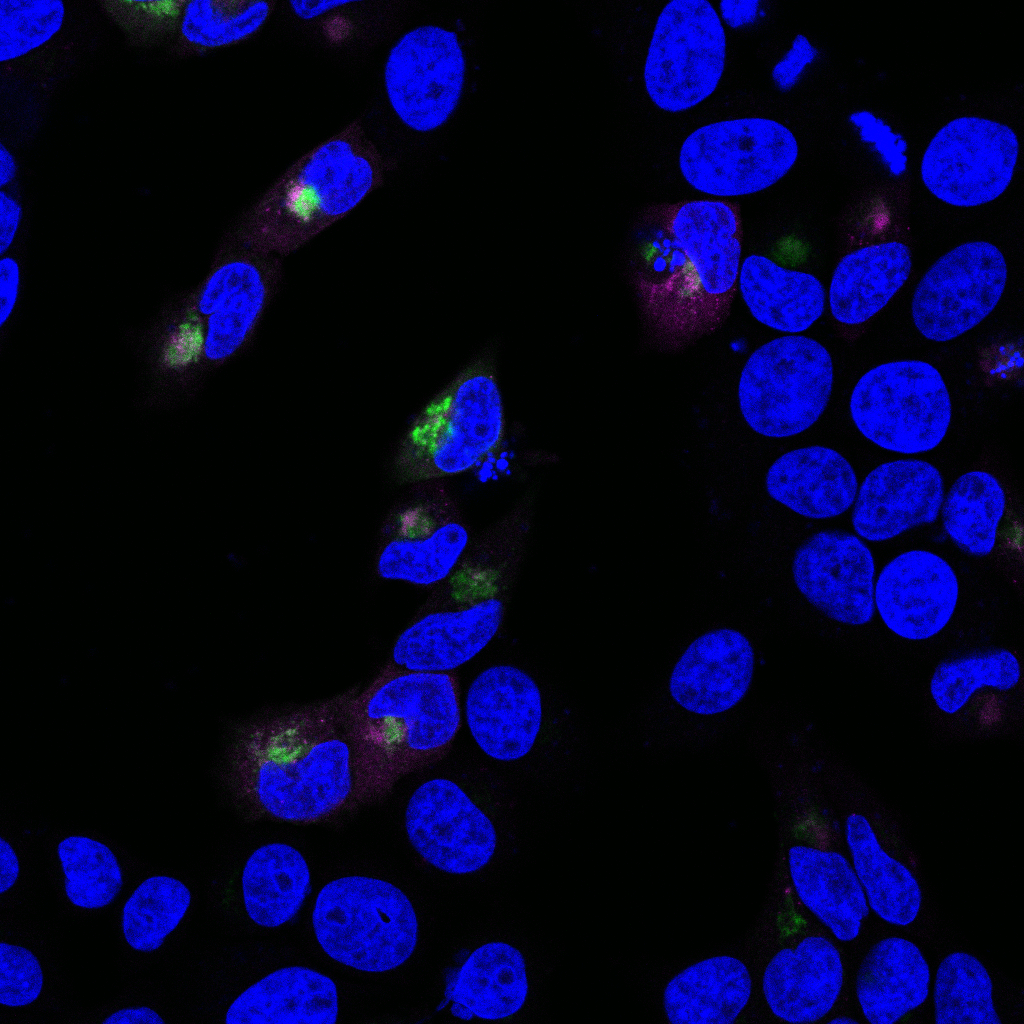

Supplement: Supplementary file 6 — Source data Fig. 4 [file 44318_2024_233_MOESM6_ESM.zip › 4G/Image/DLK1 WT RFP ATG16L1 TGOLN2 GFP_Series006_overlay.tif]

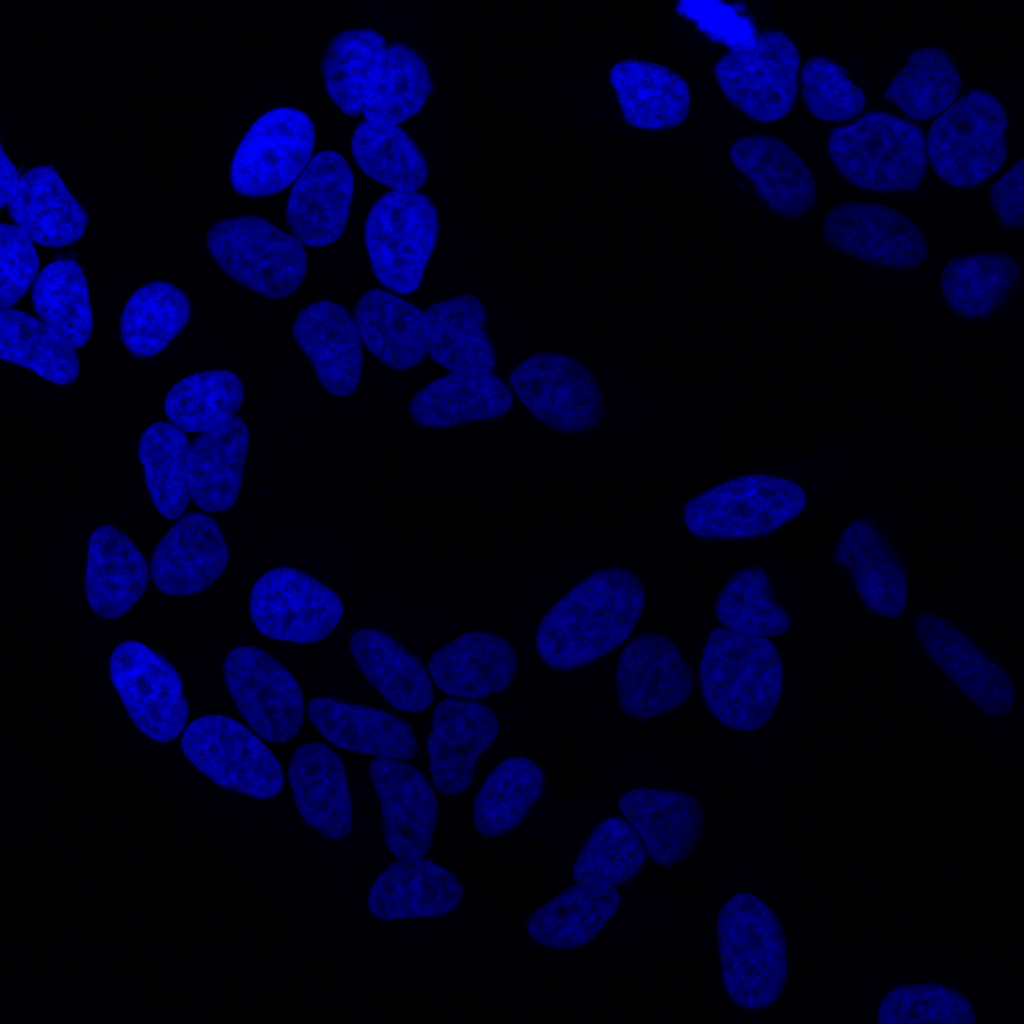

Supplement: Supplementary file 7 — Source data Fig. 5 [file 44318_2024_233_MOESM7_ESM.zip › 5A/HeLa sgATG16L1-1 AMDE-1 TFE3_Series003_ch00_SV.tif]

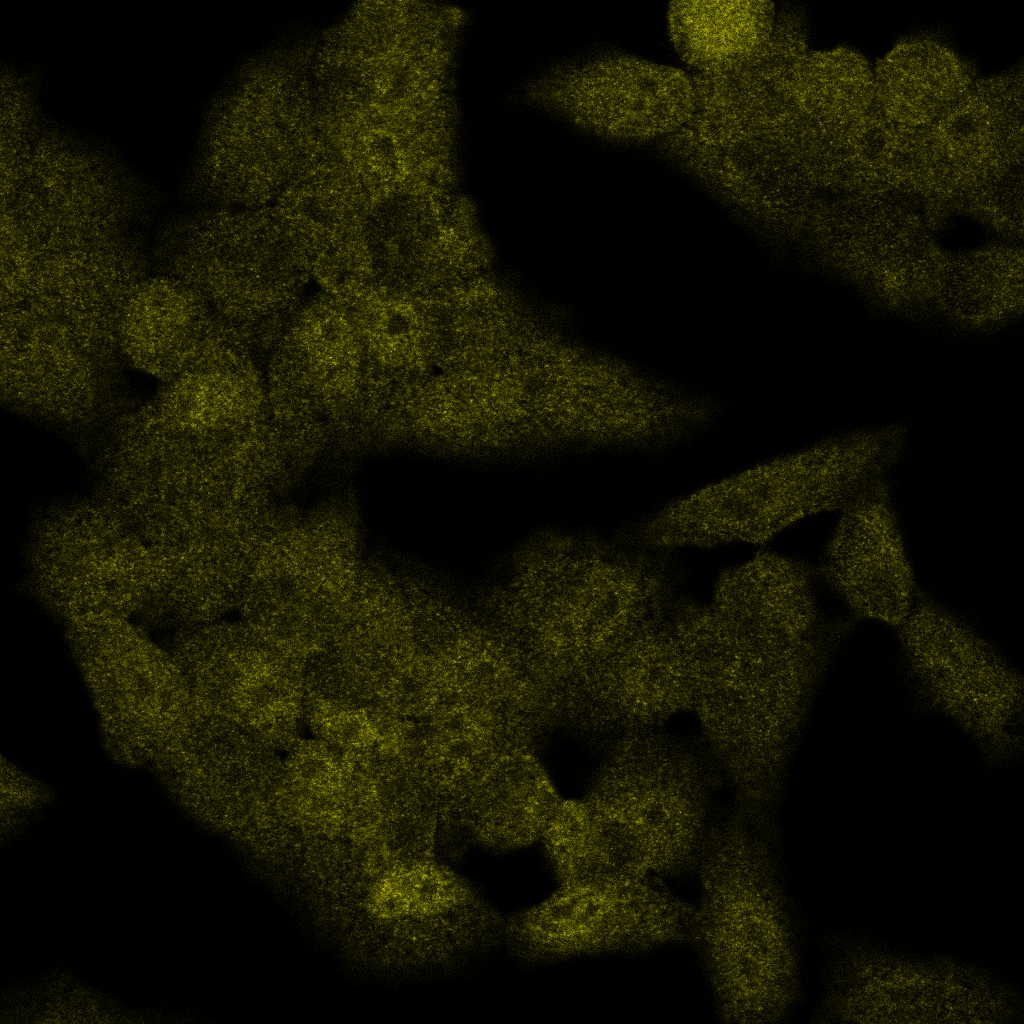

Supplement: Supplementary file 7 — Source data Fig. 5 [file 44318_2024_233_MOESM7_ESM.zip › 5A/HeLa sgATG16L1-1 AMDE-1 TFE3_Series003_ch01_SV.tif]

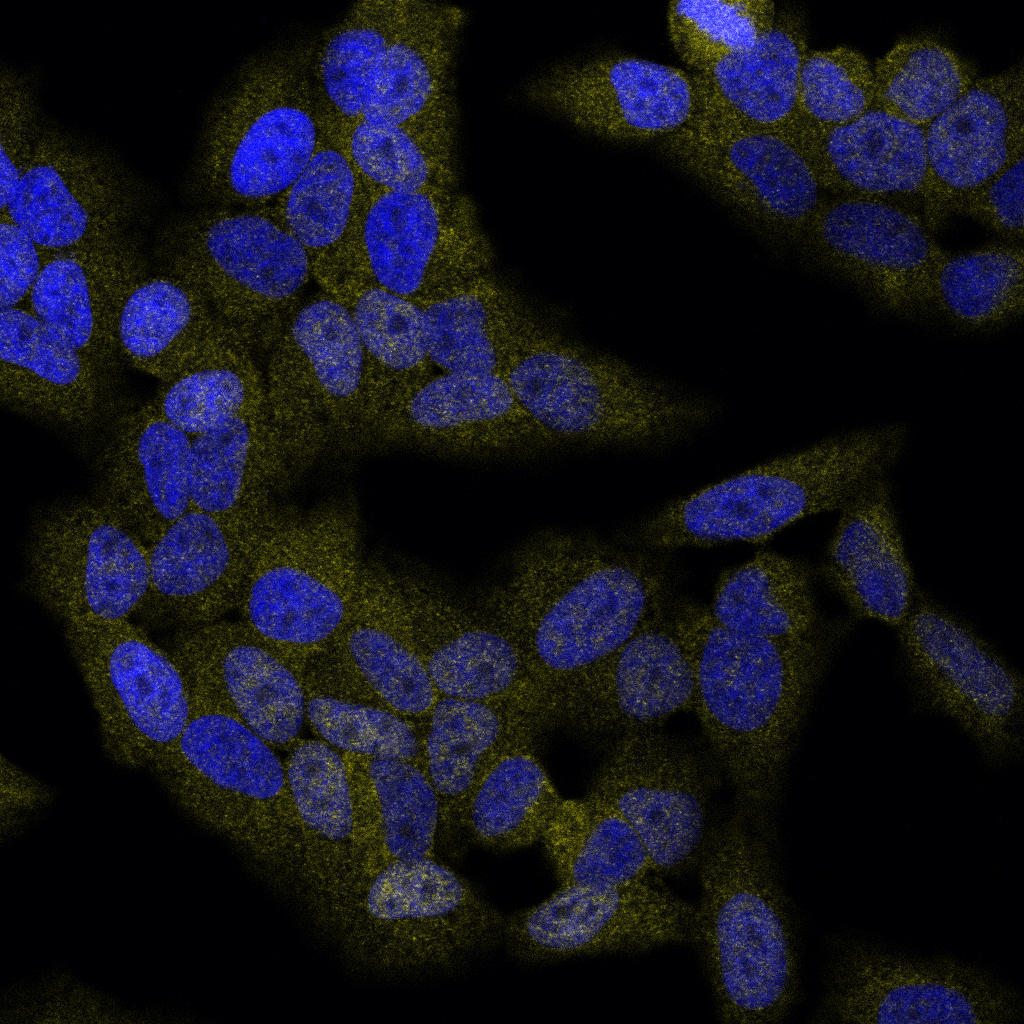

Supplement: Supplementary file 7 — Source data Fig. 5 [file 44318_2024_233_MOESM7_ESM.zip › 5A/HeLa sgATG16L1-1 AMDE-1 TFE3_Series003_overlay.tif]

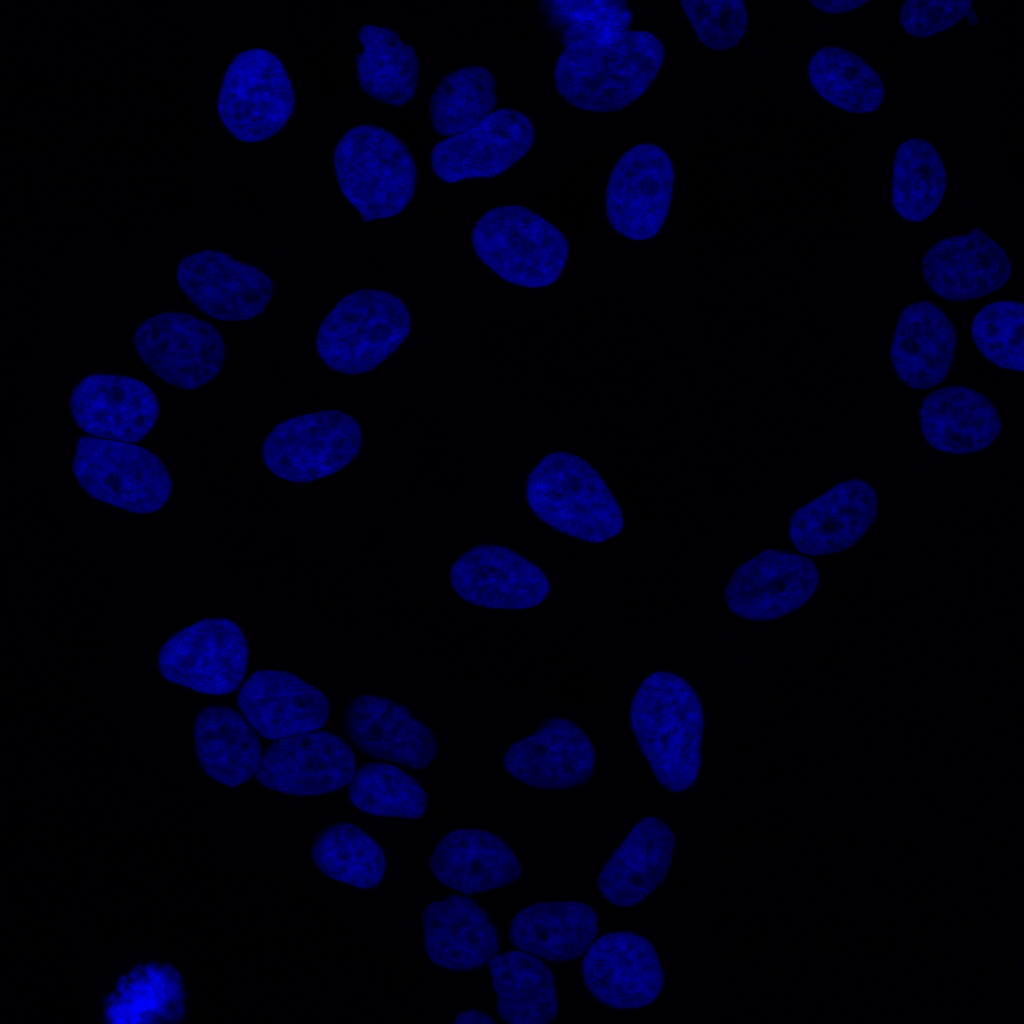

Supplement: Supplementary file 7 — Source data Fig. 5 [file 44318_2024_233_MOESM7_ESM.zip › 5A/HeLa sgATG16L1-1 Nic TFE3_Series001_ch00_SV.tif]

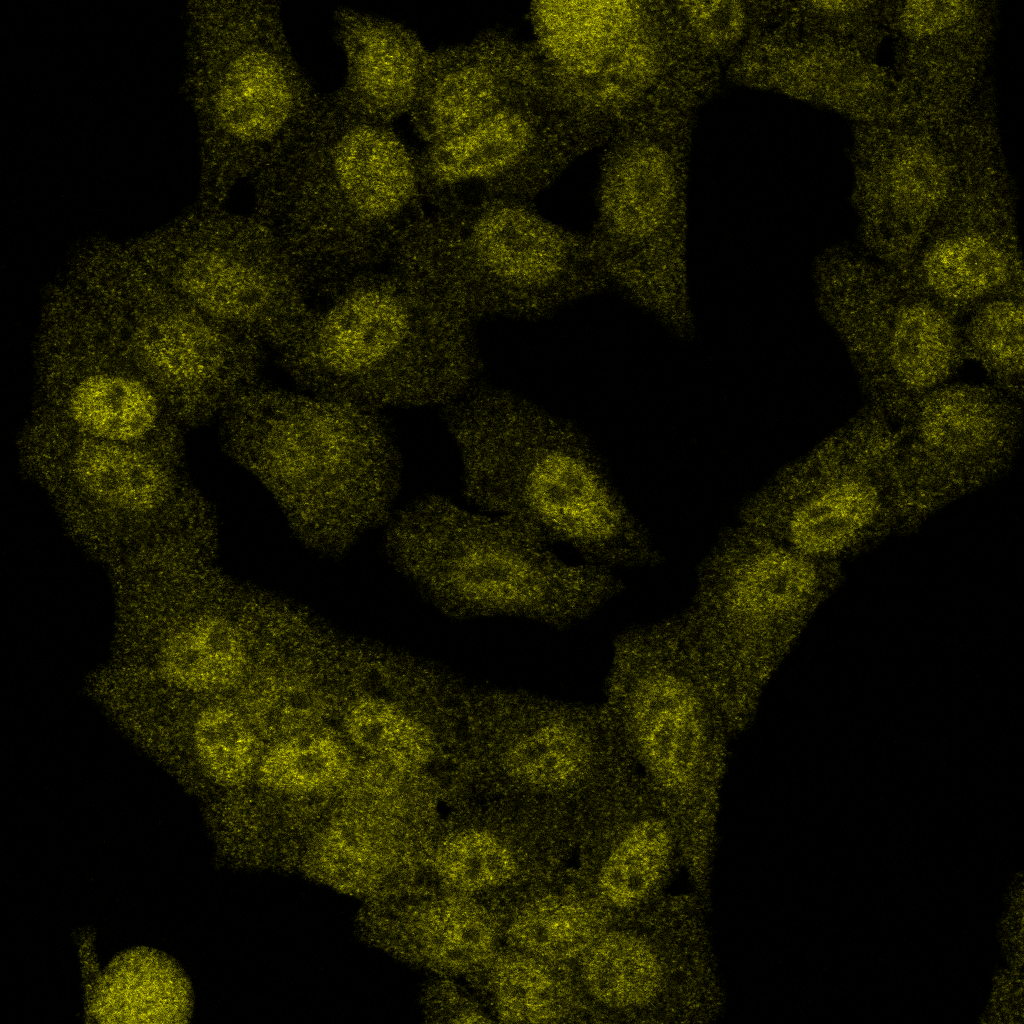

Supplement: Supplementary file 7 — Source data Fig. 5 [file 44318_2024_233_MOESM7_ESM.zip › 5A/HeLa sgATG16L1-1 Nic TFE3_Series001_ch01_SV.tif]

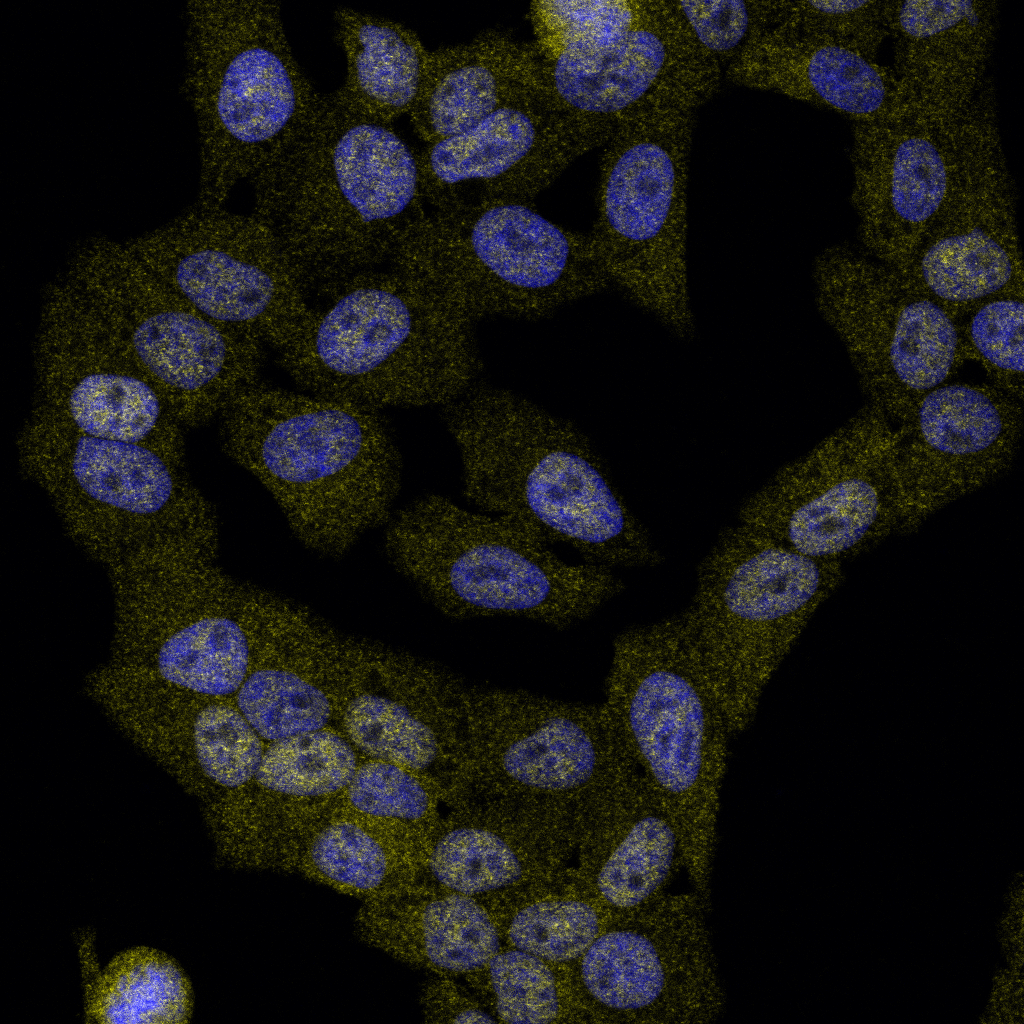

Supplement: Supplementary file 7 — Source data Fig. 5 [file 44318_2024_233_MOESM7_ESM.zip › 5A/HeLa sgATG16L1-1 Nic TFE3_Series001_overlay.tif]

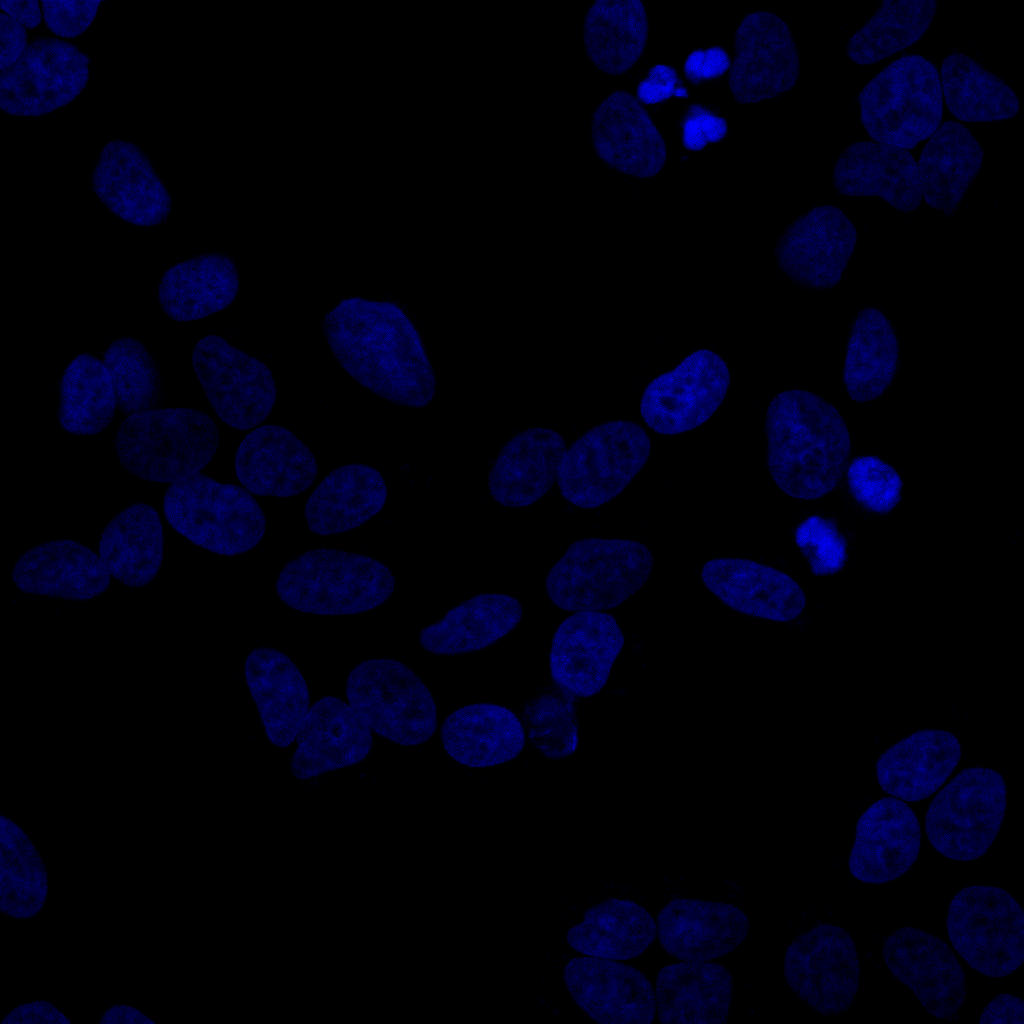

Supplement: Supplementary file 7 — Source data Fig. 5 [file 44318_2024_233_MOESM7_ESM.zip › 5A/HeLa sgATG16L1-1 Veh TFE3_Series005_ch00_SV.tif]

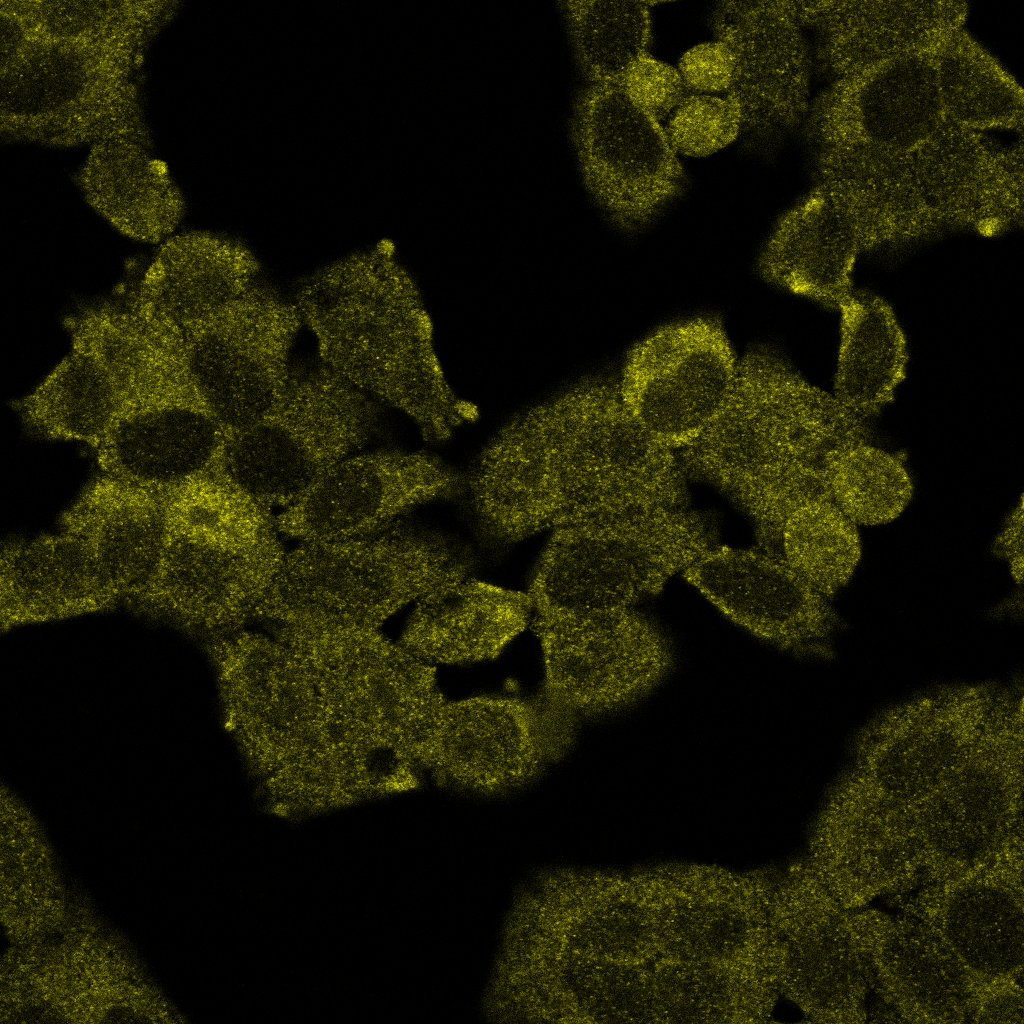

Supplement: Supplementary file 7 — Source data Fig. 5 [file 44318_2024_233_MOESM7_ESM.zip › 5A/HeLa sgATG16L1-1 Veh TFE3_Series005_ch01_SV.tif]

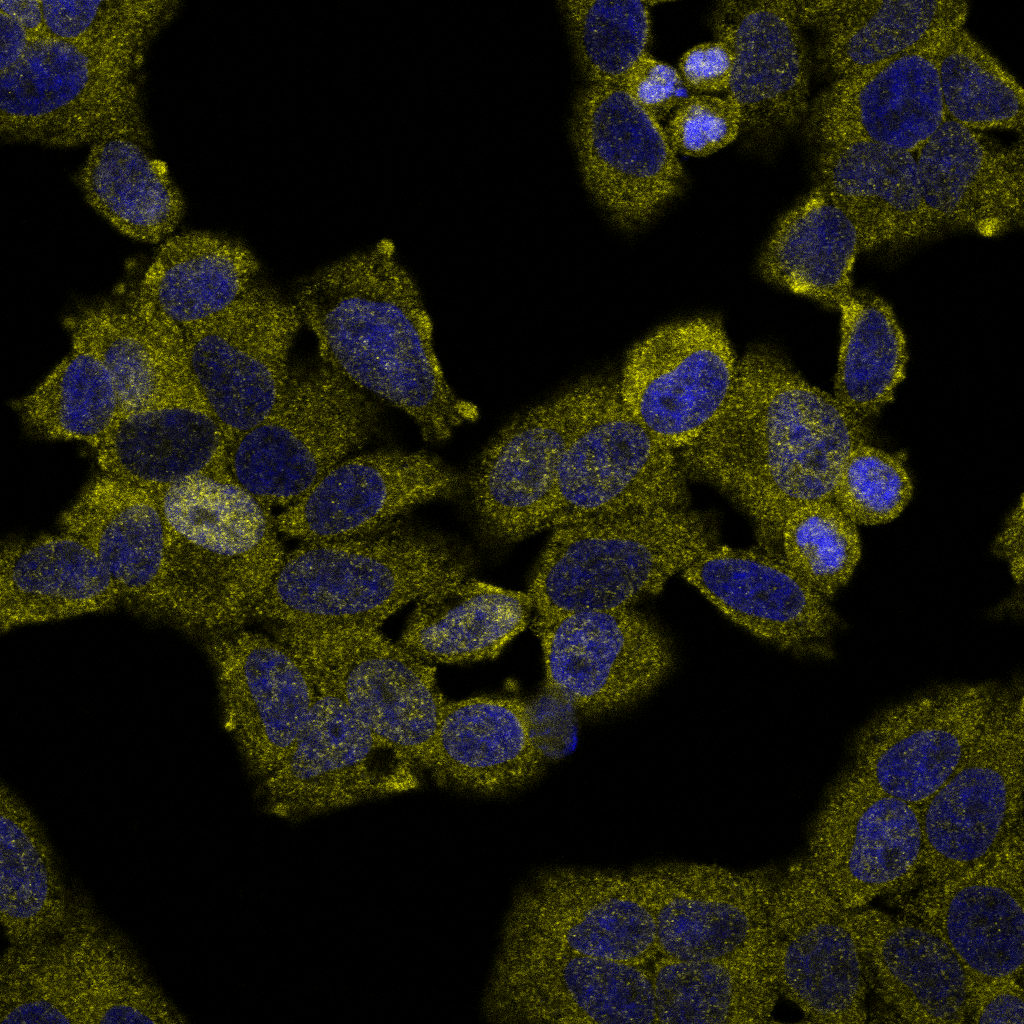

Supplement: Supplementary file 7 — Source data Fig. 5 [file 44318_2024_233_MOESM7_ESM.zip › 5A/HeLa sgATG16L1-1 Veh TFE3_Series005_overlay.tif]

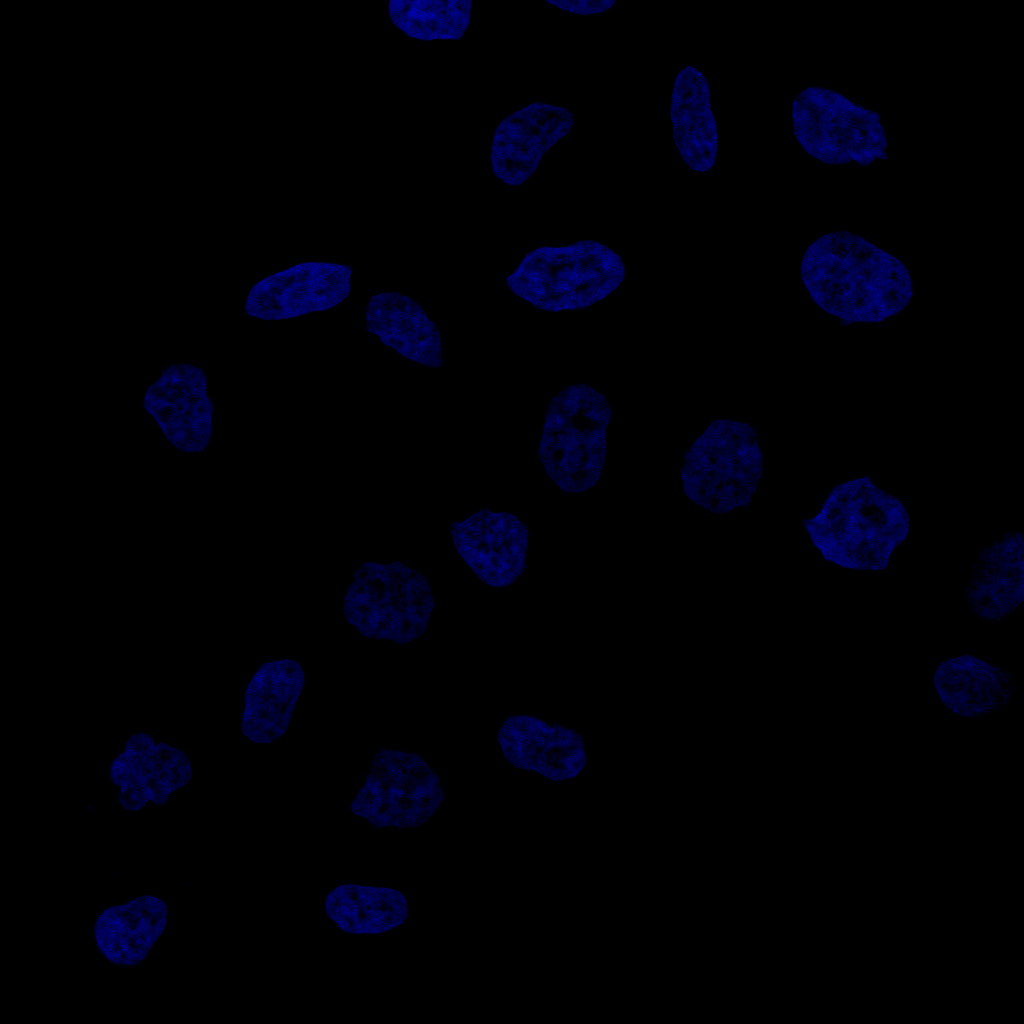

Supplement: Supplementary file 7 — Source data Fig. 5 [file 44318_2024_233_MOESM7_ESM.zip › 5A/HeLa sgATG16L1-2 AMDE TFE3_Series006_ch00_SV.tif]

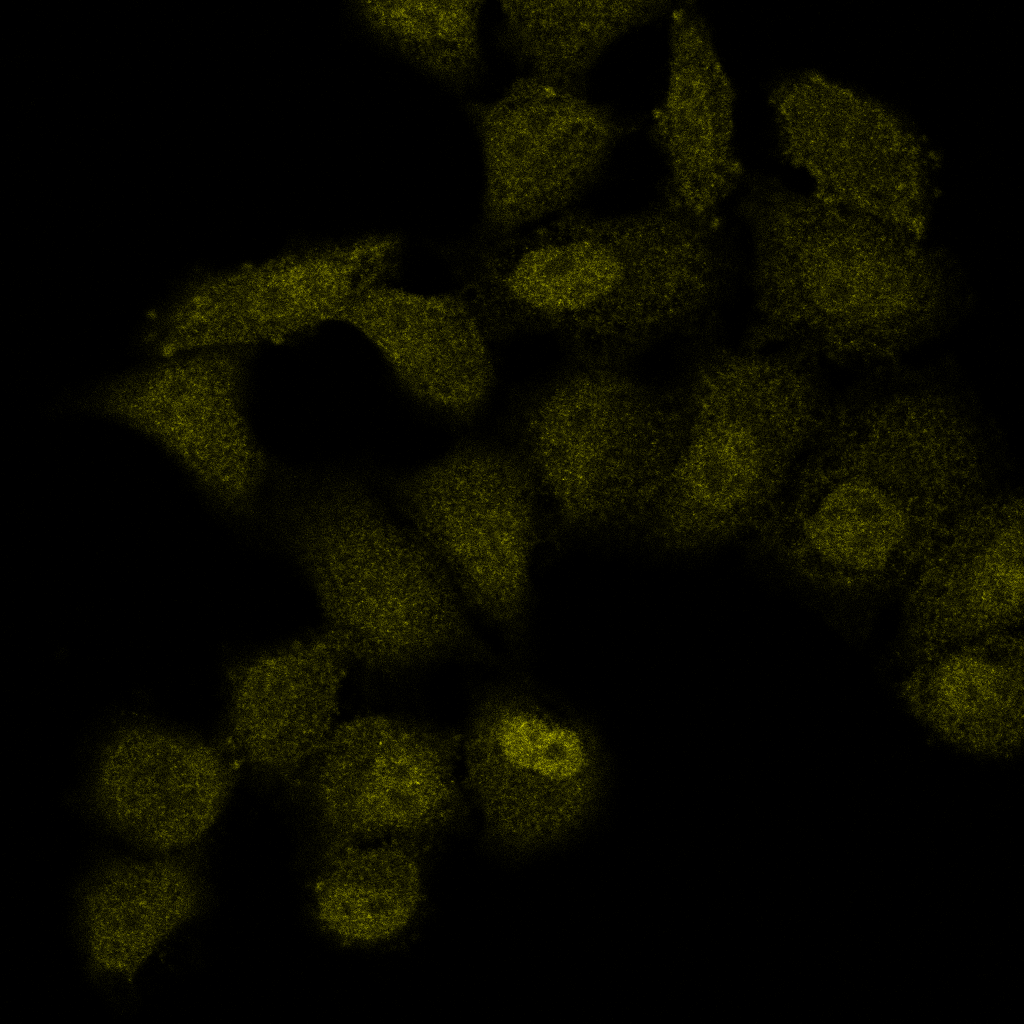

Supplement: Supplementary file 7 — Source data Fig. 5 [file 44318_2024_233_MOESM7_ESM.zip › 5A/HeLa sgATG16L1-2 AMDE TFE3_Series006_ch01_SV.tif]

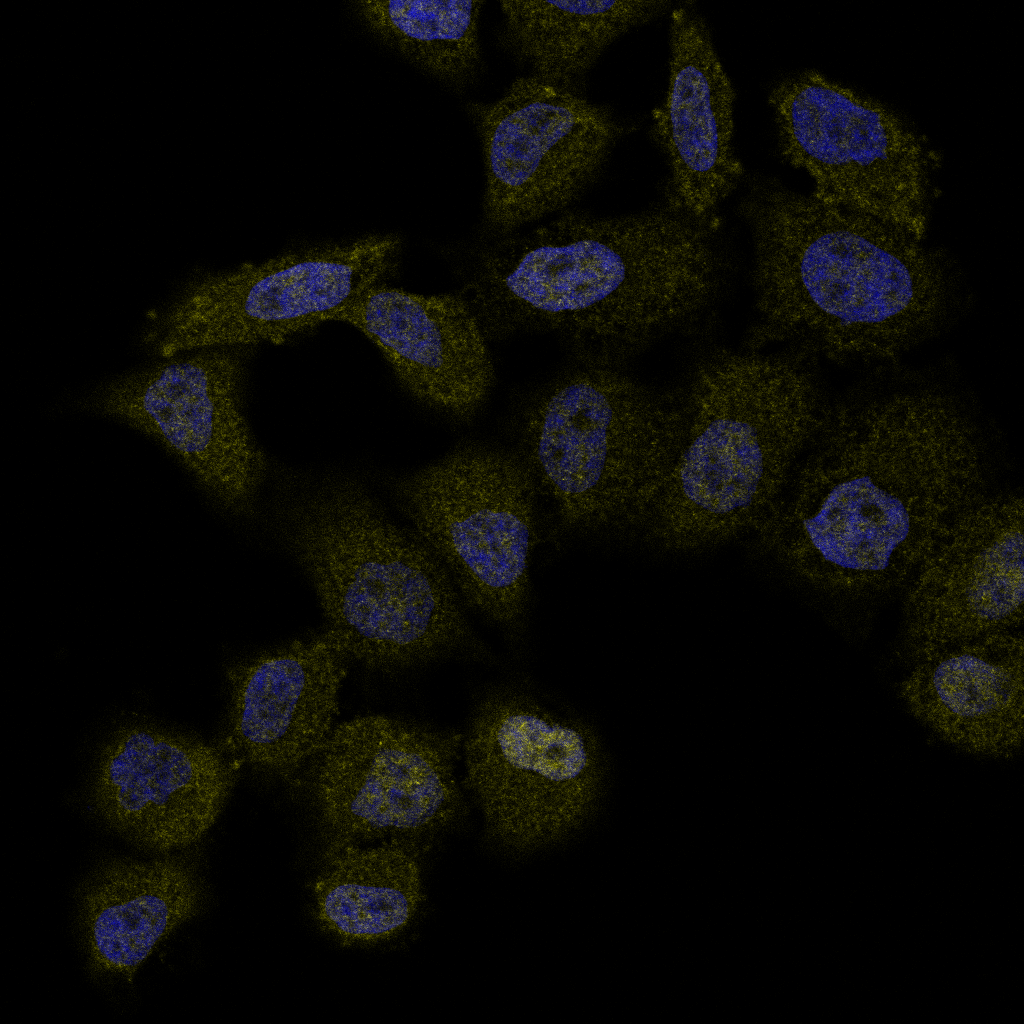

Supplement: Supplementary file 7 — Source data Fig. 5 [file 44318_2024_233_MOESM7_ESM.zip › 5A/HeLa sgATG16L1-2 AMDE TFE3_Series006_overlay.tif]

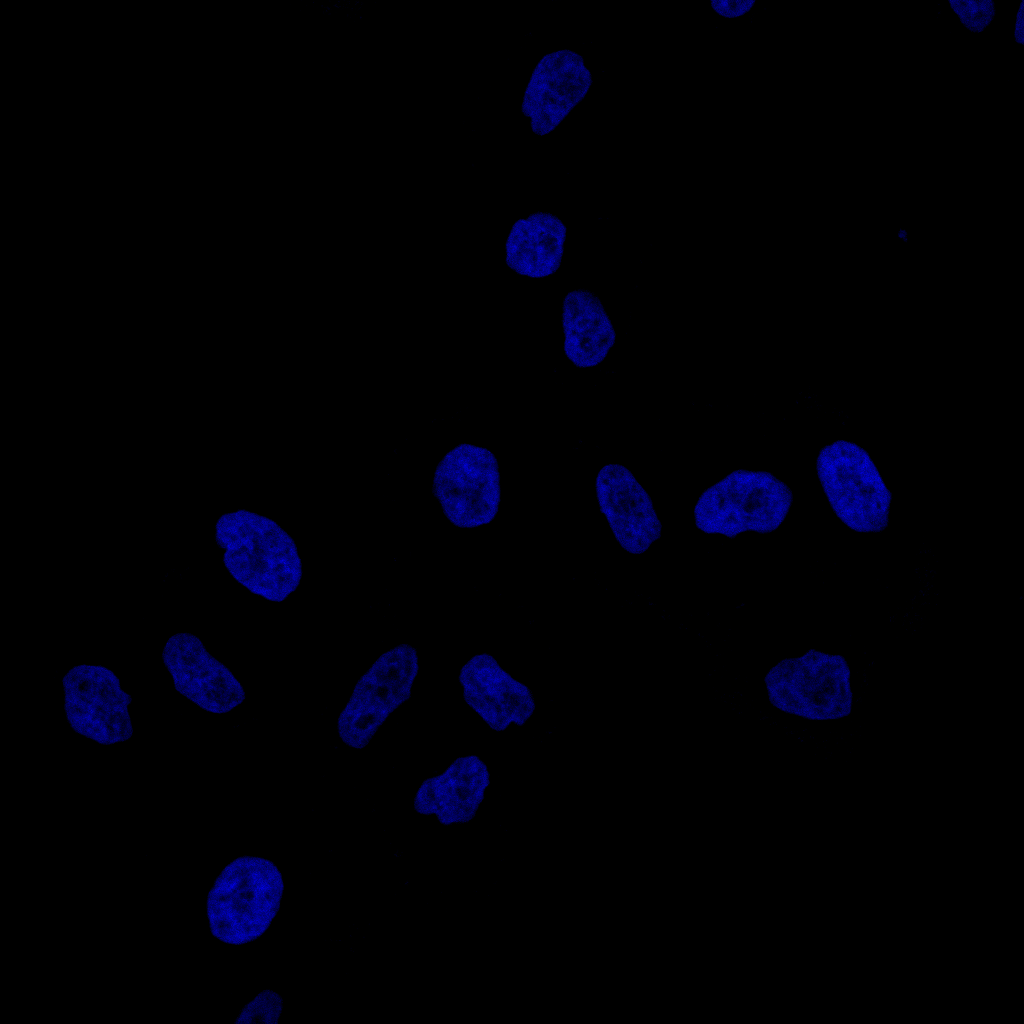

Supplement: Supplementary file 7 — Source data Fig. 5 [file 44318_2024_233_MOESM7_ESM.zip › 5A/HeLa sgATG16L1-2 Nic TFE3_Series001_ch00_SV.tif]

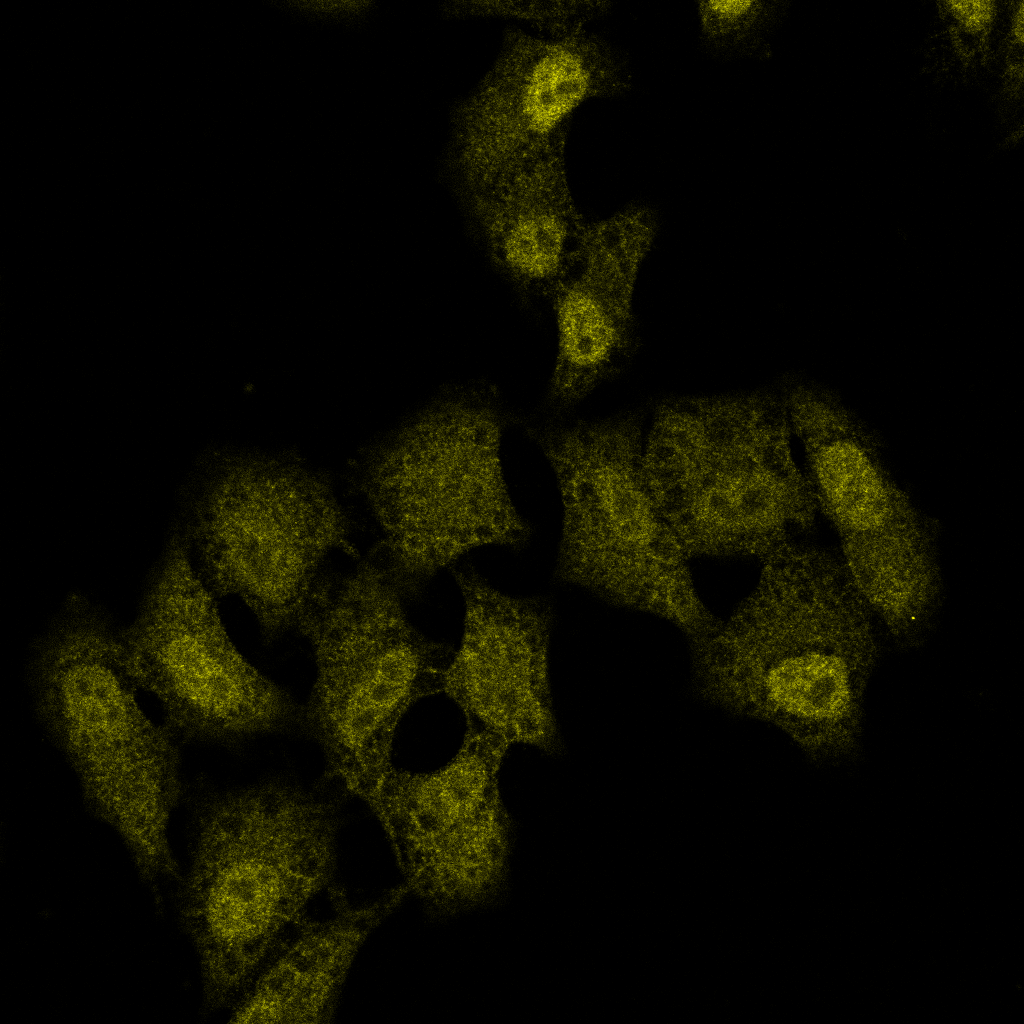

Supplement: Supplementary file 7 — Source data Fig. 5 [file 44318_2024_233_MOESM7_ESM.zip › 5A/HeLa sgATG16L1-2 Nic TFE3_Series001_ch01_SV.tif]

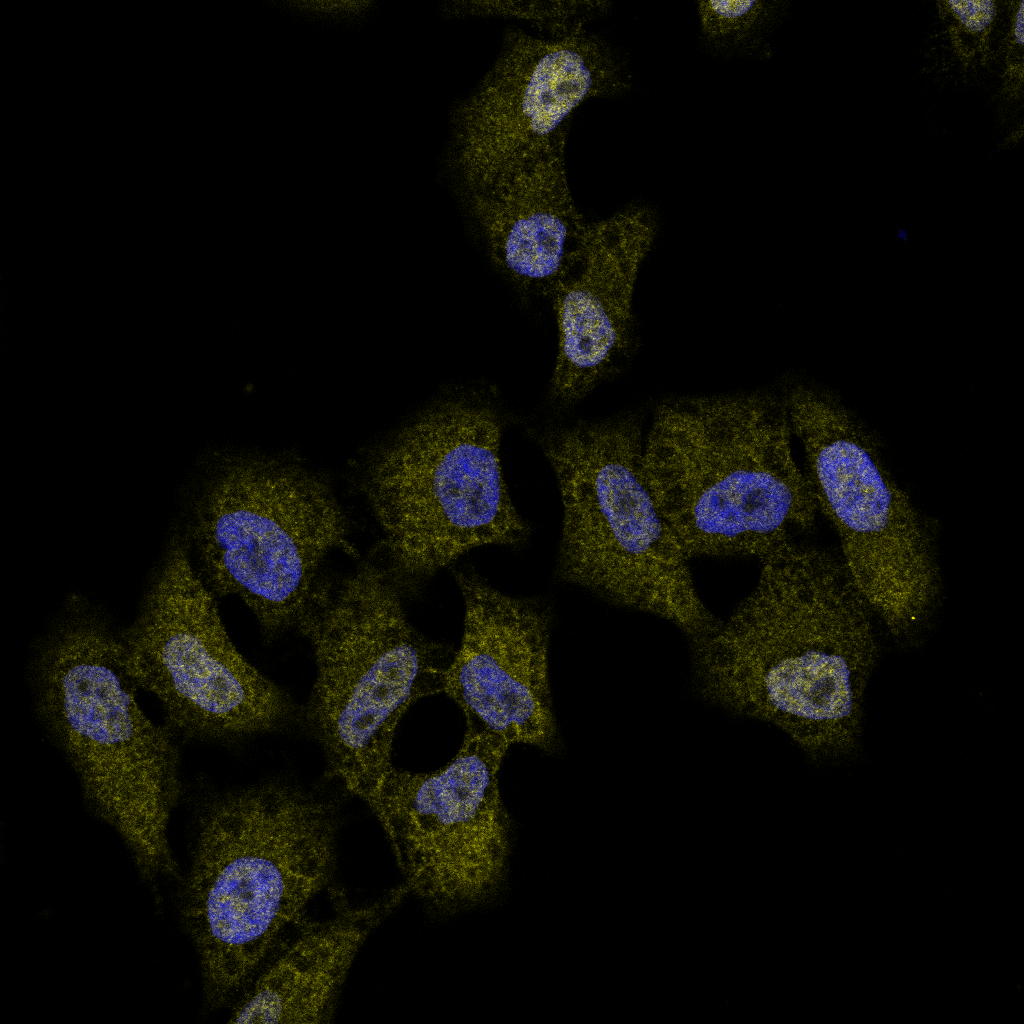

Supplement: Supplementary file 7 — Source data Fig. 5 [file 44318_2024_233_MOESM7_ESM.zip › 5A/HeLa sgATG16L1-2 Nic TFE3_Series001_overlay.tif]

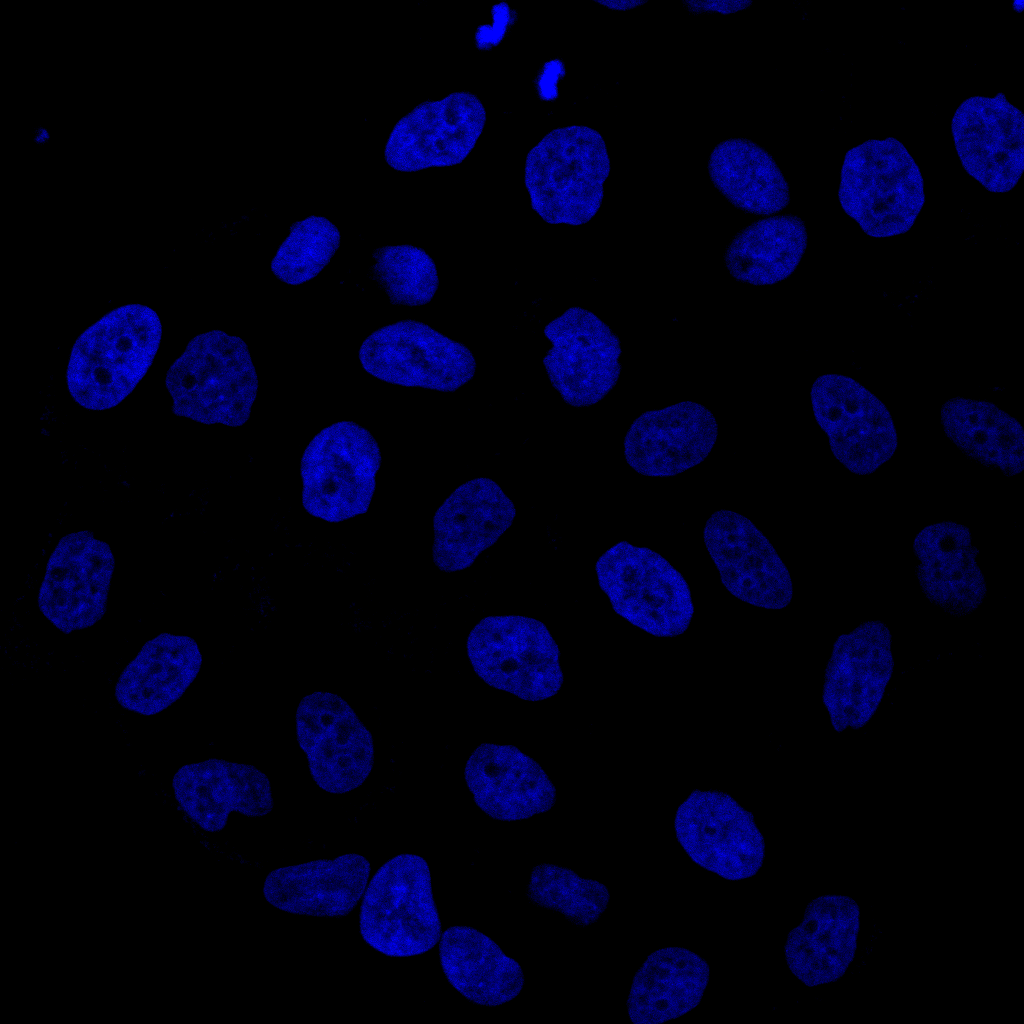

Supplement: Supplementary file 7 — Source data Fig. 5 [file 44318_2024_233_MOESM7_ESM.zip › 5A/HeLa sgATG16L1-2 veh TFE3_Series001_ch00_SV.tif]

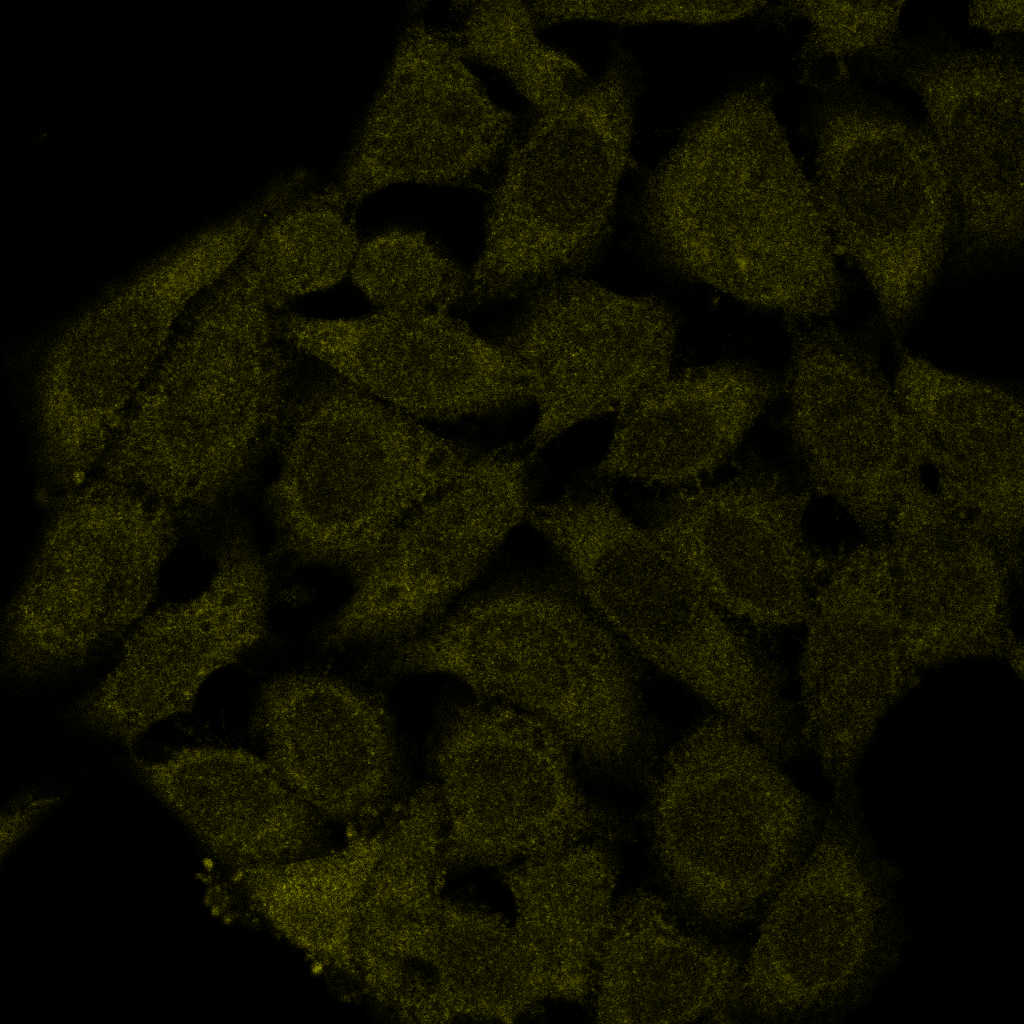

Supplement: Supplementary file 7 — Source data Fig. 5 [file 44318_2024_233_MOESM7_ESM.zip › 5A/HeLa sgATG16L1-2 veh TFE3_Series001_ch01_SV.tif]

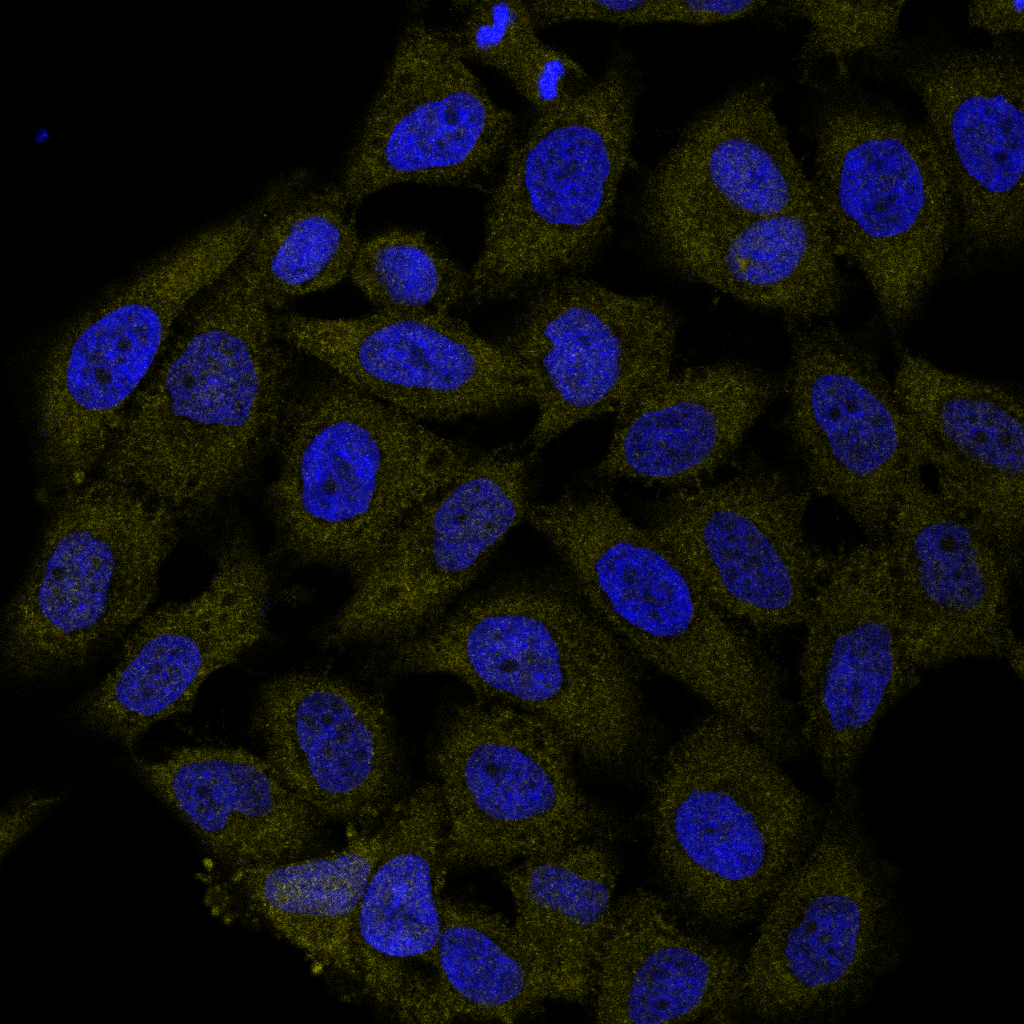

Supplement: Supplementary file 7 — Source data Fig. 5 [file 44318_2024_233_MOESM7_ESM.zip › 5A/HeLa sgATG16L1-2 veh TFE3_Series001_overlay.tif]

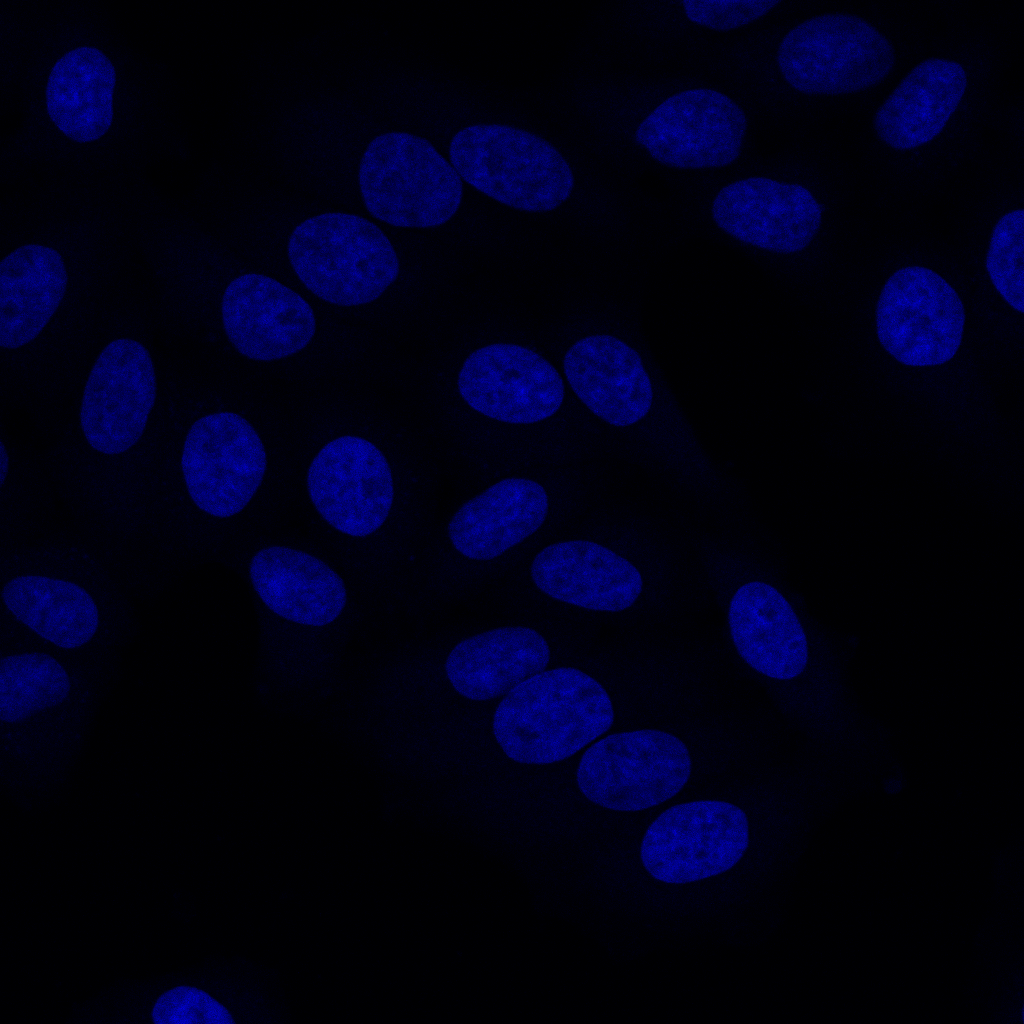

Supplement: Supplementary file 7 — Source data Fig. 5 [file 44318_2024_233_MOESM7_ESM.zip › 5A/HeLa sgCtrl AMDE-1 TFE3_Series002_ch00_SV.tif]

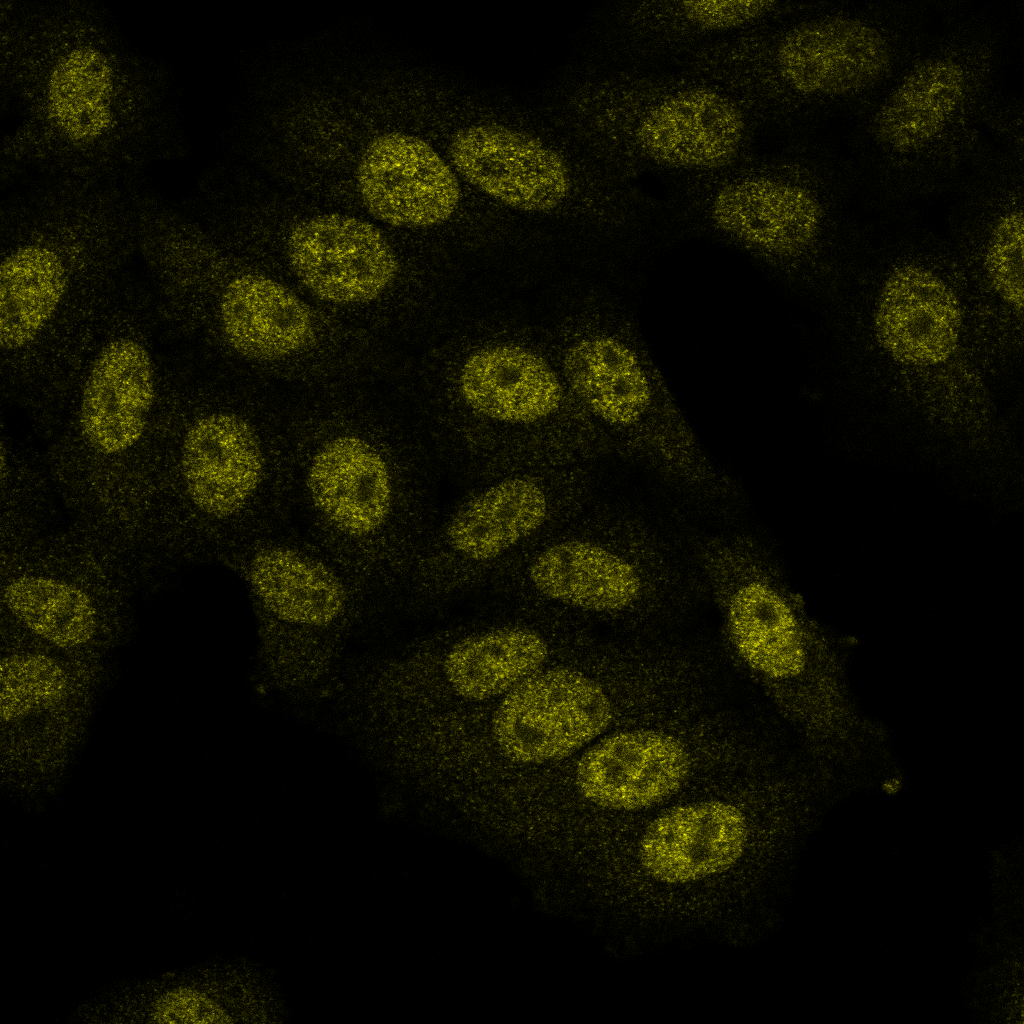

Supplement: Supplementary file 7 — Source data Fig. 5 [file 44318_2024_233_MOESM7_ESM.zip › 5A/HeLa sgCtrl AMDE-1 TFE3_Series002_ch01_SV.tif]

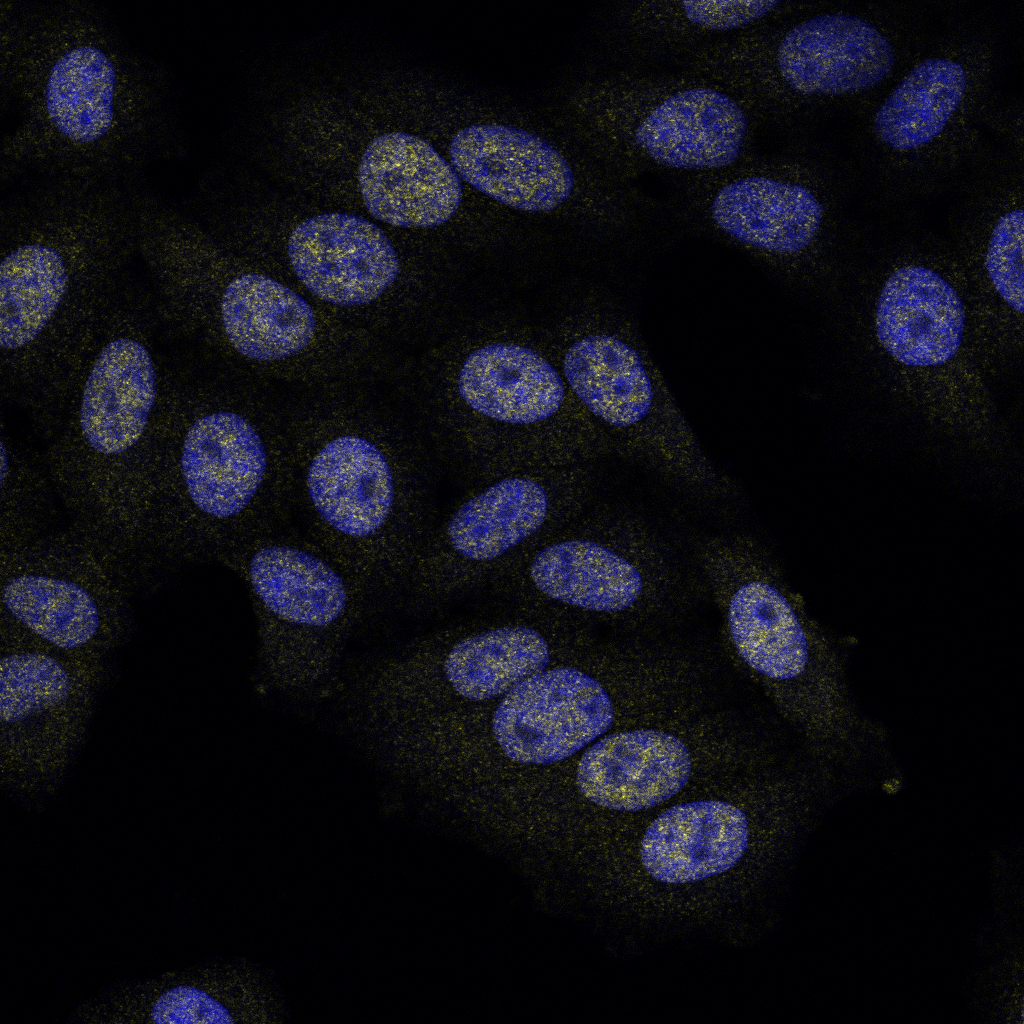

Supplement: Supplementary file 7 — Source data Fig. 5 [file 44318_2024_233_MOESM7_ESM.zip › 5A/HeLa sgCtrl AMDE-1 TFE3_Series002_overlay.tif]

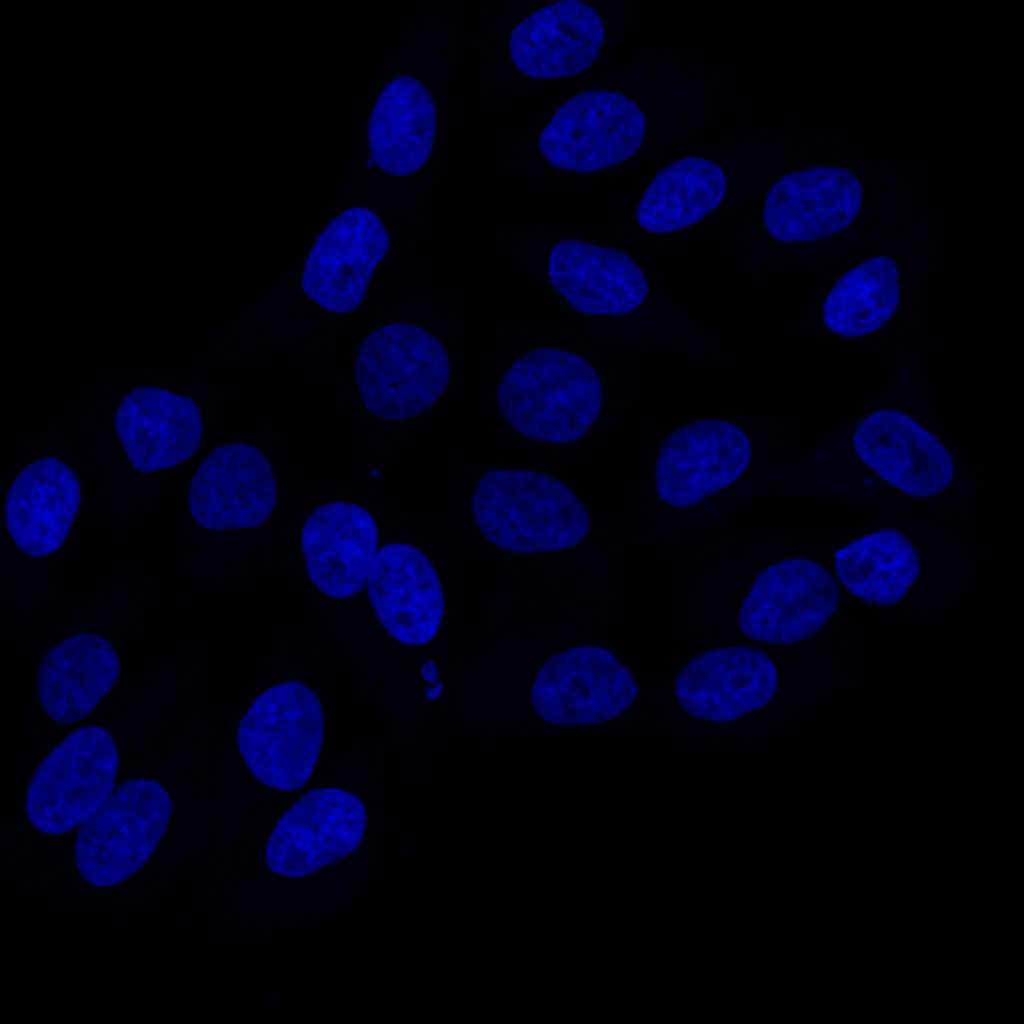

Supplement: Supplementary file 7 — Source data Fig. 5 [file 44318_2024_233_MOESM7_ESM.zip › 5A/HeLa sgCtrl Nic 6h TFE3_Series001_ch00_SV.tif]
