# Supplementary figures and images for: Non-autophagic Golgi-LC3 lipidation facilitates TFE3 stress response against Golgi dysfunction (part 3 of 3)
Source: EMBO J. 2024 Sep 16;43(21):5085–113. doi: 10.1038/s44318-024-00233-y (PMC11535212; doi:10.1038/s44318-024-00233-y)

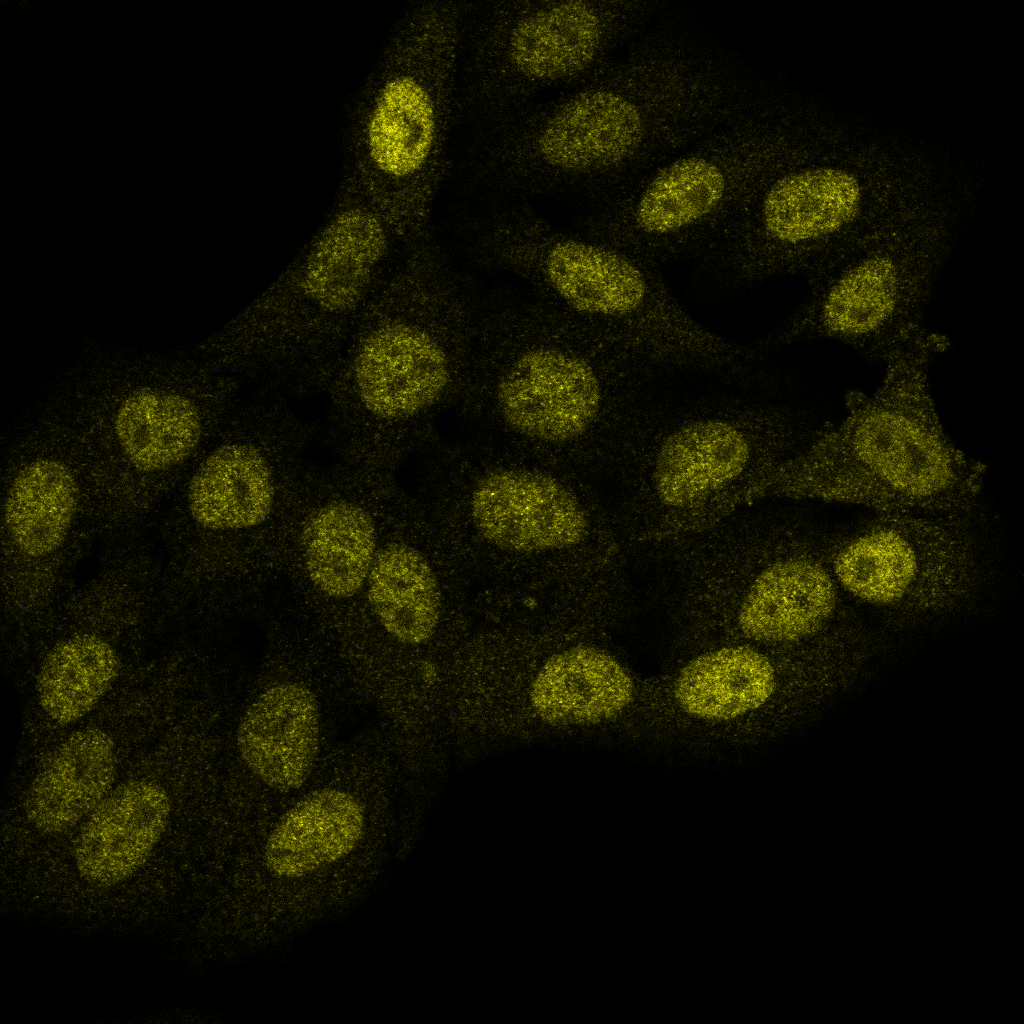

Supplement: Supplementary file 7 — Source data Fig. 5 [file 44318_2024_233_MOESM7_ESM.zip › 5A/HeLa sgCtrl Nic 6h TFE3_Series001_ch01_SV.tif]

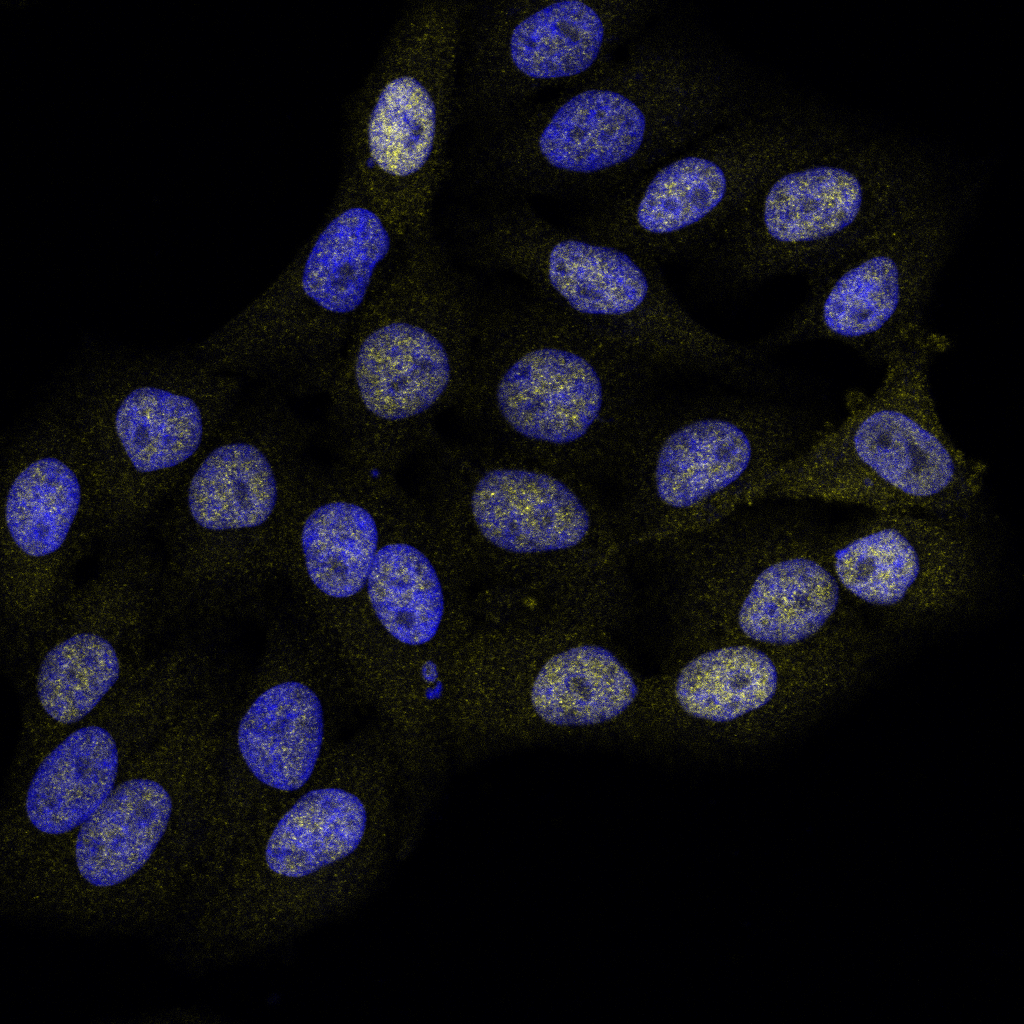

Supplement: Supplementary file 7 — Source data Fig. 5 [file 44318_2024_233_MOESM7_ESM.zip › 5A/HeLa sgCtrl Nic 6h TFE3_Series001_overlay.tif]

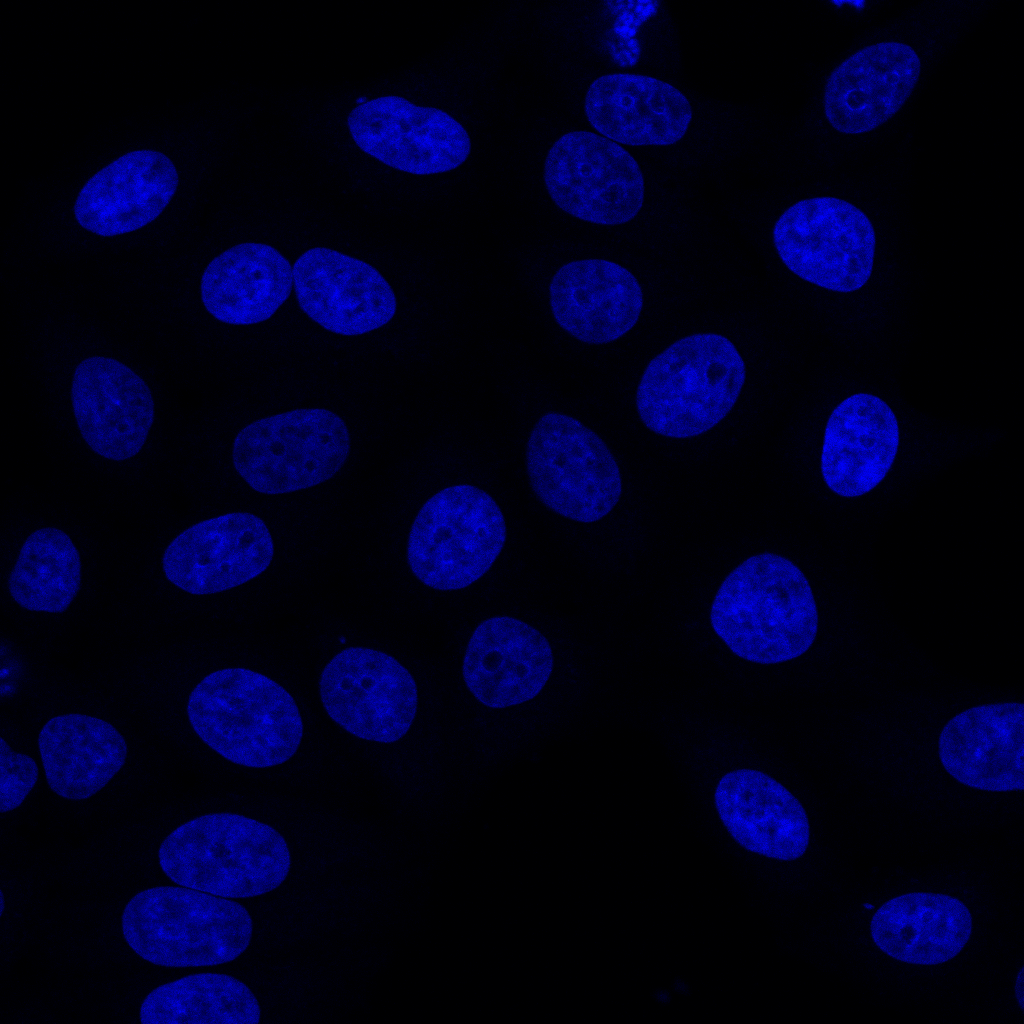

Supplement: Supplementary file 7 — Source data Fig. 5 [file 44318_2024_233_MOESM7_ESM.zip › 5A/HeLa sgCtrl veh TFE3_Series002_ch00_SV.tif]

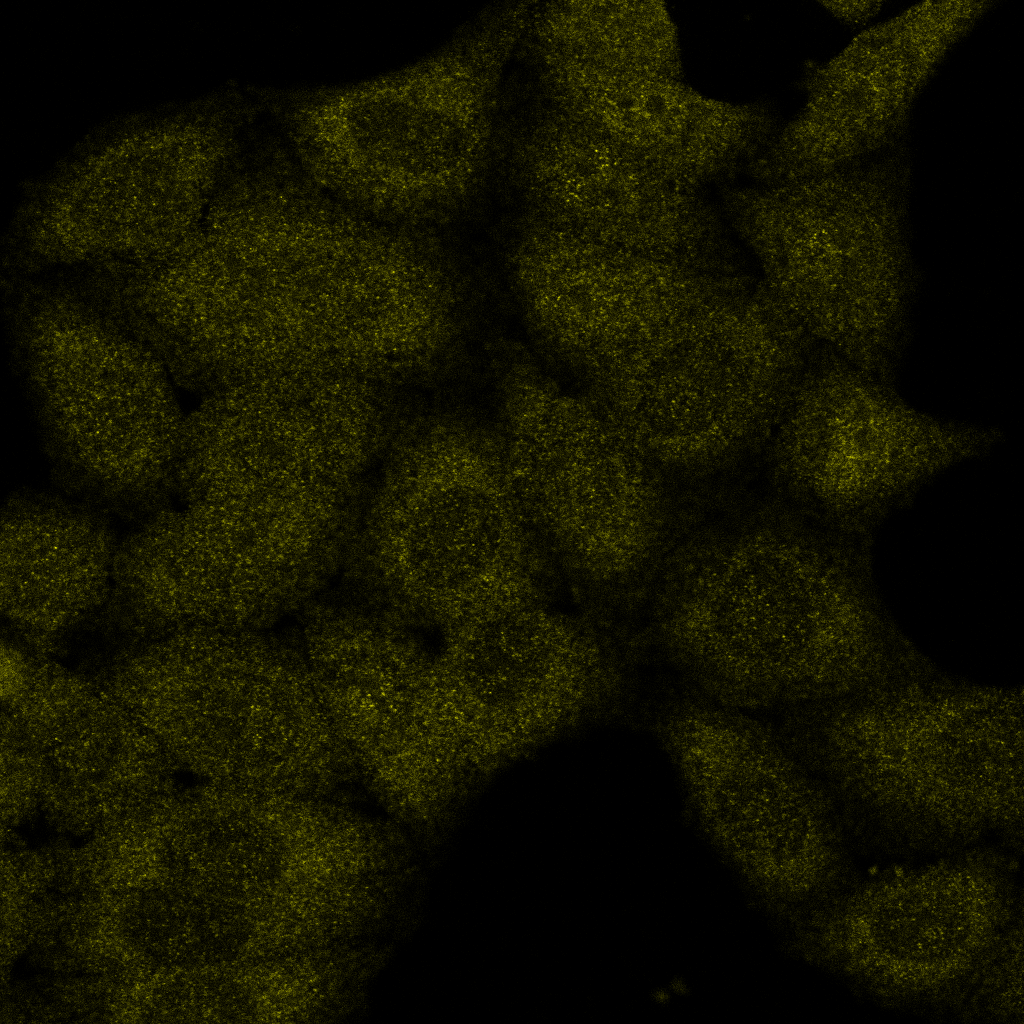

Supplement: Supplementary file 7 — Source data Fig. 5 [file 44318_2024_233_MOESM7_ESM.zip › 5A/HeLa sgCtrl veh TFE3_Series002_ch01_SV.tif]

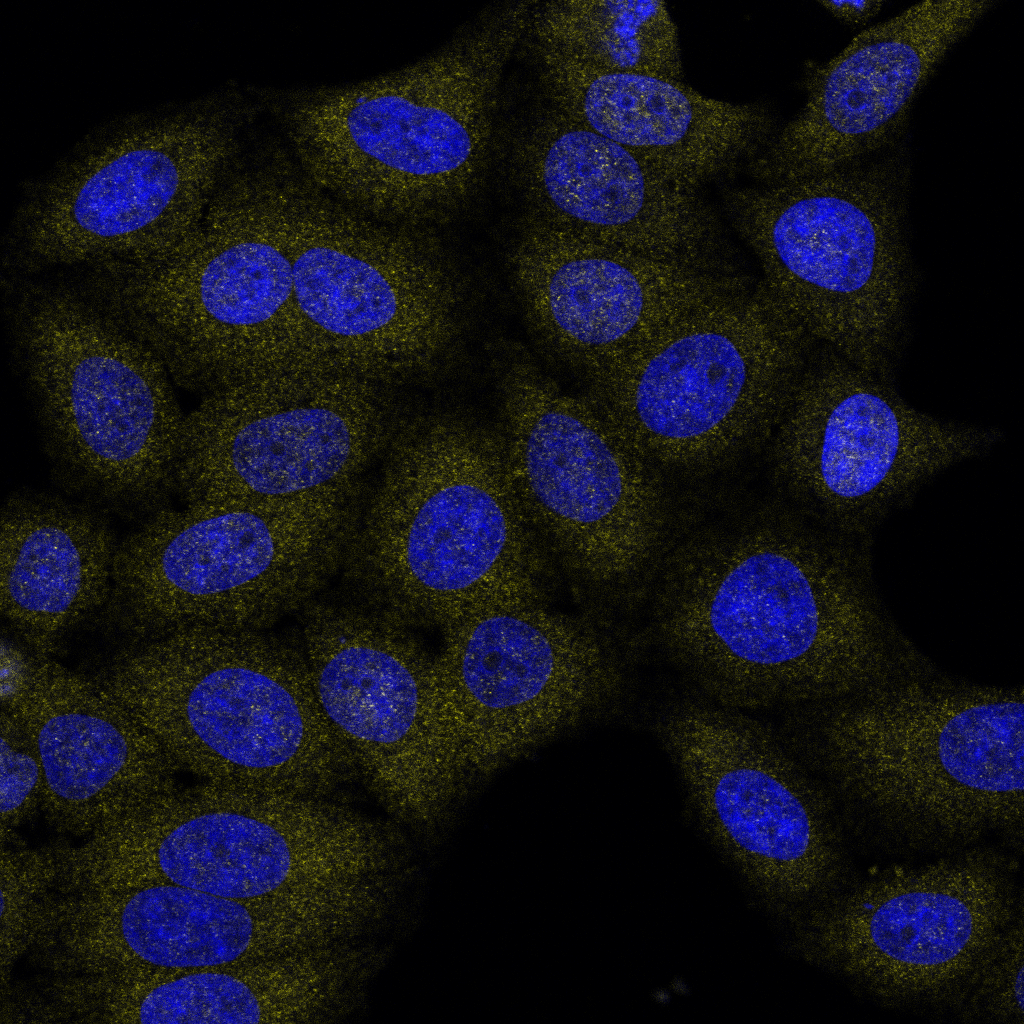

Supplement: Supplementary file 7 — Source data Fig. 5 [file 44318_2024_233_MOESM7_ESM.zip › 5A/HeLa sgCtrl veh TFE3_Series002_overlay.tif]

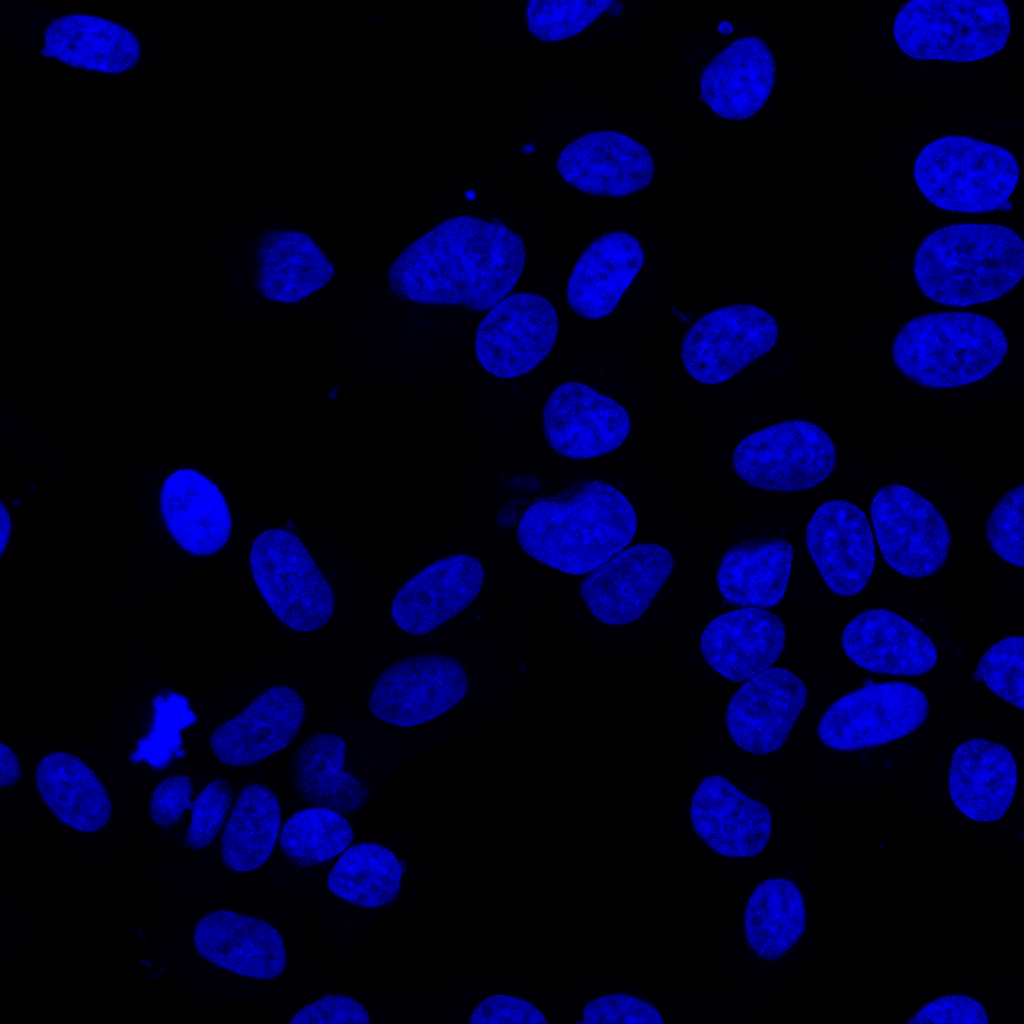

Supplement: Supplementary file 7 — Source data Fig. 5 [file 44318_2024_233_MOESM7_ESM.zip › 5B/HeLa sgATG16L1-1 DLK1 488 TFE3 594_Series004_ch00_SV.tif]

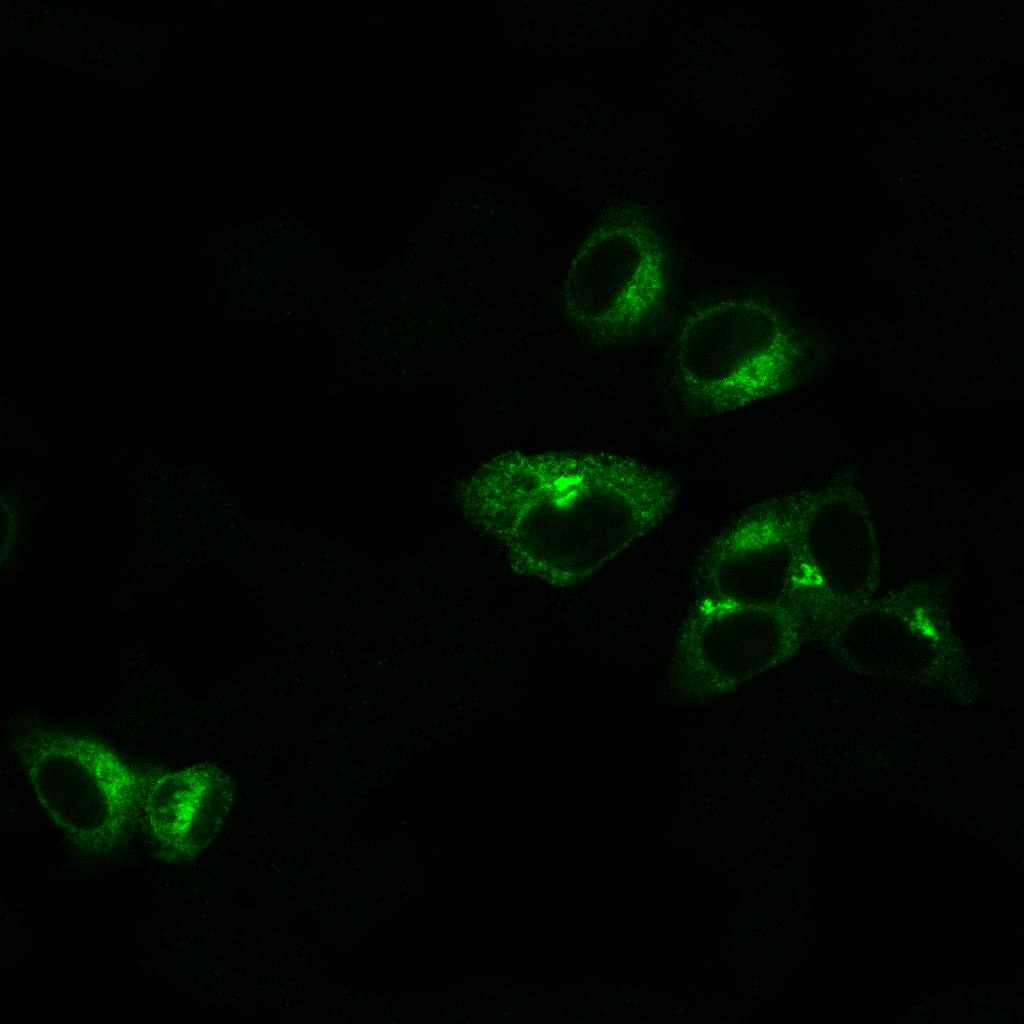

Supplement: Supplementary file 7 — Source data Fig. 5 [file 44318_2024_233_MOESM7_ESM.zip › 5B/HeLa sgATG16L1-1 DLK1 488 TFE3 594_Series004_ch01_SV.tif]

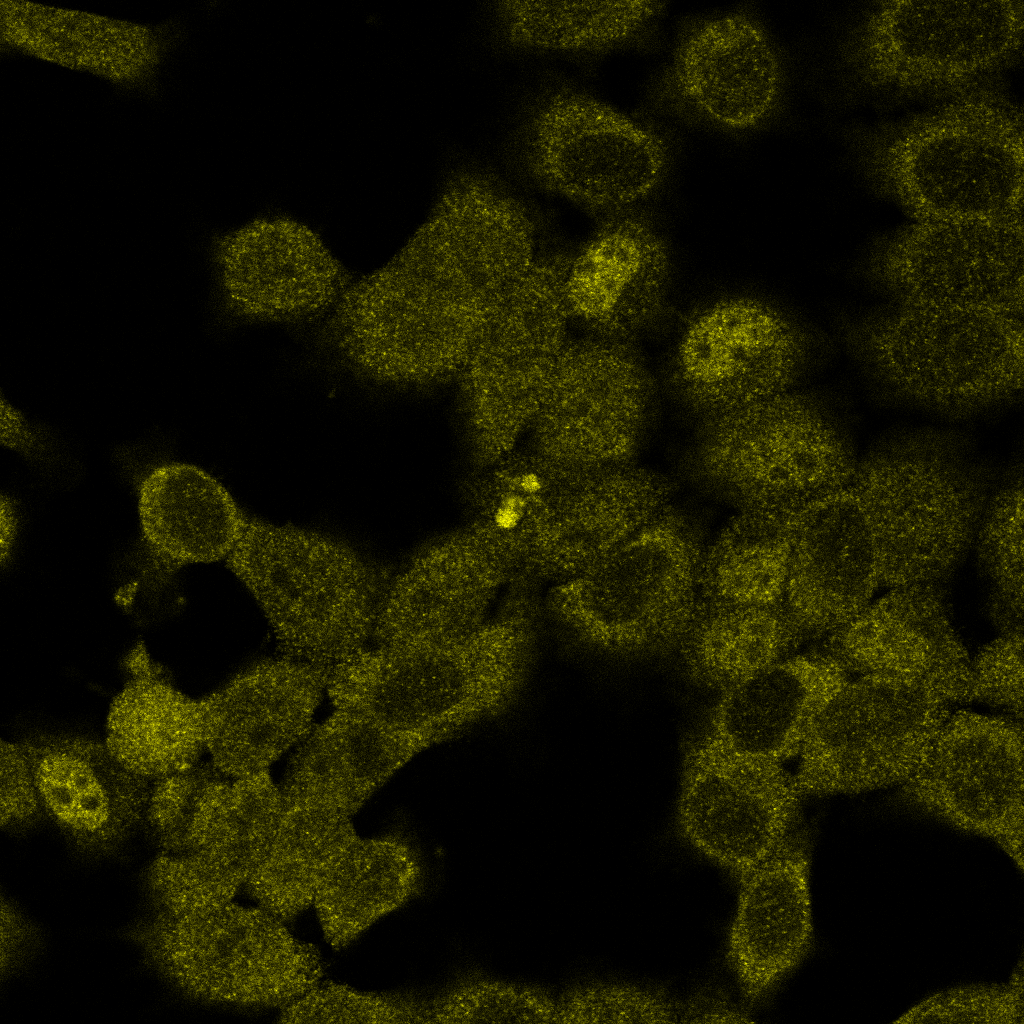

Supplement: Supplementary file 7 — Source data Fig. 5 [file 44318_2024_233_MOESM7_ESM.zip › 5B/HeLa sgATG16L1-1 DLK1 488 TFE3 594_Series004_ch02_SV.tif]

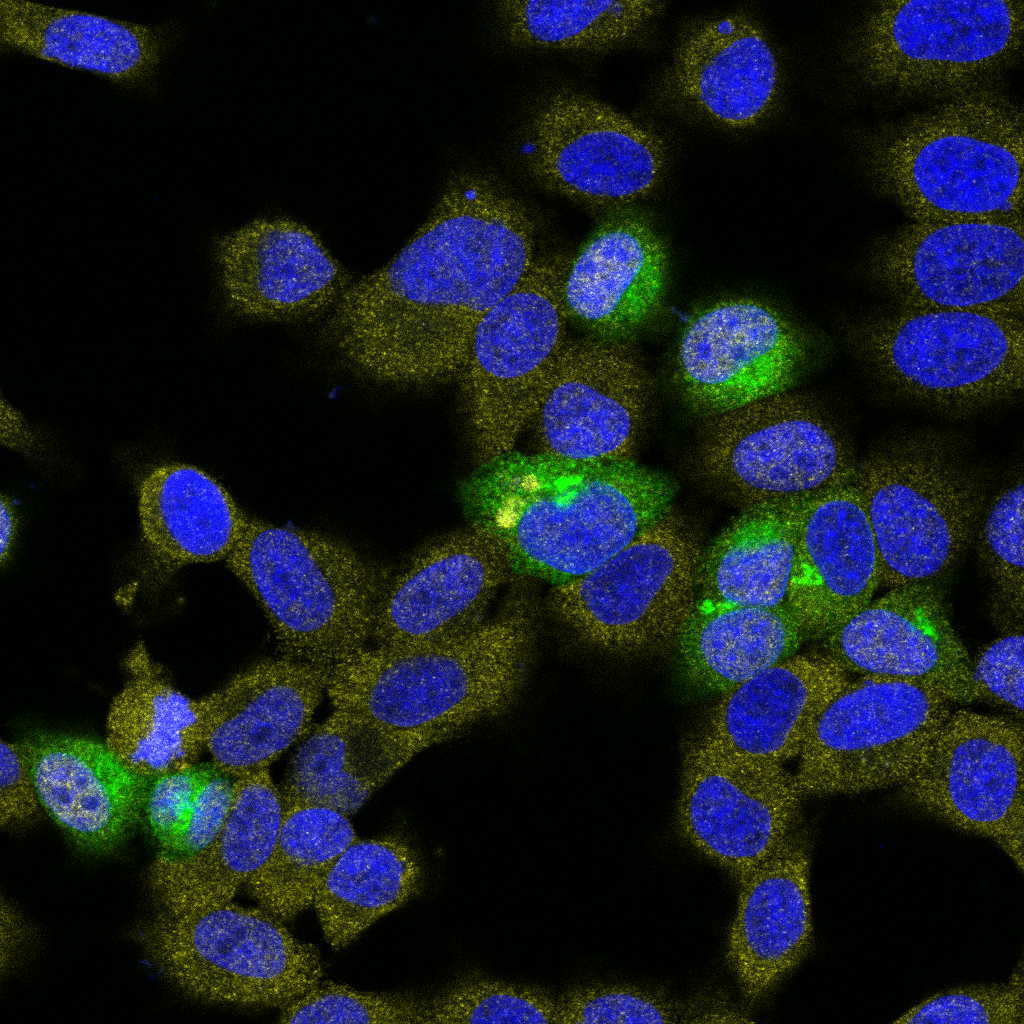

Supplement: Supplementary file 7 — Source data Fig. 5 [file 44318_2024_233_MOESM7_ESM.zip › 5B/HeLa sgATG16L1-1 DLK1 488 TFE3 594_Series004_overlay.tif]

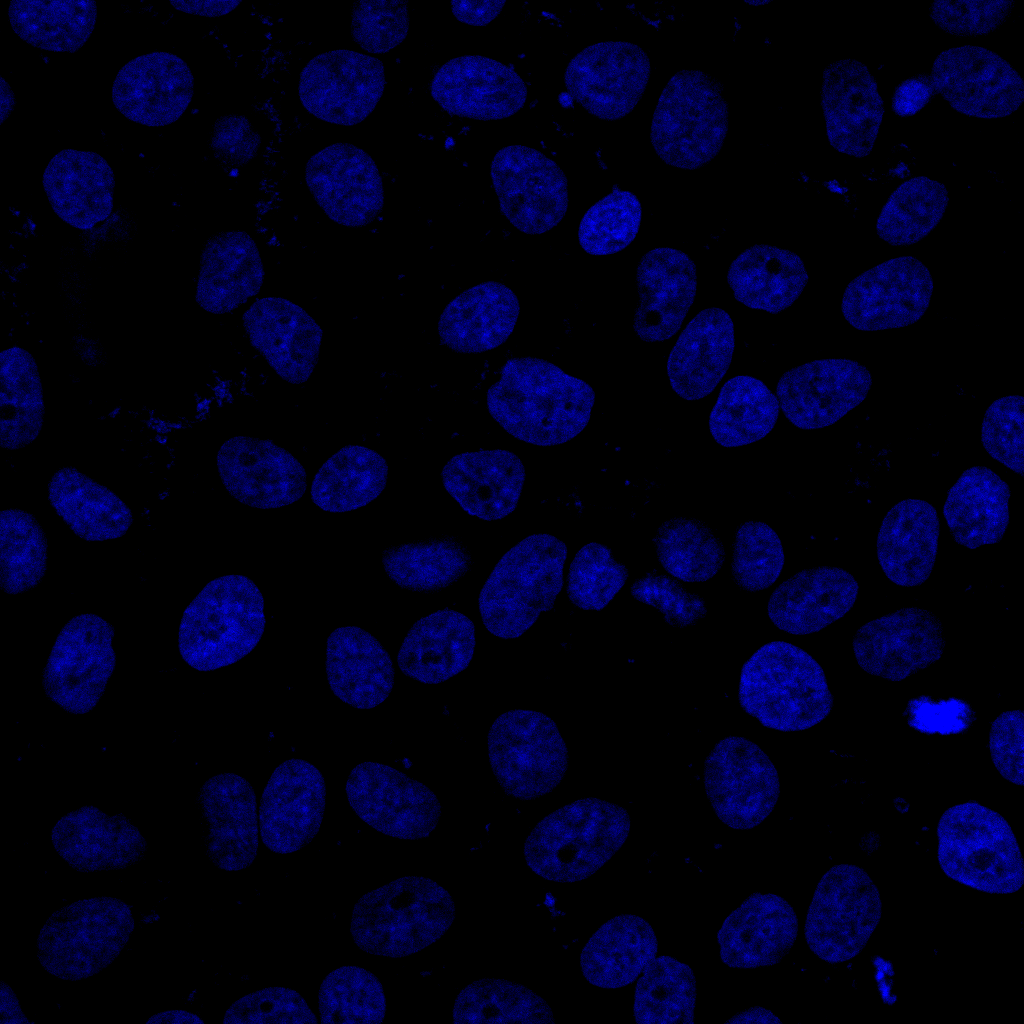

Supplement: Supplementary file 7 — Source data Fig. 5 [file 44318_2024_233_MOESM7_ESM.zip › 5B/HeLa sgATG16L1-2 DLK1 488 TFE3 594_Series007_ch00_SV.tif]

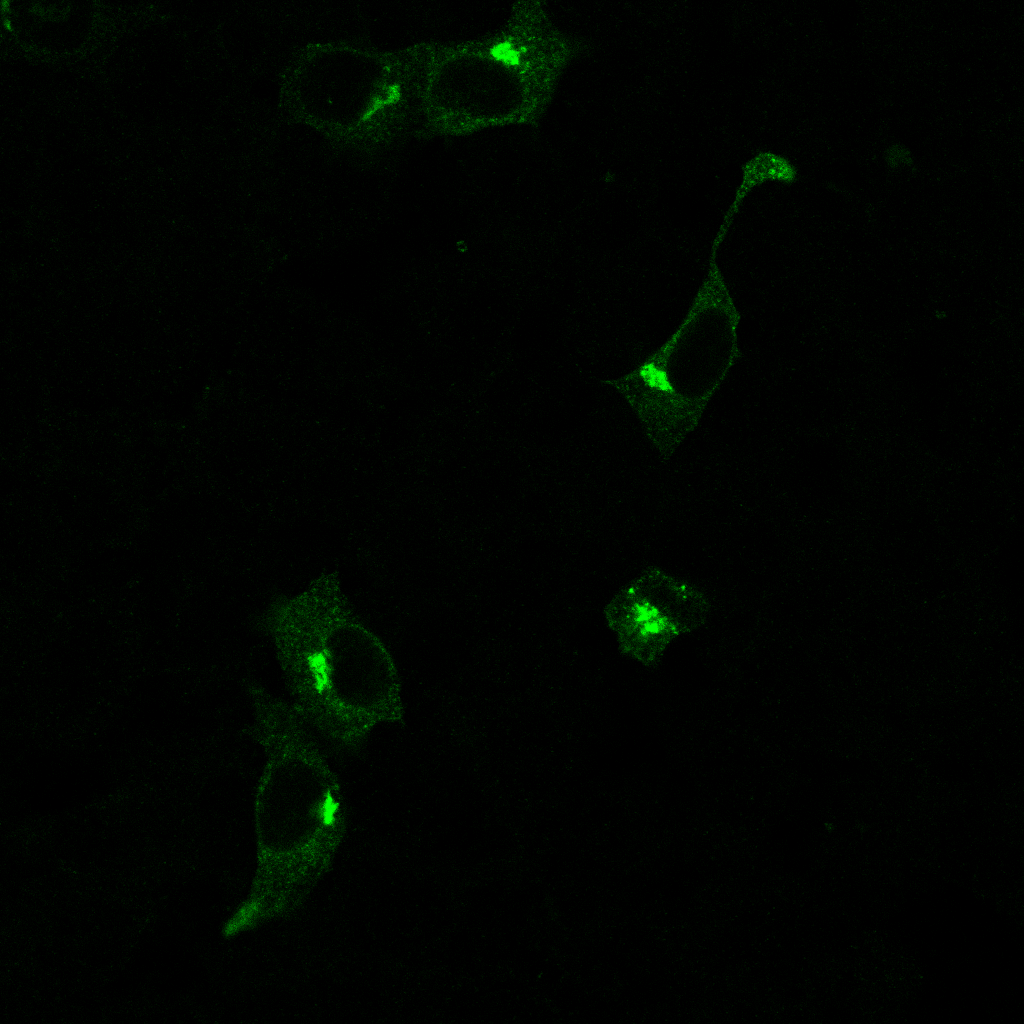

Supplement: Supplementary file 7 — Source data Fig. 5 [file 44318_2024_233_MOESM7_ESM.zip › 5B/HeLa sgATG16L1-2 DLK1 488 TFE3 594_Series007_ch01_SV.tif]

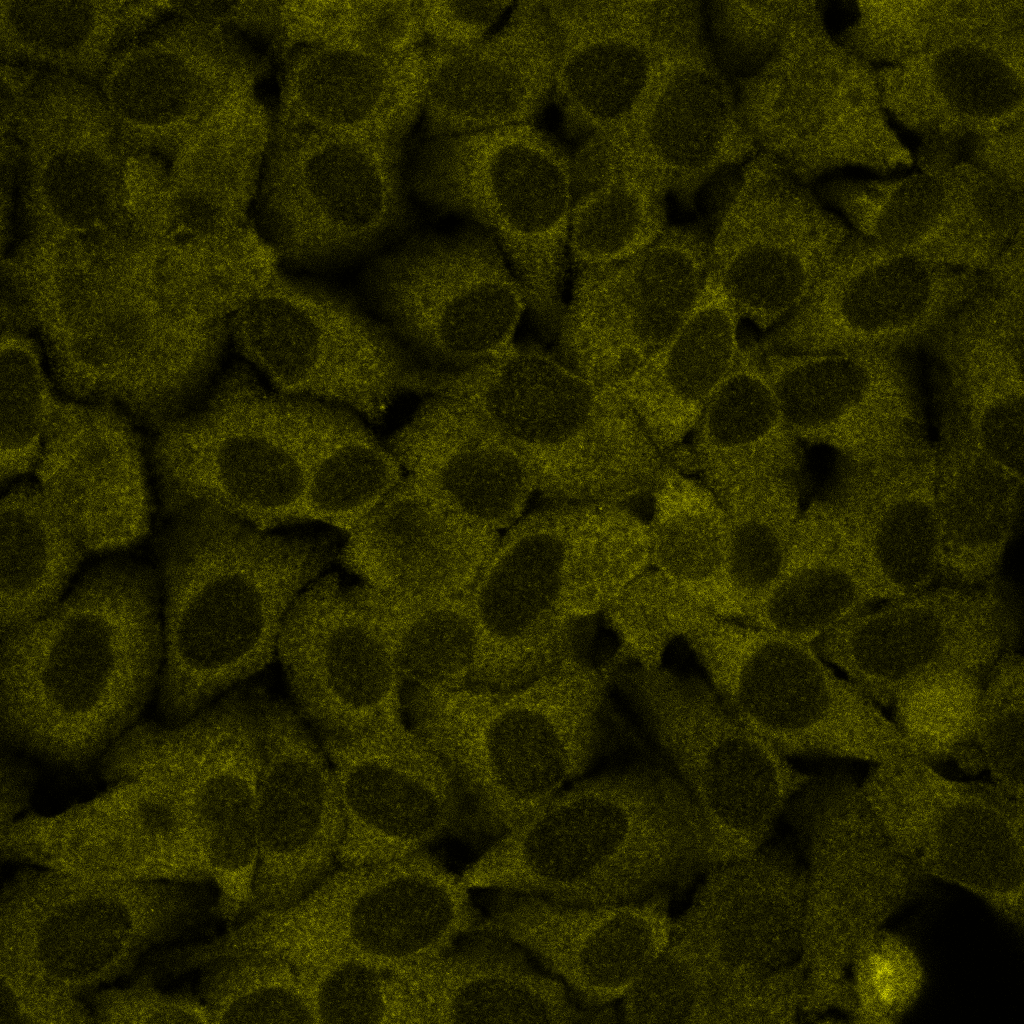

Supplement: Supplementary file 7 — Source data Fig. 5 [file 44318_2024_233_MOESM7_ESM.zip › 5B/HeLa sgATG16L1-2 DLK1 488 TFE3 594_Series007_ch02_SV.tif]

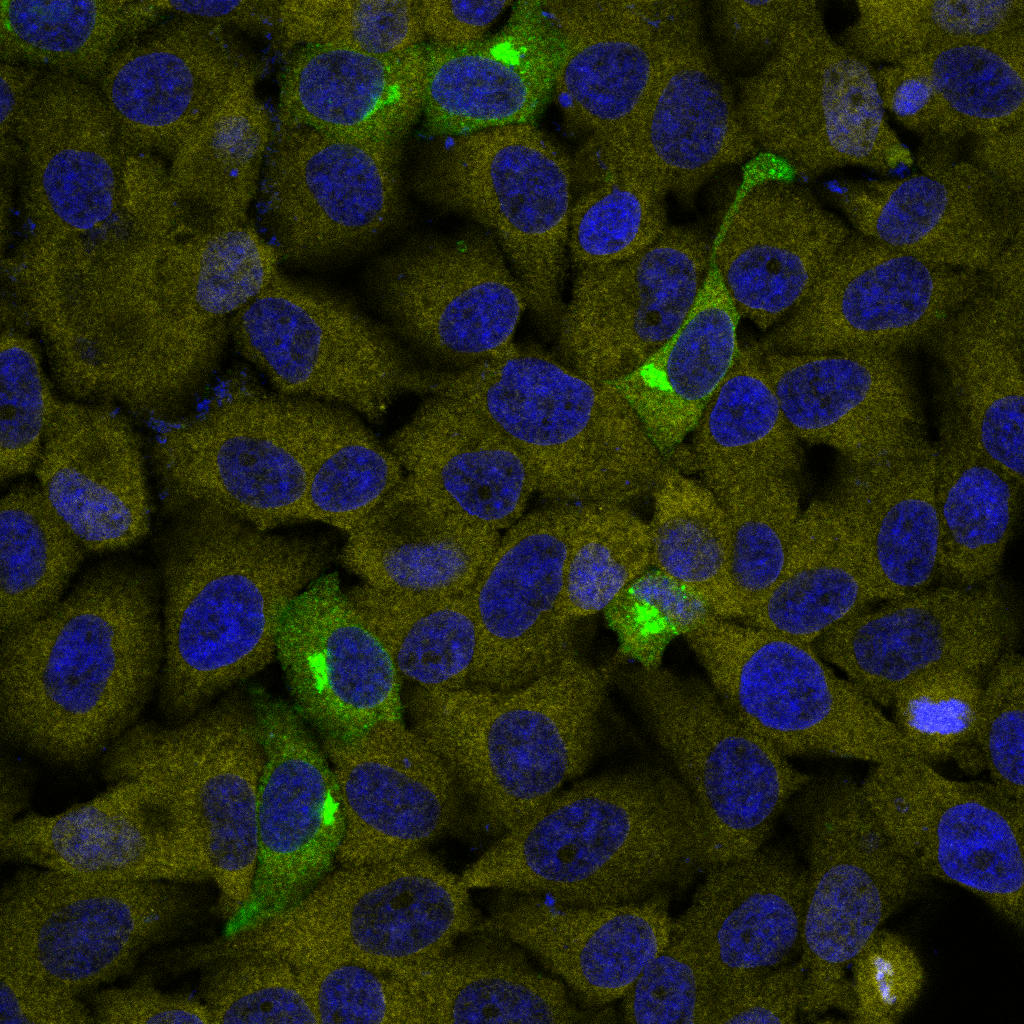

Supplement: Supplementary file 7 — Source data Fig. 5 [file 44318_2024_233_MOESM7_ESM.zip › 5B/HeLa sgATG16L1-2 DLK1 488 TFE3 594_Series007_overlay.tif]

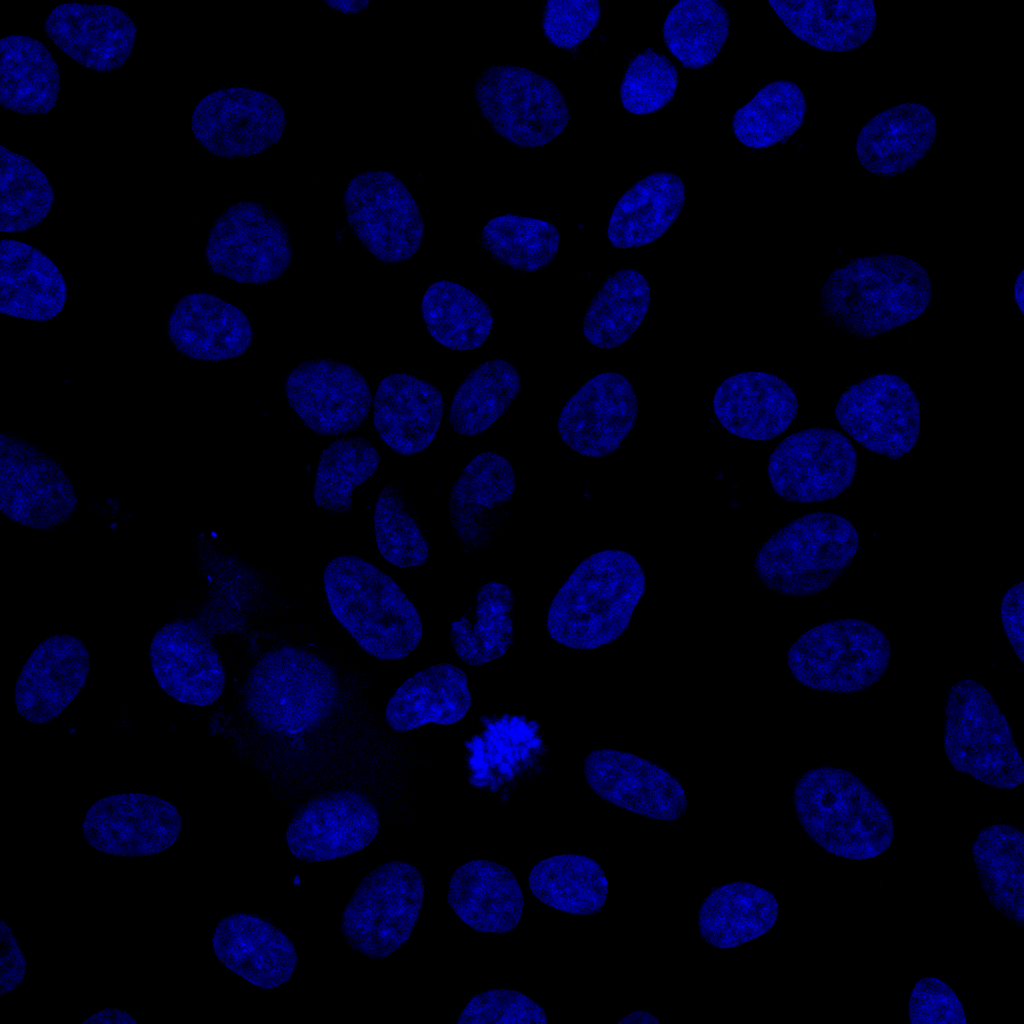

Supplement: Supplementary file 7 — Source data Fig. 5 [file 44318_2024_233_MOESM7_ESM.zip › 5B/HeLa sgCtrl DLK1 488 TFE3 594_Series001_ch00_SV.tif]

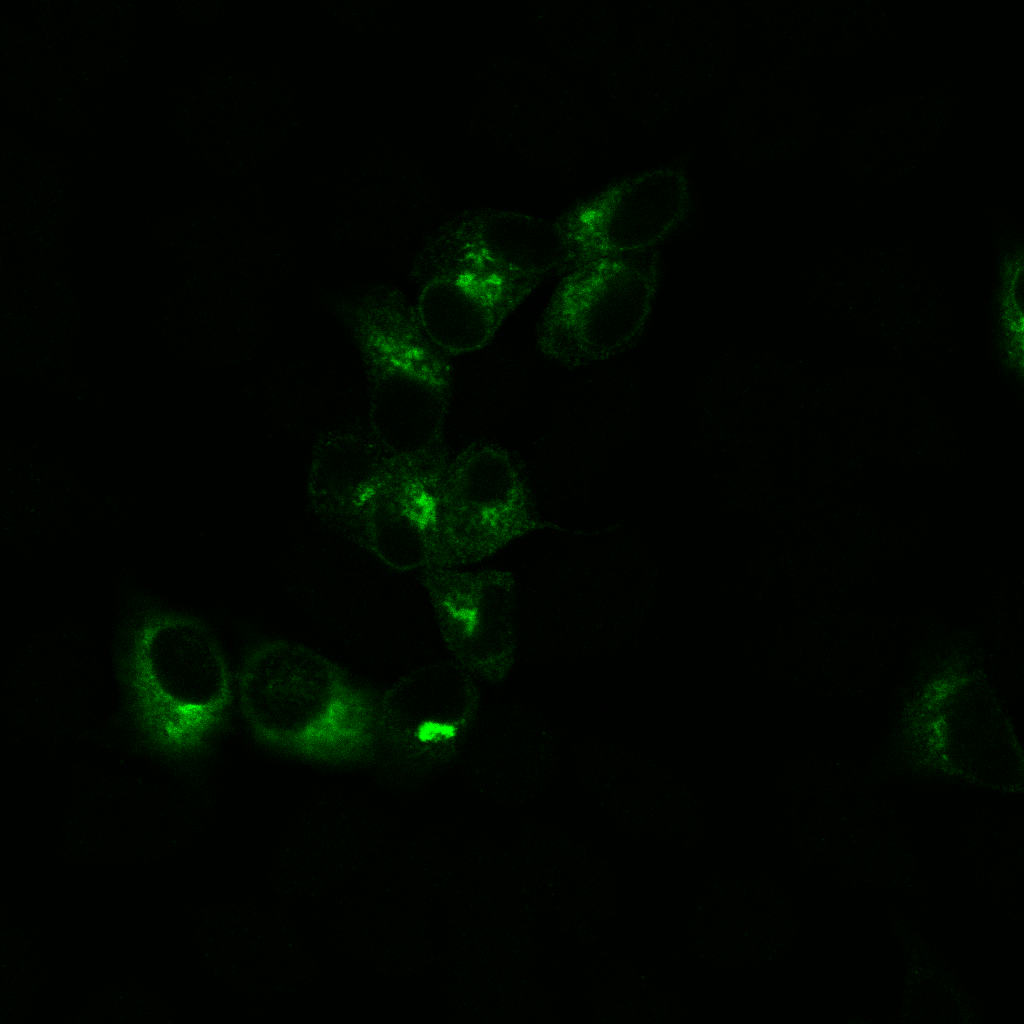

Supplement: Supplementary file 7 — Source data Fig. 5 [file 44318_2024_233_MOESM7_ESM.zip › 5B/HeLa sgCtrl DLK1 488 TFE3 594_Series001_ch01_SV.tif]

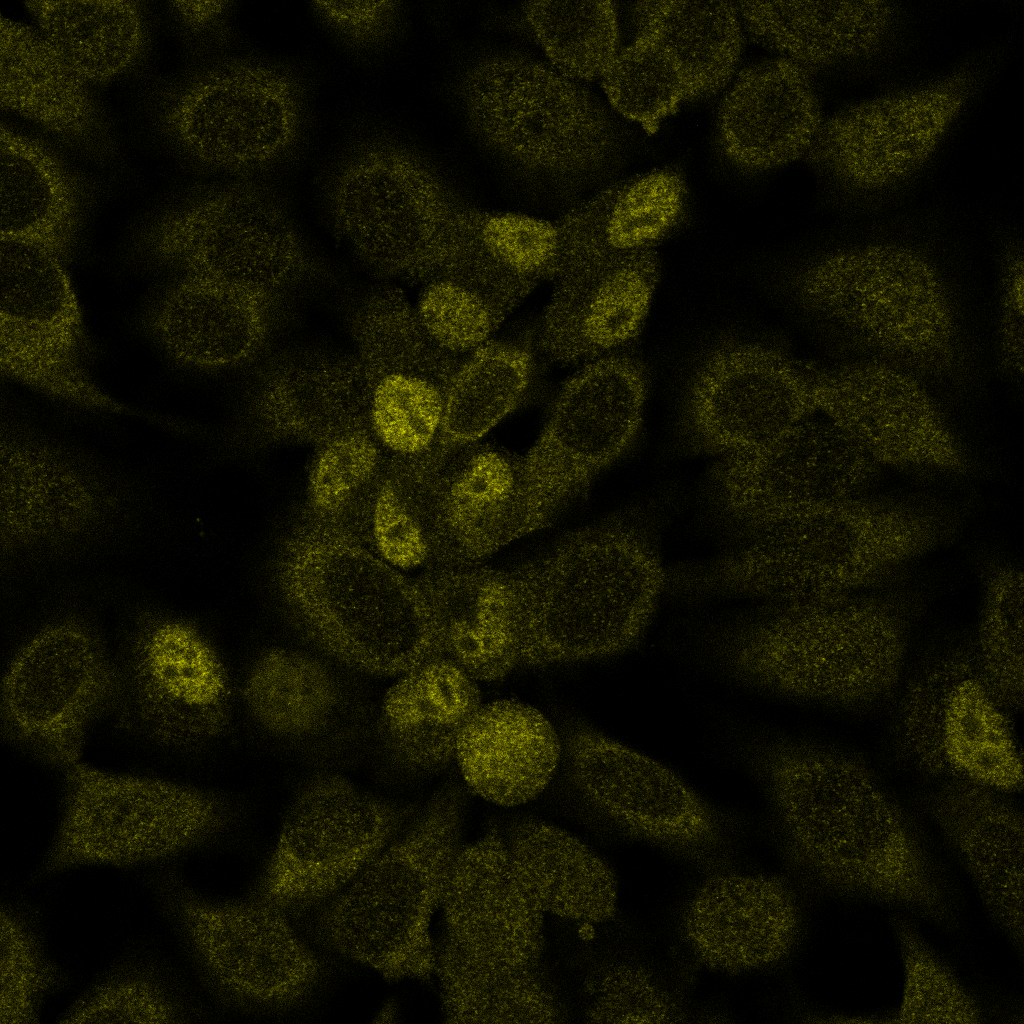

Supplement: Supplementary file 7 — Source data Fig. 5 [file 44318_2024_233_MOESM7_ESM.zip › 5B/HeLa sgCtrl DLK1 488 TFE3 594_Series001_ch02_SV.tif]

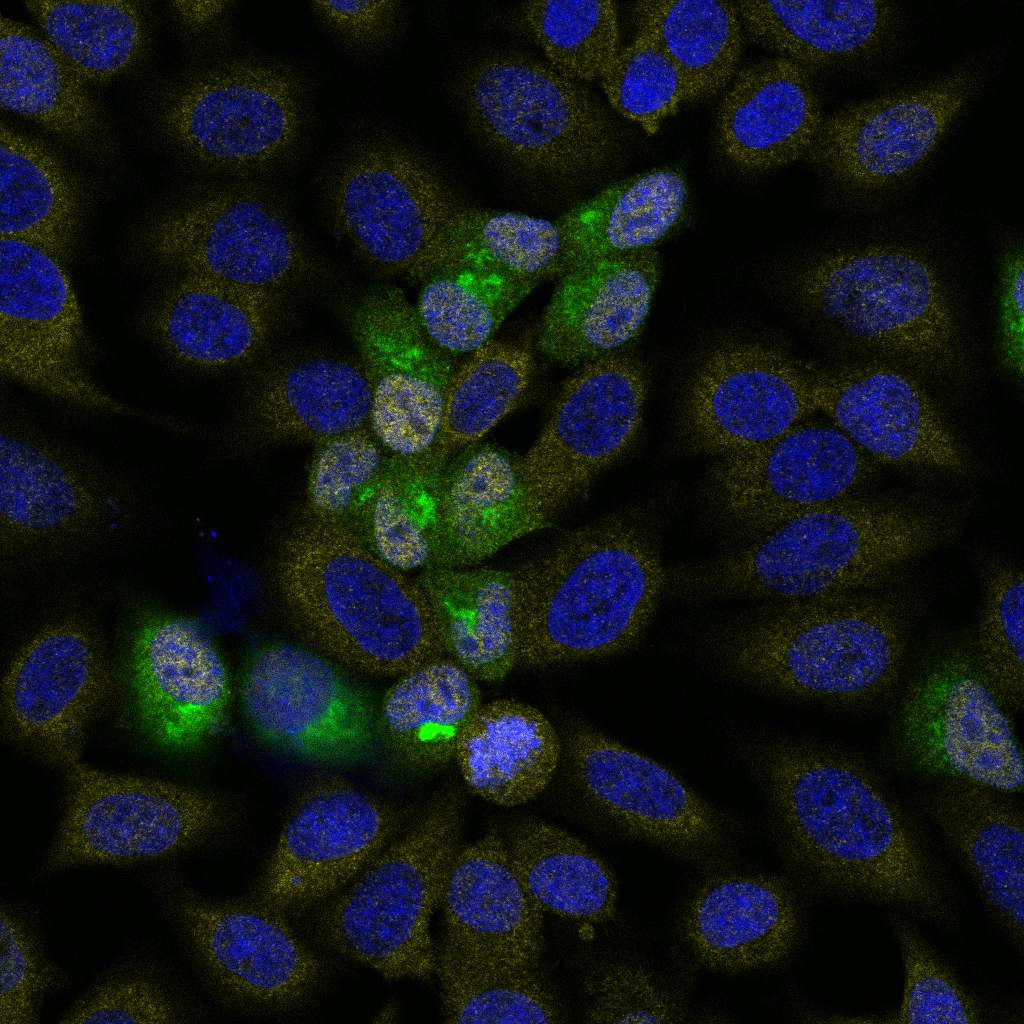

Supplement: Supplementary file 7 — Source data Fig. 5 [file 44318_2024_233_MOESM7_ESM.zip › 5B/HeLa sgCtrl DLK1 488 TFE3 594_Series001_overlay.tif]

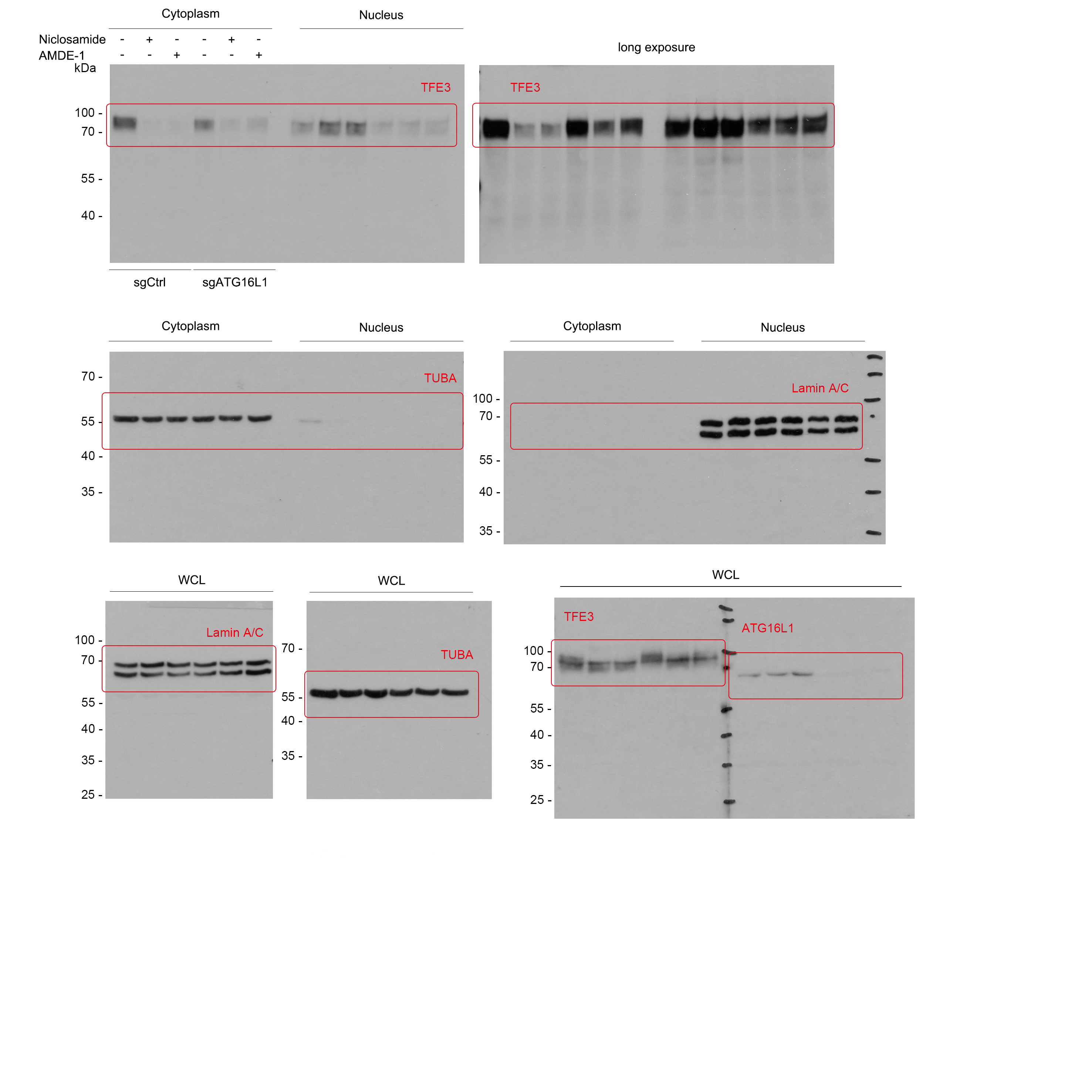

Supplement: Supplementary file 7 — Source data Fig. 5 [file 44318_2024_233_MOESM7_ESM.zip › 5E/Figure 5E.png]

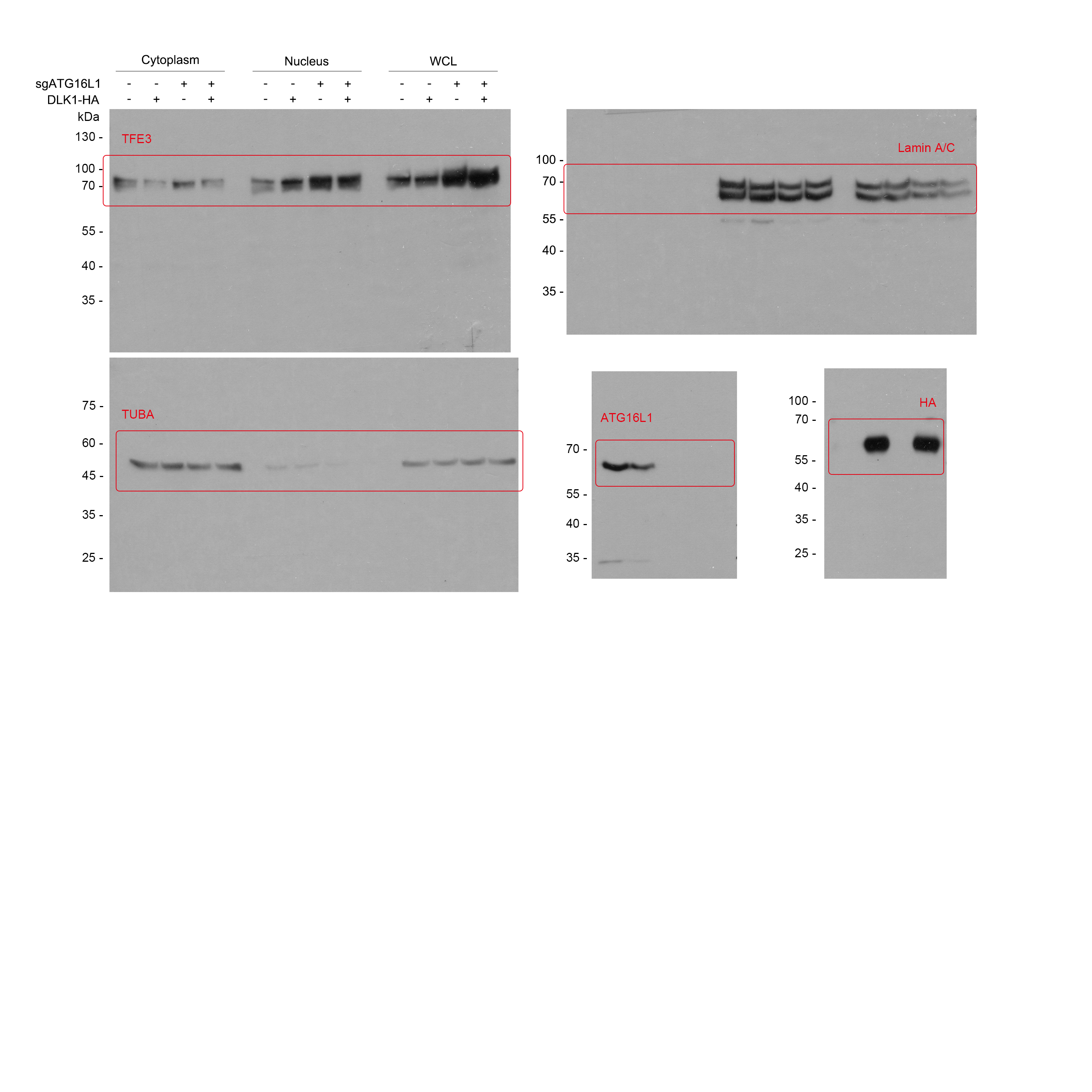

Supplement: Supplementary file 7 — Source data Fig. 5 [file 44318_2024_233_MOESM7_ESM.zip › 5F/Figure 5F.png]

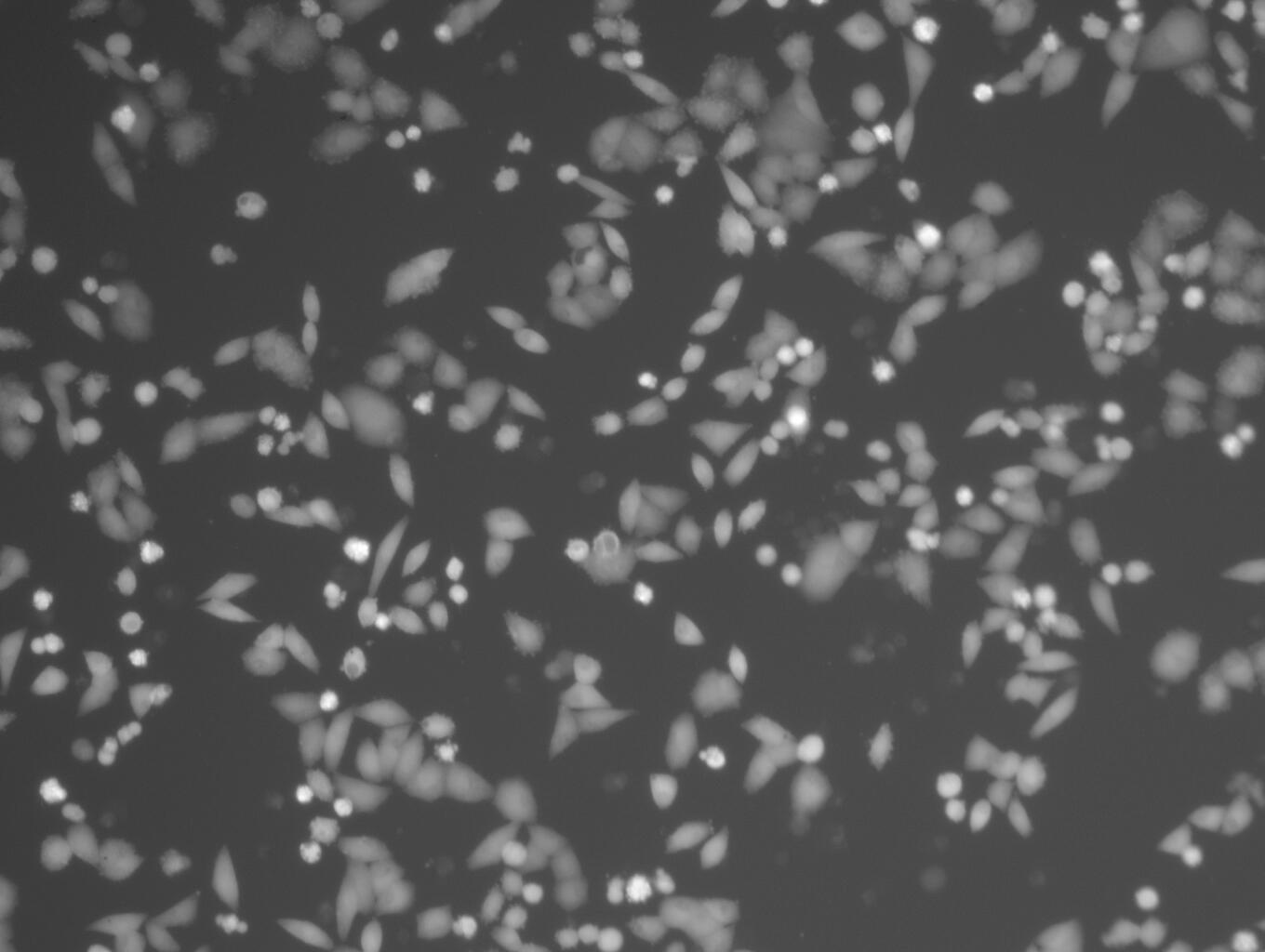

Supplement: Supplementary file 8 — Source data Fig. 6 [file 44318_2024_233_MOESM8_ESM.zip › 6D/Figure 6D Image/sgATG16L1 #1 AMDE-1 (Calcein-AM).jpg]

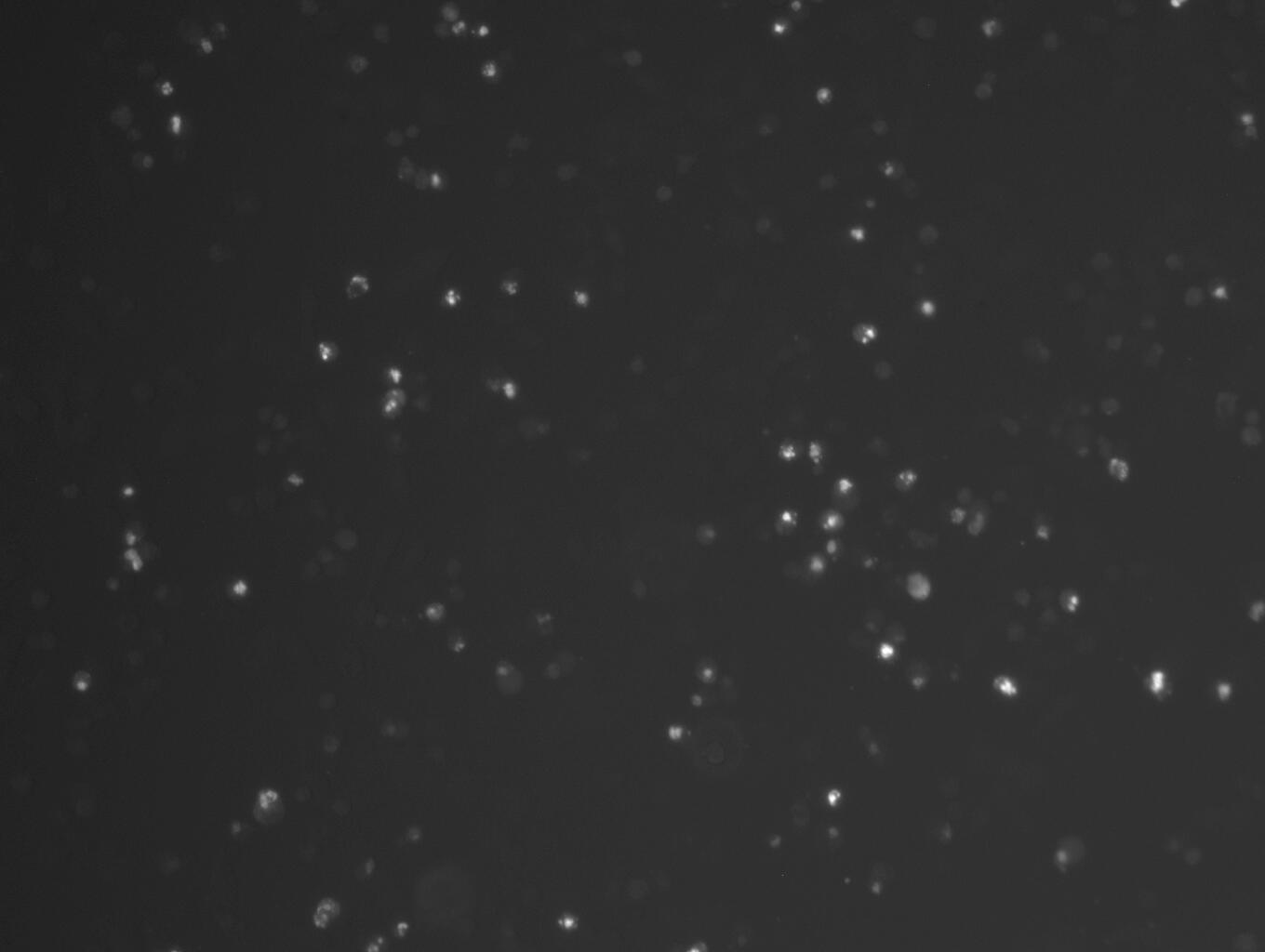

Supplement: Supplementary file 8 — Source data Fig. 6 [file 44318_2024_233_MOESM8_ESM.zip › 6D/Figure 6D Image/sgATG16L1 #1 AMDE-1 (PI).jpg]

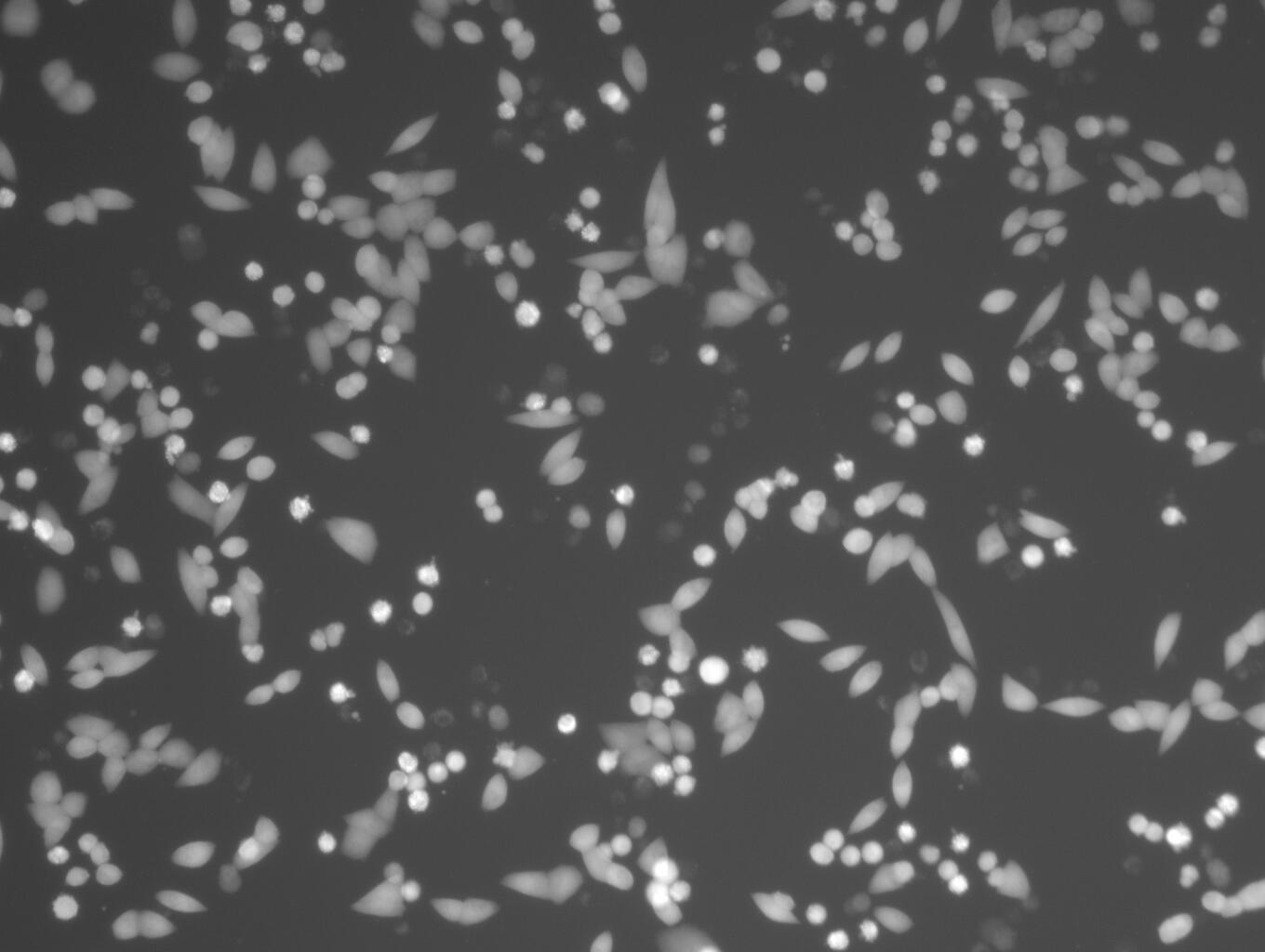

Supplement: Supplementary file 8 — Source data Fig. 6 [file 44318_2024_233_MOESM8_ESM.zip › 6D/Figure 6D Image/sgATG16L1 #1 niclosamide (Calcein-AM).jpg]

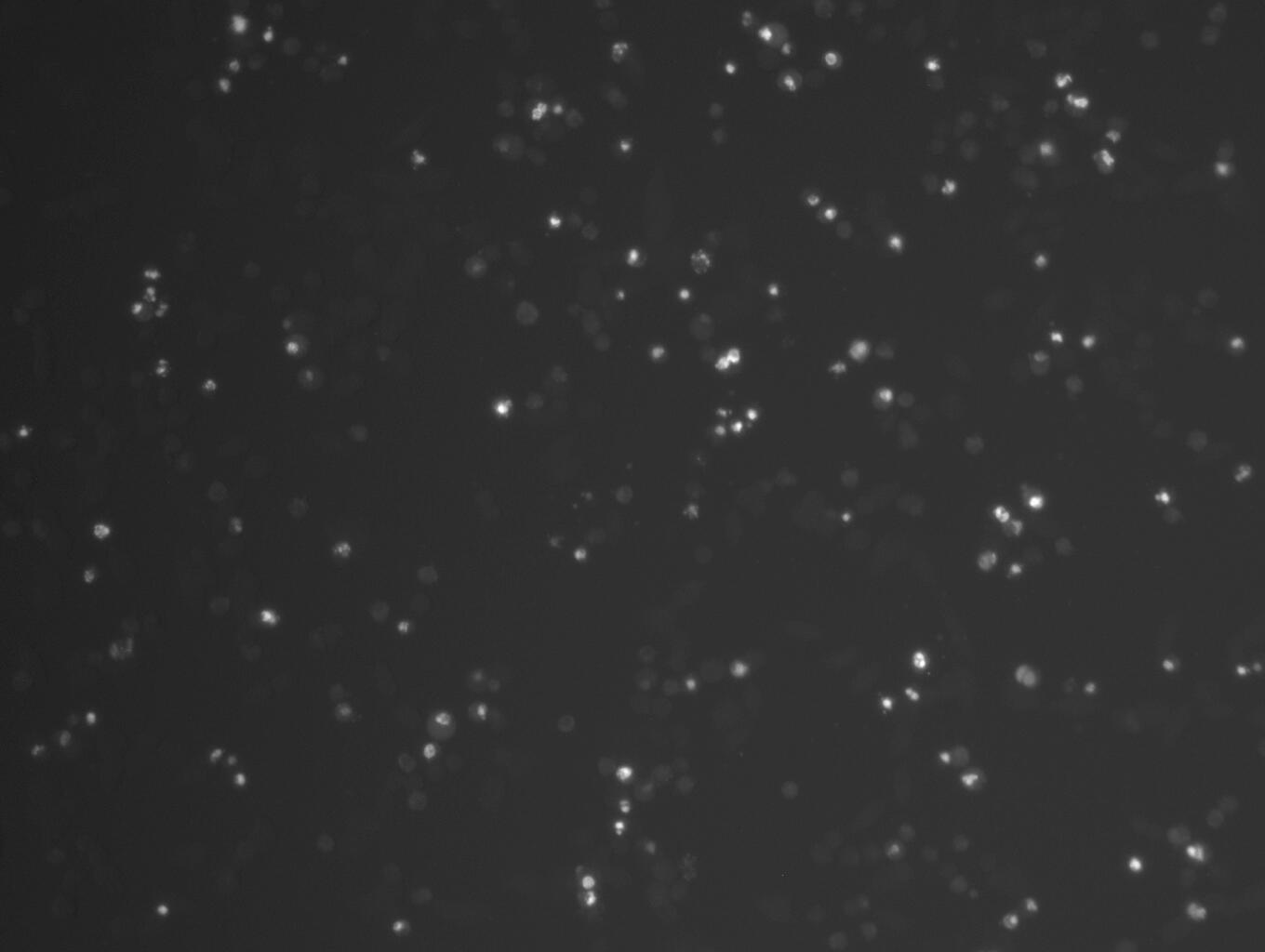

Supplement: Supplementary file 8 — Source data Fig. 6 [file 44318_2024_233_MOESM8_ESM.zip › 6D/Figure 6D Image/sgATG16L1 #1 niclosamide (PI).jpg]

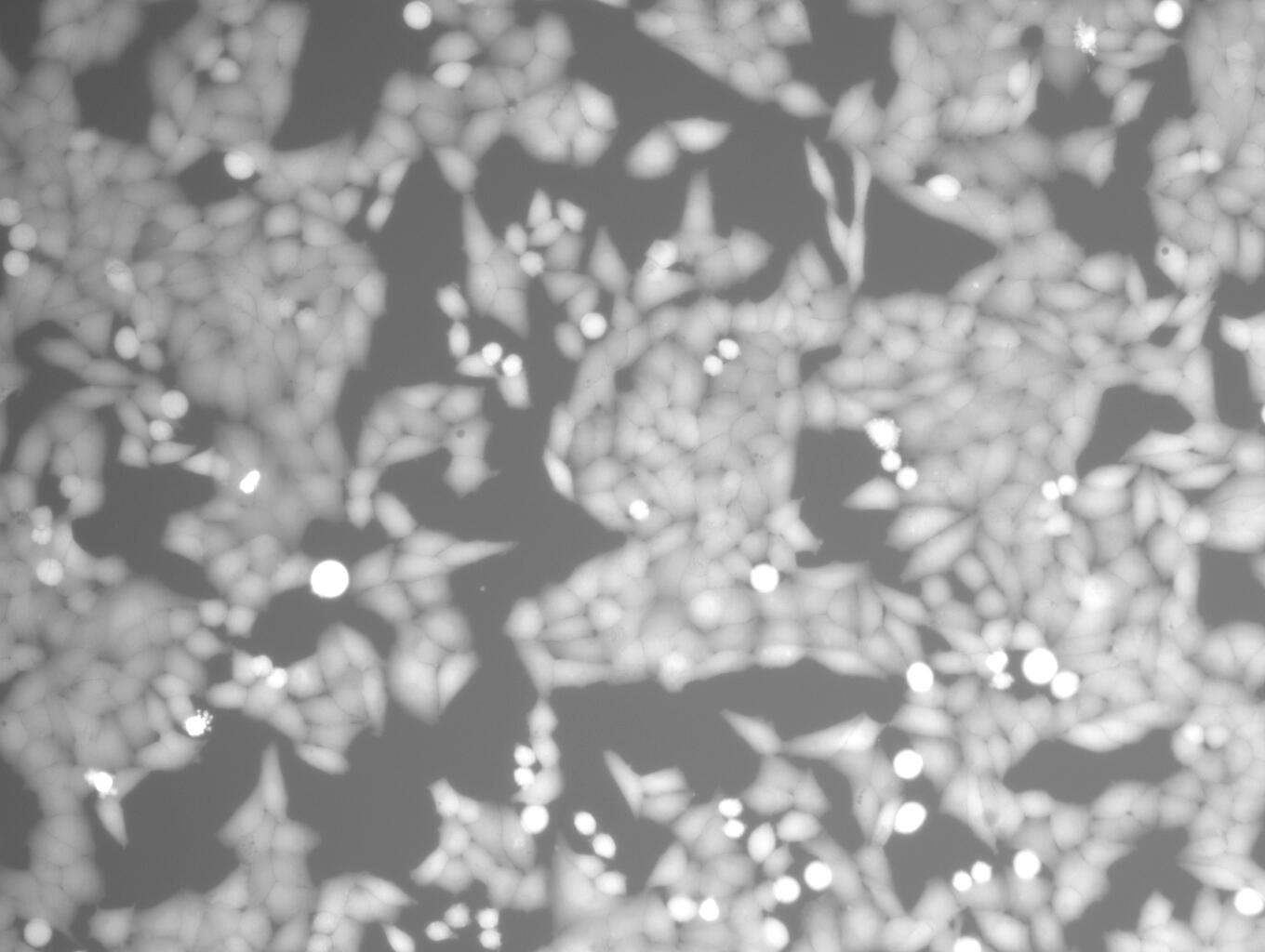

Supplement: Supplementary file 8 — Source data Fig. 6 [file 44318_2024_233_MOESM8_ESM.zip › 6D/Figure 6D Image/sgATG16L1 #1 veh (Calcein-AM).jpg]

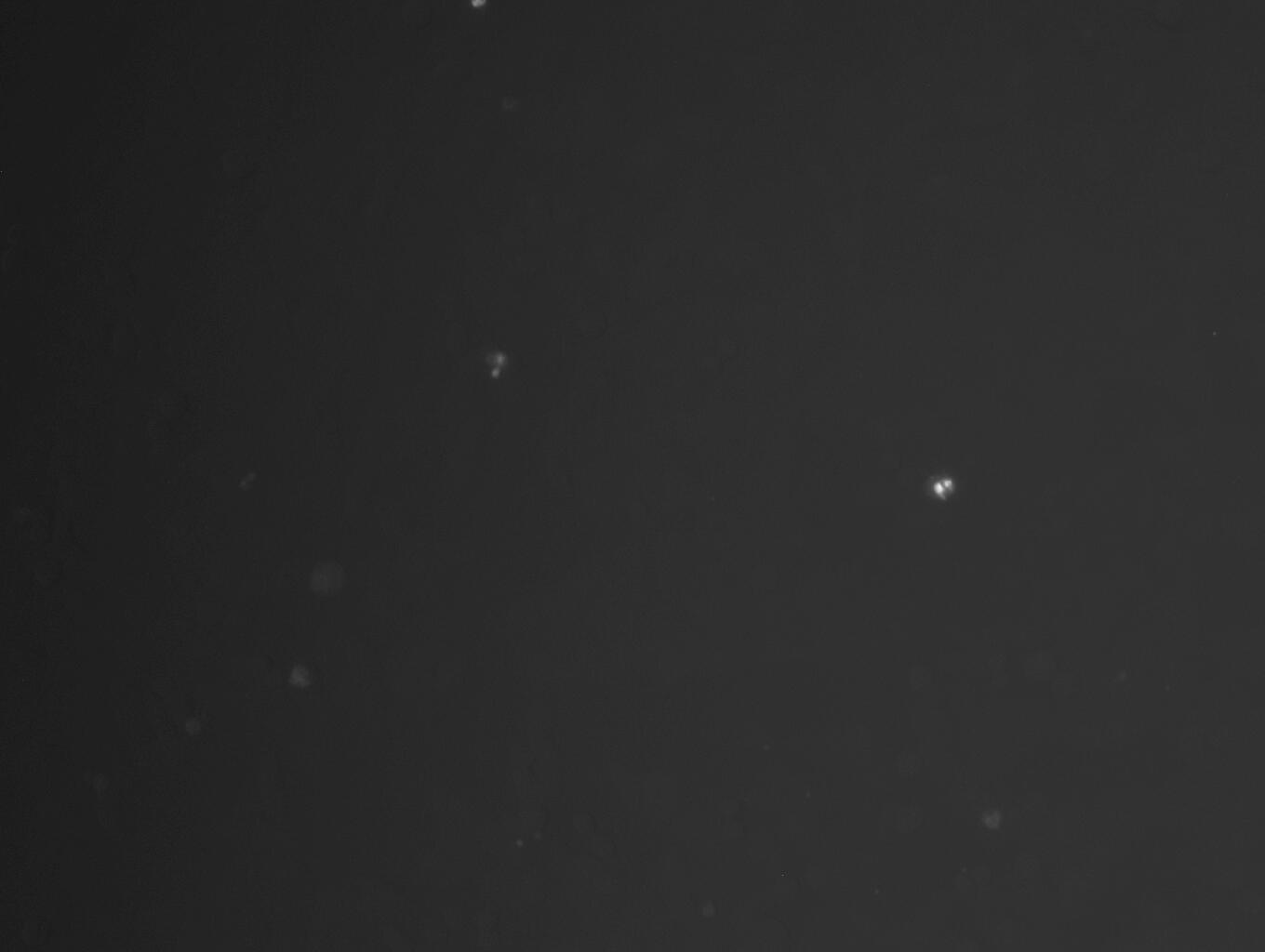

Supplement: Supplementary file 8 — Source data Fig. 6 [file 44318_2024_233_MOESM8_ESM.zip › 6D/Figure 6D Image/sgATG16L1 #1 veh (PI).jpg]

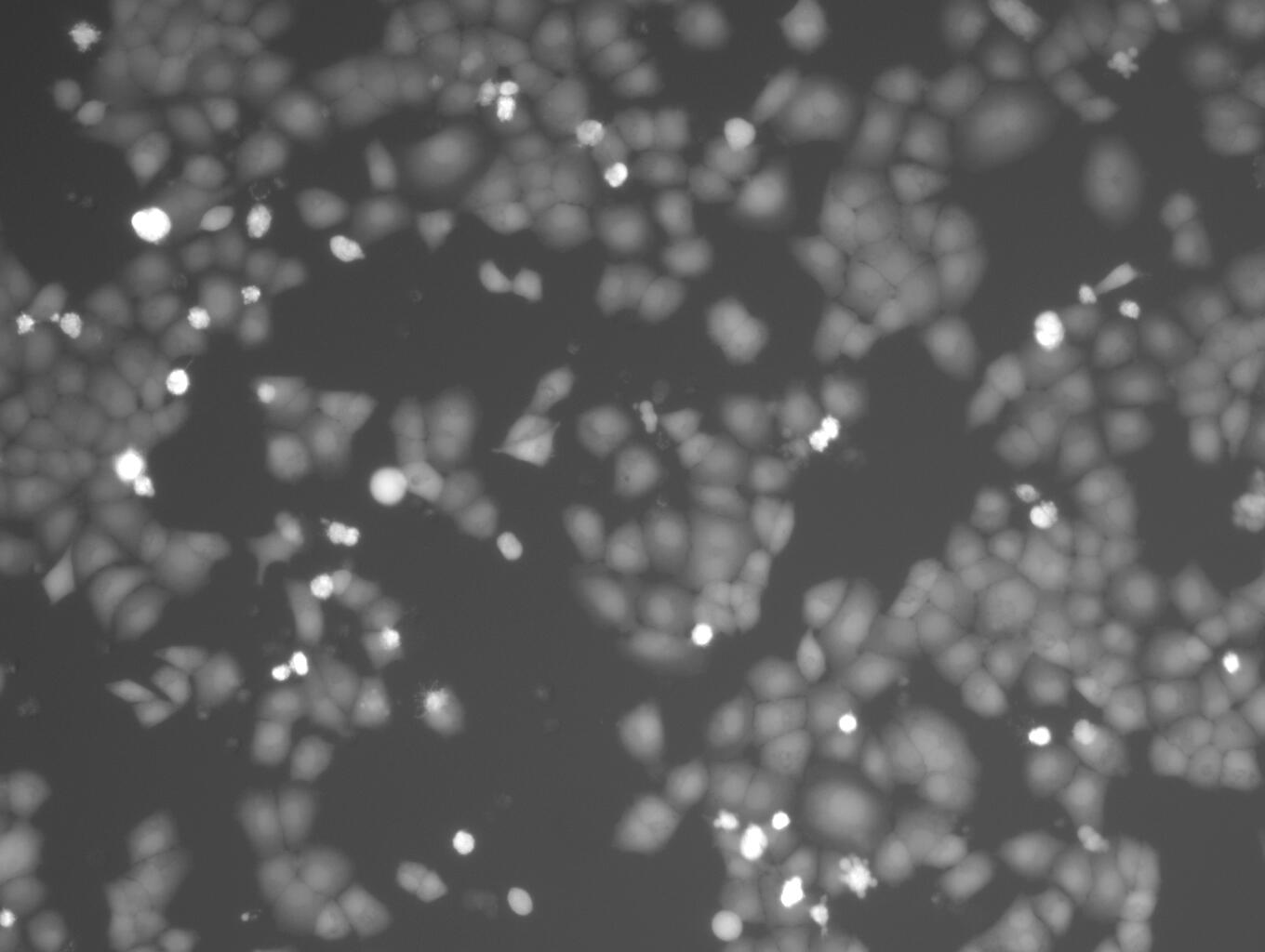

Supplement: Supplementary file 8 — Source data Fig. 6 [file 44318_2024_233_MOESM8_ESM.zip › 6D/Figure 6D Image/sgATG16L1 #2 AMDE-1 (niclosamide).jpg]

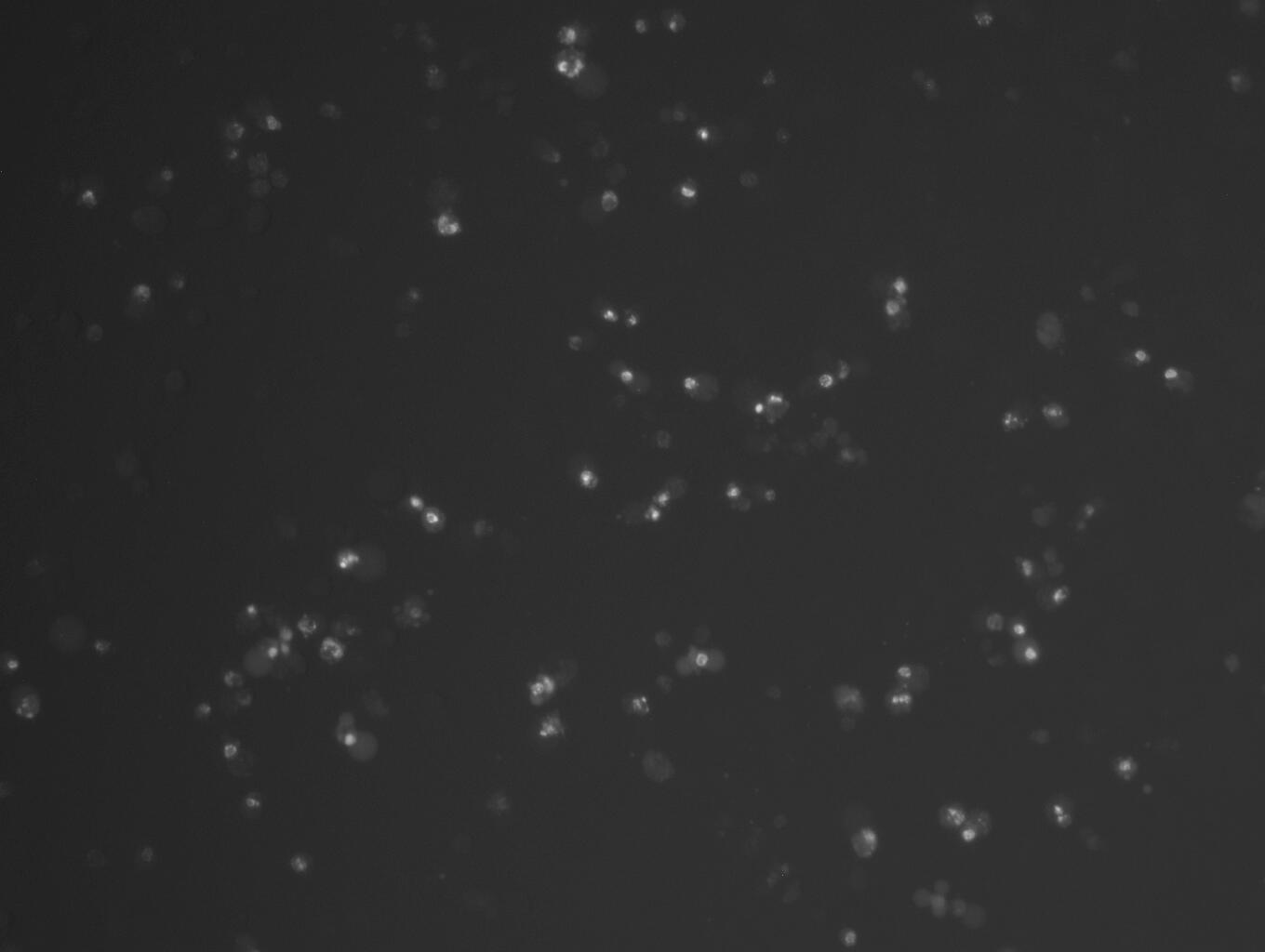

Supplement: Supplementary file 8 — Source data Fig. 6 [file 44318_2024_233_MOESM8_ESM.zip › 6D/Figure 6D Image/sgATG16L1 #2 AMDE-1 (PI).jpg]

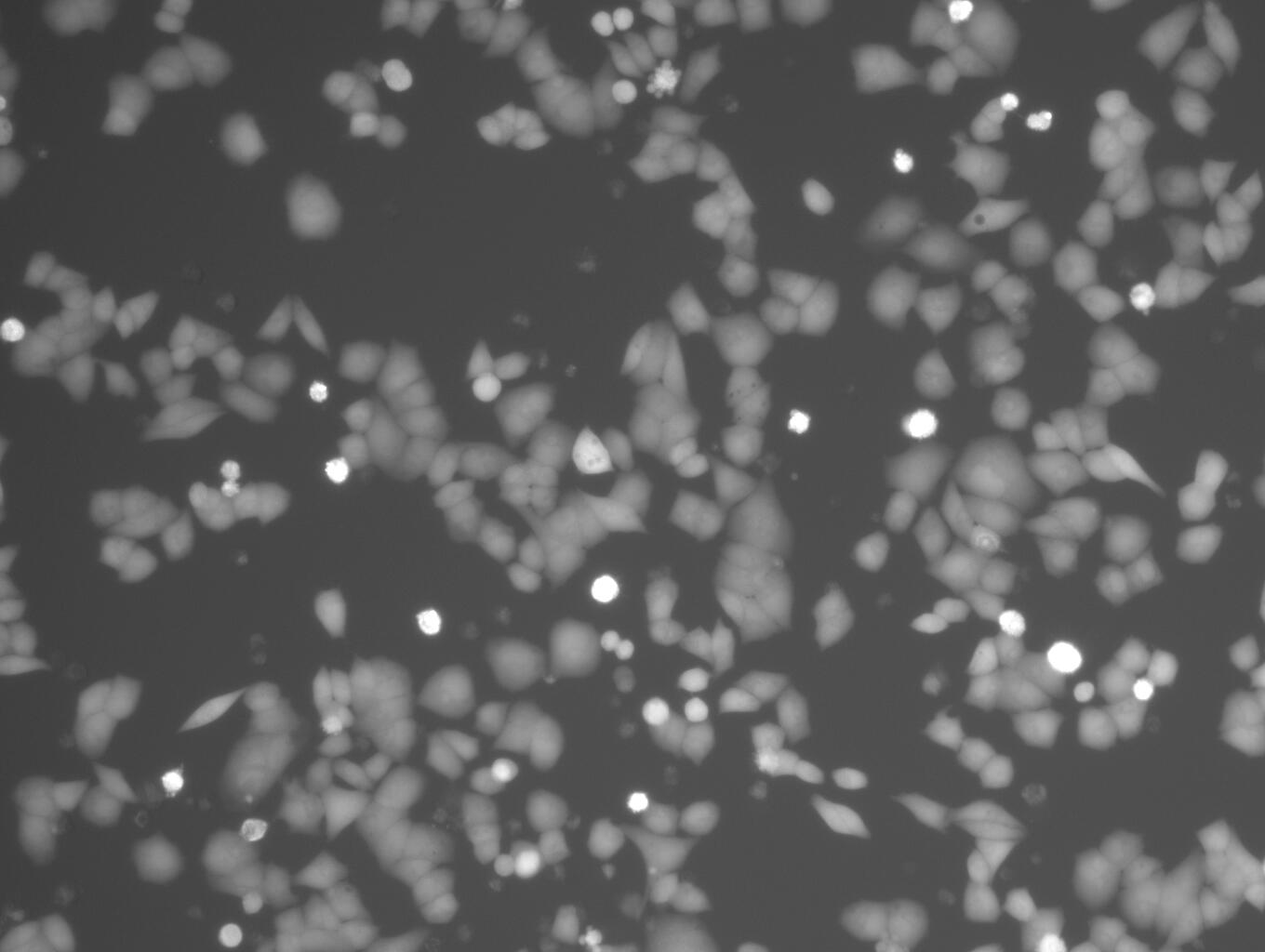

Supplement: Supplementary file 8 — Source data Fig. 6 [file 44318_2024_233_MOESM8_ESM.zip › 6D/Figure 6D Image/sgATG16L1 #2 niclosamide (Calcein-AM).jpg]

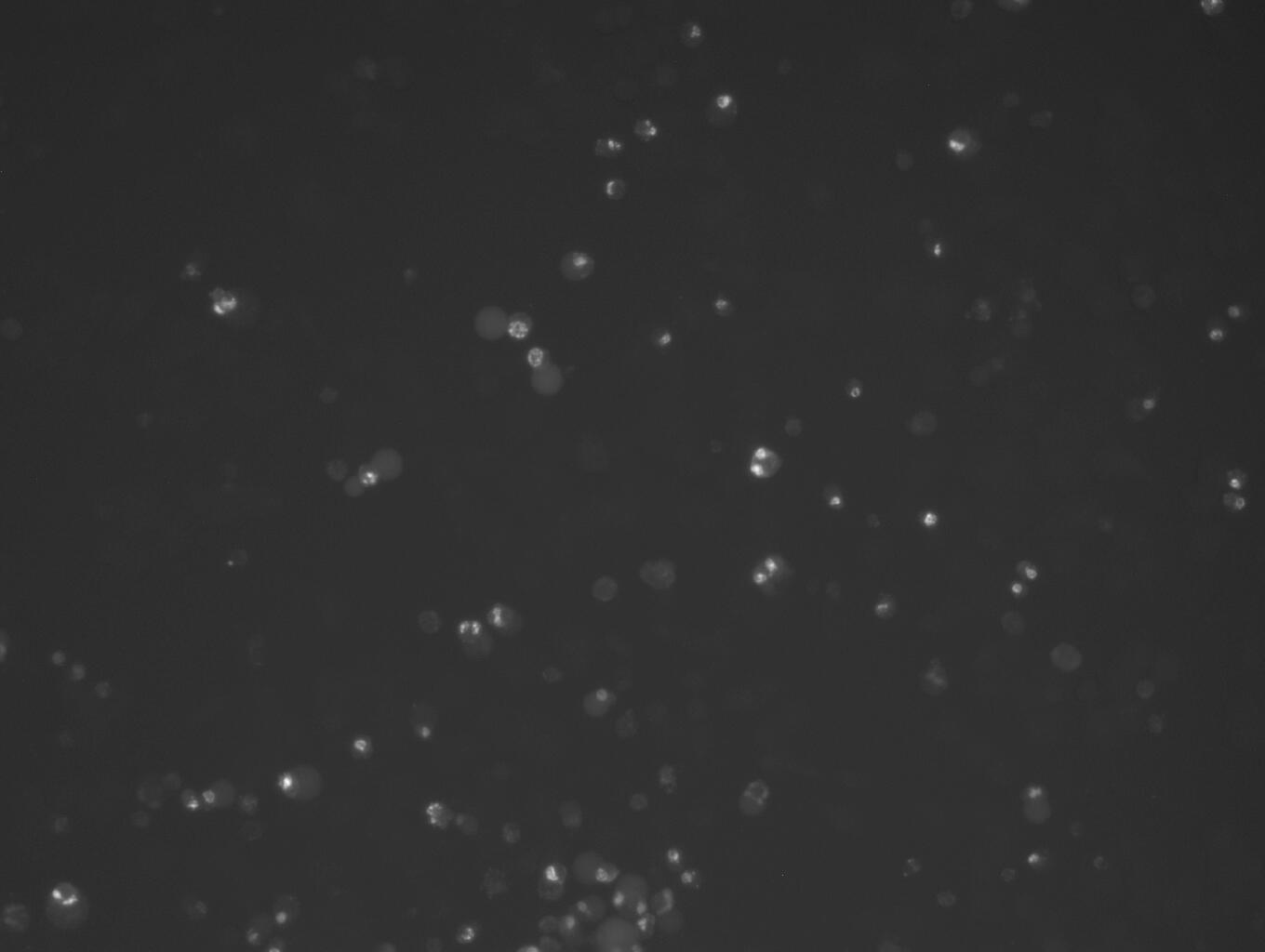

Supplement: Supplementary file 8 — Source data Fig. 6 [file 44318_2024_233_MOESM8_ESM.zip › 6D/Figure 6D Image/sgATG16L1 #2 niclosamide (PI).jpg]

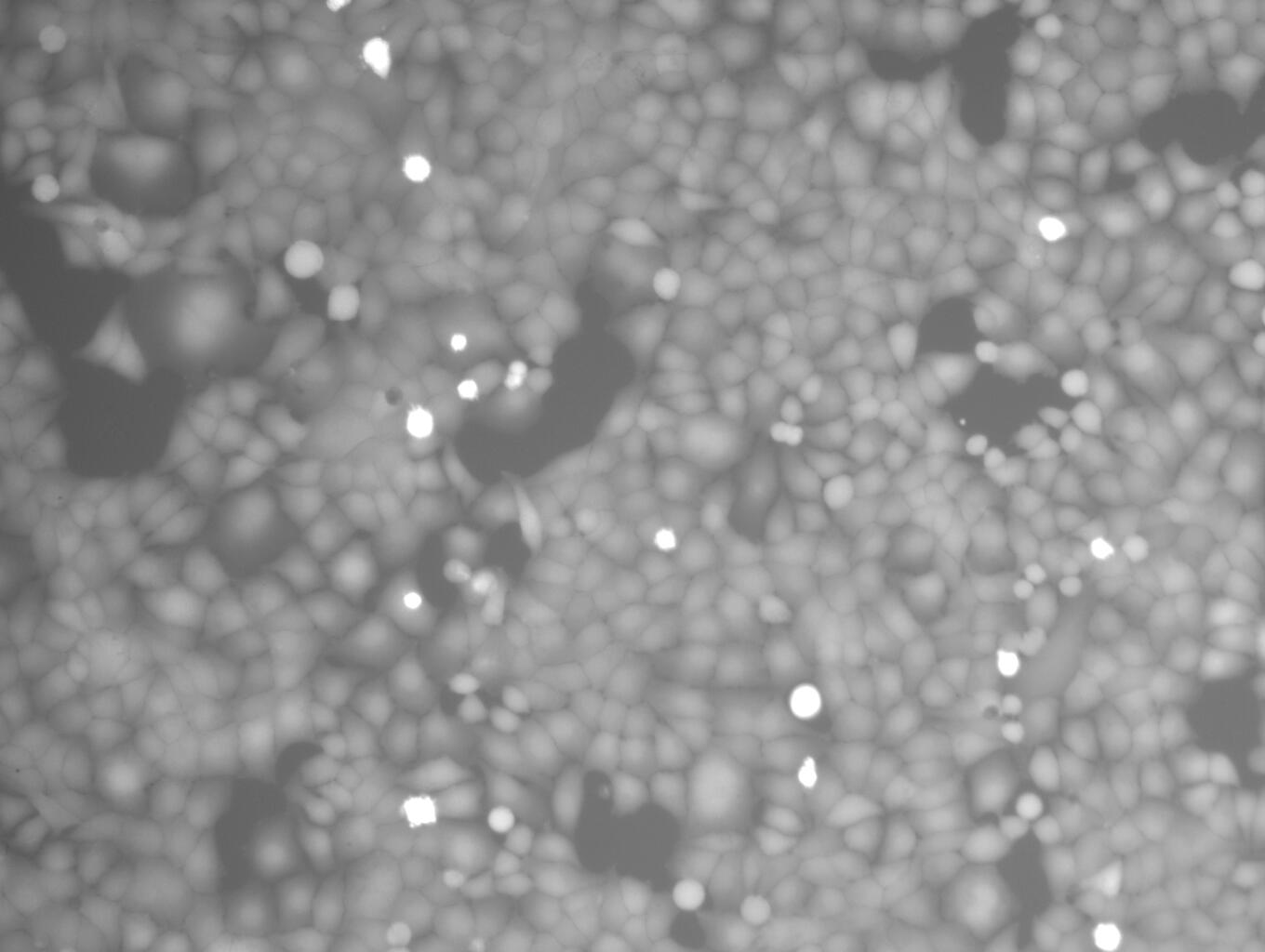

Supplement: Supplementary file 8 — Source data Fig. 6 [file 44318_2024_233_MOESM8_ESM.zip › 6D/Figure 6D Image/sgATG16L1 #2 veh (Calcein-AM).jpg]

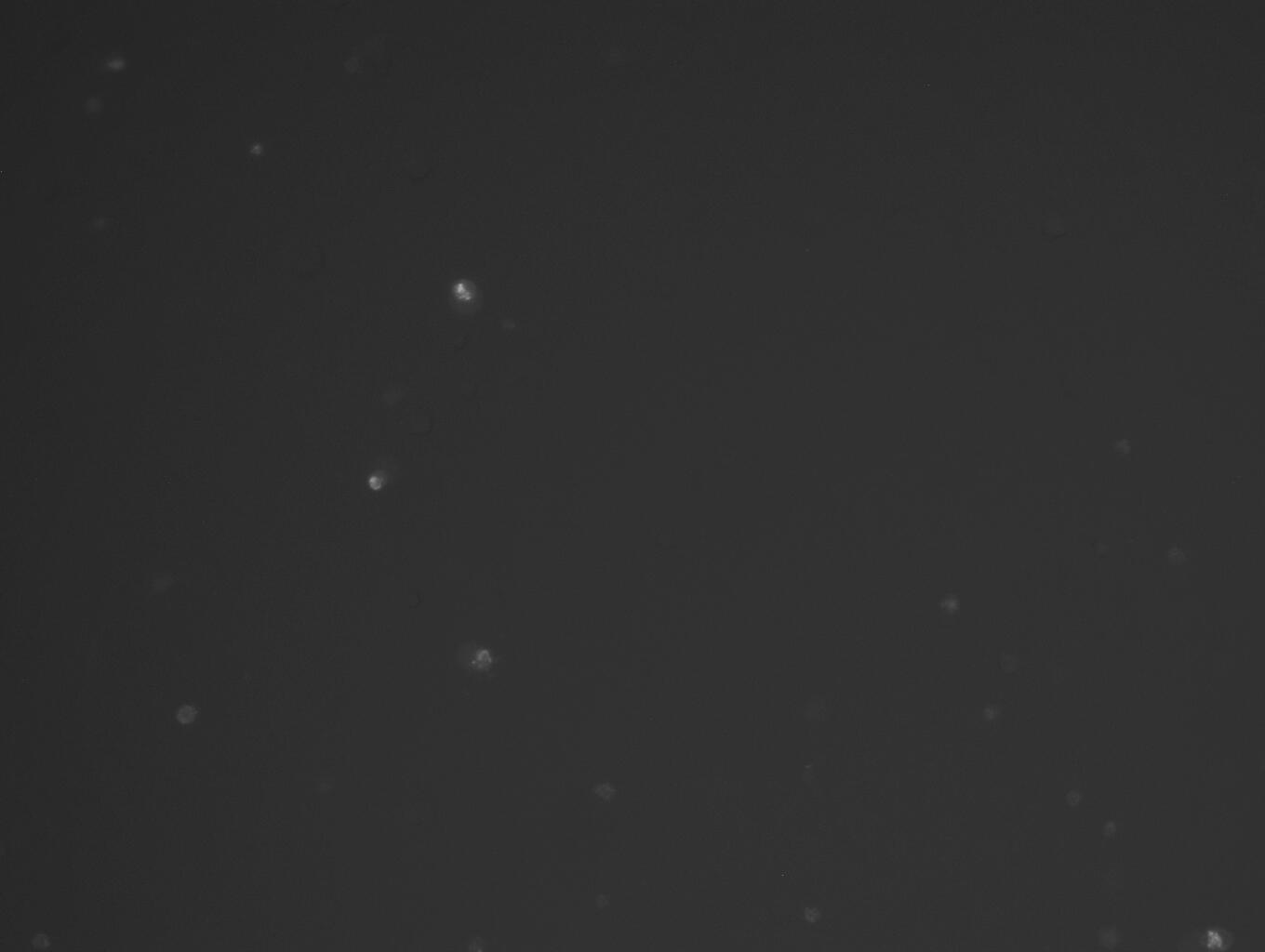

Supplement: Supplementary file 8 — Source data Fig. 6 [file 44318_2024_233_MOESM8_ESM.zip › 6D/Figure 6D Image/sgATG16L1 #2 veh (PI).jpg]

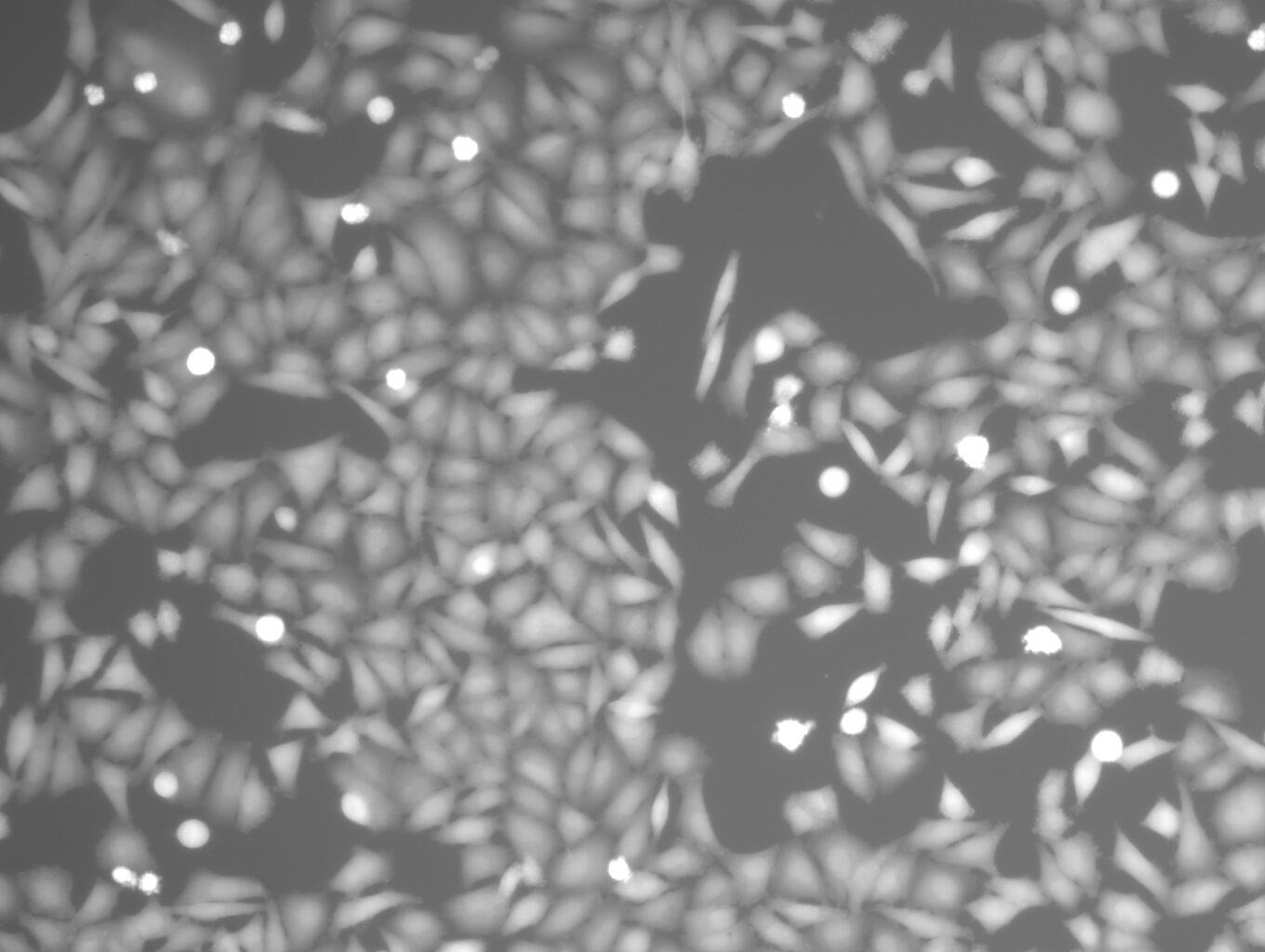

Supplement: Supplementary file 8 — Source data Fig. 6 [file 44318_2024_233_MOESM8_ESM.zip › 6D/Figure 6D Image/sgCtrl AMDE-1 (Calcein-AM).jpg]

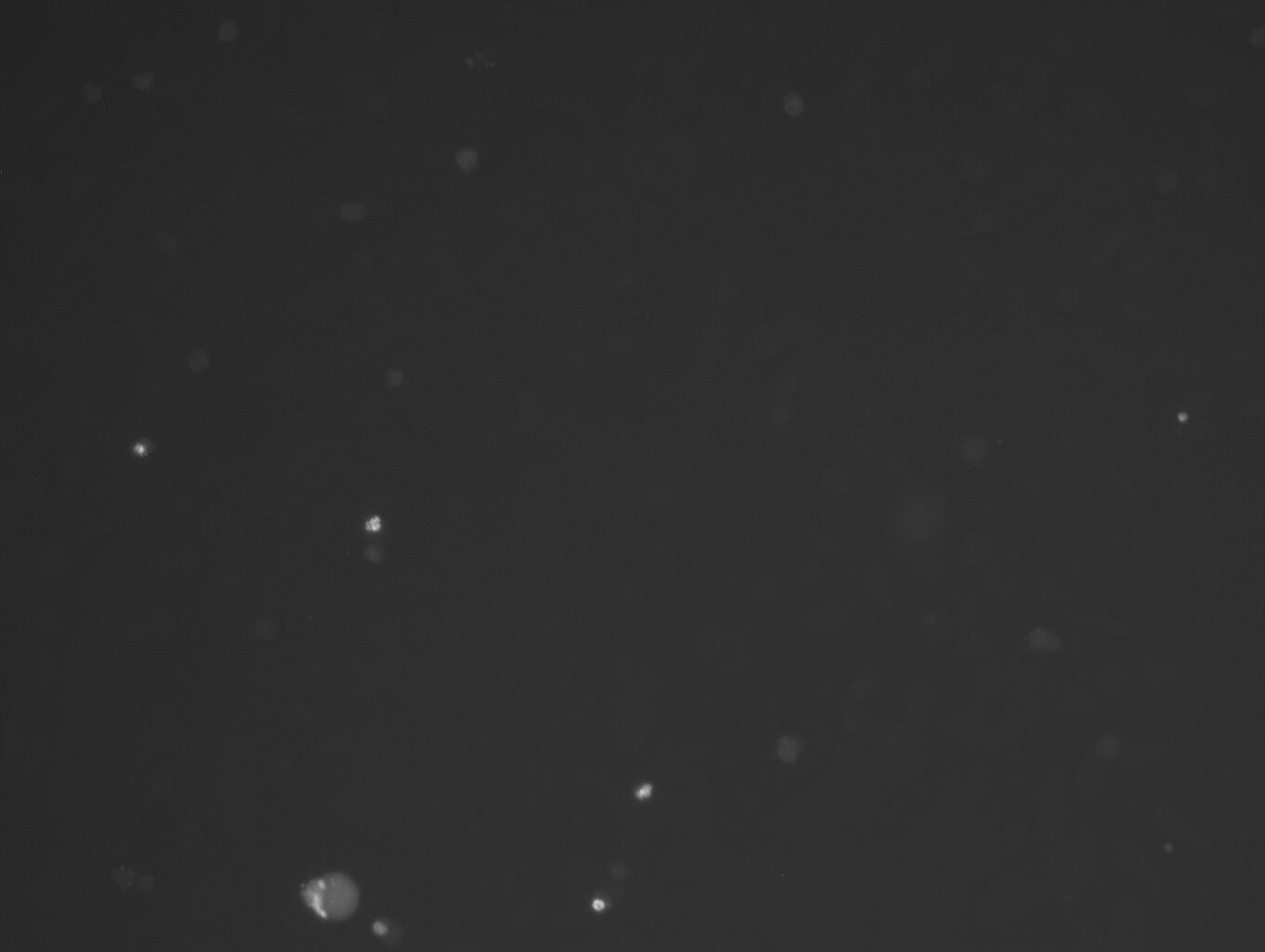

Supplement: Supplementary file 8 — Source data Fig. 6 [file 44318_2024_233_MOESM8_ESM.zip › 6D/Figure 6D Image/sgCtrl AMDE-1 (PI).jpg]

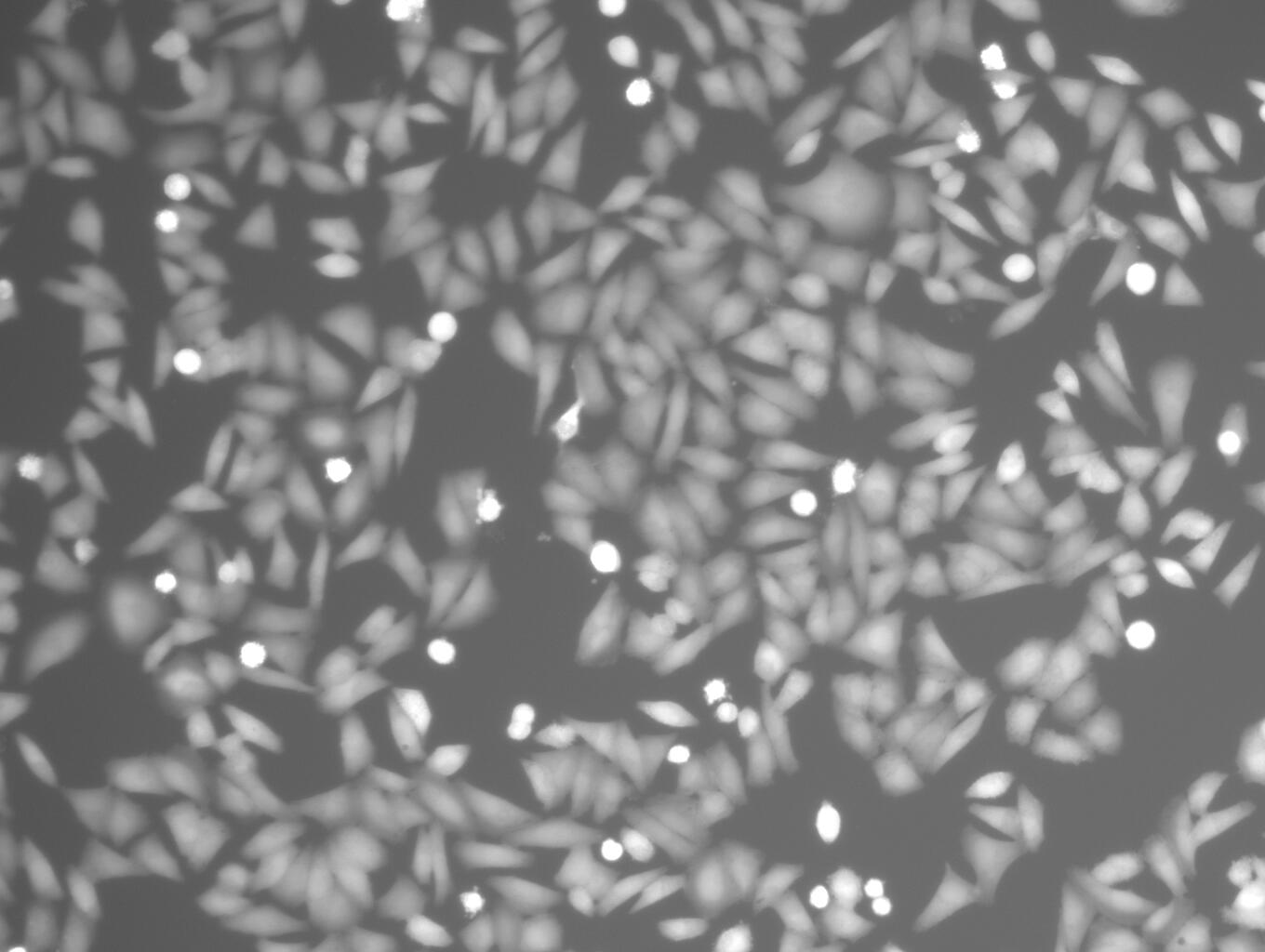

Supplement: Supplementary file 8 — Source data Fig. 6 [file 44318_2024_233_MOESM8_ESM.zip › 6D/Figure 6D Image/sgCtrl niclosamide (Calcein-AM).jpg]

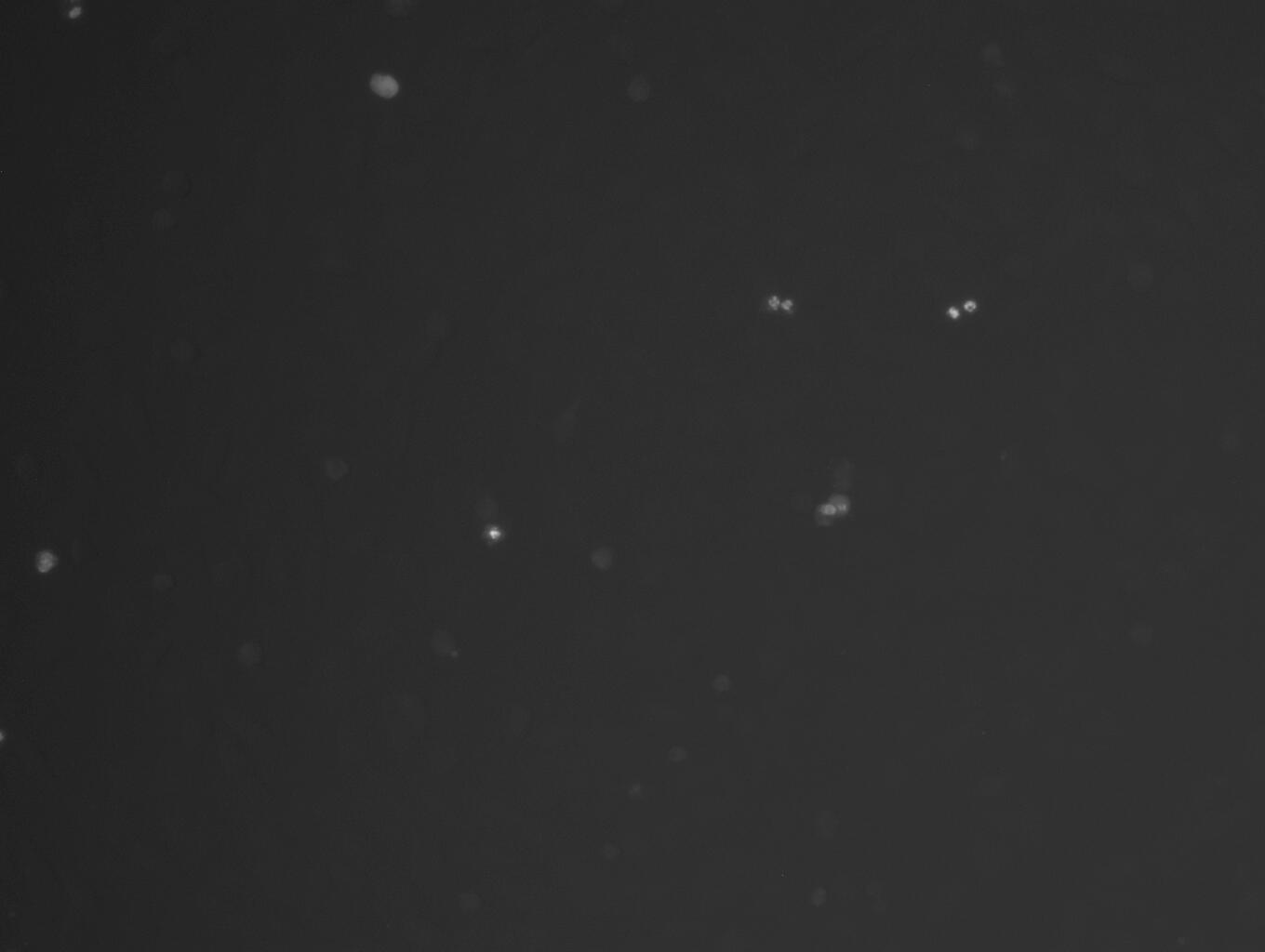

Supplement: Supplementary file 8 — Source data Fig. 6 [file 44318_2024_233_MOESM8_ESM.zip › 6D/Figure 6D Image/sgCtrl niclosamide (PI).jpg]

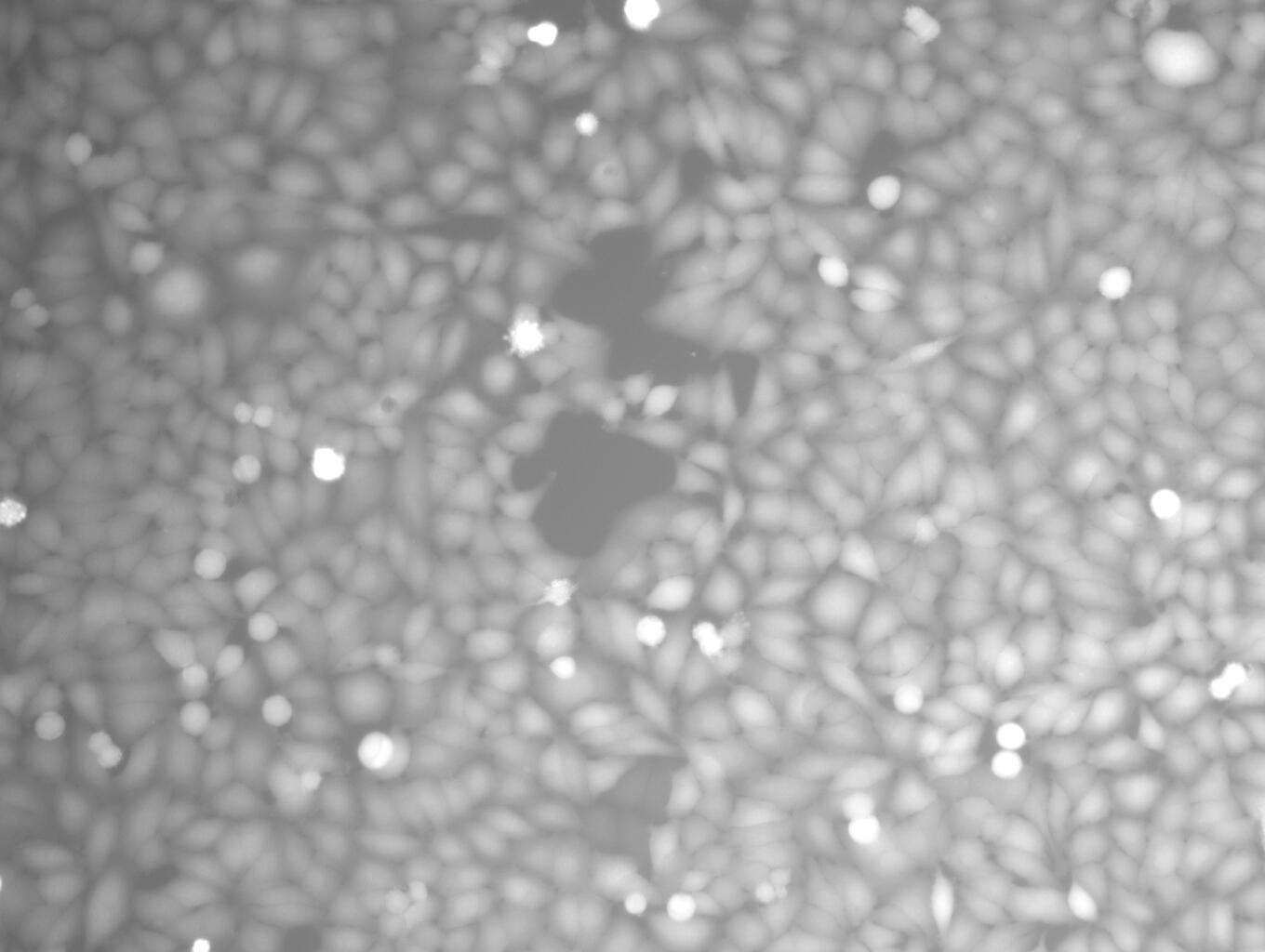

Supplement: Supplementary file 8 — Source data Fig. 6 [file 44318_2024_233_MOESM8_ESM.zip › 6D/Figure 6D Image/sgCtrl veh (Calcein-AM).jpg]

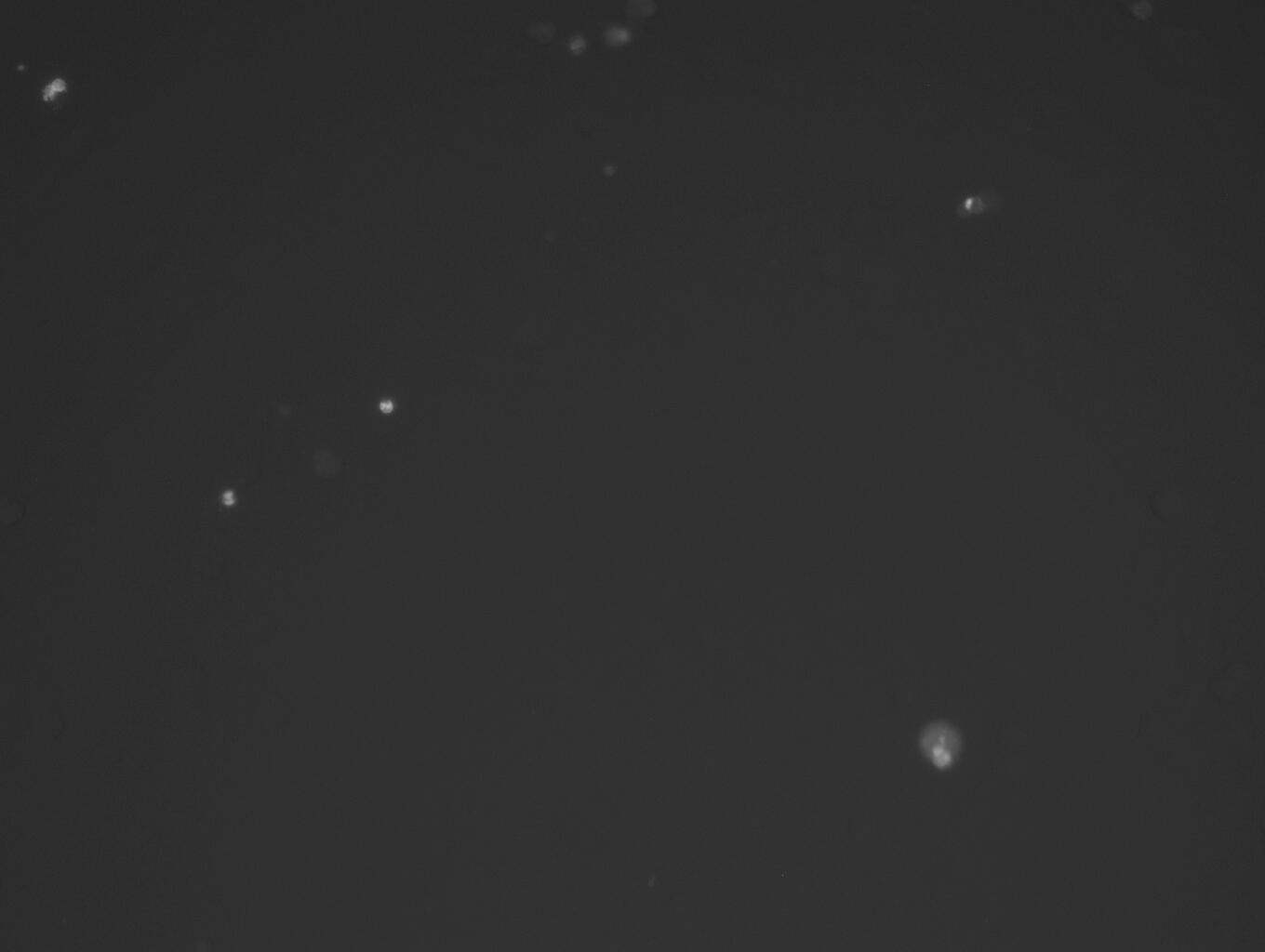

Supplement: Supplementary file 8 — Source data Fig. 6 [file 44318_2024_233_MOESM8_ESM.zip › 6D/Figure 6D Image/sgCtrl veh (PI).jpg]
