# Supplementary material for: Non-autophagic Golgi-LC3 lipidation facilitates TFE3 stress response against Golgi dysfunction
Source: EMBO J. 2024 Sep 16;43(21):5085–113. doi: 10.1038/s44318-024-00233-y (PMC11535212; doi:10.1038/s44318-024-00233-y)
Supplement: Supplementary file 9 — Expanded View Figures [file 44318_2024_233_MOESM9_ESM.pdf]

Expanded View Figures

Figure EV1. Overexpression of DLK1 Isoform 2, DLL1 and DLL3 does not induce LC3 accumulation on the *trans*-Golgi network.

(**A**) Transmission electron microscopy images of HeLa cells expressing pcDNA3-HA (Ctrl) or DLK1-HA. G, Golgi apparatus. Arrowheads indicate single-membraned vesicles associated with a *trans*-Golgi network. (**B**) Immunoblot analysis of HeLa cells expressing GFP-LC3B and either DLK1-HA WT or deletion mutants (left). Relative signals of GFP-LC3B-II and GFP-LC3B-I on the blots are represented as mean ± s.d. ($n = 5$ independent experiments, one-way ANOVA followed by Dunnett's multiple comparisons test) (right). (**C**) HeLa cells expressing TGOLN2-GFP and either DLK1-HA WT or mutants were immunostained with anti-DLK1 antibody and observed by confocal microscopy. Pearson's correlation coefficient of TGOLN2-GFP and DLK1 is represented as mean ± s.d. ($n = 3$, 91–125 cells per experiment, one-way ANOVA followed by Dunnett's multiple comparisons test, DLK1 WT vs DLK1 ΔEGF4; $p = 0.000014$). (**D**) HeLa cells expressing GFP-LC3B with either DLK1-HA, DLL1-Myc, or DLL3-Myc were observed by fluorescence microscopy (left). The percentages of cells with GFP-LC3B clusters are represented as mean ± s.d. ($n = 3$, 71–126 cells per experiment, one-way ANOVA followed by Tukey's multiple comparisons test, Ctrl vs DLK1; $p = 0.000000038$, DLK1 vs DLL1; $p = 0.000000052$, DLK1 vs DLL3; $p = 0.000000049$) (right). (**E**) Immunoblot analysis of HeLa cells expressing GFP-LC3B with either DLK1-HA, DLL1-Myc, or DLL3-Myc (left). Relative signals of GFP-LC3B-II to GFP-LC3B-I on the blots are represented as mean ± s.d. ($n = 3$ independent experiments, one-way ANOVA followed by Tukey's multiple comparisons test) (right).

 © The Author(s)

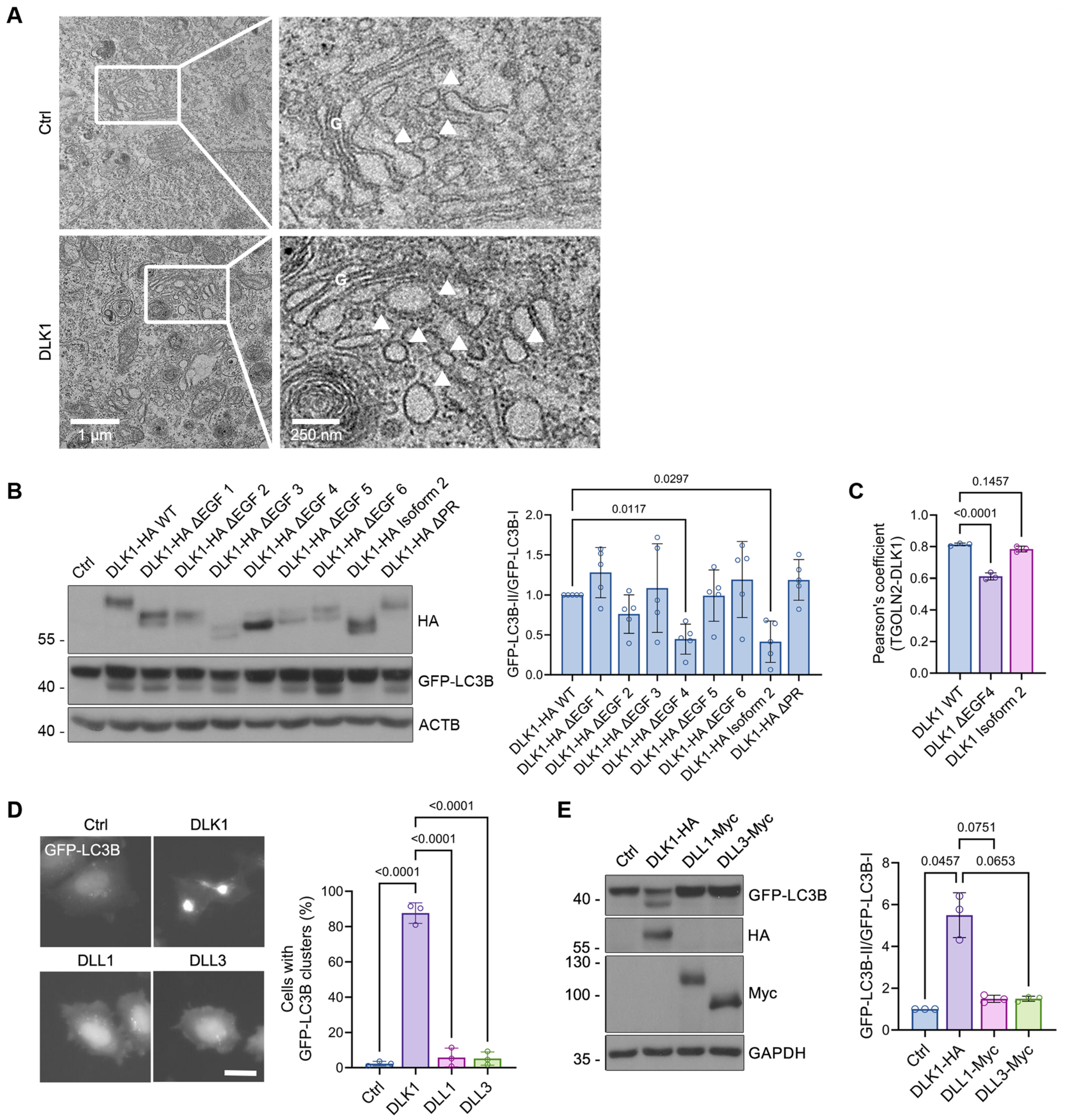
A
Ctrl
DLK1
G
1 µm
250 nm
B
Ctrl
DLK1-HA WT
DLK1-HA ΔEGF 1
DLK1-HA ΔEGF 2
DLK1-HA ΔEGF 3
DLK1-HA ΔEGF 4
DLK1-HA ΔEGF 5
DLK1-HA ΔEGF 6
DLK1-HA Isoform 2
DLK1-HA ΔPR
HA
GFP-LC3B
ACTB
55 -
40 -
40 -
0.0297
0.0117
GFP-LC3B-II/GFP-LC3B-I
C
0.1457
<0.0001
Pearson's coefficient (TGOLN2-DLK1)
DLK1 WT
DLK1 ΔEGF4
DLK1 Isoform 2
D
Ctrl
DLK1
GFP-LC3B
DLL1
DLL3
Cells with GFP-LC3B clusters (%)
<0.0001
<0.0001
<0.0001
Ctrl
DLK1
DLL1
DLL3
E
Ctrl
DLK1-HA
DLL1-Myc
DLL3-Myc
GFP-LC3B
HA
Myc
GAPDH
40 -
55 -
130 -
100 -
35 -
0.0751
0.0457
0.0653
GFP-LC3B-II/GFP-LC3B-I

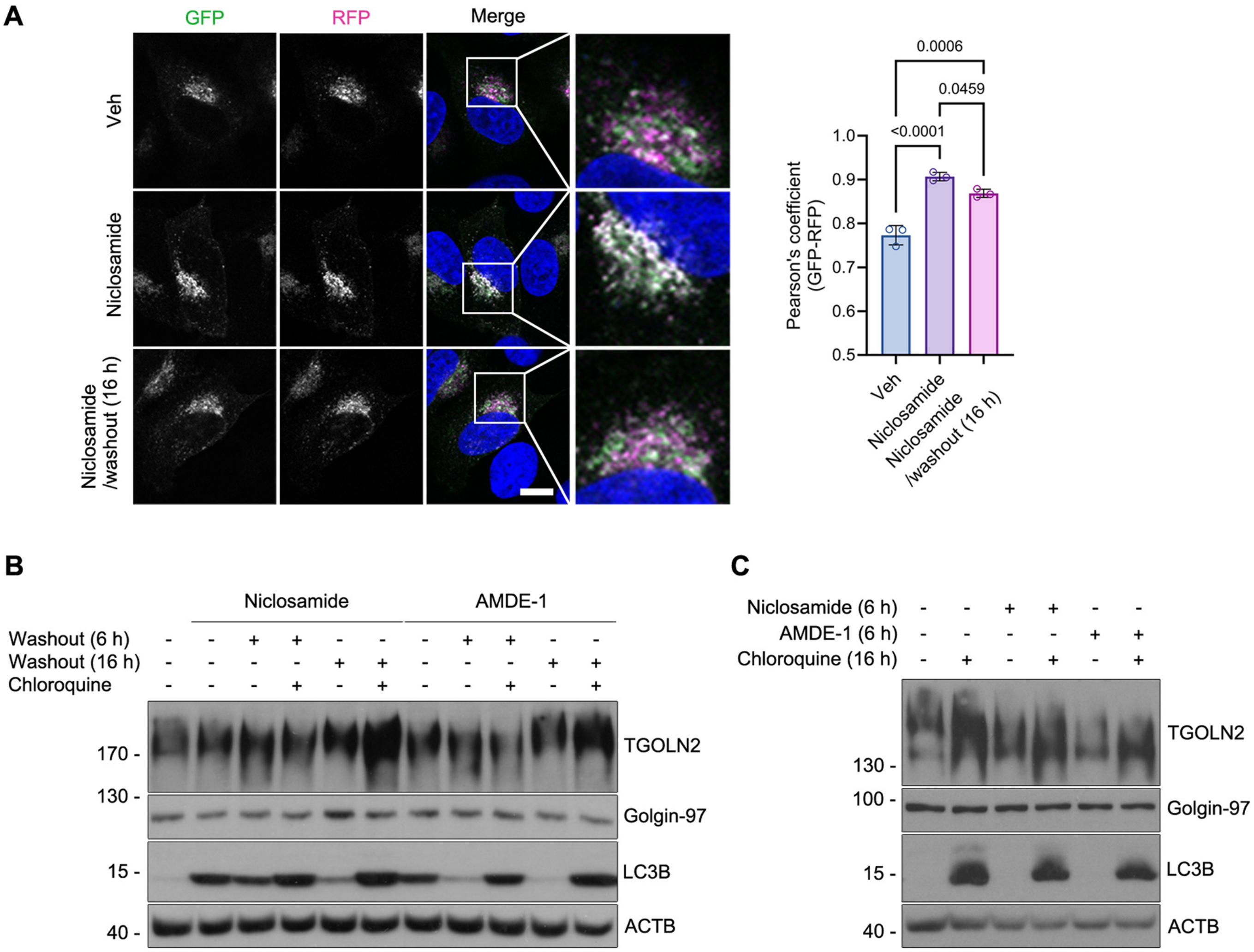

Figure EV2. LC3-lipidated Golgi membranes are not degraded by lysosomes.

(**A**) HeLa cells expressing TGOLN2-RFP-GFP were treated with 10 μM niclosamide for 6 h, and niclosamide was then removed for an additional 16 h. Cells were observed by confocal microscopy. Nuclei were stained by Hoechst dye 33342. Scale bar, 10 μm (left). Pearson's correlation coefficient of GFP and RFP is represented as mean ± s.d. ($n = 3$, 33–47 cells per experiment, one-way ANOVA followed by Tukey's multiple comparisons test, Veh vs Niclosamide; $p = 0.000084$) (right). (**B**, **C**) HeLa cells were treated with 10 μM niclosamide or 10 μM AMDE-1 for 6 h and niclosamide and AMDE-1 were removed for an additional 6 h (**B**) or 16 h (**B**, **C**) in the presence or absence of 50 μM chloroquine. Vehicle-treated cells were further incubated with 50 μM chloroquine for 16 h in (**C**). Cells were subjected to immunoblot analysis.

© The Author(s)

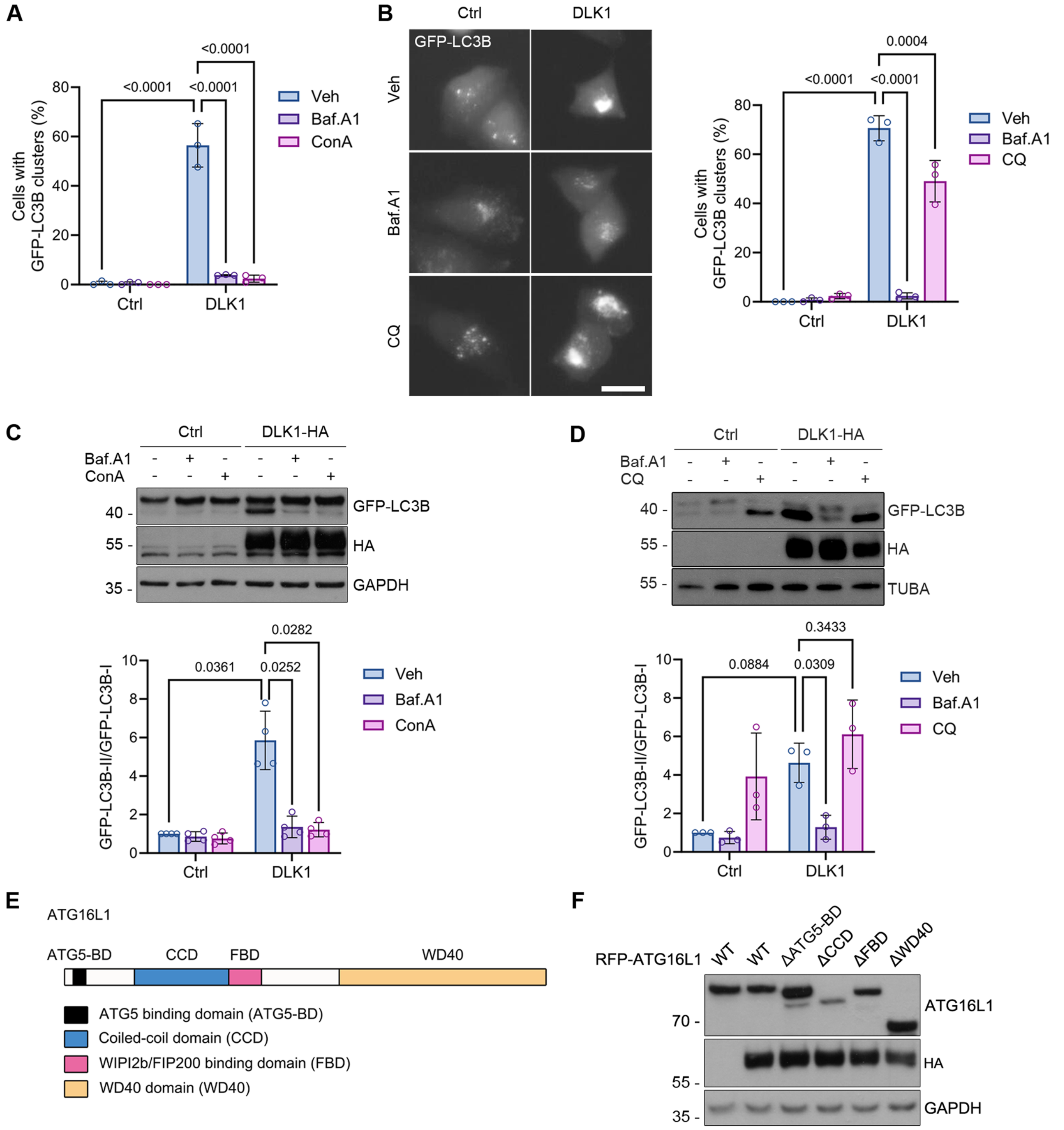
A
<0.0001
<0.0001
<0.0001
Cells with GFP-LC3B clusters (%)
Veh
Baf.A1
ConA
Ctrl
DLK1
B
Ctrl
DLK1
GFP-LC3B
Veh
Baf.A1
CQ
0.0004
<0.0001
<0.0001
Cells with GFP-LC3B clusters (%)
C
Ctrl
DLK1-HA
Baf.A1
ConA
GFP-LC3B
HA
GAPDH
0.0282
0.0361
0.0252
GFP-LC3B-II/GFP-LC3B-I
D
Ctrl
DLK1-HA
Baf.A1
CQ
GFP-LC3B
HA
TUBA
0.3433
0.0884
0.0309
GFP-LC3B-II/GFP-LC3B-I
E
ATG16L1
ATG5-BD
CCD
FBD
WD40
ATG5 binding domain (ATG5-BD)
Coiled-coil domain (CCD)
WIPI2b/FIP200 binding domain (FBD)
WD40 domain (WD40)
F
RFP-ATG16L1
WT
WT
ΔATG5-BD
ΔCCD
ΔFBD
ΔWD40
ATG16L1
HA
GAPDH

Figure EV3. V-ATPase mediates Golgi-LC3 lipidation.

(**A**) HeLa cells expressing DLK1-HA and GFP-LC3B were treated with 20 nM bafilomycin A1 (Baf.A1) or 200 nM concanamycin A (ConA) for 6 h and observed by fluorescence microscopy. The percentages of cells with GFP-LC3B clusters are represented as mean ± s.d. ($n = 3$, 90–199 cells per experiment, two-way ANOVA followed by Tukey's multiple comparisons test, Ctrl/Veh vs DLK1/Veh; $p = 0.0000000041$, DLK1/Veh vs Baf.A1; $p = 0.0000000079$, DLK1/Veh vs ConA; $p = 0.0000000060$). (**B**) HeLa cells expressing DLK1-HA and GFP-LC3B were treated with 20 nM bafilomycin A1 (Baf.A1) or 100 μM chloroquine (CQ) for 6 h and observed by fluorescence microscopy. Scale bar, 20 μm (left). The percentages of cells with GFP-LC3B clusters are represented as mean ± s.d. ($n = 3$, 139–240 cells per experiment, two-way ANOVA followed by Tukey's multiple comparisons test, Ctrl/Veh vs DLK1/Veh; $p = 0.0000000010$, DLK1/Veh vs Baf.A1; $p = 0.0000000015$) (right). (**C**, **D**) HeLa cells expressing DLK1-HA and GFP-LC3B were treated with 20 nM bafilomycin A1 (Baf.A1) or either 200 nM concanamycin A (ConA) (**C**) or 100 μM chloroquine (CQ) (**D**) for 6 h and subjected to immunoblot analysis (top). Relative signals of GFP-LC3B-II and GFP-LC3B-I on the blots are represented as mean ± s.d. [$n = 4$ independent experiments (**C**), $n = 3$ independent experiments (**D**), two-way ANOVA followed by Tukey's multiple comparisons test] (bottom). (**E**) Schematic representation of ATG16L1 domains. (**F**) Immunoblot analysis of HeLa sgATG16L1 cells expressing DLK1-HA and RFP-ATG16L1 mutants.

 © The Author(s)

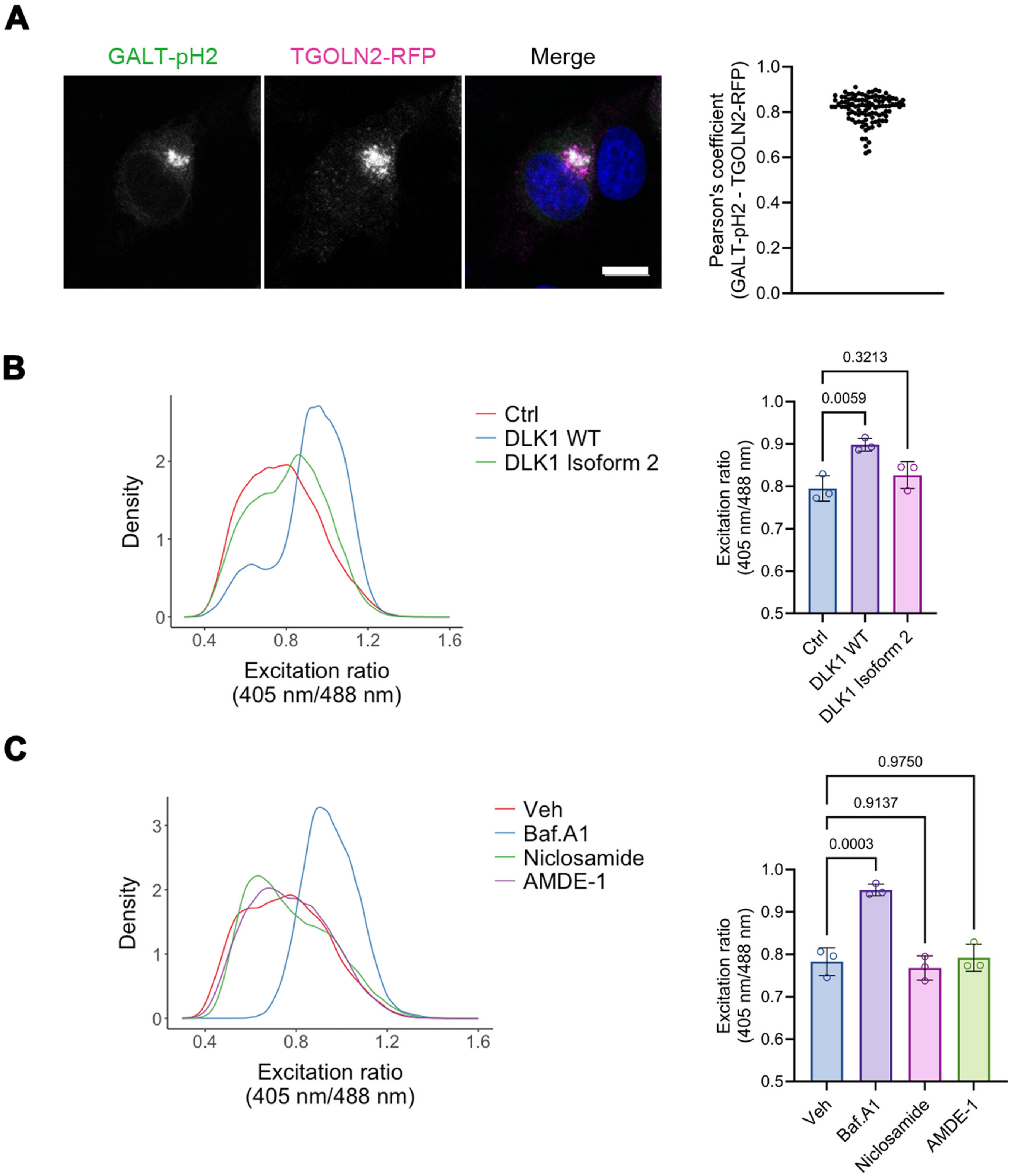
A
GALT-pH2
TGOLN2-RFP
Merge
Pearson's coefficient (GALT-pH2 - TGOLN2-RFP)
1.0
0.8
0.6
0.4
0.2
0.0
B
Ctrl
DLK1 WT
DLK1 Isoform 2
Density
Excitation ratio (405 nm/488 nm)
0.4
0.8
1.2
1.6
0.3213
0.0059
Excitation ratio (405 nm/488 nm)
Ctrl
DLK1 WT
DLK1 Isoform 2
C
Veh
Baf.A1
Niclosamide
AMDE-1
Density
Excitation ratio (405 nm/488 nm)
0.9750
0.9137
0.0003
Excitation ratio (405 nm/488 nm)
Veh
Baf.A1
Niclosamide
AMDE-1

Figure EV4. DLK1, but not niclosamide and AMDE-1, increases Golgi pH.

(**A**) Confocal images of HeLa cells expressing GALT-pH2 and TGOLN2-RFP. Nuclei were stained by Hoechst dye 33342. Scale bar, 10 µm (left). Pearson's correlation coefficient of GALT-pH2 and TGOLN2-RFP ($n = 104$ cells) was quantified (right). (**B**, **C**) HeLa cells expressing GALT-pH2 and either pcDNA3-HA (Ctrl), DLK1-HA WT, or Isoform 2 were subjected to flow cytometry analysis (**B**). HeLa cells expressing GALT-pH2 were treated with 20 nM bafilomycin A1 (Baf.A1), 10 µM niclosamide, or 10 µM AMDE-1 for 6 h and subjected to flow cytometry analysis (**C**). Frequency distributions of excitation ratios (405/408 nm) are shown (left). Median values of the excitation ratios are represented as mean ± s.d. ($n = 3$ independent experiments, one-way ANOVA followed by Tukey's multiple comparisons test) (right).

 © The Author(s)

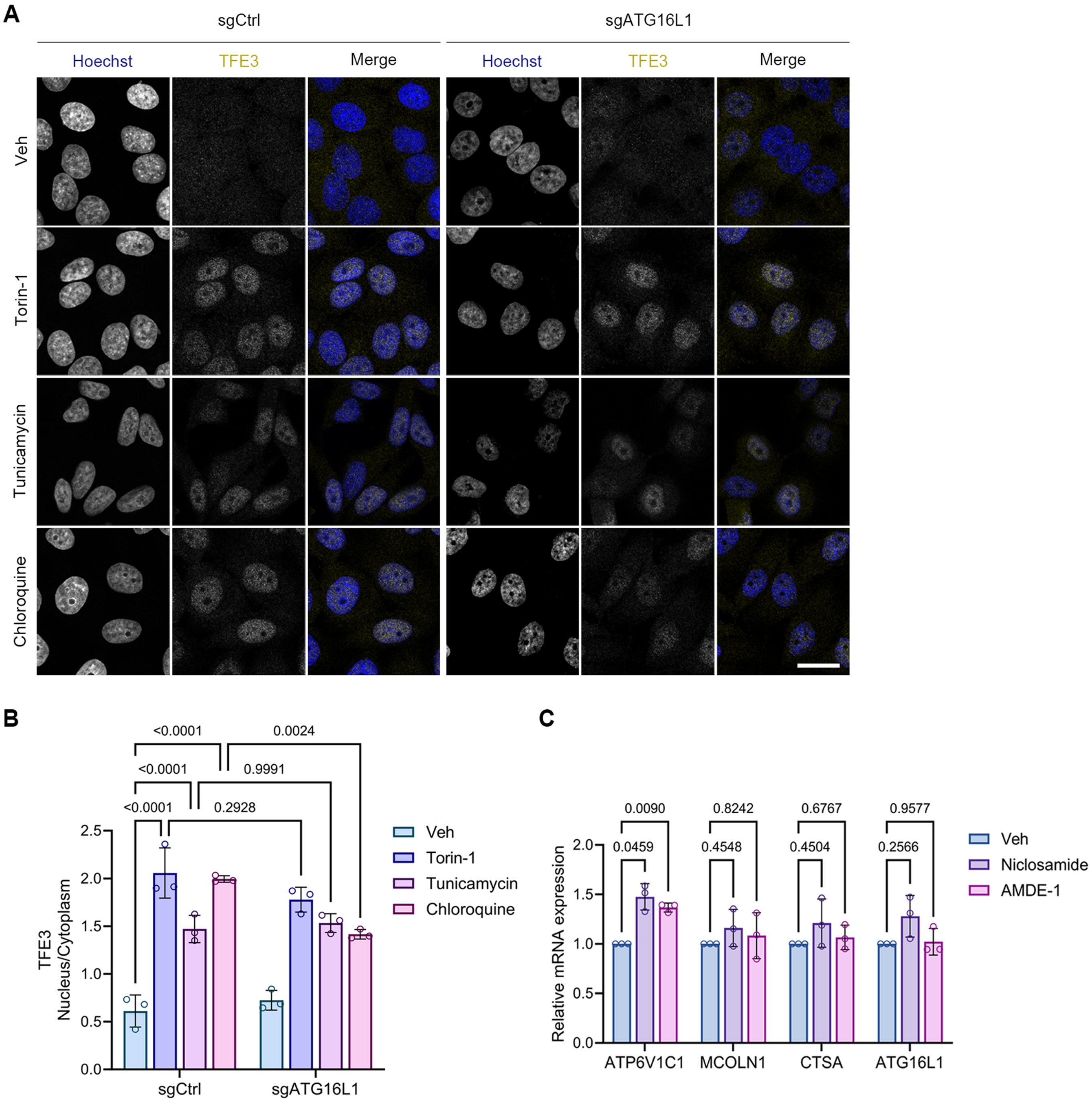

Figure EV5. Golgi stress-specific function of LC3 lipidation in TFE3 regulation.

(**A**, **B**) Confocal images of HeLa sgCtrl and sgATG16L1 cells incubated with 250 nM Torin-1 (1 h), 2 mg/ml tunicamycin (16 h), or 50 µM chloroquine (2 h) and immunostained with anti-TFE3 antibody. Nuclei were stained by Hoechst dye 33342. Scale bar, 20 µm (**A**). The nucleus/cytoplasm ratio of TFE3 fluorescence intensity is represented as mean ± s.d. ($n = 3$, 109–176 cells per experiment, two-way ANOVA followed by Tukey's multiple comparisons test, sgCtrl/Veh vs Torin-1; $p = 0.000000024$, sgCtrl/Veh vs Tunicamycin; $p = 0.000029472$, sgCtrl/Veh vs Chloroquine; $p = 0.000000046$) (**B**). (**C**) RNA of HeLa cells exposed to 10 µM niclosamide or 10 µM AMDE-1 for 6 h was analyzed with quantitative real-time PCR. Bars represent mean ± s.d. ($n = 3$ independent experiments, one-way ANOVA followed by Dunnett's multiple comparisons test).
